# Supplementary material for: A distant global control region is essential for normal expression of anterior HOXA genes during mouse and human craniofacial development
Source: Nat Commun. 2024 Jan 2;15:136. doi: 10.1038/s41467-023-44506-2 (PMC10762089; doi:10.1038/s41467-023-44506-2)
Supplement: Supplementary file 18 — Supplemental Table 14 [file 41467_2023_44506_MOESM18_ESM.pdf]

Mendeliome panel

**versie**	v5 (4732 genen)	Centrum voor Medische Genetica Gent

Gene	OMIM gene ID	Associated phenotype, OMIM phenotype ID, phenotype mapping key and inheritance pattern
A2ML1	610627	{Otitis media, susceptibility to}, 166760 (3), Autosomal dominant
A4GALT	607922	[Blood group, P1Pk system, P(2) phenotype], 111400 (3); NOR polyagglutination syndrome, 111400 (3); [Blood group, P1Pk system, p phenotype], 111400 (3)
AAAS	605378	Achalasia-addisonianism-alacrimia syndrome, 231550 (3), Autosomal recessive
AAGAB	614888	Keratoderma, palmoplantar, punctate type IA, 148600 (3), Autosomal dominant
AARS1	601065	Developmental and epileptic encephalopathy 29, 616339 (3), Autosomal recessive; Charcot-Marie-Tooth disease, axonal, type 2N, 613287 (3), Autosomal dominant; ?Leukoencephalopathy, hereditary diffuse, with spheroids 2, 619661 (3), Autosomal dominant; Trichothiodystrophy 8, nonphotosensitive, 619691 (3), Autosomal recessive
AARS2	612035	Leukoencephalopathy, progressive, with ovarian failure, 615889 (3), Autosomal recessive; Combined oxidative phosphorylation deficiency 8, 614096 (3), Autosomal recessive
AASS	605113	Hyperlysinemia, 238700 (3), Autosomal recessive
ABAT	137150	GABA-transaminase deficiency, 613163 (3), Autosomal recessive
ABCA1	600046	Tangier disease, 205400 (3), Autosomal recessive; HDL deficiency, familial, 1, 604091 (3), Autosomal dominant
ABCA12	607800	Ichthyosis, congenital, autosomal recessive 4B (harlequin), 242500 (3), Autosomal recessive; Ichthyosis, congenital, autosomal recessive 4A, 601277 (3), Autosomal recessive
ABCA2	600047	Intellectual developmental disorder with poor growth and with or without seizures or ataxia, 618808 (3), Autosomal recessive
ABCA3	601615	Surfactant metabolism dysfunction, pulmonary, 3, 610921 (3), Autosomal recessive
ABCA4	601691	Retinal dystrophy, early-onset severe, 248200 (3), Autosomal recessive; Retinitis pigmentosa 19, 601718 (3), Autosomal recessive; {Macular degeneration, age-related, 2}, 153800 (3), Autosomal dominant; Cone-rod dystrophy 3, 604116 (3), Autosomal recessive; Fundus flavimaculatus, 248200 (3), Autosomal recessive; Stargardt disease 1, 248200 (3), Autosomal recessive
ABCA5	612503	?Hypertrichosis, congenital generalized, with gingival hyperplasia, 135400 (3), Autosomal recessive
ABCA7	605414	{Alzheimer disease 9, susceptibility to}, 608907 (3), Autosomal dominant

ABCB1	171050	{Inflammatory bowel disease 13}, 612244 (3); {Colchicine resistance}, 120080 (3)
ABCB11	603201	Cholestasis, benign recurrent intrahepatic, 2, 605479 (3), Autosomal recessive; Cholestasis, progressive familial intrahepatic 2, 601847 (3), Autosomal recessive
ABCB4	171060	Gallbladder disease 1, 600803 (3), Autosomal dominant, Autosomal recessive; Cholestasis, intrahepatic, of pregnancy, 3, 614972 (3), Autosomal dominant, Autosomal recessive; Cholestasis, progressive familial intrahepatic 3, 602347 (3), Autosomal recessive
ABCB6	605452	Microphthalmia, isolated, with coloboma 7, 614497 (3), Autosomal dominant; Dyschromatosis universalis hereditaria 3, 615402 (3), Autosomal dominant; [Blood group, Langereis system], 111600 (3); Pseudohyperkalemia, familial, 2, due to red cell leak, 609153 (3), Autosomal dominant
ABCB7	300135	Anemia, sideroblastic, with ataxia, 301310 (3), X-linked recessive
ABCC1	158343	?Deafness, autosomal dominant 77, 618915 (3), Autosomal dominant
ABCC11	607040	[Axillary odor, variation in], 117800 (3), Autosomal dominant; [Earwax, wet/dry], 117800 (3), Autosomal dominant; [Colostrum secretion, variation in], 117800 (3), Autosomal dominant
ABCC2	601107	Dubin-Johnson syndrome, 237500 (3), Autosomal recessive
ABCC6	603234	Pseudoxanthoma elasticum, 264800 (3), Autosomal recessive; Arterial calcification, generalized, of infancy, 2, 614473 (3), Autosomal recessive; Pseudoxanthoma elasticum, forme fruste, 177850 (3), Autosomal dominant
ABCC8	600509	Diabetes mellitus, permanent neonatal 3, with or without neurologic features, 618857 (3), Autosomal dominant, Autosomal recessive; Diabetes mellitus, transient neonatal 2, 610374 (3); Diabetes mellitus, noninsulin-dependent, 125853 (3), Autosomal dominant; Hypoglycemia of infancy, leucine-sensitive, 240800 (3), Autosomal dominant; Hyperinsulinemic hypoglycemia, familial, 1, 256450 (3), Autosomal dominant, Autosomal recessive
ABCC9	601439	Cardiomyopathy, dilated, 1O, 608569 (3), Autosomal dominant; Hypertrichotic osteochondrodysplasia (Cantu syndrome), 239850 (3), Autosomal dominant; ?Atrial fibrillation, familial, 12, 614050 (3), Autosomal dominant; Intellectual disability and myopathy syndrome, 619719 (3), Autosomal recessive
ABCD1	300371	Adrenoleukodystrophy, 300100 (3), X-linked recessive; Adrenomyeloneuropathy, adult, 300100 (3), X-linked recessive
ABCD3	170995	?Bile acid synthesis defect, congenital, 5, 616278 (3), Autosomal recessive
ABCD4	603214	Methylmalonic aciduria and homocystinuria, cblJ type, 614857 (3), Autosomal recessive
ABCG2	603756	[Junior blood group system], 614490 (3); [Uric acid concentration, serum, QTL1], 138900 (3), ?Autosomal dominant
ABCG5	605459	Sitosterolemia 2, 618666 (3), Autosomal recessive

ABCG8	605460	Sitosterolemia 1, 210250 (3), Autosomal recessive; {Gallbladder disease 4}, 611465 (3)
ABHD12	613599	Polyneuropathy, hearing loss, ataxia, retinitis pigmentosa, and cataract, 612674 (3), Autosomal recessive
ABHD16A	142620	Spastic paraplegia 86, autosomal recessive, 619735 (3), Autosomal recessive
ABHD5	604780	Chanarin-Dorfman syndrome, 275630 (3), Autosomal recessive
ABL1	189980	Leukemia, Philadelphia chromosome-positive, resistant to imatinib, 608232 (3), Somatic mutation; Congenital heart defects and skeletal malformations syndrome, 617602 (3), Autosomal dominant
ABO	110300	[Blood group, ABO system], 616093 (3)
ACACA	200350	Acetyl-CoA carboxylase deficiency, 613933 (1), Autosomal recessive
ACAD8	604773	Isobutyryl-CoA dehydrogenase deficiency, 611283 (3), Autosomal recessive
ACAD9	611103	Mitochondrial complex I deficiency, nuclear type 20, 611126 (3), Autosomal recessive
ACADM	607008	Acyl-CoA dehydrogenase, medium chain, deficiency of, 201450 (3), Autosomal recessive
ACADS	606885	Acyl-CoA dehydrogenase, short-chain, deficiency of, 201470 (3), Autosomal recessive
ACADSB	600301	2-methylbutyrylglycinuria, 610006 (3), Autosomal recessive
ACADVL	609575	VLCAD deficiency, 201475 (3), Autosomal recessive
ACAN	155760	?Spondyloepiphyseal dysplasia, Kimberley type, 608361 (3), Autosomal dominant; Short stature and advanced bone age, with or without early-onset osteoarthritis and/or osteochondritis dissecans, 165800 (3), Autosomal dominant; Spondyloepimetaphyseal dysplasia, aggrecan type, 612813 (3), Autosomal recessive
ACAT1	607809	Alpha-methylacetoacetic aciduria, 203750 (3), Autosomal recessive
ACAT2	100678	?ACAT2 deficiency, 614055 (1), Isolated cases
ACBD5	616618	Retinal dystrophy with leukodystrophy, 618863 (3), Autosomal recessive
ACD	609377	?Dyskeratosis congenita, autosomal recessive 7, 616553 (3), Autosomal dominant, Autosomal recessive; ?Dyskeratosis congenita, autosomal dominant 6, 616553 (3), Autosomal dominant, Autosomal recessive
ACE	106180	{Stroke, hemorrhagic}, 614519 (3); Renal tubular dysgenesis, 267430 (3), Autosomal recessive; {Myocardial infarction, susceptibility to} (3); {Microvascular complications of diabetes 3}, 612624 (3); [Angiotensin I-converting enzyme, benign serum increase] (3); {SARS, progression of} (3)
ACER3	617036	?Leukodystrophy, progressive, early childhood-onset, 617762 (3), Autosomal recessive
ACHE	100740	[Blood group, Yt system], 112100 (3)
ACKR1	613665	[Blood group, Duffy system], 110700 (3), Autosomal dominant, Autosomal recessive; [White blood cell count QTL], 611862 (3), Autosomal recessive; {Malaria, vivax, protection against}, 611162 (3)
ACKR3	610376	?Oculomotor-abducens synkinesis, 619215 (3), Autosomal recessive

ACO2	100850	?Optic atrophy 9, 616289 (3), Autosomal recessive; Infantile cerebellar-retinal degeneration, 614559 (3), Autosomal recessive
ACOX1	609751	Mitchell syndrome, 618960 (3), Autosomal dominant; Peroxisomal acyl-CoA oxidase deficiency, 264470 (3), Autosomal recessive
ACOX2	601641	Bile acid synthesis defect, congenital, 6, 617308 (3), Autosomal recessive
ACP2	171650	?Lysosomal acid phosphatase deficiency, 200950 (1), Autosomal recessive
ACP4	606362	Amelogenesis imperfecta, type IJ, 617297 (3), Autosomal recessive
ACP5	171640	Spondyloenchondrodysplasia with immune dysregulation, 607944 (3), Autosomal recessive
ACR	102480	?Male infertility due to acrosin deficiency, 102480 (2)
ACSF3	614245	Combined malonic and methylmalonic aciduria, 614265 (3)
ACSL4	300157	Intellectual developmental disorder, X-linked 63, 300387 (3), X-linked dominant
ACSL6	604443	Myelodysplastic syndrome (3); Myelogenous leukemia, acute (3)
ACSM3	145505	{?Hypertension, essential} (1)
ACTA1	102610	?Myopathy, scapulohumeroperoneal, 616852 (3), Autosomal dominant; Nemaline myopathy 3, autosomal dominant or recessive, 161800 (3), Autosomal dominant, Autosomal recessive; Myopathy, actin, congenital, with excess of thin myofilaments, 161800 (3), Autosomal dominant, Autosomal recessive; Myopathy, actin, congenital, with cores, 161800 (3), Autosomal dominant, Autosomal recessive; Myopathy, congenital, with fiber-type disproportion 1, 255310 (3), Autosomal dominant, Autosomal recessive
ACTA2	102620	Multisystemic smooth muscle dysfunction syndrome, 613834 (3), Autosomal dominant; Aortic aneurysm, familial thoracic 6, 611788 (3), Autosomal dominant; Moyamoya disease 5, 614042 (3)
ACTB	102630	Baraitser-Winter syndrome 1, 243310 (3), Autosomal dominant; ?Dystonia, juvenile-onset, 607371 (3), Autosomal dominant
ACTC1	102540	Left ventricular noncompaction 4, 613424 (3), Autosomal dominant; Cardiomyopathy, hypertrophic, 11, 612098 (3), Autosomal dominant; Atrial septal defect 5, 612794 (3), Autosomal dominant; Cardiomyopathy, dilated, 1R, 613424 (3), Autosomal dominant
ACTG1	102560	Deafness, autosomal dominant 20/26, 604717 (3), Autosomal dominant; Baraitser-Winter syndrome 2, 614583 (3), Autosomal dominant
ACTG2	102545	Megacystis-microcolon-intestinal hypoperistalsis syndrome 5, 619431 (3), Autosomal dominant; Visceral myopathy 1, 155310 (3), Autosomal dominant
ACTL6B	612458	Developmental and epileptic encephalopathy 76, 618468 (3), Autosomal recessive; Intellectual developmental disorder with severe speech and ambulation defects, 618470 (3), Autosomal dominant
ACTL9	619251	Spermatogenic failure 53, 619258 (3), Autosomal recessive
ACTN1	102575	Bleeding disorder, platelet-type, 15, 615193 (3), Autosomal dominant

ACTN2	102573	Myopathy, distal, 6, adult onset, 618655 (3), Autosomal dominant; Cardiomyopathy, hypertrophic, 23, with or without LVNC, 612158 (3), Autosomal dominant; Cardiomyopathy, dilated, 1AA, with or without LVNC, 612158 (3), Autosomal dominant; Myopathy, congenital with structured cores and Z-line abnormalities, 618654 (3), Autosomal dominant
ACTN3	102574	[Sprinting performance], 617749 (3), Autosomal recessive; [Alpha-actinin-3 deficiency], 617749 (3), Autosomal recessive
ACTN4	604638	Glomerulosclerosis, focal segmental, 1, 603278 (3), Autosomal dominant
ACVR1	102576	Fibrodysplasia ossificans progressiva, 135100 (3), Autosomal dominant
ACVR1B	601300	Pancreatic cancer, somatic (3)
ACVR2B	602730	Heterotaxy, visceral, 4, autosomal, 613751 (3)
ACVRL1	601284	Telangiectasia, hereditary hemorrhagic, type 2, 600376 (3), Autosomal dominant
ACY1	104620	Aminoacylase 1 deficiency, 609924 (3), Autosomal recessive
ADA	608958	Adenosine deaminase deficiency, partial, 102700 (3), Somatic mosaicism, Autosomal recessive; Severe combined immunodeficiency due to ADA deficiency, 102700 (3), Somatic mosaicism, Autosomal recessive
ADA2	607575	Sneddon syndrome, 182410 (3), Autosomal recessive; Vasculitis, autoinflammation, immunodeficiency, and hematologic defects syndrome, 615688 (3), Autosomal recessive
ADAM10	602192	{Alzheimer disease 18, susceptibility to}, 615590 (3); Reticulate acropigmentation of Kitamura, 615537 (3), Autosomal dominant
ADAM17	603639	?Inflammatory skin and bowel disease, neonatal, 1, 614328 (3), Autosomal recessive
ADAM22	603709	Developmental and epileptic encephalopathy 61, 617933 (3), Autosomal recessive
ADAM9	602713	Cone-rod dystrophy 9, 612775 (3), Autosomal recessive
ADAMTS10	608990	Weill-Marchesani syndrome 1, recessive, 277600 (3), Autosomal recessive
ADAMTS13	604134	Thrombotic thrombocytopenic purpura, hereditary, 274150 (3), Autosomal recessive
ADAMTS17	607511	Weill-Marchesani 4 syndrome, recessive, 613195 (3), Autosomal recessive
ADAMTS18	607512	Microcornea, myopic chorioretinal atrophy, and telecanthus, 615458 (3), Autosomal recessive
ADAMTS2	604539	Ehlers-Danlos syndrome, dermatosparaxis type, 225410 (3), Autosomal recessive
ADAMTS3	605011	Hennekam lymphangiectasia-lymphedema syndrome 3, 618154 (3), Autosomal recessive
ADAMTSL2	612277	Geleophysic dysplasia 1, 231050 (3), Autosomal recessive
ADAMTSL4	610113	Ectopia lentis et pupillae, 225200 (3), Autosomal recessive; Ectopia lentis, isolated, autosomal recessive, 225100 (3), Autosomal recessive
ADAR	146920	Dyschromatosis symmetrica hereditaria, 127400 (3), Autosomal dominant; Aicardi-Goutieres syndrome 6, 615010 (3), Autosomal recessive
ADARB1	601218	Neurodevelopmental disorder with hypotonia, microcephaly, and seizures, 618862 (3), Autosomal recessive

ADAT3	615302	Neurodevelopmental disorder with brain abnormalities, poor growth, and dysmorphic facies, 615286 (3), Autosomal recessive
ADCY1	103072	?Deafness, autosomal recessive 44, 610154 (3), Autosomal recessive
ADCY10	605205	{Hypercalciuria, absorptive, susceptibility to}, 143870 (3), Autosomal dominant
ADCY3	600291	{Obesity, susceptibility to, BMIQ19}, 617885 (3), Autosomal recessive
ADCY5	600293	Dyskinesia with orofacial involvement, autosomal dominant, 606703 (3), Autosomal dominant; Neurodevelopmental disorder with hyperkinetic movements and dyskinesia, 619651 (3), Autosomal recessive; Dyskinesia with orofacial involvement, autosomal recessive, 619647 (3), Autosomal recessive
ADCY6	600294	Lethal congenital contracture syndrome 8, 616287 (3), Autosomal recessive
ADD1	102680	{Hypertension, essential, salt-sensitive}, 145500 (3), Multifactorial
ADD3	601568	Cerebral palsy, spastic quadriplegic, 3, 617008 (3), Autosomal recessive
ADGRE2	606100	Vibratory urticaria, 125630 (3), Autosomal dominant
ADGRG1	604110	Polymicrogyria, bilateral frontoparietal, 606854 (3), Autosomal recessive; Polymicrogyria, bilateral perisylvian, 615752 (3)
ADGRG2	300572	Congenital bilateral absence of vas deferens, X-linked, 300985 (3), X-linked
ADGRG6	612243	Lethal congenital contracture syndrome 9, 616503 (3), Autosomal recessive
ADGRV1	602851	Usher syndrome, type 2C, 605472 (3), Digenic dominant, Autosomal recessive; Usher syndrome, type 2C, GPR98/PDZD7 digenic, 605472 (3), Digenic dominant, Autosomal recessive; ?Febrile seizures, familial, 4, 604352 (3), Autosomal dominant
ADH1B	103720	{Aerodigestive tract cancer, squamous cell, alcohol-related, protection against}, 103780 (3), Multifactorial; {Alcohol dependence, protection against}, 103780 (3), Multifactorial
ADH1C	103730	{Alcohol dependence, protection against}, 103780 (3), Multifactorial; {Parkinson disease, susceptibility to}, 168600 (3), Multifactorial, Autosomal dominant
ADH5	103710	AMED syndrome, digenic, 619151 (3), Digenic recessive
ADIPOQ	605441	Adiponectin deficiency, 612556 (3)
ADK	102750	Hypermethioninemia due to adenosine kinase deficiency, 614300 (3), Autosomal recessive
ADNP	611386	Helsmoortel-van der Aa syndrome, 615873 (3), Autosomal dominant
ADPRS	610624	Neurodegeneration, childhood-onset, stress-induced, with variable ataxia and seizures, 618170 (3), Autosomal recessive
ADRA2C	104250	{Congestive heart failure and beta-blocker response, modifier of} (3)
ADRB1	109630	[Short sleep, familial natural, 2], 618591 (3), Autosomal dominant; [Resting heart rate], 607276 (3)

ADRB2	109690	Beta-2-adrenoreceptor agonist, reduced response to (3); {Obesity, susceptibility to}, 601665 (3), Multifactorial, Autosomal dominant, Autosomal recessive; {Asthma, nocturnal, susceptibility to}, 600807 (3), Autosomal dominant
ADRB3	109691	{Obesity, susceptibility to}, 601665 (3), Multifactorial, Autosomal dominant, Autosomal recessive
ADSL	608222	Adenylosuccinase deficiency, 103050 (3), Autosomal recessive
ADSS1	612498	Myopathy, distal, 5, 617030 (3), Autosomal recessive
AEBP1	602981	Ehlers-Danlos syndrome, classic-like, 2, 618000 (3), Autosomal recessive
AFF2	300806	Intellectual developmental disorder, X-linked 109, 309548 (3), X-linked recessive
AFF3	601464	KINSSHIP syndrome, 619297 (3), Autosomal dominant
AFF4	604417	CHOPS syndrome, 616368 (3), Autosomal dominant
AFG3L2	604581	Spastic ataxia 5, autosomal recessive, 614487 (3), Autosomal recessive; Optic atrophy 12, 618977 (3), Autosomal dominant; Spinocerebellar ataxia 28, 610246 (3), Autosomal dominant
AFP	104150	[Hereditary persistence of alpha-fetoprotein], 615970 (3), Autosomal dominant; Alpha-fetoprotein deficiency, 615969 (3), Autosomal recessive
AGA	613228	Aspartylglucosaminuria, 208400 (3), Autosomal recessive
AGBL1	615496	Corneal dystrophy, Fuchs endothelial, 8, 615523 (3), Autosomal dominant
AGBL5	615900	Retinitis pigmentosa 75, 617023 (3), Autosomal recessive
AGK	610345	Cataract 38, autosomal recessive, 614691 (3), Autosomal recessive; Sengers syndrome, 212350 (3), Autosomal recessive
AGL	610860	Glycogen storage disease IIIa, 232400 (3), Autosomal recessive; Glycogen storage disease IIIb, 232400 (3), Autosomal recessive
AGO2	606229	Lessel-Kreienkamp syndrome, 619149 (3), Autosomal dominant
AGPAT2	603100	Lipodystrophy, congenital generalized, type 1, 608594 (3), Autosomal recessive
AGPS	603051	Rhizomelic chondrodysplasia punctata, type 3, 600121 (3), Autosomal recessive
AGRN	103320	Myasthenic syndrome, congenital, 8, with pre- and postsynaptic defects, 615120 (3), Autosomal recessive
AGRP	602311	{Leanness, inherited}, 601665 (3), Multifactorial, Autosomal dominant, Autosomal recessive; {Obesity, late-onset}, 601665 (3), Multifactorial, Autosomal dominant, Autosomal recessive
AGT	106150	Renal tubular dysgenesis, 267430 (3), Autosomal recessive; {Preeclampsia, susceptibility to} (3); {Hypertension, essential, susceptibility to}, 145500 (3), Multifactorial
AGTPBP1	606830	Neurodegeneration, childhood-onset, with cerebellar atrophy, 618276 (3), Autosomal recessive
AGTR1	106165	{Hypertension, essential}, 145500 (3), Multifactorial; Renal tubular dysgenesis, 267430 (3), Autosomal recessive
AGXT	604285	Hyperoxaluria, primary, type 1, 259900 (3), Autosomal recessive

AGXT2	612471	[Beta-aminoisobutyric acid, urinary excretion of], 210100 (3), Autosomal recessive
AHCY	180960	Hypermethioninemia with deficiency of S-adenosylhomocysteine hydrolase, 613752 (3), Autosomal recessive
AHDC1	615790	Xia-Gibbs syndrome, 615829 (3), Autosomal dominant
AHI1	608894	Joubert syndrome 3, 608629 (3), Autosomal recessive
AHR	600253	?Retinitis pigmentosa 85, 618345 (3), Autosomal recessive
AHSG	138680	?Alopecia-intellectual disability syndrome 1, 203650 (3), Autosomal recessive
AICDA	605257	Immunodeficiency with hyper-IgM, type 2, 605258 (3), Autosomal recessive
AIFM1	300169	Combined oxidative phosphorylation deficiency 6, 300816 (3), X-linked recessive; Cowchock syndrome, 310490 (3), X-linked recessive; Spondyloepimetaphyseal dysplasia, X-linked, with hypomyelinating leukodystrophy, 300232 (3), X-linked recessive; Deafness, X-linked 5, 300614 (3), X-linked recessive
AIMP1	603605	Leukodystrophy, hypomyelinating, 3, 260600 (3), Autosomal recessive
AIMP2	600859	Leukodystrophy, hypomyelinating, 17, 618006 (3), Autosomal recessive
AIP	605555	Pituitary adenoma 1, multiple types, 102200 (3), Autosomal dominant, Somatic mutation; Pituitary adenoma predisposition, 102200 (3), Autosomal dominant, Somatic mutation
AIPL1	604392	Leber congenital amaurosis 4, 604393 (3), Autosomal dominant, Autosomal recessive; Retinitis pigmentosa, juvenile, 604393 (3), Autosomal dominant, Autosomal recessive; Cone-rod dystrophy, 604393 (3), Autosomal dominant, Autosomal recessive
AIRE	607358	Autoimmune polyendocrinopathy syndrome , type I, with or without reversible metaphyseal dysplasia, 240300 (3), Autosomal dominant, Autosomal recessive
AK1	103000	Hemolytic anemia due to adenylate kinase deficiency, 612631 (3), Autosomal recessive
AK2	103020	Reticular dysgenesis, 267500 (3), Autosomal recessive
AK7	615364	?Spermatogenic failure 27, 617965 (3), Autosomal recessive
AKAP9	604001	?Long QT syndrome 11, 611820 (3), Autosomal dominant
AKR1C2	600450	46XY sex reversal 8, 614279 (3), Autosomal recessive
AKR1C4	600451	{46XY sex reversal 8, modifier of}, 614279 (3), Autosomal recessive
AKR1D1	604741	Bile acid synthesis defect, congenital, 2, 235555 (3), Autosomal recessive
AKT1	164730	Breast cancer, somatic, 114480 (3); Cowden syndrome 6, 615109 (3); Colorectal cancer, somatic, 114500 (3); Proteus syndrome, somatic, 176920 (3); Ovarian cancer, somatic, 167000 (3)
AKT2	164731	Diabetes mellitus, type II, 125853 (3), Autosomal dominant; Hypoinsulinemic hypoglycemia with hemihypertrophy, 240900 (3), Autosomal dominant
AKT3	611223	Megalencephaly-polymicrogyria-polydactyly-hydrocephalus syndrome 2, 615937 (3), Autosomal dominant

ALAD	125270	Porphyria, acute hepatic, 612740 (3), Autosomal recessive; {Lead poisoning, susceptibility to}, 612740 (3), Autosomal recessive
ALAS2	301300	Anemia, sideroblastic, 1, 300751 (3), X-linked recessive; Protoporphyria, erythropoietic, X-linked, 300752 (3), X-linked
ALB	103600	?[Dysalbuminemic hypertriiodothyroninemia], 615999 (3), Autosomal dominant, Autosomal recessive; Analbuminemia, 616000 (3), Autosomal recessive; [Dysalbuminemic hyperthyroxinemia], 615999 (3), Autosomal dominant, Autosomal recessive
ALDH18A1	138250	Spastic paraplegia 9A, autosomal dominant, 601162 (3), Autosomal dominant; Cutis laxa, autosomal recessive, type IIIA, 219150 (3), Autosomal recessive; Spastic paraplegia 9B, autosomal recessive, 616586 (3), Autosomal recessive; Cutis laxa, autosomal dominant 3, 616603 (3), Autosomal dominant
ALDH1A3	600463	Microphthalmia, isolated 8, 615113 (3), Autosomal recessive
ALDH2	100650	{Esophageal cancer, alcohol-related, susceptibility to} (3); {Sublingual nitroglycerin, susceptibility to poor response to} (3); Alcohol sensitivity, acute, 610251 (3), Autosomal dominant; {Hangover, susceptibility to}, 610251 (3), Autosomal dominant
ALDH3A2	609523	Sjogren-Larsson syndrome, 270200 (3), Autosomal recessive
ALDH4A1	606811	Hyperprolinemia, type II, 239510 (3), Autosomal recessive
ALDH5A1	610045	Succinic semialdehyde dehydrogenase deficiency, 271980 (3), Autosomal recessive
ALDH6A1	603178	Methylmalonate semialdehyde dehydrogenase deficiency, 614105 (3), Autosomal recessive
ALDH7A1	107323	Epilepsy, pyridoxine-dependent, 266100 (3), Autosomal recessive
ALDOA	103850	Glycogen storage disease XII, 611881 (3), Autosomal recessive
ALDOB	612724	Fructose intolerance, hereditary, 229600 (3), Autosomal recessive
ALG1	605907	Congenital disorder of glycosylation, type Ik, 608540 (3), Autosomal recessive
ALG10B	603313	{Long QT syndrome, acquired, reduced susceptibility to}, 613688 (3), Autosomal dominant
ALG11	613666	Congenital disorder of glycosylation, type Ip, 613661 (3), Autosomal recessive
ALG12	607144	Congenital disorder of glycosylation, type Ig, 607143 (3), Autosomal recessive
ALG13	300776	Developmental and epileptic encephalopathy 36, 300884 (3), X-linked
ALG14	612866	Intellectual developmental disorder with epilepsy, behavioral abnormalities, and coarse facies, 619031 (3), Autosomal recessive; Myopathy, epilepsy, and progressive cerebral atrophy, 619036 (3), Autosomal recessive; ?Myasthenic syndrome, congenital, 15, without tubular aggregates, 616227 (3), Autosomal recessive
ALG2	607905	Congenital disorder of glycosylation, type Ii, 607906 (3), Autosomal recessive; Myasthenic syndrome, congenital, 14, with tubular aggregates, 616228 (3), Autosomal recessive

ALG3	608750	Congenital disorder of glycosylation, type Id, 601110 (3), Autosomal recessive
ALG6	604566	Congenital disorder of glycosylation, type Ic, 603147 (3), Autosomal recessive
ALG8	608103	Congenital disorder of glycosylation, type Ih, 608104 (3), Autosomal recessive; Polycystic liver disease 3 with or without kidney cysts, 617874 (3), Autosomal dominant
ALG9	606941	Gillessen-Kaesbach-Nishimura syndrome, 263210 (3), Autosomal recessive; Congenital disorder of glycosylation, type Il, 608776 (3), Autosomal recessive
ALK	105590	{Neuroblastoma, susceptibility to, 3}, 613014 (3)
ALKBH8	613306	Intellectual developmental disorder, autosomal recessive 71, 618504 (3), Autosomal recessive
ALMS1	606844	Alstrom syndrome, 203800 (3), Autosomal recessive
ALOX12B	603741	Ichthyosis, congenital, autosomal recessive 2, 242100 (3), Autosomal recessive
ALOX5	152390	{Atherosclerosis, susceptibility to} (3); {Asthma, diminished response to antileukotriene treatment in}, 600807 (3), Autosomal dominant
ALOX5AP	603700	{Stroke, susceptibility to}, 601367 (3), Multifactorial
ALOXE3	607206	Ichthyosis, congenital, autosomal recessive 3, 606545 (3), Autosomal recessive
ALPK1	607347	ROSAH syndrome, 614979 (3), Autosomal dominant
ALPK3	617608	Cardiomyopathy, familial hypertrophic 27, 618052 (3), Autosomal recessive
ALPL	171760	Odontohypophosphatasia, 146300 (3), Autosomal dominant, Autosomal recessive; Hypophosphatasia, infantile, 241500 (3), Autosomal recessive; Hypophosphatasia, childhood, 241510 (3), Autosomal recessive; Hypophosphatasia, adult, 146300 (3), Autosomal dominant, Autosomal recessive
ALS2	606352	Primary lateral sclerosis, juvenile, 606353 (3), Autosomal recessive; Spastic paralysis, infantile onset ascending, 607225 (3), Autosomal recessive; Amyotrophic lateral sclerosis 2, juvenile, 205100 (3), Autosomal recessive
ALX1	601527	Frontonasal dysplasia 3, 613456 (3), Autosomal recessive
ALX3	606014	Frontonasal dysplasia 1, 136760 (3), Autosomal recessive
ALX4	605420	Parietal foramina 2, 609597 (3), Autosomal dominant; {Craniosynostosis 5, susceptibility to}, 615529 (3), Autosomal dominant; Frontonasal dysplasia 2, 613451 (3), Autosomal recessive
AMACR	604489	Alpha-methylacyl-CoA racemase deficiency, 614307 (3), Autosomal recessive; Bile acid synthesis defect, congenital, 4, 214950 (3), Autosomal recessive
AMBN	601259	Amelogenesis imperfecta, type IF, 616270 (3), Autosomal recessive
AMELX	300391	Amelogenesis imperfecta, type 1E, 301200 (3), X-linked dominant
AMER1	300647	Osteopathia striata with cranial sclerosis, 300373 (3), X-linked dominant
AMH	600957	Persistent Mullerian duct syndrome, type I, 261550 (3), Autosomal recessive

AMHR2	600956	Persistent Mullerian duct syndrome, type II, 261550 (3), Autosomal recessive
AMMECR1	300195	Midface hypoplasia, hearing impairment, elliptocytosis, and nephrocalcinosis, 300990 (3), X-linked recessive
AMN	605799	Imerslund-Grasbeck syndrome 2, 618882 (3), Autosomal recessive
AMPD1	102770	Myopathy due to myoadenylate deaminase deficiency, 615511 (3), Autosomal recessive
AMPD2	102771	?Spastic paraplegia 63, 615686 (3), Autosomal recessive; Pontocerebellar hypoplasia, type 9, 615809 (3), Autosomal recessive
AMPD3	102772	[AMP deaminase deficiency, erythrocytic], 612874 (3), Autosomal recessive
AMT	238310	Glycine encephalopathy, 605899 (3), Autosomal recessive
AMTN	610912	?Amelogenesis imperfecta, type IIIB, 617607 (3), Autosomal dominant
ANAPC1	608473	Rothmund-Thomson syndrome, type 1, 618625 (3), Autosomal recessive
ANAPC7	606949	Ferguson-Bonni neurodevelopmental syndrome, 619699 (3), Autosomal recessive
ANG	105850	Amyotrophic lateral sclerosis 9, 611895 (3)
ANGPT1	601667	?Angioedema, hereditary, 5, 619361 (3), Autosomal dominant
ANGPT2	601922	Lymphatic malformation 10, 619369 (3), Autosomal dominant
ANGPTL3	604774	Hypobetalipoproteinemia, familial, 2, 605019 (3), Autosomal recessive
ANGPTL4	605910	Plasma triglyceride level QTL, low, 615881 (3), Autosomal dominant
ANK1	612641	Spherocytosis, type 1, 182900 (3), Autosomal dominant, Autosomal recessive
ANK2	106410	Long QT syndrome 4, 600919 (3), Autosomal dominant; Cardiac arrhythmia, ankyrin-B-related, 600919 (3), Autosomal dominant
ANK3	600465	Intellectual developmental disorder, autosomal recessive 37, 615493 (3), Autosomal recessive
ANKH	605145	Chondrocalcinosis 2, 118600 (3), Autosomal dominant; Craniometaphyseal dysplasia, 123000 (3), Autosomal dominant
ANKLE2	616062	Microcephaly 16, primary, autosomal recessive, 616681 (3), Autosomal recessive
ANKRD11	611192	KBG syndrome, 148050 (3), Autosomal dominant
ANKRD17	615929	Chopra-Amiel-Gordon syndrome, 619504 (3), Autosomal dominant
ANKRD26	610855	Thrombocytopenia 2, 188000 (3), Autosomal dominant
ANKS6	615370	Nephronophthisis 16, 615382 (3), Autosomal recessive
ANLN	616027	Focal segmental glomerulosclerosis 8, 616032 (3), Autosomal dominant
ANO10	613726	Spinocerebellar ataxia, autosomal recessive 10, 613728 (3), Autosomal recessive
ANO3	610110	Dystonia 24, 615034 (3), Autosomal dominant
ANO5	608662	Muscular dystrophy, limb-girdle, autosomal recessive 12, 611307 (3), Autosomal recessive; Miyoshi muscular dystrophy 3, 613319 (3), Autosomal recessive; Gnathodiaphyseal dysplasia, 166260 (3), Autosomal dominant
ANO6	608663	Scott syndrome, 262890 (3), Autosomal recessive

ANOS1	300836	Hypogonadotropic hypogonadism 1 with or without anosmia (Kallmann syndrome 1), 308700 (3), X-linked recessive
ANTXR1	606410	GAPO syndrome, 230740 (3), Autosomal recessive; {?Hemangioma, capillary infantile, susceptibility to}, 602089 (3), Autosomal dominant
ANTXR2	608041	Hyaline fibromatosis syndrome, 228600 (3), Autosomal recessive
ANXA11	602572	Amyotrophic lateral sclerosis 23, 617839 (3), Autosomal dominant; Inclusion body myopathy and brain white matter abnormalities, 619733 (3), Autosomal dominant
ANXA5	131230	{Pregnancy loss, recurrent, susceptibility to, 3}, 614391 (3), Autosomal dominant
AOPEP	619600	Dystonia 31, 619565 (3), Autosomal recessive
AP1B1	600157	Keratitis-ichthyosis-deafness syndrome, autosomal recessive, 242150 (3), Autosomal recessive
AP1G1	603533	Usmani-Riazuddin syndrome, autosomal recessive, 619548 (3); Usmani-Riazuddin syndrome, autosomal dominant, 619467 (3), Autosomal dominant
AP1S1	603531	MEDNIK syndrome, 609313 (3), Autosomal recessive
AP1S2	300629	Pettigrew syndrome, 304340 (3), X-linked recessive
AP1S3	615781	{Psoriasis 15, pustular, susceptibility to}, 616106 (3), Autosomal dominant
AP2M1	601024	Intellectual developmental disorder 60 with seizures, 618587 (3), Autosomal dominant
AP2S1	602242	Hypocalciuric hypercalcemia, type III, 600740 (3), Autosomal dominant
AP3B1	603401	Hermansky-Pudlak syndrome 2, 608233 (3), Autosomal recessive
AP3B2	602166	Developmental and epileptic encephalopathy 48, 617276 (3), Autosomal recessive
AP3D1	607246	?Hermansky-Pudlak syndrome 10, 617050 (3), Autosomal recessive
AP4B1	607245	Spastic paraplegia 47, autosomal recessive, 614066 (3), Autosomal recessive
AP4E1	607244	Stuttering, familial persistent, 1, 184450 (3), Autosomal dominant; Spastic paraplegia 51, autosomal recessive, 613744 (3), Autosomal recessive
AP4M1	602296	Spastic paraplegia 50, autosomal recessive, 612936 (3), Autosomal recessive
AP4S1	607243	Spastic paraplegia 52, autosomal recessive, 614067 (3), Autosomal recessive
AP5Z1	613653	Spastic paraplegia 48, autosomal recessive, 613647 (3), Autosomal recessive
APC	611731	Colorectal cancer, somatic, 114500 (3); Brain tumor-polyposis syndrome 2, 175100 (3), Autosomal dominant; Desmoid disease, hereditary, 135290 (3), Autosomal dominant; Adenoma, periampullary, somatic, 175100 (3); Hepatoblastoma, somatic, 114550 (3); Gastric cancer, somatic, 613659 (3); Gastric adenocarcinoma and proximal polyposis of the stomach, 619182 (3), Autosomal dominant; Gardner syndrome, 175100 (3), Autosomal dominant; Adenomatous polyposis coli, 175100 (3), Autosomal dominant

APC2	612034	Cortical dysplasia, complex, with other brain malformations 10, 618677 (3), Autosomal recessive; Intellectual developmental disorder, autosomal recessive 74, 617169 (3), Autosomal recessive
APCDD1	607479	Hypotrichosis 1, 605389 (3), Autosomal dominant
APCS	104770	{?Amyloidosis, secondary, susceptibility to} (1)
APOA1	107680	Hypoalphalipoproteinemia, primary, 2, 618463 (3), Autosomal recessive; Amyloidosis, 3 or more types, 105200 (3), Autosomal dominant; Hypoalphalipoproteinemia, primary, 2, intermediate, 619836 (3), Autosomal dominant
APOA2	107670	Apolipoprotein A-II deficiency (3); {Hypercholesterolemia, familial, modifier of}, 143890 (3), Autosomal dominant, Autosomal recessive
APOA5	606368	Hyperchylomicronemia, late-onset, 144650 (3), Autosomal dominant; {Hypertriglyceridemia, susceptibility to}, 145750 (3), Autosomal dominant
APOB	107730	Hypercholesterolemia, familial, 2, 144010 (3), Autosomal dominant; Hypobetalipoproteinemia, 615558 (3), Autosomal recessive
APOC2	608083	Hyperlipoproteinemia, type Ib, 207750 (3), Autosomal recessive
APOC3	107720	Apolipoprotein C-III deficiency, 614028 (3)
APOE	107741	Alzheimer disease 2, 104310 (3), Autosomal dominant; Sea-blue histiocyte disease, 269600 (3), Autosomal recessive; {?Alzheimer disease, protection against, due to APOE3-Christchurch}, 607822 (3), Autosomal dominant; {Coronary artery disease, severe, susceptibility to}, 617347 (3); Lipoprotein glomerulopathy, 611771 (3); {?Macular degeneration, age-related}, 603075 (3), Autosomal dominant; Hyperlipoproteinemia, type III, 617347 (3)
APOL1	603743	{End-stage renal disease, nondiabetic, susceptibility to}, 612551 (3); {Glomerulosclerosis, focal segmental, 4, susceptibility to}, 612551 (3)
APOL2	607252	{Schizophrenia}, 181500 (1), Autosomal dominant
APOL4	607254	{Schizophrenia}, 181500 (1), Autosomal dominant
APP	104760	Cerebral amyloid angiopathy, Dutch, Italian, Iowa, Flemish, Arctic variants, 605714 (3), Autosomal dominant; Alzheimer disease 1, familial, 104300 (3), Autosomal dominant
APPL1	604299	{Maturity-onset diabetes of the young, type 14}, 616511 (3), Autosomal dominant
APRT	102600	Adenine phosphoribosyltransferase deficiency, 614723 (3), Autosomal recessive
APTX	606350	Ataxia, early-onset, with oculomotor apraxia and hypoalbuminemia, 208920 (3), Autosomal recessive
AQP1	107776	[Aquaporin-1 deficiency], 110450 (3); [Blood group, Colton], 110450 (3)
AQP2	107777	Diabetes insipidus, nephrogenic, 2, 125800 (3), Autosomal dominant, Autosomal recessive
AQP3	600170	[Blood group GIL], 607457 (3)
AQP5	600442	Palmoplantar keratoderma, Bothnian type, 600231 (3), Autosomal dominant
AQP7	602974	[Glycerol quantitative trait locus], 614411 (3), Autosomal recessive

AR	313700	Androgen insensitivity, partial, with or without breast cancer, 312300 (3), X-linked recessive; {Prostate cancer, susceptibility to}, 176807 (3), Autosomal dominant, Somatic mutation; Androgen insensitivity, 300068 (3), X-linked recessive; Spinal and bulbar muscular atrophy of Kennedy, 313200 (3), X-linked recessive; Hypospadias 1, X-linked, 300633 (3), X-linked recessive
ARCN1	600820	Short stature, rhizomelic, with microcephaly, micrognathia, and developmental delay, 617164 (3), Autosomal dominant
ARF1	103180	Periventricular nodular heterotopia 8, 618185 (3), Autosomal dominant
ARFGEF1	604141	Developmental delay, impaired speech, and behavioral abnormalities, with or without seizures, 619964 (3), Autosomal dominant
ARFGEF2	605371	Periventricular heterotopia with microcephaly, 608097 (3), Autosomal recessive
ARG1	608313	Argininemia, 207800 (3), Autosomal recessive
ARHGAP26	605370	Leukemia, juvenile myelomonocytic, somatic, 607785 (3)
ARHGAP31	610911	Adams-Oliver syndrome 1, 100300 (3), Autosomal dominant
ARHGDIA	601925	Nephrotic syndrome, type 8, 615244 (3), Autosomal recessive
ARHGEF1	601855	?Immunodeficiency 62, 618459 (3), Autosomal recessive
ARHGEF10	608136	?Slowed nerve conduction velocity, AD, 608236 (3), Autosomal dominant
ARHGEF18	616432	Retinitis pigmentosa 78, 617433 (3), Autosomal recessive
ARHGEF2	607560	?Neurodevelopmental disorder with midbrain and hindbrain malformations, 617523 (3), Autosomal recessive
ARHGEF9	300429	Developmental and epileptic encephalopathy 8, 300607 (3), X-linked
ARID1A	603024	Coffin-Siris syndrome 2, 614607 (3), Autosomal dominant
ARID1B	614556	Coffin-Siris syndrome 1, 135900 (3), Autosomal dominant
ARID2	609539	Coffin-Siris syndrome 6, 617808 (3), Autosomal dominant
ARL13B	608922	Joubert syndrome 8, 612291 (3), Autosomal recessive
ARL2	601175	?Microcornea, rod-cone dystrophy, cataract, and posterior staphyloma 1, 619082 (3), Autosomal dominant
ARL2BP	615407	Retinitis pigmentosa with or without situs inversus, 615434 (3), Autosomal recessive
ARL3	604695	Retinitis pigmentosa 83, 618173 (3), Autosomal dominant; Joubert syndrome 35, 618161 (3), Autosomal recessive
ARL6	608845	Retinitis pigmentosa 55, 613575 (3), Autosomal recessive; {Bardet-Biedl syndrome 1, modifier of}, 209900 (3), Digenic recessive, Autosomal recessive; Bardet-Biedl syndrome 3, 600151 (3), Autosomal recessive
ARL6IP1	607669	?Spastic paraplegia 61, autosomal recessive, 615685 (3), Autosomal recessive
ARMC2	618424	Spermatogenic failure 38, 618433 (3), Autosomal recessive
ARMC5	615549	ACTH-independent macronodular adrenal hyperplasia 2, 615954 (3), Autosomal dominant, Somatic mutation
ARMC9	617612	Joubert syndrome 30, 617622 (3), Autosomal recessive
ARMS2	611313	{Macular degeneration, age-related, 8}, 613778 (3)
ARNT2	606036	?Webb-Dattani syndrome, 615926 (3), Autosomal recessive

ARPC1B	604223	Immunodeficiency 71 with inflammatory disease and congenital thrombocytopenia, 617718 (3), Autosomal recessive
ARR3	301770	Myopia 26, X-linked, female-limited, 301010 (3), X-linked
ARSA	607574	Metachromatic leukodystrophy, 250100 (3), Autosomal recessive
ARSB	611542	Mucopolysaccharidosis type VI (Maroteaux-Lamy), 253200 (3), Autosomal recessive
ARSG	610008	Usher syndrome, type IV, 618144 (3), Autosomal recessive
ARSK	610011	Mucopolysaccharidosis, type X, 619698 (3), Autosomal recessive
ARSL	300180	Chondrodysplasia punctata, X-linked recessive, 302950 (3), X-linked recessive
ART4	110600	[Blood group, Dombrock], 616060 (3)
ARV1	611647	Developmental and epileptic encephalopathy 38, 617020 (3), Autosomal recessive
ARX	300382	Proud syndrome, 300004 (3), X-linked; Hydranencephaly with abnormal genitalia, 300215 (3), X-linked; Partington syndrome, 309510 (3), X-linked recessive; Developmental and epileptic encephalopathy 1, 308350 (3), X-linked recessive; Lissencephaly, X-linked 2, 300215 (3), X-linked; Intellectual developmental disorder, X-linked 29, 300419 (3), X-linked recessive
ASAH1	613468	Spinal muscular atrophy with progressive myoclonic epilepsy, 159950 (3), Autosomal recessive; Farber lipogranulomatosis, 228000 (3), Autosomal recessive
ASB10	615054	Glaucoma 1, open angle, F, 603383 (3), Autosomal dominant
ASCC1	614215	Spinal muscular atrophy with congenital bone fractures 2, 616867 (3), Autosomal recessive; Barrett esophagus/esophageal adenocarcinoma, 614266 (3)
ASCL1	100790	Haddad syndrome, 209880 (3), Autosomal dominant; Central hypoventilation syndrome, congenital, 209880 (3), Autosomal dominant
ASH1L	607999	Intellectual developmental disorder, autosomal dominant 52, 617796 (3), Autosomal dominant
ASIP	600201	[Skin/hair/eye pigmentation 9, brown/nonbrown eyes], 611742 (3); [Skin/hair/eye pigmentation 9, dark/light hair], 611742 (3)
ASL	608310	Argininosuccinic aciduria, 207900 (3), Autosomal recessive
ASNS	108370	Asparagine synthetase deficiency, 615574 (3), Autosomal recessive
ASPA	608034	Canavan disease, 271900 (3), Autosomal recessive
ASPH	600582	Traboulsi syndrome, 601552 (3), Autosomal recessive
ASPM	605481	Microcephaly 5, primary, autosomal recessive, 608716 (3), Autosomal recessive
ASPN	608135	{Lumbar disc degeneration}, 603932 (3); {Osteoarthritis susceptibility 3}, 607850 (3), Autosomal dominant
ASPRV1	611765	Ichthyosis, lamellar, autosomal dominant, 146750 (3), Autosomal dominant
ASPSCR1	606236	Alveolar soft-part sarcoma, 606243 (3)
ASS1	603470	Citrullinemia, 215700 (3), Autosomal recessive
ASTL	608860	?Oocyte maturation defect 11, 619643 (3), Autosomal recessive

ASXL1	612990	Myelodysplastic syndrome, somatic, 614286 (3); Bohring-Opitz syndrome, 605039 (3), Autosomal dominant
ASXL2	612991	Shashi-Pena syndrome, 617190 (3), Autosomal dominant
ASXL3	615115	Bainbridge-Ropers syndrome, 615485 (3), Autosomal dominant
ATAD1	614452	Hyperekplexia 4, 618011 (3), Autosomal recessive
ATAD3A	612316	Harel-Yoon syndrome, 617183 (3), Autosomal dominant, Autosomal recessive; Pontocerebellar hypoplasia, hypotonia, and respiratory insufficiency syndrome, neonatal lethal, 618810 (3), Autosomal recessive
ATCAY	608179	Ataxia, cerebellar, Cayman type, 601238 (3), Autosomal recessive
ATF6	605537	Achromatopsia 7, 616517 (3), Autosomal recessive
ATG16L1	610767	{Inflammatory bowel disease (Crohn disease) 10}, 611081 (3)
ATG5	604261	?Spinocerebellar ataxia, autosomal recessive 25, 617584 (3), Autosomal recessive
ATG7	608760	Spinocerebellar ataxia, autosomal recessive 31, 619422 (3), Autosomal recessive
ATIC	601731	AICA-ribosiduria due to ATIC deficiency, 608688 (3), Autosomal recessive
ATL1	606439	Spastic paraplegia 3A, autosomal dominant, 182600 (3), Autosomal dominant; Neuropathy, hereditary sensory, type ID, 613708 (3), Autosomal dominant
ATL3	609369	Neuropathy, hereditary sensory, type IF, 615632 (3), Autosomal dominant
ATM	607585	Lymphoma, B-cell non-Hodgkin, somatic (3); Ataxia-telangiectasia, 208900 (3), Autosomal recessive; {Breast cancer, susceptibility to}, 114480 (3), Autosomal dominant, Somatic mutation; T-cell prolymphocytic leukemia, somatic (3); Lymphoma, mantle cell, somatic (3)
ATN1	607462	Dentatorubral-pallidoluysian atrophy, 125370 (3), Autosomal dominant; Congenital hypotonia, epilepsy, developmental delay, and digital anomalies, 618494 (3), Autosomal dominant
ATOH7	609875	Persistent hyperplastic primary vitreous, autosomal recessive, 221900 (3), Autosomal recessive
ATP11A	605868	?Leukodystrophy, hypomyelinating, 24, 619851 (3), Autosomal dominant; Deafness, autosomal dominant 84, 619810 (3), Autosomal dominant
ATP11C	300516	?Hemolytic anemia, congenital, X-linked, 301015 (3), X-linked recessive
ATP13A2	610513	Spastic paraplegia 78, autosomal recessive, 617225 (3), Autosomal recessive; Kufor-Rakeb syndrome, 606693 (3), Autosomal recessive
ATP13A3	610232	Pulmonary hypertension, primary, 5, 265400 (3), Autosomal recessive
ATP1A1	182310	Hypomagnesemia, seizures, and mental retardation 2, 618314 (3), Autosomal dominant; Charcot-Marie-Tooth disease, axonal, type 2DD, 618036 (3), Autosomal dominant
ATP1A2	182340	Developmental and epileptic encephalopathy 98, 619605 (3), Autosomal dominant; Fetal akinesia, respiratory insufficiency, microcephaly, polymicrogyria, and dysmorphic facies, 619602 (3), Autosomal recessive; Alternating hemiplegia of childhood 1, 104290 (3), Autosomal dominant; Migraine, familial basilar, 602481 (3), Autosomal dominant; Migraine, familial hemiplegic, 2, 602481 (3), Autosomal dominant

ATP1A3	182350	Alternating hemiplegia of childhood 2, 614820 (3), Autosomal dominant; Dystonia-12, 128235 (3), Autosomal dominant; CAPOS syndrome, 601338 (3), Autosomal dominant; Developmental and epileptic encephalopathy 99, 619606 (3), Autosomal dominant
ATP1B1	182330	[Blood pressure regulation QTL], 145500 (2), Multifactorial
ATP2A1	108730	Brody myopathy, 601003 (3), Autosomal recessive
ATP2A2	108740	Acrokeratosis verruciformis, 101900 (3), Autosomal dominant; Darier disease, 124200 (3), Autosomal dominant
ATP2B1	108731	Intellectual developmental disorder, autosomal dominant 66, 619910 (3), Autosomal dominant
ATP2B2	108733	Deafness, autosomal dominant 82, 619804 (3), Autosomal dominant; {Deafness, autosomal recessive 12, modifier of}, 601386 (3), Autosomal recessive
ATP2B3	300014	?Spinocerebellar ataxia, X-linked 1, 302500 (3), X-linked recessive
ATP2C1	604384	Hailey-Hailey disease, 169600 (3), Autosomal dominant
ATP5F1A	164360	?Mitochondrial complex V (ATP synthase) deficiency, nuclear type 4, 615228 (3), Autosomal recessive; ?Combined oxidative phosphorylation deficiency 22, 616045 (3), Autosomal recessive
ATP5F1D	603150	Mitochondrial complex V (ATP synthase) deficiency, 618120 (3), Autosomal recessive
ATP5F1E	606153	?Mitochondrial complex V (ATP synthase) deficiency, nuclear type 3, 614053 (3), Autosomal recessive
ATP5MC3	602736	Dystonia, early-onset, and/or spastic paraplegia, 619681 (3), Autosomal dominant
ATP5MK	615204	Mitochondrial complex V (ATP synthase) deficiency, nuclear type 6, 618683 (3), Autosomal recessive
ATP6AP1	300197	Immunodeficiency 47, 300972 (3), X-linked recessive
ATP6AP2	300556	Intellectual developmental disorder, X-linked syndromic, Hedera type, 300423 (3), X-linked recessive; ?Parkinsonism with spasticity, X-linked, 300911 (3), X-linked recessive; Congenital disorder of glycosylation, type IIr, 301045 (3), X-linked recessive
ATP6V0A1	192130	Neurodevelopmental disorder with epilepsy and brain atrophy, 619971 (3), Autosomal recessive; Developmental and epileptic encephalopathy 104, 619970 (3), Autosomal dominant
ATP6V0A2	611716	Wrinkly skin syndrome, 278250 (3), Autosomal recessive; Cutis laxa, autosomal recessive, type IIA, 219200 (3), Autosomal recessive
ATP6V0A4	605239	Distal renal tubular acidosis 3, with or without sensorineural hearing loss, 602722 (3), Autosomal recessive
ATP6V1A	607027	Cutis laxa, autosomal recessive, type IID, 617403 (3), Autosomal recessive; Developmental and epileptic encephalopathy 93, 618012 (3), Autosomal dominant
ATP6V1B1	192132	Distal renal tubular acidosis 2 with progressive sensorineural hearing loss, 267300 (3), Autosomal recessive

ATP6V1B2	606939	Zimmermann-Laband syndrome 2, 616455 (3), Autosomal dominant; Deafness, congenital, with onychodystrophy, autosomal dominant, 124480 (3), Autosomal dominant
ATP6V1E1	108746	Cutis laxa, autosomal recessive, type IIC, 617402 (3), Autosomal recessive
ATP7A	300011	Occipital horn syndrome, 304150 (3), X-linked recessive; Spinal muscular atrophy, distal, X-linked 3, 300489 (3), X-linked recessive; Menkes disease, 309400 (3), X-linked recessive
ATP7B	606882	Wilson disease, 277900 (3), Autosomal recessive
ATP8A2	605870	?Cerebellar ataxia, mental retardation, and dysequilibrium syndrome 4, 615268 (3), Autosomal recessive
ATP8B1	602397	Cholestasis, progressive familial intrahepatic 1, 211600 (3), Autosomal recessive; Cholestasis, intrahepatic, of pregnancy, 1, 147480 (3), Autosomal dominant; Cholestasis, benign recurrent intrahepatic, 243300 (3), Autosomal recessive
ATPAF2	608918	?Mitochondrial complex V (ATP synthase) deficiency, nuclear type 1, 604273 (3), Autosomal recessive
ATR	601215	Seckel syndrome 1, 210600 (3), Autosomal recessive; ?Cutaneous telangiectasia and cancer syndrome, familial, 614564 (3), Autosomal dominant
ATRX	300032	Alpha-thalassemia/mental retardation syndrome, 301040 (3), X-linked dominant; Alpha-thalassemia myelodysplasia syndrome, somatic, 300448 (3); Intellectual disability-hypotonic facies syndrome, X-linked, 309580 (3), X-linked recessive
ATXN1	601556	Spinocerebellar ataxia 1, 164400 (3), Autosomal dominant
ATXN10	611150	Spinocerebellar ataxia 10, 603516 (3), Autosomal dominant
ATXN2	601517	{Amyotrophic lateral sclerosis, susceptibility to, 13}, 183090 (3), Autosomal dominant; Spinocerebellar ataxia 2, 183090 (3), Autosomal dominant; {Parkinson disease, late-onset, susceptibility to}, 168600 (3), Multifactorial, Autosomal dominant
ATXN3	607047	Machado-Joseph disease, 109150 (3), Autosomal dominant
ATXN7	607640	Spinocerebellar ataxia 7, 164500 (3), Autosomal dominant
AUH	600529	3-methylglutaconic aciduria, type I, 250950 (3), Autosomal recessive
AURKA	603072	{Colon cancer, susceptibility to}, 114500 (3), Autosomal dominant, Somatic mutation
AURKC	603495	Spermatogenic failure 5, 243060 (3), Autosomal recessive
AUTS2	607270	Intellectual developmental disorder, autosomal dominant 26, 615834 (3), Autosomal dominant
AVIL	613397	Nephrotic syndrome, type 21, 618594 (3), Autosomal recessive
AVP	192340	Diabetes insipidus, neurohypophyseal, 125700 (3), Autosomal dominant
AVPR2	300538	Diabetes insipidus, nephrogenic, 1, 304800 (3), X-linked recessive; Nephrogenic syndrome of inappropriate antidiuresis, 300539 (3), X-linked recessive
AXIN1	603816	Hepatocellular carcinoma, somatic, 114550 (3); ?Caudal duplication anomaly, 607864 (3)

AXIN2	604025	Colorectal cancer, somatic, 114500 (3); Oligodontia-colorectal cancer syndrome, 608615 (3), Autosomal dominant
B2M	109700	?Amyloidosis, familial visceral, 105200 (3), Autosomal dominant; Immunodeficiency 43, 241600 (3), Autosomal recessive
B3GALNT1	603094	[Blood group, P1PK system, P(k) phenotype], 111400 (3); [Blood group, globoside system], 615021 (3)
B3GALNT2	610194	Muscular dystrophy-dystroglycanopathy (congenital with brain and eye anomalies, type A, 11, 615181 (3), Autosomal recessive
B3GALT6	615291	Ehlers-Danlos syndrome, spondylodysplastic type, 2, 615349 (3), Autosomal recessive; Spondyloepimetaphyseal dysplasia with joint laxity, type 1, with or without fractures, 271640 (3), Autosomal recessive; Al-Gazali syndrome, 609465 (3), Autosomal recessive
B3GAT3	606374	Multiple joint dislocations, short stature, craniofacial dysmorphism, with or without congenital heart defects, 245600 (3), Autosomal recessive
B3GLCT	610308	Peters-plus syndrome, 261540 (3), Autosomal recessive
B4GALNT1	601873	Spastic paraplegia 26, autosomal recessive, 609195 (3), Autosomal recessive
B4GALNT2	111730	[Blood group, Sid system], 615018 (3); Sd(a) polyagglutination syndrome, 615018 (3)
B4GALT1	137060	Congenital disorder of glycosylation, type IId, 607091 (3), Autosomal recessive
B4GALT7	604327	Ehlers-Danlos syndrome, spondylodysplastic type, 1, 130070 (3), Autosomal recessive
B4GAT1	605517	Muscular dystrophy-dystroglycanopathy (congenital with brain and eye anomalies), type A, 13, 615287 (3), Autosomal recessive
B9D1	614144	?Meckel syndrome 9, 614209 (3), Autosomal recessive; Joubert syndrome 27, 617120 (3), Autosomal recessive
B9D2	611951	?Meckel syndrome 10, 614175 (3), Autosomal recessive; Joubert syndrome 34, 614175 (3), Autosomal recessive
BAAT	602938	Bile acid conjugation defect 1, 619232 (3), Autosomal recessive
BACH2	605394	Immunodeficiency 60 and autoimmunity, 618394 (3), Autosomal dominant
BAG3	603883	Cardiomyopathy, dilated, 1HH, 613881 (3), Autosomal dominant; Myopathy, myofibrillar, 6, 612954 (3), Autosomal dominant
BAG5	603885	Cardiomyopathy, dilated, 2F, 619747 (3), Autosomal recessive
BANF1	603811	Nestor-Guillermo progeria syndrome, 614008 (3), Autosomal recessive
BAP1	603089	Kury-Isidor syndrome, 619762 (3), Autosomal dominant; Tumor predisposition syndrome 1, 614327 (3), Autosomal dominant; {Uveal melanoma, susceptibility to, 2}, 606661 (3), Autosomal dominant
BARD1	601593	{Breast cancer, susceptibility to}, 114480 (3), Autosomal dominant, Somatic mutation
BAX	600040	Colorectal cancer, somatic, 114500 (3); T-cell acute lymphoblastic leukemia, somatic, 613065 (3)
BBIP1	613605	?Bardet-Biedl syndrome 18, 615995 (3), Autosomal recessive

BBS1	209901	Bardet-Biedl syndrome 1, 209900 (3), Digenic recessive, Autosomal recessive
BBS10	610148	Bardet-Biedl syndrome 10, 615987 (3), Autosomal recessive
BBS12	610683	Bardet-Biedl syndrome 12, 615989 (3), Autosomal recessive
BBS2	606151	Retinitis pigmentosa 74, 616562 (3), Autosomal recessive; Bardet-Biedl syndrome 2, 615981 (3), Autosomal recessive
BBS4	600374	Bardet-Biedl syndrome 4, 615982 (3), Autosomal recessive
BBS5	603650	Bardet-Biedl syndrome 5, 615983 (3), Autosomal recessive
BBS7	607590	Bardet-Biedl syndrome 7, 615984 (3), Autosomal recessive
BBS9	607968	Bardet-Biedl syndrome 9, 615986 (3), Autosomal recessive
BCAM	612773	[Blood group, Lutheran system], 111200 (3); [Blood group, Auberger system], 111200 (3); [Blood group, Lutheran null], 247420 (3), Autosomal recessive
BCAP31	300398	Deafness, dystonia, and cerebral hypomyelination, 300475 (3), X-linked recessive
BCAS3	607470	Hengel-Maroofian-Schols syndrome, 619641 (3), Autosomal recessive
BCAT2	113530	?Hypervalinemia or hyperleucine-isoleucinemia, 618850 (3), Autosomal recessive
BCHE	177400	Butyrylcholinesterase deficiency, 617936 (3), Autosomal recessive; {Apnea, postanesthetic, susceptibility to, due to BCHE deficiency}, 617936 (3), Autosomal recessive
BCKDHA	608348	Maple syrup urine disease, type Ia, 248600 (3), Autosomal recessive
BCKDHB	248611	Maple syrup urine disease, type Ib, 248600 (3), Autosomal recessive
BCKDK	614901	Branched-chain keto acid dehydrogenase kinase deficiency, 614923 (3)
BCL10	603517	{Lymphoma, follicular, somatic}, 605027 (3); ?Immunodeficiency 37, 616098 (3), Autosomal recessive; {Sezary syndrome, somatic} (3); {Male germ cell tumor, somatic}, 273300 (3); Lymphoma, MALT, somatic, 137245 (3); {Mesothelioma, somatic}, 156240 (3)
BCL11A	606557	Dias-Logan syndrome, 617101 (3), Autosomal dominant
BCL11B	606558	Immunodeficiency 49, severe combined, 617237 (3), Autosomal dominant; Intellectual developmental disorder with dysmorphic facies, speech delay, and T-cell abnormalities, 618092 (3), Autosomal dominant
BCL2	151430	Leukemia/lymphoma, B-cell, 2 (3)
BCL3	109560	Leukemia/lymphoma, B-cell, 3, 109560 (2)
BCL7A	601406	B-cell non-Hodgkin lymphoma, high-grade (3)
BCO1	605748	?Hypercarotenemia and vitamin A deficiency, autosomal dominant, 115300 (3), Autosomal dominant
BCOR	300485	Microphthalmia, syndromic 2, 300166 (3), X-linked dominant
BCORL1	300688	Shukla-Vernon syndrome, 301029 (3), X-linked recessive
BCR	151410	Leukemia, chronic myeloid, Philadelphia chromosome positive, somatic, 608232 (4); Leukemia, acute lymphocytic, Philadelphia chromosome positive, somatic, 613065 (4)
BCS1L	603647	GRACILE syndrome, 603358 (3), Autosomal recessive; Mitochondrial complex III deficiency, nuclear type 1, 124000 (3), Autosomal recessive; Bjornstad syndrome, 262000 (3), Autosomal recessive

BDP1	607012	?Deafness, autosomal recessive 112, 618257 (3), Autosomal recessive
BEAN1	612051	Spinocerebellar ataxia 31, 117210 (3), Autosomal dominant
BEST1	607854	Macular dystrophy, vitelliform, 2, 153700 (3), Autosomal dominant; ?Microcornea, rod-cone dystrophy, cataract, and posterior staphyloma 2, 193220 (3), Autosomal dominant; Retinitis pigmentosa-50, 613194 (3); Retinitis pigmentosa, concentric, 613194 (3); Vitreoretinochoroidopathy, 193220 (3), Autosomal dominant; Bestrophinopathy, autosomal recessive, 611809 (3)
BFSP1	603307	Cataract 33, multiple types, 611391 (3), Autosomal dominant, Autosomal recessive
BFSP2	603212	Cataract 12, multiple types, 611597 (3), Autosomal dominant
BGN	301870	Meester-Loeys syndrome, 300989 (3), X-linked; Spondyloepimetaphyseal dysplasia, X-linked, 300106 (3), X-linked recessive
BHLHA9	615416	?Camptosynpolydactyly, complex, 607539 (3), Autosomal recessive; Syndactyly, mesoaxial synostotic, with phalangeal reduction, 609432 (3), Autosomal recessive
BHLHE41	606200	[Short sleep, familial natural, 1], 612975 (3), Autosomal dominant
BICC1	614295	{Renal dysplasia, cystic, susceptibility to}, 601331 (3), Autosomal dominant
BICD2	609797	Spinal muscular atrophy, lower extremity-predominant, 2B, autosomal dominant, 618291 (3), Autosomal dominant; Spinal muscular atrophy, lower extremity-predominant, 2A, autosomal dominant, 615290 (3), Autosomal dominant
BICRA	605690	Coffin-Siris syndrome 12, 619325 (3), Autosomal dominant
BIN1	601248	Centronuclear myopathy 2, 255200 (3), Autosomal recessive
BLK	191305	Maturity-onset diabetes of the young, type 11, 613375 (3), Autosomal dominant
BLM	604610	Bloom syndrome, 210900 (3), Autosomal recessive
BLNK	604515	?Agammaglobulinemia 4, 613502 (3), Autosomal recessive
BLOC1S3	609762	Hermansky-Pudlak syndrome 8, 614077 (3), Autosomal recessive
BLOC1S5	607289	Hermansky-Pudlak syndrome 11, 619172 (3), Autosomal recessive
BLOC1S6	604310	?Hermansky-Pudlak syndrome 9, 614171 (3), Autosomal recessive
BLTP1 (KIAA1109)	611565	Alkuraya-Kucinskas syndrome, 617822 (3), Autosomal recessive
BLVRA	109750	Hyperbiliverdinemia, 614156 (3), Autosomal dominant, Autosomal recessive
BMP1	112264	Osteogenesis imperfecta, type XIII, 614856 (3), Autosomal recessive
BMP15	300247	Premature ovarian failure 4, 300510 (3), X-linked; Ovarian dysgenesis 2, 300510 (3), X-linked
BMP2	112261	Short stature, facial dysmorphism, and skeletal anomalies with or without cardiac anomalies 1, 617877 (3), Autosomal dominant; Brachydactyly, type A2, 112600 (3), Autosomal dominant; {HFE hemochromatosis, modifier of}, 235200 (3), Autosomal recessive
BMP4	112262	Orofacial cleft 11, 600625 (3); Microphthalmia, syndromic 6, 607932 (3), Autosomal dominant

BMPER	608699	Diaphanospondylodysostosis, 608022 (3), Autosomal recessive
BMPR1A	601299	Polyposis syndrome, hereditary mixed, 2, 610069 (3); Polyposis, juvenile intestinal, 174900 (3), Autosomal dominant
BMPR1B	603248	Acromesomelic dysplasia 3, 609441 (3), Autosomal recessive; Brachydactyly, type A2, 112600 (3), Autosomal dominant; Brachydactyly, type A1, D, 616849 (3), Autosomal dominant
BMPR2	600799	Pulmonary hypertension, familial primary, 1, with or without HHT, 178600 (3), Autosomal dominant; Pulmonary hypertension, primary, fenfluramine or dexfenfluramine-associated, 178600 (3), Autosomal dominant; Pulmonary venoocclusive disease 1, 265450 (3), Autosomal dominant
BMS1	611448	?Aplasia cutis congenita, nonsyndromic, 107600 (3), Autosomal dominant
BNC1	601930	?Premature ovarian failure 16, 618723 (3), Autosomal dominant
BNC2	608669	Lower urinary tract obstruction, congenital, 618612 (3), Autosomal dominant
BOLA3	613183	Multiple mitochondrial dysfunctions syndrome 2 with hyperglycinemia, 614299 (3), Autosomal recessive
BPGM	613896	Erythrocytosis, familial, 8, 222800 (3), Autosomal recessive
BPNT2	614010	Chondrodysplasia with joint dislocations, GPAPP type, 614078 (3), Autosomal recessive
BPTF	601819	Neurodevelopmental disorder with dysmorphic facies and distal limb anomalies, 617755 (3), Autosomal dominant
BRAF	164757	Melanoma, malignant, somatic, 155600 (3); LEOPARD syndrome 3, 613707 (3), Autosomal dominant; Cardiofaciocutaneous syndrome, 115150 (3), Autosomal dominant; Adenocarcinoma of lung, somatic, 211980 (3); Noonan syndrome 7, 613706 (3), Autosomal dominant; Colorectal cancer, somatic, 114500 (3); Nonsmall cell lung cancer, somatic, 211980 (3)
BRAT1	614506	Neurodevelopmental disorder with cerebellar atrophy and with or without seizures, 618056 (3), Autosomal recessive; Rigidity and multifocal seizure syndrome, lethal neonatal, 614498 (3), Autosomal recessive
BRCA1	113705	Fanconi anemia, complementation group S, 617883 (3), Autosomal recessive; {Breast-ovarian cancer, familial, 1}, 604370 (3), Multifactorial, Autosomal dominant; {Pancreatic cancer, susceptibility to, 4}, 614320 (3)
BRCA2	600185	Fanconi anemia, complementation group D1, 605724 (3), Autosomal recessive; {Glioblastoma 3}, 613029 (3), Autosomal recessive; {Medulloblastoma}, 155255 (3), Autosomal dominant, Somatic mutation, Autosomal recessive; {Prostate cancer}, 176807 (3), Autosomal dominant, Somatic mutation; {Breast-ovarian cancer, familial, 2}, 612555 (3), Autosomal dominant; {Breast cancer, male, susceptibility to}, 114480 (3), Autosomal dominant, Somatic mutation; {Pancreatic cancer 2}, 613347 (3); Wilms tumor, 194070 (3), Autosomal dominant, Somatic mutation
BRDT	602144	?Spermatogenic failure 21, 617644 (3), Autosomal recessive
BRF1	604902	Cerebellofaciodental syndrome, 616202 (3), Autosomal recessive
BRIP1	605882	Fanconi anemia, complementation group J, 609054 (3); {Breast cancer, early-onset, susceptibility to}, 114480 (3), Autosomal dominant, Somatic mutation

BRPF1	602410	Intellectual developmental disorder with dysmorphic facies and ptosis, 617333 (3), Autosomal dominant
BRWD3	300553	Intellectual developmental disorder, X-linked 93, 300659 (3), X-linked recessive
BSCL2	606158	Lipodystrophy, congenital generalized, type 2, 269700 (3), Autosomal recessive; Neuropathy, distal hereditary motor, type VC, 619112 (3), Autosomal dominant; Silver spastic paraplegia syndrome, 270685 (3), Autosomal dominant; Encephalopathy, progressive, with or without lipodystrophy, 615924 (3), Autosomal recessive
BSG	109480	[Blood group, OK], 111380 (3)
BSND	606412	Sensorineural deafness with mild renal dysfunction, 602522 (3), Autosomal recessive; Bartter syndrome, type 4a, 602522 (3), Autosomal recessive
BTD	609019	Biotinidase deficiency, 253260 (3), Autosomal recessive
BTG4	605673	Oocyte maturation defect 8, 619009 (3), Autosomal recessive
BTK	300300	Agammaglobulinemia, X-linked 1, 300755 (3), X-linked recessive; Isolated growth hormone deficiency, type III, with agammaglobulinemia, 307200 (3), X-linked recessive
BTNL2	606000	{Sarcoidosis, susceptibility to, 2}, 612387 (3), Autosomal dominant
BUB1	602452	Colorectal cancer with chromosomal instability, somatic, 114500 (3)
BUB1B	602860	Colorectal cancer, somatic, 114500 (3); [Premature chromatid separation trait], 176430 (3), Autosomal dominant; Mosaic variegated aneuploidy syndrome 1, 257300 (3), Autosomal recessive
BVES	604577	Muscular dystrophy, limb-girdle, autosomal recessive 25, 616812 (3), Autosomal recessive
C11orf80	616109	Hydatidiform mole, recurrent, 4, 618432 (3), Autosomal recessive
C12orf4	616082	Intellectual developmental disorder, autosomal recessive 66, 618221 (3), Autosomal recessive
C12orf57	615140	Temtamy syndrome, 218340 (3), Autosomal recessive
C14orf39	617307	Spermatogenic failure 52, 619202 (3), Autosomal recessive; ?Premature ovarian failure 18, 619203 (3), Autosomal recessive
C18orf32	619979	?Glycosylphosphatidylinositol biosynthesis defect 25, 619985 (3), Autosomal recessive
C19orf12	614297	Neurodegeneration with brain iron accumulation 4, 614298 (3), Autosomal dominant, Autosomal recessive; ?Spastic paraplegia 43, autosomal recessive, 615043 (3), Autosomal recessive
C1GALT1C1	300611	Tn polyagglutination syndrome, somatic, 300622 (3)
C1QA	120550	C1q deficiency, 613652 (3), Autosomal recessive
C1QB	120570	C1q deficiency, 613652 (3), Autosomal recessive
C1QBP	601269	Combined oxidative phosphorylation deficiency 33, 617713 (3), Autosomal recessive
C1QC	120575	C1q deficiency, 613652 (3), Autosomal recessive
C1QTNF5	608752	Retinal degeneration, late-onset, autosomal dominant, 605670 (3), Autosomal dominant

C1R	613785	Ehlers-Danlos syndrome, periodontal type, 1, 130080 (3), Autosomal dominant
C1S	120580	C1s deficiency, 613783 (3); Ehlers-Danlos syndrome, periodontal type, 2, 617174 (3), Autosomal dominant
C2	613927	C2 deficiency, 217000 (3), Autosomal recessive; {Macular degeneration, age-related, 14, reduced risk of}, 615489 (3), Digenic dominant
C2CD3	615944	Orofaciodigital syndrome XIV, 615948 (3), Autosomal recessive
C2CD6	619776	?Spermatogenic failure 68, 619805 (3), Autosomal recessive
C2orf69	619219	Combined oxidative phosphorylation deficiency 53, 619423 (3), Autosomal recessive
C3	120700	C3 deficiency, 613779 (3), Autosomal recessive; {Hemolytic uremic syndrome, atypical, susceptibility to, 5}, 612925 (3), Autosomal dominant; {Macular degeneration, age-related, 9}, 611378 (3)
C4A	120810	[Blood group, Rodgers], 614374 (3); C4a deficiency, 614380 (3), Autosomal recessive
C4B	120820	C4B deficiency, 614379 (3)
C5	120900	C5 deficiency, 609536 (3), Autosomal recessive; [Eculizumab, poor response to], 615749 (3), Autosomal dominant
C6	217050	C6 deficiency, 612446 (3), Autosomal recessive; Combined C6/C7 deficiency (3)
C7	217070	C7 deficiency, 610102 (3)
C8A	120950	C8 deficiency, type I, 613790 (3), Autosomal recessive
C8B	120960	C8 deficiency, type II, 613789 (3), Autosomal recessive
C9	120940	C9 deficiency, 613825 (3); {Macular degeneration, age-related, 15, susceptibility to}, 615591 (3), Autosomal dominant
C9orf72	614260	Frontotemporal dementia and/or amyotrophic lateral sclerosis 1, 105550 (3), Autosomal dominant
CA12	603263	Hyperchlorhidrosis, isolated, 143860 (3), Autosomal recessive
CA2	611492	Osteopetrosis, autosomal recessive 3, with renal tubular acidosis, 259730 (3), Autosomal recessive
CA5A	114761	Hyperammonemia due to carbonic anhydrase VA deficiency, 615751 (3), Autosomal recessive
CA8	114815	Cerebellar ataxia and mental retardation with or without quadrupedal locomotion 3, 613227 (3), Autosomal recessive
CABP2	607314	Deafness, autosomal recessive 93, 614899 (3), Autosomal recessive
CABP4	608965	Cone-rod synaptic disorder, congenital nonprogressive, 610427 (3), Autosomal recessive
CACNA1A	601011	Spinocerebellar ataxia 6, 183086 (3), Autosomal dominant; Episodic ataxia, type 2, 108500 (3), Autosomal dominant; Developmental and epileptic encephalopathy 42, 617106 (3), Autosomal dominant; Migraine, familial hemiplegic, 1, with progressive cerebellar ataxia, 141500 (3), Autosomal dominant; Migraine, familial hemiplegic, 1, 141500 (3), Autosomal dominant
CACNA1B	601012	Neurodevelopmental disorder with seizures and nonepileptic hyperkinetic movements, 618497 (3), Autosomal recessive

CACNA1C	114205	Timothy syndrome, 601005 (3), Autosomal dominant; Long QT syndrome 8, 618447 (3), Autosomal dominant; Brugada syndrome 3, 611875 (3)
CACNA1D	114206	Primary aldosteronism, seizures, and neurologic abnormalities, 615474 (3), Autosomal dominant; Sinoatrial node dysfunction and deafness, 614896 (3), Autosomal recessive
CACNA1E	601013	Developmental and epileptic encephalopathy 69, 618285 (3), Autosomal dominant
CACNA1F	300110	Cone-rod dystrophy, X-linked, 3, 300476 (3), X-linked recessive; Night blindness, congenital stationary (incomplete), 2A, X-linked, 300071 (3), X-linked; Aland Island eye disease, 300600 (3), X-linked
CACNA1G	604065	Spinocerebellar ataxia 42, 616795 (3), Autosomal dominant; Spinocerebellar ataxia 42, early-onset, severe, with neurodevelopmental deficits, 618087 (3), Autosomal dominant
CACNA1H	607904	{Epilepsy, childhood absence, susceptibility to, 6}, 611942 (3); Hyperaldosteronism, familial, type IV, 617027 (3), Autosomal dominant; {Epilepsy, idiopathic generalized, susceptibility to, 6}, 611942 (3)
CACNA1S	114208	{Thyrotoxic periodic paralysis, susceptibility to, 1}, 188580 (3), Autosomal dominant; Hypokalemic periodic paralysis, type 1, 170400 (3), Autosomal dominant; {Malignant hyperthermia susceptibility 5}, 601887 (3), Autosomal dominant
CACNA2D1	114204	{Malignant hyperthermia susceptibility 3}, 154276 (2), Autosomal dominant Genomic aberrations of the CACNA2D1 gene in three patients with epilepsy and intellectual disability (Vergult (2015), Eur J Hum Genet 23, 628_632), PMID: 25074461
CACNA2D2	607082	Cerebellar atrophy with seizures and variable developmental delay, 618501 (3), Autosomal recessive
CACNA2D4	608171	Retinal cone dystrophy 4, 610478 (3), Autosomal recessive
CACNB2	600003	Brugada syndrome 4, 611876 (3)
CACNB4	601949	{Epilepsy, juvenile myoclonic, susceptibility to, 6}, 607682 (3), Autosomal dominant; Episodic ataxia, type 5, 613855 (3), Autosomal dominant; {Epilepsy, idiopathic generalized, susceptibility to, 9}, 607682 (3), Autosomal dominant
CACNG2	602911	?Intellectual developmental disorder, autosomal dominant 10, 614256 (3), Autosomal dominant
CAD	114010	Developmental and epileptic encephalopathy 50, 616457 (3), Autosomal recessive
CADM3	609743	Charcot-Marie-Tooth disease, axonal, type 2FF, 619519 (3), Autosomal dominant
CALCR	114131	{Osteoporosis, postmenopausal, susceptibility}, 166710 (3), Autosomal dominant
CALCRL	114190	?Lymphatic malformation 8, 618773 (3), Autosomal recessive
CALM1	114180	Ventricular tachycardia, catecholaminergic polymorphic, 4, 614916 (3), Autosomal dominant; Long QT syndrome 14, 616247 (3), Autosomal dominant

CALM2	114182	Long QT syndrome 15, 616249 (3), Autosomal dominant
CALM3	114183	Long QT syndrome 16, 618782 (3), Autosomal dominant; ?Ventricular tachycardia, catecholaminergic polymorphic 6, 618782 (3), Autosomal dominant
CALR	109091	Myelofibrosis, somatic, 254450 (3); Thrombocythemia, somatic, 187950 (3)
CAMK2A	114078	Intellectual developmental disorder, autosomal dominant 53, 617798 (3), Autosomal dominant; ?Intellectual developmental disorder, autosomal recessive 63, 618095 (3), Autosomal recessive
CAMK2B	607707	Intellectual developmental disorder, autosomal dominant 54, 617799 (3), Autosomal dominant
CAMK2G	602123	Intellectual developmental disorder, autosomal dominant 59, 618522 (3), Autosomal dominant
CAMTA1	611501	Cerebellar dysfunction with variable cognitive and behavioral abnormalities, 614756 (3), Autosomal dominant
CANT1	613165	Desbuquois dysplasia 1, 251450 (3), Autosomal recessive; Epiphyseal dysplasia, multiple, 7, 617719 (3), Autosomal recessive
CAPN1	114220	Spastic paraplegia 76, autosomal recessive, 616907 (3), Autosomal recessive
CAPN10	605286	{Diabetes mellitus, noninsulin-dependent 1}, 601283 (3)
CAPN15	603267	Oculogastrointestinal neurodevelopmental syndrome, 619318 (3), Autosomal recessive
CAPN3	114240	Muscular dystrophy, limb-girdle, autosomal recessive 1, 253600 (3), Autosomal recessive; Muscular dystrophy, limb-girdle, autosomal dominant 4, 618129 (3), Autosomal dominant
CAPN5	602537	Vitreoretinopathy, neovascular inflammatory, 193235 (3), Autosomal dominant
CARD10	607209	?Immunodeficiency 89 and autoimmunity, 619632 (3), Autosomal recessive
CARD11	607210	B-cell expansion with NFKB and T-cell anergy, 616452 (3), Autosomal dominant; Immunodeficiency 11B with atopic dermatitis, 617638 (3), Autosomal dominant; Immunodeficiency 11A, 615206 (3), Autosomal recessive
CARD14	607211	Psoriasis 2, 602723 (3), Autosomal dominant; Pityriasis rubra pilaris, 173200 (3), Autosomal dominant
CARD8	609051	?Inflammatory bowel disease (Crohn disease) 30, 619079 (3), Autosomal dominant
CARD9	607212	Immunodeficiency 103, susceptibility to fungal infection, 212050 (3), Autosomal recessive
CARMIL2	610859	Immunodeficiency 58, 618131 (3), Autosomal recessive
CARS1	123859	Microcephaly, developmental delay, and brittle hair syndrome, 618891 (3), Autosomal recessive
CARS2	612800	Combined oxidative phosphorylation deficiency 27, 616672 (3), Autosomal recessive

CARTPT	602606	{?Obesity, susceptibility to}, 601665 (3), Multifactorial, Autosomal dominant, Autosomal recessive
CASK	300172	Intellectual developmental disorder, with or without nystagmus, 300422 (3); Intellectual developmental disorder and microcephaly with pontine and cerebellar hypoplasia, 300749 (3), X-linked dominant; FG syndrome 4, 300422 (3)
CASP10	601762	Autoimmune lymphoproliferative syndrome, type II, 603909 (3), Autosomal dominant; Gastric cancer, somatic, 613659 (3); Lymphoma, non-Hodgkin, somatic, 605027 (3)
CASP12	608633	{Sepsis, susceptibility to} (3)
CASP14	605848	Ichthyosis, congenital, autosomal recessive 12, 617320 (3), Autosomal recessive
CASP8	601763	{Breast cancer, protection against}, 114480 (3), Autosomal dominant, Somatic mutation; Hepatocellular carcinoma, somatic, 114550 (3); ?Autoimmune lymphoproliferative syndrome, type IIB, 607271 (3), Autosomal recessive; {Lung cancer, protection against}, 211980 (3), Autosomal dominant, Somatic mutation
CASQ1	114250	Myopathy, vacuolar, with CASQ1 aggregates, 616231 (3), Autosomal dominant
CASQ2	114251	Ventricular tachycardia, catecholaminergic polymorphic, 2, 611938 (3), Autosomal recessive
CASR	601199	Hypocalcemia, autosomal dominant, with Bartter syndrome, 601198 (3), Autosomal dominant; {Epilepsy idiopathic generalized, susceptibility to, 8}, 612899 (3); Hyperparathyroidism, neonatal, 239200 (3), Autosomal dominant, Autosomal recessive; Hypocalcemia, autosomal dominant, 601198 (3), Autosomal dominant; Hypocalciuric hypercalcemia, type I, 145980 (3), Autosomal dominant
CAST	114090	Peeling skin with leukonychia, acral punctate keratoses, cheilitis, and knuckle pads, 616295 (3), Autosomal recessive
CAT	115500	Acatalasemia, 614097 (3)
CATIP	619387	?Spermatogenic failure 54, 619379 (3), Autosomal recessive
CATSPER1	606389	Spermatogenic failure 7, 612997 (3), Autosomal recessive
CAV1	601047	?Lipodystrophy, congenital generalized, type 3, 612526 (3), Autosomal recessive; Pulmonary hypertension, primary, 3, 615343 (3), Autosomal dominant; Lipodystrophy, familial partial, type 7, 606721 (3), Autosomal dominant
CAV3	601253	Myopathy, distal, Tateyama type, 614321 (3), Autosomal dominant; Creatine phosphokinase, elevated serum, 123320 (3), Autosomal dominant; Cardiomyopathy, familial hypertrophic, 192600 (3), Digenic dominant, Autosomal dominant; Rippling muscle disease 2, 606072 (3), Autosomal dominant; Long QT syndrome 9, 611818 (3), Autosomal dominant
CAVIN1	603198	Lipodystrophy, congenital generalized, type 4, 613327 (3), Autosomal recessive
CBFB	121360	Myeloid leukemia, acute, M4/M4Eo subtype, somatic, 601626 (1)

CBL	165360	Noonan syndrome-like disorder with or without juvenile myelomonocytic leukemia, 613563 (3), Autosomal dominant; ?Juvenile myelomonocytic leukemia, 607785 (3), Autosomal dominant, Somatic mutation
CBLIF	609342	Intrinsic factor deficiency, 261000 (3), Autosomal recessive
CBS	613381	Thrombosis, hyperhomocysteinemic, 236200 (3), Autosomal recessive; Homocystinuria, B6-responsive and nonresponsive types, 236200 (3), Autosomal recessive
CBX2	602770	?46XY sex reversal 5, 613080 (3), Autosomal recessive
CC2D1A	610055	Intellectual developmental disorder, autosomal recessive 3, 608443 (3), Autosomal recessive
CC2D2A	612013	COACH syndrome 2, 619111 (3), Autosomal recessive; Retinitis pigmentosa 93, 619845 (3), Autosomal recessive; Meckel syndrome 6, 612284 (3), Autosomal recessive; Joubert syndrome 9, 612285 (3), Autosomal recessive
CCBE1	612753	Hennekam lymphangiectasia-lymphedema syndrome 1, 235510 (3), Autosomal recessive
CCDC103	614677	Ciliary dyskinesia, primary, 17, 614679 (3), Autosomal recessive
CCDC115	613734	Congenital disorder of glycosylation, type IIo, 616828 (3), Autosomal recessive
CCDC134	618788	Osteogenesis imperfecta, type XXII, 619795 (3), Autosomal recessive
CCDC174	616735	Hypotonia, infantile, with psychomotor retardation, 616816 (3), Autosomal recessive
CCDC22	300859	Ritscher-Schinzel syndrome 2, 300963 (3), X-linked recessive
CCDC28B	610162	{Bardet-Biedl syndrome 1, modifier of}, 209900 (3), Digenic recessive, Autosomal recessive
CCDC32	618941	Cardiofacioneurodevelopmental syndrome, 619123 (3), Autosomal recessive
CCDC39	613798	Ciliary dyskinesia, primary, 14, 613807 (3), Autosomal recessive
CCDC40	613799	Ciliary dyskinesia, primary, 15, 613808 (3), Autosomal recessive
CCDC47	618260	Trichohepatoneurodevelopmental syndrome, 618268 (3), Autosomal recessive
CCDC50	611051	?Deafness, autosomal dominant 44, 607453 (3), Autosomal dominant
CCDC62	613481	?Spermatogenic failure 67, 619803 (3), Autosomal recessive
CCDC65	611088	Ciliary dyskinesia, primary, 27, 615504 (3), Autosomal recessive
CCDC78	614666	?Centronuclear myopathy 4, 614807 (3), Autosomal dominant
CCDC8	614145	3-M syndrome 3, 614205 (3), Autosomal recessive
CCDC88A	609736	?PEHO syndrome-like, 617507 (3), Autosomal recessive
CCDC88C	611204	?Spinocerebellar ataxia 40, 616053 (3), Autosomal dominant; Hydrocephalus, congenital, 1, 236600 (3), Autosomal recessive
CCL11	601156	{Asthma, susceptibility to}, 600807 (3), Autosomal dominant; {HIV1, resistance to}, 609423 (3)
CCL2	158105	{Mycobacterium tuberculosis, susceptibility to}, 607948 (3); {HIV-1, resistance to}, 609423 (3); {Coronary artery disease, modifier of} (3); {Spina bifida, susceptibility to}, 182940 (3), Autosomal dominant
CCL3	182283	{HIV infection, resistance to}, 609423 (2)

CCL5	187011	{HIV-1 disease, rapid progression of}, 609423 (3); {HIV-1 disease, delayed progression of}, 609423 (3)
CCM2	607929	Cerebral cavernous malformations-2, 603284 (3), Autosomal dominant
CCN6	603400	Progressive pseudorheumatoid dysplasia, 208230 (3), Autosomal recessive
CCND1	168461	{von Hippel-Lindau syndrome, modifier of}, 193300 (3), Autosomal dominant; {Colorectal cancer, susceptibility to}, 114500 (3), Autosomal dominant, Somatic mutation; {Multiple myeloma, susceptibility to}, 254500 (3), Somatic mutation
CCND2	123833	Megalencephaly-polymicrogyria-polydactyly-hydrocephalus syndrome 3, 615938 (3), Autosomal dominant
CCNF	600227	Frontotemporal dementia and/or amyotrophic lateral sclerosis 5, 619141 (3), Autosomal dominant
CCNK	603544	?Intellectual developmental disorder with hypertelorism and distinctive facies, 618147 (3), Autosomal dominant
CCNO	607752	Ciliary dyskinesia, primary, 29, 615872 (3), Autosomal recessive
CCNQ	300708	STAR syndrome, 300707 (3), X-linked dominant
CCR2	601267	{HIV infection, susceptibility/resistance to} (3)
CCR5	601373	{HIV infection, susceptibility/resistance to} (3); {Diabetes mellitus, insulin-dependent, 22}, 612522 (3); {Hepatitis C virus, resistance to}, 609532 (3); {West nile virus, susceptibility to}, 610379 (3)
CCT5	610150	Neuropathy, hereditary sensory, with spastic paraplegia, 256840 (3), Autosomal recessive
CD151	602243	[Blood group, Raph], 179620 (3); Epidermolysis bullosa simplex 7, with nephropathy and deafness, 609057 (3), Autosomal recessive
CD164	603356	?Deafness, autosomal dominant 66, 616969 (3), Autosomal dominant
CD19	107265	Immunodeficiency, common variable, 3, 613493 (3), Autosomal recessive
CD207	604862	[?Birbeck granule deficiency], 613393 (3)
CD209	604672	{HIV type 1, susceptibility to}, 609423 (3); {Mycobacterium tuberculosis, susceptibility to}, 607948 (3); {Dengue fever, protection against}, 614371 (3)
CD244	605554	{Rheumatoid arthritis, susceptibility to}, 180300 (3)
CD247	186780	?Immunodeficiency 25, 610163 (3), Autosomal recessive
CD27	186711	Lymphoproliferative syndrome 2, 615122 (3), Autosomal recessive
CD2AP	604241	Glomerulosclerosis, focal segmental, 3, 607832 (3)
CD320	606475	Methylmalonic aciduria, transient, due to transcobalamin receptor defect, 613646 (3), Autosomal recessive
CD36	173510	Platelet glycoprotein IV deficiency, 608404 (3), Autosomal recessive; {Coronary heart disease, susceptibility to, 7}, 610938 (3); {Malaria, cerebral, susceptibility to}, 611162 (3); {Malaria, cerebral, reduced risk of}, 611162 (3)
CD3D	186790	Immunodeficiency 19, severe combined, 615617 (3), Autosomal recessive
CD3E	186830	Immunodeficiency 18, 615615 (3), Autosomal recessive; Immunodeficiency 18, SCID variant, 615615 (3), Autosomal recessive
CD3G	186740	Immunodeficiency 17, CD3 gamma deficient, 615607 (3), Autosomal recessive

CD4	186940	Immunodeficiency 79, 619238 (3), Autosomal recessive; OKT4 epitope deficiency, 613949 (3)
CD40	109535	Immunodeficiency with hyper-IgM, type 3, 606843 (3), Autosomal recessive
CD40LG	300386	Immunodeficiency, X-linked, with hyper-IgM, 308230 (3), X-linked recessive
CD44	107269	[Blood group, Indian system], 609027 (3)
CD46	120920	{Hemolytic uremic syndrome, atypical, susceptibility to, 2}, 612922 (3), Autosomal dominant, Autosomal recessive
CD55	125240	[Blood group Cromer], 613793 (3), Autosomal recessive; Complement hyperactivation, angiopathic thrombosis, and protein-losing enteropathy, 226300 (3), Autosomal recessive
CD59	107271	Hemolytic anemia, CD59-mediated, with or without immune-mediated polyneuropathy, 612300 (3), Autosomal recessive
CD70	602840	Lymphoproliferative syndrome 3, 618261 (3), Autosomal recessive
CD79A	112205	Agammaglobulinemia 3, 613501 (3), Autosomal recessive
CD79B	147245	Agammaglobulinemia 6, 612692 (3), Autosomal recessive
CD81	186845	Immunodeficiency, common variable, 6, 613496 (3), Autosomal recessive
CD8A	186910	CD8 deficiency, familial, 608957 (3), Autosomal recessive
CD96	606037	C syndrome, 211750 (3), Autosomal dominant
CDAN1	607465	Dyserythropoietic anemia, congenital, type Ia, 224120 (3), Autosomal recessive
CDC14A	603504	Deafness, autosomal recessive 32, with or without immotile sperm, 608653 (3), Autosomal recessive
CDC40	605585	?Pontocerebellar hypoplasia, type 15, 619302 (3), Autosomal recessive
CDC42	116952	Takenouchi-Kosaki syndrome, 616737 (3), Autosomal dominant
CDC42BPB	614062	Chilton-Okur-Chung neurodevelopmental syndrome, 619841 (3)
CDC45	603465	Meier-Gorlin syndrome 7, 617063 (3), Autosomal recessive
CDC6	602627	?Meier-Gorlin syndrome 5, 613805 (3), Autosomal recessive
CDC73	607393	Hyperparathyroidism, familial primary, 145000 (3), Autosomal dominant; Parathyroid adenoma with cystic changes, 145001 (3), Autosomal dominant; Parathyroid carcinoma, 608266 (3); Hyperparathyroidism-jaw tumor syndrome, 145001 (3), Autosomal dominant
CDCA7	609937	Immunodeficiency-centromeric instability-facial anomalies syndrome 3, 616910 (3), Autosomal recessive
CDH1	192090	Ovarian cancer, somatic, 167000 (3); Blepharocheilodontic syndrome 1, 119580 (3), Autosomal dominant; Diffuse gastric and lobular breast cancer syndrome with or without cleft lip and/or palate, 137215 (3), Autosomal dominant; Endometrial carcinoma, somatic, 608089 (3); Breast cancer, lobular, somatic, 114480 (3); {Prostate cancer, susceptibility to}, 176807 (3), Autosomal dominant, Somatic mutation
CDH11	600023	Teebi hypertelorism syndrome 2, 619736 (3), Autosomal dominant; Elsahy-Waters syndrome, 211380 (3), Autosomal recessive
CDH15	114019	Intellectual developmental disorder, autosomal dominant 3, 612580 (3)

CDH2	114020	Arrhythmogenic right ventricular dysplasia, familial, 14, 618920 (3), Autosomal dominant; ?Attention deficit-hyperactivity disorder 8, 619957 (3), Autosomal recessive; Agenesis of corpus callosum, cardiac, ocular, and genital syndrome, 618929 (3), Autosomal dominant
CDH23	605516	Usher syndrome, type 1D, 601067 (3), Digenic recessive, Autosomal recessive; {Pituitary adenoma 5, multiple types}, 617540 (3), Autosomal dominant; Usher syndrome, type 1D/F digenic, 601067 (3), Digenic recessive, Autosomal recessive; Deafness, autosomal recessive 12, 601386 (3), Autosomal recessive
CDH3	114021	Hypotrichosis, congenital, with juvenile macular dystrophy, 601553 (3), Autosomal recessive; Ectodermal dysplasia, ectrodactyly, and macular dystrophy, 225280 (3), Autosomal recessive
CDHR1	609502	Cone-rod dystrophy 15, 613660 (3), Autosomal recessive; Retinitis pigmentosa 65, 613660 (3), Autosomal recessive
CDIN1	615626	Dyserythropoietic anemia, congenital, type Ib, 615631 (3), Autosomal recessive
CDK10	603464	Al Kaissi syndrome, 617694 (3), Autosomal recessive
CDK13	603309	Congenital heart defects, dysmorphic facial features, and intellectual developmental disorder, 617360 (3), Autosomal dominant
CDK19	614720	Developmental and epileptic encephalopathy 87, 618916 (3), Autosomal dominant
CDK4	123829	{Melanoma, cutaneous malignant, 3}, 609048 (3), Autosomal dominant
CDK5	123831	?Lissencephaly 7 with cerebellar hypoplasia, 616342 (3), Autosomal recessive
CDK5RAP2	608201	Microcephaly 3, primary, autosomal recessive, 604804 (3), Autosomal recessive
CDK6	603368	?Microcephaly 12, primary, autosomal recessive, 616080 (3), Autosomal recessive
CDK8	603184	Intellectual developmental disorder with hypotonia and behavioral abnormalities, 618748 (3), Autosomal dominant
CDKL5	300203	Developmental and epileptic encephalopathy 2, 300672 (3), X-linked dominant
CDKN1B	600778	Multiple endocrine neoplasia, type IV, 610755 (3), Autosomal dominant
CDKN1C	600856	IMAGE syndrome, 614732 (3), Autosomal dominant; Beckwith-Wiedemann syndrome, 130650 (3), Autosomal dominant
CDKN2A	600160	{Melanoma and neural system tumor syndrome}, 155755 (3), Autosomal dominant; {Melanoma, cutaneous malignant, 2}, 155601 (3), Autosomal dominant; {Melanoma-pancreatic cancer syndrome}, 606719 (3), Autosomal dominant
CDON	608707	Holoprosencephaly 11, 614226 (3), Autosomal dominant
CDSN	602593	Hypotrichosis 2, 146520 (3), Autosomal dominant; Peeling skin syndrome 1, 270300 (3), Autosomal recessive
CDT1	605525	Meier-Gorlin syndrome 4, 613804 (3), Autosomal recessive

CEACAM16	614591	Deafness, autosomal dominant 4B, 614614 (3), Autosomal dominant; Deafness, autosomal recessive 113, 618410 (3), Autosomal recessive
CEBPA	116897	Leukemia, acute myeloid, somatic, 601626 (3); ?Leukemia, acute myeloid, 601626 (3), Autosomal dominant, Somatic mutation
CEBPE	600749	Specific granule deficiency, 245480 (3), Autosomal recessive
CEL	114840	Maturity-onset diabetes of the young, type VIII, 609812 (3), Autosomal dominant
CELA2A	609443	Abdominal obesity-metabolic syndrome 4, 618620 (3), Autosomal dominant
CELF2	602538	Developmental and epileptic encephalopathy 97, 619561 (3), Autosomal dominant
CELSR1	604523	Lymphatic malformation 9, 619319 (3), Autosomal dominant
CENPE	117143	?Microcephaly 13, primary, autosomal recessive, 616051 (3), Autosomal recessive
CENPF	600236	Stromme syndrome, 243605 (3), Autosomal recessive
CENPJ	609279	Microcephaly 6, primary, autosomal recessive, 608393 (3), Autosomal recessive; ?Seckel syndrome 4, 613676 (3), Autosomal recessive
CENPT	611510	?Short stature and microcephaly with genital anomalies, 618702 (3), Autosomal recessive
CEP104	616690	Joubert syndrome 25, 616781 (3), Autosomal recessive; Intellectual developmental disorder, autosomal recessive 77, 619988 (3), Autosomal recessive
CEP112	618980	Spermatogenic failure 44, 619044 (3), Autosomal recessive
CEP120	613446	Short-rib thoracic dysplasia 13 with or without polydactyly, 616300 (3), Autosomal recessive; Joubert syndrome 31, 617761 (3), Autosomal recessive
CEP135	611423	Microcephaly 8, primary, autosomal recessive, 614673 (3), Autosomal recessive
CEP152	613529	Microcephaly 9, primary, autosomal recessive, 614852 (3), Autosomal recessive; Seckel syndrome 5, 613823 (3), Autosomal recessive
CEP164	614848	Nephronophthisis 15, 614845 (3), Autosomal recessive
CEP19	615586	Morbid obesity and spermatogenic failure, 615703 (3), Autosomal recessive
CEP250	609689	Cone-rod dystrophy and hearing loss 2, 618358 (3), Autosomal recessive
CEP290	610142	Leber congenital amaurosis 10, 611755 (3); Joubert syndrome 5, 610188 (3), Autosomal recessive; Senior-Loken syndrome 6, 610189 (3), Autosomal recessive; ?Bardet-Biedl syndrome 14, 615991 (3), Autosomal recessive; Meckel syndrome 4, 611134 (3), Autosomal recessive
CEP41	610523	Joubert syndrome 15, 614464 (3), Autosomal recessive
CEP43	605392	Myeloproliferative disorder, 605392 (2)
CEP55	610000	Multinucleated neurons, anhydramnios, renal dysplasia, cerebellar hypoplasia, and hydranencephaly, 236500 (3), Autosomal recessive
CEP57	607951	Mosaic variegated aneuploidy syndrome 2, 614114 (3), Autosomal recessive
CEP63	614724	?Seckel syndrome 6, 614728 (3), Autosomal recessive

CEP78	617110	Cone-rod dystrophy and hearing loss, 617236 (3), Autosomal recessive
CEP83	615847	Nephronophthisis 18, 615862 (3), Autosomal recessive
CEP85L	618865	Lissencephaly 10, 618873 (3), Autosomal dominant
CERKL	608381	Retinitis pigmentosa 26, 608380 (3), Autosomal recessive
CERS1	606919	Epilepsy, progressive myoclonic, 8, 616230 (3), Autosomal recessive
CERS3	615276	Ichthyosis, congenital, autosomal recessive 9, 615023 (3), Autosomal recessive
CERT1	604677	Intellectual developmental disorder, autosomal dominant 34, 616351 (3), Autosomal dominant
CES1	114835	Drug metabolism, altered, CES1-related, 618057 (3), Autosomal dominant
CETP	118470	[High density lipoprotein cholesterol level QTL 10], 143470 (3), Autosomal dominant; Hyperalphalipoproteinemia, 143470 (3), Autosomal dominant
CFAP251	618146	Spermatogenic failure 33, 618152 (3), Autosomal recessive
CFAP298	615494	Ciliary dyskinesia, primary, 26, 615500 (3), Autosomal recessive
CFAP300	618058	Ciliary dyskinesia, primary, 38, 618063 (3), Autosomal recessive
CFAP410	603191	Retinal dystrophy with macular staphyloma, 617547 (3), Autosomal recessive; Spondylometaphyseal dysplasia, axial, 602271 (3), Autosomal recessive
CFAP418	614477	Retinitis pigmentosa 64, 614500 (3), Autosomal recessive; Cone-rod dystrophy 16, 614500 (3), Autosomal recessive; Bardet-Biedl syndrome 21, 617406 (3), Autosomal recessive
CFAP43	617558	Hydrocephalus, normal pressure, 1, 236690 (3), Autosomal dominant; Spermatogenic failure 19, 617592 (3), Autosomal recessive
CFAP44	617559	?Spermatogenic failure 20, 617593 (3), Autosomal recessive
CFAP45	605152	Heterotaxy, visceral, 11, autosomal, with male infertility, 619608 (3), Autosomal recessive
CFAP47	301057	Spermatogenic failure, X-linked 3, 301059 (3), X-linked recessive
CFAP52	609804	Heterotaxy, visceral, 10, autosomal, with male infertility, 619607 (3), Autosomal recessive
CFAP53	614759	Heterotaxy, visceral, 6, autosomal recessive, 614779 (3), Autosomal recessive
CFAP58	619129	Spermatogenic failure 49, 619144 (3), Autosomal recessive
CFAP65	614270	Spermatogenic failure 40, 618664 (3), Autosomal recessive
CFAP69	617949	Spermatogenic failure 24, 617959 (3), Autosomal recessive
CFAP70	618661	?Spermatogenic failure 41, 618670 (3), Autosomal recessive
CFAP91	609910	Spermatogenic failure 51, 619177 (3), Autosomal recessive
CFB	138470	?Complement factor B deficiency, 615561 (3), Autosomal recessive; {Hemolytic uremic syndrome, atypical, susceptibility to, 4}, 612924 (3), Autosomal dominant; {Macular degeneration, age-related, 14, reduced risk of}, 615489 (3), Digenic dominant
CFC1	605194	Heterotaxy, visceral, 2, autosomal, 605376 (3), Autosomal dominant
CFD	134350	Complement factor D deficiency, 613912 (3), Autosomal recessive

CFH	134370	{Macular degeneration, age-related, 4}, 610698 (3); Basal laminar drusen, 126700 (3), Autosomal dominant; Complement factor H deficiency, 609814 (3), Autosomal dominant, Autosomal recessive; {Hemolytic uremic syndrome, atypical, susceptibility to, 1}, 235400 (3), Autosomal dominant, Autosomal recessive
CFHR1	134371	{Macular degeneration, age-related, reduced risk of}, 603075 (3), Autosomal dominant; {Hemolytic uremic syndrome, atypical, susceptibility to}, 235400 (3), Autosomal dominant, Autosomal recessive
CFHR3	605336	{Macular degeneration, age-related, reduced risk of}, 603075 (3), Autosomal dominant; {Hemolytic uremic syndrome, atypical, susceptibility to}, 235400 (3), Autosomal dominant, Autosomal recessive
CFHR5	608593	Nephropathy due to CFHR5 deficiency, 614809 (3), Autosomal dominant
CFI	217030	{Hemolytic uremic syndrome, atypical, susceptibility to, 3}, 612923 (3), Autosomal dominant; {Macular degeneration, age-related, 13, susceptibility to}, 615439 (3), Autosomal dominant; Complement factor I deficiency, 610984 (3), Autosomal recessive
CFL2	601443	Nemaline myopathy 7, autosomal recessive, 610687 (3), Autosomal recessive
CFP	300383	Properdin deficiency, X-linked, 312060 (3), X-linked recessive
CFTR	602421	Cystic fibrosis, 219700 (3), Autosomal recessive; Sweat chloride elevation without CF (3); Congenital bilateral absence of vas deferens, 277180 (3), Autosomal recessive; {Pancreatitis, hereditary}, 167800 (3), Autosomal dominant; {Bronchiectasis with or without elevated sweat chloride 1, modifier of}, 211400 (3), Autosomal dominant; {Hypertrypsinemia, neonatal} (3)
CHAMP1	616327	Intellectual developmental disorder, autosomal dominant 40, 616579 (3), Autosomal dominant
CHAT	118490	Myasthenic syndrome, congenital, 6, presynaptic, 254210 (3), Autosomal recessive
CHCHD10	615903	?Myopathy, isolated mitochondrial, autosomal dominant, 616209 (3), Autosomal dominant; Spinal muscular atrophy, Jokela type, 615048 (3), Autosomal dominant; Frontotemporal dementia and/or amyotrophic lateral sclerosis 2, 615911 (3), Autosomal dominant
CHCHD2	616244	Parkinson disease 22, autosomal dominant, 616710 (3), Autosomal dominant
CHD1	602118	Pilarowski-Bjornsson syndrome, 617682 (3), Autosomal dominant
CHD2	602119	Developmental and epileptic encephalopathy 94, 615369 (3), Autosomal dominant
CHD3	602120	Snijders Blok-Campeau syndrome, 618205 (3), Autosomal dominant
CHD4	603277	Sifrim-Hitz-Weiss syndrome, 617159 (3), Autosomal dominant
CHD5	610771	Parenti-Mignot neurodevelopmental syndrome, 619873 (3), Autosomal dominant
CHD7	608892	Hypogonadotropic hypogonadism 5 with or without anosmia, 612370 (3), Autosomal dominant; CHARGE syndrome, 214800 (3), Autosomal dominant

CHD8	610528	Intellectual developmental disorder with autism and macrocephaly, 615032 (3), Autosomal dominant
CHEK2	604373	{Colorectal cancer, susceptibility to}, 114500 (3), Autosomal dominant, Somatic mutation; Osteosarcoma, somatic, 259500 (3); {Breast cancer, susceptibility to}, 114480 (3), Autosomal dominant, Somatic mutation; {Prostate cancer, familial, susceptibility to}, 176807 (3), Autosomal dominant, Somatic mutation; Li-Fraumeni syndrome 2, 609265 (3)
CHI3L1	601525	{Asthma-related traits, susceptibility to, 7}, 611960 (3); {Schizophrenia, susceptibility to}, 181500 (3), Autosomal dominant
CHIC2	604332	{Leukemia, acute myeloid}, 601626 (3), Autosomal dominant, Somatic mutation
CHIT1	600031	[Chitotriosidase deficiency], 614122 (3), Autosomal recessive
CHKB	612395	Muscular dystrophy, congenital, megaconial type, 602541 (3), Autosomal recessive
CHM	300390	Choroideremia, 303100 (3), X-linked
CHMP1A	164010	Pontocerebellar hypoplasia, type 8, 614961 (3), Autosomal recessive
CHMP2B	609512	Frontotemporal dementia and/or amyotrophic lateral sclerosis 7, 600795 (3), Autosomal dominant
CHMP4B	610897	Cataract 31, multiple types, 605387 (3), Autosomal dominant
CHN1	118423	Duane retraction syndrome 2, 604356 (3), Autosomal dominant
CHP1	606988	?Spastic ataxia 9, autosomal recessive, 618438 (3), Autosomal recessive
CHRDL1	300350	Megalocornea 1, X-linked, 309300 (3), X-linked recessive
CHRM3	118494	Prune belly syndrome, 100100 (3), Autosomal recessive
CHRNA1	100690	Myasthenic syndrome, congenital, 1B, fast-channel, 608930 (3), Autosomal dominant, Autosomal recessive; Myasthenic syndrome, congenital, 1A, slow-channel, 601462 (3), Autosomal dominant; Multiple pterygium syndrome, lethal type, 253290 (3), Autosomal recessive
CHRNA2	118502	Epilepsy, nocturnal frontal lobe, type 4, 610353 (3), Autosomal dominant
CHRNA3	118503	{Lung cancer susceptibility 2}, 612052 (3); Bladder dysfunction, autonomic, with impaired pupillary reflex and secondary CAKUT, 191800 (3), Autosomal recessive
CHRNA4	118504	{Nicotine addiction, susceptibility to}, 188890 (3); Epilepsy, nocturnal frontal lobe, 1, 600513 (3), Autosomal dominant
CHRNA5	118505	{Nicotine dependence, susceptibility to}, 612052 (3); {Lung cancer susceptibility 2}, 612052 (3)
CHRNB1	100710	?Myasthenic syndrome, congenital, 2C, associated with acetylcholine receptor deficiency, 616314 (3), Autosomal recessive; Myasthenic syndrome, congenital, 2A, slow-channel, 616313 (3), Autosomal dominant
CHRNB2	118507	Epilepsy, nocturnal frontal lobe, 3, 605375 (3)

CHRND	100720	?Myasthenic syndrome, congenital, 3C, associated with acetylcholine receptor deficiency, 616323 (3), Autosomal recessive; Multiple pterygium syndrome, lethal type, 253290 (3), Autosomal recessive; Myasthenic syndrome, congenital, 3B, fast-channel, 616322 (3), Autosomal recessive; ?Myasthenic syndrome, congenital, 3A, slow-channel, 616321 (3), Autosomal dominant
CHRNE	100725	Myasthenic syndrome, congenital, 4A, slow-channel, 605809 (3), Autosomal dominant, Autosomal recessive; Myasthenic syndrome, congenital, 4C, associated with acetylcholine receptor deficiency, 608931 (3), Autosomal recessive; Myasthenic syndrome, congenital, 4B, fast-channel, 616324 (3), Autosomal recessive
CHRNG	100730	Multiple pterygium syndrome, lethal type, 253290 (3), Autosomal recessive; Escobar syndrome, 265000 (3), Autosomal recessive
CHST11	610128	?Osteochondrodysplasia, brachydactyly, and overlapping malformed digits, 618167 (3), Autosomal recessive
CHST14	608429	Ehlers-Danlos syndrome, musculocontractural type 1, 601776 (3), Autosomal recessive
CHST3	603799	Spondyloepiphyseal dysplasia with congenital joint dislocations, 143095 (3), Autosomal recessive
CHST6	605294	Macular corneal dystrophy, 217800 (3), Autosomal recessive
CHST8	610190	?Peeling skin syndrome 3, 616265 (3), Autosomal recessive
CHSY1	608183	Temtamy preaxial brachydactyly syndrome, 605282 (3), Autosomal recessive
CHUK	600664	?Popliteal pterygium syndrome, Bartsocas-Papas type 2, 619339 (3), Autosomal recessive; ?Cocoon syndrome, 613630 (3), Autosomal recessive
CIB1	602293	Epidermodysplasia verruciformis 3, 618267 (3), Autosomal recessive
CIB2	605564	Deafness, autosomal recessive 48, 609439 (3), Autosomal recessive; Usher syndrome, type IJ, 614869 (3), Autosomal recessive
CIBAR1	617273	?Polydactyly, postaxial, type A9, 618219 (3), Autosomal recessive
CIC	612082	Intellectual developmental disorder, autosomal dominant 45, 617600 (3), Autosomal dominant
CIDEC	612120	?Lipodystrophy, familial partial, type 5, 615238 (3), Autosomal recessive
CIITA	600005	{Rheumatoid arthritis, susceptibility to}, 180300 (3); Bare lymphocyte syndrome, type II, complementation group A, 209920 (3), Autosomal recessive
CILK1	612325	{Epilepsy, juvenile myoclonic, susceptibility to, 10}, 617924 (3), Autosomal dominant; Endocrine-cerebroosteodysplasia, 612651 (3), Autosomal recessive
CILP	603489	{Lumbar disc disease, susceptibility to}, 603932 (3)
CIROP (LMLN2)	619703	Heterotaxy, visceral, 12, autosomal, 619702 (3), Autosomal recessive
CISD2	611507	Wolfram syndrome 2, 604928 (3), Autosomal recessive
CISH	602441	{Malaria, susceptibility to}, 611162 (3); {Bacteremia, susceptibility to}, 614383 (3); {Tuberculosis, susceptibility to}, 607948 (3)

CIT	605629	Microcephaly 17, primary, autosomal recessive, 617090 (3), Autosomal recessive
CITED2	602937	Atrial septal defect 8, 614433 (3), Autosomal dominant; Ventricular septal defect 2, 614431 (3), Autosomal dominant
CKAP2L	616174	Filippi syndrome, 272440 (3), Autosomal recessive
CLCC1	617539	Retinitis pigmentosa 32, 609913 (3), Autosomal recessive
CLCF1	607672	Cold-induced sweating syndrome 2, 610313 (3), Autosomal recessive
CLCN1	118425	Myotonia levior, recessive (3); Myotonia congenita, recessive, 255700 (3), Autosomal recessive; Myotonia congenita, dominant, 160800 (3), Autosomal dominant
CLCN2	600570	Leukoencephalopathy with ataxia, 615651 (3), Autosomal recessive; Hyperaldosteronism, familial, type II, 605635 (3), Autosomal dominant; {Epilepsy, juvenile myoclonic, susceptibility to, 8}, 607628 (3), Autosomal dominant; {Epilepsy, juvenile absence, susceptibility to, 2}, 607628 (3), Autosomal dominant; {Epilepsy, idiopathic generalized, susceptibility to, 11}, 607628 (3), Autosomal dominant
CLCN3	600580	Neurodevelopmental disorder with seizures and brain abnormalities, 619517 (3), Autosomal recessive; Neurodevelopmental disorder with hypotonia and brain abnormalities, 619512 (3), Autosomal dominant
CLCN4	302910	Raynaud-Claes syndrome, 300114 (3), X-linked dominant
CLCN5	300008	Proteinuria, low molecular weight, with hypercalciuric nephrocalcinosis, 308990 (3), X-linked recessive; Hypophosphatemic rickets, 300554 (3), X-linked recessive; Dent disease 1, 300009 (3), X-linked recessive; Nephrolithiasis, type I, 310468 (3), X-linked recessive
CLCN6	602726	Neurodegeneration, childhood-onset, hypotonia, respiratory insufficiency and brain imaging abnormalities, 619173 (3), Autosomal dominant
CLCN7	602727	Hypopigmentation, organomegaly, and delayed myelination and development, 618541 (3), Autosomal dominant; Osteopetrosis, autosomal recessive 4, 611490 (3), Autosomal recessive; Osteopetrosis, autosomal dominant 2, 166600 (3), Autosomal dominant
CLCNKA	602024	Bartter syndrome, type 4b, digenic, 613090 (3), Digenic recessive
CLCNKB	602023	Bartter syndrome, type 3, 607364 (3), Autosomal recessive; Bartter syndrome, type 4b, digenic, 613090 (3), Digenic recessive
CLDN1	603718	Ichthyosis, leukocyte vacuoles, alopecia, and sclerosing cholangitis, 607626 (3), Autosomal recessive
CLDN10	617579	HELIX syndrome, 617671 (3), Autosomal recessive
CLDN11	601326	Leukodystrophy, hypomyelinating, 22, 619328 (3), Autosomal dominant
CLDN14	605608	Deafness, autosomal recessive 29, 614035 (3), Autosomal recessive
CLDN16	603959	Hypomagnesemia 3, renal, 248250 (3), Autosomal recessive
CLDN19	610036	Hypomagnesemia 5, renal, with ocular involvement, 248190 (3), Autosomal recessive
CLDN2	300520	?Azoospermia, obstructive, with nephrolithiasis, 301060 (3), X-linked recessive
CLDN9	615799	?Deafness, autosomal recessive 116, 619093 (3), Autosomal recessive

CLEC1A	606782	{Aspergillosis, susceptibility to}, 614079 (3)
CLEC3B	187520	Macular dystrophy, retinal, 4, 619977 (3), Autosomal dominant
CLEC7A	606264	Candidiasis, familial, 4, autosomal recessive, 613108 (3), Autosomal recessive; {Aspergillosis, susceptibility to}, 614079 (3)
CLIC2	300138	?Intellectual developmental disorder, X-linked syndromic 32, 300886 (3), X-linked recessive
CLIC5	607293	?Deafness, autosomal recessive 103, 616042 (3), Autosomal recessive
CLMP	611693	Congenital short bowel syndrome, 615237 (3), Autosomal recessive
CLN3	607042	Ceroid lipofuscinosis, neuronal, 3, 204200 (3), Autosomal recessive
CLN5	608102	Ceroid lipofuscinosis, neuronal, 5, 256731 (3), Autosomal recessive
CLN6	606725	Ceroid lipofuscinosis, neuronal, 6B (Kufs type), 204300 (3), Autosomal recessive; Ceroid lipofuscinosis, neuronal, 6A, 601780 (3), Autosomal recessive
CLN8	607837	Ceroid lipofuscinosis, neuronal, 8, Northern epilepsy variant, 610003 (3), Autosomal recessive; Ceroid lipofuscinosis, neuronal, 8, 600143 (3), Autosomal recessive
CLP1	608757	Pontocerebellar hypoplasia, type 10, 615803 (3), Autosomal recessive
CLPB	616254	Neutropenia, severe congenital, 9, autosomal dominant, 619813 (3), Autosomal dominant; 3-methylglutaconic aciduria, type VIIB, autosomal recessive, 616271 (3), Autosomal recessive; 3-methylglutaconic aciduria, type VIIA, autosomal dominant, 619835 (3), Autosomal dominant
CLPP	601119	Perrault syndrome 3, 614129 (3), Autosomal recessive
CLPX	615611	?Protoporphyria, erythropoietic, 2, 618015 (3), Autosomal dominant
CLRN1	606397	Usher syndrome, type 3A, 276902 (3), Autosomal recessive; Retinitis pigmentosa 61, 614180 (3)
CLRN2	618988	?Deafness, autosomal recessive 117, 619174 (3), Autosomal recessive
CLTC	118955	Intellectual developmental disorder, autosomal dominant 56, 617854 (3), Autosomal dominant
CNBP	116955	Myotonic dystrophy 2, 602668 (3), Autosomal dominant
CNGA1	123825	Retinitis pigmentosa 49, 613756 (3)
CNGA3	600053	Achromatopsia 2, 216900 (3), Autosomal recessive
CNGB1	600724	Retinitis pigmentosa 45, 613767 (3), Autosomal recessive
CNGB3	605080	Achromatopsia 3, 262300 (3), Autosomal recessive
CNKSR2	300724	Intellectual developmental disorder, X-linked syndromic, Houge type, 301008 (3), X-linked
CNNM2	607803	Hypomagnesemia 6, renal, 613882 (3), Autosomal dominant; Hypomagnesemia, seizures, and mental retardation, 616418 (3), Autosomal dominant, Autosomal recessive
CNNM4	607805	Jalili syndrome, 217080 (3), Autosomal recessive
CNOT1	604917	Vissers-Bodmer syndrome, 619033 (3), Autosomal dominant; Holoprosencephaly 12, with or without pancreatic agenesis, 618500 (3), Autosomal dominant
CNOT2	604909	Intellectual developmental disorder with nasal speech, dysmorphic facies, and variable skeletal anomalies, 618608 (3), Autosomal dominant

CNOT3	604910	Intellectual developmental disorder with speech delay, autism, and dysmorphic facies, 618672 (3), Autosomal dominant
CNP	123830	?Leukodystrophy, hypomyelinating, 20, 619071 (3), Autosomal recessive
CNPY3	610774	Developmental and epileptic encephalopathy 60, 617929 (3), Autosomal recessive
CNTN1	600016	?Myopathy, congenital, Compton-North, 612540 (3), Autosomal recessive
CNTN2	190197	?Epilepsy, myoclonic, familial adult, 5, 615400 (3), Autosomal recessive
CNTNAP1	602346	Lethal congenital contracture syndrome 7, 616286 (3), Autosomal recessive; Hypomyelinating neuropathy, congenital, 3, 618186 (3), Autosomal recessive
CNTNAP2	604569	Pitt-Hopkins like syndrome 1, 610042 (3), Autosomal recessive; {Autism susceptibility 15}, 612100 (3)
COA3	614775	?Mitochondrial complex IV deficiency, nuclear type 14, 619058 (3), Autosomal recessive
COA5	613920	?Mitochondrial complex IV, deficiency, nuclear type 9, 616500 (3), Autosomal recessive
COA6	614772	Mitochondrial complex IV deficiency, nuclear type 13, 616501 (3), Autosomal recessive
COA7	615623	Spinocerebellar ataxia, autosomal recessive, with axonal neuropathy 3, 618387 (3), Autosomal recessive
COA8	616003	Mitochondrial complex IV deficiency, nuclear type 17, 619061 (3), Autosomal recessive
COASY	609855	Pontocerebellar hypoplasia, type 12, 618266 (3), Autosomal recessive; Neurodegeneration with brain iron accumulation 6, 615643 (3), Autosomal recessive
COCH	603196	Deafness, autosomal dominant 9, 601369 (3), Autosomal dominant; ?Deafness, autosomal recessive 110, 618094 (3), Autosomal recessive
COG1	606973	Congenital disorder of glycosylation, type IIg, 611209 (3), Autosomal recessive
COG2	606974	?Congenital disorder of glycosylation, type IIq, 617395 (3), Autosomal recessive
COG4	606976	Congenital disorder of glycosylation, type IIj, 613489 (3), Autosomal recessive; Saul-Wilson syndrome, 618150 (3), Autosomal dominant
COG5	606821	Congenital disorder of glycosylation, type IIi, 613612 (3), Autosomal recessive
COG6	606977	Shaheen syndrome, 615328 (3), Autosomal recessive; Congenital disorder of glycosylation, type III, 614576 (3), Autosomal recessive
COG7	606978	Congenital disorder of glycosylation, type IIe, 608779 (3), Autosomal recessive
COG8	606979	Congenital disorder of glycosylation, type IIh, 611182 (3)
COL10A1	120110	Metaphyseal chondrodysplasia, Schmid type, 156500 (3), Autosomal dominant

COL11A1	120280	Fibrochondrogenesis 1, 228520 (3), Autosomal recessive; Stickler syndrome, type II, 604841 (3), Autosomal dominant; Marshall syndrome, 154780 (3), Autosomal dominant; Deafness, autosomal dominant 37, 618533 (3), Autosomal dominant; {Lumbar disc herniation, susceptibility to}, 603932 (3)
COL11A2	120290	Deafness, autosomal dominant 13, 601868 (3), Autosomal dominant; Otospondylomegaepiphyseal dysplasia, autosomal recessive, 215150 (3), Autosomal recessive; Fibrochondrogenesis 2, 614524 (3), Autosomal dominant, Autosomal recessive; Deafness, autosomal recessive 53, 609706 (3), Autosomal recessive; Otospondylomegaepiphyseal dysplasia, autosomal dominant, 184840 (3), Autosomal dominant
COL12A1	120320	Bethlem myopathy 2, 616471 (3), Autosomal dominant; ?Ullrich congenital muscular dystrophy 2, 616470 (3), Autosomal recessive
COL13A1	120350	Myasthenic syndrome, congenital, 19, 616720 (3), Autosomal recessive
COL17A1	113811	Epithelial recurrent erosion dystrophy, 122400 (3), Autosomal dominant; Epidermolysis bullosa, junctional 4, intermediate, 619787 (3), Autosomal recessive
COL18A1	120328	Knobloch syndrome, type 1, 267750 (3), Autosomal recessive; Glaucoma, primary closed-angle, 618880 (3), Autosomal dominant
COL1A1	120150	Osteogenesis imperfecta, type II, 166210 (3), Autosomal dominant; Caffey disease, 114000 (3), Autosomal dominant; Ehlers-Danlos syndrome, arthrochalasia type, 1, 130060 (3), Autosomal dominant; Osteogenesis imperfecta, type I, 166200 (3), Autosomal dominant; {Bone mineral density variation QTL, osteoporosis}, 166710 (3), Autosomal dominant; Combined osteogenesis imperfecta and Ehlers-Danlos syndrome 1, 619115 (3), Autosomal dominant; Osteogenesis imperfecta, type IV, 166220 (3), Autosomal dominant; Osteogenesis imperfecta, type III, 259420 (3), Autosomal dominant
COL1A2	120160	Osteogenesis imperfecta, type III, 259420 (3), Autosomal dominant; {Osteoporosis, postmenopausal}, 166710 (3), Autosomal dominant; Ehlers-Danlos syndrome, arthrochalasia type, 2, 617821 (3), Autosomal dominant; Combined osteogenesis imperfecta and Ehlers-Danlos syndrome 2, 619120 (3), Autosomal dominant; Ehlers-Danlos syndrome, cardiac valvular type, 225320 (3), Autosomal recessive; Osteogenesis imperfecta, type IV, 166220 (3), Autosomal dominant; Osteogenesis imperfecta, type II, 166210 (3), Autosomal dominant
COL25A1	610004	Fibrosis of extraocular muscles, congenital, 5, 616219 (3), Autosomal recessive
COL27A1	608461	Steel syndrome, 615155 (3), Autosomal recessive

COL2A1	120140	?Vitreoretinopathy with phalangeal epiphyseal dysplasia, 619248 (3), Autosomal dominant; Czech dysplasia, 609162 (3), Autosomal dominant; Achondrogenesis, type II or hypochondrogenesis, 200610 (3), Autosomal dominant; Spondyloperipheral dysplasia, 271700 (3), Autosomal dominant; SMED Strudwick type, 184250 (3), Autosomal dominant; ?Epiphyseal dysplasia, multiple, with myopia and deafness, 132450 (3), Autosomal dominant; SED congenita, 183900 (3), Autosomal dominant; Kniest dysplasia, 156550 (3), Autosomal dominant; Stickler syndrome, type I, nonsyndromic ocular, 609508 (3), Autosomal dominant; Osteoarthritis with mild chondrodysplasia, 604864 (3), Autosomal dominant; Stickler syndrome, type I, 108300 (3), Autosomal dominant; Platyspondylic skeletal dysplasia, Torrance type, 151210 (3), Autosomal dominant; Spondyloepiphyseal dysplasia, Stanescu type, 616583 (3), Autosomal dominant; Avascular necrosis of the femoral head, 608805 (3), Autosomal dominant; Legg-Calve-Perthes disease, 150600 (3), Autosomal dominant
COL3A1	120180	Ehlers-Danlos syndrome, vascular type, 130050 (3), Autosomal dominant; Polymicrogyria with or without vascular-type EDS, 618343 (3), Autosomal recessive
COL4A1	120130	?Retinal arteries, tortuosity of, 180000 (3), Autosomal dominant; {Hemorrhage, intracerebral, susceptibility to}, 614519 (3); Angiopathy, hereditary, with nephropathy, aneurysms, and muscle cramps, 611773 (3), Autosomal dominant; Microangiopathy and leukoencephalopathy, pontine, autosomal dominant, 618564 (3), Autosomal dominant; Brain small vessel disease with or without ocular anomalies, 175780 (3), Autosomal dominant
COL4A2	120090	Brain small vessel disease 2, 614483 (3), Autosomal dominant; {Hemorrhage, intracerebral, susceptibility to}, 614519 (3)
COL4A3	120070	Hematuria, benign familial, 141200 (3), Autosomal dominant; Alport syndrome 3, autosomal dominant, 104200 (3), Autosomal dominant; Alport syndrome 2, autosomal recessive, 203780 (3), Autosomal recessive
COL4A4	120131	Hematuria, familial benign, 141200 (3), Autosomal dominant; Alport syndrome 2, autosomal recessive, 203780 (3), Autosomal recessive
COL4A5	303630	Alport syndrome 1, X-linked, 301050 (3), X-linked dominant
COL4A6	303631	?Deafness, X-linked 6, 300914 (3), X-linked recessive
COL5A1	120215	Ehlers-Danlos syndrome, classic type, 1, 130000 (3), Autosomal dominant; Fibromuscular dysplasia, multifocal, 619329 (3), Autosomal dominant
COL5A2	120190	Ehlers-Danlos syndrome, classic type, 2, 130010 (3), Autosomal dominant
COL6A1	120220	Bethlem myopathy 1, 158810 (3), Autosomal dominant, Autosomal recessive; Ullrich congenital muscular dystrophy 1, 254090 (3), Autosomal dominant, Autosomal recessive
COL6A2	120240	Bethlem myopathy 1, 158810 (3), Autosomal dominant, Autosomal recessive; ?Myosclerosis, congenital, 255600 (3), Autosomal recessive; Ullrich congenital muscular dystrophy 1, 254090 (3), Autosomal dominant, Autosomal recessive

COL6A3	120250	Ullrich congenital muscular dystrophy 1, 254090 (3), Autosomal dominant, Autosomal recessive; Dystonia 27, 616411 (3), Autosomal recessive; Bethlem myopathy 1, 158810 (3), Autosomal dominant, Autosomal recessive
COL7A1	120120	EBD, localisata variant (3); Epidermolysis bullosa, pretibial, 131850 (3), Autosomal dominant, Autosomal recessive; Transient bullous of the newborn, 131705 (3), Autosomal dominant, Autosomal recessive; EBD, Bart type, 132000 (3), Autosomal dominant; Epidermolysis bullosa dystrophica, AD, 131750 (3), Autosomal dominant; Epidermolysis bullosa pruriginosa, 604129 (3), Autosomal dominant, Autosomal recessive; EBD inversa, 226600 (3), Autosomal recessive; Epidermolysis bullosa dystrophica, AR, 226600 (3), Autosomal recessive; Toenail dystrophy, isolated, 607523 (3), Autosomal dominant
COL8A2	120252	Corneal dystrophy, posterior polymorphous 2, 609140 (3), Autosomal dominant; Corneal dystrophy, Fuchs endothelial, 1, 136800 (3), Autosomal dominant
COL9A1	120210	Stickler syndrome, type IV, 614134 (3); ?Epiphyseal dysplasia, multiple, 6, 614135 (3), Autosomal dominant
COL9A2	120260	Epiphyseal dysplasia, multiple, 2, 600204 (3), Autosomal dominant; ?Stickler syndrome, type V, 614284 (3), Autosomal recessive
COL9A3	120270	{Intervertebral disc disease, susceptibility to}, 603932 (3); Epiphyseal dysplasia, multiple, 3, with or without myopathy, 600969 (3), Autosomal dominant
COLEC10	607620	3MC syndrome 3, 248340 (3), Autosomal recessive
COLEC11	612502	3MC syndrome 2, 265050 (3), Autosomal recessive
COLGALT1	617531	Brain small vessel disease 3, 618360 (3), Autosomal recessive
COLQ	603033	Myasthenic syndrome, congenital, 5, 603034 (3), Autosomal recessive
COMP	600310	Pseudoachondroplasia, 177170 (3), Autosomal dominant; Carpal tunnel syndrome 2, 619161 (3), Autosomal dominant; Epiphyseal dysplasia, multiple, 1, 132400 (3), Autosomal dominant
COMT	116790	{Schizophrenia, susceptibility to}, 181500 (3), Autosomal dominant; {Panic disorder, susceptibility to}, 167870 (3), ?Autosomal dominant
COPA	601924	{Autoimmune interstitial lung, joint, and kidney disease}, 616414 (3), Autosomal dominant
COPB1	600959	Baralle-Macken syndrome, 619255 (3), Autosomal recessive
COPB2	606990	Osteoporosis, childhood- or juvenile-onset, with developmental delay, 619884 (3), Autosomal dominant; ?Microcephaly 19, primary, autosomal recessive, 617800 (3), Autosomal recessive
COQ2	609825	{Multiple system atrophy, susceptibility to}, 146500 (3), Autosomal dominant, Autosomal recessive; Coenzyme Q10 deficiency, primary, 1, 607426 (3), Autosomal recessive
COQ4	612898	Coenzyme Q10 deficiency, primary, 7, 616276 (3), Autosomal recessive
COQ5	616359	?Coenzyme Q10 deficiency, primary, 9, 619028 (3), Autosomal recessive
COQ6	614647	Coenzyme Q10 deficiency, primary, 6, 614650 (3), Autosomal recessive

COQ7	601683	?Coenzyme Q10 deficiency, primary, 8, 616733 (3), Autosomal recessive
COQ8A	606980	Coenzyme Q10 deficiency, primary, 4, 612016 (3), Autosomal recessive
COQ8B	615567	Nephrotic syndrome, type 9, 615573 (3), Autosomal recessive
COQ9	612837	Coenzyme Q10 deficiency, primary, 5, 614654 (3), Autosomal recessive
CORIN	605236	Preeclampsia/eclampsia 5, 614595 (3)
CORO1A	605000	Immunodeficiency 8, 615401 (3), Autosomal recessive
COX10	602125	Mitochondrial complex IV deficiency, nuclear type 3, 619046 (3), Autosomal recessive
COX14	614478	?Mitochondrial complex IV deficiency, nuclear type 10, 619053 (3), Autosomal recessive
COX15	603646	Mitochondrial complex IV deficiency, nuclear type 6, 615119 (3), Autosomal recessive
COX16	618064	Mitochondrial complex IV deficiency, nuclear type 22, 619355 (3), Autosomal recessive
COX20	614698	Mitochondrial complex IV deficiency, nuclear type 11, 619054 (3), Autosomal recessive
COX4I1	123864	Mitochondrial complex IV deficiency, nuclear type 16, 619060 (3), Autosomal recessive
COX4I2	607976	Exocrine pancreatic insufficiency, dyserythropoietic anemia, and calvarial hyperostosis, 612714 (3), Autosomal recessive
COX5A	603773	?Mitochondrial complex IV deficiency, nuclear type 20, 619064 (3), Autosomal recessive
COX6A1	602072	Charcot-Marie-Tooth disease, recessive intermediate D, 616039 (3), Autosomal recessive
COX6A2	602009	Mitochondrial complex IV deficiency, nuclear type 18, 619062 (3), Autosomal recessive
COX6B1	124089	Mitochondrial complex IV deficiency, nuclear type 7, 619051 (3), Autosomal recessive
COX7B	300885	Linear skin defects with multiple congenital anomalies 2, 300887 (3), X-linked dominant
COX8A	123870	?Mitochondrial complex IV deficiency, nuclear type 15, 619059 (3), Autosomal recessive
CP	117700	Cerebellar ataxia, 604290 (3), Autosomal recessive; [Hypoceruloplasminemia, hereditary], 604290 (3), Autosomal recessive; Hemosiderosis, systemic, due to aceruloplasminemia, 604290 (3), Autosomal recessive
CPA6	609562	Febrile seizures, familial, 11, 614418 (3), Autosomal recessive; Epilepsy, familial temporal lobe, 5, 614417 (3), Autosomal dominant, Autosomal recessive
CPAMD8	608841	Anterior segment dysgenesis 8, 617319 (3), Autosomal recessive
CPE	114855	BDV syndrome, 619326 (3), Autosomal recessive
CPLANE1	614571	Orofaciodigital syndrome VI, 277170 (3), Autosomal recessive; Joubert syndrome 17, 614615 (3), Autosomal recessive

CPLX1	605032	Developmental and epileptic encephalopathy 63, 617976 (3), Autosomal recessive
CPN1	603103	Carboxypeptidase N deficiency, 212070 (3), Autosomal recessive
CPOX	612732	Coproporphyria, 121300 (3), Autosomal dominant, Autosomal recessive; Harderoporphyria, 618892 (3), Autosomal recessive
CPS1	608307	Carbamoylphosphate synthetase I deficiency, 237300 (3), Autosomal recessive; {Pulmonary hypertension, neonatal, susceptibility to}, 615371 (3)
CPSF1	606027	Myopia 27, 618827 (3), Autosomal dominant
CPSF3	606029	Neurodevelopmental disorder with microcephaly, hypotonia, nystagmus, and seizures, 619876 (3), Autosomal recessive
CPT1A	600528	CPT deficiency, hepatic, type IA, 255120 (3), Autosomal recessive
CPT1C	608846	?Spastic paraplegia 73, autosomal dominant, 616282 (3), Autosomal dominant
CPT2	600650	{Encephalopathy, acute, infection-induced, 4, susceptibility to}, 614212 (3), Autosomal dominant, Autosomal recessive; CPT II deficiency, infantile, 600649 (3), Autosomal recessive; CPT II deficiency, lethal neonatal, 608836 (3), Autosomal recessive; CPT II deficiency, myopathic, stress-induced, 255110 (3), Autosomal dominant, Autosomal recessive
CR1	120620	[Blood group, Knops system], 607486 (3); {Malaria, severe, resistance to}, 611162 (3)
CR2	120650	{Systemic lupus erythematosus, susceptibility to, 9}, 610927 (3); Immunodeficiency, common variable, 7, 614699 (3), Autosomal recessive
CRADD	603454	Intellectual developmental disorder, autosomal recessive 34, with variant lissencephaly, 614499 (3), Autosomal recessive
CRAT	600184	?Neurodegeneration with brain iron accumulation 8, 617917 (3), Autosomal recessive
CRB1	604210	Leber congenital amaurosis 8, 613835 (3), Autosomal recessive; Retinitis pigmentosa-12, 600105 (3), Autosomal recessive; Pigmented paravenous chorioretinal atrophy, 172870 (3), Autosomal dominant
CRB2	609720	Focal segmental glomerulosclerosis 9, 616220 (3), Autosomal recessive; Ventriculomegaly with cystic kidney disease, 219730 (3), Autosomal recessive
CRBN	609262	Intellectual developmental disorder, autosomal recessive 2, 607417 (3), Autosomal recessive
CREB1	123810	Histiocytoma, angiomatoid fibrous, somatic, 612160 (3)
CREB3L1	616215	Osteogenesis imperfecta, type XVI, 616229 (3), Autosomal recessive
CREB3L3	611998	Hypertriglyceridemia 2, 619324 (3), Autosomal dominant
CREBBP	600140	Menke-Hennekam syndrome 1, 618332 (3), Autosomal dominant; Rubinstein-Taybi syndrome 1, 180849 (3), Autosomal dominant
CRELD1	607170	Atrioventricular septal defect, partial, with heterotaxy syndrome, 606217 (3), Autosomal dominant; {Atrioventricular septal defect, susceptibility to, 2}, 606217 (3), Autosomal dominant
CRIPT	604594	Short stature with microcephaly and distinctive facies, 615789 (3), Autosomal recessive

CRLF1	604237	Cold-induced sweating syndrome 1, 272430 (3), Autosomal recessive
CRPPA	614631	Muscular dystrophy-dystroglycanopathy (limb-girdle), type C, 7, 616052 (3), Autosomal recessive; Muscular dystrophy-dystroglycanopathy (congenital with brain and eye anomalies), type A, 7, 614643 (3), Autosomal recessive
CRTAP	605497	Osteogenesis imperfecta, type VII, 610682 (3), Autosomal recessive
CRTC1	607536	Mucoepidermoid salivary gland carcinoma (3)
CRX	602225	Leber congenital amaurosis 7, 613829 (3); Cone-rod retinal dystrophy-2, 120970 (3), Autosomal dominant
CRY1	601933	{Delayed sleep phase disorder, susceptibility to}, 614163 (3), Autosomal dominant
CRYAA	123580	Cataract 9, multiple types, 604219 (3), Autosomal dominant, Autosomal recessive
CRYAB	123590	Myopathy, myofibrillar, fatal infantile hypertonic, alpha-B crystallin-related, 613869 (3), Autosomal recessive; Myopathy, myofibrillar, 2, 608810 (3), Autosomal dominant; Cataract 16, multiple types, 613763 (3), Autosomal dominant, Autosomal recessive; Cardiomyopathy, dilated, 1II, 615184 (3), Autosomal dominant
CRYBA1	123610	Cataract 10, multiple types, 600881 (3), Autosomal dominant
CRYBA2	600836	?Cataract 42, 115900 (3), Autosomal dominant
CRYBA4	123631	Cataract 23, 610425 (3)
CRYBB1	600929	Cataract 17, multiple types, 611544 (3), Autosomal dominant, Autosomal recessive
CRYBB2	123620	Cataract 3, multiple types, 601547 (3), Autosomal dominant
CRYBB3	123630	Cataract 22, 609741 (3), Autosomal dominant, Autosomal recessive
CRYGB	123670	Cataract 39, multiple types, autosomal dominant, 615188 (3), Autosomal dominant
CRYGC	123680	Cataract 2, multiple types, 604307 (3), Autosomal dominant
CRYGD	123690	Cataract 4, multiple types, 115700 (3), Autosomal dominant
CRYGS	123730	Cataract 20, multiple types, 116100 (3), Autosomal dominant
CRYM	123740	Deafness, autosomal dominant 40, 616357 (3), Autosomal dominant
CSF1R	164770	Brain abnormalities, neurodegeneration, and dysosteosclerosis, 618476 (3), Autosomal recessive; Leukoencephalopathy, diffuse hereditary, with spheroids 1, 221820 (3), Autosomal dominant
CSF2RA	306250	Surfactant metabolism dysfunction, pulmonary, 4, 300770 (3)
CSF2RB	138981	Surfactant metabolism dysfunction, pulmonary, 5, 614370 (3), Autosomal recessive
CSF3R	138971	Neutropenia, severe congenital, 7, autosomal recessive, 617014 (3), Autosomal recessive; ?Neutrophilia, hereditary, 162830 (3), Autosomal dominant
CSGALNACT1	616615	Skeletal dysplasia, mild, with joint laxity and advanced bone age, 618870 (3), Autosomal recessive
CSH1	150200	[Placental lactogen deficiency] (1)

CSNK1D	600864	Advanced sleep-phase syndrome, familial, 2, 615224 (3), Autosomal dominant
CSNK2A1	115440	Okur-Chung neurodevelopmental syndrome, 617062 (3), Autosomal dominant
CSNK2B	115441	Poirier-Bienvenu neurodevelopmental syndrome, 618732 (3), Autosomal dominant
CSPP1	611654	Joubert syndrome 21, 615636 (3), Autosomal recessive
CSRP3	600824	?Cardiomyopathy, dilated, 1M, 607482 (3); Cardiomyopathy, hypertrophic, 12, 612124 (3), Autosomal dominant
CST3	604312	{Macular degeneration, age-related, 11}, 611953 (3); Cerebral amyloid angiopathy, 105150 (3), Autosomal dominant
CST6	601891	?Ectodermal dysplasia 15, hypohidrotic/hair type, 618535 (3), Autosomal recessive
CSTA	184600	Peeling skin syndrome 4, 607936 (3), Autosomal recessive
CSTB	601145	Epilepsy, progressive myoclonic 1A (Unverricht and Lundborg), 254800 (3), Autosomal recessive
CTBP1	602618	Hypotonia, ataxia, developmental delay, and tooth enamel defect syndrome, 617915 (3), Autosomal dominant
CTC1	613129	Cerebroretinal microangiopathy with calcifications and cysts, 612199 (3), Autosomal recessive
CTCF	604167	Intellectual developmental disorder, autosomal dominant 21, 615502 (3), Autosomal dominant
CTDP1	604927	Congenital cataracts, facial dysmorphism, and neuropathy, 604168 (3), Autosomal recessive
CTH	607657	Cystathioninuria, 219500 (3), Autosomal recessive
CTHRC1	610635	Barrett esophagus/esophageal adenocarcinoma, 614266 (3)
CTLA4	123890	Immune dysregulation with autoimmunity, immunodeficiency, and lymphoproliferation, 616100 (3), Autosomal dominant; {Diabetes mellitus, insulin-dependent, 12}, 601388 (3); {Celiac disease, susceptibility to, 3}, 609755 (3); {Hashimoto thyroiditis}, 140300 (3), Autosomal dominant; {Systemic lupus erythematosus, susceptibility to}, 152700 (3), Autosomal dominant
CTNNA1	116805	Macular dystrophy, patterned, 2, 608970 (3), Autosomal dominant
CTNNA2	114025	Cortical dysplasia, complex, with other brain malformations 9, 618174 (3), Autosomal recessive
CTNNA3	607667	Arrhythmogenic right ventricular dysplasia, familial, 13, 615616 (3), Autosomal dominant
CTNNB1	116806	Exudative vitreoretinopathy 7, 617572 (3), Autosomal dominant; Pilomatricoma, somatic, 132600 (3); Colorectal cancer, somatic, 114500 (3); Neurodevelopmental disorder with spastic diplegia and visual defects, 615075 (3), Autosomal dominant; Medulloblastoma, somatic, 155255 (3); Ovarian cancer, somatic, 167000 (3); Hepatocellular carcinoma, somatic, 114550 (3)

CTNNBL1	611537	?Immunodeficiency 99 with hypogammaglobulinemia and autoimmune cytopenias, 619846 (3), Autosomal recessive
CTNND1	601045	Blepharocheilodontic syndrome 2, 617681 (3), Autosomal dominant
CTNS	606272	Cystinosis, nephropathic, 219800 (3), Autosomal recessive; Cystinosis, ocular nonnephropathic, 219750 (3), Autosomal recessive; Cystinosis, late-onset juvenile or adolescent nephropathic, 219900 (3), Autosomal recessive; Cystinosis, atypical nephropathic, 219800 (3), Autosomal recessive
CTPS1	123860	Immunodeficiency 24, 615897 (3), Autosomal recessive
CTRC	601405	{Pancreatitis, chronic, susceptibility to}, 167800 (3), Autosomal dominant
CTSA	613111	Galactosialidosis, 256540 (3), Autosomal recessive
CTSB	116810	Keratolytic winter erythema, 148370 (4), Autosomal dominant
CTSC	602365	Periodontitis 1, juvenile, 170650 (3), Autosomal recessive; Haim-Munk syndrome, 245010 (3), Autosomal recessive; Papillon-Lefevre syndrome, 245000 (3), Autosomal recessive
CTSD	116840	Ceroid lipofuscinosis, neuronal, 10, 610127 (3), Autosomal recessive
CTSF	603539	Ceroid lipofuscinosis, neuronal, 13 (Kufs type), 615362 (3), Autosomal recessive
CTSK	601105	Pycnodysostosis, 265800 (3), Autosomal recessive
CTU2	617057	Microcephaly, facial dysmorphism, renal agenesis, and ambiguous genitalia syndrome, 618142 (3), Autosomal recessive
CUBN	602997	[Proteinuria, chronic benign], 618884 (3), Autosomal recessive; Imerslund-Grasbeck syndrome 1, 261100 (3), Autosomal recessive
CUL3	603136	Neurodevelopmental disorder with or without autism or seizures, 619239 (3), Autosomal dominant; Pseudohypoaldosteronism, type IIE, 614496 (3), Autosomal dominant
CUL4B	300304	Intellectual developmental disorder, X-linked syndromic, Cabezas type, 300354 (3), X-linked recessive
CUL7	609577	3-M syndrome 1, 273750 (3), Autosomal recessive
CUX1	116896	Global developmental delay with or without impaired intellectual development, 618330 (3), Autosomal dominant
CUX2	610648	Developmental and epileptic encephalopathy 67, 618141 (3), Autosomal dominant
CWC27	617170	Retinitis pigmentosa with or without skeletal anomalies, 250410 (3), Autosomal recessive
CWF19L1	616120	Spinocerebellar ataxia, autosomal recessive 17, 616127 (3), Autosomal recessive
CX3CR1	601470	{Rapid progression to AIDS from HIV1 infection}, 609423 (3); {Macular degeneration, age-related, 12}, 613784 (3); {Coronary artery disease, resistance to}, 607339 (3)
CXCL12	600835	{AIDS, resistance to}, 609423 (3)
CXCR1	146929	{AIDS, slow progression to}, 609423 (3)
CXCR2	146928	?WHIM syndrome 2, 619407 (3), Autosomal recessive
CXCR4	162643	WHIM syndrome 1, 193670 (3), Autosomal dominant; Myelokathexis, isolated, 193670 (3), Autosomal dominant

CYB561	600019	Orthostatic hypotension 2, 618182 (3), Autosomal recessive
CYB5A	613218	Methemoglobinemia and ambiguous genitalia, 250790 (3), Autosomal recessive
CYB5R3	613213	Methemoglobinemia, type I, 250800 (3), Autosomal recessive; Methemoglobinemia, type II, 250800 (3), Autosomal recessive
CYBA	608508	Chronic granulomatous disease 4, autosomal recessive, 233690 (3), Autosomal recessive
CYBB	300481	Immunodeficiency 34, mycobacteriosis, X-linked, 300645 (3), X-linked recessive; Chronic granulomatous disease, X-linked, 306400 (3), X-linked recessive
CYBC1	618334	Chronic granulomatous disease 5, autosomal recessive, 618935 (3), Autosomal recessive
CYC1	123980	Mitochondrial complex III deficiency, nuclear type 6, 615453 (3), Autosomal recessive
CYCS	123970	Thrombocytopenia 4, 612004 (3), Autosomal dominant
CYFIP2	606323	Developmental and epileptic encephalopathy 65, 618008 (3), Autosomal dominant
CYLD	605018	Brooke-Spiegler syndrome, 605041 (3), Autosomal dominant; Cylindromatosis, familial, 132700 (3), Autosomal dominant; Trichoepithelioma, multiple familial, 1, 601606 (3), Autosomal dominant; ?Frontotemporal dementia and/or amyotrophic lateral sclerosis 8, 619132 (3), Autosomal dominant
CYP11A1	118485	Adrenal insufficiency, congenital, with 46XY sex reversal, partial or complete, 613743 (3)
CYP11B1	610613	Aldosteronism, glucocorticoid-remediable, 103900 (3), Autosomal dominant; Adrenal hyperplasia, congenital, due to 11-beta-hydroxylase deficiency, 202010 (3), Autosomal recessive
CYP11B2	124080	Hypoaldosteronism, congenital, due to CMO I deficiency, 203400 (3), Autosomal recessive; Aldosterone to renin ratio raised (3); {Low renin hypertension, susceptibility to} (3); Hypoaldosteronism, congenital, due to CMO II deficiency, 610600 (3), Autosomal recessive
CYP17A1	609300	17,20-lyase deficiency, isolated, 202110 (3), Autosomal recessive; 17-alpha-hydroxylase/17,20-lyase deficiency, 202110 (3), Autosomal recessive
CYP19A1	107910	Aromatase deficiency, 613546 (3); Aromatase excess syndrome, 139300 (3), Autosomal dominant
CYP1B1	601771	Glaucoma 3A, primary open angle, congenital, juvenile, or adult onset, 231300 (3), Autosomal recessive; Anterior segment dysgenesis 6, multiple subtypes, 617315 (3), Autosomal recessive
CYP21A2	613815	Hyperandrogenism, nonclassic type, due to 21-hydroxylase deficiency, 201910 (3), Autosomal recessive; Adrenal hyperplasia, congenital, due to 21-hydroxylase deficiency, 201910 (3), Autosomal recessive
CYP24A1	126065	Hypercalcemia, infantile, 1, 143880 (3), Autosomal recessive
CYP26B1	605207	Craniosynostosis with radiohumeral fusions and other skeletal and craniofacial anomalies, 614416 (3)

CYP26C1	608428	Focal facial dermal dysplasia 4, 614974 (3), Autosomal recessive
CYP27A1	606530	Cerebrotendinous xanthomatosis, 213700 (3), Autosomal recessive
CYP27B1	609506	Vitamin D-dependent rickets, type I, 264700 (3), Autosomal recessive
CYP2A6	122720	{Lung cancer, resistance to}, 211980 (3), Autosomal dominant, Somatic mutation; Coumarin resistance, 122700 (3), Autosomal dominant; {Nicotine addiction, protection from}, 188890 (3)
CYP2B6	123930	{Efavirenz central nervous system toxicity, susceptibility to}, 614546 (3); Efavirenz, poor metabolism of, 614546 (3)
CYP2C19	124020	Proguanil poor metabolizer, 609535 (3), Autosomal recessive; Mephenytoin poor metabolizer, 609535 (3), Autosomal recessive; Clopidogrel, impaired responsiveness to, 609535 (3), Autosomal recessive; Omeprazole poor metabolizer, 609535 (3), Autosomal recessive
CYP2C8	601129	{Drug metabolism, altered, CYP2C8-related}, 618018 (3)
CYP2C9	601130	Warfarin sensitivity, 122700 (3), Autosomal dominant; Tolbutamide poor metabolizer (3)
CYP2D6	124030	{Codeine sensitivity}, 608902 (3), Autosomal recessive; {Debrisoquine sensitivity}, 608902 (3), Autosomal recessive
CYP2R1	608713	Rickets due to defect in vitamin D 25-hydroxylation deficiency, 600081 (3), Autosomal recessive
CYP2U1	610670	Spastic paraplegia 56, autosomal recessive, 615030 (3), Autosomal recessive
CYP3A4	124010	Vitamin D-dependent rickets, type 3, 619073 (3), Autosomal dominant
CYP3A5	605325	{Hypertension, salt-sensitive essential, susceptibility to}, 145500 (3), Multifactorial
CYP4F22	611495	Ichthyosis, congenital, autosomal recessive 5, 604777 (3), Autosomal recessive
CYP4V2	608614	Bietti crystalline corneoretinal dystrophy, 210370 (3), Autosomal recessive
CYP7B1	603711	Spastic paraplegia 5A, autosomal recessive, 270800 (3), Autosomal recessive; Bile acid synthesis defect, congenital, 3, 613812 (3), Autosomal recessive
D2HGDH	609186	D-2-hydroxyglutaric aciduria, 600721 (3), Autosomal recessive
DAAM2	606627	Nephrotic syndrome, type 24, 619263 (3), Autosomal recessive
DAB1	603448	Spinocerebellar ataxia 37, 615945 (3), Autosomal dominant
DACT1	607861	?Townes-Brocks syndrome 2, 617466 (3), Autosomal dominant
DAG1	128239	Muscular dystrophy-dystroglycanopathy (congenital with brain and eye anomalies), type A, 9, 616538 (3), Autosomal recessive; Muscular dystrophy-dystroglycanopathy (limb-girdle), type C, 9, 613818 (3), Autosomal recessive
DALRD3	618904	?Developmental and epileptic encephalopathy 86, 618910 (3), Autosomal recessive
DAOA	607408	{Schizophrenia}, 181500 (2), Autosomal dominant
DARS1	603084	Hypomyelination with brainstem and spinal cord involvement and leg spasticity, 615281 (3), Autosomal recessive
DARS2	610956	Leukoencephalopathy with brain stem and spinal cord involvement and lactate elevation, 611105 (3), Autosomal recessive

DAZL	601486	{Spermatogenic failure, susceptibility to} (3)
DBH	609312	Orthostatic hypotension 1, due to DBH deficiency, 223360 (3), Autosomal recessive
DBR1	607024	{Encephalitis, acute, infection (viral)-induced, susceptibility to, 11}, 619441 (3), Autosomal recessive
DBT	248610	Maple syrup urine disease, type II, 248600 (3), Autosomal recessive
DCAF17	612515	Woodhouse-Sakati syndrome, 241080 (3), Autosomal recessive
DCAF8	615820	?Giant axonal neuropathy 2, autosomal dominant, 610100 (3), Autosomal dominant
DCC	120470	Mirror movements 1 and/or agenesis of the corpus callosum, 157600 (3), Autosomal dominant; Esophageal carcinoma, somatic, 133239 (3); Colorectal cancer, somatic, 114500 (3); Gaze palsy, familial horizontal, with progressive scoliosis, 2, 617542 (3), Autosomal recessive
DCDC2	605755	Nephronophthisis 19, 616217 (3), Autosomal recessive; ?Deafness, autosomal recessive 66, 610212 (3), Autosomal recessive; Sclerosing cholangitis, neonatal, 617394 (3), Autosomal recessive
DCHS1	603057	Mitral valve prolapse 2, 607829 (3), Autosomal dominant; Van Maldergem syndrome 1, 601390 (3), Autosomal recessive
DCLRE1C	605988	Severe combined immunodeficiency, Athabascan type, 602450 (3), Autosomal recessive; Omenn syndrome, 603554 (3), Autosomal recessive
DCN	125255	Corneal dystrophy, congenital stromal, 610048 (3), Autosomal dominant
DCPS	610534	Al-Raqad syndrome, 616459 (3), Autosomal recessive
DCT	191275	Oculocutaneous albinism, type VIII, 619165 (3), Autosomal recessive
DCTN1	601143	Neuronopathy, distal hereditary motor, type VIIB, 607641 (3), Autosomal dominant; Perry syndrome, 168605 (3), Autosomal dominant; {Amyotrophic lateral sclerosis, susceptibility to}, 105400 (3), Autosomal dominant, Autosomal recessive
DCX	300121	Subcortical laminal heterotopia, X-linked, 300067 (3), X-linked; Lissencephaly, X-linked, 300067 (3), X-linked
DCXR	608347	[Pentosuria], 260800 (3), Autosomal recessive
DDB1	600045	White-Kernohan syndrome, 619426 (3), Autosomal dominant
DDB2	600811	Xeroderma pigmentosum, group E, DDB-negative subtype, 278740 (3), Autosomal recessive
DDC	107930	Aromatic L-amino acid decarboxylase deficiency, 608643 (3), Autosomal recessive
DDHD1	614603	Spastic paraplegia 28, autosomal recessive, 609340 (3), Autosomal recessive
DDHD2	615003	Spastic paraplegia 54, autosomal recessive, 615033 (3), Autosomal recessive
DDOST	602202	Congenital disorder of glycosylation, type Ir, 614507 (3), Autosomal recessive
DDR2	191311	Warburg-Cinotti syndrome, 618175 (3), Autosomal dominant; Spondylometaepiphyseal dysplasia, short limb-hand type, 271665 (3), Autosomal recessive

DDRGK1	616177	Spondyloepimetaphyseal dysplasia, Shohat type, 602557 (3), Autosomal recessive
DDX11	601150	Warsaw breakage syndrome, 613398 (3), Autosomal recessive
DDX3X	300160	Intellectual developmental disorder, X-linked syndromic, Snijders Blok type, 300958 (3), X-linked recessive, X-linked dominant
DDX41	608170	{Myeloproliferative/lymphoproliferative neoplasms, familial (multiple types), susceptibility to}, 616871 (3), Autosomal dominant
DDX59	615464	Orofaciodigital syndrome V, 174300 (3), Autosomal recessive
DDX6	600326	Intellectual developmental disorder with impaired language and dysmorphic facies, 618653 (3), Autosomal dominant
DEAF1	602635	Vulto-van Silfout-de Vries syndrome, 615828 (3), Autosomal dominant; Neurodevelopmental disorder with hypotonia, impaired expressive language, and with or without seizures, 617171 (3), Autosomal recessive
DEF6	610094	Immunodeficiency 87 and autoimmunity, 619573 (3), Autosomal recessive
DEGS1	615843	Leukodystrophy, hypomyelinating, 18, 618404 (3), Autosomal recessive
DEK	125264	Leukemia, acute nonlymphocytic, 125264 (2)
DENND5A	617278	Developmental and epileptic encephalopathy 49, 617281 (3), Autosomal recessive
DEPDC5	614191	Epilepsy, familial focal, with variable foci 1, 604364 (3), Autosomal dominant
DES	125660	Scapuloperoneal syndrome, neurogenic, Kaeser type, 181400 (3), Autosomal dominant; Cardiomyopathy, dilated, 1I, 604765 (3), Autosomal dominant; Myopathy, myofibrillar, 1, 601419 (3), Autosomal dominant, Autosomal recessive
DGAT1	604900	?Diarrhea 7, protein-losing enteropathy type, 615863 (3), Autosomal recessive
DGKE	601440	{Hemolytic uremic syndrome, atypical, susceptibility to, 7}, 615008 (3), Autosomal recessive; Nephrotic syndrome, type 7, 615008 (3), Autosomal recessive
DGUOK	601465	Portal hypertension, noncirrhotic, 1, 617068 (3), Autosomal recessive; Progressive external ophthalmoplegia with mitochondrial DNA deletions, autosomal recessive 4, 617070 (3), Autosomal recessive; Mitochondrial DNA depletion syndrome 3 (hepatocerebral type), 251880 (3), Autosomal recessive
DHCR24	606418	Desmosterolosis, 602398 (3), Autosomal recessive
DHCR7	602858	Smith-Lemli-Opitz syndrome, 270400 (3), Autosomal recessive
DHDDS	608172	Developmental delay and seizures with or without movement abnormalities, 617836 (3), Autosomal dominant; ?Congenital disorder of glycosylation, type 1bb, 613861 (3), Autosomal recessive; Retinitis pigmentosa 59, 613861 (3), Autosomal recessive
DHFR	126060	Megaloblastic anemia due to dihydrofolate reductase deficiency, 613839 (3), Autosomal recessive
DHH	605423	46XY gonadal dysgenesis with minifascicular neuropathy, 607080 (3), Autosomal recessive; 46XY sex reversal 7, 233420 (3), Autosomal recessive

DHODH	126064	Miller syndrome, 263750 (3), Autosomal recessive
DHPS	600944	Neurodevelopmental disorder with seizures and speech and walking impairment, 618480 (3), Autosomal recessive
DHTKD1	614984	?Charcot-Marie-Tooth disease, axonal, type 2Q, 615025 (3), Autosomal dominant; Alpha-aminoadipic and alpha-ketoadipic aciduria, 204750 (3), Autosomal recessive
DHX16	603405	Neuromuscular disease and ocular or auditory anomalies with or without seizures, 618733 (3), Autosomal dominant
DHX30	616423	Neurodevelopmental disorder with severe motor impairment and absent language, 617804 (3), Autosomal dominant
DHX37	617362	Neurodevelopmental disorder with brain anomalies and with or without vertebral or cardiac anomalies, 618731 (3), Autosomal recessive; 46, XY sex reversal 11, 273250 (3), Autosomal dominant
DHX38	605584	Retinitis pigmentosa 84, 618220 (3), Autosomal recessive
DIABLO	605219	Deafness, autosomal dominant 64, 614152 (3), Autosomal dominant
DIAPH1	602121	Deafness, autosomal dominant 1, with or without thrombocytopenia, 124900 (3), Autosomal dominant; Seizures, cortical blindness, microcephaly syndrome, 616632 (3), Autosomal recessive
DIAPH2	300108	?Premature ovarian failure 2A, 300511 (3), X-linked dominant
DIAPH3	614567	Auditory neuropathy, autosomal dominant 1, 609129 (3), Autosomal dominant
DICER1	606241	Pleuropulmonary blastoma, 601200 (3), Autosomal dominant; Goiter, multinodular 1, with or without Sertoli-Leydig cell tumors, 138800 (3), Autosomal dominant; GLOW syndrome, somatic mosaic, 618272 (3); Rhabdomyosarcoma, embryonal, 2, 180295 (3)
DIO1	147892	Thyroid hormone metabolism, abnormal, 2, 619855 (3), Autosomal dominant
DIP2B	611379	Intellectual developmental disorder, autosomal dominant, FRA12A type, 136630 (3), Autosomal dominant
DIS3L2	614184	Perlman syndrome, 267000 (3), Autosomal recessive
DISC1	605210	{Schizophrenia 9, susceptibility to}, 604906 (3)
DKC1	300126	Dyskeratosis congenita, X-linked, 305000 (3), X-linked recessive
DLAT	608770	Pyruvate dehydrogenase E2 deficiency, 245348 (3), Autosomal recessive
DLC1	604258	Colorectal cancer, somatic, 114500 (3)
DLD	238331	Dihydrolipoamide dehydrogenase deficiency, 246900 (3), Autosomal recessive
DLG3	300189	Intellectual developmental disorder, X-linked 90, 300850 (3), X-linked recessive
DLG4	602887	Intellectual developmental disorder, autosomal dominant 62, 618793 (3), Autosomal dominant
DLL1	606582	Neurodevelopmental disorder with nonspecific brain abnormalities and with or without seizures, 618709 (3), Autosomal dominant
DLL3	602768	Spondylocostal dysostosis 1, autosomal recessive, 277300 (3), Autosomal recessive

DLL4	605185	Adams-Oliver syndrome 6, 616589 (3), Autosomal dominant
DLST	126063	Paragangliomas 7, 618475 (3), Autosomal dominant
DLX3	600525	Trichodontoosseous syndrome, 190320 (3), Autosomal dominant; Amelogenesis imperfecta, type IV, 104510 (3), Autosomal dominant
DLX4	601911	?Orofacial cleft 15, 616788 (3), Autosomal dominant
DLX5	600028	Split-hand/foot malformation 1, 183600 (3), Autosomal dominant; ?Split-hand/foot malformation 1 with sensorineural hearing loss, 220600 (3), Autosomal recessive
DMD	300377	Becker muscular dystrophy, 300376 (3), X-linked recessive; Cardiomyopathy, dilated, 3B, 302045 (3), X-linked; Duchenne muscular dystrophy, 310200 (3), X-linked recessive
DMGDH	605849	Dimethylglycine dehydrogenase deficiency, 605850 (3), Autosomal recessive
DMP1	600980	Hypophosphatemic rickets, AR, 241520 (3), Autosomal recessive
DMPK	605377	Myotonic dystrophy 1, 160900 (3), Autosomal dominant
DMXL2	612186	Developmental and epileptic encephalopathy 81, 618663 (3), Autosomal recessive; ?Deafness, autosomal dominant 71, 617605 (3), Autosomal dominant; ?Polyendocrine-polyneuropathy syndrome, 616113 (3), Autosomal recessive
DNA2	601810	?Seckel syndrome 8, 615807 (3), Autosomal recessive; Progressive external ophthalmoplegia with mitochondrial DNA deletions, autosomal dominant 6, 615156 (3), Autosomal dominant
DNAAF1	613190	Ciliary dyskinesia, primary, 13, 613193 (3), Autosomal recessive
DNAAF11	614930	Ciliary dyskinesia, primary, 19, 614935 (3), Autosomal recessive
DNAAF2	612517	Ciliary dyskinesia, primary, 10, 612518 (3), Autosomal recessive
DNAAF3	614566	Ciliary dyskinesia, primary, 2, 606763 (3), Autosomal recessive
DNAAF4	608706	{Dyslexia, susceptibility to, 1}, 127700 (3), Autosomal dominant; Ciliary dyskinesia, primary, 25, 615482 (3), Autosomal recessive
DNAAF5	614864	Ciliary dyskinesia, primary, 18, 614874 (3), Autosomal recessive
DNAAF6	300933	Ciliary dyskinesia, primary, 36, X-linked, 300991 (3), X-linked recessive
DNAH1	603332	Spermatogenic failure 18, 617576 (3), Autosomal recessive; ?Ciliary dyskinesia, primary, 37, 617577 (3), Autosomal recessive
DNAH10	605884	Spermatogenic failure 56, 619515 (3), Autosomal recessive
DNAH11	603339	Ciliary dyskinesia, primary, 7, with or without situs inversus, 611884 (3), Autosomal recessive
DNAH17	610063	Spermatogenic failure 39, 618643 (3), Autosomal recessive
DNAH2	603333	Spermatogenic failure 45, 619094 (3), Autosomal recessive
DNAH5	603335	Ciliary dyskinesia, primary, 3, with or without situs inversus, 608644 (3), Autosomal recessive
DNAH8	603337	Spermatogenic failure 46, 619095 (3), Autosomal recessive
DNAH9	603330	Ciliary dyskinesia, primary, 40, 618300 (3), Autosomal recessive
DNAI1	604366	Ciliary dyskinesia, primary, 1, with or without situs inversus, 244400 (3), Autosomal recessive

DNAI2	605483	Ciliary dyskinesia, primary, 9, with or without situs inversus, 612444 (3), Autosomal recessive
DNAJB11	611341	Polycystic kidney disease 6 with or without polycystic liver disease, 618061 (3), Autosomal dominant
DNAJB13	610263	Ciliary dyskinesia, primary, 34, 617091 (3), Autosomal recessive
DNAJB2	604139	Spinal muscular atrophy, distal, autosomal recessive, 5, 614881 (3), Autosomal recessive
DNAJB6	611332	Muscular dystrophy, limb-girdle, autosomal dominant 1, 603511 (3), Autosomal dominant
DNAJC12	606060	Hyperphenylalaninemia, mild, non-BH4-deficient, 617384 (3), Autosomal recessive
DNAJC19	608977	3-methylglutaconic aciduria, type V, 610198 (3), Autosomal recessive
DNAJC21	617048	Bone marrow failure syndrome 3, 617052 (3), Autosomal recessive
DNAJC3	601184	Ataxia, combined cerebellar and peripheral, with hearing loss and diabetes mellitus, 616192 (3), Autosomal recessive
DNAJC30	618202	Leber hereditary optic neuropathy, autosomal recessive, 619382 (3), Autosomal recessive
DNAJC5	611203	Ceroid lipofuscinosis, neuronal, 4 (Kufs type), autosomal dominant, 162350 (3), Autosomal dominant
DNAJC6	608375	Parkinson disease 19a, juvenile-onset, 615528 (3), Autosomal recessive; Parkinson disease 19b, early-onset, 615528 (3), Autosomal recessive
DNAL1	610062	Ciliary dyskinesia, primary, 16, 614017 (3), Autosomal recessive
DNAL4	610565	?Mirror movements 3, 616059 (3), Autosomal recessive
DNASE1	125505	{Systemic lupus erythematosus, susceptibility to}, 152700 (3), Autosomal dominant
DNASE1L3	602244	Systemic lupus erythematosus 16, 614420 (3), Autosomal recessive
DNASE2	126350	Autoinflammatory-pancytopenia syndrome, 619858 (3), Autosomal recessive
DNHD1	617277	Spermatogenic failure 65, 619712 (3), Autosomal recessive
DNM1	602377	Developmental and epileptic encephalopathy 31, 616346 (3), Autosomal dominant
DNM1L	603850	Optic atrophy 5, 610708 (3), Autosomal dominant; Encephalopathy, lethal, due to defective mitochondrial peroxisomal fission 1, 614388 (3), Autosomal dominant, Autosomal recessive
DNM2	602378	Centronuclear myopathy 1, 160150 (3), Autosomal dominant; Charcot-Marie-Tooth disease, axonal type 2M, 606482 (3), Autosomal dominant; Charcot-Marie-Tooth disease, dominant intermediate B, 606482 (3), Autosomal dominant; Lethal congenital contracture syndrome 5, 615368 (3), Autosomal recessive
DNMBP	611282	Cataract 48, 618415 (3), Autosomal recessive
DNMT1	126375	Neuropathy, hereditary sensory, type IE, 614116 (3), Autosomal dominant; Cerebellar ataxia, deafness, and narcolepsy, autosomal dominant, 604121 (3), Autosomal dominant

DNMT3A	602769	Tatton-Brown-Rahman syndrome, 615879 (3), Autosomal dominant; Acute myeloid leukemia, somatic, 601626 (3); Heyn-Sproul-Jackson syndrome, 618724 (3), Autosomal dominant
DNMT3B	602900	Immunodeficiency-centromeric instability-facial anomalies syndrome 1, 242860 (3), Autosomal recessive; Facioscapulohumeral muscular dystrophy 4, digenic, 619478 (3), Digenic dominant
DOCK2	603122	Immunodeficiency 40, 616433 (3), Autosomal recessive
DOCK3	603123	Neurodevelopmental disorder with impaired intellectual development, hypotonia, and ataxia, 618292 (3), Autosomal recessive
DOCK6	614194	Adams-Oliver syndrome 2, 614219 (3), Autosomal recessive
DOCK7	615730	Developmental and epileptic encephalopathy 23, 615859 (3), Autosomal recessive
DOCK8	611432	Hyper-IgE recurrent infection syndrome, autosomal recessive, 243700 (3), Autosomal recessive
DOK7	610285	Fetal akinesia deformation sequence 3, 618389 (3), Autosomal recessive; Myasthenic syndrome, congenital, 10, 254300 (3), Autosomal recessive
DOLK	610746	Congenital disorder of glycosylation, type Im, 610768 (3), Autosomal recessive
DONSON	611428	Microcephaly, short stature, and limb abnormalities, 617604 (3), Autosomal recessive; Microcephaly-micromelia syndrome, 251230 (3), Autosomal recessive
DPAGT1	191350	Myasthenic syndrome, congenital, 13, with tubular aggregates, 614750 (3), Autosomal recessive; Congenital disorder of glycosylation, type Ij, 608093 (3), Autosomal recessive
DPF2	601671	Coffin-Siris syndrome 7, 618027 (3), Autosomal dominant
DPH1	603527	Developmental delay with short stature, dysmorphic facial features, and sparse hair, 616901 (3), Autosomal recessive
DPM1	603503	Congenital disorder of glycosylation, type Ie, 608799 (3), Autosomal recessive
DPM2	603564	Congenital disorder of glycosylation, type Iu, 615042 (3), Autosomal recessive
DPM3	605951	?Muscular dystrophy-dystroglycanopathy (congenital with impaired intellectual development), type B, 15, 618992 (3), Autosomal recessive; Muscular dystrophy-dystroglycanopathy (limb-girdle), type C, 15, 612937 (3), Autosomal recessive
DPP6	126141	Intellectual developmental disorder, autosomal dominant 33, 616311 (3); {Ventricular fibrillation, paroxysmal familial, 2}, 612956 (3), Autosomal dominant
DPY19L2	613893	Spermatogenic failure 9, 613958 (3), Autosomal recessive
DPYD	612779	Dihydropyrimidine dehydrogenase deficiency, 274270 (3), Autosomal recessive; 5-fluorouracil toxicity, 274270 (3), Autosomal recessive
DPYS	613326	Dihydropyrimidinuria, 222748 (3), Autosomal recessive
DPYSL5	608383	Ritscher-Schinzel syndrome 4, 619435 (3), Autosomal dominant
DRAM2	613360	Cone-rod dystrophy 21, 616502 (3), Autosomal recessive

DRC1	615288	Ciliary dyskinesia, primary, 21, 615294 (3), Autosomal recessive
DRD3	126451	{Essential tremor, hereditary, 1}, 190300 (3), Autosomal dominant; {Schizophrenia, susceptibility to}, 181500 (3), Autosomal dominant
DRD4	126452	{Attention deficit-hyperactivity disorder}, 143465 (3), Autosomal dominant; Autonomic nervous system dysfunction (3)
DRD5	126453	{Blepharospasm, primary benign}, 606798 (3), Autosomal dominant; {Attention deficit-hyperactivity disorder, susceptibility to}, 143465 (3), Autosomal dominant
DSC2	125645	Arrhythmogenic right ventricular dysplasia 11 with mild palmoplantar keratoderma and woolly hair, 610476 (3), Autosomal dominant, Autosomal recessive; Arrhythmogenic right ventricular dysplasia 11, 610476 (3), Autosomal dominant, Autosomal recessive
DSC3	600271	Hypotrichosis and recurrent skin vesicles, 613102 (3), Autosomal recessive
DSE	605942	Ehlers-Danlos syndrome, musculocontractural type 2, 615539 (3), Autosomal recessive
DSG1	125670	Keratosis palmoplantaris striata I, AD, 148700 (3), Autosomal dominant; Erythroderma, congenital, with palmoplantar keratoderma, hypotrichosis, and hyper IgE, 615508 (3), Autosomal recessive
DSG2	125671	Cardiomyopathy, dilated, 1BB, 612877 (3), Autosomal recessive; Arrhythmogenic right ventricular dysplasia 10, 610193 (3), Autosomal dominant
DSG3	169615	Blistering, acantholytic, of oral and laryngeal mucosa, 619226 (3), Autosomal recessive
DSG4	607892	Hypotrichosis 6, 607903 (3), Autosomal recessive
DSP	125647	Arrhythmogenic right ventricular dysplasia 8, 607450 (3), Autosomal dominant; Skin fragility-woolly hair syndrome, 607655 (3), Autosomal recessive; Epidermolysis bullosa, lethal acantholytic, 609638 (3), Autosomal recessive; Keratosis palmoplantaris striata II, 612908 (3), Autosomal dominant; Dilated cardiomyopathy with woolly hair, keratoderma, and tooth agenesis, 615821 (3), Autosomal dominant; Cardiomyopathy, dilated, with woolly hair and keratoderma, 605676 (3), Autosomal recessive
DSPP	125485	Dentinogenesis imperfecta, Shields type III, 125500 (3), Autosomal dominant; Dentinogenesis imperfecta, Shields type II, 125490 (3), Autosomal dominant; Dentin dysplasia, type II, 125420 (3), Autosomal dominant; Deafness, autosomal dominant 39, with dentinogenesis, 605594 (3), Autosomal dominant
DST	113810	Neuropathy, hereditary sensory and autonomic, type VI, 614653 (3), Autosomal recessive; Epidermolysis bullosa simplex 3, localized or generalized intermediate, with bp230 deficiency, 615425 (3), Autosomal recessive
DSTYK	612666	Congenital anomalies of kidney and urinary tract 1, 610805 (3), Autosomal dominant; Spastic paraplegia 23, 270750 (3), Autosomal recessive
DTNA	601239	Left ventricular noncompaction 1, with or without congenital heart defects, 604169 (3), Autosomal dominant

DTNBP1	607145	Hermansky-Pudlak syndrome 7, 614076 (3), Autosomal recessive
DTYMK	188345	Neurodegeneration, childhood-onset, with progressive microcephaly, 619847 (3), Autosomal recessive
DUOX2	606759	Thyroid dyshormonogenesis 6, 607200 (3), Autosomal recessive
DUOXA2	612772	Thyroid dyshormonogenesis 5, 274900 (3), Autosomal recessive
DUSP6	602748	Hypogonadotropic hypogonadism 19 with or without anosmia, 615269 (3), Autosomal dominant
DVL1	601365	Robinow syndrome, autosomal dominant 2, 616331 (3), Autosomal dominant
DVL3	601368	Robinow syndrome, autosomal dominant 3, 616894 (3), Autosomal dominant
DYM	607461	Smith-McCort dysplasia, 607326 (3), Autosomal recessive; Dyggve-Melchior-Clausen disease, 223800 (3), Autosomal recessive
DYNC1H1	600112	Charcot-Marie-Tooth disease, axonal, type 2O, 614228 (3), Autosomal dominant; Spinal muscular atrophy, lower extremity-predominant 1, AD, 158600 (3), Autosomal dominant; Intellectual developmental disorder, autosomal dominant 13, 614563 (3), Autosomal dominant
DYNC1I2	603331	Neurodevelopmental disorder with microcephaly and structural brain anomalies, 618492 (3), Autosomal recessive
DYNC2H1	603297	Short-rib thoracic dysplasia 3 with or without polydactyly, 613091 (3), Digenic recessive, Autosomal recessive
DYNC2I1	615462	Short-rib thoracic dysplasia 8 with or without polydactyly, 615503 (3), Autosomal recessive
DYNC2I2	613363	Short-rib thoracic dysplasia 11 with or without polydactyly, 615633 (3), Autosomal recessive
DYNC2LI1	617083	Short-rib thoracic dysplasia 15 with polydactyly, 617088 (3), Autosomal recessive
DYNLT2B	617353	Short-rib thoracic dysplasia 17 with or without polydactyly, 617405 (3), Autosomal recessive
DYRK1A	600855	Intellectual developmental disorder, autosomal dominant 7, 614104 (3), Autosomal dominant
DYRK1B	604556	Abdominal obesity-metabolic syndrome 3, 615812 (3), Autosomal dominant
DYSF	603009	Muscular dystrophy, limb-girdle, autosomal recessive 2, 253601 (3), Autosomal recessive; Miyoshi muscular dystrophy 1, 254130 (3), Autosomal recessive; Myopathy, distal, with anterior tibial onset, 606768 (3), Autosomal recessive
DZIP1	608671	Spermatogenic failure 47, 619102 (3), Autosomal recessive; ?Mitral valve prolapse 3, 610840 (3), Autosomal dominant
DZIP1L	617570	Polycystic kidney disease 5, 617610 (3), Autosomal recessive
EARS2	612799	Combined oxidative phosphorylation deficiency 12, 614924 (3), Autosomal recessive
EBF3	607407	Hypotonia, ataxia, and delayed development syndrome, 617330 (3), Autosomal dominant

EBP	300205	MEND syndrome, 300960 (3), X-linked recessive; Chondrodysplasia punctata, X-linked dominant, 302960 (3), X-linked dominant
ECE1	600423	{Hypertension, essential, susceptibility to}, 145500 (3), Multifactorial; ?Hirschsprung disease, cardiac defects, and autonomic dysfunction, 613870 (3), Autosomal dominant
ECEL1	605896	Arthrogryposis, distal, type 5D, 615065 (3), Autosomal recessive
ECHS1	602292	Mitochondrial short-chain enoyl-CoA hydratase 1 deficiency, 616277 (3), Autosomal recessive
ECM1	602201	Urbach-Wiethe disease, 247100 (3), Autosomal recessive
EDA	300451	Tooth agenesis, selective, X-linked 1, 313500 (3), X-linked dominant; Ectodermal dysplasia 1, hypohidrotic, X-linked, 305100 (3), X-linked recessive
EDAR	604095	[Hair morphology 1, hair thickness], 612630 (3); Ectodermal dysplasia 10A, hypohidrotic/hair/nail type, autosomal dominant, 129490 (3), Autosomal dominant; Ectodermal dysplasia 10B, hypohidrotic/hair/tooth type, autosomal recessive, 224900 (3), Autosomal recessive
EDARADD	606603	Ectodermal dysplasia 11B, hypohidrotic/hair/tooth type, autosomal recessive, 614941 (3), Autosomal recessive; Ectodermal dysplasia 11A, hypohidrotic/hair/tooth type, autosomal dominant, 614940 (3), Autosomal dominant
EDC3	609842	?Intellectual developmental disorder, autosomal recessive 50, 616460 (3), Autosomal recessive
EDEM3	610214	Congenital disorder of glycosylation, type 2V, 619493 (3), Autosomal recessive
EDN1	131240	{High density lipoprotein cholesterol level QTL 7} (3); Question mark ears, isolated, 612798 (3), Autosomal dominant; Auriculocondylar syndrome 3, 615706 (3), Autosomal recessive
EDN3	131242	Waardenburg syndrome, type 4B, 613265 (3), Autosomal dominant, Autosomal recessive; {Hirschsprung disease, susceptibility to, 4}, 613712 (3), Autosomal dominant
EDNRA	131243	{Migraine, resistance to}, 157300 (3), Autosomal dominant; Mandibulofacial dysostosis with alopecia, 616367 (3), Autosomal dominant
EDNRB	131244	{Hirschsprung disease, susceptibility to, 2}, 600155 (3), Autosomal dominant; ?ABCD syndrome, 600501 (3), Autosomal recessive; Waardenburg syndrome, type 4A, 277580 (3), Autosomal dominant, Autosomal recessive
EED	605984	Cohen-Gibson syndrome, 617561 (3), Autosomal dominant
EEF1A2	602959	Developmental and epileptic encephalopathy 33, 616409 (3), Autosomal dominant; Intellectual developmental disorder, autosomal dominant 38, 616393 (3), Autosomal dominant
EEF2	130610	?Spinocerebellar ataxia 26, 609306 (3), Autosomal dominant
EFEMP1	601548	Doyne honeycomb degeneration of retina, 126600 (3), Autosomal dominant
EFEMP2	604633	Cutis laxa, autosomal recessive, type IB, 614437 (3), Autosomal recessive

EFHC1	608815	{Epilepsy, juvenile absence, susceptibility to, 1}, 607631 (3), Autosomal dominant; {Myoclonic epilepsy, juvenile, susceptibility to, 1}, 254770 (3), Autosomal dominant
EFL1	617538	Shwachman-Diamond syndrome 2, 617941 (3), Autosomal recessive
EFNB1	300035	Craniofrontonasal dysplasia, 304110 (3), X-linked dominant
EFTUD2	603892	Mandibulofacial dysostosis, Guion-Almeida type, 610536 (3), Autosomal dominant
EGF	131530	?Hypomagnesemia 4, renal, 611718 (3), Autosomal recessive
EGFR	131550	?Inflammatory skin and bowel disease, neonatal, 2, 616069 (3), Autosomal recessive; Nonsmall cell lung cancer, response to tyrosine kinase inhibitor in, 211980 (3), Autosomal dominant, Somatic mutation; Adenocarcinoma of lung, response to tyrosine kinase inhibitor in, 211980 (3), Autosomal dominant, Somatic mutation; {Nonsmall cell lung cancer, susceptibility to}, 211980 (3), Autosomal dominant, Somatic mutation
EGLN1	606425	Erythrocytosis, familial, 3, 609820 (3), Autosomal dominant; [Hemoglobin, high altitude adaptation], 609070 (3), Autosomal dominant
EGR2	129010	Dejerine-Sottas disease, 145900 (3), Autosomal dominant, Autosomal recessive; Charcot-Marie-Tooth disease, type 1D, 607678 (3), Autosomal dominant; Hypomyelinating neuropathy, congenital, 1, 605253 (3), Autosomal dominant, Autosomal recessive
EHBP1	609922	{Prostate cancer, hereditary, 12}, 611868 (3)
EHHADH	607037	?Fanconi renotubular syndrome 3, 615605 (3), Autosomal dominant
EHMT1	607001	Kleefstra syndrome 1, 610253 (3), Autosomal dominant
EIF2AK1	613635	?Leukoencephalopathy, motor delay, spasticity, and dysarthria syndrome, 618878 (3), Autosomal dominant
EIF2AK2	176871	Leukoencephalopathy, developmental delay, and episodic neurologic regression syndrome, 618877 (3), Autosomal dominant; Dystonia 33, 619687 (3), Autosomal dominant, Autosomal recessive
EIF2AK3	604032	Wolcott-Rallison syndrome, 226980 (3), Autosomal recessive
EIF2AK4	609280	Pulmonary venoocclusive disease 2, 234810 (3), Autosomal recessive
EIF2B1	606686	Leukoencephalopathy with vanishing white matter, 603896 (3), Autosomal recessive
EIF2B2	606454	Leukoencephalopathy with vanishing white matter, 603896 (3), Autosomal recessive; Ovarioleukodystrophy, 603896 (3), Autosomal recessive
EIF2B3	606273	Leukoencephalopathy with vanishing white matter, 603896 (3), Autosomal recessive
EIF2B4	606687	Ovarioleukodystrophy, 603896 (3), Autosomal recessive; Leukoencephalopathy with vanishing white matter, 603896 (3), Autosomal recessive
EIF2B5	603945	Ovarioleukodystrophy, 603896 (3), Autosomal recessive; Leukoencephalopathy with vanishing white matter, 603896 (3), Autosomal recessive
EIF2S3	300161	MEHMO syndrome, 300148 (3), X-linked recessive

EIF3F	603914	Intellectual developmental disorder, autosomal recessive 67, 618295 (3), Autosomal recessive
EIF4A3	608546	Robin sequence with cleft mandible and limb anomalies, 268305 (3), Autosomal recessive
EIF4E	133440	{Autism, susceptibility to, 19}, 615091 (3)
EIF4G1	600495	{Parkinson disease 18}, 614251 (3), Autosomal dominant
EIF5A	600187	Faundes-Banka syndrome, 619376 (3), Autosomal dominant
ELAC2	605367	{Prostate cancer, hereditary, 2, susceptibility to}, 614731 (3); Combined oxidative phosphorylation deficiency 17, 615440 (3), Autosomal recessive
ELANE	130130	Neutropenia, cyclic, 162800 (3), Autosomal dominant; Neutropenia, severe congenital 1, autosomal dominant, 202700 (3), Autosomal dominant
ELF4	300775	Autoinflammatory syndrome, familial, X-linked, Behcet-like 2, 301074 (3), X-linked recessive
ELMO2	606421	Vascular malformation, primary intraosseous, 606893 (3), Autosomal recessive
ELMOD3	615427	?Deafness, autosomal recessive 88, 615429 (3), Autosomal recessive; ?Deafness, autosomal dominant 81, 619500 (3), Autosomal dominant
ELN	130160	Cutis laxa, autosomal dominant, 123700 (3), Autosomal dominant; Supravalvar aortic stenosis, 185500 (3), Autosomal dominant
ELOVL1	611813	Ichthyotic keratoderma, spasticity, hypomyelination, and dysmorphic facies, 618527 (3), Autosomal dominant, Autosomal recessive
ELOVL4	605512	Spinocerebellar ataxia 34, 133190 (3), Autosomal dominant; Stargardt disease 3, 600110 (3), Autosomal dominant; Ichthyosis, spastic quadriplegia, and mental retardation, 614457 (3), Autosomal recessive
ELOVL5	611805	Spinocerebellar ataxia 38, 615957 (3), Autosomal dominant
ELP1	603722	Dysautonomia, familial, 223900 (3), Autosomal recessive; Medulloblastoma, 155255 (3), Autosomal dominant, Somatic mutation, Autosomal recessive
ELP2	616054	Intellectual developmental disorder, autosomal recessive 58, 617270 (3), Autosomal recessive
ELP4	606985	?Aniridia 2, 617141 (3), Autosomal dominant
EMC1	616846	Cerebellar atrophy, visual impairment, and psychomotor retardation, 616875 (3), Autosomal recessive
EMC10	614545	Neurodevelopmental disorder with dysmorphic facies and variable seizures, 619264 (3), Autosomal recessive
EMD	300384	Emery-Dreifuss muscular dystrophy 1, X-linked, 310300 (3), X-linked recessive
EMG1	611531	Bowen-Conradi syndrome, 211180 (3), Autosomal recessive
EML1	602033	Band heterotopia, 600348 (3), Autosomal recessive
EMP2	602334	Nephrotic syndrome, type 10, 615861 (3), Autosomal recessive
EMX2	600035	Schizencephaly, 269160 (3)
EN1	131290	?ENDOVE syndrome, limb-brain type, 619218 (3), Autosomal recessive
ENAM	606585	Amelogenesis imperfecta, type IC, 204650 (3), Autosomal recessive; Amelogenesis imperfecta, type IB, 104500 (3), Autosomal dominant

ENG	131195	Telangiectasia, hereditary hemorrhagic, type 1, 187300 (3), Autosomal dominant
ENO3	131370	Glycogen storage disease XIII, 612932 (3), Autosomal recessive
ENPP1	173335	{Obesity, susceptibility to}, 601665 (3), Multifactorial, Autosomal dominant, Autosomal recessive; Hypophosphatemic rickets, autosomal recessive, 2, 613312 (3), Autosomal recessive; {Diabetes mellitus, non-insulin-dependent, susceptibility to}, 125853 (3), Autosomal dominant; Arterial calcification, generalized, of infancy, 1, 208000 (3), Autosomal recessive; Cole disease, 615522 (3), Autosomal dominant
ENTPD1	601752	Spastic paraplegia 64, autosomal recessive, 615683 (3), Autosomal recessive
EOGT	614789	Adams-Oliver syndrome 4, 615297 (3), Autosomal recessive
EP300	602700	Menke-Hennekam syndrome 2, 618333 (3), Autosomal dominant; Colorectal cancer, somatic, 114500 (3); Rubinstein-Taybi syndrome 2, 613684 (3), Autosomal dominant
EPAS1	603349	Erythrocytosis, familial, 4, 611783 (3), Autosomal dominant
EPB41	130500	Elliptocytosis-1, 611804 (3), Autosomal dominant, Autosomal recessive
EPB41L1	602879	?Intellectual developmental disorder, autosomal dominant 11, 614257 (3), Autosomal dominant
EPB42	177070	Spherocytosis, type 5, 612690 (3)
EPCAM	185535	Colorectal cancer, hereditary nonpolyposis, type 8, 613244 (3), Autosomal dominant; Diarrhea 5, with tufting enteropathy, congenital, 613217 (3), Autosomal recessive
EPG5	615068	Vici syndrome, 242840 (3), Autosomal recessive
EPHA2	176946	Cataract 6, multiple types, 116600 (3), Autosomal dominant
EPHB2	600997	?Bleeding disorder, platelet-type, 22, 618462 (3), Autosomal recessive; {Prostate cancer/brain cancer susceptibility, somatic}, 603688 (3)
EPHB4	600011	Capillary malformation-arteriovenous malformation 2, 618196 (3), Autosomal dominant; Lymphatic malformation 7, 617300 (3), Autosomal dominant
EPHX2	132811	{Hypercholesterolemia, familial, due to LDLR defect, modifier of}, 143890 (3), Autosomal dominant, Autosomal recessive
EPM2A	607566	Epilepsy, progressive myoclonic 2A (Lafora), 254780 (3), Autosomal recessive
EPO	133170	{Microvascular complications of diabetes 2}, 612623 (3); Erythrocytosis, familial, 5, 617907 (3), Autosomal dominant; ?Diamond-Blackfan anemia-like, 617911 (3), Autosomal recessive
EPOR	133171	[Erythrocytosis, familial, 1], 133100 (3), Autosomal dominant
EPRS1	138295	Leukodystrophy, hypomyelinating, 15, 617951 (3), Autosomal recessive
EPS8	600206	?Deafness, autosomal recessive 102, 615974 (3), Autosomal recessive
EPS8L2	614988	Deafness autosomal recessive 106, 617637 (3), Autosomal recessive
EPS8L3	614989	?Hypotrichosis 5, 612841 (3), Autosomal dominant
EPX	131399	[Eosinophil peroxidase deficiency], 261500 (3), Autosomal recessive
ERAL1	607435	Perrault syndrome 6, 617565 (3), Autosomal recessive

ERBB2	164870	Gastric cancer, somatic, 613659 (3); Adenocarcinoma of lung, somatic, 211980 (3); Ovarian cancer, somatic, 167000 (3); ?Visceral neuropathy, familial, 2, autosomal recessive, 619465 (3), Autosomal recessive; Glioblastoma, somatic, 137800 (3)
ERBB3	190151	?Lethal congenital contractural syndrome 2, 607598 (3), Autosomal recessive; {?Erythroleukemia, familial, susceptibility to}, 133180 (3), Autosomal dominant; Visceral neuropathy, familial, 1, autosomal recessive, 243180 (3), Autosomal recessive
ERBB4	600543	Amyotrophic lateral sclerosis 19, 615515 (3), Autosomal dominant
ERCC1	126380	Cerebrooculofacioskeletal syndrome 4, 610758 (3), Autosomal recessive
ERCC2	126340	Xeroderma pigmentosum, group D, 278730 (3), Autosomal recessive; Trichothiodystrophy 1, photosensitive, 601675 (3), Autosomal recessive; ?Cerebrooculofacioskeletal syndrome 2, 610756 (3), Autosomal recessive
ERCC3	133510	Trichothiodystrophy 2, photosensitive, 616390 (3), Autosomal recessive; Xeroderma pigmentosum, group B, 610651 (3), Autosomal recessive
ERCC4	133520	Xeroderma pigmentosum, type F/Cockayne syndrome, 278760 (3), Autosomal recessive; XFE progeroid syndrome, 610965 (3), Autosomal recessive; Xeroderma pigmentosum, group F, 278760 (3), Autosomal recessive; Fanconi anemia, complementation group Q, 615272 (3), Autosomal recessive
ERCC5	133530	Xeroderma pigmentosum, group G, 278780 (3), Autosomal recessive; Cerebrooculofacioskeletal syndrome 3, 616570 (3), Autosomal recessive; Xeroderma pigmentosum, group G/Cockayne syndrome, 278780 (3), Autosomal recessive
ERCC6	609413	UV-sensitive syndrome 1, 600630 (3), Autosomal recessive; Cerebrooculofacioskeletal syndrome 1, 214150 (3), Autosomal recessive; ?De Sanctis-Cacchione syndrome, 278800 (3), Autosomal recessive; Cockayne syndrome, type B, 133540 (3), Autosomal recessive; {Macular degeneration, age-related, susceptibility to, 5}, 613761 (3); Premature ovarian failure 11, 616946 (3), Autosomal dominant; {Lung cancer, susceptibility to}, 211980 (3), Autosomal dominant, Somatic mutation
ERCC6L2	615667	Bone marrow failure syndrome 2, 615715 (3), Autosomal recessive
ERCC8	609412	UV-sensitive syndrome 2, 614621 (3), Autosomal recessive; Cockayne syndrome, type A, 216400 (3), Autosomal recessive
ERF	611888	Craniosynostosis 4, 600775 (3), Autosomal dominant; Chitayat syndrome, 617180 (3), Autosomal dominant
ERGIC1	617946	?Arthrogryposis multiplex congenita 2, neurogenic type, 208100 (3), Autosomal recessive
ERLIN1	611604	Spastic paraplegia 62, 615681 (3), Autosomal recessive
ERLIN2	611605	Spastic paraplegia 18, autosomal recessive, 611225 (3), Autosomal recessive
ERMAP	609017	[Blood group, Scianna system], 111750 (3); [Blood group, Radin], 111620 (3)
ERMARD	615532	?Periventricular nodular heterotopia 6, 615544 (3), Autosomal dominant

ESCO2	609353	Juberg-Hayward syndrome, 216100 (3), Autosomal recessive; Roberts-SC phocomelia syndrome, 268300 (3), Autosomal recessive
ESPN	606351	Deafness, neurosensory, without vestibular involvement, autosomal dominant, 609006 (3), Autosomal recessive; Deafness, autosomal recessive 36, 609006 (3), Autosomal recessive; ?Usher syndrome, type 1M, 618632 (3), Autosomal recessive
ESR1	133430	Breast cancer, somatic, 114480 (3); {Migraine, susceptibility to}, 157300 (3), Autosomal dominant; Estrogen resistance, 615363 (3), Autosomal recessive; {Myocardial infarction, susceptibility to}, 608446 (3)
ESR2	601663	?Ovarian dysgenesis 8, 618187 (3), Autosomal dominant
ESRP1	612959	?Deafness, autosomal recessive 109, 618013 (3), Autosomal recessive
ESRRB	602167	Deafness, autosomal recessive 35, 608565 (3), Autosomal recessive
ETFA	608053	Glutaric acidemia IIA, 231680 (3), Autosomal recessive
ETFB	130410	Glutaric acidemia IIB, 231680 (3), Autosomal recessive
ETFDH	231675	Glutaric acidemia IIC, 231680 (3), Autosomal recessive
ETHE1	608451	Ethylmalonic encephalopathy, 602473 (3), Autosomal recessive
ETV6	600618	Thrombocytopenia 5, 616216 (3), Autosomal dominant; Leukemia, acute myeloid, somatic, 601626 (3)
EVC	604831	Ellis-van Creveld syndrome, 225500 (3), Autosomal recessive; ?Weyers acrofacial dysostosis, 193530 (3), Autosomal dominant
EVC2	607261	Ellis-van Creveld syndrome, 225500 (3), Autosomal recessive; Weyers acrofacial dysostosis, 193530 (3), Autosomal dominant
EWSR1	133450	Neuroepithelioma, 612219 (3); Ewing sarcoma, 612219 (3)
EXOC2	615329	Neurodevelopmental disorder with dysmorphic facies and cerebellar hypoplasia, 619306 (3), Autosomal recessive
EXOC3L2	616927	Meckel-Gruber-like syndrome (AR) PMID: 34974531
EXOC6B	607880	Spondyloepimetaphyseal dysplasia with joint laxity, type 3, 618395 (3), Autosomal recessive
EXOC7	608163	Neurodevelopmental disorder with seizures and brain atrophy, 619072 (3), Autosomal recessive
EXOC8	615283	?Neurodevelopmental disorder with microcephaly, seizures, and brain atrophy, 619076 (3), Autosomal recessive
EXOSC1	606493	?Pontocerebellar hypoplasia, type 1F, 619304 (3), Autosomal recessive
EXOSC2	602238	Short stature, hearing loss, retinitis pigmentosa, and distinctive facies, 617763 (3), Autosomal recessive
EXOSC3	606489	Pontocerebellar hypoplasia, type 1B, 614678 (3), Autosomal recessive
EXOSC5	606492	Cerebellar ataxia, brain abnormalities, and cardiac conduction defects, 619576 (3), Autosomal recessive
EXOSC8	606019	Pontocerebellar hypoplasia, type 1C, 616081 (3), Autosomal recessive
EXOSC9	606180	Pontocerebellar hypoplasia, type 1D, 618065 (3), Autosomal recessive
EXPH5	612878	Epidermolysis bullosa simplex 4, localized or generalized intermediate, autosomal recessive, 615028 (3), Autosomal recessive
EXT1	608177	Exostoses, multiple, type 1, 133700 (3), Autosomal dominant; Chondrosarcoma, 215300 (3), Somatic mutation

EXT2	608210	Seizures, scoliosis, and macrocephaly syndrome, 616682 (3), Autosomal recessive; Exostoses, multiple, type 2, 133701 (3), Autosomal dominant
EXTL3	605744	Immunoskeletal dysplasia with neurodevelopmental abnormalities, 617425 (3), Autosomal recessive
EYA1	601653	Branchiootic syndrome 1, 602588 (3), Autosomal dominant; Branchiootorenal syndrome 1, with or without cataracts, 113650 (3), Autosomal dominant; Anterior segment anomalies with or without cataract, 602588 (3), Autosomal dominant; ?Otofaciocervical syndrome, 166780 (3), Autosomal dominant
EYA4	603550	?Cardiomyopathy, dilated, 1J, 605362 (3), Autosomal dominant; Deafness, autosomal dominant 10, 601316 (3), Autosomal dominant
EYS	612424	Retinitis pigmentosa 25, 602772 (3), Autosomal recessive
EZH2	601573	Weaver syndrome, 277590 (3), Autosomal dominant
F10	613872	Factor X deficiency, 227600 (3), Autosomal recessive
F11	264900	Factor XI deficiency, autosomal dominant, 612416 (3); Factor XI deficiency, autosomal recessive, 612416 (3)
F12	610619	Angioedema, hereditary, 3, 610618 (3), Autosomal dominant; Factor XII deficiency, 234000 (3), Autosomal recessive
F13A1	134570	Factor XIIIA deficiency, 613225 (3), Autosomal recessive; {Myocardial infarction, protection against}, 608446 (3); {Venous thrombosis, protection against}, 188050 (3), Autosomal dominant
F13B	134580	Factor XIIIB deficiency, 613235 (3), Autosomal recessive
F2	176930	Hypoprothrombinemia, 613679 (3), Autosomal recessive; {Pregnancy loss, recurrent, susceptibility to, 2}, 614390 (3), Autosomal dominant; Dysprothrombinemia, 613679 (3), Autosomal recessive; Thrombophilia 1 due to thrombin defect, 188050 (3), Autosomal dominant; {Stroke, ischemic, susceptibility to}, 601367 (3), Multifactorial
F5	612309	Thrombophilia 2 due to activated protein C resistance, 188055 (3), Autosomal dominant; {Pregnancy loss, recurrent, susceptibility to, 1}, 614389 (3), Autosomal dominant; {Thrombophilia, susceptibility to, due to factor V Leiden}, 188055 (3), Autosomal dominant; {Budd-Chiari syndrome}, 600880 (3), Autosomal recessive; {Stroke, ischemic, susceptibility to}, 601367 (3), Multifactorial; Factor V deficiency, 227400 (3), Autosomal recessive
F7	613878	{Myocardial infarction, decreased susceptibility to}, 608446 (3); Factor VII deficiency, 227500 (3), Autosomal recessive
F8	300841	Thrombophilia 13, X-linked, due to factor VIII defect, 301071 (3); Hemophilia A, 306700 (3), X-linked recessive
F9	300746	{Deep venous thrombosis, protection against}, 300807 (3), X-linked recessive; Hemophilia B, 306900 (3), X-linked recessive; Thrombophilia 8, X-linked, due to factor IX defect, 300807 (3), X-linked recessive; {Warfarin sensitivity}, 301052 (3), X-linked
FA2H	611026	Spastic paraplegia 35, autosomal recessive, 612319 (3), Autosomal recessive
FAAH	602935	{Drug addiction, susceptibility to}, 606581 (3)

FADD	602457	Immunodeficiency 90 with encephalopathy, functional hyposplenia, and hepatic dysfunction, 613759 (3), Autosomal recessive
FAH	613871	Tyrosinemia, type I, 276700 (3), Autosomal recessive
FAM111A	615292	Kenny-Caffey syndrome, type 2, 127000 (3), Autosomal dominant; Gracile bone dysplasia, 602361 (3), Autosomal dominant
FAM111B	615584	Poikiloderma, hereditary fibrosing, with tendon contractures, myopathy, and pulmonary fibrosis, 615704 (3), Autosomal dominant
FAM149B1	618413	Joubert syndrome 36, 618763 (3), Autosomal recessive
FAM161A	613596	Retinitis pigmentosa 28, 606068 (3)
FAM20A	611062	Amelogenesis imperfecta, type IG (enamel-renal syndrome), 204690 (3), Autosomal recessive
FAM20C	611061	Raine syndrome, 259775 (3), Autosomal recessive
FAM50A	300453	Intellectual developmental disorder, X-linked syndromic, Armfield type, 300261 (3), X-linked recessive
FAM83H	611927	Amelogenesis imperfecta, type IIIA, 130900 (3), Autosomal dominant
FAN1	613534	Interstitial nephritis, karyomegalic, 614817 (3), Autosomal recessive
FANCA	607139	Fanconi anemia, complementation group A, 227650 (3), Autosomal recessive
FANCB	300515	Fanconi anemia, complementation group B, 300514 (3), X-linked recessive
FANCC	613899	Fanconi anemia, complementation group C, 227645 (3), Autosomal recessive
FANCD2	613984	Fanconi anemia, complementation group D2, 227646 (3), Autosomal recessive
FANCE	613976	Fanconi anemia, complementation group E, 600901 (3), Autosomal recessive
FANCF	613897	Fanconi anemia, complementation group F, 603467 (3), Autosomal recessive
FANCG	602956	Fanconi anemia, complementation group G, 614082 (3), Autosomal recessive
FANCI	611360	Fanconi anemia, complementation group I, 609053 (3), Autosomal recessive
FANCL	608111	Fanconi anemia, complementation group L, 614083 (3), Autosomal recessive
FANCM	609644	?Premature ovarian failure 15, 618096 (3), Autosomal recessive; Spermatogenic failure 28, 618086 (3), Autosomal recessive
FAR1	616107	Peroxisomal fatty acyl-CoA reductase 1 disorder, 616154 (3), Autosomal recessive; Cataracts, spastic paraparesis, and speech delay, 619338 (3), Autosomal dominant
FARS2	611592	Combined oxidative phosphorylation deficiency 14, 614946 (3), Autosomal recessive; Spastic paraplegia 77, autosomal recessive, 617046 (3), Autosomal recessive
FARSA	602918	?Rajab interstitial lung disease with brain calcifications 2, 619013 (3), Autosomal recessive
FARSB	609690	Rajab interstitial lung disease with brain calcifications 1, 613658 (3), Autosomal recessive

FAS	134637	Squamous cell carcinoma, burn scar-related, somatic (3); Autoimmune lymphoproliferative syndrome, type IA, 601859 (3), Autosomal dominant; {Autoimmune lymphoproliferative syndrome}, 601859 (3), Autosomal dominant
FASLG	134638	Autoimmune lymphoproliferative syndrome, type IB, 601859 (3), Autosomal dominant; {Lung cancer, susceptibility to}, 211980 (3), Autosomal dominant, Somatic mutation
FASTKD2	612322	Combined oxidative phosphorylation deficiency 44, 618855 (3), Autosomal recessive
FAT2	604269	Spinocerebellar ataxia 45, 617769 (3), Autosomal dominant
FAT4	612411	Van Maldergem syndrome 2, 615546 (3), Autosomal recessive; Hennekam lymphangiectasia-lymphedema syndrome 2, 616006 (3), Autosomal recessive
FBLN1	135820	Synpolydactyly, 3/3'4, associated with metacarpal and metatarsal synostoses, 608180 (4), Autosomal dominant
FBLN5	604580	Cutis laxa, autosomal recessive, type IA, 219100 (3), Autosomal recessive; Charcot-Marie-Tooth disease, demyelinating, type 1H, 619764 (3), Autosomal dominant; Macular degeneration, age-related, 3, 608895 (3), Autosomal dominant; Neuropathy, hereditary, with or without age-related macular degeneration, 608895 (3), Autosomal dominant; ?Cutis laxa, autosomal dominant 2, 614434 (3), Autosomal dominant
FBN1	134797	Geleophysic dysplasia 2, 614185 (3), Autosomal dominant; Weill-Marchesani syndrome 2, dominant, 608328 (3), Autosomal dominant; Ectopia lentis, familial, 129600 (3), Autosomal dominant; MASS syndrome, 604308 (3), Autosomal dominant; Marfan lipodystrophy syndrome, 616914 (3), Autosomal dominant; Acromicric dysplasia, 102370 (3), Autosomal dominant; Marfan syndrome, 154700 (3), Autosomal dominant; Stiff skin syndrome, 184900 (3), Autosomal dominant
FBN2	612570	Macular degeneration, early-onset, 616118 (3), Autosomal dominant; Contractural arachnodactyly, congenital, 121050 (3), Autosomal dominant
FBP1	611570	Fructose-1,6-bisphosphatase deficiency, 229700 (3), Autosomal recessive
FBP2	603027	?Leukodystrophy, childhood-onset, remitting, 619864 (3), Autosomal dominant
FBXL3	605653	Intellectual developmental disorder with short stature, facial anomalies, and speech defects, 606220 (3), Autosomal recessive
FBXL4	605654	Mitochondrial DNA depletion syndrome 13 (encephalomyopathic type), 615471 (3), Autosomal recessive
FBXO11	607871	Intellectual developmental disorder with dysmorphic facies and behavioral abnormalities, 618089 (3), Autosomal dominant
FBXO28	609100	Developmental and epileptic encephalopathy 100, 619777 (3), Autosomal dominant
FBXO31	609102	?Intellectual developmental disorder, autosomal recessive 45, 615979 (3), Autosomal recessive

FBXO38	608533	Neuronopathy, distal hereditary motor, type IID, 615575 (3), Autosomal dominant
FBXO43	609110	Oocyte maturation defect 12, 619697 (3), Autosomal recessive; Spermatogenic failure 64, 619696 (3), Autosomal recessive
FBXO7	605648	Parkinson disease 15, autosomal recessive, 260300 (3), Autosomal recessive
FBXW11	605651	Neurodevelopmental, jaw, eye, and digital syndrome, 618914 (3), Autosomal dominant
FBXW4	608071	Split-hand/foot malformation 3, gene duplication syndrome, 246560 (4), Autosomal dominant
FCGR1A	146760	[IgG receptor I, phagocytic, familial deficiency of] (3)
FCGR2A	146790	{Malaria, severe, susceptibility to}, 611162 (3); {Pseudomonas aeruginosa, susceptibility to chronic infection by, in cystic fibrosis}, 219700 (3), Autosomal recessive; {Lupus nephritis, susceptibility to}, 152700 (3), Autosomal dominant
FCGR2B	604590	{Systemic lupus erythematosus, susceptibility to}, 152700 (3), Autosomal dominant; {Malaria, resistance to}, 611162 (3)
FCGR3A	146740	Immunodeficiency 20, 615707 (3), Autosomal recessive
FCHO1	613437	Immunodeficiency 76, 619164 (3), Autosomal recessive
FCN3	604973	Immunodeficiency due to ficolin 3 deficiency, 613860 (3), Autosomal recessive
FCSK	608675	Congenital disorder of glycosylation with defective fucosylation 2, 618324 (3), Autosomal recessive
FDFT1	184420	Squalene synthase deficiency, 618156 (3), Autosomal recessive
FDPS	134629	Porokeratosis 9, multiple types, 616631 (3), Autosomal dominant
FDX2	614585	Mitochondrial myopathy, episodic, with optic atrophy and reversible leukoencephalopathy, 251900 (3), Autosomal recessive
FDXR	103270	Auditory neuropathy and optic atrophy, 617717 (3), Autosomal recessive
FECH	612386	Protoporphyria, erythropoietic, 1, 177000 (3), Autosomal recessive
FERMT1	607900	Kindler syndrome, 173650 (3), Autosomal recessive
FERMT3	607901	Leukocyte adhesion deficiency, type III, 612840 (3), Autosomal recessive
FEZF1	613301	Hypogonadotropic hypogonadism 22, with or without anosmia, 616030 (3), Autosomal recessive
FFAR4	609044	{Obesity, susceptibility to}, 607514 (3)
FGA	134820	Hypodysfibrinogenemia, congenital, 616004 (3); Dysfibrinogenemia, congenital, 616004 (3); Amyloidosis, familial visceral, 105200 (3), Autosomal dominant; Afibrinogenemia, congenital, 202400 (3), Autosomal recessive
FGB	134830	Hypofibrinogenemia, congenital, 202400 (3), Autosomal recessive; Dysfibrinogenemia, congenital, 616004 (3); Afibrinogenemia, congenital, 202400 (3), Autosomal recessive
FGD1	300546	Intellectual developmental disorder, X-linked syndromic 16, 305400 (3), X-linked recessive; Aarskog-Scott syndrome, 305400 (3), X-linked recessive
FGD4	611104	Charcot-Marie-Tooth disease, type 4H, 609311 (3), Autosomal recessive

FGF10	602115	Aplasia of lacrimal and salivary glands, 180920 (3), Autosomal dominant; LADD syndrome, 149730 (3), Autosomal dominant
FGF12	601513	Developmental and epileptic encephalopathy 47, 617166 (3), Autosomal dominant
FGF13	300070	Developmental and epileptic encephalopathy 90, 301058 (3), X-linked recessive, X-linked dominant
FGF14	601515	Spinocerebellar ataxia 27, 609307 (3), Autosomal dominant
FGF16	300827	Metacarpal 4-5 fusion, 309630 (3), X-linked recessive
FGF17	603725	Hypogonadotropic hypogonadism 20 with or without anosmia, 615270 (3), Autosomal dominant
FGF20	605558	?Renal hypodysplasia/aplasia 2, 615721 (3), Autosomal recessive
FGF23	605380	Tumoral calcinosis, hyperphosphatemic, familial, 2, 617993 (3), Autosomal recessive; Hypophosphatemic rickets, autosomal dominant, 193100 (3), Autosomal dominant
FGF3	164950	Deafness, congenital with inner ear agenesis, microtia, and microdontia, 610706 (3), Autosomal recessive
FGF5	165190	Trichomegaly, 190330 (3), Autosomal recessive
FGF8	600483	Hypogonadotropic hypogonadism 6 with or without anosmia, 612702 (3), Autosomal dominant
FGF9	600921	Multiple synostoses syndrome 3, 612961 (3), Autosomal dominant
FGFR1	136350	Pfeiffer syndrome, 101600 (3), Autosomal dominant; Hypogonadotropic hypogonadism 2 with or without anosmia, 147950 (3), Autosomal dominant; Jackson-Weiss syndrome, 123150 (3), Autosomal dominant; Hartsfield syndrome, 615465 (3), Autosomal dominant; Trigonocephaly 1, 190440 (3), Autosomal dominant; Osteoglophonic dysplasia, 166250 (3), Autosomal dominant; Encephalocraniocutaneous lipomatosis, somatic mosaic, 613001 (3)
FGFR2	176943	Bent bone dysplasia syndrome, 614592 (3), Autosomal dominant; LADD syndrome, 149730 (3), Autosomal dominant; Scaphocephaly, maxillary retrusion, and mental retardation, 609579 (3); Antley-Bixler syndrome without genital anomalies or disordered steroidogenesis, 207410 (3), Autosomal dominant; Scaphocephaly and Axenfeld-Rieger anomaly (3); Jackson-Weiss syndrome, 123150 (3), Autosomal dominant; Gastric cancer, somatic, 613659 (3); Craniofacial-skeletal-dermatologic dysplasia, 101600 (3), Autosomal dominant; Apert syndrome, 101200 (3), Autosomal dominant; Pfeiffer syndrome, 101600 (3), Autosomal dominant; Craniosynostosis, nonspecific (3); Beare-Stevenson cutis gyrata syndrome, 123790 (3), Autosomal dominant; Crouzon syndrome, 123500 (3), Autosomal dominant; Saethre-Chotzen syndrome, 101400 (3), Autosomal dominant

FGFR3	134934	Muenke syndrome, 602849 (3), Autosomal dominant; SADDAN, 616482 (3), Autosomal dominant; Hypochondroplasia, 146000 (3), Autosomal dominant; LADD syndrome, 149730 (3), Autosomal dominant; Thanatophoric dysplasia, type II, 187601 (3), Autosomal dominant; Nevus, epidermal, somatic, 162900 (3); CATSHL syndrome, 610474 (3), Autosomal dominant, Autosomal recessive; Thanatophoric dysplasia, type I, 187600 (3), Autosomal dominant; Spermatocytic seminoma, somatic, 273300 (3); Bladder cancer, somatic, 109800 (3); Achondroplasia, 100800 (3), Autosomal dominant; Cervical cancer, somatic, 603956 (3); Colorectal cancer, somatic, 114500 (3); Crouzon syndrome with acanthosis nigricans, 612247 (3), Autosomal dominant
FGFR4	134935	{Cancer progression/metastasis} (3)
FGG	134850	Dysfibrinogenemia, congenital, 616004 (3); Hypodysfibrinogenemia, 616004 (3); Hypofibrinogenemia, congenital, 202400 (3), Autosomal recessive; Afibrinogenemia, congenital, 202400 (3), Autosomal recessive
FH	136850	Leiomyomatosis and renal cell cancer, 150800 (3), Autosomal dominant; Fumarase deficiency, 606812 (3), Autosomal recessive
FHL1	300163	Myopathy, X-linked, with postural muscle atrophy, 300696 (3), X-linked recessive; Emery-Dreifuss muscular dystrophy 6, X-linked, 300696 (3), X-linked recessive; ?Uruguay faciocardiomusculoskeletal syndrome, 300280 (3), X-linked recessive; Scapuloperoneal myopathy, X-linked dominant, 300695 (3), X-linked dominant; Reducing body myopathy, X-linked 1b, with late childhood or adult onset, 300718 (3), X-linked; Reducing body myopathy, X-linked 1a, severe, infantile or early childhood onset, 300717 (3), X-linked dominant
FHOD3	609691	Cardiomyopathy, familial hypertrophic, 28, 619402 (3), Autosomal dominant
FIBP	608296	Thauvin-Robinet-Faivre syndrome, 617107 (3), Autosomal recessive
FIG4	609390	Yunis-Varon syndrome, 216340 (3), Autosomal recessive; ?Polymicrogyria, bilateral temporooccipital, 612691 (3), Autosomal recessive; Amyotrophic lateral sclerosis 11, 612577 (3), Autosomal dominant; Charcot-Marie-Tooth disease, type 4J, 611228 (3), Autosomal recessive
FIGLA	608697	Premature ovarian failure 6, 612310 (3), Autosomal dominant
FITM2	612029	Siddiqi syndrome, 618635 (3), Autosomal recessive
FKBP10	607063	Osteogenesis imperfecta, type XI, 610968 (3), Autosomal recessive; Bruck syndrome 1, 259450 (3), Autosomal recessive
FKBP14	614505	Ehlers-Danlos syndrome, kyphoscoliotic type, 2, 614557 (3), Autosomal recessive
FKBP5	602623	{Major depressive disorder and accelerated response to antidepressant drug treatment}, 608516 (3)
FKRP	606596	Muscular dystrophy-dystroglycanopathy (congenital with or without mental retardation), type B, 5, 606612 (3), Autosomal recessive; Muscular dystrophy-dystroglycanopathy (limb-girdle), type C, 5, 607155 (3), Autosomal recessive; Muscular dystrophy-dystroglycanopathy (congenital with brain and eye anomalies), type A, 5, 613153 (3), Autosomal recessive

FKTN	607440	Muscular dystrophy-dystroglycanopathy (limb-girdle), type C, 4, 611588 (3), Autosomal recessive; Muscular dystrophy-dystroglycanopathy (congenital with brain and eye anomalies), type A, 4, 253800 (3), Autosomal recessive; Muscular dystrophy-dystroglycanopathy (congenital without mental retardation), type B, 4, 613152 (3), Autosomal recessive; Cardiomyopathy, dilated, 1X, 611615 (3), Autosomal recessive
FLAD1	610595	Lipid storage myopathy due to flavin adenine dinucleotide synthetase deficiency, 255100 (3), Autosomal recessive
FLCN	607273	Birt-Hogg-Dube syndrome, 135150 (3), Autosomal dominant; Colorectal cancer, somatic, 114500 (3); Pneumothorax, primary spontaneous, 173600 (3), Autosomal dominant; Renal carcinoma, chromophobe, somatic, 144700 (3)
FLG	135940	Ichthyosis vulgaris, 146700 (3), Autosomal dominant, Autosomal recessive; {Dermatitis, atopic, susceptibility to, 2}, 605803 (3)
FLG2	616284	Peeling skin syndrome 6, 618084 (3), Autosomal recessive
FLI1	193067	Bleeding disorder, platelet-type, 21, 617443 (3), Autosomal dominant, Autosomal recessive
FLNA	300017	Otopalatodigital syndrome, type II, 304120 (3), X-linked dominant; Intestinal pseudoobstruction, neuronal, 300048 (3), X-linked recessive; Cardiac valvular dysplasia, X-linked, 314400 (3), X-linked; ?FG syndrome 2, 300321 (3), X-linked; Melnick-Needles syndrome, 309350 (3), X-linked dominant; Terminal osseous dysplasia, 300244 (3), X-linked dominant; Congenital short bowel syndrome, 300048 (3), X-linked recessive; Otopalatodigital syndrome, type I, 311300 (3), X-linked dominant; Heterotopia, periventricular, 1, 300049 (3), X-linked dominant; Frontometaphyseal dysplasia 1, 305620 (3), X-linked recessive
FLNB	603381	Larsen syndrome, 150250 (3), Autosomal dominant; Atelosteogenesis, type I, 108720 (3), Autosomal dominant; Atelosteogenesis, type III, 108721 (3), Autosomal dominant; Spondylocarpotarsal synostosis syndrome, 272460 (3), Autosomal recessive; Boomerang dysplasia, 112310 (3), Autosomal dominant
FLNC	102565	Cardiomyopathy, familial hypertrophic, 26, 617047 (3), Autosomal dominant; Cardiomyopathy, familial restrictive 5, 617047 (3), Autosomal dominant; Myopathy, distal, 4, 614065 (3), Autosomal dominant; Myopathy, myofibrillar, 5, 609524 (3), Autosomal dominant
FLRT3	604808	Hypogonadotropic hypogonadism 21 with anosmia, 615271 (3), Autosomal dominant
FLT3	136351	Leukemia, acute lymphoblastic, somatic, 613065 (3); Leukemia, acute myeloid, reduced survival in, somatic, 601626 (3); Leukemia, acute myeloid, somatic, 601626 (3)
FLT4	136352	Hemangioma, capillary infantile, somatic, 602089 (3); Lymphatic malformation 1, 153100 (3), Autosomal dominant; Congenital heart defects, multiple types, 7, 618780 (3), Autosomal dominant

FLVCR1	609144	Ataxia, posterior column, with retinitis pigmentosa, 609033 (3), Autosomal recessive
FLVCR2	610865	Proliferative vasculopathy and hydranencephaly-hydrocephaly syndrome, 225790 (3), Autosomal recessive
FMN2	606373	Intellectual developmental disorder, autosomal recessive 47, 616193 (3), Autosomal recessive
FMO3	136132	Trimethylaminuria, 602079 (3), Autosomal recessive
FMR1	309550	Fragile X tremor/ataxia syndrome, 300623 (3), X-linked dominant; Fragile X syndrome, 300624 (3), X-linked dominant; Premature ovarian failure 1, 311360 (3), X-linked
FN1	135600	Spondylometaphyseal dysplasia, corner fracture type, 184255 (3), Autosomal dominant; Glomerulopathy with fibronectin deposits 2, 601894 (3), Autosomal dominant
FNIP1	610594	Immunodeficiency 93 and hypertrophic cardiomyopathy, 619705 (3), Autosomal recessive
FOCAD	614606	Liver disease, severe congenital, 619991 (3), Autosomal recessive
FOLR1	136430	Neurodegeneration due to cerebral folate transport deficiency, 613068 (3), Autosomal recessive
FOXC1	601090	Axenfeld-Rieger syndrome, type 3, 602482 (3), Autosomal dominant; Anterior segment dysgenesis 3, multiple subtypes, 601631 (3), Autosomal dominant
FOXC2	602402	Lymphedema-distichiasis syndrome, 153400 (3), Autosomal dominant; Lymphedema-distichiasis syndrome with renal disease and diabetes mellitus, 153400 (3), Autosomal dominant
FOXD3	611539	{Autoimmune disease, susceptibility to, 1}, 607836 (3), Autosomal dominant
FOXE1	602617	Bamforth-Lazarus syndrome, 241850 (3), Autosomal recessive; {Thyroid cancer, nonmedullary, 4}, 616534 (3), Autosomal dominant
FOXE3	601094	Anterior segment dysgenesis 2, multiple subtypes, 610256 (3), Autosomal recessive; {Aortic aneurysm, familial thoracic 11, susceptibility to}, 617349 (3), Autosomal dominant; Cataract 34, multiple types, 612968 (3)
FOXF1	601089	Alveolar capillary dysplasia with misalignment of pulmonary veins, 265380 (3), Autosomal dominant
FOXG1	164874	Rett syndrome, congenital variant, 613454 (3), Autosomal dominant
FOXI1	601093	Enlarged vestibular aqueduct, 600791 (3), Autosomal recessive
FOXJ1	602291	Ciliary dyskinesia, primary, 43, 618699 (3), Autosomal dominant
FOXL2	605597	Blepharophimosis, epicanthus inversus, and ptosis, type 2, 110100 (3), Autosomal dominant, Autosomal recessive; Blepharophimosis, epicanthus inversus, and ptosis, type 1, 110100 (3), Autosomal dominant, Autosomal recessive; Premature ovarian failure 3, 608996 (3), Autosomal dominant
FOXN1	600838	T-cell lymphopenia, infantile, with or without nail dystrophy, autosomal dominant, 618806 (3), Autosomal dominant; T-cell immunodeficiency, congenital alopecia, and nail dystrophy, 601705 (3), Autosomal recessive
FOXO1	136533	Rhabdomyosarcoma, alveolar, 268220 (3), Somatic mutation

FOXP1	605515	Intellectual developmental disorder with language impairment with or without autistic features, 613670 (3), Autosomal dominant
FOXP2	605317	Speech-language disorder-1, 602081 (3), Autosomal dominant
FOXP3	300292	Immunodysregulation, polyendocrinopathy, and enteropathy, X-linked, 304790 (3), X-linked recessive
FOXRED1	613622	Mitochondrial complex I deficiency, nuclear type 19, 618241 (3), Autosomal recessive
FRAS1	607830	Fraser syndrome 1, 219000 (3), Autosomal recessive
FREM1	608944	Manitoba oculotrichoanal syndrome, 248450 (3), Autosomal recessive; Bifid nose with or without anorectal and renal anomalies, 608980 (3), Autosomal recessive; Trigonocephaly 2, 614485 (3), Autosomal dominant
FREM2	608945	Fraser syndrome 2, 617666 (3), Autosomal recessive; Cryptophthalmos, unilateral or bilateral, isolated, 123570 (3), Autosomal recessive
FRMD4A	616305	?Corpus callosum, agenesis of, with facial anomalies and cerebellar ataxia, 616819 (3), Autosomal recessive
FRMD7	300628	Nystagmus, infantile periodic alternating, X-linked, 310700 (3), X-linked; Nystagmus 1, congenital, X-linked, 310700 (3), X-linked
FRMPD4	300838	Intellectual developmental disorder, X-linked 104, 300983 (3), X-linked
FRRS1L	604574	Developmental and epileptic encephalopathy 37, 616981 (3), Autosomal recessive
FRZB	605083	{Osteoarthritis susceptibility 1}, 165720 (3), Multifactorial
FSCN2	607643	Retinitis pigmentosa 30, 607921 (3)
FSHB	136530	Hypogonadotropic hypogonadism 24 without anosmia, 229070 (3), Autosomal recessive
FSHR	136435	Ovarian response to FSH stimulation, 276400 (3), Autosomal recessive; Ovarian hyperstimulation syndrome, 608115 (3), Autosomal dominant; Ovarian dysgenesis 1, 233300 (3), Autosomal recessive
FSIP2	615796	Spermatogenic failure 34, 618153 (3), Autosomal recessive
FTCD	606806	Glutamate formiminotransferase deficiency, 229100 (3), Autosomal recessive
FTH1	134770	?Hemochromatosis, type 5, 615517 (3), Autosomal dominant
FTL	134790	Hyperferritinemia-cataract syndrome, 600886 (3), Autosomal dominant; L-ferritin deficiency, dominant and recessive, 615604 (3), Autosomal dominant, Autosomal recessive; Neurodegeneration with brain iron accumulation 3, 606159 (3), Autosomal dominant
FTO	610966	Growth retardation, developmental delay, facial dysmorphism, 612938 (3), Autosomal recessive; {Obesity, susceptibility to, BMIQ14}, 612460 (3), Autosomal recessive
FTSJ1	300499	Intellectual developmental disorder, X-linked 9, 309549 (3), X-linked recessive
FUCA1	612280	Fucosidosis, 230000 (3), Autosomal recessive
FUS	137070	Amyotrophic lateral sclerosis 6, with or without frontotemporal dementia, 608030 (3); Essential tremor, hereditary, 4, 614782 (3), Autosomal dominant
FUT1	211100	[Bombay phenotype], 616754 (3), Autosomal recessive

FUT2	182100	{Norwalk virus infection, resistance to} (3); {Vitamin B12 plasma level QTL1}, 612542 (3); [Bombay phenotype, digenic], 616754 (3), Autosomal recessive
FUT3	111100	[Blood group, Lewis], 618983 (3)
FUT6	136836	[Fucosyltransferase 6 deficiency], 613852 (3)
FUT8	602589	Congenital disorder of glycosylation with defective fucosylation 1, 618005 (3), Autosomal recessive
FUZ	610622	{Neural tube defects, susceptibility to}, 182940 (3), Autosomal dominant
FXN	606829	Friedreich ataxia with retained reflexes, 229300 (3), Autosomal recessive; Friedreich ataxia, 229300 (3), Autosomal recessive
FXR1	600819	?Myopathy, congenital, with respiratory insufficiency and bone fractures, 618822 (3), Autosomal recessive; ?Myopathy, congenital proximal, with minicore lesions, 618823 (3), Autosomal recessive
FXYD2	601814	Hypomagnesemia 2, renal, 154020 (3), Autosomal dominant
FYB1	602731	Thrombocytopenia 3, 273900 (3), Autosomal recessive
FYCO1	607182	Cataract 18, autosomal recessive, 610019 (3), Autosomal recessive
FZD2	600667	Omodysplasia 2, 164745 (3), Autosomal dominant
FZD4	604579	Retinopathy of prematurity, 133780 (3), Autosomal dominant; Exudative vitreoretinopathy 1, 133780 (3), Autosomal dominant
FZD5	601723	Autosomal Dominant Coloboma. PMID: 33633439, 26908622
FZD6	603409	Nail disorder, nonsyndromic congenital, 1, 161050 (3), Autosomal recessive
G6PC1	613742	Glycogen storage disease Ia, 232200 (3), Autosomal recessive
G6PC3	611045	Dursun syndrome, 612541 (3), Autosomal recessive; Neutropenia, severe congenital 4, autosomal recessive, 612541 (3), Autosomal recessive
G6PD	305900	Hemolytic anemia, G6PD deficient (favism), 300908 (3), X-linked dominant; {Resistance to malaria due to G6PD deficiency}, 611162 (3)
GAA	606800	Glycogen storage disease II, 232300 (3), Autosomal recessive
GAB1	604439	?Deafness, autosomal recessive 26, 605428 (3), Autosomal recessive
GABBR2	607340	{Nicotine dependence, protection against}, 188890 (3); {Nicotine dependence, susceptibility to}, 188890 (3); Developmental and epileptic encephalopathy 59, 617904 (3), Autosomal dominant; Neurodevelopmental disorder with poor language and loss of hand skills, 617903 (3), Autosomal dominant
GABRA1	137160	{Epilepsy, juvenile myoclonic, susceptibility to, 5}, 611136 (3); Developmental and epileptic encephalopathy 19, 615744 (3), Autosomal dominant; {Epilepsy, childhood absence, susceptibility to, 4}, 611136 (3)
GABRA2	137140	Developmental and epileptic encephalopathy 78, 618557 (3), Autosomal dominant; {Alcohol dependence, susceptibility to}, 103780 (3), Multifactorial
GABRA5	137142	Developmental and epileptic encephalopathy 79, 618559 (3), Autosomal dominant
GABRB1	137190	Developmental and epileptic encephalopathy 45, 617153 (3), Autosomal dominant

GABRB2	600232	Developmental and epileptic encephalopathy 92, 617829 (3), Autosomal dominant
GABRB3	137192	{Epilepsy, childhood absence, susceptibility to, 5}, 612269 (3); Developmental and epileptic encephalopathy 43, 617113 (3), Autosomal dominant
GABRD	137163	{Epilepsy, idiopathic generalized, 10}, 613060 (3), Autosomal dominant; {Epilepsy, juvenile myoclonic, susceptibility to}, 613060 (3), Autosomal dominant; {Generalized epilepsy with febrile seizures plus, type 5, susceptibility to}, 613060 (3), Autosomal dominant
GABRG2	137164	Developmental and epileptic encephalopathy 74, 618396 (3), Autosomal dominant; Febrile seizures, familial, 8, 607681 (3), Autosomal dominant; Generalized epilepsy with febrile seizures plus, type 3, 607681 (3), Autosomal dominant
GAD1	605363	Developmental and epileptic encephalopathy 89, 619124 (3), Autosomal recessive
GAL	137035	?Epilepsy, familial temporal lobe, 8, 616461 (3), Autosomal dominant
GALC	606890	Krabbe disease, 245200 (3), Autosomal recessive
GALE	606953	Galactose epimerase deficiency, 230350 (3), Autosomal recessive
GALK1	604313	Galactokinase deficiency with cataracts, 230200 (3), Autosomal recessive
GALM	137030	Galactosemia IV, 618881 (3), Autosomal recessive
GALNS	612222	Mucopolysaccharidosis IVA, 253000 (3), Autosomal recessive
GALNT12	610290	{Colorectal cancer, susceptibility to, 1}, 608812 (3)
GALNT2	602274	Congenital disorder of glycosylation, type IIt, 618885 (3), Autosomal recessive
GALNT3	601756	Tumoral calcinosis, hyperphosphatemic, familial, 1, 211900 (3), Autosomal recessive
GALT	606999	Galactosemia, 230400 (3), Autosomal recessive
GAMT	601240	Cerebral creatine deficiency syndrome 2, 612736 (3), Autosomal recessive
GAN	605379	Giant axonal neuropathy-1, 256850 (3), Autosomal recessive
GANAB	104160	Polycystic kidney disease 3, 600666 (3), Autosomal dominant
GARS1	600287	Spinal muscular atrophy, infantile, James type, 619042 (3), Autosomal dominant; Neuronopathy, distal hereditary motor, type VA, 600794 (3), Autosomal dominant; Charcot-Marie-Tooth disease, type 2D, 601472 (3), Autosomal dominant
GAS2L2	611398	?Ciliary dyskinesia, primary, 41, 618449 (3), Autosomal recessive
GAS8	605178	Ciliary dyskinesia, primary, 33, 616726 (3), Autosomal recessive
GATA1	305371	Leukemia, megakaryoblastic, with or without Down syndrome, somatic, 190685 (3); Thrombocytopenia, X-linked, with or without dyserythropoietic anemia, 300367 (3), X-linked recessive; Anemia, X-linked, with/without neutropenia and/or platelet abnormalities, 300835 (3), X-linked recessive; Thrombocytopenia with beta-thalassemia, X-linked, 314050 (3), X-linked recessive; Hemolytic anemia due to elevated adenosine deaminase, 301083 (3), X-linked recessive

GATA2	137295	{Leukemia, acute myeloid, susceptibility to}, 601626 (3), Autosomal dominant, Somatic mutation; Emberger syndrome, 614038 (3), Autosomal dominant; Immunodeficiency 21, 614172 (3), Autosomal dominant; {Myelodysplastic syndrome, susceptibility to}, 614286 (3)
GATA3	131320	Hypoparathyroidism, sensorineural deafness, and renal dysplasia, 146255 (3), Autosomal dominant
GATA4	600576	Tetralogy of Fallot, 187500 (3), Autosomal dominant; Atrial septal defect 2, 607941 (3), Autosomal dominant; Ventricular septal defect 1, 614429 (3), Autosomal dominant; Atrioventricular septal defect 4, 614430 (3), Autosomal dominant; ?Testicular anomalies with or without congenital heart disease, 615542 (3), Autosomal dominant
GATA5	611496	Congenital heart defects, multiple types, 5, 617912 (3), Autosomal dominant, Autosomal recessive
GATA6	601656	Atrial septal defect 9, 614475 (3), Autosomal dominant; Persistent truncus arteriosus, 217095 (3); Pancreatic agenesis and congenital heart defects, 600001 (3), Autosomal dominant; Atrioventricular septal defect 5, 614474 (3), Autosomal dominant; Tetralogy of Fallot, 187500 (3), Autosomal dominant
GATAD1	614518	?Cardiomyopathy, dilated, 2B, 614672 (3), Autosomal recessive
GATAD2B	614998	GAND syndrome, 615074 (3), Autosomal dominant
GATB	603645	?Combined oxidative phosphorylation deficiency 41, 618838 (3), Autosomal recessive
GATC	617210	Combined oxidative phosphorylation deficiency 42, 618839 (3), Autosomal recessive
GATM	602360	Cerebral creatine deficiency syndrome 3, 612718 (3), Autosomal recessive; Fanconi renotubular syndrome 1, 134600 (3), Autosomal dominant
GBA1 (GBA)	606463	{Lewy body dementia, susceptibility to}, 127750 (3), Autosomal dominant; Gaucher disease, type II, 230900 (3), Autosomal recessive; Gaucher disease, type IIIC, 231005 (3), Autosomal recessive; Gaucher disease, type III, 231000 (3), Autosomal recessive; Gaucher disease, type I, 230800 (3), Autosomal recessive; Gaucher disease, perinatal lethal, 608013 (3), Autosomal recessive; {Parkinson disease, late-onset, susceptibility to}, 168600 (3), Multifactorial, Autosomal dominant
GBA2	609471	Spastic paraplegia 46, autosomal recessive, 614409 (3), Autosomal recessive
GBE1	607839	Glycogen storage disease IV, 232500 (3), Autosomal recessive; Polyglucosan body disease, adult form, 263570 (3), Autosomal recessive
GBF1	603698	Charcot-Marie-Tooth disease, axonal, type 2GG, 606483 (3), Autosomal dominant
GCDH	608801	Glutaricaciduria, type I, 231670 (3), Autosomal recessive
GCGR	138033	Mahvash disease, 619290 (3), Autosomal recessive
GCH1	600225	Dystonia, DOPA-responsive, with or without hyperphenylalaninemia, 128230 (3), Autosomal dominant, Autosomal recessive; Hyperphenylalaninemia, BH4-deficient, B, 233910 (3), Autosomal recessive

GCK	138079	MODY, type II, 125851 (3), Autosomal dominant; Diabetes mellitus, permanent neonatal 1, 606176 (3), Autosomal recessive; Hyperinsulinemic hypoglycemia, familial, 3, 602485 (3), Autosomal dominant; Diabetes mellitus, noninsulin-dependent, late onset, 125853 (3), Autosomal dominant
GCKR	600842	[Fasting plasma glucose level QTL 5], 613463 (3)
GCLC	606857	{Myocardial infarction, susceptibility to}, 608446 (3); Hemolytic anemia due to gamma-glutamylcysteine synthetase deficiency, 230450 (3), Autosomal recessive
GCLM	601176	{Myocardial infarction, susceptibility to}, 608446 (3)
GCM2	603716	Hypoparathyroidism, familial isolated 2, 618883 (3), Autosomal dominant, Autosomal recessive; Hyperparathyroidism 4, 617343 (3), Autosomal dominant
GCNA	300369	Spermatogenic failure, X-linked, 4, 301077 (3), X-linked
GCNT2	600429	[Blood group, Ii], 110800 (3), Autosomal dominant; Adult i phenotype without cataract, 110800 (3), Autosomal dominant; Cataract 13 with adult i phenotype, 116700 (3), Autosomal recessive
GCSH	238330	?Glycine encephalopathy, 605899 (3), Autosomal recessive
GDAP1	606598	Charcot-Marie-Tooth disease, axonal, with vocal cord paresis, 607706 (3), Autosomal recessive; Charcot-Marie-Tooth disease, recessive intermediate, A, 608340 (3), Autosomal recessive; Charcot-Marie-Tooth disease, axonal, type 2K, 607831 (3), Autosomal dominant, Autosomal recessive; Charcot-Marie-Tooth disease, type 4A, 214400 (3), Autosomal recessive
GDAP2	618128	Spinocerebellar ataxia, autosomal recessive 27, 618369 (3), Autosomal recessive
GDF1	602880	Congenital heart defects, multiple types, 6, 613854 (3), Autosomal dominant; Right atrial isomerism (Ivemark), 208530 (3), Autosomal recessive
GDF11	603936	?Vertebral hypersegmentation and orofacial anomalies, 619122 (3), Autosomal dominant
GDF2	605120	Telangiectasia, hereditary hemorrhagic, type 5, 615506 (3), Autosomal dominant
GDF3	606522	Klippel-Feil syndrome 3, autosomal dominant, 613702 (3); Microphthalmia, isolated, with coloboma 6, 613703 (3), Autosomal dominant; Microphthalmia, isolated 7, 613704 (3), Autosomal dominant
GDF5	601146	Acromesomelic dysplasia 2A, 200700 (3), Autosomal recessive; Acromesomelic dysplasia 2B, 228900 (3), Autosomal recessive; Multiple synostoses syndrome 2, 610017 (3), Autosomal dominant; Symphalangism, proximal, 1B, 615298 (3), Autosomal dominant; Brachydactyly, type A2, 112600 (3), Autosomal dominant; ?Acromesomelic dysplasia 2C, Hunter-Thompson type, 201250 (3), Autosomal recessive; Brachydactyly, type C, 113100 (3), Autosomal dominant; {Osteoarthritis-5}, 612400 (3); Brachydactyly, type A1, C, 615072 (3), Autosomal dominant, Autosomal recessive

GDF6	601147	Microphthalmia with coloboma 6, digenic, 613703 (3), Autosomal dominant; Microphthalmia, isolated 4, 613094 (3); Leber congenital amaurosis 17, 615360 (3), Autosomal recessive; Multiple synostoses syndrome 4, 617898 (3), Autosomal dominant; Klippel-Feil syndrome 1, autosomal dominant, 118100 (3), Autosomal dominant
GDF9	601918	?Premature ovarian failure 14, 618014 (3), Autosomal recessive
GDI1	300104	Intellectual developmental disorder, X-linked 41, 300849 (3), X-linked dominant
GDNF	600837	{Hirschsprung disease, susceptibility to, 3}, 613711 (3), Autosomal dominant
GEMIN4	606969	Neurodevelopmental disorder with microcephaly, cataracts, and renal abnormalities, 617913 (3), Autosomal recessive
GEMIN5	607005	Neurodevelopmental disorder with cerebellar atrophy and motor dysfunction, 619333 (3), Autosomal recessive
GFAP	137780	Alexander disease, 203450 (3), Autosomal dominant
GFER	600924	Myopathy, mitochondrial progressive, with congenital cataract and developmental delay, 613076 (3), Autosomal recessive
GFI1	600871	?Neutropenia, nonimmune chronic idiopathic, of adults, 607847 (3), Autosomal dominant; Neutropenia, severe congenital 2, autosomal dominant, 613107 (3), Autosomal dominant
GFI1B	604383	Bleeding disorder, platelet-type, 17, 187900 (3), Autosomal dominant, Autosomal recessive
GFM1	606639	Combined oxidative phosphorylation deficiency 1, 609060 (3), Autosomal recessive
GFM2	606544	Combined oxidative phosphorylation deficiency 39, 618397 (3), Autosomal recessive
GFPT1	138292	Myasthenia, congenital, 12, with tubular aggregates, 610542 (3), Autosomal recessive
GFRA1	601496	Renal hypodysplasia/aplasia 4, 619887 (3), Autosomal recessive
GGCX	137167	Vitamin K-dependent clotting factors, combined deficiency of, 1, 277450 (3), Autosomal recessive; Pseudoxanthoma elasticum-like disorder with multiple coagulation factor deficiency, 610842 (3)
GGN	609966	Spermatogenic failure 69, 619826 (3), Autosomal recessive
GGPS1	606982	Muscular dystrophy, congenital hearing loss, and ovarian insufficiency syndrome, 619518 (3), Autosomal recessive
GGT1	612346	?Glutathioninuria, 231950 (3), Autosomal recessive
GGT2P (GGT2)	137181	[Gamma-glutamyltransferase, familial high serum], 137181 (2)
GH1	139250	Kowarski syndrome, 262650 (3), Autosomal recessive; Growth hormone deficiency, isolated, type II, 173100 (3), Autosomal dominant; Growth hormone deficiency, isolated, type IB, 612781 (3); Growth hormone deficiency, isolated, type IA, 262400 (3), Autosomal recessive

GHR	600946	Laron dwarfism, 262500 (3), Autosomal recessive; Increased responsiveness to growth hormone, 604271 (3), Autosomal dominant; Growth hormone insensitivity, partial, 604271 (3), Autosomal dominant; {Hypercholesterolemia, familial, modifier of}, 143890 (3), Autosomal dominant, Autosomal recessive
GHRH	139190	Gigantism due to GHRF hypersecretion (1); ?Isolated growth hormone deficiency due to defect in GHRF (1)
GHRHR	139191	Growth hormone deficiency, isolated, type IV, 618157 (3), Autosomal recessive
GHRL	605353	{Obesity, susceptibility to}, 601665 (3), Multifactorial, Autosomal dominant, Autosomal recessive
GHSR	601898	Growth hormone deficiency, isolated partial, 615925 (3), Autosomal dominant, Autosomal recessive
GIGYF2	612003	{Parkinson disease 11}, 607688 (3)
GIMAP5	608086	Portal hypertension, noncirrhotic, 2, 619463 (3), Autosomal recessive
GINS1	610608	Immunodeficiency 55, 617827 (3), Autosomal recessive
GIPC1	605072	Oculopharyngodistal myopathy 2, 618940 (3), Autosomal dominant
GIPC3	608792	Deafness, autosomal recessive 15, 601869 (3), Autosomal recessive
GJA1	121014	Erythrokeratodermia variabilis et progressiva 3, 617525 (3), Autosomal dominant; Craniometaphyseal dysplasia, autosomal recessive, 218400 (3), Autosomal recessive; Oculodentodigital dysplasia, 164200 (3), Autosomal dominant; Hypoplastic left heart syndrome 1, 241550 (3), Autosomal recessive; Palmoplantar keratoderma with congenital alopecia, 104100 (3), Autosomal dominant; Syndactyly, type III, 186100 (3), Autosomal dominant; Oculodentodigital dysplasia, autosomal recessive, 257850 (3), Autosomal recessive; Atrioventricular septal defect 3, 600309 (3), Autosomal dominant
GJA3	121015	Cataract 14, multiple types, 601885 (3), Autosomal dominant
GJA5	121013	Atrial fibrillation, familial, 11, 614049 (3), Autosomal dominant; Atrial standstill, digenic (GJA5/SCN5A), 108770 (3), Autosomal dominant
GJA8	600897	Cataract 1, multiple types, 116200 (3), Autosomal dominant
GJB1	304040	Charcot-Marie-Tooth neuropathy, X-linked dominant, 1, 302800 (3), X-linked dominant
GJB2	121011	Keratoderma, palmoplantar, with deafness, 148350 (3), Autosomal dominant; Deafness, autosomal recessive 1A, 220290 (3), Digenic dominant, Autosomal recessive; Deafness, autosomal dominant 3A, 601544 (3), Autosomal dominant; Hystrix-like ichthyosis with deafness, 602540 (3), Autosomal dominant; Bart-Pumphrey syndrome, 149200 (3), Autosomal dominant; Keratitis-ichthyosis-deafness syndrome, 148210 (3), Autosomal dominant; Vohwinkel syndrome, 124500 (3), Autosomal dominant

GJB3	603324	Deafness, digenic, GJB2/GJB3, 220290 (3), Digenic dominant, Autosomal recessive; Deafness, autosomal recessive (3); Deafness, autosomal dominant 2B, 612644 (3), Autosomal dominant; Erythrokeratodermia variabilis et progressiva 1, 133200 (3), Autosomal dominant, Autosomal recessive; Deafness, autosomal dominant, with peripheral neuropathy (3)
GJB4	605425	Erythrokeratodermia variabilis et progressiva 2, 617524 (3), Autosomal dominant
GJB6	604418	Ectodermal dysplasia 2, Clouston type, 129500 (3), Autosomal dominant; Deafness, autosomal dominant 3B, 612643 (3), Autosomal dominant; Deafness, autosomal recessive 1B, 612645 (3), Autosomal recessive; Deafness, digenic GJB2/GJB6, 220290 (3), Digenic dominant, Autosomal recessive
GJC2	608803	Lymphatic malformation 3, 613480 (3), Autosomal dominant; ?Spastic paraplegia 44, autosomal recessive, 613206 (3), Autosomal recessive; Leukodystrophy, hypomyelinating, 2, 608804 (3), Autosomal recessive
GK	300474	Glycerol kinase deficiency, 307030 (3), X-linked recessive
GLA	300644	Fabry disease, cardiac variant, 301500 (3), X-linked; Fabry disease, 301500 (3), X-linked
GLB1	611458	GM1-gangliosidosis, type I, 230500 (3), Autosomal recessive; GM1-gangliosidosis, type III, 230650 (3), Autosomal recessive; Mucopolysaccharidosis type IVB (Morquio), 253010 (3), Autosomal recessive; GM1-gangliosidosis, type II, 230600 (3), Autosomal recessive
GLCCI1	614283	{Glucocorticoid therapy, response to}, 614400 (3)
GLDC	238300	Glycine encephalopathy, 605899 (3), Autosomal recessive
GLDN	608603	Lethal congenital contracture syndrome 11, 617194 (3), Autosomal recessive
GLE1	603371	Lethal congenital contracture syndrome 1, 253310 (3), Autosomal recessive; Congenital arthrogryposis with anterior horn cell disease, 611890 (3), Autosomal recessive
GLI1	165220	Polydactyly, preaxial I, 174400 (3), Autosomal recessive; Polydactyly, postaxial, type A8, 618123 (3), Autosomal recessive
GLI2	165230	Culler-Jones syndrome, 615849 (3), Autosomal dominant; Holoprosencephaly 9, 610829 (3), Autosomal dominant
GLI3	165240	Greig cephalopolysyndactyly syndrome, 175700 (3), Autosomal dominant; Polydactyly, postaxial, types A1 and B, 174200 (3), Autosomal dominant; Pallister-Hall syndrome, 146510 (3), Autosomal dominant; Polydactyly, preaxial, type IV, 174700 (3), Autosomal dominant
GLIS2	608539	Nephronophthisis 7, 611498 (3)
GLIS3	610192	Diabetes mellitus, neonatal, with congenital hypothyroidism, 610199 (3), Autosomal recessive
GLMN	601749	Glomuvenous malformations, 138000 (3), Autosomal dominant
GLRA1	138491	Hyperekplexia 1, 149400 (3), Autosomal dominant, Autosomal recessive
GLRA2	305990	Intellectual developmental disorder, X-linked syndromic, Pilorge type, 301076 (3), X-linked

GLRB	138492	Hyperekplexia 2, 614619 (3), Autosomal recessive
GLRX5	609588	Anemia, sideroblastic, 3, pyridoxine-refractory, 616860 (3), Autosomal recessive; Spasticity, childhood-onset, with hyperglycinemia, 616859 (3), Autosomal recessive
GLS	138280	Global developmental delay, progressive ataxia, and elevated glutamine, 618412 (3), Autosomal recessive; ?Infantile cataract, skin abnormalities, glutamate excess, and impaired intellectual development, 618339 (3), Autosomal dominant; Developmental and epileptic encephalopathy 71, 618328 (3), Autosomal recessive
GLUD1	138130	Hyperinsulinism-hyperammonemia syndrome, 606762 (3), Autosomal dominant
GLUD2	300144	{Parkinson disease, age of onset, modifier}, 168600 (3), Multifactorial, Autosomal dominant
GLUL	138290	Glutamine deficiency, congenital, 610015 (3), Autosomal recessive
GLYCTK	610516	D-glyceric aciduria, 220120 (3), Autosomal recessive
GM2A	613109	GM2-gangliosidosis, AB variant, 272750 (3), Autosomal recessive
GMNN	602842	Meier-Gorlin syndrome 6, 616835 (3), Autosomal dominant
GMPPA	615495	Alacrima, achalasia, and mental retardation syndrome, 615510 (3), Autosomal recessive
GMPPB	615320	Muscular dystrophy-dystroglycanopathy (limb-girdle), type C, 14, 615352 (3), Autosomal recessive; Muscular dystrophy-dystroglycanopathy (congenital with mental retardation), type B, 14, 615351 (3), Autosomal recessive; Muscular dystrophy-dystroglycanopathy (congenital with brain and eye anomalies), type A, 14, 615350 (3), Autosomal recessive
GNA11	139313	Hypocalciuric hypercalcemia, type II, 145981 (3), Autosomal dominant; Hypocalcemia, autosomal dominant 2, 615361 (3), Autosomal dominant
GNAI1	139310	Neurodevelopmental disorder with hypotonia, impaired speech, and behavioral abnormalities, 619854 (3), Autosomal dominant
GNAI2	139360	Ventricular tachycardia, idiopathic, 192605 (3), Autosomal dominant; Pituitary adenoma, ACTH-secreting, somatic (3)
GNAI3	139370	Auriculocondylar syndrome 1, 602483 (3), Autosomal dominant
GNAL	139312	Dystonia 25, 615073 (3), Autosomal dominant
GNAO1	139311	Developmental and epileptic encephalopathy 17, 615473 (3), Autosomal dominant; Neurodevelopmental disorder with involuntary movements, 617493 (3), Autosomal dominant
GNAQ	600998	Capillary malformations, congenital, 1, somatic, mosaic, 163000 (3); Sturge-Weber syndrome, somatic, mosaic, 185300 (3)

GNAS	139320	ACTH-independent macronodular adrenal hyperplasia, 219080 (3), Somatic mutation; Pituitary adenoma 3, multiple types, somatic, 617686 (3); Pseudohypoparathyroidism Ic, 612462 (3), Autosomal dominant; Pseudohypoparathyroidism Ia, 103580 (3), Autosomal dominant; Osseous heteroplasia, progressive, 166350 (3), Autosomal dominant; Pseudohypoparathyroidism Ib, 603233 (3), Autosomal dominant; McCune-Albright syndrome, somatic, mosaic, 174800 (3); Pseudopseudohypoparathyroidism, 612463 (3), Autosomal dominant
GNAS-AS1	610540	Pseudohypoparathyroidism, type IB, 603233 (3), Autosomal dominant
GNAT1	139330	Night blindness, congenital stationary, autosomal dominant 3, 610444 (3), Autosomal dominant; Night blindness, congenital stationary, type 1G, 616389 (3), Autosomal recessive
GNAT2	139340	Achromatopsia 4, 613856 (3)
GNB1	139380	Myelodysplastic syndrome, somatic, 614286 (3); Leukemia, acute lymphoblastic, somatic, 613065 (3); Intellectual developmental disorder, autosomal dominant 42, 616973 (3), Autosomal dominant
GNB2	139390	Neurodevelopmental disorder with hypotonia and dysmorphic facies, 619503 (3), Autosomal dominant; ?Sick sinus syndrome 4, 619464 (3), Autosomal dominant
GNB3	139130	Night blindness, congenital stationary, type 1H, 617024 (3), Autosomal recessive; {Hypertension, essential, susceptibility to}, 145500 (3), Multifactorial
GNB4	610863	Charcot-Marie-Tooth disease, dominant intermediate F, 615185 (3), Autosomal dominant
GNB5	604447	Language delay and ADHD/cognitive impairment with or without cardiac arrhythmia, 617182 (3), Autosomal recessive; Intellectual developmental disorder with cardiac arrhythmia, 617173 (3), Autosomal recessive
GNE	603824	Sialuria, 269921 (3), Autosomal dominant; Nonaka myopathy, 605820 (3), Autosomal recessive
GNMT	606628	Glycine N-methyltransferase deficiency, 606664 (3), Autosomal recessive
GNPAT	602744	Rhizomelic chondrodysplasia punctata, type 2, 222765 (3), Autosomal recessive
GNPNAT1	616510	?Rhizomelic dysplasia, Ain-Naz type, 616510 (3)
GNPTAB	607840	Mucolipidosis III alpha/beta, 252600 (3), Autosomal recessive; Mucolipidosis II alpha/beta, 252500 (3), Autosomal recessive
GNPTG	607838	Mucolipidosis III gamma, 252605 (3), Autosomal recessive
GNRH1	152760	?Hypogonadotropic hypogonadism 12 with or without anosmia, 614841 (3), Autosomal recessive
GNRHR	138850	Hypogonadotropic hypogonadism 7 without anosmia, 146110 (3), Autosomal recessive
GNS	607664	Mucopolysaccharidosis type IIID, 252940 (3), Autosomal recessive
GON7	617436	Galloway-Mowat syndrome 9, 619603 (3), Autosomal recessive
GORAB	607983	Geroderma osteodysplasticum, 231070 (3), Autosomal recessive
GOSR2	604027	Epilepsy, progressive myoclonic 6, 614018 (3), Autosomal recessive

GOT1	138180	Aspartate aminotransferase, serum level of, QTL1, 614419 (3)
GOT2	138150	Developmental and epileptic encephalopathy 82, 618721 (3), Autosomal recessive
GP1BA	606672	Bernard-Soulier syndrome, type A1 (recessive), 231200 (3), Autosomal recessive; Bernard-Soulier syndrome, type A2 (dominant), 153670 (3), Autosomal dominant; von Willebrand disease, platelet-type, 177820 (3), Autosomal dominant; {Nonarteritic anterior ischemic optic neuropathy, susceptibility to}, 258660 (3), Autosomal recessive
GP1BB	138720	Giant platelet disorder, isolated, 231200 (3), Autosomal recessive; Bernard-Soulier syndrome, type B, 231200 (3), Autosomal recessive
GP6	605546	Bleeding disorder, platelet-type, 11, 614201 (3), Autosomal recessive
GP9	173515	Bernard-Soulier syndrome, type C, 231200 (3), Autosomal recessive
GPAA1	603048	Glycosylphosphatidylinositol biosynthesis defect 15, 617810 (3), Autosomal recessive
GPC3	300037	Wilms tumor, somatic, 194070 (3); Simpson-Golabi-Behmel syndrome, type 1, 312870 (3), X-linked recessive
GPC4	300168	Keipert syndrome, 301026 (3), X-linked recessive
GPC6	604404	Omodysplasia 1, 258315 (3), Autosomal recessive
GPD1	138420	Hypertriglyceridemia, transient infantile, 614480 (3), Autosomal recessive
GPD1L	611778	Brugada syndrome 2, 611777 (3)
GPD2	138430	{Type 2 diabetes mellitus, susceptibility to}, 125853 (3), Autosomal dominant
GPHN	603930	Molybdenum cofactor deficiency C, 615501 (3), Autosomal recessive
GPI	172400	Hemolytic anemia, nonspherocytic, due to glucose phosphate isomerase deficiency, 613470 (3), Autosomal recessive
GPIHBP1	612757	Hyperlipoproteinemia, type 1D, 615947 (3), Autosomal recessive
GPNMB	604368	Amyloidosis, primary localized cutaneous, 3, 617920 (3), Autosomal recessive
GPR101	300393	Pituitary adenoma 2, GH-secreting, 300943 (3), X-linked
GPR143	300808	Ocular albinism, type I, Nettleship-Falls type, 300500 (3), X-linked; Nystagmus 6, congenital, X-linked, 300814 (3), X-linked recessive
GPR179	614515	Night blindness, congenital stationary (complete), 1E, autosomal recessive, 614565 (3), Autosomal recessive
GPR68	601404	Amelogenesis imperfecta, hypomaturation type, IIA6, 617217 (3), Autosomal recessive
GPR88	607468	?Chorea, childhood-onset, with psychomotor retardation, 616939 (3), Autosomal recessive
GPRASP2	300969	?Deafness, X-linked 7, 301018 (3), X-linked recessive
GPSM2	609245	Chudley-McCullough syndrome, 604213 (3), Autosomal recessive
GPT2	138210	Neurodevelopmental disorder with microcephaly and spastic paraplegia, 616281 (3), Autosomal recessive
GPX1	138320	Hemolytic anemia due to glutathione peroxidase deficiency, 614164 (1), Autosomal recessive

GPX4	138322	Spondylometaphyseal dysplasia, Sedaghatian type, 250220 (3), Autosomal recessive
GRAP	604330	Deafness, autosomal recessive 114, 618456 (3), Autosomal recessive
GREB1L	617782	Deafness, autosomal dominant 80, 619274 (3), Autosomal dominant; Renal hypodysplasia/aplasia 3, 617805 (3), Autosomal dominant
GREM2	608832	Tooth agenesis, selective, 9, 617275 (3), Autosomal dominant
GRHL2	608576	Deafness, autosomal dominant 28, 608641 (3), Autosomal dominant; Ectodermal dysplasia/short stature syndrome, 616029 (3), Autosomal recessive; Corneal dystrophy, posterior polymorphous, 4, 618031 (3), Autosomal dominant
GRHL3	608317	van der Woude syndrome 2, 606713 (3), Autosomal dominant
GRHPR	604296	Hyperoxaluria, primary, type II, 260000 (3), Autosomal recessive
GRIA1	138248	?Intellectual developmental disorder, autosomal recessive 76, 619931 (3), Autosomal recessive; Intellectual developmental disorder, autosomal dominant 67, 619927 (3), Autosomal dominant
GRIA2	138247	Neurodevelopmental disorder with language impairment and behavioral abnormalities, 618917 (3), Autosomal dominant
GRIA3	305915	Intellectual developmental disorder, X-linked syndromic, Wu type, 300699 (3), X-linked recessive
GRIA4	138246	Neurodevelopmental disorder with or without seizures and gait abnormalities, 617864 (3), Autosomal dominant
GRID2	602368	Spinocerebellar ataxia, autosomal recessive 18, 616204 (3), Autosomal recessive
GRIK2	138244	Neurodevelopmental disorder with impaired language and ataxia and with or without seizures, 619580 (3), Autosomal dominant; Intellectual developmental disorder, autosomal recessive 6, 611092 (3), Autosomal recessive
GRIN1	138249	Neurodevelopmental disorder with or without hyperkinetic movements and seizures, autosomal recessive, 617820 (3), Autosomal recessive; Developmental and epileptic encephalopathy 101, 619814 (3), Autosomal recessive; Neurodevelopmental disorder with or without hyperkinetic movements and seizures, autosomal dominant, 614254 (3), Autosomal dominant
GRIN2A	138253	Epilepsy, focal, with speech disorder and with or without impaired intellectual development, 245570 (3), Autosomal dominant
GRIN2B	138252	Developmental and epileptic encephalopathy 27, 616139 (3), Autosomal dominant; Intellectual developmental disorder, autosomal dominant 6, with or without seizures, 613970 (3), Autosomal dominant
GRIN2D	602717	Developmental and epileptic encephalopathy 46, 617162 (3), Autosomal dominant
GRIP1	604597	Fraser syndrome 3, 617667 (3), Autosomal recessive
GRK1	180381	Oguchi disease-2, 613411 (3)
GRM1	604473	Spinocerebellar ataxia, autosomal recessive 13, 614831 (3), Autosomal recessive; Spinocerebellar ataxia 44, 617691 (3), Autosomal dominant

GRM6	604096	Night blindness, congenital stationary (complete), 1B, autosomal recessive, 257270 (3), Autosomal recessive
GRM7	604101	Neurodevelopmental disorder with seizures, hypotonia, and brain abnormalities, 618922 (3), Autosomal recessive
GRN	138945	Aphasia, primary progressive, 607485 (3), Autosomal dominant; Frontotemporal lobar degeneration with ubiquitin-positive inclusions, 607485 (3), Autosomal dominant; Ceroid lipofuscinosis, neuronal, 11, 614706 (3), Autosomal recessive
GRXCR1	613283	Deafness, autosomal recessive 25, 613285 (3), Autosomal recessive
GRXCR2	615762	?Deafness, autosomal recessive 101, 615837 (3), Autosomal recessive
GSC	138890	Short stature, auditory canal atresia, mandibular hypoplasia, skeletal abnormalities, 602471 (3), Autosomal recessive
GSDME	608798	Deafness, autosomal dominant 5, 600994 (3), Autosomal dominant
GSN	137350	Amyloidosis, Finnish type, 105120 (3), Autosomal dominant
GSR	138300	Hemolytic anemia due to glutathione reductase deficiency, 618660 (3), Autosomal recessive
GSS	601002	Hemolytic anemia due to glutathione synthetase deficiency, 231900 (3), Autosomal recessive; Glutathione synthetase deficiency, 266130 (3), Autosomal recessive
GSTZ1	603758	[Maleylacetoacetate isomerase deficiency], 617596 (3), Autosomal recessive
GSX2	616253	Diencephalic-mesencephalic junction dysplasia syndrome 2, 618646 (3), Autosomal recessive
GTF2E2	189964	Trichothiodystrophy 6, nonphotosensitive, 616943 (3), Autosomal recessive
GTF2H5	608780	Trichothiodystrophy 3, photosensitive, 616395 (3), Autosomal recessive
GTPBP2	607434	Jaberi-Elahi syndrome, 617988 (3), Autosomal recessive
GTPBP3	608536	Combined oxidative phosphorylation deficiency 23, 616198 (3), Autosomal recessive
GUCA1A	600364	Cone-rod dystrophy 14, 602093 (3), Autosomal dominant; Cone dystrophy-3, 602093 (3), Autosomal dominant
GUCA1B	602275	Retinitis pigmentosa 48, 613827 (3)
GUCY1A1	139396	Moyamoya 6 with achalasia, 615750 (3), Autosomal recessive
GUCY2C	601330	Diarrhea 6, 614616 (3), Autosomal dominant; Meconium ileus, 614665 (3), Autosomal recessive
GUCY2D	600179	Cone-rod dystrophy 6, 601777 (3), Autosomal dominant, Autosomal recessive; ?Choroidal dystrophy, central areolar 1, 215500 (3), Autosomal dominant; Leber congenital amaurosis 1, 204000 (3), Autosomal recessive; Night blindness, congenital stationary, type 1I, 618555 (3), Autosomal recessive
GUF1	617064	?Developmental and epileptic encephalopathy 40, 617065 (3), Autosomal recessive
GUSB	611499	Mucopolysaccharidosis VII, 253220 (3), Autosomal recessive

GYG1	603942	?Glycogen storage disease XV, 613507 (3), Autosomal recessive; Polyglucosan body myopathy 2, 616199 (3), Autosomal recessive
GYPA	617922	{Malaria, resistance to}, 611162 (3); [Blood group, MNSs system], 111300 (3)
GYPB	617923	[Blood group, Ss], 111740 (3); {Malaria, resistance to}, 611162 (3)
GYPC	110750	[Blood group, Gerbich], 616089 (3); {Malaria, resistance to}, 611162 (3)
GYS1	138570	Glycogen storage disease 0, muscle, 611556 (3), Autosomal recessive
GYS2	138571	Glycogen storage disease 0, liver, 240600 (3), Autosomal recessive
GZF1	613842	Joint laxity, short stature, and myopia, 617662 (3), Autosomal recessive
H1-4	142220	Rahman syndrome, 617537 (3), Autosomal dominant
H3-3A	601128	Bryant-Li-Bhoj neurodevelopmental syndrome 1, 619720 (3), Autosomal dominant
H3-3B	601058	Bryant-Li-Bhoj neurodevelopmental syndrome 2, 619721 (3), Autosomal dominant
H4C11	602826	?Tessadori-van Haaften neurodevelopmental syndrome 2, 619759 (3), Autosomal dominant
H4C3	602827	Tessadori-van Haaften neurodevelopmental syndrome 1, 619758 (3), Autosomal dominant
H4C5	602830	Tessadori-van Haaften neurodevelopmental syndrome 3, 619950 (3), Autosomal dominant
H4C9	602833	Tessadori-van Haaften neurodevelopmental syndrome 4, 619951 (3), Autosomal dominant
H6PD	138090	Cortisone reductase deficiency 1, 604931 (3), Autosomal recessive
HAAO	604521	Vertebral, cardiac, renal, and limb defects syndrome 1, 617660 (3), Autosomal recessive
HABP2	603924	{?Thyroid cancer, nonmedullary, 5}, 616535 (3), Autosomal dominant; {Venous thromboembolism, susceptibility to}, 188050 (3), Autosomal dominant
HACD1	610467	Myopathy, congenital, nonprogressive, 619967 (3), Autosomal recessive
HACE1	610876	Spastic paraplegia and psychomotor retardation with or without seizures, 616756 (3), Autosomal recessive
HADH	601609	Hyperinsulinemic hypoglycemia, familial, 4, 609975 (3), Autosomal recessive; 3-hydroxyacyl-CoA dehydrogenase deficiency, 231530 (3), Autosomal recessive
HADHA	600890	HELLP syndrome, maternal, of pregnancy, 609016 (3), Autosomal recessive; Mitochondrial trifunctional protein deficiency, 609015 (3), Autosomal recessive; LCHAD deficiency, 609016 (3), Autosomal recessive; Fatty liver, acute, of pregnancy, 609016 (3), Autosomal recessive
HADHB	143450	Trifunctional protein deficiency, 609015 (3), Autosomal recessive
HAGH	138760	[Glyoxalase II deficiency], 614033 (1), Autosomal dominant
HAL	609457	[Histidinemia], 235800 (3), Autosomal dominant, Autosomal recessive
HAMP	606464	Hemochromatosis, type 2B, 613313 (3), Autosomal recessive
HARS1	142810	Charcot-Marie-Tooth disease, axonal, type 2W, 616625 (3), Autosomal dominant; Usher syndrome type 3B, 614504 (3), Autosomal recessive

HARS2	600783	Perrault syndrome 2, 614926 (3), Autosomal recessive
HAVCR2	606652	T-cell lymphoma, subcutaneous panniculitis-like, 618398 (3), Autosomal recessive
HAX1	605998	Neutropenia, severe congenital 3, autosomal recessive, 610738 (3), Autosomal recessive
HBA1	141800	Hemoglobin H disease, nondeletional, 613978 (3); Thalassemias, alpha-, 604131 (3); Heinz body anemias, alpha-, 140700 (3), Autosomal dominant; Methemoglobinemia, alpha type, 617973 (3), Autosomal dominant; Erythrocytosis 7, 617981 (3), Autosomal dominant
HBA2	141850	Heinz body anemia, 140700 (3), Autosomal dominant; Erythrocytosis 7, 617981 (3), Autosomal dominant; Thalassemia, alpha-, 604131 (3); Hemoglobin H disease, deletional and nondeletional, 613978 (3)
HBB	141900	Methemoglobinemia, beta type, 617971 (3), Autosomal dominant; Thalassemia-beta, dominant inclusion-body, 603902 (3), Autosomal dominant; Sickle cell anemia, 603903 (3), Autosomal recessive; Thalassemia, beta, 613985 (3); Delta-beta thalassemia, 141749 (3), Autosomal dominant; {Malaria, resistance to}, 611162 (3); Hereditary persistence of fetal hemoglobin, 141749 (3), Autosomal dominant; Heinz body anemia, 140700 (3), Autosomal dominant; Erythrocytosis 6, 617980 (3), Autosomal dominant
HBD	142000	Thalassemia due to Hb Lepore (3); Thalassemia, delta- (3)
HBEGF	126150	{Diphtheria, susceptibility to} (1)
HBG1	142200	Fetal hemoglobin quantitative trait locus 1, 141749 (3), Autosomal dominant
HBG2	142250	Fetal hemoglobin quantitative trait locus 1, 141749 (3), Autosomal dominant; Cyanosis, transient neonatal, 613977 (3), Autosomal dominant
HCCS	300056	Linear skin defects with multiple congenital anomalies 1, 309801 (3), X-linked dominant
HCFC1	300019	Methylmalonic aciduria and homocysteinemia, cblX type, 309541 (3), X-linked recessive
HCN1	602780	Developmental and epileptic encephalopathy 24, 615871 (3), Autosomal dominant; Generalized epilepsy with febrile seizures plus, type 10, 618482 (3), Autosomal dominant
HCN2	602781	Febrile seizures, familial, 2, 602477 (3), Autosomal dominant; {Epilepsy, idiopathic generalized, susceptibility to, 17}, 602477 (3), Autosomal dominant; Generalized epilepsy with febrile seizures plus, type 11, 602477 (3), Autosomal dominant
HCN4	605206	Sick sinus syndrome 2, 163800 (3), Autosomal dominant; {Epilepsy, idiopathic generalized, susceptibility to, 18}, 619521 (3), Autosomal dominant; Brugada syndrome 8, 613123 (3)
HCRT	602358	?Narcolepsy 1, 161400 (3), Autosomal dominant
HDAC4	605314	Neurodevelopmental disorder with central hypotonia and dysmorphic facies, 619797 (3), Autosomal dominant
HDAC6	300272	?Chondrodysplasia with platyspondyly, distinctive brachydactyly, hydrocephaly, and microphthalmia, 300863 (3), X-linked dominant

HDAC8	300269	Cornelia de Lange syndrome 5, 300882 (3), X-linked dominant
HDC	142704	{Gilles de la Tourette syndrome, susceptibility to}, 137580 (3), Autosomal dominant
HECW2	617245	Neurodevelopmental disorder with hypotonia, seizures, and absent language, 617268 (3), Autosomal dominant
HELLS	603946	Immunodeficiency-centromeric instability-facial anomalies syndrome 4, 616911 (3), Autosomal recessive
HEPACAM	611642	Megalencephalic leukoencephalopathy with subcortical cysts 2A, 613925 (3), Autosomal recessive; Megalencephalic leukoencephalopathy with subcortical cysts 2B, remitting, with or without mental retardation, 613926 (3), Autosomal dominant
HEPHL1	618455	?Abnormal hair, joint laxity, and developmental delay, 261990 (3), Autosomal recessive
HERC1	605109	Macrocephaly, dysmorphic facies, and psychomotor retardation, 617011 (3), Autosomal recessive
HERC2	605837	Intellectual developmental disorder, autosomal recessive 38, 615516 (3), Autosomal recessive; [Skin/hair/eye pigmentation 1, blond/brown hair], 227220 (3), Autosomal recessive; [Skin/hair/eye pigmentation 1, blue/nonblue eyes], 227220 (3), Autosomal recessive
HES7	608059	Spondylocostal dysostosis 4, autosomal recessive, 613686 (3), Autosomal recessive
HESX1	601802	Pituitary hormone deficiency, combined, 5, 182230 (3), Autosomal dominant, Autosomal recessive; Septooptic dysplasia, 182230 (3), Autosomal dominant, Autosomal recessive; Growth hormone deficiency with pituitary anomalies, 182230 (3), Autosomal dominant, Autosomal recessive
HEXA	606869	[Hex A pseudodeficiency], 272800 (3), Autosomal recessive; GM2-gangliosidosis, several forms, 272800 (3), Autosomal recessive; Tay-Sachs disease, 272800 (3), Autosomal recessive
HEXB	606873	Sandhoff disease, infantile, juvenile, and adult forms, 268800 (3), Autosomal recessive
HFE	613609	{Porphyria variegata, susceptibility to}, 176200 (3), Autosomal dominant; {Microvascular complications of diabetes 7}, 612635 (3); Hemochromatosis, 235200 (3), Autosomal recessive; {Alzheimer disease, susceptibility to}, 104300 (3), Autosomal dominant; [Transferrin serum level QTL2], 614193 (3); {Porphyria cutanea tarda, susceptibility to}, 176100 (3), Autosomal dominant, Autosomal recessive
HFM1	615684	Premature ovarian failure 9, 615724 (3), Autosomal recessive
HGD	607474	Alkaptonuria, 203500 (3), Autosomal recessive
HGF	142409	Deafness, autosomal recessive 39, 608265 (3), Autosomal recessive
HGSNAT	610453	Mucopolysaccharidosis type IIIC (Sanfilippo C), 252930 (3), Autosomal recessive; Retinitis pigmentosa 73, 616544 (3), Autosomal recessive
HHAT	605743	Nivelon-Nivelon-Mabille syndrome, 600092 (3), Autosomal recessive
HIBCH	610690	3-hydroxyisobutyryl-CoA hydrolase deficiency, 250620 (3), Autosomal recessive

HID1	605752	Developmental and epileptic encephalopathy 105 with hypopituitarism, 619983 (3), Autosomal recessive
HIKESHI	614908	Leukodystrophy, hypomyelinating, 13, 616881 (3), Autosomal recessive
HINT1	601314	Neuromyotonia and axonal neuropathy, autosomal recessive, 137200 (3), Autosomal recessive
HIVEP2	143054	Intellectual developmental disorder, autosomal dominant 43, 616977 (3), Autosomal dominant
HJV	608374	Hemochromatosis, type 2A, 602390 (3), Autosomal recessive
HK1	142600	Retinitis pigmentosa 79, 617460 (3), Autosomal dominant; Neuropathy, hereditary motor and sensory, Russe type, 605285 (3), Autosomal recessive; Neurodevelopmental disorder with visual defects and brain anomalies, 618547 (3), Autosomal dominant; Hemolytic anemia due to hexokinase deficiency, 235700 (3), Autosomal recessive
HKDC1	617221	Retinitis pigmentosa 92, 619614 (3), Autosomal recessive
HLA-A	142800	{Hypersensitivity syndrome, carbamazepine-induced, susceptibility to}, 608579 (3)
HLA-B	142830	{Synovitis, chronic, susceptibility to} (3); {Abacavir hypersensitivity, susceptibility to} (3); {Spondyloarthropathy, susceptibility to, 1}, 106300 (3), Multifactorial; {Stevens-Johnson syndrome, susceptibility to}, 608579 (3); {Drug-induced liver injury due to flucloxacillin} (3); {Toxic epidermal necrolysis, susceptibility to}, 608579 (3)
HLA-C	142840	{Psoriasis susceptibility 1}, 177900 (3), Multifactorial; {HIV-1 viremia, susceptibility to}, 609423 (3)
HLA-DPB1	142858	{Beryllium disease, chronic, susceptibility to} (3)
HLA-DQA1	146880	{Celiac disease, susceptibility to}, 212750 (3), Multifactorial, Autosomal recessive
HLA-DQB1	604305	{Celiac disease, susceptibility to}, 212750 (3), Multifactorial, Autosomal recessive; {Multiple sclerosis, susceptibility to, 1}, 126200 (3), Multifactorial; {Creutzfeldt-Jakob disease, variant, resistance to}, 123400 (3), Autosomal dominant
HLA-DRB1	142857	{Multiple sclerosis, susceptibility to, 1}, 126200 (3), Multifactorial; {Sarcoidosis, susceptibility to, 1}, 181000 (3), Autosomal dominant
HLA-G	142871	{Asthma, susceptibility to}, 600807 (2), Autosomal dominant
HLCS	609018	Holocarboxylase synthetase deficiency, 253270 (3), Autosomal recessive
HMBS	609806	Porphyria, acute intermittent, nonerythroid variant, 176000 (3), Autosomal dominant; Porphyria, acute intermittent, 176000 (3), Autosomal dominant
HMCN1	608548	{Macular degeneration, age-related, 1}, 603075 (3), Autosomal dominant
HMGA1	600701	{Type 2 diabetes mellitus, susceptibility to}, 125853 (3), Autosomal dominant
HMGA2	600698	Silver-Russell syndrome 5, 618908 (3), Autosomal dominant
HMGB3	300193	?Microphthalmia, syndromic 13, 300915 (3), X-linked
HMGCL	613898	HMG-CoA lyase deficiency, 246450 (3), Autosomal recessive
HMGCR	142910	[Statins, attenuated cholesterol lowering by] (3); [Low density lipoprotein cholesterol level QTL 3] (3)

HMGCS2	600234	HMG-CoA synthase-2 deficiency, 605911 (3), Autosomal recessive
HMMR	600936	{Breast cancer, susceptibility to}, 114480 (3), Autosomal dominant, Somatic mutation
HMOX1	141250	Heme oxygenase-1 deficiency, 614034 (3), Autosomal recessive; {Pulmonary disease, chronic obstructive, susceptibility to}, 606963 (3)
HMX1	142992	Oculoauricular syndrome, 612109 (3), Autosomal recessive
HNF1A	142410	Hepatic adenoma, somatic, 142330 (3); Diabetes mellitus, insulin-dependent, 20, 612520 (3); {Diabetes mellitus, noninsulin-dependent, 2}, 125853 (3), Autosomal dominant; MODY, type III, 600496 (3), Autosomal dominant; {Diabetes mellitus, insulin-dependent}, 222100 (3), Autosomal recessive; Renal cell carcinoma, 144700 (3)
HNF1B	189907	Type 2 diabetes mellitus, 125853 (3), Autosomal dominant; Renal cysts and diabetes syndrome, 137920 (3), Autosomal dominant; {Renal cell carcinoma}, 144700 (3)
HNF4A	600281	Fanconi renotubular syndrome 4, with maturity-onset diabetes of the young, 616026 (3), Autosomal dominant; {Diabetes mellitus, noninsulin-dependent}, 125853 (3), Autosomal dominant; MODY, type I, 125850 (3), Autosomal dominant
HNMT	605238	Intellectual developmental disorder, autosomal recessive 51, 616739 (3), Autosomal recessive; {Asthma, susceptibility to}, 600807 (3), Autosomal dominant
HNRNPA1	164017	?Inclusion body myopathy with early-onset Paget disease without frontotemporal dementia 3, 615424 (3), Autosomal dominant; Amyotrophic lateral sclerosis 20, 615426 (3), Autosomal dominant
HNRNPA2B1	600124	?Inclusion body myopathy with early-onset Paget disease with or without frontotemporal dementia 2, 615422 (3), Autosomal dominant
HNRNPDL	607137	Muscular dystrophy, limb-girdle, autosomal dominant 3, 609115 (3), Autosomal dominant
HNRNPH2	300610	Intellectual developmental disorder, X-linked syndromic, Bain type, 300986 (3), X-linked dominant
HNRNPK	600712	Au-Kline syndrome, 616580 (3), Autosomal dominant
HNRNPU	602869	Developmental and epileptic encephalopathy 54, 617391 (3), Autosomal dominant
HOGA1	613597	Hyperoxaluria, primary, type III, 613616 (3), Autosomal recessive
HOMER2	604799	?Deafness, autosomal dominant 68, 616707 (3), Autosomal dominant
HOXA1	142955	Bosley-Salih-Alorainy syndrome, 601536 (3), Autosomal recessive; Athabaskan brainstem dysgenesis syndrome, 601536 (3), Autosomal recessive
HOXA11	142958	Radioulnar synostosis with amegakaryocytic thrombocytopenia 1, 605432 (3), Autosomal dominant
HOXA13	142959	Hand-foot-uterus syndrome, 140000 (3), Autosomal dominant; ?Guttmacher syndrome, 176305 (3), Autosomal dominant

HOXA2	604685	Microtia with or without hearing impairment (AD), 612290 (3), Autosomal dominant, Autosomal recessive; ?Microtia, hearing impairment, and cleft palate (AR), 612290 (3), Autosomal dominant, Autosomal recessive
HOXB1	142968	Facial paresis, hereditary congenital, 3, 614744 (3), Autosomal recessive
HOXB13	604607	{Prostate cancer, hereditary, 9}, 610997 (3)
HOXC13	142976	Ectodermal dysplasia 9, hair/nail type, 614931 (3), Autosomal recessive
HOXD10	142984	Vertical talus, congenital, 192950 (3), Autosomal dominant; Charcot-Marie-Tooth disease, foot deformity of, 192950 (3), Autosomal dominant
HOXD13	142989	Syndactyly, type V, 186300 (3), Autosomal dominant; Synpolydactyly 1, 186000 (3), Autosomal dominant; Brachydactyly, type E, 113300 (3), Autosomal dominant; Brachydactyly, type D, 113200 (3), Autosomal dominant; ?Brachydactyly-syndactyly syndrome, 610713 (3)
HP	140100	[Anhaptoglobinemia], 614081 (3); [Hypohaptoglobinemia], 614081 (3)
HPCA	142622	Dystonia 2, torsion, autosomal recessive, 224500 (3), Autosomal recessive
HPD	609695	Hawkinsinuria, 140350 (3), Autosomal dominant; Tyrosinemia, type III, 276710 (3), Autosomal recessive
HPDL	618994	Neurodevelopmental disorder with progressive spasticity and brain white matter abnormalities, 619026 (3), Autosomal recessive; Spastic paraplegia 83, autosomal recessive, 619027 (3), Autosomal recessive
HPGD	601688	?Digital clubbing, isolated congenital, 119900 (3), Autosomal recessive; Hypertrophic osteoarthropathy, primary, autosomal recessive 1, 259100 (3), Autosomal recessive; Cranioosteoarthropathy, 259100 (3), Autosomal recessive
HPRT1	308000	Hyperuricemia, HRPT-related, 300323 (3), X-linked recessive; Lesch-Nyhan syndrome, 300322 (3), X-linked recessive
HPS1	604982	Hermansky-Pudlak syndrome 1, 203300 (3), Autosomal recessive
HPS3	606118	Hermansky-Pudlak syndrome 3, 614072 (3), Autosomal recessive
HPS4	606682	Hermansky-Pudlak syndrome 4, 614073 (3), Autosomal recessive
HPS5	607521	Hermansky-Pudlak syndrome 5, 614074 (3), Autosomal recessive
HPS6	607522	Hermansky-Pudlak syndrome 6, 614075 (3), Autosomal recessive
HPSE2	613469	Urofacial syndrome 1, 236730 (3), Autosomal recessive
HR	602302	Atrichia with papular lesions, 209500 (3), Autosomal recessive; Alopecia universalis, 203655 (3), Autosomal recessive
HRAS	190020	Bladder cancer, somatic, 109800 (3); Thyroid carcinoma, follicular, somatic, 188470 (3); Congenital myopathy with excess of muscle spindles, 218040 (3), Autosomal dominant; Nevus sebaceous or woolly hair nevus, somatic, 162900 (3); Schimmelpenning-Feuerstein-Mims syndrome, somatic mosaic, 163200 (3); Spitz nevus or nevus spilus, somatic, 137550 (3); Costello syndrome, 218040 (3), Autosomal dominant
HRG	142640	Thrombophilia 11 due to HRG deficiency, 613116 (3), Autosomal dominant
HRURF	619257	Hypotrichosis 4, 146550 (3), Autosomal dominant
HS2ST1	604844	Neurofacioskeletal syndrome with or without renal agenesis, 619194 (3), Autosomal recessive
HS3ST6	619210	?Angioedema, hereditary, 8, 619367 (3), Autosomal dominant

HS6ST1	604846	{Hypogonadotropic hypogonadism 15 with or without anosmia}, 614880 (3), Autosomal dominant
HS6ST2	300545	?Paganini-Miozzo syndrome, 301025 (3), X-linked recessive
HSCB	608142	?Anemia, sideroblastic, 5, 619523 (3), Autosomal recessive
HSD11B1	600713	Cortisone reductase deficiency 2, 614662 (3), Autosomal dominant
HSD11B2	614232	Apparent mineralocorticoid excess, 218030 (3), Autosomal recessive
HSD17B10	300256	HSD10 mitochondrial disease, 300438 (3), X-linked dominant
HSD17B3	605573	Pseudohermaphroditism, male, with gynecomastia, 264300 (3), Autosomal recessive
HSD17B4	601860	D-bifunctional protein deficiency, 261515 (3), Autosomal recessive; Perrault syndrome 1, 233400 (3), Autosomal recessive
HSD3B2	613890	Adrenal hyperplasia, congenital, due to 3-beta-hydroxysteroid dehydrogenase 2 deficiency, 201810 (3), Autosomal recessive
HSD3B7	607764	Bile acid synthesis defect, congenital, 1, 607765 (3), Autosomal recessive
HSF2BP	604554	Premature ovarian failure 19, 619245 (3), Autosomal recessive
HSF4	602438	Cataract 5, multiple types, 116800 (3), Autosomal dominant
HSPA9	600548	Even-plus syndrome, 616854 (3), Autosomal recessive; Anemia, sideroblastic, 4, 182170 (3), Autosomal dominant
HSPB1	602195	Neuronopathy, distal hereditary motor, type IIB, 608634 (3), Autosomal dominant; Charcot-Marie-Tooth disease, axonal, type 2F, 606595 (3), Autosomal dominant
HSPB3	604624	?Neuronopathy, distal hereditary motor, type IIC, 613376 (3), Autosomal dominant
HSPB8	608014	Neuronopathy, distal hereditary motor, type IIA, 158590 (3), Autosomal dominant; Charcot-Marie-Tooth disease, axonal, type 2L, 608673 (3), Autosomal dominant
HSPD1	118190	Spastic paraplegia 13, autosomal dominant, 605280 (3), Autosomal dominant; Leukodystrophy, hypomyelinating, 4, 612233 (3), Autosomal recessive
HSPG2	142461	Dyssegmental dysplasia, Silverman-Handmaker type, 224410 (3), Autosomal recessive; Schwartz-Jampel syndrome, type 1, 255800 (3), Autosomal recessive
HTR1A	109760	Periodic fever, menstrual cycle dependent, 614674 (3), Autosomal dominant
HTR2A	182135	{Alcohol dependence, susceptibility to}, 103780 (3), Multifactorial; {Anorexia nervosa, susceptibility to}, 606788 (3); {Major depressive disorder, response to citalopram therapy in}, 608516 (3); {Seasonal affective disorder, susceptibility to}, 608516 (3); {Obsessive-compulsive disorder, susceptibility to}, 164230 (3), Autosomal dominant; {Schizophrenia, susceptibility to}, 181500 (3), Autosomal dominant

HTRA1	602194	{Macular degeneration, age-related, neovascular type}, 610149 (3); {Macular degeneration, age-related, 7}, 610149 (3); CARASIL syndrome, 600142 (3), Autosomal recessive; Cerebral arteriopathy, autosomal dominant, with subcortical infarcts and leukoencephalopathy, type 2, 616779 (3), Autosomal dominant
HTRA2	606441	{Parkinson disease 13}, 610297 (3); 3-methylglutaconic aciduria, type VIII, 617248 (3), Autosomal recessive
HTT	613004	Lopes-Maciel-Rodan syndrome, 617435 (3), Autosomal recessive; Huntington disease, 143100 (3), Autosomal dominant
HUWE1	300697	Intellectual developmental disorder, X-linked syndromic, Turner type, 309590 (3), X-linked
HYAL1	607071	Mucopolysaccharidosis type IX, 601492 (3), Autosomal recessive
HYCC1 (FAM126A)	610531	Leukodystrophy, hypomyelinating, 5, 610532 (3), Autosomal recessive
HYDIN	610812	Ciliary dyskinesia, primary, 5, 608647 (3), Autosomal recessive
HYLS1	610693	Hydrolethalus syndrome, 236680 (3), Autosomal recessive
HYOU1	601746	?Immunodeficiency 59 and hypoglycemia, 233600 (3), Autosomal recessive
IARS1	600709	Growth retardation, impaired intellectual development, hypotonia, and hepatopathy, 617093 (3), Autosomal recessive
IARS2	612801	Cataracts, growth hormone deficiency, sensory neuropathy, sensorineural hearing loss, and skeletal dysplasia, 616007 (3), Autosomal recessive
IBA57	615316	Multiple mitochondrial dysfunctions syndrome 3, 615330 (3), Autosomal recessive; ?Spastic paraplegia 74, autosomal recessive, 616451 (3), Autosomal recessive
ICAM1	147840	{Malaria, cerebral, susceptibility to}, 611162 (3)
ICAM4	614088	[Blood group, Landsteiner-Wiener], 111250 (3)
ICOS	604558	Immunodeficiency, common variable, 1, 607594 (3), Autosomal recessive
IDH1	147700	{Glioma, susceptibility to, somatic}, 137800 (3)
IDH2	147650	D-2-hydroxyglutaric aciduria 2, 613657 (3)
IDH3A	601149	Retinitis pigmentosa 90, 619007 (3), Autosomal recessive
IDH3B	604526	Retinitis pigmentosa 46, 612572 (3), Autosomal recessive
IDS	300823	Mucopolysaccharidosis II, 309900 (3), X-linked recessive
IDUA	252800	Mucopolysaccharidosis Is, 607016 (3), Autosomal recessive; Mucopolysaccharidosis Ih/s, 607015 (3), Autosomal recessive; Mucopolysaccharidosis Ih, 607014 (3), Autosomal recessive
IER3IP1	609382	Microcephaly, epilepsy, and diabetes syndrome, 614231 (3), Autosomal recessive
IFIH1	606951	Immunodeficiency 95, 619773 (3), Autosomal recessive; Aicardi-Goutieres syndrome 7, 615846 (3), Autosomal dominant; Singleton-Merten syndrome 1, 182250 (3), Autosomal dominant
IFITM3	605579	{Influenza, severe, susceptibility to}, 614680 (3)
IFITM5	614757	Osteogenesis imperfecta, type V, 610967 (3), Autosomal dominant
IFNA1	147660	Interferon, alpha, deficiency (1)

IFNAR1	107450	Immunodeficiency 106, susceptibility to viral infections, 619935 (3), Autosomal recessive
IFNAR2	602376	{Hepatitis B virus, susceptibility to}, 610424 (3); Immunodeficiency 45, 616669 (3), Autosomal recessive
IFNG	147570	{Hepatitis C virus, response to therapy of}, 609532 (3); {TSC2 angiomyolipomas, renal, modifier of}, 613254 (3), Autosomal dominant; {Aplastic anemia}, 609135 (3); ?Immunodeficiency 69, mycobacteriosis, 618963 (3), Autosomal recessive; {Tuberculosis, protection against}, 607948 (3); {AIDS, rapid progression to}, 609423 (3)
IFNGR1	107470	{H. pylori infection, susceptibility to}, 600263 (3); Immunodeficiency 27A, mycobacteriosis, AR, 209950 (3), Autosomal recessive; Immunodeficiency 27B, mycobacteriosis, AD, 615978 (3), Autosomal dominant; {Tuberculosis infection, protection against}, 607948 (3); {Tuberculosis, susceptibility to}, 607948 (3); {Hepatitis B virus infection, susceptibility to}, 610424 (3)
IFNGR2	147569	Immunodeficiency 28, mycobacteriosis, 614889 (3), Autosomal recessive
IFNL3	607402	{Hepatitis C virus infection, response to therapy of}, 609532 (3)
IFT122	606045	Cranioectodermal dysplasia 1, 218330 (3), Autosomal recessive
IFT140	614620	Short-rib thoracic dysplasia 9 with or without polydactyly, 266920 (3), Autosomal recessive; Retinitis pigmentosa 80, 617781 (3), Autosomal recessive
IFT172	607386	Retinitis pigmentosa 71, 616394 (3), Autosomal recessive; Bardet-Biedl syndrome 20, 619471 (3), Autosomal recessive; Short-rib thoracic dysplasia 10 with or without polydactyly, 615630 (3), Autosomal recessive
IFT27	615870	Bardet-Biedl syndrome 19, 615996 (3), Autosomal recessive
IFT43	614068	?Cranioectodermal dysplasia 3, 614099 (3), Autosomal recessive; ?Retinitis pigmentosa 81, 617871 (3), Autosomal recessive; Short-rib thoracic dysplasia 18 with polydactyly, 617866 (3), Autosomal recessive
IFT52	617094	Short-rib thoracic dysplasia 16 with or without polydactyly, 617102 (3), Autosomal recessive
IFT57	606621	?Orofaciodigital syndrome XVIII, 617927 (3), Autosomal recessive
IFT74	608040	Bardet-Biedl syndrome 22, 617119 (3), Autosomal recessive; Spermatogenic failure 58, 619585 (3), Autosomal recessive; Joubert syndrome 40, 619582 (3), Autosomal recessive
IFT80	611177	Short-rib thoracic dysplasia 2 with or without polydactyly, 611263 (3), Autosomal recessive
IFT81	605489	Short-rib thoracic dysplasia 19 with or without polydactyly, 617895 (3), Autosomal recessive
IGBP1	300139	?Corpus callosum, agenesis of, with impaired intellectual development, ocular coloboma and micrognathia, 300472 (3), X-linked recessive
IGF1	147440	Growth retardation with deafness and mental retardation due to IGF1 deficiency, 608747 (3), Autosomal recessive
IGF1R	147370	Insulin-like growth factor I, resistance to, 270450 (3), Autosomal dominant, Autosomal recessive
IGF2	147470	Silver-Russell syndrome 3, 616489 (3), Autosomal dominant

IGF2BP2	608289	{Diabetes mellitus, noninsulin-dependent, susceptibility to}, 125853 (3), Autosomal dominant
IGF2R	147280	Hepatocellular carcinoma, somatic, 114550 (3)
IGFALS	601489	Acid-labile subunit, deficiency of, 615961 (3), Autosomal recessive
IGFBP7	602867	Retinal arterial macroaneurysm with supravalvular pulmonic stenosis, 614224 (3), Autosomal recessive
IGHG2	147110	IgG2 deficiency, selective (3)
IGHM	147020	Agammaglobulinemia 1, 601495 (3), Autosomal recessive
IGHMBP2	600502	Neuronopathy, distal hereditary motor, type VI, 604320 (3), Autosomal recessive; Charcot-Marie-Tooth disease, axonal, type 2S, 616155 (3), Autosomal recessive
IGKC	147200	Kappa light chain deficiency, 614102 (3), Autosomal recessive
IGLL1	146770	Agammaglobulinemia 2, 613500 (3), Autosomal recessive
IGSF1	300137	Hypothyroidism, central, and testicular enlargement, 300888 (3), X-linked recessive
IGSF3	603491	?Lacrimal duct defect, 149700 (3), Autosomal recessive
IHH	600726	Acrocapitofemoral dysplasia, 607778 (3), Autosomal recessive; Brachydactyly, type A1, 112500 (3), Autosomal dominant
IKBKB	603258	Immunodeficiency 15B, 615592 (3), Autosomal recessive; Immunodeficiency 15A, 618204 (3), Autosomal dominant
IKBKG	300248	Incontinentia pigmenti, 308300 (3), X-linked dominant; Ectodermal dysplasia and immunodeficiency 1, 300291 (3), X-linked recessive; Immunodeficiency 33, 300636 (3), X-linked recessive; Autoinflammatory disease, systemic, X-linked, 301081 (3), X-linked
IKZF1	603023	Immunodeficiency, common variable, 13, 616873 (3), Autosomal dominant
IKZF3	606221	?Immunodeficiency 84, 619437 (3)
IKZF5	606238	Thrombocytopenia, autosomal dominant, 7, 619130 (3), Autosomal dominant
IL10	124092	{Rheumatoid arthritis, progression of}, 180300 (3); {Graft-versus-host disease, protection against}, 614395 (3); {HIV-1, susceptibility to}, 609423 (3)
IL10RA	146933	Inflammatory bowel disease 28, early onset, autosomal recessive, 613148 (3), Autosomal recessive
IL10RB	123889	{Hepatitis B virus, susceptibility to}, 610424 (3); Inflammatory bowel disease 25, early onset, autosomal recessive, 612567 (3), Autosomal recessive
IL11RA	600939	Craniosynostosis and dental anomalies, 614188 (3), Autosomal recessive
IL12B	161561	Immunodeficiency 29, mycobacteriosis, 614890 (3), Autosomal recessive
IL12RB1	601604	Immunodeficiency 30, 614891 (3), Autosomal recessive
IL13	147683	{Asthma, susceptibility to}, 600807 (3), Autosomal dominant; {Allergic rhinitis, susceptibility to}, 607154 (3)
IL17F	606496	?Candidiasis, familial, 6, autosomal dominant, 613956 (3)
IL17RA	605461	Immunodeficiency 51, 613953 (3), Autosomal recessive
IL17RC	610925	Candidiasis, familial, 9, 616445 (3), Autosomal recessive

IL17RD	606807	Hypogonadotropic hypogonadism 18 with or without anosmia, 615267 (3), Digenic dominant, Autosomal dominant, Autosomal recessive
IL18BP	604113	{?Hepatitis, fulminant viral, susceptibility to}, 618549 (3), Autosomal recessive
IL1B	147720	{Gastric cancer risk after H. pylori infection}, 613659 (3)
IL1RAPL1	300206	Intellectual developmental disorder, X-linked 21, 300143 (3), X-linked recessive
IL1RN	147679	{Gastric cancer risk after H. pylori infection}, 613659 (3); {Microvascular complications of diabetes 4}, 612628 (3); Interleukin 1 receptor antagonist deficiency, 612852 (3), Autosomal recessive
IL21	605384	?Immunodeficiency, common variable, 11, 615767 (3), Autosomal recessive
IL21R	605383	[IgE, elevated level of], 147050 (3), Autosomal dominant; Immunodeficiency 56, 615207 (3), Autosomal recessive
IL23R	607562	{Inflammatory bowel disease 17, protection against}, 612261 (3); {Psoriasis, protection against}, 605606 (3)
IL2RA	147730	Immunodeficiency 41 with lymphoproliferation and autoimmunity, 606367 (3), Autosomal recessive; {Diabetes, mellitus, insulin-dependent, susceptibility to, 10}, 601942 (3)
IL2RB	146710	Immunodeficiency 63 with lymphoproliferation and autoimmunity, 618495 (3), Autosomal recessive
IL2RG	308380	Combined immunodeficiency, X-linked, moderate, 312863 (3), X-linked recessive; Severe combined immunodeficiency, X-linked, 300400 (3), X-linked recessive
IL31RA	609510	?Amyloidosis, primary localized cutaneous, 2, 613955 (3), Autosomal dominant
IL36RN	605507	Psoriasis 14, pustular, 614204 (3), Autosomal recessive
IL37	605510	?Inflammatory bowel disease (infantile ulcerative colitis) 31, 619398 (3), Autosomal recessive
IL4R	147781	{AIDS, slow progression to}, 609423 (3); {Atopy, susceptibility to}, 147050 (3), Autosomal dominant
IL6	147620	{Type 2 diabetes mellitus}, 125853 (3), Autosomal dominant; {Rheumatoid arthritis, systemic juvenile}, 604302 (3); {Intracranial hemorrhage in brain cerebrovascular malformations, susceptibility to}, 108010 (3), Somatic mutation; {Type 1 diabetes mellitus}, 222100 (3), Autosomal recessive; {Crohn disease-associated growth failure}, 266600 (3), Multifactorial; {Kaposi sarcoma, susceptibility to}, 148000 (3), Autosomal dominant
IL6R	147880	[Interleukin 6, serum level of, QTL], 614752 (3); Hyper-IgE recurrent infection syndrome 5, autosomal recessive, 618944 (3), Autosomal recessive; [Interleukin-6 receptor, soluble, serum level of, QTL], 614689 (3)

IL6ST	600694	Stuve-Wiedemann syndrome 2, 619751 (3), Autosomal recessive; Hyper-IgE recurrent infection syndrome 4A, autosomal dominant, 619752 (3), Autosomal dominant; ?Immunodeficiency 94 with autoinflammation and dysmorphic facies, 619750 (3), Autosomal dominant; Hyper-IgE recurrent infection syndrome 4B, autosomal recessive, 618523 (3), Autosomal recessive
IL7	146660	{?Epidermodysplasia verruciformis, susceptibility to, 5}, 618309 (3), Autosomal recessive
IL7R	146661	Immunodeficiency 104, severe combined, 608971 (3), Autosomal recessive
ILDR1	609739	Deafness, autosomal recessive 42, 609646 (3), Autosomal recessive
IMPA1	602064	Intellectual developmental disorder, autosomal recessive 59, 617323 (3), Autosomal recessive
IMPDH1	146690	Retinitis pigmentosa 10, 180105 (3), Autosomal dominant; Leber congenital amaurosis 11, 613837 (3), Autosomal dominant
IMPDH2	146691	[IMPDH2 enzyme activity, variation in], 617995 (3)
IMPG1	602870	Macular dystrophy, vitelliform, 4, 616151 (3), Autosomal dominant, Autosomal recessive; Retinitis pigmentosa 91, 153870 (3), Autosomal dominant
IMPG2	607056	Retinitis pigmentosa 56, 613581 (3), Autosomal recessive; Macular dystrophy, vitelliform, 5, 616152 (3), Autosomal dominant
INAVA	618051	{Inflammatory bowel disease 29}, 618077 (3), Autosomal dominant
INF2	610982	Glomerulosclerosis, focal segmental, 5, 613237 (3); Charcot-Marie-Tooth disease, dominant intermediate E, 614455 (3), Autosomal dominant
ING1	601566	Squamous cell carcinoma, head and neck, somatic, 275355 (3)
INPP5E	613037	Joubert syndrome 1, 213300 (3), Autosomal recessive; Mental retardation, truncal obesity, retinal dystrophy, and micropenis, 610156 (3), Autosomal recessive
INPP5K	607875	Muscular dystrophy, congenital, with cataracts and intellectual disability, 617404 (3), Autosomal recessive
INPPL1	600829	Opsismodysplasia, 258480 (3), Autosomal recessive
INS	176730	Diabetes mellitus, insulin-dependent, 2, 125852 (3), Autosomal dominant; Maturity-onset diabetes of the young, type 10, 613370 (3), Autosomal dominant; Hyperproinsulinemia, 616214 (3), Autosomal dominant; Diabetes mellitus, permanent neonatal 4, 618858 (3), Autosomal dominant, Autosomal recessive
INSL3	146738	Cryptorchidism, 219050 (3), Autosomal dominant
INSR	147670	Rabson-Mendenhall syndrome, 262190 (3), Autosomal recessive; Leprechaunism, 246200 (3), Autosomal recessive; Diabetes mellitus, insulin-resistant, with acanthosis nigricans, 610549 (3); Hyperinsulinemic hypoglycemia, familial, 5, 609968 (3), Autosomal dominant
INTS1	611345	Neurodevelopmental disorder with cataracts, poor growth, and dysmorphic facies, 618571 (3), Autosomal recessive
INTS8	611351	?Neurodevelopmental disorder with cerebellar hypoplasia and spasticity, 618572 (3), Autosomal recessive

INTU	610621	?Orofaciodigital syndrome XVII, 617926 (3), Autosomal recessive; ?Short-rib thoracic dysplasia 20 with polydactyly, 617925 (3), Autosomal recessive
INVS	243305	Nephronophthisis 2, infantile, 602088 (3), Autosomal recessive
IPO8	605600	VISS syndrome, 619472 (3), Autosomal recessive
IQCB1	609237	Senior-Loken syndrome 5, 609254 (3), Autosomal recessive
IQCE	617631	Polydactyly, postaxial, type A7, 617642 (3), Autosomal recessive
IQSEC1	610166	Intellectual developmental disorder with short stature and behavioral abnormalities, 618687 (3), Autosomal recessive
IQSEC2	300522	Intellectual developmental disorder, X-linked 1, 309530 (3), X-linked dominant
IRAK3	604459	{Asthma susceptibility 5}, 611064 (3)
IRAK4	606883	Immunodeficiency 67, 607676 (3), Autosomal recessive
IREB2	147582	Neurodegeneration, early-onset, with choreoathetoid movements and microcytic anemia, 618451 (3), Autosomal recessive
IRF1	147575	Nonsmall cell lung cancer, somatic, 211980 (3); Myelodysplastic syndrome, preleukemic (3); Gastric cancer, somatic, 613659 (3); Myelogenous leukemia, acute (3)
IRF2BP2	615332	?Immunodeficiency, common variable, 14, 617765 (3), Autosomal dominant
IRF2BPL	611720	Neurodevelopmental disorder with regression, abnormal movements, loss of speech, and seizures, 618088 (3), Autosomal dominant
IRF3	603734	{Encephalopathy, acute, infection-induced (herpes-specific), susceptibility to, 7}, 616532 (3), Autosomal dominant
IRF4	601900	[Skin/hair/eye pigmentation, variation in, 8], 611724 (3)
IRF5	607218	{Inflammatory bowel disease 14}, 612245 (3); {Systemic lupus erythematosus, susceptibility to, 10}, 612251 (3)
IRF6	607199	{Orofacial cleft 6}, 608864 (3), Autosomal dominant; Popliteal pterygium syndrome 1, 119500 (3), Autosomal dominant; van der Woude syndrome 1, 119300 (3), Autosomal dominant
IRF7	605047	?Immunodeficiency 39, 616345 (3), Autosomal recessive
IRF8	601565	Immunodeficiency 32A, mycobacteriosis, autosomal dominant, 614893 (3), Autosomal dominant; Immunodeficiency 32B, monocyte and dendritic cell deficiency, autosomal recessive, 226990 (3), Autosomal recessive
IRF9	147574	Immunodeficiency 65, susceptibility to viral infections, 618648 (3), Autosomal recessive
IRGM	608212	{Mycobacterium tuberculosis, protection against}, 607948 (3); {Inflammatory bowel disease (Crohn disease) 19}, 612278 (3)
IRS1	147545	{Type 2 diabetes mellitus, susceptibility to}, 125853 (3), Autosomal dominant; {Coronary artery disease, susceptibility to} (3)
IRS2	600797	{Diabetes mellitus, noninsulin-dependent}, 125853 (3), Autosomal dominant
IRS4	300904	Hypothyroidism, congenital, nongoitrous, 9, 301035 (3), X-linked recessive
IRX5	606195	Hamamy syndrome, 611174 (3), Autosomal recessive

ISCA1	611006	Multiple mitochondrial dysfunctions syndrome 5, 617613 (3), Autosomal recessive
ISCA2	615317	Multiple mitochondrial dysfunctions syndrome 4, 616370 (3), Autosomal recessive
ISCU	611911	Myopathy with lactic acidosis, hereditary, 255125 (3), Autosomal recessive
ISG15	147571	Immunodeficiency 38, 616126 (3), Autosomal recessive
ITCH	606409	Autoimmune disease, multisystem, with facial dysmorphism, 613385 (3), Autosomal recessive
ITGA2B	607759	Thrombocytopenia, neonatal alloimmune, BAK antigen related (3); Glanzmann thrombasthenia 1, 273800 (3), Autosomal recessive; Bleeding disorder, platelet-type, 16, autosomal dominant, 187800 (3), Autosomal dominant
ITGA3	605025	Epidermolysis bullosa, junctional 7, with interstitial lung disease and nephrotic syndrome, 614748 (3), Autosomal recessive
ITGA6	147556	Epidermolysis bullosa, junctional 6, with pyloric atresia, 619817 (3), Autosomal recessive
ITGA7	600536	Muscular dystrophy, congenital, due to ITGA7 deficiency, 613204 (3), Autosomal recessive
ITGA8	604063	Renal hypodysplasia/aplasia 1, 191830 (3), Autosomal recessive
ITGB2	600065	Leukocyte adhesion deficiency, 116920 (3), Autosomal recessive
ITGB3	173470	Bleeding disorder, platelet-type, 24, autosomal dominant, 619271 (3); Thrombocytopenia, neonatal alloimmune (3); Purpura, posttransfusion (3); {Myocardial infarction, susceptibility to}, 608446 (3); Glanzmann thrombasthenia 2, 619267 (3)
ITGB4	147557	Epidermolysis bullosa, junctional 5B, with pyloric atresia, 226730 (3), Autosomal recessive; Epidermolysis bullosa, junctional 5A, intermediate, 619816 (3), Autosomal recessive
ITGB6	147558	Amelogenesis imperfecta, type IH, 616221 (3), Autosomal recessive
ITK	186973	Lymphoproliferative syndrome 1, 613011 (3), Autosomal recessive
ITM2B	603904	?Retinal dystrophy with inner retinal dysfunction and ganglion cell abnormalities, 616079 (3), Autosomal dominant; Dementia, familial British, 176500 (3), Autosomal dominant; Dementia, familial Danish, 117300 (3), Autosomal dominant
ITPA	147520	[Inosine triphosphatase deficiency], 613850 (3); Developmental and epileptic encephalopathy 35, 616647 (3), Autosomal recessive
ITPR1	147265	Gillespie syndrome, 206700 (3), Autosomal dominant, Autosomal recessive; Spinocerebellar ataxia 29, congenital nonprogressive, 117360 (3), Autosomal dominant; Spinocerebellar ataxia 15, 606658 (3), Autosomal dominant
ITPR2	600144	?Anhidrosis, isolated, with normal sweat glands, 106190 (3), Autosomal recessive
ITPR3	147267	{Diabetes, type 1, susceptibility to}, 222100 (2), Autosomal recessive
IVD	607036	Isovaleric acidemia, 243500 (3), Autosomal recessive
IVNS1ABP	609209	Immunodeficiency 70, 618969 (3), Autosomal dominant

IYD	612025	Thyroid dyshormonogenesis 4, 274800 (3), Autosomal recessive
JAG1	601920	?Deafness, congenital heart defects, and posterior embryotoxon, 617992 (3), Autosomal dominant; Charcot-Marie-Tooth disease, axonal, type 2HH, 619574 (3), Autosomal dominant; Alagille syndrome 1, 118450 (3), Autosomal dominant; Tetralogy of Fallot, 187500 (3), Autosomal dominant
JAG2	602570	Muscular dystrophy, limb-girdle, autosomal recessive 27, 619566 (3), Autosomal recessive
JAGN1	616012	Neutropenia, severe congenital, 6, autosomal recessive, 616022 (3), Autosomal recessive
JAK1	147795	Autoinflammation, immune dysregulation, and eosinophilia, 618999 (3), Autosomal dominant
JAK2	147796	{Budd-Chiari syndrome, somatic}, 600880 (3); Myelofibrosis, somatic, 254450 (3); Erythrocytosis, somatic, 133100 (3); Leukemia, acute myeloid, somatic, 601626 (3); Thrombocythemia 3, 614521 (3), Autosomal dominant, Somatic mutation; Polycythemia vera, somatic, 263300 (3)
JAK3	600173	SCID, autosomal recessive, T-negative/B-positive type, 600802 (3), Autosomal recessive
JAM2	606870	Basal ganglia calcification, idiopathic, 8, autosomal recessive, 618824 (3), Autosomal recessive
JAM3	606871	Hemorrhagic destruction of the brain, subependymal calcification, and cataracts, 613730 (3), Autosomal recessive
JPH1	605266	?Charcot-Marie-Tooth disease, axonal, autosomal dominant, type 2K, 607831 (3), Autosomal dominant, Autosomal recessive
JPH2	605267	Cardiomyopathy, dilated, 2E, 619492 (3), Autosomal recessive; Cardiomyopathy, hypertrophic, 17, 613873 (3), Autosomal dominant
JPH3	605268	Huntington disease-like 2, 606438 (3), Autosomal dominant
JUP	173325	Naxos disease, 601214 (3), Autosomal recessive; ?Arrhythmogenic right ventricular dysplasia 12, 611528 (3), Autosomal dominant
KANK1	607704	Cerebral palsy, spastic quadriplegic, 2, 612900 (3)
KANK2	614610	Nephrotic syndrome, type 16, 617783 (3), Autosomal recessive; Palmoplantar keratoderma and woolly hair, 616099 (3), Autosomal recessive
KANSL1	612452	Koolen-De Vries syndrome, 610443 (3), Autosomal dominant
KARS1	601421	Deafness, autosomal recessive 89, 613916 (3), Autosomal recessive; Leukoencephalopathy, progressive, infantile-onset, with or without deafness, 619147 (3), Autosomal recessive; ?Charcot-Marie-Tooth disease, recessive intermediate, B, 613641 (3), Autosomal recessive; Deafness, congenital, and adult-onset progressive leukoencephalopathy, 619196 (3), Autosomal recessive
KAT5	601409	Neurodevelopmental disorder with dysmorphic facies, sleep disturbance, and brain abnormalities, 619103 (3), Autosomal dominant
KAT6A	601408	Arboleda-Tham syndrome, 616268 (3), Autosomal dominant
KAT6B	605880	SBBYSS syndrome, 603736 (3), Autosomal dominant; Genitopatellar syndrome, 606170 (3), Autosomal dominant

KAT8	609912	Li-Ghorgani-Weisz-Hubshman syndrome, 618974 (3), Autosomal dominant
KATNB1	602703	Lissencephaly 6, with microcephaly, 616212 (3), Autosomal recessive
KATNIP	616650	Joubert syndrome 26, 616784 (3), Autosomal recessive
KBTBD13	613727	Nemaline myopathy 6, autosomal dominant, 609273 (3), Autosomal dominant
KCNA1	176260	Episodic ataxia/myokymia syndrome, 160120 (3), Autosomal dominant
KCNA2	176262	Developmental and epileptic encephalopathy 32, 616366 (3), Autosomal dominant
KCNA4	176266	Microcephaly, cataracts, impaired intellectual development, and dystonia with abnormal striatum, 618284 (3), Autosomal recessive
KCNA5	176267	Atrial fibrillation, familial, 7, 612240 (3), Autosomal dominant
KCNB1	600397	Developmental and epileptic encephalopathy 26, 616056 (3), Autosomal dominant
KCNC1	176258	Epilepsy, progressive myoclonic 7, 616187 (3), Autosomal dominant
KCNC2	176256	Developmental and epileptic encephalopathy 103, 619913 (3), Autosomal dominant
KCNC3	176264	Spinocerebellar ataxia 13, 605259 (3), Autosomal dominant
KCND3	605411	Spinocerebellar ataxia 19, 607346 (3), Autosomal dominant; Brugada syndrome 9, 616399 (3), Autosomal dominant
KCNE1	176261	Jervell and Lange-Nielsen syndrome 2, 612347 (3), Autosomal recessive; Long QT syndrome 5, 613695 (3), Autosomal dominant
KCNE2	603796	Long QT syndrome 6, 613693 (3), Autosomal dominant; Atrial fibrillation, familial, 4, 611493 (3)
KCNE3	604433	?Brugada syndrome 6, 613119 (3)
KCNH1	603305	Zimmermann-Laband syndrome 1, 135500 (3), Autosomal dominant; Temple-Baraitser syndrome, 611816 (3), Autosomal dominant
KCNH2	152427	Short QT syndrome 1, 609620 (3); Long QT syndrome 2, 613688 (3), Autosomal dominant; {Long QT syndrome 2, acquired, susceptibility to}, 613688 (3), Autosomal dominant
KCNJ1	600359	Bartter syndrome, type 2, 241200 (3), Autosomal recessive
KCNJ10	602208	Enlarged vestibular aqueduct, digenic, 600791 (3), Autosomal recessive; SESAME syndrome, 612780 (3), Autosomal recessive
KCNJ11	600937	Diabetes, permanent neonatal 2, with or without neurologic features, 618856 (3), Autosomal dominant; {Diabetes mellitus, type 2, susceptibility to}, 125853 (3), Autosomal dominant; Maturity-onset diabetes of the young, type 13, 616329 (3), Autosomal dominant; Diabetes mellitus, transient neonatal 3, 610582 (3), Autosomal dominant; Hyperinsulinemic hypoglycemia, familial, 2, 601820 (3), Autosomal dominant, Autosomal recessive
KCNJ13	603208	Snowflake vitreoretinal degeneration, 193230 (3), Autosomal dominant; Leber congenital amaurosis 16, 614186 (3), Autosomal recessive
KCNJ16	605722	Hypokalemic tubulopathy and deafness, 619406 (3), Autosomal recessive
KCNJ18	613236	{Thyrotoxic periodic paralysis, susceptibility to, 2}, 613239 (3), Autosomal dominant

KCNJ2	600681	Atrial fibrillation, familial, 9, 613980 (3), Autosomal dominant; Andersen syndrome, 170390 (3), Autosomal dominant; Short QT syndrome 3, 609622 (3)
KCNJ5	600734	Long QT syndrome 13, 613485 (3), Autosomal dominant; Hyperaldosteronism, familial, type III, 613677 (3), Autosomal dominant
KCNJ6	600877	Keppen-Lubinsky syndrome, 614098 (3), Autosomal dominant
KCNK18	613655	{Migraine, with or without aura, susceptibility to, 13}, 613656 (3), Autosomal dominant
KCNK3	603220	Pulmonary hypertension, primary, 4, 615344 (3), Autosomal dominant
KCNK4	605720	Facial dysmorphism, hypertrichosis, epilepsy, intellectual/developmental delay, and gingival overgrowth syndrome, 618381 (3), Autosomal dominant
KCNK9	605874	Birk-Barel syndrome, 612292 (3)
KCNMA1	600150	{Epilepsy, idiopathic generalized, susceptibility to, 16}, 618596 (3), Autosomal dominant; Paroxysmal nonkinesigenic dyskinesia, 3, with or without generalized epilepsy, 609446 (3), Autosomal dominant; Cerebellar atrophy, developmental delay, and seizures, 617643 (3), Autosomal recessive; Liang-Wang syndrome, 618729 (3), Autosomal dominant
KCNMB1	603951	{Hypertension, diastolic, resistance to}, 608622 (3), Autosomal dominant
KCNN2	605879	?Dystonia 34, myoclonic, 619724 (3), Autosomal dominant; Neurodevelopmental disorder with or without variable movement or behavioral abnormalities, 619725 (3), Autosomal dominant
KCNN3	602983	Zimmermann-Laband syndrome 3, 618658 (3), Autosomal dominant
KCNN4	602754	Dehydrated hereditary stomatocytosis 2, 616689 (3), Autosomal dominant
KCNQ1	607542	Short QT syndrome 2, 609621 (3), Autosomal dominant; Atrial fibrillation, familial, 3, 607554 (3), Autosomal dominant; Long QT syndrome 1, 192500 (3), Autosomal dominant; {Long QT syndrome 1, acquired, susceptibility to}, 192500 (3), Autosomal dominant; Jervell and Lange-Nielsen syndrome, 220400 (3), Autosomal recessive
KCNQ1OT1	604115	Beckwith-Wiedemann syndrome, 130650 (3), Autosomal dominant
KCNQ2	602235	Developmental and epileptic encephalopathy 7, 613720 (3), Autosomal dominant; Seizures, benign neonatal, 1, 121200 (3), Autosomal dominant; Myokymia, 121200 (3), Autosomal dominant
KCNQ3	602232	Seizures, benign neonatal, 2, 121201 (3), Autosomal dominant
KCNQ4	603537	Deafness, autosomal dominant 2A, 600101 (3), Autosomal dominant
KCNQ5	607357	Intellectual developmental disorder, autosomal dominant 46, 617601 (3), Autosomal dominant
KCNT1	608167	Developmental and epileptic encephalopathy 14, 614959 (3), Autosomal dominant; Epilepsy nocturnal frontal lobe, 5, 615005 (3), Autosomal dominant
KCNT2	610044	Developmental and epileptic encephalopathy 57, 617771 (3), Autosomal dominant
KCNV2	607604	Retinal cone dystrophy 3B, 610356 (3), Autosomal recessive
KCTD1	613420	Scalp-ear-nipple syndrome, 181270 (3), Autosomal dominant

KCTD17	616386	Dystonia 26, myoclonic, 616398 (3), Autosomal dominant
KCTD7	611725	Epilepsy, progressive myoclonic 3, with or without intracellular inclusions, 611726 (3), Autosomal recessive
KDELR2	609024	Osteogenesis imperfecta, type XXI, 619131 (3), Autosomal recessive
KDF1	616758	?Ectodermal dysplasia 12, hypohidrotic/hair/tooth/nail type, 617337 (3), Autosomal dominant
KDM1A	609132	Cleft palate, psychomotor retardation, and distinctive facial features, 616728 (3), Autosomal dominant
KDM3B	609373	Diets-Jongmans syndrome, 618846 (3), Autosomal dominant
KDM4B	609765	Intellectual developmental disorder, autosomal dominant 65, 619320 (3), Autosomal dominant
KDM5B	605393	Intellectual developmental disorder, autosomal recessive 65, 618109 (3), Autosomal recessive
KDM5C	314690	Intellectual developmental disorder, X-linked syndromic, Claes-Jensen type, 300534 (3), X-linked recessive
KDM6A	300128	Kabuki syndrome 2, 300867 (3), X-linked dominant
KDM6B	611577	Neurodevelopmental disorder with coarse facies and mild distal skeletal abnormalities, 618505 (3), Autosomal dominant
KDR	191306	{Hemangioma, capillary infantile, susceptibility to}, 602089 (3), Autosomal dominant; Hemangioma, capillary infantile, somatic, 602089 (3)
KDSR	136440	Erythrokeratodermia variabilis et progressiva 4, 617526 (3), Autosomal recessive
KEL	613883	[Blood group, Kell], 110900 (3)
KERA	603288	Cornea plana 2, autosomal recessive, 217300 (3), Autosomal recessive
KHDC3L	611687	Hydatidiform mole, recurrent, 2, 614293 (3), Autosomal recessive
KHK	614058	?[Fructosuria, essential], 229800 (3), Autosomal recessive
KIAA0586	610178	Short-rib thoracic dysplasia 14 with polydactyly, 616546 (3), Autosomal recessive; Joubert syndrome 23, 616490 (3), Autosomal recessive
KIAA0753	617112	?Orofaciodigital syndrome XV, 617127 (3), Autosomal recessive; ?Joubert syndrome 38, 619476 (3), Autosomal recessive; Short-rib thoracic dysplasia 21 without polydactyly, 619479 (3), Autosomal recessive
KIAA0825	617266	Polydactyly, postaxial, type A10, 618498 (3), Autosomal recessive
KIAA1549	613344	Retinitis pigmentosa 86, 618613 (3), Autosomal recessive
KIDINS220	615759	Spastic paraplegia, intellectual disability, nystagmus, and obesity, 617296 (3), Autosomal dominant; Ventriculomegaly and arthrogryposis, 619501 (3), Autosomal recessive
KIF11	148760	Microcephaly with or without chorioretinopathy, lymphedema, or mental retardation, 152950 (3), Autosomal dominant
KIF12	611278	Cholestasis, progressive familial intrahepatic, 8, 619662 (3), Autosomal recessive
KIF14	611279	Microcephaly 20, primary, autosomal recessive, 617914 (3), Autosomal recessive; ?Meckel syndrome 12, 616258 (3), Autosomal recessive
KIF15	617569	?Braddock-Carey syndrome 2, 619981 (3), Autosomal recessive

KIF1A	601255	NESCAV syndrome, 614255 (3), Autosomal dominant; Neuropathy, hereditary sensory, type IIC, 614213 (3), Autosomal recessive; Spastic paraplegia 30, autosomal dominant, 610357 (3), Autosomal dominant, Autosomal recessive; Spastic paraplegia 30, autosomal recessive, 610357 (3), Autosomal dominant, Autosomal recessive
KIF1B	605995	Pheochromocytoma, 171300 (3), Autosomal dominant; {Neuroblastoma, susceptibility to, 1}, 256700 (3), Autosomal dominant, Somatic mutation; Charcot-Marie-Tooth disease, type 2A1, 118210 (3), Autosomal dominant
KIF1C	603060	Spastic ataxia 2, autosomal recessive, 611302 (3), Autosomal recessive
KIF20A	605664	?Cardiomyopathy, familial restrictive, 6, 619433 (3), Autosomal recessive
KIF21A	608283	Fibrosis of extraocular muscles, congenital, 3B, 135700 (3), Autosomal dominant; Fibrosis of extraocular muscles, congenital, 1, 135700 (3), Autosomal dominant
KIF22	603213	Spondyloepimetaphyseal dysplasia with joint laxity, type 2, 603546 (3), Autosomal dominant
KIF23	605064	Anemia, congenital dyserythropoietic, type IIIA, 105600 (3), Autosomal dominant
KIF2A	602591	Cortical dysplasia, complex, with other brain malformations 3, 615411 (3), Autosomal dominant
KIF3B	603754	Retinitis pigmentosa 89, 618955 (3), Autosomal dominant
KIF4A	300521	?Intellectual developmental disorder, X-linked 100, 300923 (3), X-linked recessive
KIF5A	602821	Myoclonus, intractable, neonatal, 617235 (3), Autosomal dominant; {Amyotrophic lateral sclerosis, susceptibility to, 25}, 617921 (3), Autosomal dominant; Spastic paraplegia 10, autosomal dominant, 604187 (3), Autosomal dominant
KIF5C	604593	Cortical dysplasia, complex, with other brain malformations 2, 615282 (3), Autosomal dominant
KIF7	611254	Joubert syndrome 12, 200990 (3), Autosomal recessive; Acrocallosal syndrome, 200990 (3), Autosomal recessive; ?Hydrolethalus syndrome 2, 614120 (3), Autosomal recessive; ?Al-Gazali-Bakalinova syndrome, 607131 (3), Autosomal recessive
KIFBP	609367	Goldberg-Shprintzen megacolon syndrome, 609460 (3), Autosomal recessive
KIR3DL1	604946	{AIDS, delayed/rapid progression to}, 609423 (3)
KIRREL1	607428	Nephrotic syndrome, type 23, 619201 (3), Autosomal recessive
KISS1	603286	?Hypogonadotropic hypogonadism 13 with or without anosmia, 614842 (3), Autosomal recessive
KISS1R	604161	Hypogonadotropic hypogonadism 8 with or without anosmia, 614837 (3), Autosomal recessive; ?Precocious puberty, central, 1, 176400 (3), Autosomal dominant

KIT	164920	Gastrointestinal stromal tumor, familial, 606764 (3), Autosomal dominant, Isolated cases; Mastocytosis, cutaneous, 154800 (3), Autosomal dominant; Piebaldism, 172800 (3), Autosomal dominant; Germ cell tumors, somatic, 273300 (3); Mastocytosis, systemic, somatic, 154800 (3); Leukemia, acute myeloid, somatic, 601626 (3)
KITLG	184745	Hyperpigmentation with or without hypopigmentation, 145250 (3), Autosomal dominant; Waardenburg syndrome, type 2F, 619947 (3), Autosomal recessive; Deafness, autosomal dominant 69, unilateral or asymmetric, 616697 (3), Autosomal dominant; [Skin/hair/eye pigmentation 7, blond/brown hair], 611664 (3)
KIZ	615757	Retinitis pigmentosa 69, 615780 (3), Autosomal recessive
KL	604824	?Tumoral calcinosis, hyperphosphatemic, familial, 3, 617994 (3), Autosomal recessive
KLC2	611729	Spastic paraplegia, optic atrophy, and neuropathy, 609541 (3), Autosomal recessive
KLF1	600599	Blood group--Lutheran inhibitor, 111150 (3); Dyserythropoietic anemia, congenital, type IV, 613673 (3), Autosomal dominant; [Hereditary persistence of fetal hemoglobin], 613566 (3)
KLF11	603301	Maturity-onset diabetes of the young, type VII, 610508 (3)
KLF6	602053	Gastric cancer, somatic, 613659 (3); Prostate cancer, somatic, 176807 (3)
KLHDC8B	613169	{Hodgkin lymphoma, susceptibility to}, 236000 (3), Autosomal recessive
KLHL10	608778	Spermatogenic failure 11, 615081 (3), Autosomal dominant
KLHL15	300980	Intellectual developmental disorder, X-linked 103, 300982 (3), X-linked recessive
KLHL24	611295	Epidermolysis bullosa simplex 6, generalized, with scarring and hair loss, 617294 (3), Autosomal dominant
KLHL3	605775	Pseudohypoaldosteronism, type IID, 614495 (3), Autosomal dominant, Autosomal recessive
KLHL40	615340	Nemaline myopathy 8, autosomal recessive, 615348 (3), Autosomal recessive
KLHL41	607701	Nemaline myopathy 9, 615731 (3), Autosomal recessive
KLHL7	611119	Retinitis pigmentosa 42, 612943 (3), Autosomal dominant; PERCHING syndrome, 617055 (3), Autosomal recessive
KLK1	147910	[Kallikrein, decreased urinary activity of], 615953 (3)
KLK4	603767	Amelogenesis imperfecta, type IIA1, 204700 (3), Autosomal recessive
KLKB1	229000	Fletcher factor (prekallikrein) deficiency, 612423 (3), Autosomal recessive
KLLN	612105	Cowden syndrome 4, 615107 (3)
KMT2A	159555	Wiedemann-Steiner syndrome, 605130 (3), Autosomal dominant
KMT2B	606834	Intellectual developmental disorder, autosomal dominant 68, 619934 (3), Autosomal dominant; Dystonia 28, childhood-onset, 617284 (3), Autosomal dominant
KMT2C	606833	Kleefstra syndrome 2, 617768 (3), Autosomal dominant
KMT2D	602113	Kabuki syndrome 1, 147920 (3), Autosomal dominant
KMT2E	608444	O'Donnell-Luria-Rodan syndrome, 618512 (3), Autosomal dominant

KMT5B	610881	Intellectual developmental disorder, autosomal dominant 51, 617788 (3), Autosomal dominant
KNG1	612358	[Kininogen deficiency], 228960 (3), Autosomal recessive; Angioedema, hereditary, 6, 619363 (3), Autosomal dominant; [High molecular weight kininogen deficiency], 228960 (3), Autosomal recessive
KNL1	609173	Microcephaly 4, primary, autosomal recessive, 604321 (3), Autosomal recessive
KNSTRN	614718	?Roifman-Chitayat syndrome, digenic, 613328 (3), Digenic recessive
KPTN	615620	Intellectual developmental disorder, autosomal recessive 41, 615637 (3), Autosomal recessive
KRAS	190070	Gastric cancer, somatic, 613659 (3); Oculoectodermal syndrome, somatic, 600268 (3); Breast cancer, somatic, 114480 (3); Noonan syndrome 3, 609942 (3), Autosomal dominant; RAS-associated autoimmune leukoproliferative disorder, 614470 (3), Autosomal dominant; Arteriovenous malformation of the brain, somatic, 108010 (3); Lung cancer, somatic, 211980 (3); Pancreatic carcinoma, somatic, 260350 (3); Leukemia, acute myeloid, somatic, 601626 (3); Schimmelpenning-Feuerstein-Mims syndrome, somatic mosaic, 163200 (3); Cardiofaciocutaneous syndrome 2, 615278 (3), Autosomal dominant; Bladder cancer, somatic, 109800 (3)
KREMEN1	609898	Ectodermal dysplasia 13, hair/tooth type, 617392 (3), Autosomal recessive
KRIT1	604214	Hyperkeratotic cutaneous capillary-venous malformations associated with cerebral capillary malformations, 116860 (3), Autosomal dominant; Cerebral cavernous malformations-1, 116860 (3), Autosomal dominant; Cavernous malformations of CNS and retina, 116860 (3), Autosomal dominant
KRT1	139350	Ichthyosis, cyclic, with epidermolytic hyperkeratosis, 607602 (3), Autosomal dominant; Epidermolytic hyperkeratosis, 113800 (3), Autosomal dominant, Autosomal recessive; Palmoplantar keratoderma, nonepidermolytic, 600962 (3), Autosomal dominant; Keratosis palmoplantaris striata III, 607654 (3); Palmoplantar keratoderma, epidermolytic, 144200 (3), Autosomal dominant; Ichthyosis histrix, Curth-Macklin type, 146590 (3), Autosomal dominant
KRT10	148080	Epidermolytic hyperkeratosis, 113800 (3), Autosomal dominant, Autosomal recessive; Ichthyosis with confetti, 609165 (3), Autosomal dominant; Ichthyosis, cyclic, with epidermolytic hyperkeratosis, 607602 (3), Autosomal dominant
KRT12	601687	Meesmann corneal dystrophy 1, 122100 (3), Autosomal dominant
KRT13	148065	White sponge nevus 2, 615785 (3), Autosomal dominant

KRT14	148066	Epidermolysis bullosa simplex 1D, generalized, intermediate or severe, autosomal recessive, 601001 (3), Autosomal recessive; Epidermolysis bullosa simplex 1C, localized, 131800 (3), Autosomal dominant; Dermatopathia pigmentosa reticularis, 125595 (3), Autosomal dominant; Epidermolysis bullosa simplex 1A, generalized severe, 131760 (3), Autosomal dominant; Naegeli-Franceschetti-Jadassohn syndrome, 161000 (3), Autosomal dominant; Epidermolysis bullosa simplex 1B, generalized intermediate, 131900 (3), Autosomal dominant
KRT16	148067	Palmoplantar keratoderma, nonepidermolytic, focal, 613000 (3), Autosomal dominant; Pachyonychia congenita 1, 167200 (3), Autosomal dominant
KRT17	148069	Steatocystoma multiplex, 184500 (3), Autosomal dominant; Pachyonychia congenita 2, 167210 (3), Autosomal dominant
KRT18	148070	Cirrhosis, cryptogenic, 215600 (3), Autosomal recessive; {Cirrhosis, noncryptogenic, susceptibility to}, 215600 (3), Autosomal recessive
KRT2	600194	Ichthyosis bullosa of Siemens, 146800 (3), Autosomal dominant
KRT25	616646	Woolly hair, autosomal recessive 3, 616760 (3), Autosomal recessive
KRT3	148043	Meesmann corneal dystrophy 2, 618767 (3), Autosomal dominant
KRT4	123940	White sponge nevus 1, 193900 (3), Autosomal dominant
KRT5	148040	Epidermolysis bullosa simplex 2A, generalized severe, 619555 (3), Autosomal dominant; Dowling-Degos disease 1, 179850 (3), Autosomal dominant; Epidermolysis bullosa simplex 2F, with mottled pigmentation, 131960 (3), Autosomal dominant; Epidermolysis bullosa simplex 2D, generalized, intermediate or severe, autosomal recessive, 619599 (3), Autosomal recessive; Epidermolysis bullosa simplex 2B, generalized intermediate, 619588 (3), Autosomal dominant; Epidermolysis bullosa simplex 2C, localized, 619594 (3), Autosomal dominant; Epidermolysis bullosa simplex 2E, with migratory circinate erythema, 609352 (3), Autosomal dominant
KRT6A	148041	Pachyonychia congenita 3, 615726 (3), Autosomal dominant
KRT6B	148042	Pachyonychia congenita 4, 615728 (3), Autosomal dominant
KRT6C	612315	Palmoplantar keratoderma, nonepidermolytic, focal or diffuse, 615735 (3), Autosomal dominant
KRT71	608245	?Hypotrichosis 13, 615896 (3), Autosomal dominant
KRT74	608248	Woolly hair, autosomal dominant, 194300 (3), Autosomal dominant; ?Hypotrichosis 3, 613981 (3), Autosomal dominant; ?Ectodermal dysplasia 7, hair/nail type, 614929 (3), Autosomal recessive
KRT75	609025	{Pseudofolliculitis barbae, susceptibility to}, 612318 (3)
KRT8	148060	{Cirrhosis, noncryptogenic, susceptibility to}, 215600 (3), Autosomal recessive; Cirrhosis, cryptogenic, 215600 (3), Autosomal recessive
KRT81	602153	Monilethrix, 158000 (3), Autosomal dominant
KRT83	602765	Monilethrix, 158000 (3), Autosomal dominant; Erythrokeratodermia variabilis et progressiva 5, 617756 (3), Autosomal recessive
KRT85	602767	Ectodermal dysplasia 4, hair/nail type, 602032 (3), Autosomal recessive
KRT86	601928	Monilethrix, 158000 (3), Autosomal dominant

KRT9	607606	Palmoplantar keratoderma, epidermolytic, 144200 (3), Autosomal dominant
KY	605739	Myopathy, myofibrillar, 7, 617114 (3), Autosomal recessive
KYNU	605197	?Hydroxykynureninuria, 236800 (3), Autosomal recessive; Vertebral, cardiac, renal, and limb defects syndrome 2, 617661 (3), Autosomal recessive
L1CAM	308840	MASA syndrome, 303350 (3), X-linked recessive; Hydrocephalus with congenital idiopathic intestinal pseudoobstruction, 307000 (3), X-linked recessive; Corpus callosum, partial agenesis of, 304100 (3), X-linked recessive; CRASH syndrome, 303350 (3), X-linked recessive; Hydrocephalus with Hirschsprung disease, 307000 (3), X-linked recessive; Hydrocephalus due to aqueductal stenosis, 307000 (3), X-linked recessive
L2HGDH	609584	L-2-hydroxyglutaric aciduria, 236792 (3), Autosomal recessive
LACC1	613409	Juvenile arthritis, 618795 (3), Autosomal recessive
LAGE3	300060	Galloway-Mowat syndrome 2, X-linked, 301006 (3), X-linked recessive
LAMA1	150320	Poretti-Boltshauser syndrome, 615960 (3), Autosomal recessive
LAMA2	156225	Muscular dystrophy, limb-girdle, autosomal recessive 23, 618138 (3), Autosomal recessive; Muscular dystrophy, congenital, merosin deficient or partially deficient, 607855 (3), Autosomal recessive
LAMA3	600805	Epidermolysis bullosa, junctional 2A, intermediate, 619783 (3), Autosomal recessive; Epidermolysis bullosa, junctional 2C, laryngoonychocutaneous, 245660 (3), Autosomal recessive; Epidermolysis bullosa, junctional 2B, severe, 619784 (3), Autosomal recessive
LAMA4	600133	Cardiomyopathy, dilated, 1JJ, 615235 (3), Autosomal dominant
LAMB1	150240	Lissencephaly 5, 615191 (3), Autosomal recessive
LAMB2	150325	Nephrotic syndrome, type 5, with or without ocular abnormalities, 614199 (3); Pierson syndrome, 609049 (3), Autosomal recessive
LAMB3	150310	Epidermolysis bullosa, junctional 1B, severe, 226700 (3), Autosomal recessive; Epidermolysis bullosa, junctional 1A, intermediate, 226650 (3), Autosomal recessive; Amelogenesis imperfecta, type IA, 104530 (3), Autosomal dominant
LAMC2	150292	Epidermolysis bullosa, junctional 3B, severe, 619786 (3), Autosomal recessive; Epidermolysis bullosa, junctional 3A, intermediate, 619785 (3), Autosomal recessive
LAMC3	604349	Cortical malformations, occipital, 614115 (3), Autosomal recessive
LAMP2	309060	Danon disease, 300257 (3), X-linked dominant
LAMTOR2	610389	Immunodeficiency due to defect in MAPBP-interacting protein, 610798 (3), Autosomal recessive
LARGE1	603590	Muscular dystrophy-dystroglycanopathy (congenital with mental retardation), type B, 6, 608840 (3), Autosomal recessive; Muscular dystrophy-dystroglycanopathy (congenital with brain and eye anomalies), type A, 6, 613154 (3), Autosomal recessive
LARP7	612026	Alazami syndrome, 615071 (3), Autosomal recessive
LARS1	151350	?Infantile liver failure syndrome 1, 615438 (3), Autosomal recessive

LARS2	604544	Perrault syndrome 4, 615300 (3), Autosomal recessive; Hydrops, lactic acidosis, and sideroblastic anemia, 617021 (3), Autosomal recessive
LAS1L	300964	Wilson-Turner syndrome, 309585 (3), X-linked recessive
LAT	602354	Immunodeficiency 52, 617514 (3), Autosomal recessive
LBR	600024	Pelger-Huet anomaly, 169400 (3), Autosomal dominant; ?Reynolds syndrome, 613471 (3), Autosomal dominant; Rhizomelic skeletal dysplasia with or without Pelger-Huet anomaly, 618019 (3), Autosomal recessive; Greenberg skeletal dysplasia, 215140 (3), Autosomal recessive
LBX1	604255	?Central hypoventilation syndrome, congenital, 3, 619483 (3), Autosomal recessive
LCA5	611408	Leber congenital amaurosis 5, 604537 (3), Autosomal recessive
LCAT	606967	Fish-eye disease, 136120 (3), Autosomal recessive; Norum disease, 245900 (3), Autosomal recessive
LCK	153390	?Immunodeficiency 22, 615758 (3), Autosomal recessive
LCP2	601603	?Immunodeficiency 81, 619374 (3), Autosomal recessive
LCT	603202	Lactase deficiency, congenital, 223000 (3), Autosomal recessive
LDB3	605906	Left ventricular noncompaction 3, 601493 (3), Autosomal dominant; Cardiomyopathy, hypertrophic, 24, 601493 (3), Autosomal dominant; Myopathy, myofibrillar, 4, 609452 (3), Autosomal dominant; Cardiomyopathy, dilated, 1C, with or without LVNC, 601493 (3), Autosomal dominant
LDHA	150000	Glycogen storage disease XI, 612933 (3), Autosomal recessive
LDHB	150100	[Lactate dehydrogenase-B deficiency], 614128 (3)
LDHD	607490	D-lactic aciduria with susceptibility to gout, 245450 (3), Autosomal recessive
LDLR	606945	LDL cholesterol level QTL2, 143890 (3), Autosomal dominant, Autosomal recessive; Hypercholesterolemia, familial, 1, 143890 (3), Autosomal dominant, Autosomal recessive
LDLRAP1	605747	Hypercholesterolemia, familial, 4, 603813 (3), Autosomal recessive
LEF1	153245	Sebaceous tumors, somatic (3)
LEMD2	616312	Marbach-Rustad progeroid syndrome, 619322 (3), Autosomal dominant; Cataract 46, juvenile-onset, 212500 (3), Autosomal recessive
LEMD3	607844	Buschke-Ollendorff syndrome, 166700 (3), Autosomal dominant; Osteopoikilosis with or without melorheostosis, 166700 (3), Autosomal dominant
LEP	164160	Obesity, morbid, due to leptin deficiency, 614962 (3), Autosomal recessive
LEPR	601007	Obesity, morbid, due to leptin receptor deficiency, 614963 (3), Autosomal recessive
LFNG	602576	Spondylocostal dysostosis 3, autosomal recessive, 609813 (3), Autosomal recessive
LGALS2	150571	{Myocardial infarction, susceptibility to}, 608446 (3)
LGI1	604619	Epilepsy, familial temporal lobe, 1, 600512 (3), Autosomal dominant
LGI3	608302	Intellectual developmental disorder with muscle tone abnormalities and distal skeletal defects, 620007 (3)

LGI4	608303	Arthrogryposis multiplex congenita 1, neurogenic, with myelin defect, 617468 (3), Autosomal recessive
LGR4	606666	Delayed puberty, self-limited, 619613 (3), Autosomal dominant; {Bone mineral density, low, susceptibility to}, 615311 (3)
LHB	152780	Hypogonadotropic hypogonadism 23 with or without anosmia, 228300 (3), Autosomal recessive
LHCGR	152790	Leydig cell adenoma, somatic, with precocious puberty, 176410 (3); Leydig cell hypoplasia with pseudohermaphroditism, 238320 (3), Autosomal recessive; Leydig cell hypoplasia with hypergonadotropic hypogonadism, 238320 (3), Autosomal recessive; Luteinizing hormone resistance, female, 238320 (3), Autosomal recessive; Precocious puberty, male, 176410 (3), Autosomal dominant
LHFPL5	609427	Deafness, autosomal recessive 67, 610265 (3), Autosomal recessive
LHX3	600577	Pituitary hormone deficiency, combined, 3, 221750 (3), Autosomal recessive
LHX4	602146	Pituitary hormone deficiency, combined, 4, 262700 (3), Autosomal dominant
LIAS	607031	Hyperglycinemia, lactic acidosis, and seizures, 614462 (3), Autosomal recessive
LIFR	151443	Stuve-Wiedemann syndrome/Schwartz-Jampel type 2 syndrome, 601559 (3), Autosomal recessive
LIG1	126391	Immunodeficiency 96, 619774 (3), Autosomal recessive
LIG3	600940	Mitochondrial DNA depletion syndrome 20 (MNGIE type), 619780 (3), Autosomal recessive
LIG4	601837	LIG4 syndrome, 606593 (3), Autosomal recessive; {Multiple myeloma, resistance to}, 254500 (3), Somatic mutation
LIM2	154045	Cataract 19, multiple types, 615277 (3), Autosomal recessive
LIMA1	608364	[Low density lipoprotein cholesterol level QTL 8], 618079 (3)
LIMS2	607908	?Muscular dystrophy, autosomal recessive, with cardiomyopathy and triangular tongue, 616827 (3), Autosomal recessive
LINGO1	609791	Intellectual developmental disorder, autosomal recessive 64, 618103 (3), Autosomal recessive
LINS1	610350	Intellectual developmental disorder, autosomal recessive 27, 614340 (3), Autosomal recessive
LIPA	613497	Wolman disease, 278000 (3), Autosomal recessive; Cholesteryl ester storage disease, 278000 (3), Autosomal recessive
LIPC	151670	{Diabetes mellitus, noninsulin-dependent}, 125853 (3), Autosomal dominant; Hepatic lipase deficiency, 614025 (3), Autosomal recessive; [High density lipoprotein cholesterol level QTL 12], 612797 (3)
LIPE	151750	Lipodystrophy, familial partial, type 6, 615980 (3), Autosomal recessive
LIPH	607365	Hypotrichosis 7, 604379 (3), Autosomal recessive; Woolly hair, autosomal recessive 2 with or without hypotrichosis, 604379 (3), Autosomal recessive
LIPN	613924	Ichthyosis, congenital, autosomal recessive 8, 613943 (3), Autosomal recessive

LIPT1	610284	Lipoyltransferase 1 deficiency, 616299 (3), Autosomal recessive
LIPT2	617659	Encephalopathy, neonatal severe, with lactic acidosis and brain abnormalities, 617668 (3), Autosomal recessive
LITAF	603795	Charcot-Marie-Tooth disease, type 1C, 601098 (3), Autosomal dominant
LMAN1	601567	Combined factor V and VIII deficiency, 227300 (3), Autosomal recessive
LMAN2L	609552	?Intellectual developmental disorder, autosomal dominant 69, 617863 (3); ?Intellectual developmental disorder, autosomal recessive 52, 616887 (3), Autosomal recessive
LMBR1	605522	Triphalangeal thumb, type I, 174500 (3), Autosomal dominant; Syndactyly, type IV, 186200 (3), Autosomal dominant; Laurin-Sandrow syndrome, 135750 (3), Autosomal dominant; Hypoplastic or aplastic tibia with polydactyly, 188740 (3), Autosomal dominant; Polydactyly, preaxial type II, 174500 (3), Autosomal dominant; Acheiropody, 200500 (3), Autosomal recessive; Triphalangeal thumb-polysyndactyly syndrome, 174500 (3), Autosomal dominant
LMBRD1	612625	Methylmalonic aciduria and homocystinuria, cblF type, 277380 (3), Autosomal recessive
LMBRD2	619490	Developmental delay with variable neurologic and brain abnormalities, 619694 (3), Autosomal dominant
LMF1	611761	Lipase deficiency, combined, 246650 (3), Autosomal recessive
LMNA	150330	Mandibuloacral dysplasia, 248370 (3), Autosomal recessive; Heart-hand syndrome, Slovenian type, 610140 (3), Autosomal dominant; Cardiomyopathy, dilated, 1A, 115200 (3), Autosomal dominant; Emery-Dreifuss muscular dystrophy 3, autosomal recessive, 616516 (3), Autosomal recessive; Restrictive dermopathy 2, 619793 (3); Charcot-Marie-Tooth disease, type 2B1, 605588 (3), Autosomal recessive; Emery-Dreifuss muscular dystrophy 2, autosomal dominant, 181350 (3), Autosomal dominant; Hutchinson-Gilford progeria, 176670 (3), Autosomal dominant; Lipodystrophy, familial partial, type 2, 151660 (3), Autosomal dominant; Muscular dystrophy, congenital, 613205 (3), Autosomal dominant; Malouf syndrome, 212112 (3), Autosomal dominant
LMNB1	150340	Leukodystrophy, adult-onset, autosomal dominant, 169500 (3), Autosomal dominant; Microcephaly 26, primary, autosomal dominant, 619179 (3), Autosomal dominant
LMNB2	150341	Microcephaly 27, primary, autosomal dominant, 619180 (3), Autosomal dominant; ?Epilepsy, progressive myoclonic, 9, 616540 (3), Autosomal recessive; {Lipodystrophy, partial, acquired, susceptibility to}, 608709 (3), Autosomal dominant
LMO1	186921	Leukemia, T-cell acute lymphoblastic, 186921 (2)
LMO2	180385	Leukemia, acute T-cell, 180385 (2)
LMOD1	602715	?Megacystis-microcolon-intestinal hypoperistalsis syndrome 3, 619362 (3), Autosomal recessive
LMOD2	608006	Cardiomyopathy, dilated, 2G, 619897 (3), Autosomal recessive
LMOD3	616112	Nemaline myopathy 10, 616165 (3), Autosomal recessive

LMX1A	600298	Deafness, autosomal dominant 7, 601412 (3), Autosomal dominant
LMX1B	602575	Focal segmental glomerulosclerosis 10, 256020 (3), Autosomal dominant; Nail-patella syndrome, 161200 (3), Autosomal dominant
LNPK	610236	Neurodevelopmental disorder with epilepsy and hypoplasia of the corpus callosum, 618090 (3), Autosomal recessive
LONP1	605490	CODAS syndrome, 600373 (3), Autosomal recessive
LORICRIN	152445	Vohwinkel syndrome with ichthyosis, 604117 (3), Autosomal dominant
LOX	153455	Aortic aneurysm, familial thoracic 10, 617168 (3), Autosomal dominant
LOXHD1	613072	Deafness, autosomal recessive 77, 613079 (3), Autosomal recessive
LOXL1	153456	{Exfoliation syndrome, susceptibility to}, 177650 (3), Autosomal dominant
LOXL3	607163	Myopia 28, autosomal recessive, 619781 (3), Autosomal recessive
LPA	152200	[LPA deficiency, congenital], 618807 (3), Autosomal dominant; {Coronary artery disease, susceptibility to}, 618807 (3), Autosomal dominant
LPAR6	609239	Hypotrichosis 8, 278150 (3), Autosomal recessive; Woolly hair, autosomal recessive 1, with or without hypotrichosis, 278150 (3), Autosomal recessive
LPIN1	605518	Myoglobinuria, acute recurrent, autosomal recessive, 268200 (3), Autosomal recessive
LPIN2	605519	Majeed syndrome, 609628 (3)
LPL	609708	Lipoprotein lipase deficiency, 238600 (3), Autosomal recessive; [High density lipoprotein cholesterol level QTL 11], 238600 (3), Autosomal recessive; Combined hyperlipidemia, familial, 144250 (3), Autosomal dominant
LPP	600700	Lipoma (3); Leukemia, acute myeloid, 601626 (3), Autosomal dominant, Somatic mutation
LRAT	604863	Leber congenital amaurosis 14, 613341 (3), Autosomal recessive; Retinal dystrophy, early-onset severe, 613341 (3), Autosomal recessive; Retinitis pigmentosa, juvenile, 613341 (3), Autosomal recessive
LRBA	606453	Immunodeficiency, common variable, 8, with autoimmunity, 614700 (3), Autosomal recessive
LRIF1	615354	?Facioscapulohumeral muscular dystrophy 3, digenic, 619477 (3), Digenic recessive
LRIG2	608869	Urofacial syndrome 2, 615112 (3), Autosomal recessive
LRIT3	615004	Night blindness, congenital stationary (complete), 1F, autosomal recessive, 615058 (3), Autosomal recessive
LRMDA	614537	Albinism, oculocutaneous, type VII, 615179 (3), Autosomal recessive
LRP1	107770	?Keratosis pilaris atrophicans, 604093 (3), Autosomal recessive
LRP12	618299	Oculopharyngodistal myopathy 1, 164310 (3), Autosomal dominant
LRP2	600073	Donnai-Barrow syndrome, 222448 (3), Autosomal recessive
LRP4	604270	?Myasthenic syndrome, congenital, 17, 616304 (3), Autosomal recessive; Sclerosteosis 2, 614305 (3), Autosomal dominant, Autosomal recessive; Cenani-Lenz syndactyly syndrome, 212780 (3), Autosomal recessive

LRP5	603506	Osteopetrosis, autosomal dominant 1, 607634 (3), Autosomal dominant; [Bone mineral density variability 1], 601884 (3), Autosomal dominant; {Osteoporosis}, 166710 (3), Autosomal dominant; Hyperostosis, endosteal, 144750 (3), Autosomal dominant; Osteosclerosis, 144750 (3), Autosomal dominant; Polycystic liver disease 4 with or without kidney cysts, 617875 (3), Autosomal dominant; Osteoporosis-pseudoglioma syndrome, 259770 (3), Autosomal recessive; Exudative vitreoretinopathy 4, 601813 (3), Autosomal dominant, Autosomal recessive; van Buchem disease, type 2, 607636 (3)
LRP6	603507	{Coronary artery disease, autosomal dominant, 2}, 610947 (3), Autosomal dominant; Tooth agenesis, selective, 7, 616724 (3), Autosomal dominant
LRP8	602600	{Myocardial infarction, susceptibility to}, 608446 (3)
LRPAP1	104225	Myopia 23, autosomal recessive, 615431 (3), Autosomal recessive
LRPPRC	607544	Mitochondrial complex IV deficiency, nuclear type 5, (French-Canadian), 220111 (3), Autosomal recessive
LRRC32	137207	Cleft palate, proliferative retinopathy, and developmental delay, 619074 (3), Autosomal recessive
LRRC56	618227	Ciliary dyskinesia, primary, 39, 618254 (3), Autosomal recessive
LRRC8A	608360	?Agammaglobulinemia 5, 613506 (3), Autosomal dominant
LRRK1	610986	Osteosclerotic metaphyseal dysplasia, 615198 (3), Autosomal recessive
LRRK2	609007	{Parkinson disease 8}, 607060 (3), Autosomal dominant
LRSAM1	610933	Charcot-Marie-Tooth disease, axonal, type 2P, 614436 (3), Autosomal dominant, Autosomal recessive
LRTOMT	612414	Deafness, autosomal recessive 63, 611451 (3), Autosomal recessive
LSM11	617910	?Aicardi-Goutieres syndrome 8, 619486 (3), Autosomal recessive
LSS	600909	Hypotrichosis 14, 618275 (3), Autosomal recessive; Cataract 44, 616509 (3), Autosomal recessive; Alopecia-intellectual disability syndrome 4, 618840 (3), Autosomal recessive
LTA	153440	{Psoriatic arthritis, susceptibility to}, 607507 (3); {Myocardial infarction, susceptibility to}, 608446 (3); {Leprosy, susceptibility to, 4}, 610988 (3)
LTBP1	150390	Cutis laxa, autosomal recessive, type IIE, 619451 (3), Autosomal recessive
LTBP2	602091	Glaucoma 3, primary congenital, D, 613086 (3); Microspherophakia and/or megalocornea, with ectopia lentis and with or without secondary glaucoma, 251750 (3), Autosomal recessive; ?Weill-Marchesani syndrome 3, recessive, 614819 (3), Autosomal recessive
LTBP3	602090	Dental anomalies and short stature, 601216 (3), Autosomal recessive; Geleophysic dysplasia 3, 617809 (3), Autosomal dominant
LTBP4	604710	Cutis laxa, autosomal recessive, type IC, 613177 (3), Autosomal recessive
LTC4S	246530	Leukotriene C4 synthase deficiency, 614037 (1), Autosomal recessive
LYL1	151440	Leukemia, T-cell acute lymphoblastoid, 151440 (2)
LYRM4	613311	?Combined oxidative phosphorylation deficiency 19, 615595 (3), Autosomal recessive
LYRM7	615831	Mitochondrial complex III deficiency, nuclear type 8, 615838 (3), Autosomal recessive
LYST	606897	Chediak-Higashi syndrome, 214500 (3), Autosomal recessive

LYZ	153450	Amyloidosis, renal, 105200 (3), Autosomal dominant
LZTFL1	606568	Bardet-Biedl syndrome 17, 615994 (3), Autosomal recessive
LZTR1	600574	Noonan syndrome 2, 605275 (3), Autosomal recessive; Noonan syndrome 10, 616564 (3), Autosomal dominant; {Schwannomatosis-2, susceptibility to}, 615670 (3), Autosomal dominant
LZTS1	606551	Esophageal squamous cell carcinoma, somatic, 133239 (3)
M1AP	619098	Spermatogenic failure 48, 619108 (3), Autosomal recessive
MAB21L1	601280	Cerebellar, ocular, craniofacial, and genital syndrome, 618479 (3), Autosomal recessive
MAB21L2	604357	Microphthalmia/coloboma and skeletal dysplasia syndrome, 615877 (3), Autosomal dominant, Autosomal recessive
MACF1	608271	Lissencephaly 9 with complex brainstem malformation, 618325 (3), Autosomal dominant
MAD1L1	602686	Prostate cancer, somatic, 176807 (3); Lymphoma, somatic (3)
MAD2L2	604094	?Fanconi anemia, complementation group V, 617243 (3), Autosomal recessive
MADD	603584	Neurodevelopmental disorder with dysmorphic facies, impaired speech and hypotonia, 619005 (3), Autosomal recessive; DEEAH syndrome, 619004 (3), Autosomal recessive
MAF	177075	Cataract 21, multiple types, 610202 (3), Autosomal dominant; Ayme-Gripp syndrome, 601088 (3), Autosomal dominant
MAFA	610303	Insulinomatosis and diabetes mellitus, 147630 (3), Autosomal dominant
MAFB	608968	Duane retraction syndrome 3, 617041 (3), Autosomal dominant; Multicentric carpotarsal osteolysis syndrome, 166300 (3), Autosomal dominant
MAG	159460	Spastic paraplegia 75, autosomal recessive, 616680 (3), Autosomal recessive
MAGED2	300470	Bartter syndrome, type 5, antenatal, transient, 300971 (3), X-linked recessive
MAGEL2	605283	Schaaf-Yang syndrome, 615547 (3), Autosomal dominant
MAGI2	606382	Nephrotic syndrome, type 15, 617609 (3), Autosomal recessive
MAGT1	300715	Immunodeficiency, X-linked, with magnesium defect, Epstein-Barr virus infection and neoplasia, 300853 (3), X-linked recessive; Congenital disorder of glycosylation, type Icc, 301031 (3), X-linked recessive
MAK	154235	Retinitis pigmentosa 62, 614181 (3), Autosomal recessive
MALT1	604860	Immunodeficiency 12, 615468 (3), Autosomal recessive
MAML2	607537	Mucoepidermoid salivary gland carcinoma (3)
MAMLD1	300120	Hypospadias 2, X-linked, 300758 (3), X-linked recessive
MAN1B1	604346	Rafiq syndrome, 614202 (3), Autosomal recessive
MAN2B1	609458	Mannosidosis, alpha-, types I and II, 248500 (3), Autosomal recessive
MAN2C1	154580	Congenital disorder of deglycosylation 2, 619775 (3), Autosomal recessive
MANBA	609489	Mannosidosis, beta, 248510 (3), Autosomal recessive
MAOA	309850	{Antisocial behavior}, 300615 (3), X-linked recessive; Brunner syndrome, 300615 (3), X-linked recessive

MAP1B	157129	?Deafness, autosomal dominant 83, 619808 (3), Autosomal dominant; Periventricular nodular heterotopia 9, 618918 (3), Autosomal dominant
MAP2K1	176872	Cardiofaciocutaneous syndrome 3, 615279 (3), Autosomal dominant; Melorheostosis, isolated, somatic mosaic, 155950 (3)
MAP2K2	601263	Cardiofaciocutaneous syndrome 4, 615280 (3), Autosomal dominant
MAP3K1	600982	46XY sex reversal 6, 613762 (3), Autosomal dominant
MAP3K20	609479	Centronuclear myopathy 6 with fiber-type disproportion, 617760 (3), Autosomal recessive; Split-foot malformation with mesoaxial polydactyly, 616890 (3), Autosomal recessive
MAP3K7	602614	Frontometaphyseal dysplasia 2, 617137 (3), Autosomal dominant; Cardiospondylocarpofacial syndrome, 157800 (3), Autosomal dominant
MAP3K8	191195	Lung cancer, somatic, 211980 (3)
MAPK1	176948	Noonan syndrome 13, 619087 (3), Autosomal dominant
MAPK8IP1	604641	{Diabetes mellitus, noninsulin-dependent}, 125853 (3), Autosomal dominant
MAPK8IP3	605431	Neurodevelopmental disorder with or without variable brain abnormalities, 618443 (3), Autosomal dominant
MAPKAPK3	602130	?Macular dystrophy, patterned, 3, 617111 (3), Autosomal dominant
MAPKAPK5	606723	Neurocardiofaciodigital syndrome, 619869 (3), Autosomal recessive
MAPKBP1	616786	Nephronophthisis 20, 617271 (3), Autosomal recessive
MAPRE2	605789	Symmetric circumferential skin creases, congenital, 2, 616734 (3), Autosomal dominant
MAPT	157140	Supranuclear palsy, progressive, 601104 (3), Autosomal dominant; Supranuclear palsy, progressive atypical, 260540 (3), Autosomal recessive; Dementia, frontotemporal, with or without parkinsonism, 600274 (3), Autosomal dominant; {Parkinson disease, susceptibility to}, 168600 (3), Multifactorial, Autosomal dominant; Pick disease, 172700 (3), Autosomal dominant
MARCHF6	613297	Epilepsy, familial adult myoclonic, 3, 613608 (3), Autosomal dominant
MARK3	602678	?Visual impairment and progressive phthisis bulbi, 618283 (3), Autosomal recessive
MARS1	156560	Interstitial lung and liver disease, 615486 (3), Autosomal recessive; ?Trichothiodystrophy 9, nonphotosensitive, 619692 (3), Autosomal recessive; Charcot-Marie-Tooth disease, axonal, type 2U, 616280 (3), Autosomal dominant
MARS2	609728	?Combined oxidative phosphorylation deficiency 25, 616430 (3), Autosomal recessive; Spastic ataxia 3, autosomal recessive, 611390 (3), Autosomal recessive
MARVELD2	610572	Deafness, autosomal recessive 49, 610153 (3), Autosomal recessive
MASP1	600521	3MC syndrome 1, 257920 (3), Autosomal recessive
MASP2	605102	MASP2 deficiency, 613791 (3), Autosomal recessive
MAST1	612256	Mega-corpus-callosum syndrome with cerebellar hypoplasia and cortical malformations, 618273 (3), Autosomal dominant

MAT1A	610550	Hypermethioninemia, persistent, autosomal dominant, due to methionine adenosyltransferase I/III deficiency, 250850 (3), Autosomal dominant, Autosomal recessive; Methionine adenosyltransferase deficiency, autosomal recessive, 250850 (3), Autosomal dominant, Autosomal recessive
MATN3	602109	{Osteoarthritis susceptibility 2}, 140600 (3), Autosomal dominant; Spondyloepimetaphyseal dysplasia, Borochowitz-Cormier-Daire type, 608728 (3), Autosomal recessive; Epiphyseal dysplasia, multiple, 5, 607078 (3), Autosomal dominant
MATR3	164015	Amyotrophic lateral sclerosis 21, 606070 (3), Autosomal dominant
MAX	154950	{Pheochromocytoma, susceptibility to}, 171300 (3), Autosomal dominant
MBD4	603574	{Uveal melanoma, susceptibility to, 1}, 606660 (3), Autosomal dominant; Tumor predisposition syndrome 2, 619975 (3), Autosomal recessive
MBD5	611472	Intellectual developmental disorder, autosomal dominant 1, 156200 (3), Autosomal dominant
MBL2	154545	{Chronic infections, due to MBL deficiency}, 614372 (3), Autosomal dominant
MBOAT7	606048	Intellectual developmental disorder, autosomal recessive 57, 617188 (3), Autosomal recessive
MBTPS1	603355	?Spondyloepiphyseal dysplasia, Kondo-Fu type, 618392 (3), Autosomal recessive
MBTPS2	300294	Keratosis follicularis spinulosa decalvans, X-linked, 308800 (3), X-linked recessive; Osteogenesis imperfecta, type XIX, 301014 (3), X-linked recessive; IFAP syndrome with or without BRESHECK syndrome, 308205 (3), X-linked recessive; ?Olmsted syndrome, X-linked, 300918 (3), X-linked recessive
MC1R	155555	[Analgesia from kappa-opioid receptor agonist, female-specific], 613098 (3); [Skin/hair/eye pigmentation 2, red hair/fair skin], 266300 (3), Autosomal recessive; [Skin/hair/eye pigmentation 2, blond hair/fair skin], 266300 (3), Autosomal recessive; {Melanoma, cutaneous malignant, 5}, 613099 (3); {Albinism, oculocutaneous, type II, modifier of}, 203200 (3), Autosomal recessive; {UV-induced skin damage}, 266300 (3), Autosomal recessive
MC2R	607397	Glucocorticoid deficiency, due to ACTH unresponsiveness, 202200 (3), Autosomal recessive
MC3R	155540	{Obesity, severe, susceptibility to, BMIQ9}, 602025 (3)
MC4R	155541	Obesity (BMIQ20), 618406 (3), Autosomal dominant, Autosomal recessive; {Obesity, resistance to (BMIQ20)}, 618406 (3), Autosomal dominant, Autosomal recessive
MCC	159350	Colorectal cancer, somatic, 114500 (3)
MCCC1	609010	3-Methylcrotonyl-CoA carboxylase 1 deficiency, 210200 (3), Autosomal recessive
MCCC2	609014	3-Methylcrotonyl-CoA carboxylase 2 deficiency, 210210 (3), Autosomal recessive
MCEE	608419	Methylmalonyl-CoA epimerase deficiency, 251120 (3), Autosomal recessive

MCFD2	607788	Factor V and factor VIII, combined deficiency of, 613625 (3)
MCIDAS	614086	Ciliary dyskinesia, primary, 42, 618695 (3), Autosomal recessive
MCM10	609357	Immunodeficiency 80 with or without cardiomyopathy, 619313 (3), Autosomal recessive
MCM2	116945	?Deafness, autosomal dominant 70, 616968 (3), Autosomal dominant
MCM3AP	603294	Peripheral neuropathy, autosomal recessive, with or without impaired intellectual development, 618124 (3), Autosomal recessive
MCM4	602638	Immunodeficiency 54, 609981 (3), Autosomal recessive
MCM5	602696	?Meier-Gorlin syndrome 8, 617564 (3), Autosomal recessive
MCM6	601806	Lactase persistence/nonpersistence, 223100 (3), Autosomal dominant
MCM8	608187	?Premature ovarian failure 10, 612885 (3), Autosomal recessive
MCM9	610098	Ovarian dysgenesis 4, 616185 (3), Autosomal recessive
MCOLN1	605248	Mucolipidosis IV, 252650 (3), Autosomal recessive
MCPH1	607117	Microcephaly 1, primary, autosomal recessive, 251200 (3), Autosomal recessive
MDFIC	614511	Lymphatic malformation 12, 620014 (3)
MDH1	154200	?Developmental and epileptic encephalopathy 88, 618959 (3), Autosomal recessive
MDH2	154100	Developmental and epileptic encephalopathy 51, 617339 (3), Autosomal recessive
MDM2	164785	{Accelerated tumor formation, susceptibility to}, 614401 (3), Autosomal dominant; ?Lessel-Kubisch syndrome, 618681 (3), Autosomal recessive
MDM4	602704	?Bone marrow failure syndrome 6, 618849 (3), Autosomal dominant
MECOM	165215	Radioulnar synostosis with amegakaryocytic thrombocytopenia 2, 616738 (3), Autosomal dominant
MECP2	300005	Rett syndrome, atypical, 312750 (3), X-linked dominant; Encephalopathy, neonatal severe, 300673 (3), X-linked recessive; Intellectual developmental disorder, X-linked syndromic, Lubs type, 300260 (3), X-linked recessive; {Autism susceptibility, X-linked 3}, 300496 (3), X-linked; Intellectual developmental disorder, X-linked syndromic 13, 300055 (3), X-linked recessive; Rett syndrome, 312750 (3), X-linked dominant; Rett syndrome, preserved speech variant, 312750 (3), X-linked dominant
MECR	608205	Dystonia, childhood-onset, with optic atrophy and basal ganglia abnormalities, 617282 (3), Autosomal recessive
MED12	300188	Lujan-Fryns syndrome, 309520 (3), X-linked recessive; Ohdo syndrome, X-linked, 300895 (3), X-linked recessive; Hardikar syndrome, 301068 (3), X-linked dominant; Opitz-Kaveggia syndrome, 305450 (3), X-linked recessive
MED12L	611318	Nizon-Isidor syndrome, 618872 (3), Autosomal dominant
MED13	603808	Intellectual developmental disorder, autosomal dominant 61, 618009 (3), Autosomal dominant
MED13L	608771	Impaired intellectual development and distinctive facial features with or without cardiac defects, 616789 (3), Autosomal dominant
MED17	603810	Microcephaly, postnatal progressive, with seizures and brain atrophy, 613668 (3), Autosomal recessive

MED23	605042	Intellectual developmental disorder, autosomal recessive 18, with or without epilepsy, 614249 (3), Autosomal recessive
MED25	610197	Basel-Vanagait-Smirin-Yosef syndrome, 616449 (3), Autosomal recessive
MED27	605044	Neurodevelopmental disorder with spasticity, cataracts, and cerebellar hypoplasia, 619286 (3), Autosomal recessive
MEF2A	600660	{Coronary artery disease, autosomal dominant, 1}, 608320 (3), Autosomal dominant
MEF2C	600662	Chromosome 5q14.3 deletion syndrome, 613443 (4), Autosomal dominant; Neurodevelopmental disorder with hypotonia, stereotypic hand movements, and impaired language, 613443 (3), Autosomal dominant
MEFV	608107	Neutrophilic dermatosis, acute febrile, 608068 (3), Autosomal dominant; Familial Mediterranean fever, AR, 249100 (3), Autosomal recessive; Familial Mediterranean fever, AD, 134610 (3), Autosomal dominant
MEGF10	612453	Myopathy, areflexia, respiratory distress, and dysphagia, early-onset, mild variant, 614399 (3), Autosomal recessive; Myopathy, areflexia, respiratory distress, and dysphagia, early-onset, 614399 (3), Autosomal recessive
MEGF8	604267	Carpenter syndrome 2, 614976 (3), Autosomal recessive
MEI1	608797	Hydatidiform mole, recurrent, 3, 618431 (3), Autosomal recessive
MEIOB	617670	?Spermatogenic failure 22, 617706 (3), Autosomal recessive
MEIS2	601740	Cleft palate, cardiac defects, and mental retardation, 600987 (3), Autosomal dominant
MEN1	613733	Lipoma, somatic (3); Angiofibroma, somatic (3); Multiple endocrine neoplasia 1, 131100 (3), Autosomal dominant; Carcinoid tumor of lung (3); Adrenal adenoma, somatic (3); Parathyroid adenoma, somatic (3)
MEOX1	600147	Klippel-Feil syndrome 2, 214300 (3), Autosomal recessive
MERTK	604705	Retinitis pigmentosa 38, 613862 (3), Autosomal recessive
MESD	607783	Osteogenesis imperfecta, type XX, 618644 (3), Autosomal recessive
MESP2	605195	Spondylocostal dysostosis 2, autosomal recessive, 608681 (3), Autosomal recessive
MET	164860	Renal cell carcinoma, papillary, 1, familial and somatic, 605074 (3); Hepatocellular carcinoma, childhood type, somatic, 114550 (3); {Osteofibrous dysplasia, susceptibility to}, 607278 (3), Autosomal dominant; ?Deafness, autosomal recessive 97, 616705 (3), Autosomal recessive
METTL13	617987	{?Deafness, autosomal recessive 26, modifier of}, 605429 (3), Autosomal dominant
METTL23	615262	Intellectual developmental disorder, autosomal recessive 44, 615942 (3), Autosomal recessive
METTL5	618628	Intellectual developmental disorder, autosomal recessive 72, 618665 (3), Autosomal recessive
MFAP5	601103	Aortic aneurysm, familial thoracic 9, 616166 (3), Autosomal dominant
MFF	614785	Encephalopathy due to defective mitochondrial and peroxisomal fission 2, 617086 (3), Autosomal recessive
MFHAS1	605352	Malignant fibrous histiocytoma, 605352 (2)

MFN2	608507	Charcot-Marie-Tooth disease, axonal, type 2A2A, 609260 (3), Autosomal dominant; Charcot-Marie-Tooth disease, axonal, type 2A2B, 617087 (3), Autosomal recessive; Hereditary motor and sensory neuropathy VIA, 601152 (3), Autosomal dominant
MFRP	606227	Microphthalmia, isolated 5, 611040 (3), Autosomal recessive; Nanophthalmos 2, 609549 (3)
MFSD2A	614397	Neurodevelopmental disorder with progressive microcephaly, spasticity, and brain abnormalities, 616486 (3), Autosomal recessive
MFSD8	611124	Macular dystrophy with central cone involvement, 616170 (3), Autosomal recessive; Ceroid lipofuscinosis, neuronal, 7, 610951 (3), Autosomal recessive
MGAT2	602616	Congenital disorder of glycosylation, type IIa, 212066 (3), Autosomal recessive
MGME1	615076	Mitochondrial DNA depletion syndrome 11, 615084 (3), Autosomal recessive
MGP	154870	Keutel syndrome, 245150 (3), Autosomal recessive
MIA3	613455	?Ondontochondrodysplasia 2 with hearing loss and diabetes, 619269 (3), Autosomal recessive
MIAT	611082	{Myocardial infarction, susceptibility to}, 608446 (3)
MIB1	608677	Left ventricular noncompaction 7, 615092 (3), Autosomal dominant
MICOS13	616658	Combined oxidative phosphorylation deficiency 37, 618329 (3), Autosomal recessive
MICU1	605084	Myopathy with extrapyramidal signs, 615673 (3), Autosomal recessive
MID1	300552	Opitz GBBB syndrome, 300000 (3), X-linked recessive
MID2	300204	?Intellectual developmental disorder, X-linked 101, 300928 (3), X-linked recessive
MIEF2	615498	?Combined oxidative phosphorylation deficiency 49, 619024 (3), Autosomal recessive
MIF	153620	{Rheumatoid arthritis, systemic juvenile, susceptibility to}, 604302 (3)
MINPP1	605391	{Thyroid carcinoma, follicular}, 188470 (3), Autosomal dominant, Somatic mutation; Pontocerebellar hypoplasia, type 16, 619527 (3), Autosomal recessive
MIP	154050	Cataract 15, multiple types, 615274 (3), Autosomal dominant
MIPEP	602241	Combined oxidative phosphorylation deficiency 31, 617228 (3), Autosomal recessive
MIR140	611894	Spondyloepiphyseal dysplasia, Nishimura type, 618618 (3), Autosomal dominant
MIR184	613146	EDICT syndrome, 614303 (3), Autosomal dominant
MIR204	610942	?Retinal dystrophy and iris coloboma with or without cataract, 616722 (3), Autosomal dominant
MIR2861	613405	[Bone mineral density QTL 15], 613418 (3), Autosomal dominant, Autosomal recessive
MIR96	611606	Deafness, autosomal dominant 50, 613074 (3), Autosomal dominant

MITF	156845	Waardenburg syndrome, type 2A, 193510 (3), Autosomal dominant; {Melanoma, cutaneous malignant, susceptibility to, 8}, 614456 (3); Tietz albinism-deafness syndrome, 103500 (3), Autosomal dominant; Waardenburg syndrome/ocular albinism, digenic, 103470 (3); COMMAD syndrome, 617306 (3), Autosomal recessive
MKKS	604896	McKusick-Kaufman syndrome, 236700 (3), Autosomal recessive; Bardet-Biedl syndrome 6, 605231 (3), Autosomal recessive
MKRN3	603856	Precocious puberty, central, 2, 615346 (3), Autosomal dominant
MKS1	609883	Bardet-Biedl syndrome 13, 615990 (3), Autosomal recessive; Meckel syndrome 1, 249000 (3), Autosomal recessive; Joubert syndrome 28, 617121 (3), Autosomal recessive
MLC1	605908	Megalencephalic leukoencephalopathy with subcortical cysts, 604004 (3), Autosomal recessive
MLH1	120436	Colorectal cancer, hereditary nonpolyposis, type 2, 609310 (3); Muir-Torre syndrome, 158320 (3), Autosomal dominant; Mismatch repair cancer syndrome 1, 276300 (3), Autosomal recessive
MLH3	604395	{Endometrial cancer, susceptibility to}, 608089 (3), Autosomal dominant, Somatic mutation; Colorectal cancer, somatic, 114500 (3); Colorectal cancer, hereditary nonpolyposis, type 7, 614385 (3)
MLLT10	602409	Leukemia, acute myeloid, 601626 (3), Autosomal dominant, Somatic mutation
MLPH	606526	Griscelli syndrome, type 3, 609227 (3), Autosomal recessive
MLYCD	606761	Malonyl-CoA decarboxylase deficiency, 248360 (3), Autosomal recessive
MMAA	607481	Methylmalonic aciduria, vitamin B12-responsive, cblA type, 251100 (3), Autosomal recessive
MMAB	607568	Methylmalonic aciduria, vitamin B12-responsive, cblB type, 251110 (3), Autosomal recessive
MMACHC	609831	Methylmalonic aciduria and homocystinuria, cblC type, 277400 (3), Autosomal recessive
MMADHC	611935	Methylmalonic aciduria, cblD type, variant 2, 277410 (3), Autosomal recessive; Methylmalonic aciduria and homocystinuria, cblD type, 277410 (3), Autosomal recessive; Homocystinuria, cblD type, variant 1, 277410 (3), Autosomal recessive
MME	120520	?Spinocerebellar ataxia 43, 617018 (3), Autosomal dominant; Charcot-Marie-Tooth disease, axonal, type 2T, 617017 (3), Autosomal dominant, Autosomal recessive
MMP1	120353	COPD, rate of decline of lung function in, 606963 (3); {Epidermolysis bullosa dystrophica, autosomal recessive, modifier of}, 226600 (3), Autosomal recessive
MMP13	600108	?Spondyloepimetaphyseal dysplasia, Missouri type, 602111 (3), Autosomal dominant; Metaphyseal anadysplasia 1, 602111 (3), Autosomal dominant; Metaphyseal dysplasia, Spahr type, 250400 (3), Autosomal recessive
MMP14	600754	?Winchester syndrome, 277950 (3)
MMP19	601807	Cavitary optic disc anomalies, 611543 (3), Autosomal dominant

MMP2	120360	Multicentric osteolysis, nodulosis, and arthropathy, 259600 (3), Autosomal recessive
MMP20	604629	Amelogenesis imperfecta, type IIA2, 612529 (3), Autosomal recessive
MMP21	608416	Heterotaxy, visceral, 7, autosomal, 616749 (3), Autosomal recessive
MMP3	185250	{Coronary heart disease, susceptibility to, 6}, 614466 (3)
MMP9	120361	Metaphyseal anadysplasia 2, 613073 (3), Autosomal recessive
MMUT	609058	Methylmalonic aciduria, mut(0) type, 251000 (3), Autosomal recessive
MN1	156100	CEBALID syndrome, 618774 (3), Autosomal dominant; Meningioma, 607174 (3), Autosomal dominant
MNS1	610766	Heterotaxy, visceral, 9, autosomal, with male infertility, 618948 (3), Autosomal recessive
MNX1	142994	Currarino syndrome, 176450 (3), Autosomal dominant
MOCOS	613274	Xanthinuria, type II, 603592 (3), Autosomal recessive
MOCS1	603707	Molybdenum cofactor deficiency A, 252150 (3), Autosomal recessive
MOCS2	603708	Molybdenum cofactor deficiency B, 252160 (3), Autosomal recessive
MOG	159465	?Narcolepsy 7, 614250 (3), Autosomal dominant
MOGS	601336	Congenital disorder of glycosylation, type IIb, 606056 (3), Autosomal recessive
MORC2	616661	Charcot-Marie-Tooth disease, axonal, type 2Z, 616688 (3), Autosomal dominant; Developmental delay, impaired growth, dysmorphic facies, and axonal neuropathy, 619090 (3), Autosomal dominant
MOV10L1	605794	?Spermatogenic failure 73, 619878 (3), Autosomal recessive
MPC1	614738	Mitochondrial pyruvate carrier deficiency, 614741 (3), Autosomal recessive
MPDU1	604041	Congenital disorder of glycosylation, type If, 609180 (3), Autosomal recessive
MPDZ	603785	Hydrocephalus, congenital, 2, with or without brain or eye anomalies, 615219 (3), Autosomal recessive
MPEG1	610390	Immunodeficiency 77, 619223 (3), Autosomal dominant
MPI	154550	Congenital disorder of glycosylation, type Ib, 602579 (3), Autosomal recessive
MPIG6B	606520	?Thrombocytopenia, anemia, and myelofibrosis, 617441 (3), Autosomal recessive
MPL	159530	Myelofibrosis with myeloid metaplasia, somatic, 254450 (3); Thrombocythemia 2, 601977 (3), Autosomal dominant, Somatic mutation; Thrombocytopenia, congenital amegakaryocytic, 604498 (3), Autosomal recessive
MPLKIP	609188	Trichothiodystrophy 4, nonphotosensitive, 234050 (3), Autosomal recessive
MPO	606989	{Alzheimer disease, susceptibility to}, 104300 (3), Autosomal dominant; Myeloperoxidase deficiency, 254600 (3), Autosomal recessive; {Lung cancer, protection against, in smokers} (3)
MPV17	137960	Charcot-Marie-Tooth disease, axonal, type 2EE, 618400 (3), Autosomal recessive; Mitochondrial DNA depletion syndrome 6 (hepatocerebral type), 256810 (3), Autosomal recessive

MPZ	159440	Charcot-Marie-Tooth disease, type 2I, 607677 (3), Autosomal dominant; Dejerine-Sottas disease, 145900 (3), Autosomal dominant, Autosomal recessive; Charcot-Marie-Tooth disease, type 1B, 118200 (3), Autosomal dominant; Roussy-Levy syndrome, 180800 (3), Autosomal dominant; Charcot-Marie-Tooth disease, dominant intermediate D, 607791 (3), Autosomal dominant; Hypomyelinating neuropathy, congenital, 2, 618184 (3), Autosomal dominant; Charcot-Marie-Tooth disease, type 2J, 607736 (3), Autosomal dominant
MPZL2	604873	Deafness, autosomal recessive 111, 618145 (3), Autosomal recessive
MRAP	609196	Glucocorticoid deficiency 2, 607398 (3), Autosomal recessive
MRAP2	615410	{?Obesity, susceptibility to, BMIQ18}, 615457 (3), Autosomal dominant
MRAS	608435	Noonan syndrome 11, 618499 (3), Autosomal dominant
MRE11	600814	Ataxia-telangiectasia-like disorder 1, 604391 (3), Autosomal recessive
MRM2	606906	?Mitochondrial DNA depletion syndrome 17, 618567 (3), Autosomal recessive
MRPL12	602375	?Combined oxidative phosphorylation deficiency 45, 618951 (3), Autosomal recessive
MRPL3	607118	Combined oxidative phosphorylation deficiency 9, 614582 (3), Autosomal recessive
MRPL44	611849	Combined oxidative phosphorylation deficiency 16, 615395 (3), Autosomal recessive
MRPS14	611978	?Combined oxidative phosphorylation deficiency 38, 618378 (3), Autosomal recessive
MRPS16	609204	Combined oxidative phosphorylation deficiency 2, 610498 (3), Autosomal recessive
MRPS2	611971	Combined oxidative phosphorylation deficiency 36, 617950 (3), Autosomal recessive
MRPS22	605810	Ovarian dysgenesis 7, 618117 (3), Autosomal recessive; Combined oxidative phosphorylation deficiency 5, 611719 (3), Autosomal recessive
MRPS23	611985	?Combined oxidative phosphorylation deficiency 46, 618952 (3), Autosomal recessive
MRPS25	611987	?Combined oxidative phosphorylation deficiency 50, 619025 (3), Autosomal recessive
MRPS28	611990	?Combined oxidative phosphorylation deficiency 47, 618958 (3), Autosomal recessive
MRPS34	611994	Combined oxidative phosphorylation deficiency 32, 617664 (3), Autosomal recessive
MRPS7	611974	?Combined oxidative phosphorylation deficiency 34, 617872 (3), Autosomal recessive
MRTFA	606078	?Immunodeficiency 66, 618847 (3), Autosomal recessive
MS4A1	112210	?Immunodeficiency, common variable, 5, 613495 (3), Autosomal recessive
MS4A2	147138	{Atopy, susceptibility to}, 147050 (3), Autosomal dominant

MSH2	609309	Muir-Torre syndrome, 158320 (3), Autosomal dominant; Colorectal cancer, hereditary nonpolyposis, type 1, 120435 (3), Autosomal dominant; Mismatch repair cancer syndrome 2, 619096 (3), Autosomal recessive
MSH3	600887	Familial adenomatous polyposis 4, 617100 (3), Autosomal recessive; Endometrial carcinoma, somatic, 608089 (3)
MSH4	602105	Premature ovarian failure 20, 619938 (3), Autosomal recessive; Spermatogenic failure 2, 108420 (3), Autosomal recessive
MSH5	603382	?Premature ovarian failure 13, 617442 (3), Autosomal recessive; Spermatogenic failure 74, 619937 (3), Autosomal recessive
MSH6	600678	Colorectal cancer, hereditary nonpolyposis, type 5, 614350 (3), Autosomal dominant; Mismatch repair cancer syndrome 3, 619097 (3), Autosomal recessive; {Endometrial cancer, familial}, 608089 (3), Autosomal dominant, Somatic mutation
MSL3	300609	Basilicata-Akhtar syndrome, 301032 (3), X-linked dominant
MSMB	157145	{Prostate cancer, hereditary, 13}, 611928 (3)
MSMO1	607545	Microcephaly, congenital cataract, and psoriasiform dermatitis, 616834 (3), Autosomal recessive
MSN	309845	Immunodeficiency 50, 300988 (3), X-linked recessive
MSR1	153622	Barrett esophagus/esophageal adenocarcinoma, 614266 (3)
MSRB3	613719	Deafness, autosomal recessive 74, 613718 (3), Autosomal recessive
MST1R	600168	{Nasopharyngeal carcinoma, susceptibility to, 3}, 617075 (3), Autosomal dominant
MSTN	601788	?Muscle hypertrophy, 614160 (3), Autosomal recessive
MSTO1	617619	Myopathy, mitochondrial, and ataxia, 617675 (3), Autosomal dominant, Autosomal recessive
MSX1	142983	Tooth agenesis, selective, 1, with or without orofacial cleft, 106600 (3), Autosomal dominant; Ectodermal dysplasia 3, Witkop type, 189500 (3), Autosomal dominant; Orofacial cleft 5, 608874 (3), Autosomal dominant
MSX2	123101	Parietal foramina with cleidocranial dysplasia, 168550 (3), Autosomal dominant; Craniosynostosis 2, 604757 (3), Autosomal dominant; Parietal foramina 1, 168500 (3), Autosomal dominant
MTAP	156540	Diaphyseal medullary stenosis with malignant fibrous histiocytoma, 112250 (3), Autosomal dominant
MTFMT	611766	Combined oxidative phosphorylation deficiency 15, 614947 (3), Autosomal recessive; Mitochondrial complex I deficiency, nuclear type 27, 618248 (3), Autosomal recessive
MTHFD1	172460	{Neural tube defects, folate-sensitive, susceptibility to}, 601634 (3), Autosomal recessive; Combined immunodeficiency and megaloblastic anemia with or without hyperhomocysteinemia, 617780 (3), Autosomal recessive

MTHFR	607093	{Vascular disease, susceptibility to} (3); Homocystinuria due to MTHFR deficiency, 236250 (3), Autosomal recessive; {Thromboembolism, susceptibility to}, 188050 (3), Autosomal dominant; {Schizophrenia, susceptibility to}, 181500 (3), Autosomal dominant; {Neural tube defects, susceptibility to}, 601634 (3), Autosomal recessive
MTHFS	604197	Neurodevelopmental disorder with microcephaly, epilepsy, and hypomyelination, 618367 (3), Autosomal recessive
MTM1	300415	Myopathy, centronuclear, X-linked, 310400 (3), X-linked recessive
MTMR14	611089	{Centronuclear myopathy, autosomal, modifier of}, 160150 (3), Autosomal dominant
MTMR2	603557	Charcot-Marie-Tooth disease, type 4B1, 601382 (3), Autosomal recessive
MTNR1B	600804	{Diabetes mellitus, type 2, susceptibility to}, 125853 (3), Autosomal dominant
MTO1	614667	Combined oxidative phosphorylation deficiency 10, 614702 (3), Autosomal recessive
MTOR	601231	Focal cortical dysplasia, type II, somatic, 607341 (3); Smith-Kingsmore syndrome, 616638 (3), Autosomal dominant
MTPAP	613669	?Spastic ataxia 4, autosomal recessive, 613672 (3), Autosomal recessive
MTR	156570	{Neural tube defects, folate-sensitive, susceptibility to}, 601634 (3), Autosomal recessive; Homocystinuria-megaloblastic anemia, cblG complementation type, 250940 (3), Autosomal recessive
MTRFR	613541	Spastic paraplegia 55, autosomal recessive, 615035 (3), Autosomal recessive; Combined oxidative phosphorylation deficiency 7, 613559 (3), Autosomal recessive
MTRR	602568	Homocystinuria-megaloblastic anemia, cbl E type, 236270 (3), Autosomal recessive; {Neural tube defects, folate-sensitive, susceptibility to}, 601634 (3), Autosomal recessive
MTTP	157147	{Metabolic syndrome, protection against}, 605552 (3), Autosomal dominant; Abetalipoproteinemia, 200100 (3), Autosomal recessive
MTX2	608555	Mandibuloacral dysplasia progeroid syndrome, 619127 (3), Autosomal recessive
MUC1	158340	Tubulointerstitial kidney disease, autosomal dominant, 2, 174000 (3), Autosomal dominant
MUC5B	600770	{Pulmonary fibrosis, idiopathic, susceptibility to}, 178500 (3), Autosomal dominant
MUC7	158375	{Asthma, protection against}, 600807 (3), Autosomal dominant
MUSK	601296	Fetal akinesia deformation sequence 1, 208150 (3), Autosomal recessive; Myasthenic syndrome, congenital, 9, associated with acetylcholine receptor deficiency, 616325 (3), Autosomal recessive
MUTYH	604933	Adenomas, multiple colorectal, 608456 (3), Autosomal recessive; Gastric cancer, somatic, 613659 (3)
MVD	603236	Porokeratosis 7, multiple types, 614714 (3), Autosomal dominant

MVK	251170	Hyper-IgD syndrome, 260920 (3), Autosomal recessive; Porokeratosis 3, multiple types, 175900 (3), Autosomal dominant; Mevalonic aciduria, 610377 (3), Autosomal recessive
MVP	605088/157700	-/Mitral valve prolapse, myxomatous 1, 157700 (2), Autosomal dominant
MXI1	600020	Prostate cancer, somatic, 176807 (3); Neurofibrosarcoma, somatic (3)
MYB	189990	{T-cell acute lymphoblastic leukemia} (3)
MYBPC1	160794	Myopathy, congenital, with tremor, 618524 (3), Autosomal dominant; Lethal congenital contracture syndrome 4, 614915 (3), Autosomal recessive; Arthrogryposis, distal, type 1B, 614335 (3), Autosomal dominant
MYBPC3	600958	Cardiomyopathy, hypertrophic, 4, 115197 (3), Autosomal dominant, Autosomal recessive; Cardiomyopathy, dilated, 1MM, 615396 (3), Autosomal dominant; Left ventricular noncompaction 10, 615396 (3), Autosomal dominant
MYC	190080	Burkitt lymphoma, somatic, 113970 (3)
MYCN	164840	Feingold syndrome 1, 164280 (3), Autosomal dominant
MYD88	602170	Macroglobulinemia, Waldenstrom, somatic, 153600 (3); Immunodeficiency 68, 612260 (3), Autosomal recessive
MYF5	159990	Ophthalmoplegia, external, with rib and vertebral anomalies, 618155 (3), Autosomal recessive
MYH10	160776	MYH10-related Multiple congenital anomalies; Bilateral ventriculomegaly;aqueductal stenosis PMID:Â 25003005, 25356899, 24825879
MYH11	160745	Megacystis-microcolon-intestinal hypoperistalsis syndrome 2, 619351 (3), Autosomal recessive; Aortic aneurysm, familial thoracic 4, 132900 (3), Autosomal dominant; Visceral myopathy 2, 619350 (3), Autosomal dominant
MYH14	608568	?Peripheral neuropathy, myopathy, hoarseness, and hearing loss, 614369 (3), Autosomal dominant; Deafness, autosomal dominant 4A, 600652 (3), Autosomal dominant
MYH2	160740	Proximal myopathy and ophthalmoplegia, 605637 (3), Autosomal dominant, Autosomal recessive
MYH3	160720	Contractures, pterygia, and spondylocarpostarsal fusion syndrome 1A, 178110 (3), Autosomal dominant; Contractures, pterygia, and spondylocarpotarsal fusion syndrome 1B, 618469 (3), Autosomal recessive; Arthrogryposis, distal, type 2B3 (Sheldon-Hall), 618436 (3), Autosomal dominant; Arthrogryposis, distal, type 2A (Freeman-Sheldon), 193700 (3), Autosomal dominant
MYH6	160710	{Sick sinus syndrome 3}, 614090 (3); Atrial septal defect 3, 614089 (3); Cardiomyopathy, dilated, 1EE, 613252 (3), Autosomal dominant; Cardiomyopathy, hypertrophic, 14, 613251 (3), Autosomal dominant

MYH7	160760	Laing distal myopathy, 160500 (3), Autosomal dominant; Cardiomyopathy, hypertrophic, 1, 192600 (3), Digenic dominant, Autosomal dominant; Left ventricular noncompaction 5, 613426 (3), Autosomal dominant; Cardiomyopathy, dilated, 1S, 613426 (3), Autosomal dominant; Scapuloperoneal syndrome, myopathic type, 181430 (3), Autosomal dominant; Myopathy, myosin storage, autosomal dominant, 608358 (3), Autosomal dominant; Myopathy, myosin storage, autosomal recessive, 255160 (3), Autosomal recessive
MYH8	160741	Carney complex variant, 608837 (3); Trismus-pseudocamptodactyly syndrome, 158300 (3), Autosomal dominant
MYH9	160775	Macrothrombocytopenia and granulocyte inclusions with or without nephritis or sensorineural hearing loss, 155100 (3), Autosomal dominant; Deafness, autosomal dominant 17, 603622 (3), Autosomal dominant
MYL1	160780	Myopathy, congenital, with fast-twitch (type II) fiber atrophy, 618414 (3), Autosomal recessive
MYL11 (MYLPF)	617378	Arthrogryposis, distal, type 1C, 619110 (3), Autosomal dominant, Autosomal recessive
MYL2	160781	Cardiomyopathy, hypertrophic, 10, 608758 (3), Autosomal dominant; Myopathy, myofibrillar, 12, infantile-onset, with cardiomyopathy, 619424 (3), Autosomal recessive
MYL3	160790	Cardiomyopathy, hypertrophic, 8, 608751 (3), Autosomal dominant, Autosomal recessive
MYL4	160770	?Atrial fibrillation, familial, 18, 617280 (3), Autosomal dominant
MYL9	609905	?Megacystis-microcolon-intestinal hypoperistalsis syndrome 4, 619365 (3), Autosomal recessive
MYLK	600922	Megacystis-microcolon-intestinal hypoperistalsis syndrome 1, 249210 (3), Autosomal recessive; Aortic aneurysm, familial thoracic 7, 613780 (3), Autosomal dominant
MYLK2	606566	Cardiomyopathy, hypertrophic, 1, digenic, 192600 (3), Digenic dominant, Autosomal dominant
MYMK	615345	Carey-Fineman-Ziter syndrome, 254940 (3), Autosomal recessive
MYMX	619912	?Carey-Fineman-Ziter syndrome 2, 619941 (3), Autosomal recessive
MYO15A	602666	Deafness, autosomal recessive 3, 600316 (3), Autosomal recessive
MYO18B	607295	Klippel-Feil syndrome 4, autosomal recessive, with myopathy and facial dysmorphism, 616549 (3), Autosomal recessive
MYO1E	601479	Glomerulosclerosis, focal segmental, 6, 614131 (3), Autosomal recessive
MYO1H	614636	?Central hypoventilation syndrome, congenital, 2, and autonomic dysfunction, 619482 (3), Autosomal recessive
MYO3A	606808	Deafness, autosomal recessive 30, 607101 (3), Autosomal recessive
MYO5A	160777	Griscelli syndrome, type 1, 214450 (3), Autosomal recessive
MYO5B	606540	Diarrhea 2, with microvillus atrophy, with or without cholestasis, 251850 (3), Autosomal recessive; Cholestasis, progressive familial intrahepatic, 10, 619868 (3), Autosomal recessive

MYO6	600970	Deafness, autosomal dominant 22, with hypertrophic cardiomyopathy, 606346 (3), Autosomal dominant; Deafness, autosomal dominant 22, 606346 (3), Autosomal dominant; Deafness, autosomal recessive 37, 607821 (3), Autosomal recessive
MYO7A	276903	Deafness, autosomal recessive 2, 600060 (3), Autosomal recessive; Usher syndrome, type 1B, 276900 (3), Autosomal recessive; Deafness, autosomal dominant 11, 601317 (3), Autosomal dominant
MYO9A	604875	Myasthenic syndrome, congenital, 24, presynaptic, 618198 (3), Autosomal recessive
MYO9B	602129	{Celiac disease, susceptibility to, 4}, 609753 (3)
MYOC	601652	Glaucoma 1A, primary open angle, 137750 (3), Autosomal dominant
MYOCD	606127	Megabladder, congenital, 618719 (3), Autosomal dominant
MYOD1	159970	Myopathy, congenital, with diaphragmatic defects, respiratory insufficiency, and dysmorphic facies, 618975 (3), Autosomal recessive
MYOF	604603	?Angioedema, hereditary, 7, 619366 (3), Autosomal dominant
MYORG	618255	Basal ganglia calcification, idiopathic, 7, autosomal recessive, 618317 (3), Autosomal recessive
MYOT	604103	Myopathy, myofibrillar, 3, 609200 (3), Autosomal dominant; Myopathy, spheroid body, 182920 (3), Autosomal dominant
MYOZ2	605602	Cardiomyopathy, hypertrophic, 16, 613838 (3), Autosomal dominant
MYPN	608517	Cardiomyopathy, hypertrophic, 22, 615248 (3), Autosomal dominant; Cardiomyopathy, familial restrictive, 4, 615248 (3), Autosomal dominant; Cardiomyopathy, dilated, 1KK, 615248 (3), Autosomal dominant; Nemaline myopathy 11, autosomal recessive, 617336 (3), Autosomal recessive
MYRF	608329	Encephalitis/encephalopathy, mild, with reversible myelin vacuolization, 618113 (3), Autosomal dominant; Cardiac-urogenital syndrome, 618280 (3), Autosomal dominant
MYSM1	612176	Bone marrow failure syndrome 4, 618116 (3), Autosomal recessive
MYT1	600379	Oculo-auriculo-vertebral spectrum (OAVS);OAVS/Goldenhar syndrome PMID: 28612832, 27358179, 32871052
MYT1L	613084	Intellectual developmental disorder, autosomal dominant 39, 616521 (3), Autosomal dominant
NAA10	300013	Microphthalmia, syndromic 1, 309800 (3), X-linked; Ogden syndrome, 300855 (3), X-linked recessive, X-linked dominant
NAA15	608000	Intellectual developmental disorder, autosomal dominant 50, with behavioral abnormalities, 617787 (3), Autosomal dominant
NAA20	610833	Intellectual developmental disorder, autosomal recessive 73, 619717 (3), Autosomal recessive
NACC1	610672	Neurodevelopmental disorder with epilepsy, cataracts, feeding difficulties, and delayed brain myelination, 617393 (3), Autosomal dominant
NADK2	615787	2,4-dienoyl-CoA reductase deficiency, 616034 (3), Autosomal recessive
NADSYN1	608285	Vertebral, cardiac, renal, and limb defects syndrome 3, 618845 (3), Autosomal recessive

NAGA	104170	Schindler disease, type I, 609241 (3), Autosomal recessive; Kanzaki disease, 609242 (3), Autosomal recessive; Schindler disease, type III, 609241 (3), Autosomal recessive
NAGLU	609701	?Charcot-Marie-Tooth disease, axonal, type 2V, 616491 (3), Autosomal dominant; Mucopolysaccharidosis type IIIB (Sanfilippo B), 252920 (3), Autosomal recessive
NAGS	608300	N-acetylglutamate synthase deficiency, 237310 (3), Autosomal recessive
NALCN	611549	Congenital contractures of the limbs and face, hypotonia, and developmental delay, 616266 (3), Autosomal dominant; Hypotonia, infantile, with psychomotor retardation and characteristic facies 1, 615419 (3), Autosomal recessive
NANOS1	608226	Spermatogenic failure 12, 615413 (3), Autosomal dominant
NANS	605202	Spondyloepimetaphyseal dysplasia, Camera-Genevieve type, 610442 (3), Autosomal recessive
NARS1	108410	Neurodevelopmental disorder with microcephaly, impaired language, epilepsy, and gait abnormalities, autosomal dominant, 619092 (3), Autosomal dominant; Neurodevelopmental disorder with microcephaly, impaired language, and gait abnormalities, autosomal recessive, 619091 (3), Autosomal recessive
NARS2	612803	Combined oxidative phosphorylation deficiency 24, 616239 (3), Autosomal recessive; ?Deafness, autosomal recessive 94, 618434 (3), Autosomal recessive
NAT2	612182	[Acetylation, slow], 243400 (3), Autosomal recessive
NAT8L	610647	?N-acetylaspartate deficiency, 614063 (3), Autosomal recessive
NAXD	615910	Encephalopathy, progressive, early-onset, with brain edema and/or leukoencephalopathy, 2, 618321 (3), Autosomal recessive
NAXE	608862	Encephalopathy, progressive, early-onset, with brain edema and/or leukoencephalopathy, 617186 (3), Autosomal recessive
NBAS	608025	Short stature, optic nerve atrophy, and Pelger-Huet anomaly, 614800 (3), Autosomal recessive; Infantile liver failure syndrome 2, 616483 (3), Autosomal recessive
NBEA	604889	Neurodevelopmental disorder with or without early-onset generalized epilepsy, 619157 (3), Autosomal dominant
NBEAL2	614169	Gray platelet syndrome, 139090 (3), Autosomal recessive
NBN	602667	Leukemia, acute lymphoblastic, 613065 (3); Aplastic anemia, 609135 (3); Nijmegen breakage syndrome, 251260 (3), Autosomal recessive
NCAPD2	615638	?Microcephaly 21, primary, autosomal recessive, 617983 (3), Autosomal recessive
NCAPD3	609276	Microcephaly 22, primary, autosomal recessive, 617984 (3), Autosomal recessive
NCAPG2	608532	Khan-Khan-Katsanis syndrome, 618460 (3), Autosomal recessive
NCAPH	602332	?Microcephaly 23, primary, autosomal recessive, 617985 (3), Autosomal recessive

NCDN	608458	Neurodevelopmental disorder with infantile epileptic spasms, 619373 (3), Autosomal dominant
NCF1	608512	Chronic granulomatous disease 1, autosomal recessive, 233700 (3), Autosomal recessive
NCF2	608515	Chronic granulomatous disease 2, autosomal recessive, 233710 (3), Autosomal recessive
NCF4	601488	Chronic granulomatous disease 3, autosomal recessive, 613960 (3), Autosomal recessive
NCKAP1L	141180	Immunodeficiency 72 with autoinflammation, 618982 (3), Autosomal recessive
NCR3	611550	{Malaria, mild, susceptibility to}, 609148 (3)
NCSTN	605254	Acne inversa, familial, 1, 142690 (3), Autosomal dominant
NDE1	609449	Microhydranencephaly, 605013 (3), Autosomal recessive; Lissencephaly 4 (with microcephaly), 614019 (3), Autosomal recessive
NDNF	616506	Hypogonadotropic hypogonadism 25 with anosmia, 618841 (3), Autosomal dominant
NDP	300658	Exudative vitreoretinopathy 2, X-linked, 305390 (3), X-linked recessive, X-linked dominant; Norrie disease, 310600 (3), X-linked recessive
NDRG1	605262	Charcot-Marie-Tooth disease, type 4D, 601455 (3), Autosomal recessive
NDST1	600853	Intellectual developmental disorder, autosomal recessive 46, 616116 (3), Autosomal recessive
NDUFA1	300078	Mitochondrial complex I deficiency, nuclear type 12, 301020 (3), X-linked recessive
NDUFA10	603835	Mitochondrial complex I deficiency, nuclear type 22, 618243 (3), Autosomal recessive
NDUFA11	612638	Mitochondrial complex I deficiency, nuclear type 14, 618236 (3), Autosomal recessive
NDUFA12	614530	Mitochondrial complex I deficiency, nuclear type 23, 618244 (3), Autosomal recessive
NDUFA13	609435	{Thyroid carcinoma, Hurthle cell}, 607464 (3); ?Mitochondrial complex I deficiency, nuclear type 28, 618249 (3), Autosomal recessive
NDUFA2	602137	Mitochondrial complex I deficiency, nuclear type 13, 618235 (3), Autosomal recessive
NDUFA4	603833	?Mitochondrial complex IV deficiency, nuclear type 21, 619065 (3), Autosomal recessive
NDUFA6	602138	Mitochondrial complex I deficiency, nuclear type 33, 618253 (3), Autosomal recessive
NDUFA8	603359	Mitochondrial complex I deficiency, nuclear type 37, 619272 (3), Autosomal recessive
NDUFA9	603834	Mitochondrial complex I deficiency, nuclear type 26, 618247 (3), Autosomal recessive
NDUFAF1	606934	Mitochondrial complex I deficiency, nuclear type 11, 618234 (3), Autosomal recessive

NDUFAF2	609653	Mitochondrial complex I deficiency, nuclear type 10, 618233 (3), Autosomal recessive
NDUFAF3	612911	Mitochondrial complex I deficiency, nuclear type 18, 618240 (3), Autosomal recessive
NDUFAF4	611776	Mitochondrial complex I deficiency, nuclear type 15, 618237 (3), Autosomal recessive
NDUFAF5	612360	Mitochondrial complex I deficiency, nuclear type 16, 618238 (3), Autosomal recessive
NDUFAF6	612392	Mitochondrial complex I deficiency, nuclear type 17, 618239 (3), Autosomal recessive; Fanconi renotubular syndrome 5, 618913 (3), Autosomal recessive
NDUFAF8	618461	Mitochondrial complex I deficiency, nuclear type 34, 618776 (3), Autosomal recessive
NDUFB10	603843	?Mitochondrial complex I deficiency, nuclear type 35, 619003 (3), Autosomal recessive
NDUFB11	300403	Linear skin defects with multiple congenital anomalies 3, 300952 (3), X-linked dominant; ?Mitochondrial complex I deficiency, nuclear type 30, 301021 (3), X-linked
NDUFB3	603839	Mitochondrial complex I deficiency, nuclear type 25, 618246 (3), Autosomal recessive
NDUFB8	602140	Mitochondrial complex I deficiency, nuclear type 32, 618252 (3), Autosomal recessive
NDUFB9	601445	?Mitochondrial complex I deficiency, nuclear type 24, 618245 (3), Autosomal recessive
NDUFC2	603845	Mitochondrial complex I deficiency, nuclear type 36, 619170 (3), Autosomal recessive
NDUFS1	157655	Mitochondrial complex I deficiency, nuclear type 5, 618226 (3), Autosomal recessive
NDUFS2	602985	Mitochondrial complex I deficiency, nuclear type 6, 618228 (3), Autosomal recessive
NDUFS3	603846	Mitochondrial complex I deficiency, nuclear type 8, 618230 (3), Autosomal recessive
NDUFS4	602694	Mitochondrial complex I deficiency, nuclear type 1, 252010 (3), Autosomal recessive
NDUFS6	603848	Mitochondrial complex I deficiency, nuclear type 9, 618232 (3), Autosomal recessive
NDUFS7	601825	Mitochondrial complex I deficiency, nuclear type 3, 618224 (3), Autosomal recessive
NDUFS8	602141	Mitochondrial complex I deficiency, nuclear type 2, 618222 (3), Autosomal recessive
NDUFV1	161015	Mitochondrial complex I deficiency, nuclear type 4, 618225 (3), Autosomal recessive
NDUFV2	600532	Mitochondrial complex I deficiency, nuclear type 7, 618229 (3), Autosomal recessive

NEB	161650	Nemaline myopathy 2, autosomal recessive, 256030 (3), Autosomal recessive; Arthrogryposis multiplex congenita 6, 619334 (3), Autosomal recessive
NECAP1	611623	Developmental and epileptic encephalopathy 21, 615833 (3), Autosomal recessive
NECTIN1	600644	Cleft lip/palate-ectodermal dysplasia syndrome, 225060 (3), Autosomal recessive; Orofacial cleft 7, 225060 (3), Autosomal recessive
NECTIN4	609607	Ectodermal dysplasia-syndactyly syndrome 1, 613573 (3), Autosomal recessive
NEDD4L	606384	Periventricular nodular heterotopia 7, 617201 (3), Autosomal dominant
NEFH	162230	Charcot-Marie-Tooth disease, axonal, type 2CC, 616924 (3), Autosomal dominant; {?Amyotrophic lateral sclerosis, susceptibility to}, 105400 (3), Autosomal dominant, Autosomal recessive
NEFL	162280	Charcot-Marie-Tooth disease, type 1F, 607734 (3), Autosomal dominant, Autosomal recessive; Charcot-Marie-Tooth disease, dominant intermediate G, 617882 (3), Autosomal dominant; Charcot-Marie-Tooth disease, type 2E, 607684 (3), Autosomal dominant
NEK1	604588	Short-rib thoracic dysplasia 6 with or without polydactyly, 263520 (3), Digenic recessive, Autosomal recessive; {Amyotrophic lateral sclerosis, susceptibility to, 24}, 617892 (3), Autosomal dominant
NEK10	618726	Ciliary dyskinesia, primary, 44, 618781 (3), Autosomal recessive
NEK2	604043	?Retinitis pigmentosa 67, 615565 (3), Autosomal recessive
NEK8	609799	Renal-hepatic-pancreatic dysplasia 2, 615415 (3), Autosomal recessive; ?Nephronophthisis 9, 613824 (3)
NEK9	609798	?Arthrogryposis, Perthes disease, and upward gaze palsy, 614262 (3), Autosomal recessive; Nevus comedonicus, somatic, 617025 (3); Lethal congenital contracture syndrome 10, 617022 (3), Autosomal recessive
NEMF	608378	Intellectual developmental disorder with speech delay and axonal peripheral neuropathy, 619099 (3), Autosomal recessive
NEPRO	617089	Anauxetic dysplasia 3, 618853 (3), Autosomal recessive
NEU1	608272	Sialidosis, type II, 256550 (3), Autosomal recessive; Sialidosis, type I, 256550 (3), Autosomal recessive
NEUROD1	601724	{Type 2 diabetes mellitus, susceptibility to}, 125853 (3), Autosomal dominant; Maturity-onset diabetes of the young 6, 606394 (3)
NEUROD2	601725	Developmental and epileptic encephalopathy 72, 618374 (3), Autosomal dominant
NEUROG3	604882	Diarrhea 4, malabsorptive, congenital, 610370 (3), Autosomal recessive
NEXMIF	300524	Intellectual developmental disorder, X-linked 98, 300912 (3), X-linked dominant
NEXN	613121	Cardiomyopathy, dilated, 1CC, 613122 (3), Autosomal dominant; Cardiomyopathy, hypertrophic, 20, 613876 (3), Autosomal dominant

NF1	613113	Watson syndrome, 193520 (3), Autosomal dominant; Leukemia, juvenile myelomonocytic, 607785 (3), Autosomal dominant, Somatic mutation; Neurofibromatosis, familial spinal, 162210 (3), Autosomal dominant; Neurofibromatosis, type 1, 162200 (3), Autosomal dominant; Neurofibromatosis-Noonan syndrome, 601321 (3), Autosomal dominant
NF2	607379	Neurofibromatosis, type 2, 101000 (3), Autosomal dominant; Meningioma, NF2-related, somatic, 607174 (3); Schwannomatosis, somatic, 162091 (3)
NFASC	609145	Neurodevelopmental disorder with central and peripheral motor dysfunction, 618356 (3), Autosomal recessive
NFE2L2	600492	Immunodeficiency, developmental delay, and hypohomocysteinemia, 617744 (3), Autosomal dominant
NFIA	600727	Brain malformations with or without urinary tract defects, 613735 (3), Autosomal dominant
NFIB	600728	Macrocephaly, acquired, with impaired intellectual development, 618286 (3), Autosomal dominant
NFIX	164005	Marshall-Smith syndrome, 602535 (3), Autosomal dominant; Malan syndrome, 614753 (3), Autosomal dominant
NFKB1	164011	Immunodeficiency, common variable, 12, 616576 (3), Autosomal dominant
NFKB2	164012	Immunodeficiency, common variable, 10, 615577 (3), Autosomal dominant
NFKBIA	164008	Ectodermal dysplasia and immunodeficiency 2, 612132 (3), Autosomal dominant
NFKBIL1	601022	{Rheumatoid arthritis, susceptibility to}, 180300 (3)
NFS1	603485	Combined oxidative phosphorylation deficiency 52, 619386 (3), Autosomal recessive
NFU1	608100	Multiple mitochondrial dysfunctions syndrome 1, 605711 (3), Autosomal recessive
NGF	162030	Neuropathy, hereditary sensory and autonomic, type V, 608654 (3), Autosomal recessive
NGLY1	610661	Congenital disorder of deglycosylation 1, 615273 (3), Autosomal recessive
NHEJ1	611290	Severe combined immunodeficiency with microcephaly, growth retardation, and sensitivity to ionizing radiation, 611291 (3)
NHLH2	162361	?Hypogonadotropic hypogonadism 27 without anosmia, 619755 (3), Autosomal recessive
NHLRC1	608072	Epilepsy, progressive myoclonic 2B (Lafora), 254780 (3), Autosomal recessive
NHLRC2	618277	FINCA syndrome, 618278 (3), Autosomal recessive
NHP2	606470	Dyskeratosis congenita, autosomal recessive 2, 613987 (3), Autosomal recessive
NHS	300457	Cataract 40, X-linked, 302200 (3), X-linked; Nance-Horan syndrome, 302350 (3), X-linked dominant
NIN	608684	?Seckel syndrome 7, 614851 (3), Autosomal recessive
NIPA1	608145	Spastic paraplegia 6, autosomal dominant, 600363 (3), Autosomal dominant

NIPAL4	609383	Ichthyosis, congenital, autosomal recessive 6, 612281 (3), Autosomal recessive
NIPBL	608667	Cornelia de Lange syndrome 1, 122470 (3), Autosomal dominant
NKAP	300766	Intellectual developmental disorder, X-linked syndromic, Hackman-Di Donato type, 301039 (3), X-linked recessive
NKX2-1	600635	Chorea, hereditary benign, 118700 (3), Autosomal dominant; {Thyroid cancer, nonmedullary, 1}, 188550 (3), Autosomal dominant; Choreoathetosis, hypothyroidism, and neonatal respiratory distress, 610978 (3), Autosomal dominant
NKX2-5	600584	Hypoplastic left heart syndrome 2, 614435 (3), Autosomal dominant; Tetralogy of Fallot, 187500 (3), Autosomal dominant; Hypothyroidism, congenital nongoitrous, 5, 225250 (3), Autosomal dominant; Conotruncal heart malformations, variable, 217095 (3); Ventricular septal defect 3, 614432 (3), Autosomal dominant; Atrial septal defect 7, with or without AV conduction defects, 108900 (3), Autosomal dominant
NKX2-6	611770	Persistent truncus arteriosus, 217095 (3); Conotruncal heart malformations, 217095 (3)
NKX3-2	602183	Spondylo-megaepiphyseal-metaphyseal dysplasia, 613330 (3), Autosomal recessive
NKX6-2	605955	Spastic ataxia 8, autosomal recessive, with hypomyelinating leukodystrophy, 617560 (3), Autosomal recessive
NLGN1	600568	{Autism, susceptibility to, 20}, 618830 (3), Autosomal dominant
NLGN3	300336	{Asperger syndrome susceptibility, X-linked 1}, 300494 (3), Multifactorial, Isolated cases, X-linked; {Autism susceptibility, X-linked 1}, 300425 (3), X-linked
NLGN4X	300427	{Asperger syndrome susceptibility, X-linked 2}, 300497 (3), X-linked; Intellectual developmental disorder, X-linked, 300495 (3), Multifactorial, Isolated cases, X-linked; {Autism susceptibility, X-linked 2}, 300495 (3), Multifactorial, Isolated cases, X-linked
NLRC4	606831	?Familial cold autoinflammatory syndrome 4, 616115 (3), Autosomal dominant; Autoinflammation with infantile enterocolitis, 616050 (3), Autosomal dominant
NLRP1	606636	{Vitiligo-associated multiple autoimmune disease susceptibility 1}, 606579 (3); ?Respiratory papillomatosis, juvenile recurrent, congenital, 618803 (3), Autosomal recessive; Autoinflammation with arthritis and dyskeratosis, 617388 (3), Autosomal dominant, Autosomal recessive; Palmoplantar carcinoma, multiple self-healing, 615225 (3), Autosomal dominant
NLRP12	609648	Familial cold autoinflammatory syndrome 2, 611762 (3), Autosomal dominant

NLRP3	606416	CINCA syndrome, 607115 (3), Autosomal dominant; Familial cold inflammatory syndrome 1, 120100 (3), Autosomal dominant; Keratoendothelitis fugax hereditaria, 148200 (3), Autosomal dominant; Deafness, autosomal dominant 34, with or without inflammation, 617772 (3), Autosomal dominant; Muckle-Wells syndrome, 191900 (3), Autosomal dominant
NLRP7	609661	Hydatidiform mole, recurrent, 1, 231090 (3), Autosomal recessive
NME8	607421	Ciliary dyskinesia, primary, 6, 610852 (3), Autosomal recessive
NMNAT1	608700	Spondyloepiphyseal dysplasia, sensorineural hearing loss, intellectual developmental disorder, and Leber congenital amaurosis, 619260 (3), Autosomal recessive; Leber congenital amaurosis 9, 608553 (3), Autosomal recessive
NMNAT2	608701	NeuropathyÂ PMID: 31132363, 33248200
NNMT	600008	Homocysteine plasma level, 600008 (2)
NNT	607878	Glucocorticoid deficiency 4, with or without mineralocorticoid deficiency, 614736 (3), Autosomal recessive
NOBOX	610934	Premature ovarian failure 5, 611548 (3), Autosomal dominant
NOD2	605956	Blau syndrome, 186580 (3), Autosomal dominant; {Yao syndrome}, 617321 (3), Multifactorial; {Inflammatory bowel disease 1, Crohn disease}, 266600 (3), Multifactorial
NODAL	601265	Heterotaxy, visceral, 5, 270100 (3), Autosomal dominant
NOG	602991	Symphalangism, proximal, 1A, 185800 (3), Autosomal dominant; Brachydactyly, type B2, 611377 (3), Autosomal dominant; Stapes ankylosis with broad thumbs and toes, 184460 (3), Autosomal dominant; Tarsal-carpal coalition syndrome, 186570 (3), Autosomal dominant; Multiple synostoses syndrome 1, 186500 (3), Autosomal dominant
NOL3	605235	?Myoclonus, familial, 1, 614937 (3), Autosomal dominant
NONO	300084	Intellectual developmental disorder, X-linked syndromic 34, 300967 (3), X-linked
NOP10	606471	Dyskeratosis congenita, autosomal recessive 1, 224230 (3), Autosomal recessive
NOP56	614154	Spinocerebellar ataxia 36, 614153 (3), Autosomal dominant
NOS1AP	605551	Nephrotic syndrome, type 22, 619155 (3), Autosomal recessive
NOS2	163730	{Malaria, resistance to}, 611162 (3)
NOS3	163729	{Coronary artery spasm 1, susceptibility to} (3); {Hypertension, susceptibility to}, 145500 (3), Multifactorial; {Placental abruption} (3); {Alzheimer disease, late-onset, susceptibility to}, 104300 (3), Autosomal dominant; {Hypertension, pregnancy-induced}, 189800 (3), Autosomal dominant; {Ischemic stroke, susceptibility to}, 601367 (3), Multifactorial
NOTCH1	190198	Adams-Oliver syndrome 5, 616028 (3), Autosomal dominant; Aortic valve disease 1, 109730 (3), Autosomal dominant
NOTCH2	600275	Alagille syndrome 2, 610205 (3), Autosomal dominant; Hajdu-Cheney syndrome, 102500 (3), Autosomal dominant

NOTCH2NLC	618025	Tremor, hereditary essential, 6, 618866 (3), Autosomal dominant; Oculopharyngodistal myopathy 3, 619473 (3), Autosomal dominant; Neuronal intranuclear inclusion disease, 603472 (3), Autosomal dominant
NOTCH3	600276	Lateral meningocele syndrome, 130720 (3), Autosomal dominant; ?Myofibromatosis, infantile 2, 615293 (3), Autosomal dominant; Cerebral arteriopathy with subcortical infarcts and leukoencephalopathy 1, 125310 (3), Autosomal dominant
NOVA2	601991	Neurodevelopmental disorder with or without autistic features and/or structural brain abnormalities, 618859 (3), Autosomal dominant
NPC1	607623	Niemann-Pick disease, type C1, 257220 (3), Autosomal recessive; Niemann-Pick disease, type D, 257220 (3), Autosomal recessive
NPC1L1	608010	[Ezetimibe, nonresponse to], 617966 (3); [Low density lipoprotein cholesterol level QTL 7], 617966 (3)
NPC2	601015	Niemann-pick disease, type C2, 607625 (3), Autosomal recessive
NPHP1	607100	Joubert syndrome 4, 609583 (3), Autosomal recessive; Nephronophthisis 1, juvenile, 256100 (3), Autosomal recessive; Senior-Loken syndrome-1, 266900 (3), Autosomal recessive
NPHP3	608002	Nephronophthisis 3, 604387 (3), Autosomal recessive; Renal-hepatic-pancreatic dysplasia 1, 208540 (3), Autosomal recessive; Meckel syndrome 7, 267010 (3), Autosomal recessive
NPHP4	607215	Senior-Loken syndrome 4, 606996 (3), Autosomal recessive; Nephronophthisis 4, 606966 (3), Autosomal recessive
NPHS1	602716	Nephrotic syndrome, type 1, 256300 (3), Autosomal recessive
NPHS2	604766	Nephrotic syndrome, type 2, 600995 (3), Autosomal recessive
NPM1	164040	Leukemia, acute myeloid, somatic, 601626 (3)
NPPA	108780	Atrial standstill 2, 615745 (3), Autosomal recessive; Atrial fibrillation, familial, 6, 612201 (3), Autosomal dominant
NPR2	108961	Epiphyseal chondrodysplasia, Miura type, 615923 (3), Autosomal dominant; Short stature with nonspecific skeletal abnormalities, 616255 (3), Autosomal dominant; Acromesomelic dysplasia 1, Maroteaux type, 602875 (3), Autosomal recessive
NPR3	108962	Boudin-Mortier syndrome, 619543 (3), Autosomal recessive
NPRL2	607072	Epilepsy, familial focal, with variable foci 2, 617116 (3), Autosomal dominant
NPRL3	600928	Epilepsy, familial focal, with variable foci 3, 617118 (3), Autosomal dominant
NPSR1	608595	{Asthma, susceptibility to, 2}, 608584 (3)
NQO1	125860	{Breast cancer, poor survival after chemotherapy for} (3); {Leukemia, post-chemotherapy, susceptibility to} (3); {Benzene toxicity, susceptibility to} (3)
NQO2	160998	{?Breast cancer susceptibility}, 114480 (1), Autosomal dominant, Somatic mutation
NR0B1	300473	Adrenal hypoplasia, congenital, 300200 (3), X-linked recessive; 46XY sex reversal 2, dosage-sensitive, 300018 (3), X-linked

NR0B2	604630	Obesity, mild, early-onset, 601665 (3), Multifactorial, Autosomal dominant, Autosomal recessive
NR1H4	603826	Cholestasis, progressive familial intrahepatic, 5, 617049 (3), Autosomal recessive
NR2E3	604485	Retinitis pigmentosa 37, 611131 (3), Autosomal dominant, Autosomal recessive; Enhanced S-cone syndrome, 268100 (3), Autosomal recessive
NR2F1	132890	Bosch-Boonstra-Schaaf optic atrophy syndrome, 615722 (3), Autosomal dominant
NR2F2	107773	46,XX sex reversal 5, 618901 (3), Autosomal dominant; Congenital heart defects, multiple types, 4, 615779 (3), Autosomal dominant
NR3C1	138040	Glucocorticoid resistance, 615962 (3), Autosomal dominant
NR3C2	600983	Pseudohypoaldosteronism type I, autosomal dominant, 177735 (3), Autosomal dominant; Hypertension, early-onset, autosomal dominant, with exacerbation in pregnancy, 605115 (3)
NR4A2	601828	Intellectual developmental disorder with language impairment and early-onset DOPA-responsive dystonia-parkinsonism, 619911 (3), Autosomal dominant
NR4A3	600542	Chondrosarcoma, extraskeletal myxoid, 612237 (3)
NR5A1	184757	46, XX sex reversal 4, 617480 (3), Autosomal dominant; Premature ovarian failure 7, 612964 (3), Autosomal dominant; 46XY sex reversal 3, 612965 (3), Autosomal dominant; Adrenocortical insufficiency, 612964 (3), Autosomal dominant; Spermatogenic failure 8, 613957 (3), Autosomal dominant
NRAS	164790	Noonan syndrome 6, 613224 (3), Autosomal dominant; ?RAS-associated autoimmune lymphoproliferative syndrome type IV, somatic, 614470 (3); Melanocytic nevus syndrome, congenital, somatic, 137550 (3); Epidermal nevus, somatic, 162900 (3); Schimmelpenning-Feuerstein-Mims syndrome, somatic mosaic, 163200 (3); Thyroid carcinoma, follicular, somatic, 188470 (3); Neurocutaneous melanosis, somatic, 249400 (3); Colorectal cancer, somatic, 114500 (3)
NRCAM	601581	Neurodevelopmental disorder with neuromuscular and skeletal abnormalities, 619833 (3), Autosomal recessive
NRG1	142445	{?Schizophrenia, susceptibility to}, 603013 (1)
NRIP1	602490	?Congenital anomalies of kidney and urinary tract 3, 618270 (3), Autosomal dominant
NRL	162080	Retinitis pigmentosa 27, 613750 (3), Autosomal dominant; Retinal degeneration, autosomal recessive, clumped pigment type (3)
NRROS	615322	Seizures, early-onset, with neurodegeneration and brain calcification, 618875 (3), Autosomal recessive
NRXN1	600565	Pitt-Hopkins-like syndrome 2, 614325 (3), Autosomal recessive; {Schizophrenia, susceptibility to, 17}, 614332 (3)
NSD1	606681	Sotos syndrome, 117550 (3), Autosomal dominant
NSD2	602952	Rauch-Steindl syndrome, 619695 (3), Autosomal dominant
NSDHL	300275	CK syndrome, 300831 (3), X-linked recessive; CHILD syndrome, 308050 (3), X-linked dominant

NSF	601633	Developmental and epileptic encephalopathy 96, 619340 (3), Autosomal dominant
NSMCE2	617246	Seckel syndrome 10, 617253 (3), Autosomal recessive
NSMCE3	608243	Lung disease, immunodeficiency, and chromosome breakage syndrome, 617241 (3), Autosomal recessive
NSMF	608137	Hypogonadotropic hypogonadism 9 with or without anosmia, 614838 (3), Autosomal dominant
NSRP1	616173	Neurodevelopmental disorder with spasticity, seizures, and brain abnormalities, 620001 (3)
NSUN2	610916	Intellectual developmental disorder, autosomal recessive 5, 611091 (3), Autosomal recessive
NSUN3	617491	Combined oxidative phosphorylation deficiency 48, 619012 (3), Autosomal recessive
NT5C2	600417	Spastic paraplegia 45, autosomal recessive, 613162 (3), Autosomal recessive
NT5C3A	606224	Anemia, hemolytic, due to UMPH1 deficiency, 266120 (3), Autosomal recessive
NT5E	129190	Calcification of joints and arteries, 211800 (3), Autosomal recessive
NTF4	162662	Glaucoma 1, open angle, 1O, 613100 (3)
NTHL1	602656	Familial adenomatous polyposis 3, 616415 (3), Autosomal recessive
NTN1	601614	Mirror movements 4, 618264 (3), Autosomal dominant
NTNG2	618689	Neurodevelopmental disorder with behavioral abnormalities, absent speech, and hypotonia, 618718 (3), Autosomal recessive
NTRK1	191315	Insensitivity to pain, congenital, with anhidrosis, 256800 (3), Autosomal recessive
NTRK2	600456	Developmental and epileptic encephalopathy 58, 617830 (3), Autosomal dominant; Obesity, hyperphagia, and developmental delay, 613886 (3), Autosomal dominant
NUAK2	608131	?Anencephaly 2, 619452 (3), Autosomal recessive
NUBPL	613621	Mitochondrial complex I deficiency, nuclear type 21, 618242 (3), Autosomal recessive
NUDT15	615792	{Thiopurines, poor metabolism of, 2}, 616903 (3), Autosomal dominant
NUDT2	602852	Intellectual developmental disorder with or without peripheral neuropathy, 619844 (3), Autosomal recessive
NUMA1	164009	Leukemia, acute promyelocytic, somatic, 612376 (3)
NUP107	607617	?Ovarian dysgenesis 6, 618078 (3), Autosomal recessive; Galloway-Mowat syndrome 7, 618348 (3), Autosomal recessive; Nephrotic syndrome, type 11, 616730 (3), Autosomal recessive
NUP133	607613	?Galloway-Mowat syndrome 8, 618349 (3), Autosomal recessive; Nephrotic syndrome, type 18, 618177 (3), Autosomal recessive
NUP155	606694	?Atrial fibrillation 15, 615770 (3), Autosomal recessive
NUP160	607614	?Nephrotic syndrome, type 19, 618178 (3), Autosomal recessive
NUP188	615587	Sandestig-Stefanova syndrome, 618804 (3), Autosomal recessive
NUP205	614352	?Nephrotic syndrome, type 13, 616893 (3), Autosomal recessive

NUP214	114350	Leukemia, T-cell acute lymphoblastic, somatic, 613065 (3); Leukemia, acute myeloid, somatic, 601626 (3); {Encephalopathy, acute, infection-induced, susceptibility to, 9}, 618426 (3), Autosomal recessive
NUP37	609264	?Microcephaly 24, primary, autosomal recessive, 618179 (3), Autosomal recessive
NUP62	605815	Striatonigral degeneration, infantile, 271930 (3), Autosomal recessive
NUP85	170285	Nephrotic syndrome, type 17, 618176 (3), Autosomal recessive
NUP88	602552	Fetal akinesia deformation sequence 4, 618393 (3), Autosomal recessive
NUP93	614351	Nephrotic syndrome, type 12, 616892 (3), Autosomal recessive
NUS1	610463	Intellectual developmental disorder, autosomal dominant 55, with seizures, 617831 (3), Autosomal dominant; ?Congenital disorder of glycosylation, type 1aa, 617082 (3), Autosomal recessive
NXN	612895	Robinow syndrome, autosomal recessive 2, 618529 (3), Autosomal recessive
NYX	300278	Night blindness, congenital stationary (complete), 1A, X-linked, 310500 (3), X-linked recessive
OAS1	164350	Immunodeficiency 100 with pulmonary alveolar proteinosis and hypogammaglobulinemia, 618042 (3), Autosomal dominant
OAT	613349	Gyrate atrophy of choroid and retina with or without ornithinemia, 258870 (3), Autosomal recessive
OBSL1	610991	3-M syndrome 2, 612921 (3), Autosomal recessive
OCA2	611409	[Skin/hair/eye pigmentation 1, blue/nonblue eyes], 227220 (3), Autosomal recessive; [Skin/hair/eye pigmentation 1, blond/brown hair], 227220 (3), Autosomal recessive; Albinism, brown oculocutaneous, 203200 (3), Autosomal recessive; Albinism, oculocutaneous, type II, 203200 (3), Autosomal recessive
OCLN	602876	Pseudo-TORCH syndrome 1, 251290 (3), Autosomal recessive
OCRL	300535	Dent disease 2, 300555 (3), X-linked recessive; Lowe syndrome, 309000 (3), X-linked recessive
ODAD1	615038	Ciliary dyskinesia, primary, 20, 615067 (3), Autosomal recessive
ODAD2	615408	Ciliary dyskinesia, primary, 23, 615451 (3), Autosomal recessive
ODAD3	615956	Ciliary dyskinesia, primary, 30, 616037 (3), Autosomal recessive
ODAD4	617095	Ciliary dyskinesia, primary, 35, 617092 (3), Autosomal recessive
ODAPH	614829	Amelogenesis imperfecta, type IIA4, 614832 (3), Autosomal recessive
ODC1	165640	Bachmann-Bupp syndrome, 619075 (3), Autosomal dominant
OFD1	300170	Simpson-Golabi-Behmel syndrome, type 2, 300209 (3), X-linked recessive; ?Retinitis pigmentosa 23, 300424 (3), X-linked recessive; Orofaciodigital syndrome I, 311200 (3), X-linked dominant; Joubert syndrome 10, 300804 (3), X-linked recessive
OGDH	613022	?Oxoglutarate dehydrogenase deficiency, 203740 (3), Autosomal recessive
OGDHL	617513	Yoon-Bellen neurodevelopmental syndrome, 619701 (3), Autosomal recessive
OGG1	601982	Renal cell carcinoma, clear cell, somatic, 144700 (3)

OGT	300255	Intellectual developmental disorder, X-linked 106, 300997 (3), X-linked recessive
OLR1	602601	{Myocardial infarction, susceptibility to}, 608446 (3)
OPA1	605290	Optic atrophy plus syndrome, 125250 (3), Autosomal dominant; {Glaucoma, normal tension, susceptibility to}, 606657 (3); Optic atrophy 1, 165500 (3), Autosomal dominant; Behr syndrome, 210000 (3), Autosomal recessive; ?Mitochondrial DNA depletion syndrome 14 (encephalocardiomyopathic type), 616896 (3), Autosomal recessive
OPA3	606580	3-methylglutaconic aciduria, type III, 258501 (3), Autosomal recessive; Optic atrophy 3 with cataract, 165300 (3), Autosomal dominant
OPCML	600632	Ovarian cancer, somatic, 167000 (3)
OPHN1	300127	Intellectual developmental disorder, X-linked syndromic, Billuart type, 300486 (3), X-linked recessive
OPLAH	614243	5-oxoprolinase deficiency, 260005 (3), Autosomal dominant, Autosomal recessive
OPN1LW	300822	Blue cone monochromacy, 303700 (3), X-linked recessive; Colorblindness, protan, 303900 (3), X-linked
OPN1MW	300821	Colorblindness, deutan, 303800 (3), X-linked; Blue cone monochromacy, 303700 (3), X-linked recessive
OPN1SW	613522	Colorblindness, tritan, 190900 (3), Autosomal dominant
OPTN	602432	Glaucoma 1, open angle, E, 137760 (3), Autosomal dominant; Amyotrophic lateral sclerosis 12 with or without frontotemporal dementia, 613435 (3), Autosomal dominant, Autosomal recessive; {Glaucoma, normal tension, susceptibility to}, 606657 (3)
OR2J3	615016	[C3HEX, ability to smell], 615082 (3), Autosomal dominant
ORAI1	610277	Immunodeficiency 9, 612782 (3), Autosomal recessive; Myopathy, tubular aggregate, 2, 615883 (3), Autosomal dominant
ORC1	601902	Meier-Gorlin syndrome 1, 224690 (3), Autosomal recessive
ORC4	603056	Meier-Gorlin syndrome 2, 613800 (3), Autosomal recessive
ORC6	607213	Meier-Gorlin syndrome 3, 613803 (3), Autosomal recessive
OSBPL2	606731	Deafness, autosomal dominant 67, 616340 (3), Autosomal dominant
OSGEP	610107	Galloway-Mowat syndrome 3, 617729 (3), Autosomal recessive
OSMR	601743	Amyloidosis, primary localized cutaneous, 1, 105250 (3), Autosomal dominant
OSTM1	607649	Osteopetrosis, autosomal recessive 5, 259720 (3), Autosomal recessive
OTC	300461	Ornithine transcarbamylase deficiency, 311250 (3), X-linked
OTOA	607038	Deafness, autosomal recessive 22, 607039 (3), Autosomal recessive
OTOF	603681	Auditory neuropathy, autosomal recessive, 1, 601071 (3), Autosomal recessive; Deafness, autosomal recessive 9, 601071 (3), Autosomal recessive
OTOG	604487	Deafness, autosomal recessive 18B, 614945 (3), Autosomal recessive
OTOGL	614925	Deafness, autosomal recessive 84B, 614944 (3), Autosomal recessive
OTUD5	300713	Multiple congenital anomalies-neurodevelopmental syndrome, X-linked, 301056 (3), X-linked recessive

OTUD6B	612021	Intellectual developmental disorder with dysmorphic facies, seizures, and distal limb anomalies, 617452 (3), Autosomal recessive
OTULIN	615712	Autoinflammation, panniculitis, and dermatosis syndrome, 617099 (3), Autosomal recessive; {Immunodeficiency 107, susceptibility to invasive staphylococcus aureus infection}, 619986 (3), Autosomal dominant
OTX2	600037	Retinal dystrophy, early-onset, with or without pituitary dysfunction, 610125 (3), Autosomal dominant; Pituitary hormone deficiency, combined, 6, 613986 (3), Autosomal dominant; Microphthalmia, syndromic 5, 610125 (3), Autosomal dominant
OVOL2	616441	Corneal dystrophy, posterior polymorphous, 1, 122000 (3), Autosomal dominant
OXCT1	601424	Succinyl CoA:3-oxoacid CoA transferase deficiency, 245050 (3), Autosomal recessive
OXR1	605609	Cerebellar hypoplasia/atrophy, epilepsy, and global developmental delay, 213000 (3), Autosomal recessive
P2RX2	600844	Deafness, autosomal dominant 41, 608224 (3), Autosomal dominant
P2RY12	600515	Bleeding disorder, platelet-type, 8, 609821 (3), Autosomal recessive
P3H1	610339	Osteogenesis imperfecta, type VIII, 610915 (3), Autosomal recessive
P3H2	610341	Myopia, high, with cataract and vitreoretinal degeneration, 614292 (3), Autosomal recessive
P4HA2	600608	Myopia 25, autosomal dominant, 617238 (3), Autosomal dominant
P4HB	176790	Cole-Carpenter syndrome 1, 112240 (3), Autosomal dominant
P4HTM	614584	Hypotonia, hypoventilation, impaired intellectual development, dysautonomia, epilepsy, and eye abnormalities, 618493 (3), Autosomal recessive
PABPN1	602279	Oculopharyngeal muscular dystrophy, 164300 (3), Autosomal dominant
PACS1	607492	Schuurs-Hoeijmakers syndrome, 615009 (3), Autosomal dominant
PACS2	610423	Developmental and epileptic encephalopathy 66, 618067 (3), Autosomal dominant
PADI3	606755	Uncombable hair syndrome, 191480 (3), Autosomal recessive
PADI6	610363	Preimplantation embryonic lethality 2, 617234 (3), Autosomal recessive
PAFAH1B1	601545	Subcortical laminar heterotopia, 607432 (3), Autosomal dominant; Lissencephaly 1, 607432 (3), Autosomal dominant
PAH	612349	[Hyperphenylalaninemia, non-PKU mild], 261600 (3), Autosomal recessive; Phenylketonuria, 261600 (3), Autosomal recessive
PAICS	172439	?Phosphoribosylaminoimidazole carboxylase deficiency, 619859 (3), Autosomal recessive
PAK1	602590	Intellectual developmental disorder with macrocephaly, seizures, and speech delay, 618158 (3), Autosomal dominant
PAK2	605022	?Knobloch syndrome 2, 618458 (3), Autosomal dominant
PAK3	300142	Intellectual developmental disorder, X-linked 30, 300558 (3), X-linked recessive

PALB2	610355	{Breast cancer, susceptibility to}, 114480 (3), Autosomal dominant, Somatic mutation; {Pancreatic cancer, susceptibility to, 3}, 613348 (3); Fanconi anemia, complementation group N, 610832 (3)
PALLD	608092	{Pancreatic cancer, susceptibility to, 1}, 606856 (3), Autosomal dominant
PAM16	614336	Spondylometaphyseal dysplasia, Megarbane-Dagher-Melike type, 613320 (3), Autosomal recessive
PANK2	606157	HARP syndrome, 607236 (3), Autosomal recessive; Neurodegeneration with brain iron accumulation 1, 234200 (3), Autosomal recessive
PANK4	606162	?Cataract 49, 619593 (3), Autosomal dominant
PANX1	608420	Oocyte maturation defect 7, 618550 (3), Autosomal dominant
PAPPA2	619485	Short stature, Dauber-Argente type, 619489 (3), Autosomal recessive
PAPSS2	603005	Brachyolmia 4 with mild epiphyseal and metaphyseal changes, 612847 (3), Autosomal recessive
PARK7	602533	Parkinson disease 7, autosomal recessive early-onset, 606324 (3), Autosomal recessive
PARN	604212	Dyskeratosis congenita, autosomal recessive 6, 616353 (3), Autosomal recessive; Pulmonary fibrosis and/or bone marrow failure, telomere-related, 4, 616371 (3), Autosomal dominant
PARS2	612036	Developmental and epileptic encephalopathy 75, 618437 (3), Autosomal recessive
PATL2	614661	Oocyte maturation defect 4, 617743 (3), Autosomal recessive
PAX1	167411	Otofaciocervical syndrome 2, 615560 (3), Autosomal recessive
PAX2	167409	Glomerulosclerosis, focal segmental, 7, 616002 (3), Autosomal dominant; Papillorenal syndrome, 120330 (3), Autosomal dominant
PAX3	606597	Craniofacial-deafness-hand syndrome, 122880 (3), Autosomal dominant; Waardenburg syndrome, type 3, 148820 (3), Autosomal dominant, Autosomal recessive; Waardenburg syndrome, type 1, 193500 (3), Autosomal dominant; Rhabdomyosarcoma 2, alveolar, 268220 (3), Somatic mutation
PAX4	167413	{Diabetes mellitus, ketosis-prone, susceptibility to}, 612227 (3), Autosomal dominant, Autosomal recessive; Maturity-onset diabetes of the young, type IX, 612225 (3); Diabetes mellitus, type 2, 125853 (3), Autosomal dominant
PAX5	167414	{Leukemia, acute lymphoblastic, susceptibility to, 3}, 615545 (3)
PAX6	607108	Optic nerve hypoplasia, 165550 (3), Autosomal dominant; Cataract with late-onset corneal dystrophy, 106210 (3), Autosomal dominant; ?Coloboma, ocular, 120200 (3), Autosomal dominant; ?Coloboma of optic nerve, 120430 (3), Autosomal dominant; Aniridia, 106210 (3), Autosomal dominant; Anterior segment dysgenesis 5, multiple subtypes, 604229 (3), Autosomal dominant; ?Morning glory disc anomaly, 120430 (3), Autosomal dominant; Foveal hypoplasia 1, 136520 (3), Autosomal dominant; Keratitis, 148190 (3), Autosomal dominant
PAX7	167410	Rhabdomyosarcoma 2, alveolar, 268220 (3), Somatic mutation; Myopathy, congenital, progressive, with scoliosis, 618578 (3), Autosomal recessive

PAX8	167415	Hypothyroidism, congenital, due to thyroid dysgenesis or hypoplasia, 218700 (3), Autosomal dominant
PAX9	167416	Tooth agenesis, selective, 3, 604625 (3), Autosomal dominant
PBRM1	606083	?Renal cell carcinoma, clear cell, 144700 (3)
PBX1	176310	Congenital anomalies of kidney and urinary tract syndrome with or without hearing loss, abnormal ears, or developmental delay, 617641 (3), Autosomal dominant
PC	608786	Pyruvate carboxylase deficiency, 266150 (3), Autosomal recessive
PCARE	613425	Retinitis pigmentosa 54, 613428 (3)
PCBD1	126090	Hyperphenylalaninemia, BH4-deficient, D, 264070 (3), Autosomal recessive
PCCA	232000	Propionicacidemia, 606054 (3), Autosomal recessive
PCCB	232050	Propionicacidemia, 606054 (3), Autosomal recessive
PCDH12	605622	Diencephalic-mesencephalic junction dysplasia syndrome 1, 251280 (3), Autosomal recessive
PCDH15	605514	Usher syndrome, type 1D/F digenic, 601067 (3), Digenic recessive, Autosomal recessive; Deafness, autosomal recessive 23, 609533 (3), Autosomal recessive; Usher syndrome, type 1F, 602083 (3), Autosomal recessive
PCDH19	300460	Developmental and epileptic encephalopathy 9, 300088 (3), X-linked
PCDHGC4	606305	Neurodevelopmental disorder with poor growth and skeletal anomalies, 619880 (3), Autosomal recessive
PCGF2	600346	Turnpenny-Fry syndrome, 618371 (3), Autosomal dominant
PCK1	614168	?Phosphoenolpyruvate carboxykinase deficiency, cytosolic, 261680 (3), Autosomal recessive
PCK2	614095	PEPCK deficiency, mitochondrial, 261650 (1), Autosomal recessive
PCLO	604918	?Pontocerebellar hypoplasia, type 3, 608027 (3), Autosomal recessive
PCNA	176740	?Ataxia-telangiectasia-like disorder 2, 615919 (3), Autosomal recessive
PCNT	605925	Microcephalic osteodysplastic primordial dwarfism, type II, 210720 (3), Autosomal recessive
PCSK1	162150	Obesity with impaired prohormone processing, 600955 (3), Autosomal recessive; {Obesity, susceptibility to, BMIQ12}, 612362 (3)
PCSK9	607786	{Low density lipoprotein cholesterol level QTL 1}, 603776 (3), Autosomal dominant; Hypercholesterolemia, familial, 3, 603776 (3), Autosomal dominant
PCYT1A	123695	Spondylometaphyseal dysplasia with cone-rod dystrophy, 608940 (3), Autosomal recessive
PCYT2	602679	Spastic paraplegia 82, autosomal recessive, 618770 (3), Autosomal recessive
PDCD1	600244	{Multiple sclerosis, disease progression, modifier of}, 126200 (3), Multifactorial; {Systemic lupus erythematosus, susceptibility to, 2}, 605218 (3)
PDCD10	609118	Cerebral cavernous malformations-3, 603285 (3), Autosomal dominant
PDE10A	610652	Striatal degeneration, autosomal dominant, 616922 (3), Autosomal dominant; Dyskinesia, limb and orofacial, infantile-onset, 616921 (3), Autosomal recessive

PDE11A	604961	Pigmented nodular adrenocortical disease, primary, 2, 610475 (3), Autosomal dominant
PDE1C	602987	?Deafness, autosomal dominant 74, 618140 (3), Autosomal dominant
PDE2A	602658	Intellectual developmental disorder with paroxysmal dyskinesia or seizures, 619150 (3), Autosomal recessive
PDE3A	123805	Hypertension and brachydactyly syndrome, 112410 (3), Autosomal dominant
PDE4D	600129	Acrodysostosis 2, with or without hormone resistance, 614613 (3), Autosomal dominant
PDE6A	180071	Retinitis pigmentosa 43, 613810 (3)
PDE6B	180072	Retinitis pigmentosa-40, 613801 (3), Autosomal recessive; Night blindness, congenital stationary, autosomal dominant 2, 163500 (3), Autosomal dominant
PDE6C	600827	Cone dystrophy 4, 613093 (3), Autosomal recessive
PDE6D	602676	Joubert syndrome 22, 615665 (3), Autosomal recessive
PDE6G	180073	Retinitis pigmentosa 57, 613582 (3), Autosomal recessive
PDE6H	601190	Retinal cone dystrophy 3, 610024 (3), Autosomal dominant, Autosomal recessive; Achromatopsia 6, 610024 (3), Autosomal dominant, Autosomal recessive
PDE8B	603390	Pigmented nodular adrenocortical disease, primary, 3, 614190 (3); Striatal degeneration, autosomal dominant, 609161 (3), Autosomal dominant
PDGFB	190040	Meningioma, SIS-related, 607174 (3), Autosomal dominant; Basal ganglia calcification, idiopathic, 5, 615483 (3), Autosomal dominant; Dermatofibrosarcoma protuberans, 607907 (3)
PDGFRA	173490	Gastrointestinal stromal tumor/GIST-plus syndrome, somatic or familial, 175510 (3); Hypereosinophilic syndrome, idiopathic, resistant to imatinib, 607685 (3), Isolated cases, Somatic mutation
PDGFRB	173410	Premature aging syndrome, Penttinen type, 601812 (3), Autosomal dominant; Kosaki overgrowth syndrome, 616592 (3), Autosomal dominant; Myofibromatosis, infantile, 1, 228550 (3), Autosomal dominant; Basal ganglia calcification, idiopathic, 4, 615007 (3), Autosomal dominant; Myeloproliferative disorder with eosinophilia, 131440 (4), Autosomal dominant
PDGFRL	604584	Hepatocellular cancer, somatic, 114550 (3); Colorectal cancer, somatic, 114500 (3)
PDHA1	300502	Pyruvate dehydrogenase E1-alpha deficiency, 312170 (3), X-linked dominant
PDHA2	179061	Spermatogenic failure 70, 619828 (3), Autosomal recessive
PDHB	179060	Pyruvate dehydrogenase E1-beta deficiency, 614111 (3), Autosomal recessive
PDHX	608769	Lacticacidemia due to PDX1 deficiency, 245349 (3), Autosomal recessive
PDK3	300906	?Charcot-Marie-Tooth disease, X-linked dominant, 6, 300905 (3), X-linked dominant
PDLIM4	603422	{Osteoporosis, susceptibility to}, 166710 (3), Autosomal dominant

PDP1	605993	Pyruvate dehydrogenase phosphatase deficiency, 608782 (3), Autosomal recessive
PDSS1	607429	Coenzyme Q10 deficiency, primary, 2, 614651 (3), Autosomal recessive
PDSS2	610564	Coenzyme Q10 deficiency, primary, 3, 614652 (3), Autosomal recessive
PDX1	600733	{Diabetes mellitus, type II, susceptibility to}, 125853 (3), Autosomal dominant; Pancreatic agenesis 1, 260370 (3), Autosomal recessive; MODY, type IV, 606392 (3)
PDXK	179020	Neuropathy, hereditary motor and sensory, type VIC, with optic atrophy, 618511 (3), Autosomal recessive
PDYN	131340	Spinocerebellar ataxia 23, 610245 (3), Autosomal dominant
PDZD7	612971	Deafness, autosomal recessive 57, 618003 (3), Autosomal recessive; {Retinal disease in Usher syndrome type IIA, modifier of}, 276901 (3), Autosomal recessive; Usher syndrome, type IIC, GPR98/PDZD7 digenic, 605472 (3), Digenic dominant, Autosomal recessive
PEPD	613230	Prolidase deficiency, 170100 (3), Autosomal recessive
PER2	603426	?Advanced sleep phase syndrome, familial, 1, 604348 (3), Autosomal dominant
PER3	603427	?Advanced sleep phase syndrome, familial, 3, 616882 (3), Autosomal dominant
PERCC1	618656	Diarrhea 11, malabsorptive, congenital, 618662 (3), Autosomal recessive
PERP	609301	Erythrokeratodermia variabilis et progressiva 7, 619209 (3), Autosomal recessive; Olmsted syndrome 2, 619208 (3), Autosomal dominant
PET100	614770	Mitochondrial complex IV deficiency, nuclear type 12, 619055 (3), Autosomal recessive
PET117	614771	?Mitochondrial complex IV deficiency, nuclear type 19, 619063 (3), Autosomal recessive
PEX1	602136	Heimler syndrome 1, 234580 (3), Autosomal recessive; Peroxisome biogenesis disorder 1B (NALD/IRD), 601539 (3), Autosomal recessive; Peroxisome biogenesis disorder 1A (Zellweger), 214100 (3), Autosomal recessive
PEX10	602859	Peroxisome biogenesis disorder 6A (Zellweger), 614870 (3), Autosomal recessive; Peroxisome biogenesis disorder 6B, 614871 (3), Autosomal recessive
PEX11B	603867	Peroxisome biogenesis disorder 14B, 614920 (3), Autosomal recessive
PEX12	601758	Peroxisome biogenesis disorder 3B, 266510 (3), Autosomal recessive; Peroxisome biogenesis disorder 3A (Zellweger), 614859 (3), Autosomal recessive
PEX13	601789	Peroxisome biogenesis disorder 11A (Zellweger), 614883 (3), Autosomal recessive; Peroxisome biogenesis disorder 11B, 614885 (3), Autosomal recessive
PEX14	601791	Peroxisome biogenesis disorder 13A (Zellweger), 614887 (3), Autosomal recessive

PEX16	603360	Peroxisome biogenesis disorder 8B, 614877 (3), Autosomal recessive; Peroxisome biogenesis disorder 8A (Zellweger), 614876 (3), Autosomal recessive
PEX19	600279	Peroxisome biogenesis disorder 12A (Zellweger), 614886 (3), Autosomal recessive
PEX2	170993	Peroxisome biogenesis disorder 5A (Zellweger), 614866 (3), Autosomal recessive; Peroxisome biogenesis disorder 5B, 614867 (3), Autosomal recessive
PEX26	608666	Peroxisome biogenesis disorder 7B, 614873 (3), Autosomal recessive; Peroxisome biogenesis disorder 7A (Zellweger), 614872 (3), Autosomal recessive
PEX3	603164	Peroxisome biogenesis disorder 10A (Zellweger), 614882 (3), Autosomal recessive; ?Peroxisome biogenesis disorder 10B, 617370 (3), Autosomal recessive
PEX5	600414	Peroxisome biogenesis disorder 2B, 202370 (3), Autosomal recessive; Peroxisome biogenesis disorder 2A (Zellweger), 214110 (3), Autosomal recessive; Rhizomelic chondrodysplasia punctata, type 5, 616716 (3), Autosomal recessive
PEX6	601498	Peroxisome biogenesis disorder 4B, 614863 (3), Autosomal dominant, Autosomal recessive; Peroxisome biogenesis disorder 4A (Zellweger), 614862 (3), Autosomal recessive; Heimler syndrome 2, 616617 (3), Autosomal recessive
PEX7	601757	Rhizomelic chondrodysplasia punctata, type 1, 215100 (3), Autosomal recessive; Peroxisome biogenesis disorder 9B, 614879 (3), Autosomal recessive
PFKL	171860	Hemolytic anemia due to phosphofructokinase deficiency (1)
PFKM	610681	Glycogen storage disease VII, 232800 (3), Autosomal recessive
PFN1	176610	Amyotrophic lateral sclerosis 18, 614808 (3)
PGAM2	612931	Glycogen storage disease X, 261670 (3), Autosomal recessive
PGAP1	611655	Neurodevelopmental disorder with dysmorphic features, spasticity, and brain abnormalities, 615802 (3), Autosomal recessive
PGAP2	615187	Hyperphosphatasia with mental retardation syndrome 3, 614207 (3), Autosomal recessive
PGAP3	611801	Hyperphosphatasia with mental retardation syndrome 4, 615716 (3), Autosomal recessive
PGK1	311800	Phosphoglycerate kinase 1 deficiency, 300653 (3), X-linked recessive
PGM1	171900	Congenital disorder of glycosylation, type It, 614921 (3), Autosomal recessive
PGM3	172100	Immunodeficiency 23, 615816 (3), Autosomal recessive
PGR	607311	?Progesterone resistance, 264080 (2), Autosomal recessive
PHACTR1	608723	Developmental and epileptic encephalopathy 70, 618298 (3), Autosomal dominant
PHB1 (PHB)	176705	{Breast cancer, susceptibility to}, 114480 (3), Autosomal dominant, Somatic mutation

PHC1	602978	?Microcephaly 11, primary, autosomal recessive, 615414 (3), Autosomal recessive
PHEX	300550	Hypophosphatemic rickets, X-linked dominant, 307800 (3), X-linked dominant
PHF21A	608325	Intellectual developmental disorder with behavioral abnormalities and craniofacial dysmorphism with or without seizures, 618725 (3), Autosomal dominant
PHF6	300414	Borjeson-Forssman-Lehmann syndrome, 301900 (3), X-linked recessive
PHF8	300560	Intellectual developmental disorder, X-linked syndromic, Siderius type, 300263 (3), X-linked recessive
PHGDH	606879	Neu-Laxova syndrome 1, 256520 (3), Autosomal recessive; Phosphoglycerate dehydrogenase deficiency, 601815 (3), Autosomal recessive
PHIP	612870	Chung-Jansen syndrome, 617991 (3), Autosomal dominant
PHKA1	311870	Muscle glycogenosis, 300559 (3), X-linked recessive
PHKA2	300798	Glycogen storage disease, type IXa2, 306000 (3), X-linked recessive; Glycogen storage disease, type IXa1, 306000 (3), X-linked recessive
PHKB	172490	Phosphorylase kinase deficiency of liver and muscle, autosomal recessive, 261750 (3), Autosomal recessive
PHKG2	172471	Glycogen storage disease IXc, 613027 (3), Autosomal recessive
PHOX2A	602753	Fibrosis of extraocular muscles, congenital, 2, 602078 (3), Autosomal recessive
PHOX2B	603851	{Neuroblastoma, susceptibility to, 2}, 613013 (3); Neuroblastoma with Hirschsprung disease, 613013 (3); Central hypoventilation syndrome, congenital, 1, with or without Hirschsprung disease, 209880 (3), Autosomal dominant
PHYH	602026	Refsum disease, 266500 (3), Autosomal recessive
PHYKPL	614683	[?Phosphohydroxylysinuria], 615011 (3)
PI4KA	600286	Spastic paraplegia 84, autosomal recessive, 619621 (3), Autosomal recessive; Gastrointestinal defects and immunodeficiency syndrome 2, 619708 (3), Autosomal recessive; Polymicrogyria, perisylvian, with cerebellar hypoplasia and arthrogryposis, 616531 (3), Autosomal recessive
PIBF1	607532	Joubert syndrome 33, 617767 (3), Autosomal recessive
PICALM	603025	Leukemia, acute myeloid, somatic, 601626 (3)
PIDD1	605247	Intellectual developmental disorder, autosomal recessive 75, with neuropsychiatric features and variant lissencephaly, 619827 (3), Autosomal recessive
PIEZO1	611184	Lymphatic malformation 6, 616843 (3), Autosomal recessive; Dehydrated hereditary stomatocytosis with or without pseudohyperkalemia and/or perinatal edema, 194380 (3), Autosomal dominant
PIEZO2	613629	Arthrogryposis, distal, type 5, 108145 (3), Autosomal dominant; Arthrogryposis, distal, with impaired proprioception and touch, 617146 (3), Autosomal recessive; Arthrogryposis, distal, type 3, 114300 (3), Autosomal dominant; ?Marden-Walker syndrome, 248700 (3), Autosomal dominant

PIGA	311770	Paroxysmal nocturnal hemoglobinuria, somatic, 300818 (3); Multiple congenital anomalies-hypotonia-seizures syndrome 2, 300868 (3), X-linked recessive; Neurodevelopmental disorder with epilepsy and hemochromatosis, 301072 (3)
PIGB	604122	Developmental and epileptic encephalopathy 80, 618580 (3), Autosomal recessive
PIGC	601730	Glycosylphosphatidylinositol biosynthesis defect 16, 617816 (3), Autosomal recessive
PIGF	600153	Onychodystrophy, osteodystrophy, impaired intellectual development, and seizures syndrome, 619356 (3), Autosomal recessive
PIGG	616918	[Blood group, EMM system], 619812 (3), Autosomal recessive; Neurodevelopmental disorder with or without hypotonia, seizures, and cerebellar atrophy, 616917 (3), Autosomal recessive
PIGH	600154	Glycosylphosphatidylinositol biosynthesis defect 17, 618010 (3), Autosomal recessive
PIGK	605087	Neurodevelopmental disorder with hypotonia and cerebellar atrophy, with or without seizures, 618879 (3), Autosomal recessive
PIGL	605947	CHIME syndrome, 280000 (3), Autosomal recessive
PIGM	610273	Glycosylphosphatidylinositol deficiency, 610293 (3), Autosomal recessive
PIGN	606097	Multiple congenital anomalies-hypotonia-seizures syndrome 1, 614080 (3), Autosomal recessive
PIGO	614730	Hyperphosphatasia with mental retardation syndrome 2, 614749 (3), Autosomal recessive
PIGP	605938	Developmental and epileptic encephalopathy 55, 617599 (3), Autosomal recessive
PIGQ	605754	Multiple congenital anomalies-hypotonia-seizures syndrome 4, 618548 (3), Autosomal recessive
PIGS	610271	Developmental and epileptic encephalopathy 95, 618143 (3), Autosomal recessive
PIGT	610272	?Paroxysmal nocturnal hemoglobinuria 2, 615399 (3), Autosomal dominant, Somatic mutation; Multiple congenital anomalies-hypotonia-seizures syndrome 3, 615398 (3), Autosomal recessive
PIGU	608528	Neurodevelopmental disorder with brain anomalies, seizures, and scoliosis, 618590 (3), Autosomal recessive
PIGV	610274	Hyperphosphatasia with mental retardation syndrome 1, 239300 (3), Autosomal recessive
PIGW	610275	Glycosylphosphatidylinositol biosynthesis defect 11, 616025 (3), Autosomal recessive
PIGY	610662	Hyperphosphatasia with mental retardation syndrome 6, 616809 (3), Autosomal recessive
PIK3C2A	603601	Oculoskeletodental syndrome, 618440 (3), Autosomal recessive

PIK3CA	171834	CLOVE syndrome, somatic, 612918 (3); Hepatocellular carcinoma, somatic, 114550 (3); Breast cancer, somatic, 114480 (3); Cerebral cavernous malformations 4, somatic, 619538 (3); Ovarian cancer, somatic, 167000 (3); Colorectal cancer, somatic, 114500 (3); Macrodactyly, somatic, 155500 (3); CLAPO syndrome, somatic, 613089 (3); Keratosis, seborrheic, somatic, 182000 (3); Nevus, epidermal, somatic, 162900 (3); Gastric cancer, somatic, 613659 (3); Nonsmall cell lung cancer, somatic, 211980 (3); Megalencephaly-capillary malformation-polymicrogyria syndrome, somatic, 602501 (3); Cowden syndrome 5, 615108 (3)
PIK3CD	602839	Immunodeficiency 14A, autosomal dominant, 615513 (3), Autosomal dominant; Immunodeficiency 14B, autosomal recessive, 619281 (3), Autosomal recessive; ?Roifman-Chitayat syndrome, digenic, 613328 (3), Digenic recessive
PIK3CG	601232	Immunodeficiency 97 with autoinflammation, 619802 (3), Autosomal recessive
PIK3R1	171833	Immunodeficiency 36, 616005 (3), Autosomal dominant; ?Agammaglobulinemia 7, autosomal recessive, 615214 (3), Autosomal recessive; SHORT syndrome, 269880 (3), Autosomal dominant
PIK3R2	603157	Megalencephaly-polymicrogyria-polydactyly-hydrocephalus syndrome 1, 603387 (3), Autosomal dominant
PIK3R5	611317	Ataxia-oculomotor apraxia 3, 615217 (3), Autosomal recessive
PIKFYVE	609414	Corneal fleck dystrophy, 121850 (3), Autosomal dominant
PINK1	608309	Parkinson disease 6, early onset, 605909 (3), Autosomal recessive
PIP5K1C	606102	Lethal congenital contractural syndrome 3, 611369 (3), Autosomal recessive
PISD	612770	Liberfarb syndrome, 618889 (3), Autosomal recessive
PITPNM3	608921	Cone-rod dystrophy 5, 600977 (3), Autosomal dominant
PITRM1	618211	Spinocerebellar ataxia, autosomal recessive 30, 619405 (3), Autosomal recessive
PITX1	602149	Liebenberg syndrome, 186550 (4), Autosomal dominant; Clubfoot, congenital, with or without deficiency of long bones and/or mirror-image polydactyly, 119800 (3), Autosomal dominant
PITX2	601542	Ring dermoid of cornea, 180550 (3), Autosomal dominant; Axenfeld-Rieger syndrome, type 1, 180500 (3), Autosomal dominant; Anterior segment dysgenesis 4, 137600 (3), Autosomal dominant
PITX3	602669	Cataract 11, multiple types, 610623 (3), Autosomal dominant, Autosomal recessive; Anterior segment dysgenesis 1, multiple subtypes, 107250 (3), Autosomal dominant; Cataract 11, syndromic, autosomal recessive, 610623 (3), Autosomal dominant, Autosomal recessive
PJVK	610219	Deafness, autosomal recessive 59, 610220 (3), Autosomal recessive
PKD1	601313	Polycystic kidney disease 1, 173900 (3), Autosomal dominant
PKD1L1	609721	Heterotaxy, visceral, 8, autosomal, 617205 (3), Autosomal recessive
PKD2	173910	Polycystic kidney disease 2, 613095 (3), Autosomal dominant

PKDCC	614150	Rhizomelic limb shortening with dysmorphic features, 618821 (3), Autosomal recessive
PKHD1	606702	Polycystic kidney disease 4, with or without hepatic disease, 263200 (3), Autosomal recessive
PKLR	609712	Adenosine triphosphate, elevated, of erythrocytes, 102900 (3), Autosomal dominant; Pyruvate kinase deficiency, 266200 (3), Autosomal recessive
PKP1	601975	Ectodermal dysplasia/skin fragility syndrome, 604536 (3), Autosomal recessive
PKP2	602861	Arrhythmogenic right ventricular dysplasia 9, 609040 (3), Autosomal dominant
PLA2G2A	172411	{?Colorectal cancer, susceptibility to}, 114500 (3), Autosomal dominant, Somatic mutation
PLA2G4A	600522	Gastrointestinal ulceration, recurrent, with dysfunctional platelets, 618372 (3), Autosomal recessive
PLA2G5	601192	[Fleck retina, familial benign], 228980 (3), Autosomal recessive
PLA2G6	603604	Parkinson disease 14, autosomal recessive, 612953 (3), Autosomal recessive; Neurodegeneration with brain iron accumulation 2B, 610217 (3), Autosomal recessive; Infantile neuroaxonal dystrophy 1, 256600 (3), Autosomal recessive
PLA2G7	601690	Platelet-activating factor acetylhydrolase deficiency, 614278 (3), Autosomal recessive; {Asthma, susceptibility to}, 600807 (3), Autosomal dominant; {Atopy, susceptibility to}, 147050 (3), Autosomal dominant
PLAA	603873	Neurodevelopmental disorder with progressive microcephaly, spasticity, and brain anomalies, 617527 (3), Autosomal recessive
PLAG1	603026	Adenomas, salivary gland pleomorphic, somatic, 181030 (3); Silver-Russell syndrome 4, 618907 (3), Autosomal dominant
PLAT	173370	Thrombophilia, familial, due to decreased release of PLAT, 612348 (1); Hyperfibrinolysis, familial, due to increased release of PLAT, 612348 (1)
PLAU	191840	Quebec platelet disorder, 601709 (3), Autosomal dominant; {Alzheimer disease, late-onset, susceptibility to}, 104300 (3), Autosomal dominant
PLCB1	607120	Developmental and epileptic encephalopathy 12, 613722 (3), Autosomal recessive
PLCB2	604114	Platelet PLC beta-2 deficiency (1)
PLCB3	600230	Spondylometaphyseal dysplasia with corneal dystrophy, 618961 (3), Autosomal recessive
PLCB4	600810	Auriculocondylar syndrome 2, 614669 (3), Autosomal dominant, Autosomal recessive
PLCD1	602142	Nail disorder, nonsyndromic congenital, 3, (leukonychia), 151600 (3), Autosomal dominant, Autosomal recessive
PLCE1	608414	Nephrotic syndrome, type 3, 610725 (3), Autosomal recessive
PLCG2	600220	Autoinflammation, antibody deficiency, and immune dysregulation syndrome, 614878 (3), Autosomal dominant; Familial cold autoinflammatory syndrome 3, 614468 (3), Autosomal dominant
PLCH1	612835	Holoprosencephaly 14, 619895 (3), Autosomal recessive

PLCZ1	608075	Spermatogenic failure 17, 617214 (3), Autosomal recessive
PLD1	602382	Cardiac valvular defect, developmental, 212093 (3), Autosomal recessive
PLD3	615698	?Spinocerebellar ataxia 46, 617770 (3), Autosomal dominant
PLEC	601282	?Epidermolysis bullosa simplex 5D, generalized intermediate, autosomal recessive, 616487 (3), Autosomal recessive; Epidermolysis bullosa simplex 5B, with muscular dystrophy, 226670 (3), Autosomal recessive; Epidermolysis bullosa simplex 5C, with pyloric atresia, 612138 (3), Autosomal recessive; Epidermolysis bullosa simplex 5A, Ogna type, 131950 (3), Autosomal dominant; Muscular dystrophy, limb-girdle, autosomal recessive 17, 613723 (3), Autosomal recessive
PLEKHG2	611893	Leukodystrophy and acquired microcephaly with or without dystonia, 616763 (3), Autosomal recessive
PLEKHG5	611101	Spinal muscular atrophy, distal, autosomal recessive, 4, 611067 (3), Autosomal recessive; Charcot-Marie-Tooth disease, recessive intermediate C, 615376 (3), Autosomal recessive
PLEKHM1	611466	?Osteopetrosis, autosomal recessive 6, 611497 (3), Autosomal recessive; Osteopetrosis, autosomal dominant 3, 618107 (3), Autosomal dominant
PLG	173350	Dysplasminogenemia, 217090 (3), Autosomal recessive; Angioedema, hereditary, 4, 619360 (3), Autosomal dominant; Plasminogen deficiency, type I, 217090 (3), Autosomal recessive
PLIN1	170290	Lipodystrophy, familial partial, type 4, 613877 (3), Autosomal dominant
PLK4	605031	Microcephaly and chorioretinopathy, autosomal recessive, 2, 616171 (3), Autosomal recessive
PLN	172405	Cardiomyopathy, dilated, 1P, 609909 (3); Cardiomyopathy, hypertrophic, 18, 613874 (3), Autosomal dominant
PLOD1	153454	Ehlers-Danlos syndrome, kyphoscoliotic type, 1, 225400 (3), Autosomal recessive
PLOD2	601865	Bruck syndrome 2, 609220 (3), Autosomal recessive
PLOD3	603066	Lysyl hydroxylase 3 deficiency, 612394 (3), Autosomal recessive
PLP1	300401	Pelizaeus-Merzbacher disease, 312080 (3), X-linked recessive; Spastic paraplegia 2, X-linked, 312920 (3), X-linked recessive
PLPBP	604436	Epilepsy, early-onset, vitamin B6-dependent, 617290 (3), Autosomal recessive
PLS1	602734	Deafness, autosomal dominant 76, 618787 (3), Autosomal dominant
PLS3	300131	Bone mineral density QTL18, osteoporosis, 300910 (3), X-linked dominant
PLTP	172425	[High density lipoprotein cholesterol level QTL 9] (3)
PLVAP	607647	Diarrhea 10, protein-losing enteropathy type, 618183 (3), Autosomal recessive
PLXNA1	601055	Dworschak-Punetha neurodevelopmental syndrome, 619955 (3), Autosomal recessive
PMFBP1	618085	Spermatogenic failure 31, 618112 (3), Autosomal recessive
PML	102578	Leukemia, acute promyelocytic, PML/RARA type (3)
PMM2	601785	Congenital disorder of glycosylation, type Ia, 212065 (3), Autosomal recessive

PMP2	170715	Charcot-Marie-Tooth disease, demyelinating, type 1G, 618279 (3), Autosomal dominant
PMP22	601097	Charcot-Marie-Tooth disease, type 1A, 118220 (3), Autosomal dominant; Roussy-Levy syndrome, 180800 (3), Autosomal dominant; Charcot-Marie-Tooth disease, type 1E, 118300 (3), Autosomal dominant; ?Neuropathy, inflammatory demyelinating, 139393 (3), ?Autosomal dominant; Neuropathy, recurrent, with pressure palsies, 162500 (3), Autosomal dominant; Dejerine-Sottas disease, 145900 (3), Autosomal dominant, Autosomal recessive
PMPCA	613036	Spinocerebellar ataxia, autosomal recessive 2, 213200 (3), Autosomal recessive
PMPCB	603131	Multiple mitochondrial dysfunctions syndrome 6, 617954 (3), Autosomal recessive
PMS2	600259	Colorectal cancer, hereditary nonpolyposis, type 4, 614337 (3); Mismatch repair cancer syndrome 4, 619101 (3), Autosomal recessive
PMVK	607622	Porokeratosis 1, multiple types, 175800 (3), Autosomal dominant
PNKD	609023	Paroxysmal nonkinesigenic dyskinesia 1, 118800 (3), Autosomal dominant
PNKP	605610	?Charcot-Marie-Tooth disease, type 2B2, 605589 (3), Autosomal recessive; Ataxia-oculomotor apraxia 4, 616267 (3), Autosomal recessive; Microcephaly, seizures, and developmental delay, 613402 (3), Autosomal recessive
PNLDC1	619529	Spermatogenic failure 57, 619528 (3), Autosomal recessive
PNLIP	246600	?Pancreatic lipase deficiency, 614338 (3), Autosomal recessive
PNP	164050	Immunodeficiency due to purine nucleoside phosphorylase deficiency, 613179 (3), Autosomal recessive
PNPLA1	612121	Ichthyosis, congenital, autosomal recessive 10, 615024 (3), Autosomal recessive
PNPLA2	609059	Neutral lipid storage disease with myopathy, 610717 (3), Autosomal recessive
PNPLA6	603197	Spastic paraplegia 39, autosomal recessive, 612020 (3), Autosomal recessive; Oliver-McFarlane syndrome, 275400 (3), Autosomal recessive; ?Laurence-Moon syndrome, 245800 (3), Autosomal recessive; Boucher-Neuhauser syndrome, 215470 (3), Autosomal recessive
PNPLA8	612123	?Mitochondrial myopathy with lactic acidosis, 251950 (3), Autosomal recessive
PNPO	603287	Pyridoxamine 5'-phosphate oxidase deficiency, 610090 (3), Autosomal recessive
PNPT1	610316	Spinocerebellar ataxia 25, 608703 (3), Autosomal dominant; Deafness, autosomal recessive 70, with or without adult-onset neurodegeneration, 614934 (3), Autosomal recessive; Combined oxidative phosphorylation deficiency 13, 614932 (3), Autosomal recessive
POC1A	614783	Short stature, onychodysplasia, facial dysmorphism, and hypotrichosis, 614813 (3), Autosomal recessive
POC1B	614784	Cone-rod dystrophy 20, 615973 (3), Autosomal recessive

POF1B	300603	?Premature ovarian failure 2B, 300604 (3), X-linked recessive
POFUT1	607491	Dowling-Degos disease 2, 615327 (3), Autosomal dominant
POGLUT1	615618	Dowling-Degos disease 4, 615696 (3), Autosomal dominant; ?Muscular dystrophy, limb-girdle, autosomal recessive 21, 617232 (3), Autosomal recessive
POGZ	614787	White-Sutton syndrome, 616364 (3), Autosomal dominant
POLA1	312040	Pigmentary disorder, reticulate, with systemic manifestations, X-linked, 301220 (3), X-linked recessive; Van Esch-O'Driscoll syndrome, 301030 (3), X-linked recessive
POLD1	174761	Mandibular hypoplasia, deafness, progeroid features, and lipodystrophy syndrome, 615381 (3), Autosomal dominant; {Colorectal cancer, susceptibility to, 10}, 612591 (3), Autosomal dominant
POLE	174762	{Colorectal cancer, susceptibility to, 12}, 615083 (3), Autosomal dominant; FILS syndrome, 615139 (3), Autosomal recessive; IMAGE-I syndrome, 618336 (3), Autosomal recessive
POLG	174763	Mitochondrial recessive ataxia syndrome (includes SANDO and SCAE), 607459 (3), Autosomal recessive; Mitochondrial DNA depletion syndrome 4B (MNGIE type), 613662 (3), Autosomal recessive; Mitochondrial DNA depletion syndrome 4A (Alpers type), 203700 (3), Autosomal recessive; Progressive external ophthalmoplegia, autosomal dominant 1, 157640 (3), Autosomal dominant; Progressive external ophthalmoplegia, autosomal recessive 1, 258450 (3), Autosomal recessive
POLG2	604983	Progressive external ophthalmoplegia with mitochondrial DNA deletions, autosomal dominant 4, 610131 (3), Autosomal dominant; ?Mitochondrial DNA depletion syndrome 16 (hepatic type), 618528 (3), Autosomal recessive; ?Mitochondrial DNA depletion syndrome 16B (neuroophthalmic type), 619425 (3), Autosomal recessive
POLH	603968	Xeroderma pigmentosum, variant type, 278750 (3), Autosomal recessive
POLR1A	616404	Acrofacial dysostosis, Cincinnati type, 616462 (3), Autosomal dominant
POLR1B	602000	Treacher-Collins syndrome 4, 618939 (3), Autosomal dominant
POLR1C	610060	Leukodystrophy, hypomyelinating, 11, 616494 (3), Autosomal recessive; Treacher Collins syndrome 3, 248390 (3), Autosomal recessive
POLR1D	613715	Treacher Collins syndrome 2, 613717 (3), Autosomal dominant, Autosomal recessive
POLR2A	180660	Neurodevelopmental disorder with hypotonia and variable intellectual and behavioral abnormalities, 618603 (3), Autosomal dominant
POLR3A	614258	Wiedemann-Rautenstrauch syndrome, 264090 (3), Autosomal recessive; Leukodystrophy, hypomyelinating, 7, with or without oligodontia and/or hypogonadotropic hypogonadism, 607694 (3), Autosomal recessive
POLR3B	614366	Leukodystrophy, hypomyelinating, 8, with or without oligodontia and/or hypogonadotropic hypogonadism, 614381 (3), Autosomal recessive; Charcot-Marie-Tooth disease, demyelinating, type 1I, 619742 (3), Autosomal dominant

POLR3F	617455	?Immunodeficiency 101 (varicella zoster virus-specific), 619872 (3), Autosomal dominant
POLR3GL	617457	Short stature, oligodontia, dysmorphic facies, and motor delay, 619234 (3), Autosomal recessive
POLR3K	606007	Leukodystrophy, hypomyelinating, 21, 619310 (3), Autosomal recessive
POLRMT	601778	Combined oxidative phosphorylation deficiency 55, 619743 (3), Autosomal dominant, Autosomal recessive
POMC	176830	{Obesity, early-onset, susceptibility to}, 601665 (3), Multifactorial, Autosomal dominant, Autosomal recessive; Obesity, adrenal insufficiency, and red hair due to POMC deficiency, 609734 (3), Autosomal recessive
POMGNT1	606822	Muscular dystrophy-dystroglycanopathy (congenital with mental retardation), type B, 3, 613151 (3), Autosomal recessive; Muscular dystrophy-dystroglycanopathy (limb-girdle), type C, 3, 613157 (3), Autosomal recessive; Retinitis pigmentosa 76, 617123 (3), Autosomal recessive; Muscular dystrophy-dystroglycanopathy (congenital with brain and eye anomalies), type A, 3, 253280 (3), Autosomal recessive
POMGNT2	614828	Muscular dystrophy-dystroglycanopathy (congenital with brain and eye anomalies), type A, 8, 614830 (3), Autosomal recessive; Muscular dystrophy-dystroglycanopathy (limb-girdle) type C, 8, 618135 (3), Autosomal recessive
POMK	615247	?Muscular dystrophy-dystroglycanopathy (limb-girdle), type C, 12, 616094 (3), Autosomal recessive; Muscular dystrophy-dystroglycanopathy (congenital with brain and eye anomalies), type A, 12, 615249 (3), Autosomal recessive
POMP	613386	Proteasome-associated autoinflammatory syndrome 2, 618048 (3), Autosomal dominant; Keratosis linearis with ichthyosis congenita and sclerosing keratoderma, 601952 (3), Autosomal recessive
POMT1	607423	Muscular dystrophy-dystroglycanopathy (congenital with brain and eye anomalies), type A, 1, 236670 (3), Autosomal recessive; Muscular dystrophy-dystroglycanopathy (limb-girdle), type C, 1, 609308 (3), Autosomal recessive; Muscular dystrophy-dystroglycanopathy (congenital with mental retardation), type B, 1, 613155 (3), Autosomal recessive
POMT2	607439	Muscular dystrophy-dystroglycanopathy (limb-girdle), type C, 2, 613158 (3), Autosomal recessive; Muscular dystrophy-dystroglycanopathy (congenital with mental retardation), type B, 2, 613156 (3), Autosomal recessive; Muscular dystrophy-dystroglycanopathy (congenital with brain and eye anomalies), type A, 2, 613150 (3), Autosomal recessive
PON1	168820	{Coronary artery spasm 2, susceptibility to} (3); {Organophosphate poisoning, sensitivity to} (3); {Coronary artery disease, susceptibility to} (3); {Microvascular complications of diabetes 5}, 612633 (3)
PON2	602447	{Coronary artery disease, susceptibility to} (3)
POP1	602486	Anauxetic dysplasia 2, 617396 (3), Autosomal recessive
POPDC3	605824	Muscular dystrophy, limb-girdle, autosomal recessive 26, 618848 (3), Autosomal recessive

POR	124015	Antley-Bixler syndrome with genital anomalies and disordered steroidogenesis, 201750 (3), Autosomal recessive; Disordered steroidogenesis due to cytochrome P450 oxidoreductase, 613571 (3)
PORCN	300651	Focal dermal hypoplasia, 305600 (3), X-linked dominant
POT1	606478	{Glioma susceptibility 9}, 616568 (3), Autosomal dominant; {Melanoma, cutaneous malignant, susceptibility to, 10}, 615848 (3), Autosomal dominant
POU1F1	173110	Pituitary hormone deficiency, combined or isolated, 1, 613038 (3), Autosomal dominant, Autosomal recessive
POU3F3	602480	Snijders Blok-Fisher syndrome, 618604 (3), Autosomal dominant
POU3F4	300039	Deafness, X-linked 2, 304400 (3), X-linked recessive
POU4F1	601632	Ataxia, intention tremor, and hypotonia syndrome, childhood-onset, 619352 (3), Autosomal dominant
POU4F3	602460	Deafness, autosomal dominant 15, 602459 (3), Autosomal dominant
POU6F2	609062	{Wilms tumor susceptibility-5}, 601583 (3), Autosomal dominant, Somatic mutation
PPA2	609988	?Sudden cardiac failure, alcohol-induced, 617223 (3), Autosomal recessive; Sudden cardiac failure, infantile, 617222 (3), Autosomal recessive
PPARA	170998	{Hyperapobetalipoproteinemia, susceptibility to} (3)
PPARG	601487	{Diabetes, type 2}, 125853 (3), Autosomal dominant; Insulin resistance, severe, digenic, 604367 (3), Autosomal dominant; Lipodystrophy, familial partial, type 3, 604367 (3), Autosomal dominant; [Obesity, resistance to] (3); Obesity, severe, 601665 (3), Multifactorial, Autosomal dominant, Autosomal recessive; Carotid intimal medial thickness 1, 609338 (3)
PPCS	609853	Cardiomyopathy, dilated, 2C, 618189 (3), Autosomal recessive
PPIB	123841	Osteogenesis imperfecta, type IX, 259440 (3), Autosomal recessive
PPIL1	601301	Pontocerebellar hypoplasia, type 14, 619301 (3), Autosomal recessive
PPIP5K2	611648	Deafness, autosomal recessive 100, 618422 (3), Autosomal recessive
PPM1D	605100	Breast cancer, somatic, 114480 (3); Jansen de Vries syndrome, 617450 (3), Autosomal dominant
PPM1K	611065	?Maple syrup urine disease, mild variant, 615135 (3)
PPOX	600923	Porphyria variegata, 176200 (3), Autosomal dominant
PPP1CB	600590	Noonan syndrome-like disorder with loose anagen hair 2, 617506 (3), Autosomal dominant
PPP1R12A	602021	Genitourinary and/or/brain malformation syndrome, 618820 (3), Autosomal dominant
PPP1R15B	613257	Microcephaly, short stature, and impaired glucose metabolism 2, 616817 (3), Autosomal recessive
PPP1R17	604088	{Hypercholesterolemia, susceptibility to}, 143890 (3), Autosomal dominant, Autosomal recessive
PPP1R21	618159	Neurodevelopmental disorder with hypotonia, facial dysmorphism, and brain abnormalities, 619383 (3), Autosomal recessive
PPP1R3A	600917	Insulin resistance, severe, digenic, 125853 (3), Autosomal dominant

PPP2CA	176915	Neurodevelopmental disorder and language delay with or without structural brain abnormalities, 618354 (3), Autosomal dominant
PPP2R1A	605983	Intellectual developmental disorder, autosomal dominant 36, 616362 (3), Autosomal dominant
PPP2R1B	603113	Lung cancer, somatic, 211980 (3)
PPP2R2B	604325	Spinocerebellar ataxia 12, 604326 (3), Autosomal dominant
PPP2R3C	615902	Gonadal dysgenesis, dysmorphic facies, retinal dystrophy, and myopathy, 618419 (3), Autosomal recessive; Spermatogenic failure 36, 618420 (3), Autosomal dominant
PPP2R5D	601646	Intellectual developmental disorder, autosomal dominant 35, 616355 (3), Autosomal dominant
PPP3CA	114105	Arthrogryposis, cleft palate, craniosynostosis, and impaired intellectual development, 618265 (3), Autosomal dominant; Developmental and epileptic encephalopathy 91, 617711 (3), Autosomal dominant
PPT1	600722	Ceroid lipofuscinosis, neuronal, 1, 256730 (3), Autosomal recessive
PQBP1	300463	Renpenning syndrome, 309500 (3), X-linked recessive
PRCC	179755	Renal cell carcinoma, papillary, 605074 (3)
PRCD	610598	Retinitis pigmentosa 36, 610599 (3)
PRDM12	616458	Neuropathy, hereditary sensory and autonomic, type VIII, 616488 (3), Autosomal recessive
PRDM13	616741	Pontocerebellar hypoplasia, type 17, 619909 (3), Autosomal recessive; Cerebellar dysfunction, impaired intellectual development, and hypogonadotropic hypogonadism, 619761 (3), Autosomal recessive
PRDM16	605557	Left ventricular noncompaction 8, 615373 (3), Autosomal dominant; Cardiomyopathy, dilated, 1LL, 615373 (3), Autosomal dominant
PRDM5	614161	Brittle cornea syndrome 2, 614170 (3), Autosomal recessive
PRDM6	616982	Patent ductus arteriosus 3, 617039 (3), Autosomal dominant
PRDM8	616639	?Epilepsy, progressive myoclonic, 10, 616640 (3), Autosomal recessive
PRDX1	176763	Methylmalonic aciduria and homocystinuria, cblC type, digenic, 277400 (3), Autosomal recessive
PRDX3	604769	Spinocerebellar ataxia, autosomal recessive 32, 619862 (3); Corneal dystrophy, punctiform and polychromatic pre-Descemet, 619871 (3), Autosomal dominant
PREPL	609557	Myasthenic syndrome, congenital, 22, 616224 (3), Autosomal recessive
PRF1	170280	Hemophagocytic lymphohistiocytosis, familial, 2, 603553 (3), Autosomal recessive; Aplastic anemia, 609135 (3); Lymphoma, non-Hodgkin, 605027 (3)
PRG4	604283	Camptodactyly-arthropathy-coxa vara-pericarditis syndrome, 208250 (3), Autosomal recessive
PRICKLE1	608500	Epilepsy, progressive myoclonic 1B, 612437 (3), Autosomal recessive
PRICKLE3	300111	{Leber hereditary optic neuropathy, modifier of}, 308905 (3), X-linked dominant
PRIM1	176635	Primordial dwarfism-immunodeficiency-lipodystrophy syndrome, 620005 (3), Autosomal recessive

PRIMPOL	615421	Myopia 22, autosomal dominant, 615420 (3), Autosomal dominant
PRKACA	601639	Cushing syndrome, ACTH-independent adrenal, somatic, 615830 (3); Cardioacrofacial dysplasia 1, 619142 (3), Autosomal dominant
PRKACB	176892	Cardioacrofacial dysplasia 2, 619143 (3), Autosomal dominant, Somatic mosaicism
PRKACG	176893	?Bleeding disorder, platelet-type, 19, 616176 (3), Autosomal recessive
PRKAG2	602743	Glycogen storage disease of heart, lethal congenital, 261740 (3), Autosomal dominant; Wolff-Parkinson-White syndrome, 194200 (3), Autosomal dominant; Cardiomyopathy, hypertrophic 6, 600858 (3), Autosomal dominant
PRKAG3	604976	[Skeletal muscle glycogen content and metabolism QTL], 619030 (3), Autosomal recessive
PRKAR1A	188830	Pigmented nodular adrenocortical disease, primary, 1, 610489 (3), Autosomal dominant; Acrodysostosis 1, with or without hormone resistance, 101800 (3), Autosomal dominant; Adrenocortical tumor, somatic (3); Carney complex, type 1, 160980 (3), Autosomal dominant; Myxoma, intracardiac, 255960 (3), Autosomal dominant
PRKAR1B	176911	Marbach-Schaaf neurodevelopmental syndrome, 619680 (3), Autosomal dominant
PRKCA	176960	Pituitary tumor, invasive (3)
PRKCD	176977	Autoimmune lymphoproliferative syndrome, type III, 615559 (3), Autosomal recessive
PRKCG	176980	Spinocerebellar ataxia 14, 605361 (3), Autosomal dominant
PRKCH	605437	{Cerebral infarction, susceptibility to}, 601367 (3), Multifactorial
PRKCSH	177060	Polycystic liver disease 1, 174050 (3), Autosomal dominant
PRKD1	605435	Congenital heart defects and ectodermal dysplasia, 617364 (3), Autosomal dominant
PRKDC	600899	Immunodeficiency 26, with or without neurologic abnormalities, 615966 (3), Autosomal recessive
PRKG1	176894	Aortic aneurysm, familial thoracic 8, 615436 (3), Autosomal dominant
PRKG2	601591	Spondylometaphyseal dysplasia, Pagnamenta type, 619638 (3), Autosomal recessive; Acromesomelic dysplasia 4, 619636 (3), Autosomal recessive
PRKN	602544	Adenocarcinoma of lung, somatic, 211980 (3); Parkinson disease, juvenile, type 2, 600116 (3), Autosomal recessive; Ovarian cancer, somatic, 167000 (3)
PRKRA	603424	Dystonia 16, 612067 (3), Autosomal recessive
PRLR	176761	Multiple fibroadenomas of the breast, 615554 (3), Autosomal dominant; Hyperprolactinemia, 615555 (3), Autosomal dominant, Autosomal recessive
PRMT7	610087	Short stature, brachydactyly, intellectual developmental disability, and seizures, 617157 (3), Autosomal recessive

PRNP	176640	Spongiform encephalopathy with neuropsychiatric features, 606688 (3), Autosomal dominant; Gerstmann-Straussler disease, 137440 (3), Autosomal dominant; Huntington disease-like 1, 603218 (3), Autosomal dominant; Insomnia, fatal familial, 600072 (3), Autosomal dominant; {Kuru, susceptibility to}, 245300 (3); Cerebral amyloid angiopathy, PRNP-related, 137440 (3), Autosomal dominant; Creutzfeldt-Jakob disease, 123400 (3), Autosomal dominant
PROC	612283	Thrombophilia 3 due to protein C deficiency, autosomal dominant, 176860 (3), Autosomal dominant; Thrombophilia 3 due to protein C deficiency, autosomal recessive, 612304 (3), Autosomal recessive
PRODH	606810	{Schizophrenia, susceptibility to, 4}, 600850 (3), Autosomal dominant; Hyperprolinemia, type I, 239500 (3), Autosomal recessive
PROK2	607002	Hypogonadotropic hypogonadism 4 with or without anosmia, 610628 (3), Autosomal dominant
PROKR2	607123	Hypogonadotropic hypogonadism 3 with or without anosmia, 244200 (3), Autosomal dominant
PROM1	604365	Macular dystrophy, retinal, 2, 608051 (3), Autosomal dominant; Retinitis pigmentosa 41, 612095 (3), Autosomal recessive; Stargardt disease 4, 603786 (3), Autosomal dominant; Cone-rod dystrophy 12, 612657 (3), Autosomal dominant, Autosomal recessive
PROP1	601538	Pituitary hormone deficiency, combined, 2, 262600 (3), Autosomal recessive
PRORP	609947	Combined oxidative phosphorylation deficiency 54, 619737 (3), Autosomal recessive
PROS1	176880	Thrombophilia 5 due to protein S deficiency, autosomal recessive, 614514 (3), Autosomal recessive; Thrombophilia 5 due to protein S deficiency, autosomal dominant, 612336 (3), Autosomal dominant
PROZ	176895	[Protein Z deficiency], 614024 (3)
PRPF3	607301	Retinitis pigmentosa 18, 601414 (3), Autosomal dominant
PRPF31	606419	Retinitis pigmentosa 11, 600138 (3), Autosomal dominant
PRPF4	607795	Retinitis pigmentosa 70, 615922 (3), Autosomal dominant
PRPF6	613979	Retinitis pigmentosa 60, 613983 (3), Autosomal dominant
PRPF8	607300	Retinitis pigmentosa 13, 600059 (3), Autosomal dominant
PRPH	170710	{Amyotrophic lateral sclerosis, susceptibility to}, 105400 (3), Autosomal dominant, Autosomal recessive
PRPH2	179605	Macular dystrophy, patterned, 1, 169150 (3), Autosomal dominant; Choroidal dystrophy, central areolar 2, 613105 (3), Autosomal dominant; Retinitis punctata albescens, 136880 (3), Autosomal dominant, Autosomal recessive; Leber congenital amaurosis 18, 608133 (3), Digenic dominant, Autosomal dominant, Autosomal recessive; Macular dystrophy, vitelliform, 3, 608161 (3), Autosomal dominant; Retinitis pigmentosa 7 and digenic form, 608133 (3), Digenic dominant, Autosomal dominant, Autosomal recessive

PRPS1	311850	Arts syndrome, 301835 (3), X-linked recessive; Phosphoribosylpyrophosphate synthetase superactivity, 300661 (3), X-linked recessive; Charcot-Marie-Tooth disease, X-linked recessive, 5, 311070 (3), X-linked recessive; Deafness, X-linked 1, 304500 (3), X-linked; Gout, PRPS-related, 300661 (3), X-linked recessive
PRR12	616633	Neuroocular syndrome, 619539 (3), Autosomal dominant
PRRT2	614386	Convulsions, familial infantile, with paroxysmal choreoathetosis, 602066 (3), Autosomal dominant; Seizures, benign familial infantile, 2, 605751 (3), Autosomal dominant; Episodic kinesigenic dyskinesia 1, 128200 (3), Autosomal dominant
PRRX1	167420	Agnathia-otocephaly complex, 202650 (3), Autosomal dominant, Autosomal recessive
PRSS1	276000	Pancreatitis, hereditary, 167800 (3), Autosomal dominant
PRSS12	606709	Intellectual developmental disorder, autosomal recessive 1, 249500 (3), Autosomal recessive
PRSS2	601564	{Pancreatitis, chronic, protection against}, 167800 (3), Autosomal dominant
PRSS56	613858	Microphthalmia, isolated 6, 613517 (3), Autosomal recessive
PRUNE1	617413	Neurodevelopmental disorder with microcephaly, hypotonia, and variable brain anomalies, 617481 (3), Autosomal recessive
PRX	605725	Charcot-Marie-Tooth disease, type 4F, 614895 (3), Autosomal recessive; Dejerine-Sottas disease, 145900 (3), Autosomal dominant, Autosomal recessive
PSAP	176801	Combined SAP deficiency, 611721 (3), Autosomal recessive; Krabbe disease, atypical, 611722 (3), Autosomal recessive; Metachromatic leukodystrophy due to SAP-b deficiency, 249900 (3), Autosomal recessive; Gaucher disease, atypical, 610539 (3); {Parkinson disease 24, autosomal dominant, susceptibility to}, 619491 (3), Autosomal dominant
PSAT1	610936	Neu-Laxova syndrome 2, 616038 (3), Autosomal recessive; ?Phosphoserine aminotransferase deficiency, 610992 (3), Autosomal recessive
PSEN1	104311	Pick disease, 172700 (3), Autosomal dominant; Alzheimer disease, type 3, with spastic paraparesis and apraxia, 607822 (3), Autosomal dominant; Dementia, frontotemporal, 600274 (3), Autosomal dominant; ?Acne inversa, familial, 3, 613737 (3), Autosomal dominant; Cardiomyopathy, dilated, 1U, 613694 (3), Autosomal dominant; Alzheimer disease, type 3, with spastic paraparesis and unusual plaques, 607822 (3), Autosomal dominant; Alzheimer disease, type 3, 607822 (3), Autosomal dominant
PSEN2	600759	Alzheimer disease-4, 606889 (3), Autosomal dominant; Cardiomyopathy, dilated, 1V, 613697 (3), Autosomal dominant
PSENEN	607632	Acne inversa, familial, 2, with or without Dowling-Degos disease, 613736 (3), Autosomal dominant
PSMA6	602855	{Myocardial infarction, susceptibility to}, 608446 (3)
PSMB10	176847	Proteasome-associated autoinflammatory syndrome 5, 619175 (3), Autosomal recessive

PSMB4	602177	?Proteasome-associated autoinflammatory syndrome 3 and digenic forms, 617591 (3), Autosomal recessive
PSMB8	177046	Proteasome-associated autoinflammatory syndrome 1 and digenic forms, 256040 (3), Autosomal recessive
PSMB9	177045	?Proteasome-associated autoinflammatory syndrome 3, digenic, 617591 (3), Autosomal recessive
PSMC3	186852	?Deafness, cataract, impaired intellectual development, and polyneuropathy, 619354 (3), Autosomal recessive
PSMC3IP	608665	Ovarian dysgenesis 3, 614324 (3), Autosomal recessive
PSMD12	604450	Stankiewicz-Isidor syndrome, 617516 (3), Autosomal dominant
PSMG2	609702	?Proteasome-associated autoinflammatory syndrome 4, 619183 (3), Autosomal recessive
PSPH	172480	Phosphoserine phosphatase deficiency, 614023 (3), Autosomal recessive
PSTPIP1	606347	Pyogenic sterile arthritis, pyoderma gangrenosum, and acne, 604416 (3), Autosomal dominant
PTCD3	614918	?Combined oxidative phosphorylation deficiency 51, 619057 (3), Autosomal recessive
PTCH1	601309	Basal cell carcinoma, somatic, 605462 (3); Holoprosencephaly 7, 610828 (3), Autosomal dominant; Basal cell nevus syndrome, 109400 (3), Autosomal dominant
PTCH2	603673	Medulloblastoma, somatic, 155255 (3); Basal cell nevus syndrome, 109400 (3), Autosomal dominant; Basal cell carcinoma, somatic, 605462 (3)
PTCHD1	300828	{Autism, susceptibility to, X-linked 4}, 300830 (3), X-linked recessive
PTDSS1	612792	Lenz-Majewski hyperostotic dwarfism, 151050 (3), Autosomal dominant
PTEN	601728	{Glioma susceptibility 2}, 613028 (3); {Meningioma}, 607174 (3), Autosomal dominant; Cowden syndrome 1, 158350 (3), Autosomal dominant; Lhermitte-Duclos disease, 158350 (3), Autosomal dominant; Prostate cancer, somatic, 176807 (3); Macrocephaly/autism syndrome, 605309 (3), Autosomal dominant
PTF1A	607194	Pancreatic and cerebellar agenesis, 609069 (3), Autosomal recessive; Pancreatic agenesis 2, 615935 (3), Autosomal recessive
PTGDR	604687	{Asthma, susceptibility to, 1}, 607277 (3)
PTGER2	176804	{Asthma, aspirin-induced, susceptibility to}, 208550 (3), Autosomal recessive
PTGIS	601699	Hypertension, essential, 145500 (3), Multifactorial
PTH	168450	Hypoparathyroidism, familial isolated 1, 146200 (3), Autosomal dominant, Autosomal recessive
PTH1R	168468	Metaphyseal chondrodysplasia, Murk Jansen type, 156400 (3), Autosomal dominant; Eiken syndrome, 600002 (3), Autosomal recessive; Failure of tooth eruption, primary, 125350 (3), Autosomal dominant; Chondrodysplasia, Blomstrand type, 215045 (3), Autosomal recessive
PTHLH	168470	Brachydactyly, type E2, 613382 (3), Autosomal dominant
PTPN1	176885	{Insulin resistance, susceptibility to}, 125853 (3), Autosomal dominant

PTPN11	176876	Noonan syndrome 1, 163950 (3), Autosomal dominant; LEOPARD syndrome 1, 151100 (3), Autosomal dominant; Metachondromatosis, 156250 (3), Autosomal dominant; Leukemia, juvenile myelomonocytic, somatic, 607785 (3)
PTPN12	600079	Colon cancer, somatic, 114500 (3)
PTPN14	603155	Choanal atresia and lymphedema, 613611 (3), Autosomal recessive
PTPN22	600716	{Rheumatoid arthritis, susceptibility to}, 180300 (3); {Systemic lupus erythematosus susceptibility to}, 152700 (3), Autosomal dominant; {Diabetes, type 1, susceptibility to}, 222100 (3), Autosomal recessive
PTPN23	606584	Neurodevelopmental disorder and structural brain anomalies with or without seizures and spasticity, 618890 (3), Autosomal recessive
PTPRC	151460	Immunodeficiency 105, severe combined, 619924 (3), Autosomal recessive
PTPRF	179590	?Breasts and/or nipples, aplasia or hypoplasia of, 2, 616001 (3), Autosomal recessive
PTPRJ	600925	Colon cancer, somatic, 114500 (3)
PTPRO	600579	Nephrotic syndrome, type 6, 614196 (3), Autosomal recessive
PTPRQ	603317	Deafness, autosomal dominant 73, 617663 (3), Autosomal dominant; Deafness, autosomal recessive 84A, 613391 (3), Autosomal recessive
PTRH2	608625	Infantile-onset multisystem neurologic, endocrine, and pancreatic disease, 616263 (3), Autosomal recessive
PTS	612719	Hyperphenylalaninemia, BH4-deficient, A, 261640 (3), Autosomal recessive
PUF60	604819	Verheij syndrome, 615583 (3), Autosomal dominant
PUM1	607204	Spinocerebellar ataxia 47, 617931 (3), Autosomal dominant
PURA	600473	Neurodevelopmental disorder with neonatal respiratory insufficiency, hypotonia, and feeding difficulties, 616158 (3), Autosomal dominant
PUS1	608109	Myopathy, lactic acidosis, and sideroblastic anemia 1, 600462 (3), Autosomal recessive
PUS3	616283	Neurodevelopmental disorder with microcephaly and gray sclerae, 617051 (3), Autosomal recessive
PUS7	616261	Intellectual developmental disorder with abnormal behavior, microcephaly, and short stature, 618342 (3), Autosomal recessive
PXDN	605158	Anterior segment dysgenesis 7, with sclerocornea, 269400 (3), Autosomal recessive
PYCR1	179035	Cutis laxa, autosomal recessive, type IIIB, 614438 (3), Autosomal recessive; Cutis laxa, autosomal recessive, type IIB, 612940 (3), Autosomal recessive
PYCR2	616406	Leukodystrophy, hypomyelinating, 10, 616420 (3), Autosomal recessive
PYGL	613741	Glycogen storage disease VI, 232700 (3), Autosomal recessive
PYGM	608455	McArdle disease, 232600 (3), Autosomal recessive
PYROXD1	617220	Myopathy, myofibrillar, 8, 617258 (3), Autosomal recessive
QARS1	603727	Microcephaly, progressive, seizures, and cerebral and cerebellar atrophy, 615760 (3), Autosomal recessive
QDPR	612676	Hyperphenylalaninemia, BH4-deficient, C, 261630 (3), Autosomal recessive
QRICH1	617387	Ververi-Brady syndrome, 617982 (3), Autosomal dominant
QRICH2	618304	Spermatogenic failure 35, 618341 (3), Autosomal recessive

QRSL1	617209	Combined oxidative phosphorylation deficiency 40, 618835 (3), Autosomal recessive
RAB11B	604198	Neurodevelopmental disorder with ataxic gait, absent speech, and decreased cortical white matter, 617807 (3), Autosomal dominant
RAB18	602207	Warburg micro syndrome 3, 614222 (3), Autosomal recessive
RAB23	606144	Carpenter syndrome, 201000 (3), Autosomal recessive
RAB27A	603868	Griscelli syndrome, type 2, 607624 (3), Autosomal recessive
RAB28	612994	Cone-rod dystrophy 18, 615374 (3), Autosomal recessive
RAB33B	605950	Smith-McCort dysplasia 2, 615222 (3), Autosomal recessive
RAB39B	300774	Intellectual developmental disorder, X-linked 72, 300271 (3), X-linked recessive; Waisman syndrome, 311510 (3), X-linked recessive
RAB3GAP1	602536	Martsolf syndrome 2, 619420 (3), Autosomal recessive; Warburg micro syndrome 1, 600118 (3), Autosomal recessive
RAB3GAP2	609275	Martsolf syndrome 1, 212720 (3), Autosomal recessive; Warburg micro syndrome 2, 614225 (3), Autosomal recessive
RAB5IF	619960	?Craniofacial dysmorphism, skeletal anomalies, and impaired intellectual development syndrome 2, 616994 (3), Autosomal recessive
RAB7A	602298	Charcot-Marie-Tooth disease, type 2B, 600882 (3), Autosomal dominant
RABL3	618542	{?Pancreatic cancer, susceptibility to, 5}, 618680 (3), Autosomal dominant
RAC1	602048	Intellectual developmental disorder, autosomal dominant 48, 617751 (3), Autosomal dominant
RAC2	602049	Immunodeficiency 73A with defective neutrophil chemotaxix and leukocytosis, 608203 (3), Autosomal dominant; ?Immunodeficiency 73C with defective neutrophil chemotaxis and hypogammaglobulinemia, 618987 (3), Autosomal recessive; Immunodeficiency 73B with defective neutrophil chemotaxis and lymphopenia, 618986 (3), Autosomal dominant
RAC3	602050	Neurodevelopmental disorder with structural brain anomalies and dysmorphic facies, 618577 (3), Autosomal dominant
RACGAP1	604980	?Anemia, congenital dyserythropoietic, type IIIb, autosomal recessive, 619789 (3), Autosomal recessive
RAD21	606462	Cornelia de Lange syndrome 4, 614701 (3), Autosomal dominant; ?Mungan syndrome, 611376 (3), Autosomal recessive
RAD50	604040	Nijmegen breakage syndrome-like disorder, 613078 (3), Autosomal recessive
RAD51	179617	Mirror movements 2, 614508 (3), Autosomal dominant; {Breast cancer, susceptibility to}, 114480 (3), Autosomal dominant, Somatic mutation; Fanconi anemia, complementation group R, 617244 (3), Autosomal dominant
RAD51C	602774	{Breast-ovarian cancer, familial, susceptibility to, 3}, 613399 (3); Fanconi anemia, complementation group O, 613390 (3), Autosomal recessive
RAD51D	602954	{Breast-ovarian cancer, familial, susceptibility to, 4}, 614291 (3)
RAD54B	604289	Colon cancer, somatic, 114500 (3); Lymphoma, non-Hodgkin, somatic, 605027 (3)

RAD54L	603615	{Breast cancer, invasive ductal}, 114480 (3), Autosomal dominant, Somatic mutation; Adenocarcinoma, colonic, somatic (3); Lymphoma, non-Hodgkin, somatic, 605027 (3)
RAF1	164760	Cardiomyopathy, dilated, 1NN, 615916 (3), Autosomal dominant; Noonan syndrome 5, 611553 (3), Autosomal dominant; LEOPARD syndrome 2, 611554 (3)
RAG1	179615	Omenn syndrome, 603554 (3), Autosomal recessive; Severe combined immunodeficiency, B cell-negative, 601457 (3), Autosomal recessive; Combined cellular and humoral immune defects with granulomas, 233650 (3), Autosomal recessive; Alpha/beta T-cell lymphopenia with gamma/delta T-cell expansion, severe cytomegalovirus infection, and autoimmunity, 609889 (3)
RAG2	179616	Severe combined immunodeficiency, B cell-negative, 601457 (3), Autosomal recessive; Combined cellular and humoral immune defects with granulomas, 233650 (3), Autosomal recessive; Omenn syndrome, 603554 (3), Autosomal recessive
RAI1	607642	Smith-Magenis syndrome, 182290 (3), Autosomal dominant, Isolated cases
RALA	179550	Hiatt-Neu-Cooper neurodevelopmental syndrome, 619311 (3), Autosomal dominant
RALGAPA1	608884	Neurodevelopmental disorder with hypotonia, neonatal respiratory insufficiency, and thermodysregulation, 618797 (3), Autosomal recessive
RANBP2	601181	{Encephalopathy, acute, infection-induced, 3, susceptibility to}, 608033 (3), Autosomal dominant
RAP1GDS1	179502	Lymphocytic leukemia, acute T-cell (3)
RAPGEF2	609530	?Epilepsy, familial adult myoclonic, 7, 618075 (3), Autosomal dominant
RAPSN	601592	Fetal akinesia deformation sequence 2, 618388 (3), Autosomal recessive; Myasthenic syndrome, congenital, 11, associated with acetylcholine receptor deficiency, 616326 (3), Autosomal recessive
RARA	180240	Leukemia, acute promyelocytic, 612376 (1)
RARB	180220	Microphthalmia, syndromic 12, 615524 (3), Autosomal dominant, Autosomal recessive
RARS1	107820	Leukodystrophy, hypomyelinating, 9, 616140 (3), Autosomal recessive
RARS2	611524	Pontocerebellar hypoplasia, type 6, 611523 (3), Autosomal recessive
RASA1	139150	Capillary malformation-arteriovenous malformation 1, 608354 (3), Autosomal dominant; Basal cell carcinoma, somatic, 605462 (3)
RASGRP1	603962	Immunodeficiency 64, 618534 (3), Autosomal recessive
RASGRP2	605577	?Bleeding disorder, platelet-type, 18, 615888 (3), Autosomal recessive
RAX	601881	Microphthalmia, isolated 3, 611038 (3), Autosomal recessive
RAX2	610362	Cone-rod dystrophy 11, 610381 (3), Autosomal dominant; ?Macular degeneration, age-related, 6, 613757 (3)
RB1	614041	Small cell cancer of the lung, somatic, 182280 (3); Bladder cancer, somatic, 109800 (3); Retinoblastoma, trilateral, 180200 (3), Autosomal dominant, Somatic mutation; Osteosarcoma, somatic, 259500 (3); Retinoblastoma, 180200 (3), Autosomal dominant, Somatic mutation

RB1CC1	606837	Breast cancer, somatic, 114480 (3)
RBBP8	604124	Seckel syndrome 2, 606744 (3), Autosomal recessive; Jawad syndrome, 251255 (3), Autosomal recessive; Pancreatic carcinoma, somatic (3)
RBCK1	610924	Polyglucosan body myopathy 1 with or without immunodeficiency, 615895 (3), Autosomal recessive
RBL2	180203	Brunet-Wagner neurodevelopmental syndrome, 619690 (3), Autosomal recessive
RBM10	300080	TARP syndrome, 311900 (3), X-linked recessive
RBM12	607179	{Schizophrenia 19, susceptibility to}, 617629 (3), Autosomal dominant
RBM15	606077	Megakaryoblastic leukemia, acute, 606077 (2)
RBM20	613171	Cardiomyopathy, dilated, 1DD, 613172 (3), Autosomal dominant
RBM28	612074	?Alopecia, neurologic defects, and endocrinopathy syndrome, 612079 (3), Autosomal recessive
RBM8A	605313	Thrombocytopenia-absent radius syndrome, 274000 (3), Autosomal recessive
RBMX	300199	?Intellectual developmental disorder, X-linked syndromic 11, Shashi type, 300238 (3), X-linked recessive
RBP3	180290	?Retinitis pigmentosa 66, 615233 (3), Autosomal recessive
RBP4	180250	Microphthalmia, isolated, with coloboma 10, 616428 (3), Autosomal dominant; Retinal dystrophy, iris coloboma, and comedogenic acne syndrome, 615147 (3), Autosomal recessive
RBPJ	147183	Adams-Oliver syndrome 3, 614814 (3), Autosomal dominant
RC3H1	609424	?Immune dysregulation and systemic hyperinflammation syndrome, 618998 (3)
RCBTB1	607867	Retinal dystrophy with or without extraocular anomalies, 617175 (3), Autosomal recessive
RD3	180040	Leber congenital amaurosis 12, 610612 (3), Autosomal recessive
RDH11	607849	?Retinal dystrophy, juvenile cataracts, and short stature syndrome, 616108 (3), Autosomal recessive
RDH12	608830	Leber congenital amaurosis 13, 612712 (3), Autosomal dominant, Autosomal recessive
RDH5	601617	Fundus albipunctatus, 136880 (3), Autosomal dominant, Autosomal recessive
RDX	179410	Deafness, autosomal recessive 24, 611022 (3), Autosomal recessive
REC114	618421	Oocyte maturation defect 10, 619176 (3), Autosomal recessive
RECQL4	603780	Baller-Gerold syndrome, 218600 (3), Autosomal recessive; Rothmund-Thomson syndrome, type 2, 268400 (3), Autosomal recessive; RAPADILINO syndrome, 266280 (3), Autosomal recessive
REEP1	609139	?Neuronopathy, distal hereditary motor, type VB, 614751 (3), Autosomal dominant; Spastic paraplegia 31, autosomal dominant, 610250 (3), Autosomal dominant
REEP2	609347	?Spastic paraplegia 72, autosomal dominant, 615625 (3), Autosomal dominant, Autosomal recessive; ?Spastic paraplegia 72, autosomal recessive, 615625 (3), Autosomal dominant, Autosomal recessive

REEP6	609346	Retinitis pigmentosa 77, 617304 (3), Autosomal recessive
REL	164910	Immunodeficiency 92, 619652 (3), Autosomal recessive
RELA	164014	?Mucocutaneous ulceration, chronic, 618287 (3), Autosomal dominant
RELB	604758	?Immunodeficiency 53, 617585 (3), Autosomal recessive
RELN	600514	{Epilepsy, familial temporal lobe, 7}, 616436 (3), Autosomal dominant; Lissencephaly 2 (Norman-Roberts type), 257320 (3), Autosomal recessive
RELT	611211	Amelogenesis imperfecta, type IIIC, 618386 (3), Autosomal recessive
REN	179820	Renal tubular dysgenesis, 267430 (3), Autosomal recessive; [Hyperproreninemia] (3); Tubulointerstitial kidney disease, autosomal dominant, 4, 613092 (3), Autosomal dominant
REPS1	614825	?Neurodegeneration with brain iron accumulation 7, 617916 (3), Autosomal recessive
RERE	605226	Neurodevelopmental disorder with or without anomalies of the brain, eye, or heart, 616975 (3), Autosomal dominant
REST	600571	Deafness, autosomal dominant 27, 612431 (3), Autosomal dominant; {Wilms tumor 6, susceptibility to}, 616806 (3), Autosomal dominant; Fibromatosis, gingival, 5, 617626 (3), Autosomal dominant
RET	164761	{Hirschsprung disease, susceptibility to, 1}, 142623 (3), Autosomal dominant; Multiple endocrine neoplasia IIA, 171400 (3), Autosomal dominant; {Hirschsprung disease, protection against}, 142623 (3), Autosomal dominant; Medullary thyroid carcinoma, 155240 (3), Autosomal dominant; Pheochromocytoma, 171300 (3), Autosomal dominant; Multiple endocrine neoplasia IIB, 162300 (3), Autosomal dominant
RETN	605565	{Hypertension, insulin resistance-related, susceptibility to}, 125853 (3), Autosomal dominant; {Diabetes mellitus, noninsulin-dependent, susceptibility to}, 125853 (3), Autosomal dominant
RETREG1	613114	Neuropathy, hereditary sensory and autonomic, type IIB, 613115 (3), Autosomal recessive
RFC1	102579	Cerebellar ataxia, neuropathy, and vestibular areflexia syndrome, 614575 (3), Autosomal recessive
RFT1	611908	Congenital disorder of glycosylation, type In, 612015 (3), Autosomal recessive
RFWD3	614151	?Fanconi anemia, complementation group W, 617784 (3), Autosomal recessive
RFX5	601863	Bare lymphocyte syndrome, type II, complementation group C, 209920 (3), Autosomal recessive; Bare lymphocyte syndrome, type II, complementation group E, 209920 (3), Autosomal recessive
RFX6	612659	Mitchell-Riley syndrome, 615710 (3), Autosomal recessive
RFXANK	603200	MHC class II deficiency, complementation group B, 209920 (3), Autosomal recessive
RFXAP	601861	Bare lymphocyte syndrome, type II, complementation group D, 209920 (3), Autosomal recessive
RGR	600342	Retinitis pigmentosa 44, 613769 (3)
RGS5	603276	[Blood pressure regulation QTL], 145500 (2), Multifactorial

RGS9	604067	Bradyopsia, 608415 (3)
RGS9BP	607814	Bradyopsia, 608415 (3)
RHAG	180297	Overhydrated hereditary stomatocytosis, 185000 (3), Autosomal dominant; Anemia, hemolytic, Rh-null, regulator type, 268150 (3), Autosomal dominant
RHBDF2	614404	Tylosis with esophageal cancer, 148500 (3), Autosomal dominant
RHCE	111700	Rh-null disease, amorph type, 617970 (3)
RHD	111680	{Hemolytic disease of fetus and newborn, RH-induced}, 619462 (3); [Blood group, RH system], 111690 (3)
RHO	180380	Night blindness, congenital stationary, autosomal dominant 1, 610445 (3), Autosomal dominant; Retinitis pigmentosa 4, autosomal dominant or recessive, 613731 (3), Autosomal dominant, Autosomal recessive; Retinitis punctata albescens, 136880 (3), Autosomal dominant, Autosomal recessive
RHOA	165390	Ectodermal dysplasia with facial dysmorphism and acral, ocular, and brain anomalies, somatic mosaic, 618727 (3)
RHOBTB2	607352	Developmental and epileptic encephalopathy 64, 618004 (3), Autosomal dominant
RHOH	602037	{?Epidermodysplasia verruciformis, susceptibility to, 4}, 618307 (3), Autosomal recessive
RIC1	610354	CATIFA syndrome, 618761 (3), Autosomal recessive
RIGI (DDX58)	609631	Singleton-Merten syndrome 2, 616298 (3), Autosomal dominant
RILPL1	614092	Oculopharyngodistal myopathy 4, 619790 (3), Autosomal dominant
RIMS1	606629	Cone-rod dystrophy 7, 603649 (3), Autosomal dominant
RIMS2	606630	Cone-rod synaptic disorder syndrome, congenital nonprogressive, 618970 (3), Autosomal recessive
RIN2	610222	Macrocephaly, alopecia, cutis laxa, and scoliosis, 613075 (3), Autosomal recessive
RINT1	610089	Infantile liver failure syndrome 3, 618641 (3), Autosomal recessive
RIPK1	603453	Immunodeficiency 57 with autoinflammation, 618108 (3), Autosomal recessive; Autoinflammation with episodic fever and lymphadenopathy, 618852 (3), Autosomal dominant
RIPK4	605706	CHAND syndrome, 214350 (3), Autosomal recessive; Popliteal pterygium syndrome, Bartsocas-Papas type 1, 263650 (3), Autosomal recessive
RIPOR2	611410	Deafness, autosomal dominant 21, 607017 (3), Autosomal dominant; ?Deafness, autosomal recessive 104, 616515 (3), Autosomal recessive
RIPPLY2	609891	?Spondylocostal dysostosis 6, 616566 (3), Autosomal recessive
RIT1	609591	Noonan syndrome 8, 615355 (3), Autosomal dominant
RLBP1	180090	Bothnia retinal dystrophy, 607475 (3), Autosomal recessive; Newfoundland rod-cone dystrophy, 607476 (3); Retinitis punctata albescens, 136880 (3), Autosomal dominant, Autosomal recessive; Fundus albipunctatus, 136880 (3), Autosomal dominant, Autosomal recessive
RLIM	300379	Tonne-Kalscheuer syndrome, 300978 (3), X-linked

RMND1	614917	Combined oxidative phosphorylation deficiency 11, 614922 (3), Autosomal recessive
RMRP	157660	Anauxetic dysplasia 1, 607095 (3), Autosomal recessive; Metaphyseal dysplasia without hypotrichosis, 250460 (3), Autosomal recessive; Cartilage-hair hypoplasia, 250250 (3), Autosomal recessive
RNASEH1	604123	Progressive external ophthalmoplegia with mitochondrial DNA deletions, autosomal recessive 2, 616479 (3), Autosomal recessive
RNASEH2A	606034	Aicardi-Goutieres syndrome 4, 610333 (3), Autosomal recessive
RNASEH2B	610326	Aicardi-Goutieres syndrome 2, 610181 (3), Autosomal recessive
RNASEH2C	610330	Aicardi-Goutieres syndrome 3, 610329 (3), Autosomal recessive
RNASEL	180435	Prostate cancer 1, 601518 (3), Autosomal dominant
RNASET2	612944	Leukoencephalopathy, cystic, without megalencephaly, 612951 (3), Autosomal recessive
RNF113A	300951	Trichothiodystrophy 5, nonphotosensitive, 300953 (3), X-linked
RNF125	610432	Tenorio syndrome, 616260 (3), Autosomal dominant
RNF13	609247	Developmental and epileptic encephalopathy 73, 618379 (3), Autosomal dominant
RNF139	603046	Renal cell carcinoma, 144700 (3)
RNF168	612688	RIDDLE syndrome, 611943 (3), Autosomal recessive
RNF170	614649	Ataxia, sensory, 1, autosomal dominant, 608984 (3), Autosomal dominant; Spastic paraplegia 85, autosomal recessive, 619686 (3), Autosomal recessive
RNF2	608985	Luo-Schoch-Yamamoto syndrome, 619460 (3), Autosomal dominant
RNF212	612041	?Spermatogenic failure 62, 619673 (3), Autosomal recessive; Recombination rate QTL 1, 612042 (3)
RNF213	613768	{Moyamoya disease 2, susceptibility to}, 607151 (3), Autosomal dominant, Autosomal recessive
RNF216	609948	Cerebellar ataxia and hypogonadotropic hypogonadism, 212840 (3), Autosomal recessive
RNF220	616136	Leukodystrophy, hypomyelinating, 23, with ataxia, deafness, liver dysfunction, and dilated cardiomyopathy, 619688 (3), Autosomal recessive
RNF43	612482	Sessile serrated polyposis cancer syndrome, 617108 (3), Autosomal dominant
RNF6	604242	Esophageal carcinoma, somatic, 133239 (3)
RNPC3	618016	Pituitary hormone deficiency, combined or isolated, 7, 618160 (3), Autosomal recessive
RNU4ATAC	601428	Roifman syndrome, 616651 (3), Autosomal recessive; Lowry-Wood syndrome, 226960 (3), Autosomal recessive; Microcephalic osteodysplastic primordial dwarfism, type I, 210710 (3), Autosomal recessive
RNU7-1	617876	Aicardi-Goutieres syndrome 9, 619487 (3), Autosomal recessive
ROBO1	602430	Tetralogy of Fallot and septal defects.Â PMID: 30692597, 31448886, 30530901
ROBO2	602431	Vesicoureteral reflux 2, 610878 (3), Autosomal dominant

ROBO3	608630	Gaze palsy, familial horizontal, with progressive scoliosis, 1, 607313 (3), Autosomal recessive
ROBO4	607528	Aortic valve disease 3, 618496 (3), Autosomal dominant
ROGDI	614574	Kohlschutter-Tonz syndrome, 226750 (3), Autosomal recessive
ROM1	180721	Retinitis pigmentosa 7, digenic form, 608133 (3), Digenic dominant, Autosomal dominant, Autosomal recessive
ROR1	602336	?Deafness, autosomal recessive 108, 617654 (3), Autosomal recessive
ROR2	602337	Brachydactyly, type B1, 113000 (3), Autosomal dominant; Robinow syndrome, autosomal recessive, 268310 (3), Autosomal recessive
RORA	600825	Intellectual developmental disorder with or without epilepsy or cerebellar ataxia, 618060 (3), Autosomal dominant
RORB	601972	{Epilepsy, idiopathic generalized, susceptibility to, 15}, 618357 (3), Autosomal dominant
RORC	602943	Immunodeficiency 42, 616622 (3), Autosomal recessive
RP1	603937	Retinitis pigmentosa 1, 180100 (3), Autosomal dominant, Autosomal recessive
RP1L1	608581	Occult macular dystrophy, 613587 (3), Autosomal dominant; Retinitis pigmentosa 88, 618826 (3), Autosomal recessive
RP2	300757	Retinitis pigmentosa 2, 312600 (3), X-linked
RP9	607331	?Retinitis pigmentosa 9, 180104 (3), Autosomal dominant
RPA1	179835	Pulmonary fibrosis and/or bone marrow failure, telomere-related, 6, 619767 (3), Autosomal dominant
RPE65	180069	Retinitis pigmentosa 20, 613794 (3), Autosomal recessive; Retinitis pigmentosa 87 with choroidal involvement, 618697 (3), Autosomal dominant; Leber congenital amaurosis 2, 204100 (3), Autosomal recessive
RPGR	312610	Retinitis pigmentosa, X-linked, and sinorespiratory infections, with or without deafness, 300455 (3); Cone-rod dystrophy, X-linked, 1, 304020 (3), X-linked recessive; Retinitis pigmentosa 3, 300029 (3); Macular degeneration, X-linked atrophic, 300834 (3), X-linked recessive
RPGRIP1	605446	Cone-rod dystrophy 13, 608194 (3), Autosomal recessive; Leber congenital amaurosis 6, 613826 (3), Autosomal recessive
RPGRIP1L	610937	Joubert syndrome 7, 611560 (3), Autosomal recessive; Meckel syndrome 5, 611561 (3), Autosomal recessive; ?COACH syndrome 3, 619113 (3), Autosomal recessive
RPIA	180430	Ribose 5-phosphate isomerase deficiency, 608611 (3), Autosomal recessive
RPL10	312173	{Autism, susceptibility to, X-linked 5}, 300847 (3); Intellectual developmental disorder, X-linked syndromic 35, 300998 (3), X-linked recessive
RPL10L	619655	?Spermatogenic failure 63, 619689 (3), Autosomal recessive
RPL11	604175	Diamond-Blackfan anemia 7, 612562 (3), Autosomal dominant
RPL13	113703	Spondyloepimetaphyseal dysplasia, Isidor-Toutain type, 618728 (3), Autosomal dominant
RPL15	604174	?Diamond-Blackfan anemia 12, 615550 (3), Autosomal dominant
RPL18	604179	?Diamond-Blackfan anemia 18, 618310 (3), Autosomal dominant

RPL21	603636	Hypotrichosis 12, 615885 (3), Autosomal dominant
RPL26	603704	?Diamond-Blackfan anemia 11, 614900 (3), Autosomal dominant
RPL27	607526	?Diamond-Blackfan anemia 16, 617408 (3), Autosomal dominant
RPL35	618315	?Diamond-Blackfan anemia 19, 618312 (3), Autosomal dominant
RPL35A	180468	Diamond-Blackfan anemia 5, 612528 (3), Autosomal dominant
RPL3L	617416	Cardiomyopathy, dilated, 2D, 619371 (3), Autosomal recessive
RPL5	603634	Diamond-Blackfan anemia 6, 612561 (3), Autosomal dominant
RPS10	603632	Diamond-Blackfan anemia 9, 613308 (3), Autosomal dominant
RPS14	130620	Macrocytic anemia, refractory, due to 5q deletion, somatic, 153550 (3)
RPS15A	603674	?Diamond-Blackfan anemia 20, 618313 (3), Autosomal dominant
RPS17	180472	Diamond-Blackfan anemia 4, 612527 (3), Autosomal dominant
RPS19	603474	Diamond-Blackfan anemia 1, 105650 (3), Autosomal dominant
RPS23	603683	Brachycephaly, trichomegaly, and developmental delay, 617412 (3), Autosomal dominant
RPS24	602412	Diamond-blackfan anemia 3, 610629 (3), Autosomal dominant
RPS26	603701	Diamond-Blackfan anemia 10, 613309 (3), Autosomal dominant
RPS27	603702	?Diamond-Blackfan anemia 17, 617409 (3), Autosomal dominant
RPS28	603685	Diamond Blackfan anemia 15 with mandibulofacial dysostosis, 606164 (3), Autosomal dominant
RPS29	603633	Diamond-Blackfan anemia 13, 615909 (3), Autosomal dominant
RPS6KA3	300075	Intellectual developmental disorder, X-linked 19, 300844 (3), X-linked dominant; Coffin-Lowry syndrome, 303600 (3), X-linked dominant
RPS7	603658	Diamond-Blackfan anemia 8, 612563 (3), Autosomal dominant
RPSA	150370	Asplenia, isolated congenital, 271400 (3), Autosomal dominant
RRAS2	600098	Ovarian carcinoma (3); Noonan syndrome 12, 618624 (3), Autosomal dominant
RRM2B	604712	Mitochondrial DNA depletion syndrome 8B (MNGIE type), 612075 (3), Autosomal recessive; Mitochondrial DNA depletion syndrome 8A (encephalomyopathic type with renal tubulopathy), 612075 (3), Autosomal recessive; Rod-cone dystrophy, sensorineural deafness, and Fanconi-type renal dysfunction, 268315 (3), Autosomal recessive; Progressive external ophthalmoplegia with mitochondrial DNA deletions, autosomal dominant 5, 613077 (3), Autosomal dominant
RRP7A	619449	?Microcephaly 28, primary, autosomal recessive, 619453 (3), Autosomal recessive
RS1	300839	Retinoschisis, 312700 (3), X-linked recessive
RSPH1	609314	Ciliary dyskinesia, primary, 24, 615481 (3), Autosomal recessive
RSPH3	615876	Ciliary dyskinesia, primary, 32, 616481 (3), Autosomal recessive
RSPH4A	612647	Ciliary dyskinesia, primary, 11, 612649 (3)
RSPH9	612648	Ciliary dyskinesia, primary, 12, 612650 (3)
RSPO1	609595	Palmoplantar hyperkeratosis and true hermaphroditism, 610644 (3), Autosomal recessive; Palmoplantar hyperkeratosis with squamous cell carcinoma of skin and sex reversal, 610644 (3), Autosomal recessive

RSPO2	610575	?Humerofemoral hypoplasia with radiotibial ray deficiency, 618022 (3), Autosomal recessive; Tetraamelia syndrome 2, 618021 (3), Autosomal recessive
RSPO4	610573	Anonychia congenita, 206800 (3), Autosomal recessive
RSPRY1	616585	Spondyloepimetaphyseal dysplasia, Faden-Alkuraya type, 616723 (3), Autosomal recessive
RSRC1	613352	Intellectual developmental disorder, autosomal recessive 70, 618402 (3), Autosomal recessive
RTEL1	608833	Pulmonary fibrosis and/or bone marrow failure, telomere-related, 3, 616373 (3), Autosomal dominant; Dyskeratosis congenita, autosomal dominant 4, 615190 (3), Autosomal dominant, Autosomal recessive; Dyskeratosis congenita, autosomal recessive 5, 615190 (3), Autosomal dominant, Autosomal recessive
RTN2	603183	Spastic paraplegia 12, autosomal dominant, 604805 (3), Autosomal dominant
RTN4IP1	610502	Optic atrophy 10 with or without ataxia, mental retardation, and seizures, 616732 (3), Autosomal recessive
RTN4R	605566	{Schizophrenia, susceptibility to}, 181500 (3), Autosomal dominant
RTTN	610436	Microcephaly, short stature, and polymicrogyria with seizures, 614833 (3), Autosomal recessive
RUBCN	613516	Spinocerebellar ataxia, autosomal recessive 15, 615705 (3), Autosomal recessive
RUNX1	151385	Platelet disorder, familial, with associated myeloid malignancy, 601399 (3), Autosomal dominant; Leukemia, acute myeloid, 601626 (3), Autosomal dominant, Somatic mutation
RUNX2	600211	Metaphyseal dysplasia with maxillary hypoplasia with or without brachydactyly, 156510 (3), Autosomal dominant; Cleidocranial dysplasia, forme fruste, with brachydactyly, 119600 (3), Autosomal dominant; Cleidocranial dysplasia, forme fruste, dental anomalies only, 119600 (3), Autosomal dominant; Cleidocranial dysplasia, 119600 (3), Autosomal dominant
RUSC2	611053	Intellectual developmental disorder, autosomal recessive 61, 617773 (3), Autosomal recessive
RXYLT1	605862	Muscular dystrophy-dystroglycanopathy (congenital with brain and eye anomalies), type A, 10, 615041 (3), Autosomal recessive
RYR1	180901	Neuromuscular disease, congenital, with uniform type 1 fiber, 117000 (3), Autosomal dominant, Autosomal recessive; Central core disease, 117000 (3), Autosomal dominant, Autosomal recessive; King-Denborough syndrome, 619542 (3), Autosomal dominant; {Malignant hyperthermia susceptibility 1}, 145600 (3), Autosomal dominant; Minicore myopathy with external ophthalmoplegia, 255320 (3), Autosomal recessive

RYR2	180902	Ventricular tachycardia, catecholaminergic polymorphic, 1, 604772 (3), Autosomal dominant; Ventricular arrhythmias due to cardiac ryanodine receptor calcium release deficiency syndrome, 115000 (3), Autosomal dominant; Arrhythmogenic right ventricular dysplasia 2, 600996 (3), Autosomal dominant
S1PR2	605111	Deafness, autosomal recessive 68, 610419 (3), Autosomal recessive
SACS	604490	Spastic ataxia, Charlevoix-Saguenay type, 270550 (3), Autosomal recessive
SAG	181031	Retinitis pigmentosa 47, 613758 (3); Oguchi disease-1, 258100 (3), Autosomal recessive
SALL1	602218	Townes-Brocks syndrome 1, 107480 (3), Autosomal dominant; Townes-Brocks branchiootorenal-like syndrome, 107480 (3), Autosomal dominant
SALL2	602219	?Coloboma, ocular, autosomal recessive, 216820 (3), Autosomal recessive
SALL4	607343	?IVIC syndrome, 147750 (3), Autosomal dominant; Duane-radial ray syndrome, 607323 (3), Autosomal dominant
SAMD12	618073	Epilepsy, familial adult myoclonic, 1, 601068 (3), Autosomal dominant
SAMD9	610456	Tumoral calcinosis, familial, normophosphatemic, 610455 (3), Autosomal recessive; Monosomy 7 myelodysplasia and leukemia syndrome 2, 619041 (3), Autosomal dominant; MIRAGE syndrome, 617053 (3), Autosomal dominant
SAMD9L	611170	Ataxia-pancytopenia syndrome, 159550 (3), Autosomal dominant; Monosomy 7 myelodysplasia and leukemia syndrome 1, 252270 (3), Autosomal dominant; Spinocerebellar ataxia 49, 619806 (3), Autosomal dominant
SAMHD1	606754	?Chilblain lupus 2, 614415 (3), Autosomal dominant; Aicardi-Goutieres syndrome 5, 612952 (3), Autosomal recessive
SAR1B	607690	Chylomicron retention disease, 246700 (3), Autosomal recessive
SARDH	604455	[Sarcosinemia], 268900 (3), Autosomal recessive
SARS1	607529	Neurodevelopmental disorder with microcephaly, ataxia, and seizures, 617709 (3), Autosomal recessive
SARS2	612804	Hyperuricemia, pulmonary hypertension, renal failure, and alkalosis, 613845 (3), Autosomal recessive
SASH1	607955	Dyschromatosis universalis hereditaria 1, 127500 (3), Autosomal dominant; ?Cancer, alopecia, pigment dyscrasia, onychodystrophy, and keratoderma, 618373 (3), Autosomal recessive
SASH3	300441	Immunodeficiency 102, 301082 (3), X-linked recessive
SASS6	609321	Microcephaly 14, primary, autosomal recessive, 616402 (3), Autosomal recessive
SATB1	602075	Kohlschutter-Tonz syndrome-like, 619229 (3), Autosomal dominant; Developmental delay with dysmorphic facies and dental anomalies, 619228 (3), Autosomal dominant
SATB2	608148	Glass syndrome, 612313 (3), Autosomal dominant
SBDS	607444	{Aplastic anemia, susceptibility to}, 609135 (3); Shwachman-Diamond syndrome 1, 260400 (3), Autosomal recessive
SBF1	603560	Charcot-Marie-Tooth disease, type 4B3, 615284 (3), Autosomal recessive

SBF2	607697	Charcot-Marie-Tooth disease, type 4B2, 604563 (3), Autosomal recessive
SC5D	602286	Lathosterolosis, 607330 (3), Autosomal recessive
SCAPER	611611	Intellectual developmental disorder and retinitis pigmentosa, 618195 (3), Autosomal recessive
SCARB1	601040	[High density lipoprotein cholesterol level QTL6], 610762 (3)
SCARB2	602257	Epilepsy, progressive myoclonic 4, with or without renal failure, 254900 (3), Autosomal recessive
SCARF2	613619	Van den Ende-Gupta syndrome, 600920 (3), Autosomal recessive
SCD5	608370	?Deafness, autosomal dominant 79, 619086 (3), Autosomal dominant
SCGB3A2	606531	{Asthma, susceptibility to}, 600807 (3), Autosomal dominant
SCN10A	604427	Episodic pain syndrome, familial, 2, 615551 (3), Autosomal dominant
SCN11A	604385	Episodic pain syndrome, familial, 3, 615552 (3), Autosomal dominant; Neuropathy, hereditary sensory and autonomic, type VII, 615548 (3), Autosomal dominant
SCN1A	182389	Developmental and epileptic encephalopathy 6B, non-Dravet, 619317 (3), Autosomal dominant; Migraine, familial hemiplegic, 3, 609634 (3), Autosomal dominant; Dravet syndrome, 607208 (3), Autosomal dominant; Febrile seizures, familial, 3A, 604403 (3), Autosomal dominant; Generalized epilepsy with febrile seizures plus, type 2, 604403 (3), Autosomal dominant
SCN1B	600235	Generalized epilepsy with febrile seizures plus, type 1, 604233 (3), Autosomal dominant; Developmental and epileptic encephalopathy 52, 617350 (3), Autosomal recessive; Cardiac conduction defect, nonspecific, 612838 (3); Atrial fibrillation, familial, 13, 615377 (3), Autosomal dominant; Brugada syndrome 5, 612838 (3)
SCN2A	182390	Seizures, benign familial infantile, 3, 607745 (3), Autosomal dominant; Developmental and epileptic encephalopathy 11, 613721 (3), Autosomal dominant; Episodic ataxia, type 9, 618924 (3), Autosomal dominant
SCN2B	601327	Atrial fibrillation, familial, 14, 615378 (3), Autosomal dominant
SCN3A	182391	Epilepsy, familial focal, with variable foci 4, 617935 (3), Autosomal dominant; Developmental and epileptic encephalopathy 62, 617938 (3), Autosomal dominant
SCN3B	608214	Atrial fibrillation, familial, 16, 613120 (3), Autosomal dominant; Brugada syndrome 7, 613120 (3), Autosomal dominant
SCN4A	603967	Paramyotonia congenita, 168300 (3), Autosomal dominant; Hypokalemic periodic paralysis, type 2, 613345 (3), Autosomal dominant; Myotonia congenita, atypical, acetazolamide-responsive, 608390 (3), Autosomal dominant; Myasthenic syndrome, congenital, 16, 614198 (3), Autosomal recessive; Hyperkalemic periodic paralysis, type 2, 170500 (3), Autosomal dominant
SCN4B	608256	Atrial fibrillation, familial, 17, 611819 (3), Autosomal dominant; Long QT syndrome 10, 611819 (3), Autosomal dominant

SCN5A	600163	Ventricular fibrillation, familial, 1, 603829 (3); Heart block, progressive, type IA, 113900 (3), Autosomal dominant; Cardiomyopathy, dilated, 1E, 601154 (3), Autosomal dominant; Heart block, nonprogressive, 113900 (3), Autosomal dominant; Long QT syndrome 3, 603830 (3), Autosomal dominant; Sick sinus syndrome 1, 608567 (3), Autosomal recessive; Brugada syndrome 1, 601144 (3), Autosomal dominant; Atrial fibrillation, familial, 10, 614022 (3), Autosomal dominant; {Sudden infant death syndrome, susceptibility to}, 272120 (3), Autosomal recessive
SCN8A	600702	?Myoclonus, familial, 2, 618364 (3), Autosomal dominant; Seizures, benign familial infantile, 5, 617080 (3), Autosomal dominant; Cognitive impairment with or without cerebellar ataxia, 614306 (3), Autosomal dominant; Developmental and epileptic encephalopathy 13, 614558 (3), Autosomal dominant
SCN9A	603415	Erythermalgia, primary, 133020 (3), Autosomal dominant; Insensitivity to pain, congenital, 243000 (3), Autosomal recessive; Small fiber neuropathy, 133020 (3), Autosomal dominant; Paroxysmal extreme pain disorder, 167400 (3), Autosomal dominant; Neuropathy, hereditary sensory and autonomic, type IID, 243000 (3), Autosomal recessive
SCNN1A	600228	Pseudohypoaldosteronism, type I, 264350 (3), Autosomal recessive; ?Liddle syndrome 3, 618126 (3), Autosomal dominant; Bronchiectasis with or without elevated sweat chloride 2, 613021 (3), Autosomal dominant
SCNN1B	600760	Bronchiectasis with or without elevated sweat chloride 1, 211400 (3), Autosomal dominant; Pseudohypoaldosteronism, type I, 264350 (3), Autosomal recessive; Liddle syndrome 1, 177200 (3), Autosomal dominant
SCNN1G	600761	Bronchiectasis with or without elevated sweat chloride 3, 613071 (3), Autosomal dominant; Pseudohypoaldosteronism, type I, 264350 (3), Autosomal recessive; Liddle syndrome 2, 618114 (3), Autosomal dominant
SCO1	603644	Mitochondrial complex IV deficiency, nuclear type 4, 619048 (3), Autosomal recessive
SCO2	604272	Myopia 6, 608908 (3), Autosomal dominant; Mitochondrial complex IV deficiency, nuclear type 2, 604377 (3), Autosomal recessive
SCP2	184755	?Leukoencephalopathy with dystonia and motor neuropathy, 613724 (3), Autosomal recessive
SCUBE3	614708	Short stature, facial dysmorphism, and skeletal anomalies with or without cardiac anomalies, 619184 (3), Autosomal recessive
SCYL1	607982	Spinocerebellar ataxia, autosomal recessive 21, 616719 (3), Autosomal recessive
SCYL2	616365	Arthrogryposis multiplex congenita 4, neurogenic, with agenesis of the corpus callosum, 618766 (3), Autosomal recessive
SDC3	186357	{Obesity, association with}, 601665 (3), Multifactorial, Autosomal dominant, Autosomal recessive
SDCCAG8	613524	Senior-Loken syndrome 7, 613615 (3), Autosomal recessive; Bardet-Biedl syndrome 16, 615993 (3), Autosomal recessive

SDHA	600857	Cardiomyopathy, dilated, 1GG, 613642 (3), Autosomal recessive; Mitochondrial complex II deficiency, nuclear type 1, 252011 (3), Autosomal recessive; Neurodegeneration with ataxia and late-onset optic atrophy, 619259 (3), Autosomal dominant; Paragangliomas 5, 614165 (3), Autosomal dominant
SDHAF1	612848	Mitochondrial complex II deficiency, nuclear type 2, 619166 (3), Autosomal recessive
SDHAF2	613019	Paragangliomas 2, 601650 (3), Autosomal dominant
SDHB	185470	Paragangliomas 4, 115310 (3), Autosomal dominant; Mitochondrial complex II deficiency, nuclear type 4, 619224 (3), Autosomal recessive; Gastrointestinal stromal tumor, 606764 (3), Autosomal dominant, Isolated cases; Pheochromocytoma, 171300 (3), Autosomal dominant; Paraganglioma and gastric stromal sarcoma, 606864 (3)
SDHC	602413	Paragangliomas 3, 605373 (3), Autosomal dominant; Paraganglioma and gastric stromal sarcoma, 606864 (3); Gastrointestinal stromal tumor, 606764 (3), Autosomal dominant, Isolated cases
SDHD	602690	Paragangliomas 1, with or without deafness, 168000 (3), Autosomal dominant; Paraganglioma and gastric stromal sarcoma, 606864 (3); Mitochondrial complex II deficiency, nuclear type 3, 619167 (3), Autosomal recessive; Pheochromocytoma, 171300 (3), Autosomal dominant
SDR9C7	609769	Ichthyosis, congenital, autosomal recessive 13, 617574 (3), Autosomal recessive
SEC23A	610511	Craniolenticulosutural dysplasia, 607812 (3), Autosomal dominant, Autosomal recessive
SEC23B	610512	?Cowden syndrome 7, 616858 (3), Autosomal dominant; Dyserythropoietic anemia, congenital, type II, 224100 (3), Autosomal recessive
SEC24D	607186	Cole-Carpenter syndrome 2, 616294 (3), Autosomal recessive
SEC31A	610257	?Halperin-Birk syndrome, 618651 (3), Autosomal recessive
SEC61A1	609213	Tubulointerstitial kidney disease, autosomal dominant, 5, 617056 (3), Autosomal dominant
SEC63	608648	Polycystic liver disease 2, 617004 (3), Autosomal dominant
SECISBP2	607693	Thyroid hormone metabolism, abnormal, 1, 609698 (3), Autosomal recessive; Thyroid hormone metabolism, abnormal, 609698 (3), Autosomal recessive
SELENBP1	604188	Extraoral halitosis due to MTO deficiency, 618148 (3), Autosomal recessive
SELENOI	607915	Spastic paraplegia 81, autosomal recessive, 618768 (3), Autosomal recessive
SELENON	606210	Myopathy, congenital, with fiber-type disproportion, 255310 (3), Autosomal dominant, Autosomal recessive; Muscular dystrophy, rigid spine, 1, 602771 (3), Autosomal recessive
SEMA3A	603961	{Hypogonadotropic hypogonadism 16 with or without anosmia}, 614897 (3), Autosomal dominant
SEMA3E	608166	?CHARGE syndrome, 214800 (3), Autosomal dominant

SEMA4A	607292	Retinitis pigmentosa 35, 610282 (3), Autosomal dominant, Autosomal recessive; Cone-rod dystrophy 10, 610283 (3), Autosomal recessive
SEMA6B	608873	Epilepsy, progressive myoclonic, 11, 618876 (3), Autosomal dominant
SEMA7A	607961	?Cholestasis, progressive familial intrahepatic, 11, 619874 (3), Autosomal recessive; [Blood group, John-Milton-Hagen system], 614745 (3)
SEPSECS	613009	Pontocerebellar hypoplasia type 2D, 613811 (3), Autosomal recessive
SEPTIN12	611562	Spermatogenic failure 10, 614822 (3), Autosomal dominant
SEPTIN9	604061	Amyotrophy, hereditary neuralgic, 162100 (3), Autosomal dominant
SERAC1	614725	3-methylglutaconic aciduria with deafness, encephalopathy, and Leigh-like syndrome, 614739 (3), Autosomal recessive
SERPINA1	107400	Hemorrhagic diathesis due to antithrombin Pittsburgh, 613490 (3), Autosomal recessive; Emphysema due to AAT deficiency, 613490 (3), Autosomal recessive; Emphysema-cirrhosis, due to AAT deficiency, 613490 (3), Autosomal recessive
SERPINA3	107280	Alpha-1-antichymotrypsin deficiency (3); Cerebrovascular disease, occlusive (3)
SERPINA6	122500	Corticosteroid-binding globulin deficiency, 611489 (3), Autosomal dominant, Autosomal recessive
SERPINA7	314200	[Thyroxine-binding globulin QTL], 300932 (3), X-linked
SERPINB6	173321	?Deafness, autosomal recessive 91, 613453 (3), Autosomal recessive
SERPINB7	603357	Palmoplantar keratoderma, Nagashima type, 615598 (3), Autosomal recessive
SERPINB8	601697	Peeling skin syndrome 5, 617115 (3), Autosomal recessive
SERPINC1	107300	Thrombophilia 7 due to antithrombin III deficiency, 613118 (3), Autosomal dominant, Autosomal recessive
SERPIND1	142360	Thrombophilia 10 due to heparin cofactor II deficiency, 612356 (3), Autosomal dominant
SERPINE1	173360	Plasminogen activator inhibitor-1 deficiency, 613329 (3), Autosomal dominant, Autosomal recessive; {Transcription of plasminogen activator inhibitor, modulator of} (3)
SERPINF1	172860	Osteogenesis imperfecta, type VI, 613982 (3), Autosomal recessive
SERPINF2	613168	Alpha-2-plasmin inhibitor deficiency, 262850 (3), Autosomal recessive
SERPING1	606860	Angioedema, hereditary, 1 and 2, 106100 (3), Autosomal dominant, Autosomal recessive; Complement component 4, partial deficiency of, 120790 (3), Autosomal dominant
SERPINH1	600943	{Preterm premature rupture of the membranes, susceptibility to}, 610504 (3); Osteogenesis imperfecta, type X, 613848 (3), Autosomal recessive
SERPINI1	602445	Encephalopathy, familial, with neuroserpin inclusion bodies, 604218 (3), Autosomal dominant
SET	600960	Intellectual developmental disorder, autosomal dominant 58, 618106 (3), Autosomal dominant
SETBP1	611060	Schinzel-Giedion midface retraction syndrome, 269150 (3), Autosomal dominant; Intellectual developmental disorder, autosomal dominant 29, 616078 (3), Autosomal dominant

SETD1A	611052	Epilepsy, early-onset, with or without developmental delay, 618832 (3), Autosomal dominant; Neurodevelopmental disorder with speech impairment and dysmorphic facies, 619056 (3), Autosomal dominant
SETD1B	611055	Intellectual developmental disorder with seizures and language delay, 619000 (3), Autosomal dominant
SETD2	612778	Luscan-Lumish syndrome, 616831 (3), Autosomal dominant
SETD5	615743	Intellectual developmental disorder, autosomal dominant 23, 615761 (3), Autosomal dominant
SETX	608465	Spinocerebellar ataxia, autosomal recessive, with axonal neuropathy 2, 606002 (3), Autosomal recessive; Amyotrophic lateral sclerosis 4, juvenile, 602433 (3), Autosomal dominant
SF3B1	605590	Myelodysplastic syndrome, somatic, 614286 (3)
SF3B2	605591	Craniofacial microsomia, 164210 (3), Autosomal dominant
SF3B4	605593	Acrofacial dysostosis 1, Nager type, 154400 (3), Autosomal dominant
SFRP4	606570	Pyle disease, 265900 (3), Autosomal recessive
SFTPA1	178630	Interstitial lung disease 1, 619611 (3), Autosomal dominant, Autosomal recessive
SFTPA2	178642	Interstitial lung disease 2, 178500 (3), Autosomal dominant
SFTPB	178640	Surfactant metabolism dysfunction, pulmonary, 1, 265120 (3), Autosomal recessive
SFTPC	178620	Surfactant metabolism dysfunction, pulmonary, 2, 610913 (3), Autosomal dominant
SFXN4	615564	Combined oxidative phosphorylation deficiency 18, 615578 (3), Autosomal recessive
SGCA	600119	Muscular dystrophy, limb-girdle, autosomal recessive 3, 608099 (3), Autosomal recessive
SGCB	600900	Muscular dystrophy, limb-girdle, autosomal recessive 4, 604286 (3), Autosomal recessive
SGCD	601411	Cardiomyopathy, dilated, 1L, 606685 (3); Muscular dystrophy, limb-girdle, autosomal recessive 6, 601287 (3), Autosomal recessive
SGCE	604149	Dystonia-11, myoclonic, 159900 (3), Autosomal dominant
SGCG	608896	Muscular dystrophy, limb-girdle, autosomal recessive 5, 253700 (3), Autosomal recessive
SGMS2	611574	Calvarial doughnut lesions with bone fragility with or without spondylometaphyseal dysplasia, 126550 (3), Autosomal dominant
SGO1	609168	Chronic atrial and intestinal dysrhythmia, 616201 (3), Autosomal recessive
SGPL1	603729	Nephrotic syndrome, type 14, 617575 (3), Autosomal recessive
SGSH	605270	Mucopolysaccharidosis type IIIA (Sanfilippo A), 252900 (3), Autosomal recessive
SH2B3	605093	Thrombocythemia, somatic, 187950 (3); Myelofibrosis, somatic, 254450 (3); Erythrocytosis, somatic, 133100 (3)
SH2D1A	300490	Lymphoproliferative syndrome, X-linked, 1, 308240 (3), X-linked recessive
SH3BP2	602104	Cherubism, 118400 (3), Autosomal dominant

SH3GL1	601768	Leukemia, acute myeloid, 601626 (1), Autosomal dominant, Somatic mutation
SH3KBP1	300374	?Immunodeficiency 61, 300310 (3), X-linked recessive
SH3PXD2B	613293	Frank-ter Haar syndrome, 249420 (3), Autosomal recessive
SH3TC2	608206	Charcot-Marie-Tooth disease, type 4C, 601596 (3), Autosomal recessive; Mononeuropathy of the median nerve, mild, 613353 (3), Autosomal dominant
SHANK1	604999	Spectrum of NDD. PMID: 34113010
SHANK2	603290	{Autism susceptibility 17}, 613436 (3)
SHANK3	606230	Phelan-McDermid syndrome, 606232 (3), Autosomal dominant; {Schizophrenia 15}, 613950 (3), Autosomal dominant
SHH	600725	Microphthalmia with coloboma 5, 611638 (3), Autosomal dominant; Schizencephaly, 269160 (3); Single median maxillary central incisor, 147250 (3), Autosomal dominant; Holoprosencephaly 3, 142945 (3), Autosomal dominant
SHMT2	138450	Neurodevelopmental disorder with cardiomyopathy, spasticity, and brain abnormalities, 619121 (3), Autosomal recessive
SHOC1	618038	Spermatogenic failure 75, 619949 (3), Autosomal recessive
SHOC2	602775	Noonan syndrome-like with loose anagen hair 1, 607721 (3), Autosomal dominant
SHOX	400020	Short stature, idiopathic familial, 300582 (3); Langer mesomelic dysplasia, 249700 (3), Pseudoautosomal recessive; Leri-Weill dyschondrosteosis, 127300 (3), Pseudoautosomal dominant
SHPK	605060	[Sedoheptulokinase deficiency], 617213 (3), Autosomal recessive
SHQ1	613663	Neurodevelopmental disorder with dystonia and seizures, 619922 (3), Autosomal recessive; ?Dystonia 35, childhood-onset, 619921 (3), Autosomal recessive
SHROOM4	300579	Intellectual developmental disorder, X-linked syndromic, Stocco dos Santos type, 300434 (3), X-linked
SI	609845	Sucrase-isomaltase deficiency, congenital, 222900 (3), Autosomal recessive
SIAE	610079	{Autoimmune disease, susceptibility to, 6}, 613551 (3)
SIAH1	602212	Buratti-Harel syndrome, 619314 (3), Autosomal dominant
SIGMAR1	601978	?Spinal muscular atrophy, distal, autosomal recessive, 2, 605726 (3), Autosomal recessive; ?Amyotrophic lateral sclerosis 16, juvenile, 614373 (3), Autosomal recessive
SIK1	605705	Developmental and epileptic encephalopathy 30, 616341 (3), Autosomal dominant
SIK3	614776	?Spondyloepimetaphyseal dysplasia, Krakow type, 618162 (3), Autosomal recessive
SIL1	608005	Marinesco-Sjogren syndrome, 248800 (3), Autosomal recessive
SIM1	603128	Early onset obesity and hypopituitarism. PMID: 33434169
SIN3A	607776	Witteveen-Kolk syndrome, 613406 (3), Autosomal dominant
SIPA1L3	616655	?Cataract 45, 616851 (3), Autosomal recessive

SIX1	601205	Deafness, autosomal dominant 23, 605192 (3), Autosomal dominant; Branchiootic syndrome 3, 608389 (3), Autosomal dominant
SIX3	603714	Schizencephaly, 269160 (3); Holoprosencephaly 2, 157170 (3), Autosomal dominant
SIX5	600963	Branchiootorenal syndrome 2, 610896 (3)
SIX6	606326	Optic disc anomalies with retinal and/or macular dystrophy, 212550 (3), Autosomal recessive
SKI	164780	Shprintzen-Goldberg syndrome, 182212 (3), Autosomal dominant
SKIC2 (SKIV2L)	600478	Trichohepatoenteric syndrome 2, 614602 (3), Autosomal recessive
SKIC3 (TTC37)	614589	Trichohepatoenteric syndrome 1, 222470 (3), Autosomal recessive
SLC10A1	182396	Hypercholanemia, familial 2, 619256 (3), Autosomal recessive
SLC10A2	601295	?Bile acid malabsorption, primary, 1, 613291 (3), Autosomal recessive
SLC10A7	611459	Short stature, amelogenesis imperfecta, and skeletal dysplasia with scoliosis, 618363 (3), Autosomal recessive
SLC11A1	600266	{Mycobacterium tuberculosis, susceptibility to infection by}, 607948 (3); {Buruli ulcer, susceptibility to}, 610446 (3)
SLC11A2	600523	Anemia, hypochromic microcytic, with iron overload 1, 206100 (3), Autosomal recessive
SLC12A1	600839	Bartter syndrome, type 1, 601678 (3), Autosomal recessive
SLC12A2	600840	Kilquist syndrome, 619080 (3), Autosomal recessive; Delpire-McNeill syndrome, 619083 (3), Autosomal dominant; Deafness, autosomal dominant 78, 619081 (3), Autosomal dominant
SLC12A3	600968	Gitelman syndrome, 263800 (3), Autosomal recessive
SLC12A5	606726	{Epilepsy, idiopathic generalized, susceptibility to, 14}, 616685 (3), Autosomal dominant; Developmental and epileptic encephalopathy 34, 616645 (3), Autosomal recessive
SLC12A6	604878	Agenesis of the corpus callosum with peripheral neuropathy, 218000 (3), Autosomal recessive
SLC13A3	606411	Leukoencephalopathy, acute reversible, with increased urinary alpha-ketoglutarate, 618384 (3), Autosomal recessive
SLC13A5	608305	Developmental and epileptic encephalopathy 25, with amelogenesis imperfecta, 615905 (3), Autosomal recessive
SLC14A1	613868	[Blood group, Kidd], 111000 (3)
SLC16A1	600682	Hyperinsulinemic hypoglycemia, familial, 7, 610021 (3), Autosomal dominant; Erythrocyte lactate transporter defect, 245340 (3), Autosomal dominant; Monocarboxylate transporter 1 deficiency, 616095 (3), Autosomal dominant, Autosomal recessive
SLC16A12	611910	Cataract 47, juvenile, with microcornea, 612018 (3), Autosomal dominant
SLC16A2	300095	Allan-Herndon-Dudley syndrome, 300523 (3), X-linked
SLC17A3	611034	[Uric acid concentration, serum, QTL4], 612671 (3), Autosomal dominant; {Gout susceptibility 4}, 612671 (3), Autosomal dominant

SLC17A5	604322	Salla disease, 604369 (3), Autosomal recessive; Sialic acid storage disorder, infantile, 269920 (3), Autosomal recessive
SLC17A8	607557	Deafness, autosomal dominant 25, 605583 (3), Autosomal dominant
SLC17A9	612107	Porokeratosis 8, disseminated superficial actinic type, 616063 (3), Autosomal dominant
SLC18A2	193001	?Parkinsonism-dystonia, infantile, 2, 618049 (3), Autosomal recessive
SLC18A3	600336	Myasthenic syndrome, congenital, 21, presynaptic, 617239 (3), Autosomal recessive
SLC19A1	600424	?Megaloblastic anemia, folate-responsive, 601775 (3), Autosomal recessive
SLC19A2	603941	Thiamine-responsive megaloblastic anemia syndrome, 249270 (3), Autosomal recessive
SLC19A3	606152	Thiamine metabolism dysfunction syndrome 2 (biotin- or thiamine-responsive encephalopathy type 2), 607483 (3), Autosomal recessive
SLC1A1	133550	Dicarboxylic aminoaciduria, 222730 (3), Autosomal recessive; {?Schizophrenia susceptibility 18}, 615232 (3)
SLC1A2	600300	Developmental and epileptic encephalopathy 41, 617105 (3), Autosomal dominant
SLC1A3	600111	Episodic ataxia, type 6, 612656 (3), Autosomal dominant
SLC1A4	600229	Spastic tetraplegia, thin corpus callosum, and progressive microcephaly, 616657 (3), Autosomal recessive
SLC20A2	158378	Basal ganglia calcification, idiopathic, 1, 213600 (3), Autosomal dominant
SLC22A12	607096	Hypouricemia, renal, 220150 (3), Autosomal recessive
SLC22A18	602631	Breast cancer, somatic, 114480 (3); Lung cancer, somatic, 211980 (3); Rhabdomyosarcoma, somatic, 268210 (3)
SLC22A4	604190	{Rheumatoid arthritis, susceptibility to}, 180300 (3)
SLC22A5	603377	Carnitine deficiency, systemic primary, 212140 (3), Autosomal recessive
SLC24A1	603617	Night blindness, congenital stationary (complete), 1D, autosomal recessive, 613830 (3), Autosomal recessive
SLC24A4	609840	[Skin/hair/eye pigmentation 6, blond/brown hair], 210750 (3), Autosomal recessive; Amelogenesis imperfecta, type IIA5, 615887 (3), Autosomal recessive; [Skin/hair/eye pigmentation 6, blue/green eyes], 210750 (3), Autosomal recessive
SLC24A5	609802	[Skin/hair/eye pigmentation 4, fair/dark skin], 113750 (3), Autosomal recessive; Albinism, oculocutaneous, type VI, 113750 (3), Autosomal recessive
SLC25A1	190315	Combined D-2- and L-2-hydroxyglutaric aciduria, 615182 (3), Autosomal recessive; Myasthenic syndrome, congenital, 23, presynaptic, 618197 (3), Autosomal recessive
SLC25A10	606794	?Mitochondrial DNA depletion syndrome 19, 618972 (3), Autosomal recessive
SLC25A11	604165	Paragangliomas 6, 618464 (3), Autosomal dominant
SLC25A12	603667	Developmental and epileptic encephalopathy 39, 612949 (3), Autosomal recessive

SLC25A13	603859	Citrullinemia, type II, neonatal-onset, 605814 (3), Autosomal recessive; Citrullinemia, adult-onset type II, 603471 (3), Autosomal recessive
SLC25A15	603861	Hyperornithinemia-hyperammonemia-homocitrullinemia syndrome, 238970 (3), Autosomal recessive
SLC25A19	606521	Microcephaly, Amish type, 607196 (3), Autosomal recessive; Thiamine metabolism dysfunction syndrome 4 (progressive polyneuropathy type), 613710 (3), Autosomal recessive
SLC25A20	613698	Carnitine-acylcarnitine translocase deficiency, 212138 (3), Autosomal recessive
SLC25A21	607571	?Mitochondrial DNA depletion syndrome 18, 618811 (3), Autosomal recessive
SLC25A22	609302	Developmental and epileptic encephalopathy 3, 609304 (3), Autosomal recessive
SLC25A24	608744	Fontaine progeroid syndrome, 612289 (3), Autosomal dominant
SLC25A26	611037	Combined oxidative phosphorylation deficiency 28, 616794 (3), Autosomal recessive
SLC25A3	600370	Mitochondrial phosphate carrier deficiency, 610773 (3), Autosomal recessive
SLC25A32	138480	?Exercise intolerance, riboflavin-responsive, 616839 (3), Autosomal recessive
SLC25A38	610819	Anemia, sideroblastic, 2, pyridoxine-refractory, 205950 (3), Autosomal recessive
SLC25A4	103220	Mitochondrial DNA depletion syndrome 12B (cardiomyopathic type) AR, 615418 (3), Autosomal recessive; Progressive external ophthalmoplegia with mitochondrial DNA deletions, autosomal dominant 2, 609283 (3), Autosomal dominant; Mitochondrial DNA depletion syndrome 12A (cardiomyopathic type) AD, 617184 (3), Autosomal dominant
SLC25A42	610823	Metabolic crises, recurrent, with variable encephalomyopathic features and neurologic regression, 618416 (3), Autosomal recessive
SLC25A46	610826	Neuropathy, hereditary motor and sensory, type VIB, 616505 (3), Autosomal recessive; Pontocerebellar hypoplasia, type 1E, 619303 (3), Autosomal recessive
SLC26A1	610130	?Nephrolithiasis, calcium oxalate, 167030 (3), Autosomal recessive
SLC26A2	606718	Epiphyseal dysplasia, multiple, 4, 226900 (3), Autosomal recessive; De la Chapelle dysplasia, 256050 (3), Autosomal recessive; Diastrophic dysplasia, 222600 (3), Autosomal recessive; Diastrophic dysplasia, broad bone-platyspondylic variant, 222600 (3), Autosomal recessive; Achondrogenesis Ib, 600972 (3), Autosomal recessive; Atelosteogenesis, type II, 256050 (3), Autosomal recessive
SLC26A3	126650	Diarrhea 1, secretory chloride, congenital, 214700 (3), Autosomal recessive
SLC26A4	605646	Deafness, autosomal recessive 4, with enlarged vestibular aqueduct, 600791 (3), Autosomal recessive; Pendred syndrome, 274600 (3), Autosomal recessive
SLC26A5	604943	?Deafness, autosomal recessive 61, 613865 (3), Autosomal recessive

SLC26A8	608480	Spermatogenic failure 3, 606766 (3), Autosomal dominant, Autosomal recessive
SLC27A4	604194	Ichthyosis prematurity syndrome, 608649 (3), Autosomal recessive
SLC28A1	606207	[Uridine-cytidineuria], 618477 (3), Autosomal recessive
SLC29A3	612373	Histiocytosis-lymphadenopathy plus syndrome, 602782 (3), Autosomal recessive
SLC2A1	138140	Dystonia 9, 601042 (3), Autosomal dominant; GLUT1 deficiency syndrome 1, infantile onset, severe, 606777 (3), Autosomal dominant, Autosomal recessive; Stomatin-deficient cryohydrocytosis with neurologic defects, 608885 (3), Autosomal dominant; {Epilepsy, idiopathic generalized, susceptibility to, 12}, 614847 (3), Autosomal dominant; GLUT1 deficiency syndrome 2, childhood onset, 612126 (3), Autosomal dominant
SLC2A10	606145	Arterial tortuosity syndrome, 208050 (3), Autosomal recessive
SLC2A2	138160	Fanconi-Bickel syndrome, 227810 (3), Autosomal recessive; {Diabetes mellitus, noninsulin-dependent}, 125853 (3), Autosomal dominant
SLC2A9	606142	{Uric acid concentration, serum, QTL 2}, 612076 (3), Autosomal dominant, Autosomal recessive; Hypouricemia, renal, 2, 612076 (3), Autosomal dominant, Autosomal recessive
SLC30A10	611146	Hypermanganesemia with dystonia 1, 613280 (3), Autosomal recessive
SLC30A2	609617	Zinc deficiency, transient neonatal, 608118 (3), Autosomal dominant
SLC30A8	611145	{Diabetes mellitus, noninsulin-dependent, susceptibility to}, 125853 (3), Autosomal dominant
SLC30A9	604604	Birk-Landau-Perez syndrome, 617595 (3), Autosomal recessive
SLC33A1	603690	Spastic paraplegia 42, autosomal dominant, 612539 (3), Autosomal dominant; Congenital cataracts, hearing loss, and neurodegeneration, 614482 (3), Autosomal recessive
SLC34A1	182309	?Fanconi renotubular syndrome 2, 613388 (3), Autosomal recessive; Hypercalcemia, infantile, 2, 616963 (3), Autosomal recessive; Nephrolithiasis/osteoporosis, hypophosphatemic, 1, 612286 (3), Autosomal dominant
SLC34A2	604217	Pulmonary alveolar microlithiasis, 265100 (3), Autosomal recessive
SLC34A3	609826	Hypophosphatemic rickets with hypercalciuria, 241530 (3), Autosomal recessive
SLC35A1	605634	Congenital disorder of glycosylation, type IIf, 603585 (3), Autosomal recessive
SLC35A2	314375	Congenital disorder of glycosylation, type IIm, 300896 (3), Somatic mosaicism, X-linked dominant
SLC35A3	605632	Arthrogryposis, impaired intellectual development, and seizures, 615553 (3), Autosomal recessive
SLC35C1	605881	Congenital disorder of glycosylation, type IIc, 266265 (3), Autosomal recessive
SLC35D1	610804	Schneckenbecken dysplasia, 269250 (3), Autosomal recessive
SLC36A2	608331	Iminoglycinuria, digenic, 242600 (3), Digenic recessive, Autosomal recessive; Hyperglycinuria, 138500 (3), Autosomal dominant

SLC37A4	602671	Glycogen storage disease Ib, 232220 (3), Autosomal recessive; Congenital disorder of glycosylation, type IIw, 619525 (3), Autosomal dominant; Glycogen storage disease Ic, 232240 (3), Autosomal recessive
SLC38A3	604437	Developmental and epileptic encephalopathy 102, 619881 (3), Autosomal recessive
SLC38A8	615585	Foveal hypoplasia 2, with or without optic nerve misrouting and/or anterior segment dysgenesis, 609218 (3), Autosomal recessive
SLC39A13	608735	Ehlers-Danlos syndrome, spondylodysplastic type, 3, 612350 (3), Autosomal recessive
SLC39A14	608736	?Hyperostosis cranalis interna, 144755 (3), Autosomal dominant; Hypermanganesemia with dystonia 2, 617013 (3), Autosomal recessive
SLC39A4	607059	Acrodermatitis enteropathica, 201100 (3), Autosomal recessive
SLC39A5	608730	Myopia 24, autosomal dominant, 615946 (3), Autosomal dominant
SLC39A7	601416	Agammaglobulinemia 9, autosomal recessive, 619693 (3), Autosomal recessive
SLC39A8	608732	Congenital disorder of glycosylation, type IIn, 616721 (3), Autosomal recessive
SLC3A1	104614	Cystinuria, 220100 (3), Autosomal dominant, Autosomal recessive
SLC40A1	604653	Hemochromatosis, type 4, 606069 (3), Autosomal dominant
SLC41A1	610801	?Nephronophthisis-like nephropathy 2, 619468 (3), Autosomal recessive
SLC44A1	606105	Neurodegeneration, childhood-onset, with ataxia, tremor, optic atrophy, and cognitive decline, 618868 (3), Autosomal recessive
SLC44A4	606107	?Deafness, autosomal dominant 72, 617606 (3), Autosomal dominant
SLC45A1	605763	Intellectual developmental disorder with neuropsychiatric features, 617532 (3), Autosomal recessive
SLC45A2	606202	[Skin/hair/eye pigmentation 5, dark/light eyes], 227240 (3), Autosomal recessive; [Skin/hair/eye pigmentation 5, black/nonblack hair], 227240 (3), Autosomal recessive; Albinism, oculocutaneous, type IV, 606574 (3), Autosomal recessive; [Skin/hair/eye pigmentation 5, dark/fair skin], 227240 (3), Autosomal recessive
SLC46A1	611672	Folate malabsorption, hereditary, 229050 (3), Autosomal recessive
SLC4A1	109270	[Blood group, Swann], 601550 (3); [Blood group, Wright], 112050 (3); Distal renal tubular acidosis 1, 179800 (3), Autosomal dominant; [Blood group, Waldner], 112010 (3); Spherocytosis, type 4, 612653 (3), Autosomal dominant; [Blood group, Froese], 601551 (3); Distal renal tubular acidosis 4 with hemolytic anemia, 611590 (3), Autosomal recessive; {Malaria, resistance to}, 611162 (3); Cryohydrocytosis, 185020 (3), Autosomal dominant; Ovalocytosis, SA type, 166900 (3), Autosomal dominant; [Blood group, Diego], 110500 (3)
SLC4A11	610206	Corneal endothelial dystrophy, autosomal recessive, 217700 (3), Autosomal recessive; Corneal dystrophy, Fuchs endothelial, 4, 613268 (3); Corneal endothelial dystrophy and perceptive deafness, 217400 (3), Autosomal recessive

SLC4A4	603345	Renal tubular acidosis, proximal, with ocular abnormalities, 604278 (3), Autosomal recessive
SLC51A	612084	?Cholestasis, progressive familial intrahepatic, 6, 619484 (3), Autosomal recessive
SLC51B	612085	?Bile acid malabsorption, primary, 2, 619481 (3), Autosomal recessive
SLC52A1	607883	Riboflavin deficiency, 615026 (3), Autosomal dominant
SLC52A2	607882	Brown-Vialetto-Van Laere syndrome 2, 614707 (3), Autosomal recessive
SLC52A3	613350	?Fazio-Londe disease, 211500 (3), Autosomal recessive; Brown-Vialetto-Van Laere syndrome 1, 211530 (3), Autosomal recessive
SLC5A1	182380	Glucose/galactose malabsorption, 606824 (3), Autosomal recessive
SLC5A2	182381	Renal glucosuria, 233100 (3), Autosomal dominant, Autosomal recessive
SLC5A5	601843	Thyroid dyshormonogenesis 1, 274400 (3), Autosomal recessive
SLC5A6	604024	Sodium-dependent multivitamin transporter deficiency, 618973 (3), Autosomal recessive; Peripheral motor neuropathy, childhood-onset, biotin-responsive, 619903 (3), Autosomal recessive
SLC5A7	608761	Neuronopathy, distal hereditary motor, type VIIA, 158580 (3), Autosomal dominant; Myasthenic syndrome, congenital, 20, presynaptic, 617143 (3), Autosomal recessive
SLC6A1	137165	Myoclonic-atonic epilepsy, 616421 (3), Autosomal dominant
SLC6A17	610299	Intellectual developmental disorder, autosomal recessive 48, 616269 (3), Autosomal recessive
SLC6A19	608893	Iminoglycinuria, digenic, 242600 (3), Digenic recessive, Autosomal recessive; Hartnup disorder, 234500 (3), Autosomal recessive; Hyperglycinuria, 138500 (3), Autosomal dominant
SLC6A2	163970	?Orthostatic intolerance, 604715 (3), Autosomal dominant
SLC6A20	605616	Iminoglycinuria, digenic, 242600 (3), Digenic recessive, Autosomal recessive; Hyperglycinuria, 138500 (3), Autosomal dominant
SLC6A3	126455	Parkinsonism-dystonia, infantile, 1, 613135 (3), Autosomal recessive; {Nicotine dependence, protection against}, 188890 (3)
SLC6A4	182138	{Obsessive-compulsive disorder}, 164230 (3), Autosomal dominant; {Anxiety-related personality traits}, 607834 (3)
SLC6A5	604159	Hyperekplexia 3, 614618 (3), Autosomal dominant, Autosomal recessive
SLC6A6	186854	Hypotaurinemic retinal degeneration and cardiomyopathy, 145350 (3), Autosomal recessive
SLC6A8	300036	Cerebral creatine deficiency syndrome 1, 300352 (3), X-linked recessive
SLC6A9	601019	Glycine encephalopathy with normal serum glycine, 617301 (3), Autosomal recessive
SLC7A14	615720	Retinitis pigmentosa 68, 615725 (3), Autosomal recessive
SLC7A6OS	619192	Epilepsy, progressive myoclonic, 12, 619191 (3), Autosomal recessive
SLC7A7	603593	Lysinuric protein intolerance, 222700 (3), Autosomal recessive
SLC7A9	604144	Cystinuria, 220100 (3), Autosomal dominant, Autosomal recessive
SLC9A1	107310	Lichtenstein-Knorr syndrome, 616291 (3), Autosomal recessive
SLC9A3	182307	Diarrhea 8, secretory sodium, congenital, 616868 (3), Autosomal recessive

SLC9A3R1	604990	Nephrolithiasis/osteoporosis, hypophosphatemic, 2, 612287 (3), Autosomal dominant
SLC9A6	300231	Intellectual developmental disorder, X-linked syndromic, Christianson type, 300243 (3), X-linked
SLC9A7	300368	Intellectual developmental disorder, X-linked 108, 301024 (3), X-linked recessive
SLC9A9	608396	{?Autism susceptibility 16}, 613410 (3)
SLCO1B1	604843	Hyperbilirubinemia, Rotor type, digenic, 237450 (3), Digenic recessive
SLCO1B3	605495	Hyperbilirubinemia, Rotor type, digenic, 237450 (3), Digenic recessive
SLCO2A1	601460	Hypertrophic osteoarthropathy, primary, autosomal dominant, 167100 (3), Autosomal dominant; Hypertrophic osteoarthropathy, primary, autosomal recessive 2, 614441 (3), Autosomal recessive
SLFN14	614958	Bleeding disorder, platelet-type, 20, 616913 (3), Autosomal dominant
SLITRK1	609678	Tourette syndrome, 137580 (3), Autosomal dominant; ?Trichotillomania, 613229 (3), Multifactorial, Autosomal dominant
SLITRK6	609681	Deafness and myopia, 221200 (3), Autosomal recessive
SLURP1	606119	Meleda disease, 248300 (3), Autosomal recessive
SLX4	613278	Fanconi anemia, complementation group P, 613951 (3), Autosomal recessive
SMAD2	601366	Loeys-Dietz syndrome 6, 619656 (3), Autosomal dominant; Congenital heart defects, multiple types, 8, with or without heterotaxy, 619657 (3), Autosomal dominant
SMAD3	603109	Loeys-Dietz syndrome 3, 613795 (3), Autosomal dominant
SMAD4	600993	Pancreatic cancer, somatic, 260350 (3); Myhre syndrome, 139210 (3), Autosomal dominant; Polyposis, juvenile intestinal, 174900 (3), Autosomal dominant; Juvenile polyposis/hereditary hemorrhagic telangiectasia syndrome, 175050 (3), Autosomal dominant
SMAD6	602931	Aortic valve disease 2, 614823 (3), Autosomal dominant; {Radioulnar synostosis, nonsyndromic}, 179300 (3), Autosomal dominant; {Craniosynostosis 7, susceptibility to}, 617439 (3), Autosomal dominant
SMAD7	602932	{Colorectal cancer, susceptibility to, 3}, 612229 (3)
SMAD9	603295	Pulmonary hypertension, primary, 2, 615342 (3), Autosomal dominant
SMARCA2	600014	Nicolaides-Baraitser syndrome, 601358 (3), Autosomal dominant; Blepharophimosis-impaired intellectual development syndrome, 619293 (3), Autosomal dominant
SMARCA4	603254	Coffin-Siris syndrome 4, 614609 (3), Autosomal dominant; {Rhabdoid tumor predisposition syndrome 2}, 613325 (3), Autosomal dominant
SMARCAD1	612761	Basan syndrome, 129200 (3), Autosomal dominant; Huriez syndrome, 181600 (3), Autosomal dominant; Adermatoglyphia, 136000 (3), Autosomal dominant
SMARCAL1	606622	Schimke immunoosseous dysplasia, 242900 (3), Autosomal recessive

SMARCB1	601607	Rhabdoid tumors, somatic, 609322 (3); {Schwannomatosis-1, susceptibility to}, 162091 (3), Autosomal dominant; Coffin-Siris syndrome 3, 614608 (3), Autosomal dominant; {Rhabdoid tumor predisposition syndrome 1}, 609322 (3), Autosomal dominant
SMARCC2	601734	Coffin-Siris syndrome 8, 618362 (3), Autosomal dominant
SMARCD1	601735	Coffin-Siris syndrome 11, 618779 (3), Autosomal dominant
SMARCD2	601736	Specific granule deficiency 2, 617475 (3), Autosomal recessive
SMARCE1	603111	{Meningioma, familial, susceptibility to}, 607174 (3), Autosomal dominant; Coffin-Siris syndrome 5, 616938 (3), Autosomal dominant
SMC1A	300040	Cornelia de Lange syndrome 2, 300590 (3), X-linked dominant; Developmental and epileptic encephalopathy 85, with or without midline brain defects, 301044 (3), X-linked dominant
SMC3	606062	Cornelia de Lange syndrome 3, 610759 (3), Autosomal dominant
SMCHD1	614982	Bosma arhinia microphthalmia syndrome, 603457 (3), Autosomal dominant; Fascioscapulohumeral muscular dystrophy 2, digenic, 158901 (3), Digenic dominant
SMG8	613175	Alzahrani-Kuwahara syndrome, 619268 (3), Autosomal recessive
SMG9	613176	Heart and brain malformation syndrome, 616920 (3), Autosomal recessive; Neurodevelopmental disorder with intention tremor, pyramidal signs, dyspraxia, and ocular anomalies, 619995 (3), Autosomal recessive
SMIM1	615242	[Blood group, Vel system], 615264 (3), Autosomal recessive
SMN1	600354	Spinal muscular atrophy-2, 253550 (3), Autosomal recessive; Spinal muscular atrophy-4, 271150 (3), Autosomal recessive; Spinal muscular atrophy-3, 253400 (3), Autosomal recessive; Spinal muscular atrophy-1, 253300 (3), Autosomal recessive
SMN2	601627	{Spinal muscular atrophy, type III, modifier of}, 253400 (3), Autosomal recessive
SMO	601500	Pallister-Hall-like syndrome, 241800 (3), Autosomal recessive; Basal cell carcinoma, somatic, 605462 (3); Curry-Jones syndrome, somatic mosaic, 601707 (3)
SMOC1	608488	Microphthalmia with limb anomalies, 206920 (3), Autosomal recessive
SMOC2	607223	Dentin dysplasia, type I, with microdontia and misshapen teeth, 125400 (3), Autosomal recessive
SMPD1	607608	Niemann-Pick disease, type B, 607616 (3), Autosomal recessive; Niemann-Pick disease, type A, 257200 (3), Autosomal recessive
SMPD4	610457	Neurodevelopmental disorder with microcephaly, arthrogryposis, and structural brain anomalies, 618622 (3), Autosomal recessive
SMPX	300226	Myopathy, distal, 7, adult-onset, X-linked, 301075 (3), X-linked recessive; Deafness, X-linked 4, 300066 (3), X-linked dominant
SMS	300105	Intellectual developmental disorder, X-linked syndromic, Snyder-Robinson type, 309583 (3), X-linked recessive
SNAI2	602150	Waardenburg syndrome, type 2D, 608890 (3), Autosomal recessive; Piebaldism, 172800 (3), Autosomal dominant
SNAP25	600322	?Myasthenic syndrome, congenital, 18, 616330 (3), Autosomal dominant

SNAP29	604202	Cerebral dysgenesis, neuropathy, ichthyosis, and palmoplantar keratoderma syndrome, 609528 (3), Autosomal recessive
SNCA	163890	Dementia, Lewy body, 127750 (3), Autosomal dominant; Parkinson disease 1, 168601 (3), Autosomal dominant; Parkinson disease 4, 605543 (3), Autosomal dominant
SNCB	602569	Dementia, Lewy body, 127750 (3), Autosomal dominant
SNIP1	608241	Neurodevelopmental disorder with hypotonia, craniofacial abnormalities, and seizures, 614501 (3), Autosomal recessive
SNORA31	619378	{Encephalopathy, acute, infection-induced (herpes-specific), susceptibility to, 10}, 619396 (3), Autosomal dominant
SNORD118	616663	Leukoencephalopathy, brain calcifications, and cysts, 614561 (3), Autosomal recessive
SNRNP200	601664	Retinitis pigmentosa 33, 610359 (3), Autosomal dominant
SNRPB	182282	Cerebrocostomandibular syndrome, 117650 (3), Autosomal dominant
SNRPE	128260	Hypotrichosis 11, 615059 (3), Autosomal dominant
SNRPN	182279	Prader-Willi syndrome, 176270 (3), Autosomal dominant
SNTA1	601017	Long QT syndrome 12, 612955 (3), Autosomal dominant
SNX10	614780	Osteopetrosis, autosomal recessive 8, 615085 (3), Autosomal recessive
SNX14	616105	Spinocerebellar ataxia, autosomal recessive 20, 616354 (3), Autosomal recessive
SOBP	613667	Mental retardation, anterior maxillary protrusion, and strabismus, 613671 (3), Autosomal recessive
SOCS1	603597	Autoinflammatory syndrome, familial, with or without immunodeficiency, 619375 (3), Autosomal dominant
SOD1	147450	Spastic tetraplegia and axial hypotonia, progressive, 618598 (3), Autosomal recessive; Amyotrophic lateral sclerosis 1, 105400 (3), Autosomal dominant, Autosomal recessive
SOD2	147460	{Microvascular complications of diabetes 6}, 612634 (3)
SOD3	185490	[Superoxide dismutase, elevated extracellular] (3)
SOHLH1	610224	Ovarian dysgenesis 5, 617690 (3), Autosomal recessive; Spermatogenic failure 32, 618115 (3), Autosomal dominant
SON	182465	ZTTK syndrome, 617140 (3), Autosomal dominant
SORD	182500	Sorbitol dehydrogenase deficiency with peripheral neuropathy, 618912 (3), Autosomal recessive
SORT1	602458	[Low density lipoprotein cholesterol level QTL6], 613589 (3), Autosomal dominant
SOS1	182530	Noonan syndrome 4, 610733 (3), Autosomal dominant; ?Fibromatosis, gingival, 1, 135300 (3), Autosomal dominant
SOS2	601247	Noonan syndrome 9, 616559 (3), Autosomal dominant
SOST	605740	Sclerosteosis 1, 269500 (3), Autosomal recessive; Craniodiaphyseal dysplasia, autosomal dominant, 122860 (3), Autosomal dominant
SOX10	602229	Waardenburg syndrome, type 4C, 613266 (3), Autosomal dominant; PCWH syndrome, 609136 (3), Autosomal dominant; Waardenburg syndrome,

		type 2E, with or without neurologic involvement, 611584 (3), Autosomal dominant
SOX11	600898	Coffin-Siris syndrome 9, 615866 (3), Autosomal dominant
SOX17	610928	Vesicoureteral reflux 3, 613674 (3), Autosomal dominant
SOX18	601618	Hypotrichosis-lymphedema-telangiectasia syndrome, 607823 (3), Autosomal recessive; Hypotrichosis-lymphedema-telangiectasia-renal defect syndrome, 137940 (3), Autosomal dominant
SOX2	184429	Optic nerve hypoplasia and abnormalities of the central nervous system, 206900 (3), Autosomal dominant; Microphthalmia, syndromic 3, 206900 (3), Autosomal dominant
SOX3	313430	Intellectual developmental disorder, X-linked, with isolated growth hormone deficiency, 300123 (3); Panhypopituitarism, X-linked, 312000 (3), X-linked
SOX4	184430	Coffin-Siris syndrome 10, 618506 (3), Autosomal dominant
SOX5	604975	Lamb-Shaffer syndrome, 616803 (3), Autosomal dominant
SOX6	607257	Tolchin-Le Caignec syndrome, 618971 (3), Autosomal dominant
SOX9	608160	Campomelic dysplasia with autosomal sex reversal, 114290 (3), Autosomal dominant; Acampomelic campomelic dysplasia, 114290 (3), Autosomal dominant; Campomelic dysplasia, 114290 (3), Autosomal dominant
SP110	604457	{Mycobacterium tuberculosis, susceptibility to}, 607948 (3); Hepatic venoocclusive disease with immunodeficiency, 235550 (3), Autosomal recessive
SP7	606633	Osteogenesis imperfecta, type XII, 613849 (3), Autosomal recessive
SPAG1	603395	Ciliary dyskinesia, primary, 28, 615505 (3), Autosomal recessive
SPAG17	616554	?Spermatogenic failure 55, 619380 (3), Autosomal recessive
SPARC	182120	Osteogenesis imperfecta, type XVII, 616507 (3), Autosomal recessive
SPART	607111	Troyer syndrome, 275900 (3), Autosomal recessive
SPAST	604277	Spastic paraplegia 4, autosomal dominant, 182601 (3), Autosomal dominant
SPATA16	609856	?Spermatogenic failure 6, 102530 (3), Autosomal recessive
SPATA5	613940	Neurodevelopmental disorder with hearing loss, seizures, and brain abnormalities, 616577 (3), Autosomal recessive
SPATA5L1	619578	Deafness, autosomal recessive 119, 619615 (3), Autosomal recessive; Neurodevelopmental disorder with hearing loss and spasticity, 619616 (3), Autosomal recessive
SPATA7	609868	Leber congenital amaurosis 3, 604232 (3), Autosomal recessive; Retinitis pigmentosa 94, variable age at onset, autosomal recessive, 604232 (3), Autosomal recessive
SPECC1L	614140	Teebi hypertelorism syndrome 1, 145420 (3), Autosomal dominant; ?Facial clefting, oblique, 1, 600251 (3), Autosomal dominant
SPEF2	610172	Spermatogenic failure 43, 618751 (3), Autosomal recessive
SPEG	615950	Centronuclear myopathy 5, 615959 (3), Autosomal recessive
SPEN	613484	Radio-Tartaglia syndrome, 619312 (3), Autosomal dominant

SPG11	610844	Amyotrophic lateral sclerosis 5, juvenile, 602099 (3), Autosomal recessive; Charcot-Marie-Tooth disease, axonal, type 2X, 616668 (3), Autosomal recessive; Spastic paraplegia 11, autosomal recessive, 604360 (3), Autosomal recessive
SPG21	608181	Mast syndrome, 248900 (3), Autosomal recessive
SPG7	602783	Spastic paraplegia 7, autosomal recessive, 607259 (3), Autosomal dominant, Autosomal recessive
SPI1	165170	Agammaglobulinemia 10, autosomal dominant, 619707 (3), Autosomal dominant
SPIDR	615384	Ovarian dysgenesis 9, 619665 (3), Autosomal recessive
SPINK1	167790	Tropical calcific pancreatitis, 608189 (3), Autosomal dominant, Autosomal recessive; Pancreatitis, hereditary, 167800 (3), Autosomal dominant; {Fibrocalculous pancreatic diabetes, susceptibility to}, 608189 (3), Autosomal dominant, Autosomal recessive
SPINK2	605753	?Spermatogenic failure 29, 618091 (3), Autosomal recessive
SPINK5	605010	Netherton syndrome, 256500 (3), Autosomal recessive
SPINT2	605124	Diarrhea 3, secretory sodium, congenital, syndromic, 270420 (3), Autosomal recessive
SPNS2	612584	?Deafness, autosomal recessive 115, 618457 (3), Autosomal recessive
SPOP	602650	Nabais Sa-de Vries syndrome, type 1, 618828 (3), Autosomal dominant; Nabais Sa-de Vries syndrome, type 2, 618829 (3), Autosomal dominant
SPPL2A	608238	Immunodeficiency 86, mycobacteriosis, 619549 (3), Autosomal recessive
SPR	182125	Dystonia, dopa-responsive, due to sepiapterin reductase deficiency, 612716 (3), ?Autosomal dominant, Autosomal recessive
SPRED1	609291	Legius syndrome, 611431 (3), Autosomal dominant
SPRED2	609292	Noonan syndrome 14, 619745 (3), Autosomal recessive
SPRTN	616086	Ruijs-Aalfs syndrome, 616200 (3), Autosomal recessive
SPRY2	602466	{?IgA nephropathy, susceptibility to, 3}, 616818 (3), Autosomal dominant
SPRY4	607984	Hypogonadotropic hypogonadism 17 with or without anosmia, 615266 (3), Autosomal dominant
SPTA1	182860	Spherocytosis, type 3, 270970 (3), Autosomal recessive; Elliptocytosis-2, 130600 (3), Autosomal dominant; Pyropoikilocytosis, 266140 (3), Autosomal recessive
SPTAN1	182810	Developmental and epileptic encephalopathy 5, 613477 (3), Autosomal dominant
SPTB	182870	Anemia, neonatal hemolytic, fatal or near-fatal, 617948 (3); Elliptocytosis-3, 617948 (3); Spherocytosis, type 2, 616649 (3), Autosomal dominant
SPTBN1	182790	Developmental delay, impaired speech, and behavioral abnormalities, 619475 (3), Autosomal dominant
SPTBN2	604985	Spinocerebellar ataxia 5, 600224 (3), Autosomal dominant; Spinocerebellar ataxia, autosomal recessive 14, 615386 (3), Autosomal recessive
SPTBN4	606214	Neurodevelopmental disorder with hypotonia, neuropathy, and deafness, 617519 (3), Autosomal recessive

SPTLC1	605712	Neuropathy, hereditary sensory and autonomic, type IA, 162400 (3), Autosomal dominant
SPTLC2	605713	Neuropathy, hereditary sensory and autonomic, type IC, 613640 (3), Autosomal dominant
SQOR	617658	Sulfide:quinone oxidoreductase deficiency, 619221 (3), Autosomal recessive
SQSTM1	601530	Neurodegeneration with ataxia, dystonia, and gaze palsy, childhood-onset, 617145 (3), Autosomal recessive; Frontotemporal dementia and/or amyotrophic lateral sclerosis 3, 616437 (3), Autosomal dominant; Myopathy, distal, with rimmed vacuoles, 617158 (3), Autosomal dominant; Paget disease of bone 3, 167250 (3), Autosomal dominant
SRC	190090	?Thrombocytopenia 6, 616937 (3), Autosomal dominant; Colon cancer, advanced, somatic, 114500 (3)
SRCAP	611421	Developmental delay, hypotonia, musculoskeletal defects, and behavioral abnormalities, 619595 (3), Autosomal dominant; Floating-Harbor syndrome, 136140 (3), Autosomal dominant
SRD5A2	607306	Pseudovaginal perineoscrotal hypospadias, 264600 (3), Autosomal recessive
SRD5A3	611715	Kahrizi syndrome, 612713 (3), Autosomal recessive; Congenital disorder of glycosylation, type Iq, 612379 (3), Autosomal recessive
SREBF1	184756	Ichthyosis, follicular, with atrichia and photophobia syndrome 2, 619016 (3), Autosomal dominant; Mucoepithelial dysplasia, hereditary, 158310 (3), Autosomal dominant
SRGAP1	606523	{Thyroid cancer, nonmedullary, 2}, 188470 (3), Autosomal dominant, Somatic mutation
SRP54	604857	Neutropenia, severe congenital, 8, autosomal dominant, 618752 (3), Autosomal dominant
SRP72	602122	Bone marrow failure syndrome 1, 614675 (3), Autosomal dominant
SRPX2	300642	?Rolandic epilepsy, impaired intellectual development, and speech dyspraxia, 300643 (3)
SRY	480000	46XY sex reversal 1, 400044 (3), Y-linked; 46XX sex reversal 1, 400045 (4), X-linked dominant
SS18	600192	Sarcoma, synovial (1)
SSBP1	600439	Optic atrophy 13 with retinal and foveal abnormalities, 165510 (3), Autosomal dominant
SSR4	300090	Congenital disorder of glycosylation, type Iy, 300934 (3), X-linked recessive
SSX1	312820	?Sarcoma, synovial, 300813 (3)
SSX2	300192	?Sarcoma, synovial, 300813 (3)
ST14	606797	Ichthyosis, congenital, autosomal recessive 11, 602400 (3), Autosomal recessive
ST3GAL3	606494	Developmental and epileptic encephalopathy 15, 615006 (3), Autosomal recessive; Intellectual developmental disorder, autosomal recessive 12, 611090 (3), Autosomal recessive

ST3GAL5	604402	Salt and pepper developmental regression syndrome, 609056 (3), Autosomal recessive
STAC3	615521	Myopathy, congenital, Baily-Bloch, 255995 (3), Autosomal recessive
STAG1	604358	Intellectual developmental disorder, autosomal dominant 47, 617635 (3), Autosomal dominant
STAG2	300826	Holoprosencephaly 13, X-linked, 301043 (3), X-linked recessive, X-linked dominant; Mullegama-Klein-Martinez syndrome, 301022 (3), X-linked
STAG3	608489	Spermatogenic failure 61, 619672 (3), Autosomal recessive; Premature ovarian failure 8, 615723 (3), Autosomal recessive
STAMBP	606247	Microcephaly-capillary malformation syndrome, 614261 (3), Autosomal recessive
STAR	600617	Lipoid adrenal hyperplasia, 201710 (3), Autosomal recessive
STARD7	616712	Epilepsy, familial adult myoclonic, 2, 607876 (3), Autosomal dominant
STAT1	600555	Immunodeficiency 31C, chronic mucocutaneous candidiasis, autosomal dominant, 614162 (3), Autosomal dominant; Immunodeficiency 31A, mycobacteriosis, autosomal dominant, 614892 (3), Autosomal dominant; Immunodeficiency 31B, mycobacterial and viral infections, autosomal recessive, 613796 (3), Autosomal recessive
STAT2	600556	Pseudo-TORCH syndrome 3, 618886 (3), Autosomal recessive; Immunodeficiency 44, 616636 (3), Autosomal recessive
STAT3	102582	Hyper-IgE recurrent infection syndrome, 147060 (3), Autosomal dominant; Autoimmune disease, multisystem, infantile-onset, 1, 615952 (3), Autosomal dominant
STAT4	600558	{Systemic lupus erythematosus, susceptibility to, 11}, 612253 (3)
STAT5B	604260	Growth hormone insensitivity with immune dysregulation 1, autosomal recessive, 245590 (3), Autosomal recessive; Growth hormone insensitivity with immune dysregulation 2, autosomal dominant, 618985 (3), Autosomal dominant; Leukemia, acute promyelocytic, somatic, 102578 (3)
STEAP3	609671	?Anemia, hypochromic microcytic, with iron overload 2, 615234 (3), Autosomal dominant
STEEP1	301012	?Intellectual developmental disorder, X-linked 107, 301013 (3), X-linked
STIL	181590	Microcephaly 7, primary, autosomal recessive, 612703 (3), Autosomal recessive
STIM1	605921	Myopathy, tubular aggregate, 1, 160565 (3), Autosomal dominant; Stormorken syndrome, 185070 (3), Autosomal dominant; Immunodeficiency 10, 612783 (3), Autosomal recessive
STING1	612374	STING-associated vasculopathy, infantile-onset, 615934 (3), Autosomal dominant
STK11	602216	Melanoma, malignant, somatic, 155600 (3); Pancreatic cancer, somatic, 260350 (3); Peutz-Jeghers syndrome, 175200 (3), Autosomal dominant; Testicular tumor, somatic, 273300 (3)
STK36	607652	?Ciliary dyskinesia, primary, 46, 619436 (3), Autosomal recessive
STK4	604965	T-cell immunodeficiency, recurrent infections, autoimmunity, and cardiac malformations, 614868 (3)

STN1	613128	Cerebroretinal microangiopathy with calcifications and cysts 2, 617341 (3), Autosomal recessive
STOX1	609397	Preeclampsia/eclampsia 4, 609404 (3), Autosomal dominant
STRA6	610745	Microphthalmia, syndromic 9, 601186 (3), Autosomal recessive; Microphthalmia, isolated, with coloboma 8, 601186 (3), Autosomal recessive
STRADA	608626	Polyhydramnios, megalencephaly, and symptomatic epilepsy, 611087 (3), Autosomal recessive
STRC	606440	Deafness, autosomal recessive 16, 603720 (3), Autosomal recessive
STS	300747	Ichthyosis, X-linked, 308100 (3), X-linked recessive
STT3A	601134	Congenital disorder of glycosylation, type Iw, autosomal dominant, 619714 (3), Autosomal dominant; Congenital disorder of glycosylation, type Iw, autosomal recessive, 615596 (3), Autosomal recessive
STT3B	608605	Congenital disorder of glycosylation, type Ix, 615597 (3), Autosomal recessive
STUB1	607207	Spinocerebellar ataxia 48, 618093 (3), Autosomal dominant; Spinocerebellar ataxia, autosomal recessive 16, 615768 (3), Autosomal recessive
STX11	605014	Hemophagocytic lymphohistiocytosis, familial, 4, 603552 (3), Autosomal recessive
STX16	603666	Pseudohypoparathyroidism, type IB, 603233 (3), Autosomal dominant
STX1B	601485	Generalized epilepsy with febrile seizures plus, type 9, 616172 (3), Autosomal dominant
STX3	600876	Retinal dystrophy and microvillus inclusion disease, 619446 (3), Autosomal recessive; Diarrhea 12, with microvillus atrophy, 619445 (3), Autosomal recessive
STXBP1	602926	Developmental and epileptic encephalopathy 4, 612164 (3), Autosomal dominant
STXBP2	601717	Hemophagocytic lymphohistiocytosis, familial, 5, with or without microvillus inclusion disease, 613101 (3)
SUCLA2	603921	Mitochondrial DNA depletion syndrome 5 (encephalomyopathic with or without methylmalonic aciduria), 612073 (3), Autosomal recessive
SUCLG1	611224	Mitochondrial DNA depletion syndrome 9 (encephalomyopathic type with methylmalonic aciduria), 245400 (3), Autosomal recessive
SUFU	607035	{Meningioma, familial, susceptibility to}, 607174 (3), Autosomal dominant; Joubert syndrome 32, 617757 (3), Autosomal recessive; Medulloblastoma, desmoplastic, 155255 (3), Autosomal dominant, Somatic mutation, Autosomal recessive; Basal cell nevus syndrome, 109400 (3), Autosomal dominant
SUGCT	609187	Glutaric aciduria III, 231690 (3), Autosomal recessive
SULT2B1	604125	Ichthyosis, congenital, autosomal recessive 14, 617571 (3), Autosomal recessive
SUMF1	607939	Multiple sulfatase deficiency, 272200 (3), Autosomal recessive
SUMO1	601912	?Orofacial cleft 10, 613705 (3), Isolated cases
SUMO4	608829	{Diabetes mellitus, insulin-dependent, 5}, 600320 (3)

SUN5	613942	Spermatogenic failure 16, 617187 (3), Autosomal recessive
SUOX	606887	Sulfite oxidase deficiency, 272300 (3), Autosomal recessive
SUPT16H	605012	Neurodevelopmental disorder with dysmorphic facies and thin corpus callosum, 619480 (3), Autosomal dominant
SURF1	185620	Charcot-Marie-Tooth disease, type 4K, 616684 (3), Autosomal recessive; Mitochondrial complex IV deficiency, nuclear type 1, 220110 (3), Autosomal recessive
SUZ12	606245	Imagawa-Matsumoto syndrome, 618786 (3), Autosomal dominant
SVBP	617853	Neurodevelopmental disorder with ataxia, hypotonia, and microcephaly, 618569 (3), Autosomal recessive
SVIL	604126	Myofibrillar myopathy 10, 619040 (3), Autosomal recessive
SYCE1	611486	?Spermatogenic failure 15, 616950 (3), Autosomal recessive; ?Premature ovarian failure 12, 616947 (3), Autosomal recessive
SYCP2	604105	Spermatogenic failure 1, 258150 (3), Autosomal dominant
SYCP3	604759	Pregnancy loss, recurrent, 4, 270960 (3), Autosomal dominant; Spermatogenic failure 4, 270960 (3), Autosomal dominant
SYK	600085	Immunodeficiency 82 with systemic inflammation, 619381 (3), Autosomal dominant
SYN1	313440	Intellectual developmental disorder, X-linked 50, 300115 (3), X-linked; Epilepsy, X-linked, with variable learning disabilities and behavior disorders, 300491 (3), X-linked recessive, X-linked dominant
SYN2	600755	{Schizophrenia, susceptibility to}, 181500 (3), Autosomal dominant
SYNE1	608441	Arthrogryposis multiplex congenita 3, myogenic type, 618484 (3), Autosomal recessive; Emery-Dreifuss muscular dystrophy 4, autosomal dominant, 612998 (3), Autosomal dominant; Spinocerebellar ataxia, autosomal recessive 8, 610743 (3), Autosomal recessive
SYNE2	608442	Emery-Dreifuss muscular dystrophy 5, autosomal dominant, 612999 (3), Autosomal dominant
SYNE4	615535	Deafness, autosomal recessive 76, 615540 (3), Autosomal recessive
SYNGAP1	603384	Intellectual developmental disorder, autosomal dominant 5, 612621 (3), Autosomal dominant
SYNJ1	604297	Parkinson disease 20, early-onset, 615530 (3), Autosomal recessive; Developmental and epileptic encephalopathy 53, 617389 (3), Autosomal recessive
SYP	313475	Intellectual developmental disorder, X-linked 96, 300802 (3), X-linked recessive
SYT1	185605	Baker-Gordon syndrome, 618218 (3), Autosomal dominant
SYT14	610949	?Spinocerebellar ataxia, autosomal recessive 11, 614229 (3), Autosomal recessive
SYT2	600104	Myasthenic syndrome, congenital, 7A, presynaptic, and distal motor neuropathy, autosomal dominant, 616040 (3), Autosomal dominant; Myasthenic syndrome, congenital, 7B, presynaptic, autosomal recessive, 619461 (3), Autosomal recessive

SZT2	615463	Developmental and epileptic encephalopathy 18, 615476 (3), Autosomal recessive
TAB2	605101	Congenital heart defects, nonsyndromic, 2, 614980 (3), Autosomal dominant
TAC3	162330	Hypogonadotropic hypogonadism 10 with or without anosmia, 614839 (3), Autosomal recessive
TACO1	612958	Mitochondrial complex IV deficiency, nuclear type 8, 619052 (3), Autosomal recessive
TACR3	162332	Hypogonadotropic hypogonadism 11 with or without anosmia, 614840 (3), Autosomal recessive
TACSTD2	137290	Corneal dystrophy, gelatinous drop-like, 204870 (3), Autosomal recessive
TAF1	313650	Intellectual developmental disorder, X-linked syndromic 33, 300966 (3), X-linked recessive; Dystonia-Parkinsonism, X-linked, 314250 (3), X-linked recessive
TAF13	600774	Intellectual developmental disorder, autosomal recessive 60, 617432 (3), Autosomal recessive
TAF15	601574	Chondrosarcoma, extraskeletal myxoid, 612237 (1)
TAF2	604912	Intellectual developmental disorder, autosomal recessive 40, 615599 (3), Autosomal recessive
TAF4B	601689	?Spermatogenic failure 13, 615841 (3), Autosomal recessive
TAF6	602955	Alazami-Yuan syndrome, 617126 (3), Autosomal recessive
TAF8	609514	Neurodevelopmental disorder with severe motor impairment, absent language, cerebral hypomyelination, and brain atrophy, 619972 (3), Autosomal recessive
TAFAZZIN	300394	Barth syndrome, 302060 (3), X-linked recessive
TAL1	187040	Leukemia, T-cell acute lymphocytic, somatic, 613065 (3)
TAL2	186855	Leukemia, T-cell acute lymphocytic, somatic, 613065 (3)
TALDO1	602063	Transaldolase deficiency, 606003 (3), Autosomal recessive
TANC2	615047	Intellectual developmental disorder with autistic features and language delay, with or without seizures, 618906 (3), Autosomal dominant
TANGO2	616830	Metabolic encephalomyopathic crises, recurrent, with rhabdomyolysis, cardiac arrhythmias, and neurodegeneration, 616878 (3), Autosomal recessive
TAOK1	610266	Developmental delay with or without intellectual impairment or behavioral abnormalities, 619575 (3), Autosomal dominant
TAP1	170260	Bare lymphocyte syndrome, type I, 604571 (3), Autosomal recessive
TAP2	170261	Bare lymphocyte syndrome, type I, due to TAP2 deficiency, 604571 (3), Autosomal recessive
TAPBP	601962	Bare lymphocyte syndrome, type I, 604571 (3), Autosomal recessive
TAPT1	612758	Osteochondrodysplasia, complex lethal, Symoens-Barnes-Gistelinck type, 616897 (3), Autosomal recessive
TARDBP	605078	Frontotemporal lobar degeneration, TARDBP-related, 612069 (3), Autosomal dominant; Amyotrophic lateral sclerosis 10, with or without FTD, 612069 (3), Autosomal dominant

TARS1	187790	Trichothiodystrophy 7, nonphotosensitive, 618546 (3), Autosomal recessive
TARS2	612805	Combined oxidative phosphorylation deficiency 21, 615918 (3), Autosomal recessive
TAS2R16	604867	{Alcohol dependence, susceptibility to}, 103780 (3), Multifactorial; [Beta-glycopyranoside tasting], 617956 (3), Autosomal dominant
TAS2R38	607751	[Phenylthiocarbamide tasting], 171200 (3), Autosomal dominant
TASP1	608270	Suleiman-El-Hattab syndrome, 618950 (3), Autosomal recessive
TAT	613018	Tyrosinemia, type II, 276600 (3), Autosomal recessive
TBC1D20	611663	Warburg micro syndrome 4, 615663 (3), Autosomal recessive
TBC1D23	617687	Pontocerebellar hypoplasia, type 11, 617695 (3), Autosomal recessive
TBC1D24	613577	Deafness, autosomal recessive 86, 614617 (3), Autosomal recessive; Epilepsy, rolandic, with paroxysmal exercise-induce dystonia and writer's cramp, 608105 (3), Autosomal recessive; Myoclonic epilepsy, infantile, familial, 605021 (3), Autosomal recessive; Deafness, autosomal dominant 65, 616044 (3), Autosomal dominant; Developmental and epileptic encephalopathy 16, 615338 (3), Autosomal recessive; DOORS syndrome, 220500 (3), Autosomal recessive
TBC1D2B	619152	Neurodevelopmental disorder with seizures and gingival overgrowth, 619323 (3), Autosomal recessive
TBC1D4	612465	{Diabetes mellitus, noninsulin-dependent, 5}, 616087 (3)
TBC1D7	612655	Macrocephaly/megalencephaly syndrome, autosomal recessive, 248000 (3), Autosomal recessive
TBC1D8B	301027	Nephrotic syndrome, type 20, 301028 (3), X-linked
TBCD	604649	Encephalopathy, progressive, early-onset, with brain atrophy and thin corpus callosum, 617193 (3), Autosomal recessive
TBCE	604934	Kenny-Caffey syndrome, type 1, 244460 (3), Autosomal recessive; Hypoparathyroidism-retardation-dysmorphism syndrome, 241410 (3), Autosomal recessive; Encephalopathy, progressive, with amyotrophy and optic atrophy, 617207 (3), Autosomal recessive
TBCK	616899	Hypotonia, infantile, with psychomotor retardation and characteristic facies 3, 616900 (3), Autosomal recessive
TBK1	604834	{Encephalopathy, acute, infection-induced (herpes-specific), susceptibility to, 8}, 617900 (3), Autosomal dominant; Frontotemporal dementia and/or amyotrophic lateral sclerosis 4, 616439 (3), Autosomal dominant
TBL1X	300196	Hypothyroidism, congenital, nongoitrous, 8, 301033 (3), X-linked
TBL1XR1	608628	Intellectual developmental disorder, autosomal dominant 41, 616944 (3), Autosomal dominant; Pierpont syndrome, 602342 (3), Autosomal dominant
TBL1Y	400033	?Deafness, Y-linked 2, 400047 (3), Y-linked
TBP	600075	Spinocerebellar ataxia 17, 607136 (3), Autosomal dominant; {Parkinson disease, susceptibility to}, 168600 (3), Multifactorial, Autosomal dominant
TBR1	604616	Intellectual developmental disorder with autism and speech delay, 606053 (3), Autosomal dominant

TBX1	602054	Tetralogy of Fallot, 187500 (3), Autosomal dominant; DiGeorge syndrome, 188400 (3), Autosomal dominant; Conotruncal anomaly face syndrome, 217095 (3); Velocardiofacial syndrome, 192430 (3), Autosomal dominant
TBX15	604127	Cousin syndrome, 260660 (3), Autosomal recessive
TBX18	604613	Congenital anomalies of kidney and urinary tract 2, 143400 (3), Autosomal dominant
TBX19	604614	Adrenocorticotropic hormone deficiency, 201400 (3), Autosomal recessive
TBX2	600747	Vertebral anomalies and variable endocrine and T-cell dysfunction, 618223 (3), Autosomal dominant
TBX20	606061	Atrial septal defect 4, 611363 (3)
TBX21	604895	Asthma and nasal polyps, 208550 (3), Autosomal recessive; ?Immunodeficiency 88, 619630 (3), Autosomal recessive; {Asthma, aspirin-induced, susceptibility to}, 208550 (3), Autosomal recessive
TBX22	300307	Cleft palate with ankyloglossia, 303400 (3), X-linked; ?Abruzzo-Erickson syndrome, 302905 (3), X-linked
TBX3	601621	Ulnar-mammary syndrome, 181450 (3), Autosomal dominant
TBX4	601719	Ischiocoxopodopatellar syndrome with or without pulmonary arterial hypertension, 147891 (3), Autosomal dominant; Amelia, posterior, with pelvic and pulmonary hypoplasia syndrome, 601360 (3), Autosomal recessive
TBX5	601620	Holt-Oram syndrome, 142900 (3), Autosomal dominant
TBX6	602427	Spondylocostal dysostosis 5, 122600 (3), Autosomal dominant, Autosomal recessive
TBXA2R	188070	{Bleeding disorder, platelet-type, 13, susceptibility to}, 614009 (3), Autosomal dominant
TBXAS1	274180	Ghosal hematodiaphyseal syndrome, 231095 (3), Autosomal recessive
TBXT	601397	Sacral agenesis with vertebral anomalies, 615709 (3), Autosomal recessive; {Neural tube defects, susceptibility to}, 182940 (3), Autosomal dominant
TCAP	604488	Cardiomyopathy, hypertrophic, 25, 607487 (3), Autosomal dominant; Muscular dystrophy, limb-girdle, autosomal recessive 7, 601954 (3), Autosomal recessive
TCF12	600480	Craniosynostosis 3, 615314 (3), Autosomal dominant; Hypogonadotropic hypogonadism 26 with or without anosmia, 619718 (3), Autosomal dominant, Autosomal recessive
TCF20	603107	Developmental delay with variable intellectual impairment and behavioral abnormalities, 618430 (3), Autosomal dominant
TCF3	147141	Agammaglobulinemia 8B, autosomal recessive, 619824 (3), Autosomal recessive; Agammaglobulinemia 8A, autosomal dominant, 616941 (3), Autosomal dominant
TCF4	602272	Pitt-Hopkins syndrome, 610954 (3), Autosomal dominant; Corneal dystrophy, Fuchs endothelial, 3, 613267 (3), Autosomal dominant
TCF7L2	602228	{Diabetes mellitus, type 2, susceptibility to}, 125853 (3), Autosomal dominant
TCHH	190370	?Uncombable hair syndrome 3, 617252 (3), Autosomal recessive
TCIRG1	604592	Osteopetrosis, autosomal recessive 1, 259700 (3), Autosomal recessive

TCL1A	186960	Leukemia/lymphoma, T-cell, 186960 (2)
TCL1B	603769	Leukemia/lymphoma, T-cell, 603769 (2)
TCN2	613441	Transcobalamin II deficiency, 275350 (3), Autosomal recessive
TCOF1	606847	Treacher Collins syndrome 1, 154500 (3), Autosomal dominant
TCTN1	609863	Joubert syndrome 13, 614173 (3), Autosomal recessive
TCTN2	613846	Joubert syndrome 24, 616654 (3), Autosomal recessive; ?Meckel syndrome 8, 613885 (3), Autosomal recessive
TCTN3	613847	Joubert syndrome 18, 614815 (3), Autosomal recessive; Orofaciodigital syndrome IV, 258860 (3), Autosomal recessive
TDGF1	187395	Forebrain defects (3)
TDO2	191070	[?Hypertryptophanemia], 600627 (3), Autosomal recessive
TDP1	607198	?Spinocerebellar ataxia, autosomal recessive, with axonal neuropathy 1, 607250 (3), Autosomal recessive
TDP2	605764	Spinocerebellar ataxia, autosomal recessive 23, 616949 (3), Autosomal recessive
TDRD7	611258	Cataract 36, 613887 (3), Autosomal recessive
TDRD9	617963	?Spermatogenic failure 30, 618110 (3), Autosomal recessive
TEAD1	189967	Sveinsson chorioretinal atrophy, 108985 (3), Autosomal dominant
TECPR2	615000	Neuropathy, hereditary sensory and autonomic, type IX, with developmental delay, 615031 (3), Autosomal recessive
TECR	610057	Intellectual developmental disorder, autosomal recessive 14, 614020 (3), Autosomal recessive
TECRL	617242	Ventricular tachycardia, catecholaminergic polymorphic, 3, 614021 (3), Autosomal recessive
TECTA	602574	Deafness, autosomal dominant 8/12, 601543 (3), Autosomal dominant; Deafness, autosomal recessive 21, 603629 (3), Autosomal recessive
TEK	600221	Venous malformations, multiple cutaneous and mucosal, 600195 (3), Autosomal dominant; Glaucoma 3, primary congenital, E, 617272 (3), Autosomal dominant
TELO2	611140	You-Hoover-Fong syndrome, 616954 (3), Autosomal recessive
TENM3	610083	Microphthalmia, syndromic 15, 615145 (3), Autosomal recessive; ?Microphthalmia, isolated, with coloboma 9, 615145 (3), Autosomal recessive
TENM4	610084	Essential tremor, hereditary, 5, 616736 (3), Autosomal dominant
TENT5A	611357	Osteogenesis imperfecta, type XVIII, 617952 (3), Autosomal recessive
TERB1	617332	Spermatogenic failure 60, 619646 (3), Autosomal recessive
TERB2	617131	?Spermatogenic failure 59, 619645 (3), Autosomal recessive
TERC	602322	{Pulmonary fibrosis, idiopathic, susceptibility to}, 614743 (3), Autosomal dominant; Dyskeratosis congenita, autosomal dominant 1, 127550 (3), Autosomal dominant; {Aplastic anemia}, 614743 (3), Autosomal dominant

TERT	187270	Dyskeratosis congenita, autosomal dominant 2, 613989 (3), Autosomal dominant, Autosomal recessive; Dyskeratosis congenita, autosomal recessive 4, 613989 (3), Autosomal dominant, Autosomal recessive; {Melanoma, cutaneous malignant, 9}, 615134 (3); Pulmonary fibrosis and/or bone marrow failure, telomere-related, 1, 614742 (3), Autosomal dominant; {Leukemia, acute myeloid}, 601626 (3), Autosomal dominant, Somatic mutation
TET2	612839	Myelodysplastic syndrome, somatic, 614286 (3); Immunodeficiency 75, 619126 (3), Autosomal recessive
TET3	613555	Beck-Fahrner syndrome, 618798 (3), Autosomal dominant, Autosomal recessive
TEX11	300311	Spermatogenic failure, X-linked 2, 309120 (3), X-linked recessive
TEX14	605792	Spermatogenic failure 23, 617707 (3), Autosomal recessive
TEX15	605795	Spermatogenic failure 25, 617960 (3), Autosomal recessive
TF	190000	Atransferrinemia, 209300 (3), Autosomal recessive
TFAM	600438	?Mitochondrial DNA depletion syndrome 15 (hepatocerebral type), 617156 (3), Autosomal recessive
TFAP2A	107580	Branchiooculofacial syndrome, 113620 (3), Autosomal dominant
TFAP2B	601601	Patent ductus arteriosus 2, 617035 (3), Autosomal dominant; Char syndrome, 169100 (3), Autosomal dominant
TFE3	314310	Intellectual developmental disorder, X-linked syndromic, with pigmentary mosaicism and coarse facies, 301066 (3), X-linked; Renal cell carcinoma, papillary, 1, 300854 (3)
TFG	602498	?Spastic paraplegia 57, autosomal recessive, 615658 (3), Autosomal recessive; Hereditary motor and sensory neuropathy, Okinawa type, 604484 (3), Autosomal dominant
TFR2	604720	Hemochromatosis, type 3, 604250 (3), Autosomal recessive
TFRC	190010	Immunodeficiency 46, 616740 (3), Autosomal recessive
TG	188450	{Autoimmune thyroid disease, susceptibility to, 3}, 608175 (3); Thyroid dyshormonogenesis 3, 274700 (3), Autosomal recessive
TGDS	616146	Catel-Manzke syndrome, 616145 (3), Autosomal recessive
TGFB1	190180	Inflammatory bowel disease, immunodeficiency, and encephalopathy, 618213 (3), Autosomal recessive; Camurati-Engelmann disease, 131300 (3), Autosomal dominant; {Cystic fibrosis lung disease, modifier of}, 219700 (3), Autosomal recessive
TGFB2	190220	Loeys-Dietz syndrome 4, 614816 (3), Autosomal dominant
TGFB3	190230	Arrhythmogenic right ventricular dysplasia 1, 107970 (3), Autosomal dominant; Loeys-Dietz syndrome 5, 615582 (3), Autosomal dominant

TGFBI	601692	Corneal dystrophy, Avellino type, 607541 (3), Autosomal dominant; Corneal dystrophy, Reis-Bucklers type, 608470 (3), Autosomal dominant; Corneal dystrophy, Thiel-Behnke type, 602082 (3), Autosomal dominant; Corneal dystrophy, Groenouw type I, 121900 (3), Autosomal dominant; Corneal dystrophy, epithelial basement membrane, 121820 (3), Autosomal dominant; Corneal dystrophy, lattice type I, 122200 (3), Autosomal dominant; Corneal dystrophy, lattice type IIIA, 608471 (3), Autosomal dominant
TGFBR1	190181	{Multiple self-healing squamous epithelioma, susceptibility to}, 132800 (3), Autosomal dominant; Loeys-Dietz syndrome 1, 609192 (3), Autosomal dominant
TGFBR2	190182	Loeys-Dietz syndrome 2, 610168 (3), Autosomal dominant; Colorectal cancer, hereditary nonpolyposis, type 6, 614331 (3); Esophageal cancer, somatic, 133239 (3)
TGIF1	602630	Holoprosencephaly 4, 142946 (3), Autosomal dominant
TGM1	190195	Ichthyosis, congenital, autosomal recessive 1, 242300 (3), Autosomal recessive
TGM3	600238	?Uncombable hair syndrome 2, 617251 (3), Autosomal recessive
TGM5	603805	Peeling skin syndrome 2, 609796 (3), Autosomal recessive
TGM6	613900	Spinocerebellar ataxia 35, 613908 (3), Autosomal dominant
TH	191290	Segawa syndrome, recessive, 605407 (3), Autosomal recessive
THAP1	609520	Dystonia 6, torsion, 602629 (3), Autosomal dominant
THBD	188040	Thrombophilia 12 due to thrombomodulin defect, 614486 (3); {Hemolytic uremic syndrome, atypical, susceptibility to, 6}, 612926 (3), Autosomal dominant
THBS2	188061	{Lumbar disc herniation, susceptibility to}, 603932 (3)
THG1L	618802	Spinocerebellar ataxia, autosomal recessive 28, 618800 (3), Autosomal recessive
THOC2	300395	Intellectual developmental disorder, X-linked 12, 300957 (3), X-linked recessive
THOC6	615403	Beaulieu-Boycott-Innes syndrome, 613680 (3), Autosomal recessive
THPO	600044	Thrombocythemia 1, 187950 (3), Autosomal dominant
THRA	190120	Hypothyroidism, congenital, nongoitrous, 6, 614450 (3), Autosomal dominant
THRB	190160	Thyroid hormone resistance, autosomal recessive, 274300 (3), Autosomal recessive; Thyroid hormone resistance, 188570 (3), Autosomal dominant; Thyroid hormone resistance, selective pituitary, 145650 (3), Autosomal dominant
THSD1	616821	?Aneurysm, intracranial berry, 12, 618734 (3), Autosomal dominant
THSD4	614476	Aortic aneurysm, familial thoracic 12, 619825 (3), Autosomal dominant
THUMPD1	616662	Neurodevelopmental disorder with speech delay and variable ocular anomalies, 619989 (3), Autosomal recessive

TIA1	603518	Welander distal myopathy, 604454 (3), Autosomal dominant, Autosomal recessive; Amyotrophic lateral sclerosis 26 with or without frontotemporal dementia, 619133 (3), Autosomal dominant
TIAM1	600687	Neurodevelopmental disorder with language delay and seizures, 619908 (3), Autosomal recessive
TICAM1	607601	{Encephalopathy, acute, infection-induced (herpes-specific), susceptibility to, 6}, 614850 (3), Autosomal dominant, Autosomal recessive
TIE1	600222	Lymphatic malformation 11, 619401 (3), Autosomal dominant
TIMELESS	603887	Advance sleep phase syndrome, familial, 4, 620015 (3)
TIMM22	607251	?Combined oxidative phosphorylation deficiency 43, 618851 (3), Autosomal recessive
TIMM50	607381	3-methylglutaconic aciduria, type IX, 617698 (3), Autosomal recessive
TIMM8A	300356	Mohr-Tranebjaerg syndrome, 304700 (3), X-linked recessive
TIMMDC1	615534	Mitochondrial complex I deficiency, nuclear type 31, 618251 (3), Autosomal recessive
TIMP3	188826	Sorsby fundus dystrophy, 136900 (3), Autosomal dominant
TINF2	604319	Dyskeratosis congenita, autosomal dominant 3, 613990 (3), Autosomal dominant; Revesz syndrome, 268130 (3), Autosomal dominant
TIRAP	606252	{Malaria, protection against}, 611162 (3); {Tuberculosis, protection against}, 607948 (3); {Bacteremia, protection against}, 614382 (3)
TJP2	607709	Hypercholanemia, familial 1, 607748 (3), Autosomal recessive; Cholestasis, progressive familial intrahepatic 4, 615878 (3), Autosomal recessive
TK2	188250	Mitochondrial DNA depletion syndrome 2 (myopathic type), 609560 (3), Autosomal recessive; ?Progressive external ophthalmoplegia with mitochondrial DNA deletions, autosomal recessive 3, 617069 (3), Autosomal recessive
TKFC	615844	Triokinase and FMN cyclase deficiency syndrome, 618805 (3), Autosomal recessive
TKT	606781	Short stature, developmental delay, and congenital heart defects, 617044 (3), Autosomal recessive
TLCD3B	615175	Cone-rod dystrophy 22, 619531 (3), Autosomal recessive
TLE6	612399	Preimplantation embryonic lethality, 616814 (3), Autosomal recessive
TLK2	608439	Intellectual developmental disorder, autosomal dominant 57, 618050 (3), Autosomal dominant
TLL1	606742	Atrial septal defect 6, 613087 (3), Autosomal dominant
TLR1	601194	{Leprosy, susceptibility to, 5}, 613223 (3); {Leprosy, protection against}, 613223 (3)
TLR2	603028	{Colorectal cancer, susceptibility to}, 114500 (3), Autosomal dominant, Somatic mutation; {Leprosy, susceptibility to}, 246300 (3), Autosomal dominant; {Mycobacterium tuberculosis, susceptibility to}, 607948 (3)
TLR3	603029	{HIV1 infection, resistance to}, 609423 (3); {Immunodeficiency 83, susceptibility to viral infections}, 613002 (3), Autosomal dominant, Autosomal recessive
TLR4	603030	Macular degeneration, age-related, 10, 611488 (2)

TLR5	603031	{Melioidosis, susceptibility to}, 615557 (3), Autosomal dominant; {Systemic lupus erythematosus, susceptibility to, 1}, 601744 (3); {Systemic lupus erythematosus, resistance to}, 601744 (3); {Legionnaire disease, susceptibility to}, 608556 (3)
TLR7	300365	Immunodeficiency 74, COVID19-related, X-linked, 301051 (3), X-linked recessive; Systemic lupus erythematosus 17, 301080 (3), X-linked dominant
TLR8	300366	Immunodeficiency 98 with autoinflammation, X-linked, 301078 (3), Somatic mosaicism, X-linked
TM4SF20	615404	{Specific language impairment 5}, 615432 (3), Autosomal dominant
TMC1	606706	Deafness, autosomal dominant 36, 606705 (3), Autosomal dominant; Deafness, autosomal recessive 7, 600974 (3), Autosomal recessive
TMC6	605828	Epidermodysplasia verruciformis, 226400 (3), Autosomal recessive
TMC8	605829	Epidermodysplasia verruciformis 2, 618231 (3), Autosomal recessive
TMCO1	614123	Craniofacial dysmorphism, skeletal anomalies, and impaired intellectual development 1, 213980 (3), Autosomal recessive
TMEM106B	613413	Leukodystrophy, hypomyelinating, 16, 617964 (3), Autosomal dominant
TMEM107	616183	Orofaciodigital syndrome XVI, 617563 (3), Autosomal recessive; Meckel syndrome 13, 617562 (3), Autosomal recessive; ?Joubert syndrome 29, 617562 (3), Autosomal recessive
TMEM126A	612988	Optic atrophy 7, 612989 (3), Autosomal recessive
TMEM126B	615533	Mitochondrial complex I deficiency, nuclear type 29, 618250 (3), Autosomal recessive
TMEM127	613403	{Pheochromocytoma, susceptibility to}, 171300 (3), Autosomal dominant
TMEM132E	616178	Deafness, autosomal recessive 99, 618481 (3), Autosomal recessive
TMEM138	614459	Joubert syndrome 16, 614465 (3), Autosomal recessive
TMEM165	614726	Congenital disorder of glycosylation, type IIk, 614727 (3), Autosomal recessive
TMEM199	616815	Congenital disorder of glycosylation, type IIp, 616829 (3), Autosomal recessive
TMEM216	613277	Joubert syndrome 2, 608091 (3), Autosomal recessive; Meckel syndrome 2, 603194 (3), Autosomal recessive
TMEM218	619285	Joubert syndrome 39, 619562 (3), Autosomal recessive
TMEM222	619469	Neurodevelopmental disorder with motor and speech delay and behavioral abnormalities, 619470 (3), Autosomal recessive
TMEM231	614949	Joubert syndrome 20, 614970 (3), Autosomal recessive; Meckel syndrome 11, 615397 (3), Autosomal recessive
TMEM237	614423	Joubert syndrome 14, 614424 (3), Autosomal recessive
TMEM240	616101	Spinocerebellar ataxia 21, 607454 (3), Autosomal dominant
TMEM251	619332	Dysostosis multiplex, Ain-Naz type, 619345 (3), Autosomal recessive
TMEM260	617449	Structural heart defects and renal anomalies syndrome, 617478 (3), Autosomal recessive
TMEM38B	611236	Osteogenesis imperfecta, type XIV, 615066 (3), Autosomal recessive

TMEM43	612048	Arrhythmogenic right ventricular dysplasia 5, 604400 (3), Autosomal dominant; Auditory neuropathy, autosomal dominant 3, 619832 (3), Autosomal dominant; Emery-Dreifuss muscular dystrophy 7, AD, 614302 (3), Autosomal dominant
TMEM53	619722	Craniotubular dysplasia, Ikegawa type, 619727 (3), Autosomal recessive
TMEM63A	618685	Leukodystrophy, hypomyelinating, 19, transient infantile, 618688 (3), Autosomal dominant
TMEM63C	619953	Spastic paraplegia 87, autosomal recessive, 619966 (3), Autosomal recessive
TMEM67	609884	Nephronophthisis 11, 613550 (3), Autosomal recessive; {Bardet-Biedl syndrome 14, modifier of}, 615991 (3), Autosomal recessive; Joubert syndrome 6, 610688 (3), Autosomal recessive; Meckel syndrome 3, 607361 (3), Autosomal recessive; ?RHYNS syndrome, 602152 (3), Autosomal recessive; COACH syndrome 1, 216360 (3), Autosomal recessive
TMEM70	612418	Mitochondrial complex V (ATP synthase) deficiency, nuclear type 2, 614052 (3), Autosomal recessive
TMEM94	618163	Intellectual developmental disorder with cardiac defects and dysmorphic facies, 618316 (3), Autosomal recessive
TMEM98	615949	Nanophthalmos 4, 615972 (3), Autosomal dominant
TMIE	607237	Deafness, autosomal recessive 6, 600971 (3), Autosomal recessive
TMLHE	300777	{Autism, susceptibility to, X-linked 6}, 300872 (3), X-linked recessive
TMPRSS15	606635	Enterokinase deficiency, 226200 (3), Autosomal recessive
TMPRSS3	605511	Deafness, autosomal recessive 8/10, 601072 (3), Autosomal recessive
TMPRSS6	609862	Iron-refractory iron deficiency anemia, 206200 (3), Autosomal recessive
TMTC3	617218	Lissencephaly 8, 617255 (3), Autosomal recessive
TMX2	616715	Neurodevelopmental disorder with microcephaly, cortical malformations, and spasticity, 618730 (3), Autosomal recessive
TNC	187380	Deafness, autosomal dominant 56, 615629 (3), Autosomal dominant
TNF	191160	{Migraine without aura, susceptibility to}, 157300 (3), Autosomal dominant; {Dementia, vascular, susceptibility to} (3); {Asthma, susceptibility to}, 600807 (3), Autosomal dominant; {Septic shock, susceptibility to} (3); {Malaria, cerebral, susceptibility to}, 611162 (3)
TNFAIP3	191163	Autoinflammatory syndrome, familial, Behcet-like 1, 616744 (3), Autosomal dominant
TNFRSF10B	603612	Squamous cell carcinoma, head and neck, 275355 (3), Autosomal recessive
TNFRSF11A	603499	Osteopetrosis, autosomal recessive 7, 612301 (3), Autosomal recessive; {Paget disease of bone 2, early-onset}, 602080 (3), Autosomal dominant; Osteolysis, familial expansile, 174810 (3), Autosomal dominant
TNFRSF11B	602643	Paget disease of bone 5, juvenile-onset, 239000 (3), Autosomal recessive
TNFRSF13B	604907	Immunodeficiency, common variable, 2, 240500 (3), Autosomal dominant, Autosomal recessive; Immunoglobulin A deficiency 2, 609529 (3)
TNFRSF13C	606269	Immunodeficiency, common variable, 4, 613494 (3), Autosomal recessive
TNFRSF1A	191190	{Multiple sclerosis, susceptibility to, 5}, 614810 (3); Periodic fever, familial, 142680 (3), Autosomal dominant

TNFRSF4	600315	?Immunodeficiency 16, 615593 (3), Autosomal recessive
TNFSF11	602642	Osteopetrosis, autosomal recessive 2, 259710 (3), Autosomal recessive
TNFSF4	603594	{Myocardial infarction, susceptibility to}, 608446 (3)
TNIK	610005	Intellectual developmental disorder, autosomal recessive 54, 617028 (3), Autosomal recessive
TNNC1	191040	Cardiomyopathy, dilated, 1Z, 611879 (3); Cardiomyopathy, hypertrophic, 13, 613243 (3), Autosomal dominant
TNNI2	191043	Arthrogryposis, distal, type 2B1, 601680 (3), Autosomal dominant
TNNI3	191044	?Cardiomyopathy, dilated, 2A, 611880 (3), Autosomal recessive; Cardiomyopathy, hypertrophic, 7, 613690 (3), Autosomal dominant; Cardiomyopathy, familial restrictive, 1, 115210 (3), Autosomal dominant; Cardiomyopathy, dilated, 1FF, 613286 (3)
TNNI3K	613932	Cardiac conduction disease with or without dilated cardiomyopathy, 616117 (3), Autosomal dominant
TNNT1	191041	Nemaline myopathy 5, Amish type, 605355 (3), Autosomal recessive
TNNT2	191045	Cardiomyopathy, dilated, 1D, 601494 (3), Autosomal dominant; Cardiomyopathy, hypertrophic, 2, 115195 (3), Autosomal dominant; Cardiomyopathy, familial restrictive, 3, 612422 (3), Autosomal dominant; Left ventricular noncompaction 6, 601494 (3), Autosomal dominant
TNNT3	600692	Arthrogryposis, distal, type 2B2, 618435 (3), Autosomal dominant
TNPO2	603002	Intellectual developmental disorder with hypotonia, impaired speech, and dysmorphic facies, 619556 (3), Autosomal dominant
TNPO3	610032	Muscular dystrophy, limb-girdle, autosomal dominant 2, 608423 (3), Autosomal dominant
TNR	601995	Neurodevelopmental disorder, nonprogressive, with spasticity and transient opisthotonus, 619653 (3), Autosomal recessive
TNRC6A	610739	?Epilepsy, familial adult myoclonic, 6, 618074 (3), Autosomal dominant
TNRC6B	610740	Global developmental delay with speech and behavioral abnormalities, 619243 (3), Autosomal dominant
TNXB	600985	Ehlers-Danlos syndrome, classic-like, 1, 606408 (3), Autosomal recessive; Vesicoureteral reflux 8, 615963 (3), Autosomal dominant
TOE1	613931	Pontocerebellar hypoplasia, type 7, 614969 (3), Autosomal recessive
TOGARAM1	617618	Joubert syndrome 37, 619185 (3), Autosomal recessive
TOM1	604700	?Immunodeficiency 85 and autoimmunity, 619510 (3), Autosomal dominant
TONSL	604546	Spondyloepimetaphyseal dysplasia, sponastrime type, 271510 (3), Autosomal recessive
TOP1	126420	DNA topoisomerase I, camptothecin-resistant (3)
TOP2A	126430	DNA topoisomerase II, resistance to inhibition of, by amsacrine (3)
TOP2B	126431	B-cell immunodeficiency, distal limb anomalies, and urogenital malformations, 609296 (3), Autosomal dominant

TOP3A	601243	?Progressive external ophthalmoplegia with mitochondrial DNA deletions, autosomal recessive 5, 618098 (3), Autosomal recessive; Microcephaly, growth restriction, and increased sister chromatid exchange 2, 618097 (3), Autosomal recessive
TOPORS	609507	Retinitis pigmentosa 31, 609923 (3)
TOR1A	605204	{Dystonia-1, modifier of} (3); Arthrogryposis multiplex congenita 5, 618947 (3), Autosomal recessive; Dystonia-1, torsion, 128100 (3), Autosomal dominant
TOR1AIP1	614512	?Muscular dystrophy, autosomal recessive, with rigid spine and distal joint contractures, 617072 (3), Autosomal recessive
TP53	191170	{Basal cell carcinoma 7}, 614740 (3), Autosomal dominant; {Adrenocortical carcinoma, pediatric}, 202300 (3), Autosomal dominant; Hepatocellular carcinoma, somatic, 114550 (3); Breast cancer, somatic, 114480 (3); Li-Fraumeni syndrome, 151623 (3), Autosomal dominant; Pancreatic cancer, somatic, 260350 (3); Nasopharyngeal carcinoma, somatic, 607107 (3); {Osteosarcoma}, 259500 (3), Somatic mutation; {Choroid plexus papilloma}, 260500 (3), Autosomal dominant; {Colorectal cancer}, 114500 (3), Autosomal dominant, Somatic mutation; {Glioma susceptibility 1}, 137800 (3), Autosomal dominant, Somatic mutation; Bone marrow failure syndrome 5, 618165 (3), Autosomal dominant
TP53RK	608679	Galloway-Mowat syndrome 4, 617730 (3), Autosomal recessive
TP63	603273	Ectrodactyly, ectodermal dysplasia, and cleft lip/palate syndrome 3, 604292 (3), Autosomal dominant; Hay-Wells syndrome, 106260 (3), Autosomal dominant; Split-hand/foot malformation 4, 605289 (3), Autosomal dominant; Orofacial cleft 8, 618149 (3); Rapp-Hodgkin syndrome, 129400 (3), Autosomal dominant; ADULT syndrome, 103285 (3), Autosomal dominant; Limb-mammary syndrome, 603543 (3), Autosomal dominant
TP73	601990	Ciliary dyskinesia, primary, 47, and lissencephaly, 619466 (3), Autosomal recessive
TPCN2	612163	[Skin/hair/eye pigmentation 10, blond/brown hair], 612267 (3)
TPH2	607478	{?Attention deficit-hyperactivity disorder, susceptibility to, 7}, 613003 (3); {Unipolar depression, susceptibility to}, 608516 (3)
TPI1	190450	Hemolytic anemia due to triosephosphate isomerase deficiency, 615512 (3), Autosomal recessive
TPK1	606370	Thiamine metabolism dysfunction syndrome 5 (episodic encephalopathy type), 614458 (3), Autosomal recessive
TPM1	191010	Left ventricular noncompaction 9, 611878 (3), Autosomal dominant; Cardiomyopathy, hypertrophic, 3, 115196 (3), Autosomal dominant; Cardiomyopathy, dilated, 1Y, 611878 (3), Autosomal dominant
TPM2	190990	Arthrogryposis, distal, type 2B4, 108120 (3), Autosomal dominant; Arthrogryposis, distal, type 1A, 108120 (3), Autosomal dominant; Nemaline myopathy 4, autosomal dominant, 609285 (3), Autosomal dominant; CAP myopathy 2, 609285 (3), Autosomal dominant

TPM3	191030	CAP myopathy 1, 609284 (3), Autosomal dominant, Autosomal recessive; Myopathy, congenital, with fiber-type disproportion, 255310 (3), Autosomal dominant, Autosomal recessive; Nemaline myopathy 1, autosomal dominant or recessive, 609284 (3), Autosomal dominant, Autosomal recessive
TPMT	187680	{Thiopurines, poor metabolism of, 1}, 610460 (3), Autosomal recessive
TPO	606765	Thyroid dyshormonogenesis 2A, 274500 (3), Autosomal recessive
TPP1	607998	Ceroid lipofuscinosis, neuronal, 2, 204500 (3), Autosomal recessive; Spinocerebellar ataxia, autosomal recessive 7, 609270 (3), Autosomal recessive
TPP2	190470	Immunodeficiency 78 with autoimmunity and developmental delay, 619220 (3), Autosomal recessive
TPRKB	608680	Galloway-Mowat syndrome 5, 617731 (3), Autosomal recessive
TPRN	613354	Deafness, autosomal recessive 79, 613307 (3), Autosomal recessive
TRAC	186880	Immunodeficiency 7, TCR-alpha/beta deficient, 615387 (3), Autosomal recessive
TRAF3	601896	{?Encephalopathy, acute, infection-induced (herpes-specific), susceptibility to, 5}, 614849 (3)
TRAF3IP1	607380	Senior-Loken syndrome 9, 616629 (3), Autosomal recessive
TRAF3IP2	607043	?Candidiasis, familial, 8, 615527 (3), Autosomal recessive; {Psoriasis susceptibility 13}, 614070 (3)
TRAF7	606692	Cardiac, facial, and digital anomalies with developmental delay, 618164 (3), Autosomal dominant
TRAIP	605958	Seckel syndrome 9, 616777 (3), Autosomal recessive
TRAK1	608112	Developmental and epileptic encephalopathy 68, 618201 (3), Autosomal recessive
TRAPPC11	614138	Muscular dystrophy, limb-girdle, autosomal recessive 18, 615356 (3), Autosomal recessive
TRAPPC12	614139	Encephalopathy, progressive, early-onset, with brain atrophy and spasticity, 617669 (3), Autosomal recessive
TRAPPC14	618350	?Microcephaly 25, primary, autosomal recessive, 618351 (3), Autosomal recessive
TRAPPC2	300202	Spondyloepiphyseal dysplasia tarda, 313400 (3), X-linked recessive
TRAPPC2L	610970	Encephalopathy, progressive, early-onset, with episodic rhabdomyolysis, 618331 (3), Autosomal recessive
TRAPPC4	610971	Neurodevelopmental disorder with epilepsy, spasticity, and brain atrophy, 618741 (3), Autosomal recessive
TRAPPC6B	610397	Neurodevelopmental disorder with microcephaly, epilepsy, and brain atrophy, 617862 (3), Autosomal recessive
TRAPPC9	611966	Intellectual developmental disorder, autosomal recessive 13, 613192 (3), Autosomal recessive
TRDN	603283	Cardiac arrhythmia syndrome, with or without skeletal muscle weakness, 615441 (3), Autosomal recessive
TREH	275360	Trehalase deficiency, 612119 (3), Autosomal recessive

TREM2	605086	Polycystic lipomembranous osteodysplasia with sclerosing leukoencephalopathy 2, 618193 (3), Autosomal recessive
TREX1	606609	Vasculopathy, retinal, with cerebral leukoencephalopathy and systemic manifestations, 192315 (3), Autosomal dominant; Aicardi-Goutieres syndrome 1, dominant and recessive, 225750 (3), Autosomal dominant, Autosomal recessive; {Systemic lupus erythematosus, susceptibility to}, 152700 (3), Autosomal dominant; Chilblain lupus, 610448 (3), Autosomal dominant
TRH	613879	Thyrotropin-releasing hormone deficiency, 275120 (1), Autosomal recessive
TRHR	188545	Hypothyroidism, congenital, nongoitrous, 7, 618573 (3), Autosomal recessive
TRIM2	614141	Charcot-Marie-Tooth disease, type 2R, 615490 (3), Autosomal recessive
TRIM32	602290	?Bardet-Biedl syndrome 11, 615988 (3), Autosomal recessive; Muscular dystrophy, limb-girdle, autosomal recessive 8, 254110 (3), Autosomal recessive
TRIM36	609317	?Anencephaly 1, 206500 (3), Autosomal recessive
TRIM37	605073	Mulibrey nanism, 253250 (3), Autosomal recessive
TRIM44	612298	?Aniridia 3, 617142 (3), Autosomal dominant
TRIM71	618570	Hydrocephalus, congenital communicating, 1, 618667 (3), Autosomal dominant
TRIM8	606125	Focal segmental glomerulosclerosis and neurodevelopmental syndrome, 619428 (3), Autosomal dominant
TRIO	601893	Intellectual developmental disorder, autosomal dominant 44, with microcephaly, 617061 (3), Autosomal dominant; Intellectual developmental disorder, autosomal dominant 63, with macrocephaly, 618825 (3), Autosomal dominant
TRIOBP	609761	Deafness, autosomal recessive 28, 609823 (3), Autosomal recessive
TRIP11	604505	Odontochondrodysplasia 1, 184260 (3), Autosomal recessive; Achondrogenesis, type IA, 200600 (3), Autosomal recessive
TRIP12	604506	Intellectual developmental disorder, autosomal dominant 49, 617752 (3), Autosomal dominant
TRIP13	604507	Oocyte maturation defect 9, 619011 (3), Autosomal recessive; Mosaic variegated aneuploidy syndrome 3, 617598 (3), Autosomal recessive
TRIP4	604501	?Muscular dystrophy, congenital, Davignon-Chauveau type, 617066 (3), Autosomal recessive; Spinal muscular atrophy with congenital bone fractures 1, 616866 (3), Autosomal recessive
TRIT1	617840	Combined oxidative phosphorylation deficiency 35, 617873 (3), Autosomal recessive
TRMT1	611669	Intellectual developmental disorder, autosomal recessive 68, 618302 (3), Autosomal recessive
TRMT10A	616013	Microcephaly, short stature, and impaired glucose metabolism 1, 616033 (3), Autosomal recessive

TRMT10C	615423	Combined oxidative phosphorylation deficiency 30, 616974 (3), Autosomal recessive
TRMT5	611023	Peripheral neuropathy with variable spasticity, exercise intolerance, and developmental delay, 616539 (3), Autosomal recessive
TRMU	610230	{Deafness, mitochondrial, modifier of}, 580000 (3), Mitochondrial; Liver failure, transient infantile, 613070 (3), Autosomal recessive
TRNT1	612907	Sideroblastic anemia with B-cell immunodeficiency, periodic fevers, and developmental delay, 616084 (3), Autosomal recessive; Retinitis pigmentosa and erythrocytic microcytosis, 616959 (3), Autosomal recessive
TRPA1	604775	?Episodic pain syndrome, familial, 1, 615040 (3), Autosomal dominant
TRPC3	602345	?Spinocerebellar ataxia 41, 616410 (3), Autosomal dominant
TRPC6	603652	Glomerulosclerosis, focal segmental, 2, 603965 (3), Autosomal dominant
TRPM1	603576	Night blindness, congenital stationary (complete), 1C, autosomal recessive, 613216 (3)
TRPM4	606936	Progressive familial heart block, type IB, 604559 (3), Autosomal dominant; Erythrokeratodermia variabilis et progressiva 6, 618531 (3), Autosomal dominant
TRPM6	607009	Hypomagnesemia 1, intestinal, 602014 (3), Autosomal recessive
TRPM7	605692	{Amyotrophic lateral sclerosis-parkinsonism/dementia complex, susceptibility to}, 105500 (3), Autosomal dominant
TRPS1	604386	Trichorhinophalangeal syndrome, type III, 190351 (3), Autosomal dominant; Trichorhinophalangeal syndrome, type I, 190350 (3), Autosomal dominant
TRPV3	607066	?Palmoplantar keratoderma, nonepidermolytic, focal 2, 616400 (3), Autosomal dominant; Olmsted syndrome 1, 614594 (3), Autosomal dominant
TRPV4	605427	Spondylometaphyseal dysplasia, Kozlowski type, 184252 (3), Autosomal dominant; Digital arthropathy-brachydactyly, familial, 606835 (3), Autosomal dominant; [Sodium serum level QTL 1], 613508 (3); SED, Maroteaux type, 184095 (3), Autosomal dominant; Metatropic dysplasia, 156530 (3), Autosomal dominant; Scapuloperoneal spinal muscular atrophy, 181405 (3), Autosomal dominant; Hereditary motor and sensory neuropathy, type IIc, 606071 (3), Autosomal dominant; ?Avascular necrosis of femoral head, primary, 2, 617383 (3), Autosomal dominant; Neuronopathy, distal hereditary motor, type VIII, 600175 (3), Autosomal dominant; Parastremmatic dwarfism, 168400 (3), Autosomal dominant; Brachyolmia type 3, 113500 (3), Autosomal dominant
TRPV6	606680	Hyperparathyroidism, transient neonatal, 618188 (3), Autosomal recessive
TRRAP	603015	?Deafness, autosomal dominant 75, 618778 (3), Autosomal dominant; Developmental delay with or without dysmorphic facies and autism, 618454 (3), Autosomal dominant
TSC1	605284	Focal cortical dysplasia, type II, somatic, 607341 (3); Tuberous sclerosis-1, 191100 (3), Autosomal dominant; Lymphangioleiomyomatosis, 606690 (3)

TSC2	191092	Lymphangioleiomyomatosis, somatic, 606690 (3); ?Focal cortical dysplasia, type II, somatic, 607341 (3); Tuberous sclerosis-2, 613254 (3), Autosomal dominant
TSEN15	608756	Pontocerebellar hypoplasia, type 2F, 617026 (3), Autosomal recessive
TSEN2	608753	Pontocerebellar hypoplasia type 2B, 612389 (3), Autosomal recessive
TSEN34	608754	?Pontocerebellar hypoplasia type 2C, 612390 (3), Autosomal recessive
TSEN54	608755	Pontocerebellar hypoplasia type 2A, 277470 (3), Autosomal recessive; Pontocerebellar hypoplasia type 4, 225753 (3), Autosomal recessive; ?Pontocerebellar hypoplasia type 5, 610204 (3), Autosomal recessive
TSFM	604723	Combined oxidative phosphorylation deficiency 3, 610505 (3), Autosomal recessive
TSGA10	607166	?Spermatogenic failure 26, 617961 (3), Autosomal recessive
TSHB	188540	Hypothyroidism, congenital, nongoitrous 4, 275100 (3), Autosomal recessive
TSHR	603372	Hyperthyroidism, familial gestational, 603373 (3), Autosomal dominant; Hyperthyroidism, nonautoimmune, 609152 (3), Autosomal dominant; Thyroid adenoma, hyperfunctioning, somatic (3); Hypothyroidism, congenital, nongoitrous, 1, 275200 (3), Autosomal recessive; Thyroid carcinoma with thyrotoxicosis, somatic (3)
TSHZ1	614427	Aural atresia, congenital, 607842 (3), Autosomal dominant
TSPAN12	613138	Exudative vitreoretinopathy 5, 613310 (3), Autosomal dominant
TSPAN7	300096	Intellectual developmental disorder, X-linked 58, 300210 (3), X-linked recessive
TSPEAR	612920	?Deafness, autosomal recessive 98, 614861 (3), Autosomal recessive; Ectodermal dysplasia 14, hair/tooth type with or without hypohidrosis, 618180 (3), Autosomal recessive
TSPYL1	604714	Sudden infant death with dysgenesis of the testes syndrome, 608800 (3), Autosomal recessive
TSR2	300945	?Diamond-Blackfan anemia 14 with mandibulofacial dysostosis, 300946 (3), X-linked recessive
TTBK2	611695	Spinocerebellar ataxia 11, 604432 (3), Autosomal dominant
TTC12	610732	Ciliary dyskinesia, primary, 45, 618801 (3), Autosomal recessive
TTC19	613814	Mitochondrial complex III deficiency, nuclear type 2, 615157 (3), Autosomal recessive
TTC21A	611430	Spermatogenic failure 37, 618429 (3), Autosomal recessive
TTC21B	612014	Short-rib thoracic dysplasia 4 with or without polydactyly, 613819 (3), Autosomal recessive; Nephronophthisis 12, 613820 (3), Autosomal dominant, Autosomal recessive
TTC26	617453	Biliary, renal, neurologic, and skeletal syndrome, 619534 (3), Autosomal recessive
TTC29	618735	Spermatogenic failure 42, 618745 (3), Autosomal recessive
TTC5	619014	Neurodevelopmental disorder with cerebral atrophy and variable facial dysmorphism, 619244 (3), Autosomal recessive

TTC7A	609332	Gastrointestinal defects and immunodeficiency syndrome, 243150 (3), Autosomal recessive
TTC8	608132	Bardet-Biedl syndrome 8, 615985 (3), Autosomal recessive; ?Retinitis pigmentosa 51, 613464 (3), Autosomal recessive
TTI2	614426	Intellectual developmental disorder, autosomal recessive 39, 615541 (3), Autosomal recessive
TTLL5	612268	Cone-rod dystrophy 19, 615860 (3), Autosomal recessive
TTN	188840	Muscular dystrophy, limb-girdle, autosomal recessive 10, 608807 (3), Autosomal recessive; Cardiomyopathy, familial hypertrophic, 9, 613765 (3), Autosomal dominant; Tibial muscular dystrophy, tardive, 600334 (3), Autosomal dominant; Salih myopathy, 611705 (3), Autosomal recessive; Cardiomyopathy, dilated, 1G, 604145 (3), Autosomal dominant; Myopathy, myofibrillar, 9, with early respiratory failure, 603689 (3), Autosomal dominant
TTPA	600415	Ataxia with isolated vitamin E deficiency, 277460 (3), Autosomal recessive
TTR	176300	Amyloidosis, hereditary, transthyretin-related, 105210 (3), Autosomal dominant; Carpal tunnel syndrome, familial, 115430 (3), Autosomal dominant; [Dystransthyretinemic hyperthyroxinemia], 145680 (3), Autosomal dominant
TUB	601197	?Retinal dystrophy and obesity, 616188 (3), Autosomal recessive
TUBA1A	602529	Lissencephaly 3, 611603 (3), Autosomal dominant
TUBA3D	617878	Keratoconus 9, 617928 (3), Autosomal dominant
TUBA4A	191110	Amyotrophic lateral sclerosis 22 with or without frontotemporal dementia, 616208 (3), Autosomal dominant
TUBA8	605742	Macrothrombocytopenia, isolated, 2, autosomal dominant, 619840 (3), Autosomal dominant
TUBB	191130	Symmetric circumferential skin creases, congenital, 1, 156610 (3), Autosomal dominant; Cortical dysplasia, complex, with other brain malformations 6, 615771 (3), Autosomal dominant
TUBB1	612901	Macrothrombocytopenia, isolated, 1, autosomal dominant, 613112 (3), Autosomal dominant
TUBB2A	615101	Cortical dysplasia, complex, with other brain malformations 5, 615763 (3), Autosomal dominant
TUBB2B	612850	Cortical dysplasia, complex, with other brain malformations 7, 610031 (3), Autosomal dominant
TUBB3	602661	Fibrosis of extraocular muscles, congenital, 3A, 600638 (3), Autosomal dominant; Cortical dysplasia, complex, with other brain malformations 1, 614039 (3), Autosomal dominant
TUBB4A	602662	Dystonia 4, torsion, autosomal dominant, 128101 (3), Autosomal dominant; Leukodystrophy, hypomyelinating, 6, 612438 (3), Autosomal dominant
TUBB4B	602660	Leber congenital amaurosis with early-onset deafness, 617879 (3), Autosomal dominant

TUBB6	615103	?Facial palsy, congenital, with ptosis and velopharyngeal dysfunction, 617732 (3), Autosomal dominant
TUBB8	616768	Oocyte maturation defect 2, 616780 (3), Autosomal dominant, Autosomal recessive
TUBG1	191135	Cortical dysplasia, complex, with other brain malformations 4, 615412 (3), Autosomal dominant
TUBGCP2	617817	Pachygyria, microcephaly, developmental delay, and dysmorphic facies, with or without seizures, 618737 (3), Autosomal recessive
TUBGCP4	609610	Microcephaly and chorioretinopathy, autosomal recessive, 3, 616335 (3), Autosomal recessive
TUBGCP6	610053	Microcephaly and chorioretinopathy, autosomal recessive, 1, 251270 (3), Autosomal recessive
TUFM	602389	Combined oxidative phosphorylation deficiency 4, 610678 (3), Autosomal recessive
TULP1	602280	Leber congenital amaurosis 15, 613843 (3), Autosomal recessive; Retinitis pigmentosa 14, 600132 (3), Autosomal recessive
TULP3	604730	Hepatorenocardiac degenerative fibrosis, 619902 (3), Autosomal recessive
TUSC3	601385	Intellectual developmental disorder, autosomal recessive 7, 611093 (3), Autosomal recessive
TWIST1	601622	Craniosynostosis 1, 123100 (3), Autosomal dominant; Robinow-Sorauf syndrome, 180750 (3), Autosomal dominant; Sweeney-Cox syndrome, 617746 (3), Autosomal dominant; Saethre-Chotzen syndrome with or without eyelid anomalies, 101400 (3), Autosomal dominant
TWIST2	607556	Ablepharon-macrostomia syndrome, 200110 (3), Autosomal dominant; Barber-Say syndrome, 209885 (3), Autosomal dominant; Focal facial dermal dysplasia 3, Setleis type, 227260 (3), Autosomal recessive
TWNK	606075	Mitochondrial DNA depletion syndrome 7 (hepatocerebral type), 271245 (3), Autosomal recessive; Progressive external ophthalmoplegia with mitochondrial DNA deletions, autosomal dominant 3, 609286 (3), Autosomal dominant; Perrault syndrome 5, 616138 (3), Autosomal recessive
TXN2	609063	?Combined oxidative phosphorylation deficiency 29, 616811 (3), Autosomal recessive
TXNDC15	617778	Meckel syndrome 14, 619879 (3), Autosomal recessive
TXNL4A	611595	Burn-McKeown syndrome, 608572 (3), Autosomal recessive
TXNRD2	606448	?Glucocorticoid deficiency 5, 617825 (3), Autosomal recessive
TYK2	176941	Immunodeficiency 35, 611521 (3), Autosomal recessive
TYMP	131222	Mitochondrial DNA depletion syndrome 1 (MNGIE type), 603041 (3), Autosomal recessive

TYR	606933	[Skin/hair/eye pigmentation 3, light/dark/freckling skin], 601800 (3), Autosomal dominant; [Skin/hair/eye pigmentation 3, blue/green eyes], 601800 (3), Autosomal dominant; {Melanoma, cutaneous malignant, susceptibility to, 8}, 601800 (3), Autosomal dominant; Albinism, oculocutaneous, type IB, 606952 (3), Autosomal recessive; Waardenburg syndrome/albinism, digenic, 103470 (3); Albinism, oculocutaneous, type IA, 203100 (3), Autosomal recessive
TYROBP	604142	Polycystic lipomembranous osteodysplasia with sclerosing leukoencephalopathy 1, 221770 (3), Autosomal recessive
TYRP1	115501	[Skin/hair/eye pigmentation, variation in, 11 (Melanesian blond hair)], 612271 (3); Albinism, oculocutaneous, type III, 203290 (3), Autosomal recessive
UBA1	314370	Spinal muscular atrophy, X-linked 2, infantile, 301830 (3), X-linked recessive; VEXAS syndrome, somatic, 301054 (3)
UBA2	613295	ACCES syndrome, 619959 (3), Autosomal dominant
UBA5	610552	?Spinocerebellar ataxia, autosomal recessive 24, 617133 (3), Autosomal recessive; Developmental and epileptic encephalopathy 44, 617132 (3), Autosomal recessive
UBAP1	609787	Spastic paraplegia 80, autosomal dominant, 618418 (3), Autosomal dominant
UBE2A	312180	Intellectual developmental disorder, X-linked syndromic, Nascimento type, 300860 (3), X-linked recessive
UBE2T	610538	Fanconi anemia, complementation group T, 616435 (3), Autosomal recessive
UBE3A	601623	Angelman syndrome, 105830 (3), Autosomal dominant
UBE3B	608047	Kaufman oculocerebrofacial syndrome, 244450 (3), Autosomal recessive
UBE4A	603753	Neurodevelopmental disorder with hypotonia and gross motor and seech delay, 619639 (3), Autosomal recessive
UBIAD1	611632	Corneal dystrophy, Schnyder type, 121800 (3), Autosomal dominant
UBQLN2	300264	Amyotrophic lateral sclerosis 15, with or without frontotemporal dementia, 300857 (3), X-linked dominant
UBR1	605981	Johanson-Blizzard syndrome, 243800 (3), Autosomal recessive
UBR7	613816	Li-Campeau syndrome, 619189 (3), Autosomal recessive
UBTF	600673	Neurodegeneration, childhood-onset, with brain atrophy, 617672 (3), Autosomal dominant
UCHL1	191342	Spastic paraplegia 79, autosomal recessive, 615491 (3), Autosomal recessive; {?Parkinson disease 5, susceptibility to}, 613643 (3), Autosomal dominant
UCP2	601693	{Obesity, susceptibility to, BMIQ4}, 607447 (3)
UCP3	602044	{Obesity, severe, and type II diabetes}, 601665 (3), Multifactorial, Autosomal dominant, Autosomal recessive
UFC1	610554	Neurodevelopmental disorder with spasticity and poor growth, 618076 (3), Autosomal recessive
UFM1	610553	Leukodystrophy, hypomyelinating, 14, 617899 (3), Autosomal recessive

UFSP2	611482	?Hip dysplasia, Beukes type, 142669 (3), Autosomal dominant; ?Spondyloepimetaphyseal dysplasia, Di Rocco type, 617974 (3), Autosomal dominant
UGDH	603370	Developmental and epileptic encephalopathy 84, 618792 (3), Autosomal recessive
UGP2	191760	Developmental and epileptic encephalopathy 83, 618744 (3), Autosomal recessive
UGT1A1	191740	Crigler-Najjar syndrome, type I, 218800 (3), Autosomal recessive; [Bilirubin, serum level of, QTL1], 601816 (3); Hyperbilirubinemia, familial transient neonatal, 237900 (3), Autosomal dominant, Autosomal recessive; Crigler-Najjar syndrome, type II, 606785 (3), Autosomal recessive; [Gilbert syndrome], 143500 (3), Autosomal recessive
UGT2B17	601903	{Bone mineral density QTL 12, osteoporosis}, 612560 (3)
UMOD	191845	Tubulointerstitial kidney disease, autosomal dominant, 1, 162000 (3), Autosomal dominant
UMPS	613891	Orotic aciduria, 258900 (3), Autosomal recessive
UNC119	604011	?Immunodeficiency 13, 615518 (3), Autosomal dominant; ?Cone-rod dystrophy (3)
UNC13D	608897	Hemophagocytic lymphohistiocytosis, familial, 3, 608898 (3), Autosomal recessive
UNC45A	611219	Osteootohepatoenteric syndrome, 619377 (3), Autosomal recessive
UNC45B	611220	?Cataract 43, 616279 (3), Autosomal dominant; Myofibrillar myopathy 11, 619178 (3), Autosomal recessive
UNC80	612636	Hypotonia, infantile, with psychomotor retardation and characteristic facies 2, 616801 (3), Autosomal recessive
UNC93B1	608204	{Encephalopathy, acute, infection-induced (herpes-specific), susceptibility to, 1}, 610551 (3)
UNG	191525	Immunodeficiency with hyper IgM, type 5, 608106 (3), Autosomal recessive
UPB1	606673	Beta-ureidopropionase deficiency, 613161 (3), Autosomal recessive
UPF3B	300298	Intellectual developmental disorder, X-linked syndromic 14, 300676 (3), X-linked recessive
UQCC2	614461	Mitochondrial complex III deficiency, nuclear type 7, 615824 (3), Autosomal recessive
UQCC3	616097	?Mitochondrial complex III deficiency, nuclear type 9, 616111 (3), Autosomal recessive
UQCRB	191330	Mitochondrial complex III deficiency, nuclear type 3, 615158 (3), Autosomal recessive
UQCRC1	191328	Parkinsonism with polyneuropathy, 619279 (3), Autosomal dominant
UQCRC2	191329	Mitochondrial complex III deficiency, nuclear type 5, 615160 (3), Autosomal recessive
UQCRFS1	191327	Mitochondrial complex III deficiency, nuclear type 10, 618775 (3), Autosomal recessive

UQCRQ	612080	Mitochondrial complex III deficiency, nuclear type 4, 615159 (3), Autosomal recessive
UROC1	613012	?Urocanase deficiency, 276880 (3), Autosomal recessive
UROD	613521	Porphyria, hepatoerythropoietic, 176100 (3), Autosomal dominant, Autosomal recessive; Porphyria cutanea tarda, 176100 (3), Autosomal dominant, Autosomal recessive
UROS	606938	Porphyria, congenital erythropoietic, 263700 (3), Autosomal recessive
USB1	613276	Poikiloderma with neutropenia, 604173 (3), Autosomal recessive
USF1	191523	{Hyperlipidemia, familial combined, susceptibility to}, 602491 (3)
USH1C	605242	Usher syndrome, type 1C, 276904 (3), Autosomal recessive; Deafness, autosomal recessive 18A, 602092 (3), Autosomal recessive
USH1G	607696	Usher syndrome, type 1G, 606943 (3), Autosomal recessive
USH2A	608400	Usher syndrome, type 2A, 276901 (3), Autosomal recessive; Retinitis pigmentosa 39, 613809 (3)
USP18	607057	Pseudo-TORCH syndrome 2, 617397 (3), Autosomal recessive
USP27X	300975	Intellectual developmental disorder, X-linked 105, 300984 (3), X-linked recessive
USP45	618439	?Leber congenital amaurosis 19, 618513 (3), Autosomal recessive
USP53	617431	Cholestasis, progressive familial intrahepatic, 7, with or without hearing loss, 619658 (3), Autosomal recessive
USP7	602519	Hao-Fountain syndrome, 616863 (3), Autosomal dominant
USP8	603158	Pituitary adenoma 4, ACTH-secreting, somatic, 219090 (3)
USP9X	300072	Intellectual developmental disorder, X-linked 99, 300919 (3), X-linked recessive; Intellectual developmental disorder, X-linked 99, syndromic, female-restricted, 300968 (3), X-linked dominant
USP9Y	400005	Spermatogenic failure, Y-linked, 2, 415000 (3), Y-linked
UVSSA	614632	UV-sensitive syndrome 3, 614640 (3), Autosomal recessive
VAC14	604632	Striatonigral degeneration, childhood-onset, 617054 (3), Autosomal recessive
VAMP1	185880	Myasthenic syndrome, congenital, 25, 618323 (3), Autosomal recessive; Spastic ataxia 1, autosomal dominant, 108600 (3), Autosomal dominant
VAMP2	185881	Neurodevelopmental disorder with hypotonia and autistic features with or without hyperkinetic movements, 618760 (3), Autosomal dominant
VANGL1	610132	{Neural tube defects, susceptibility to}, 182940 (3), Autosomal dominant; Caudal regression syndrome, 600145 (3), Autosomal dominant
VANGL2	600533	Neural tube defects, 182940 (3), Autosomal dominant
VAPB	605704	Spinal muscular atrophy, late-onset, Finkel type, 182980 (3), Autosomal dominant; Amyotrophic lateral sclerosis 8, 608627 (3), Autosomal dominant
VARS1	192150	Neurodevelopmental disorder with microcephaly, seizures, and cortical atrophy, 617802 (3), Autosomal recessive
VARS2	612802	Combined oxidative phosphorylation deficiency 20, 615917 (3), Autosomal recessive
VAX1	604294	?Microphthalmia, syndromic 11, 614402 (3), Autosomal recessive
VCAN	118661	Wagner syndrome 1, 143200 (3), Autosomal dominant

VCL	193065	Cardiomyopathy, dilated, 1W, 611407 (3); Cardiomyopathy, hypertrophic, 15, 613255 (3), Autosomal dominant
VCP	601023	Frontotemporal dementia and/or amyotrophic lateral sclerosis 6, 613954 (3), Autosomal dominant; Charcot-Marie-Tooth disease, type 2Y, 616687 (3), Autosomal dominant; Inclusion body myopathy with early-onset Paget disease and frontotemporal dementia 1, 167320 (3), Autosomal dominant
VDR	601769	Rickets, vitamin D-resistant, type IIA, 277440 (3), Autosomal recessive
VEGFA	192240	{Microvascular complications of diabetes 1}, 603933 (3)
VEGFC	601528	Lymphatic malformation 4, 615907 (3), Autosomal dominant
VHL	608537	Hemangioblastoma, cerebellar, somatic (3); Erythrocytosis, familial, 2, 263400 (3), Autosomal recessive; von Hippel-Lindau syndrome, 193300 (3), Autosomal dominant; Renal cell carcinoma, somatic, 144700 (3); Pheochromocytoma, 171300 (3), Autosomal dominant
VIM	193060	Cataract 30, pulverulent, 116300 (3), Autosomal dominant
VIPAS39	613401	Arthrogryposis, renal dysfunction, and cholestasis 2, 613404 (3), Autosomal recessive
VKORC1	608547	Vitamin K-dependent clotting factors, combined deficiency of, 2, 607473 (3), Autosomal recessive; Warfarin resistance, 122700 (3), Autosomal dominant
VLDLR	192977	Cerebellar hypoplasia and mental retardation with or without quadrupedal locomotion 1, 224050 (3), Autosomal recessive
VMA21	300913	Myopathy, X-linked, with excessive autophagy, 310440 (3), X-linked recessive
VNN1	603570	[High density lipoprotein cholesterol level QTL 8] (3)
VPS11	608549	?Dystonia 32, 619637 (3), Autosomal recessive; Leukodystrophy, hypomyelinating, 12, 616683 (3), Autosomal recessive
VPS13A	605978	Choreoacanthocytosis, 200150 (3), Autosomal recessive
VPS13B	607817	Cohen syndrome, 216550 (3), Autosomal recessive
VPS13C	608879	Parkinson disease 23, autosomal recessive, early onset, 616840 (3), Autosomal recessive
VPS13D	608877	Spinocerebellar ataxia, autosomal recessive 4, 607317 (3), Autosomal recessive
VPS16	608550	Dystonia 30, 619291 (3), Autosomal dominant
VPS33A	610034	Mucopolysaccharidosis-plus syndrome, 617303 (3), Autosomal recessive
VPS33B	608552	Keratoderma-ichthyosis-deafness syndrome, autosomal recessive, 620009 (3), Autosomal recessive; Cholestasis, progressive familial intrahepatic, 12, 620010 (3), Autosomal recessive; Arthrogryposis, renal dysfunction, and cholestasis 1, 208085 (3), Autosomal recessive
VPS35	601501	{Parkinson disease 17}, 614203 (3), Autosomal dominant
VPS35L	618981	Ritscher-Schinzel syndrome 3, 619135 (3), Autosomal recessive
VPS37A	609927	Spastic paraplegia 53, autosomal recessive, 614898 (3), Autosomal recessive
VPS41	605485	Spinocerebellar ataxia, autosomal recessive 29, 619389 (3), Autosomal recessive

VPS45	610035	Neutropenia, severe congenital, 5, autosomal recessive, 615285 (3), Autosomal recessive
VPS4A	609982	CIMDAG syndrome, 619273 (3), Autosomal dominant
VPS50	616465	Neurodevelopmental disorder with microcephaly, seizures, and neonatal cholestasis, 619685 (3), Autosomal recessive
VPS51	615738	Pontocerebellar hypoplasia, type 13, 618606 (3), Autosomal recessive
VPS53	615850	Pontocerebellar hypoplasia, type 2E, 615851 (3), Autosomal recessive
VRK1	602168	Pontocerebellar hypoplasia type 1A, 607596 (3), Autosomal recessive
VSX1	605020	?Craniofacial anomalies and anterior segment dysgenesis syndrome, 614195 (3); Keratoconus 1, 148300 (3), Autosomal dominant
VSX2	142993	Microphthalmia, isolated 2, 610093 (3); Microphthalmia with coloboma 3, 610092 (3)
VWA1	611901	Neuropathy, hereditary motor, with myopathic features, 619216 (3), Autosomal recessive
VWA3B	614884	?Spinocerebellar ataxia, autosomal recessive 22, 616948 (3), Autosomal recessive
VWF	613160	von Willebrand disease, type 1, 193400 (3), Autosomal dominant; von Willebrand disease, types 2A, 2B, 2M, and 2N, 613554 (3), Autosomal dominant, Autosomal recessive; von Willebrand disease, type 3, 277480 (3), Autosomal recessive
WAC	615049	Desanto-Shinawi syndrome, 616708 (3), Autosomal dominant
WARS1	191050	Neuronopathy, distal hereditary motor, type IX, 617721 (3), Autosomal dominant
WARS2	604733	Parkinsonism-dystonia 3, childhood-onset, 619738 (3), Autosomal recessive; Neurodevelopmental disorder, mitochondrial, with abnormal movements and lactic acidosis, with or without seizures, 617710 (3), Autosomal recessive
WAS	300392	Wiskott-Aldrich syndrome, 301000 (3), X-linked recessive; Neutropenia, severe congenital, X-linked, 300299 (3), X-linked recessive; Thrombocytopenia, X-linked, intermittent, 313900 (3), X-linked recessive; Thrombocytopenia, X-linked, 313900 (3), X-linked recessive
WASF1	605035	Neurodevelopmental disorder with absent language and variable seizures, 618707 (3), Autosomal dominant
WASHC4	615748	Intellectual developmental disorder, autosomal recessive 43, 615817 (3), Autosomal recessive
WASHC5	610657	Ritscher-Schinzel syndrome 1, 220210 (3), Autosomal recessive; Spastic paraplegia 8, autosomal dominant, 603563 (3), Autosomal dominant
WBP11	618083	Vertebral, cardiac, tracheoesophageal, renal, and limb defects, 619227 (3), Autosomal dominant
WBP2	606962	Deafness, autosomal recessive 107, 617639 (3), Autosomal recessive
WDFY3	617485	?Microcephaly 18, primary, autosomal dominant, 617520 (3), Autosomal dominant

WDPCP	613580	?Bardet-Biedl syndrome 15, 615992 (3), Autosomal recessive; Congenital heart defects, hamartomas of tongue, and polysyndactyly, 217085 (3), Autosomal recessive
WDR1	604734	Periodic fever, immunodeficiency, and thrombocytopenia syndrome, 150550 (3), Autosomal recessive
WDR11	606417	Hypogonadotropic hypogonadism 14 with or without anosmia, 614858 (3), Autosomal dominant
WDR19	608151	Nephronophthisis 13, 614377 (3), Autosomal recessive; Cranioectodermal dysplasia 4, 614378 (3), Autosomal recessive; Senior-Loken syndrome 8, 616307 (3), Autosomal recessive; Short-rib thoracic dysplasia 5 with or without polydactyly, 614376 (3), Autosomal recessive; ?Spermatogenic failure 72, 619867 (3), Autosomal recessive
WDR26	617424	Skraban-Deardorff syndrome, 617616 (3), Autosomal dominant
WDR35	613602	Short-rib thoracic dysplasia 7 with or without polydactyly, 614091 (3), Autosomal recessive; Cranioectodermal dysplasia 2, 613610 (3), Autosomal recessive
WDR36	609669	Glaucoma 1, open angle, G, 609887 (3)
WDR37	618586	Neurooculocardiogenitourinary syndrome, 618652 (3), Autosomal dominant
WDR4	605924	Galloway-Mowat syndrome 6, 618347 (3), Autosomal recessive; Microcephaly, growth deficiency, seizures, and brain malformations, 618346 (3), Autosomal recessive
WDR45	300526	Neurodegeneration with brain iron accumulation 5, 300894 (3), X-linked dominant
WDR45B	609226	Neurodevelopmental disorder with spastic quadriplegia and brain abnormalities with or without seizures, 617977 (3), Autosomal recessive
WDR62	613583	Microcephaly 2, primary, autosomal recessive, with or without cortical malformations, 604317 (3), Autosomal recessive
WDR72	613214	Amelogenesis imperfecta, type IIA3, 613211 (3), Autosomal recessive
WDR73	616144	Galloway-Mowat syndrome 1, 251300 (3), Autosomal recessive
WDR81	614218	Cerebellar ataxia, mental retardation, and dysequilibrium syndrome 2, 610185 (3), Autosomal recessive; Hydrocephalus, congenital, 3, with brain anomalies, 617967 (3), Autosomal recessive
WEE2	614084	Oocyte maturation defect 5, 617996 (3), Autosomal recessive
WFS1	606201	Deafness, autosomal dominant 6/14/38, 600965 (3), Autosomal dominant; ?Cataract 41, 116400 (3), Autosomal dominant; Wolfram-like syndrome, autosomal dominant, 614296 (3), Autosomal dominant; {Diabetes mellitus, noninsulin-dependent, association with}, 125853 (3), Autosomal dominant; Wolfram syndrome 1, 222300 (3), Autosomal recessive
WHRN	607928	Deafness, autosomal recessive 31, 607084 (3), Autosomal recessive; Usher syndrome, type 2D, 611383 (3), Autosomal recessive
WIPF1	602357	Wiskott-Aldrich syndrome 2, 614493 (3), Autosomal recessive
WIPI2	609225	?Intellectual developmental disorder with short stature and variable skeletal anomalies, 618453 (3), Autosomal recessive

WLS	611514	Zaki syndrome, 619648 (3), Autosomal recessive
WNK1	605232	Neuropathy, hereditary sensory and autonomic, type II, 201300 (3), Autosomal recessive; Pseudohypoaldosteronism, type IIC, 614492 (3), Autosomal dominant
WNK4	601844	Pseudohypoaldosteronism, type IIB, 614491 (3), Autosomal dominant
WNT1	164820	{Osteoporosis, early-onset, susceptibility to, autosomal dominant}, 615221 (3); Osteogenesis imperfecta, type XV, 615220 (3), Autosomal recessive
WNT10A	606268	Schopf-Schulz-Passarge syndrome, 224750 (3), Autosomal recessive; Tooth agenesis, selective, 4, 150400 (3), Autosomal dominant, Autosomal recessive; Odontoonychodermal dysplasia, 257980 (3), Autosomal recessive
WNT10B	601906	Tooth agenesis, selective, 8, 617073 (3), Autosomal dominant; Split-hand/foot malformation 6, 225300 (3), Autosomal recessive
WNT2B	601968	Diarrhea 9, 618168 (3), Autosomal recessive
WNT3	165330	?Tetra-amelia syndrome 1, 273395 (3), Autosomal recessive
WNT4	603490	?SERKAL syndrome, 611812 (3), Autosomal recessive; Mullerian aplasia and hyperandrogenism, 158330 (3), Autosomal dominant
WNT5A	164975	Robinow syndrome, autosomal dominant 1, 180700 (3), Autosomal dominant
WNT7A	601570	Fuhrmann syndrome, 228930 (3), Autosomal recessive; Ulna and fibula, absence of, with severe limb deficiency, 276820 (3), Autosomal recessive
WRAP53	612661	Dyskeratosis congenita, autosomal recessive 3, 613988 (3), Autosomal recessive
WRN	604611	Werner syndrome, 277700 (3), Autosomal recessive
WT1	607102	Mesothelioma, somatic, 156240 (3); Meacham syndrome, 608978 (3), Autosomal dominant; Frasier syndrome, 136680 (3), Autosomal dominant, Somatic mutation; Nephrotic syndrome, type 4, 256370 (3), Autosomal dominant; Denys-Drash syndrome, 194080 (3), Autosomal dominant, Somatic mutation; Wilms tumor, type 1, 194070 (3), Autosomal dominant, Somatic mutation
WWC1	610533	[Memory, enhanced, QTL], 615602 (3)
WWOX	605131	Esophageal squamous cell carcinoma, somatic, 133239 (3); Developmental and epileptic encephalopathy 28, 616211 (3), Autosomal recessive; Spinocerebellar ataxia, autosomal recessive 12, 614322 (3), Autosomal recessive
XBP1	194355	{Major affective disorder-7, susceptibility to}, 612371 (3)
XDH	607633	Xanthinuria, type I, 278300 (3), Autosomal recessive
XG	300879/314705	-/[XG blood group system, Xg(a-) phenotype, 314700 (3)
XIAP	300079	Lymphoproliferative syndrome, X-linked, 2, 300635 (3), X-linked recessive
XIST	314670	X-inactivation, familial skewed, 300087 (3)
XK	314850	McLeod syndrome with or without chronic granulomatous disease, 300842 (3), X-linked
XPA	611153	Xeroderma pigmentosum, group A, 278700 (3), Autosomal recessive
XPC	613208	Xeroderma pigmentosum, group C, 278720 (3), Autosomal recessive

XPNPEP2	300145	{Angioedema induced by ACE inhibitors, susceptibility to}, 300909 (3)
XPNPEP3	613553	Nephronophthisis-like nephropathy 1, 613159 (3), Autosomal recessive
XPR1	605237	Basal ganglia calcification, idiopathic, 6, 616413 (3), Autosomal dominant
XRCC1	194360	?Spinocerebellar ataxia, autosomal recessive 26, 617633 (3), Autosomal recessive
XRCC2	600375	Spermatogenic failure 50, 619145 (3), Autosomal recessive; ?Premature ovarian failure 17, 619146 (3), Autosomal recessive; ?Fanconi anemia, complementation group U, 617247 (3), Autosomal recessive
XRCC3	600675	{Breast cancer, susceptibility to}, 114480 (3), Autosomal dominant, Somatic mutation; {Melanoma, cutaneous malignant, 6}, 613972 (3)
XRCC4	194363	Short stature, microcephaly, and endocrine dysfunction, 616541 (3), Autosomal recessive
XYLT1	608124	Desbuquois dysplasia 2, 615777 (3), Autosomal recessive; {Pseudoxanthoma elasticum, modifier of severity of}, 264800 (3), Autosomal recessive
XYLT2	608125	{Pseudoxanthoma elasticum, modifier of severity of}, 264800 (3), Autosomal recessive; Spondyloocular syndrome, 605822 (3), Autosomal recessive
YAP1	606608	Coloboma, ocular, with or without hearing impairment, cleft lip/palate, and/or mental retardation, 120433 (3), Autosomal dominant
YARS1	603623	Infantile-onset multisystem neurologic, endocrine, and pancreatic disease 2, 619418 (3), Autosomal recessive; Charcot-Marie-Tooth disease, dominant intermediate C, 608323 (3), Autosomal dominant
YARS2	610957	Myopathy, lactic acidosis, and sideroblastic anemia 2, 613561 (3), Autosomal recessive
YEATS2	613373	?Epilepsy, myoclonic, familial adult, 4, 615127 (3), Autosomal dominant
YIF1B	619109	Kaya-Barakat-Masson syndrome, 619125 (3), Autosomal recessive
YIPF5	611483	Microcephaly, epilepsy, and diabetes syndrome 2, 619278 (3), Autosomal recessive
YME1L1	607472	?Optic atrophy 11, 617302 (3), Autosomal recessive
YRDC	612276	Galloway-Mowat syndrome 10, 619609 (3), Autosomal recessive
YWHAG	605356	Developmental and epileptic encephalopathy 56, 617665 (3), Autosomal dominant
YY1	600013	Gabriele-de Vries syndrome, 617557 (3), Autosomal dominant
YY1AP1	607860	Grange syndrome, 602531 (3), Autosomal recessive
ZAP70	176947	Immunodeficiency 48, 269840 (3), Autosomal recessive; Autoimmune disease, multisystem, infantile-onset, 2, 617006 (3), Autosomal recessive
ZBTB11	618181	Intellectual developmental disorder, autosomal recessive 69, 618383 (3), Autosomal recessive
ZBTB16	176797	Leukemia, acute promyelocytic, PL2F/RARA type (3)
ZBTB18	608433	Intellectual developmental disorder, autosomal dominant 22, 612337 (3), Autosomal dominant
ZBTB20	606025	Primrose syndrome, 259050 (3), Autosomal dominant
ZBTB24	614064	Immunodeficiency-centromeric instability-facial anomalies syndrome 2, 614069 (3), Autosomal recessive

ZBTB42	613915	?Lethal congenital contracture syndrome 6, 616248 (3), Autosomal recessive
ZBTB7A	605878	Macrocephaly, neurodevelopmental delay, lymphoid hyperplasia, and persistent fetal hemoglobin, 619769 (3), Autosomal dominant
ZC3H14	613279	Intellectual developmental disorder, autosomal recessive 56, 617125 (3), Autosomal recessive
ZC4H2	300897	Wieacker-Wolff syndrome, 314580 (3), X-linked recessive; Wieacker-Wolff syndrome, female-restricted, 301041 (3), X-linked dominant
ZCCHC8	616381	?Pulmonary fibrosis and/or bone marrow failure, telomere-related, 5, 618674 (3), Autosomal dominant
ZDHHC15	300576	Mental retardation, X-linked 91, 300577 (4), X-linked dominant
ZDHHC9	300646	Intellectual developmental disorder, X-linked syndromic, Raymond type, 300799 (3), X-linked
ZEB1	189909	Corneal dystrophy, posterior polymorphous, 3, 609141 (3); Corneal dystrophy, Fuchs endothelial, 6, 613270 (3)
ZEB2	605802	Mowat-Wilson syndrome, 235730 (3), Autosomal dominant
ZFAT	610931	{Autoimmune thyroid disease, susceptibility to, 3}, 608175 (3)
ZFHX2	617828	?Marsili syndrome, 147430 (3), Autosomal dominant
ZFHX3	104155	Prostate cancer, somatic, 176807 (3)
ZFHX4	606940	?Ptosis, congenital, 178300 (2), Autosomal dominant
ZFP57	612192	Diabetes mellitus, transient neonatal 1, 601410 (3), Autosomal dominant, Autosomal recessive
ZFPM2	603693	Diaphragmatic hernia 3, 610187 (3); 46XY sex reversal 9, 616067 (3), Autosomal dominant; Tetralogy of Fallot, 187500 (3), Autosomal dominant
ZFYVE19	619635	Cholestasis, progressive familial intrahepatic, 9, 619849 (3), Autosomal recessive
ZFYVE26	612012	Spastic paraplegia 15, autosomal recessive, 270700 (3), Autosomal recessive
ZFYVE27	610243	Spastic paraplegia 33, autosomal dominant, 610244 (3), Autosomal dominant
ZIC1	600470	?Craniosynostosis 6, 616602 (3), Autosomal dominant; Structural brain anomalies with impaired intellectual development and craniosynostosis, 618736 (3), Autosomal dominant
ZIC2	603073	Holoprosencephaly 5, 609637 (3), Autosomal dominant
ZIC3	300265	Congenital heart defects, nonsyndromic, 1, X-linked, 306955 (3), X-linked recessive; Heterotaxy, visceral, 1, X-linked, 306955 (3), X-linked recessive; VACTERL association, X-linked, 314390 (3), X-linked recessive
ZMIZ1	607159	Neurodevelopmental disorder with dysmorphic facies and distal skeletal anomalies, 618659 (3), Autosomal dominant
ZMPSTE24	606480	Mandibuloacral dysplasia with type B lipodystrophy, 608612 (3), Autosomal recessive; Restrictive dermopathy 1, 275210 (3), Autosomal recessive
ZMYM2	602221	Neurodevelopmental-craniofacial syndrome with variable renal and cardiac abnormalities, 619522 (3), Autosomal dominant
ZMYND10	607070	Ciliary dyskinesia, primary, 22, 615444 (3), Autosomal recessive

ZMYND11	608668	Intellectual developmental disorder, autosomal dominant 30, 616083 (3), Autosomal dominant
ZMYND15	614312	?Spermatogenic failure 14, 615842 (3), Autosomal recessive
ZNF141	194648	?Polydactyly, postaxial, type A6, 615226 (3), Autosomal recessive
ZNF142	604083	Neurodevelopmental disorder with impaired speech and hyperkinetic movements, 618425 (3), Autosomal recessive
ZNF148	601897	Global developmental delay, absent or hypoplastic corpus callosum, and dysmorphic facies, 617260 (3), Autosomal dominant
ZNF292	616213	Intellectual developmental disorder, autosomal dominant 64, 619188 (3), Autosomal dominant
ZNF335	610827	Microcephaly 10, primary, autosomal recessive, 615095 (3), Autosomal recessive
ZNF341	618269	Hyper-IgE recurrent infection syndrome 3, autosomal recessive, 618282 (3), Autosomal recessive
ZNF365	607818	{Nephrolithiasis, uric acid, susceptibility to}, 605990 (3)
ZNF407	615894	SIMHA syndrome, 619557 (3), Autosomal recessive
ZNF408	616454	Retinitis pigmentosa 72, 616469 (3), Autosomal recessive; ?Exudative vitreoretinopathy 6, 616468 (3), Autosomal dominant
ZNF423	604557	Nephronophthisis 14, 614844 (3), Autosomal dominant, Autosomal recessive; Joubert syndrome 19, 614844 (3), Autosomal dominant, Autosomal recessive
ZNF462	617371	Weiss-Kruszka syndrome, 618619 (3), Autosomal dominant
ZNF469	612078	Brittle cornea syndrome 1, 229200 (3), Autosomal recessive
ZNF513	613598	?Retinitis pigmentosa 58, 613617 (3), Autosomal recessive
ZNF526	614387	Dentici-Novelli neurodevelopmental syndrome, 619877 (3), Autosomal recessive
ZNF644	614159	Myopia 21, autosomal dominant, 614167 (3), Autosomal dominant
ZNF687	610568	Paget disease of bone 6, 616833 (3), Autosomal dominant
ZNF699	609571	DEGCAGS syndrome, 619488 (3), Autosomal recessive
ZNF711	314990	Intellectual developmental disorder, X-linked 97, 300803 (3), X-linked
ZNF750	610226	?Seborrhea-like dermatitis with psoriasiform elements, 610227 (3)
ZNFX1	618931	Immunodeficiency 91 and hyperinflammation, 619644 (3), Autosomal recessive
ZNHIT3	604500	PEHO syndrome, 260565 (3), Autosomal recessive
ZP1	195000	Oocyte maturation defect 1, 615774 (3), Autosomal recessive
ZP2	182888	Oocyte maturation defect 6, 618353 (3), Autosomal recessive
ZP3	182889	Oocyte maturation defect 3, 617712 (3), Autosomal dominant
ZPBP	608498	?Spermatogenic failure 66, 619799 (3), Autosomal recessive
ZPR1	603901	?Growth restriction, hypoplastic kidneys, alopecia, and distinctive facies, 619321 (3), Autosomal recessive
ZSWIM6	615951	Neurodevelopmental disorder with movement abnormalities, abnormal gait, and autistic features, 617865 (3), Autosomal dominant; Acromelic frontonasal dysostosis, 603671 (3), Autosomal dominant

ZSWIM7	614535	Spermatogenic failure 71, 619831 (3), Autosomal recessive; ?Ovarian dysgenesis 10, 619834 (3), Autosomal recessive

Gene symbols used are according to the HGNC guidelines. For some genes a previously HGNC-approved symbol is in brackets.
Each Phenotype is followed by its MIM number, phenotype mapping key and inheritance pattern.
OMIM release used for OMIM disease identifiers and descriptions: July 26, 2021

Possible phenotype mapping keys
(1) the disorder is placed on the map based on its association with a gene, but the underlying defect is not known
(2) the disorder has been placed on the map by linkage; no mutation has been found
(3) the molecular basis for the disorder is known; a mutation has been found in the gene
(4) a contiguous gene deletion or duplication syndrome, multiple genes are deleted or duplicated causing the phenotype

Brackets, "[ ]", indicate "nondiseases," mainly genetic variations that lead to apparently abnormal laboratory test values (e.g., dysalbuminemic euthyroidal hyperthyroxinemia).
Braces, "{ }", indicate mutations that contribute to susceptibility to multifactorial disorders (e.g., diabetes, asthma) or to susceptibility to infection (e.g., malaria).
A question mark, "?", before the phenotype name indicates that the relationship between the phenotype and gene is provisional. More details about this relationship are provided in the comment field of the map and in the gene and phenotype OMIM entries.
