# Supplementary material for: A distant global control region is essential for normal expression of anterior HOXA genes during mouse and human craniofacial development
Source: Nat Commun. 2024 Jan 2;15:136. doi: 10.1038/s41467-023-44506-2 (PMC10762089; doi:10.1038/s41467-023-44506-2)
Supplement: Supplementary file 21 — Supplementary Information [file 41467_2023_44506_MOESM21_ESM.pdf]

Supplemental Text

Gynecologists report:
A 30-year old G1P0 was referred to our tertiary centre for targeted ultrasonography at 13 weeks due to abnormal profile. We noted an abnormal retronasal triangle, absence of the palate in sagittal view and a protruding median part of the maxilla, indicating a severe bilateral cheilognathopalatoschisis. Forearms and hands were structurally normal but afunctional. Both feet were abnormally positioned. Termination of pregnancy on the couple's request was performed at 14 weeks. Consent was given to publish photographs and molecular data.

For the second case report consent was given to publish Magnetic Resonance Imaging (MRI), Computed Tomography (CT) Images, and molecular data.

16 Supplemental Tables and 29 Supplemental Figures

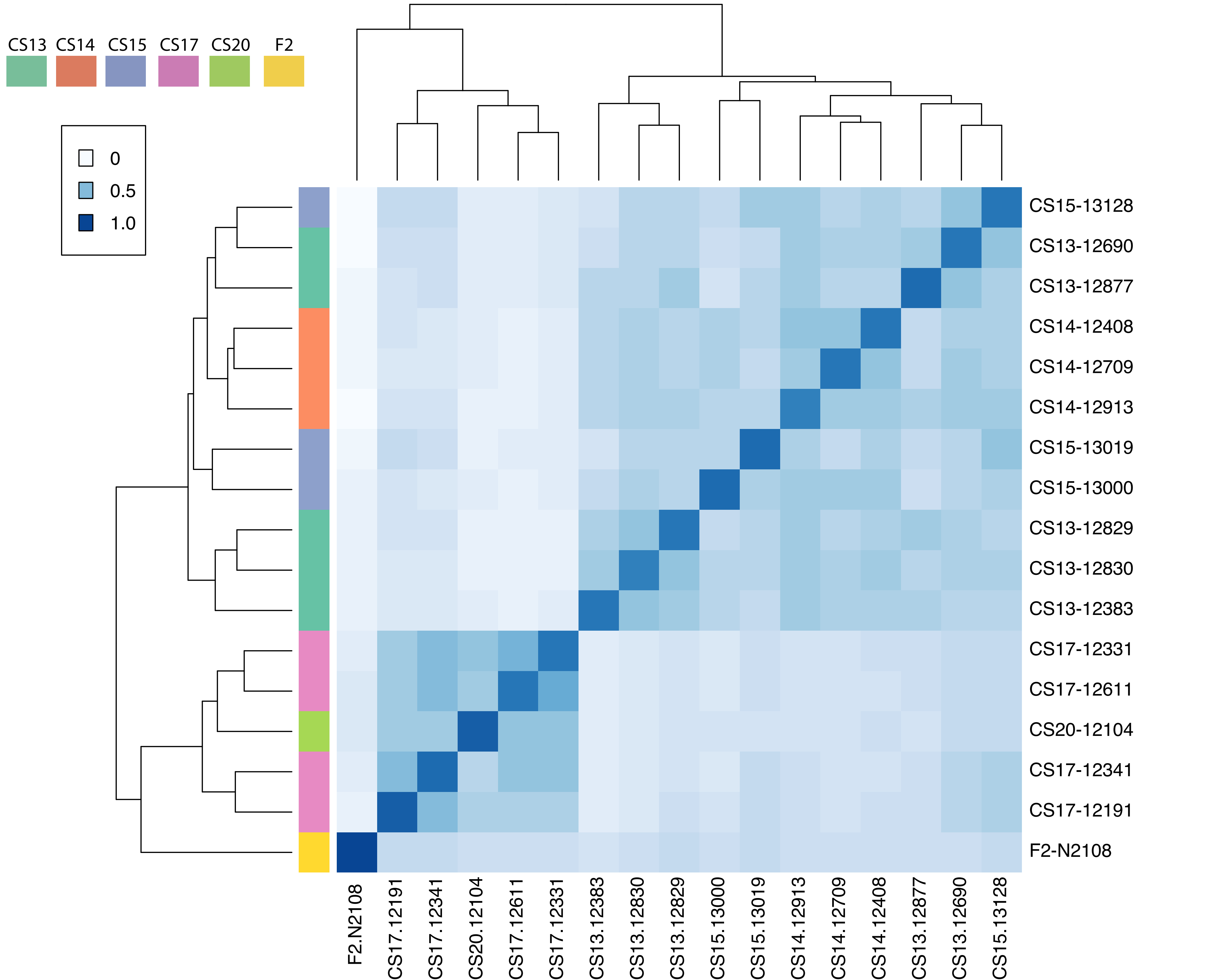
CS13
CS14
CS15
CS17
CS20
F2
0
0.5
1.0
CS15-13128
CS13-12690
CS13-12877
CS14-12408
CS14-12709
CS14-12913
CS15-13019
CS15-13000
CS13-12829
CS13-12830
CS13-12383
CS17-12331
CS17-12611
CS20-12104
CS17-12341
CS17-12191
F2-N2108
F2.N2108
CS17.12191
CS17.12341
CS20.12104
CS17.12611
CS17.12331
CS13.12383
CS13.12830
CS13.12829
CS15.13000
CS15.13019
CS14.12913
CS14.12709
CS14.12408
CS13.12877
CS13.12690
CS15.13128

Figure S1- Jaccard similarity of superenhancer sequences in human embryonic craniofacial tissue samples.

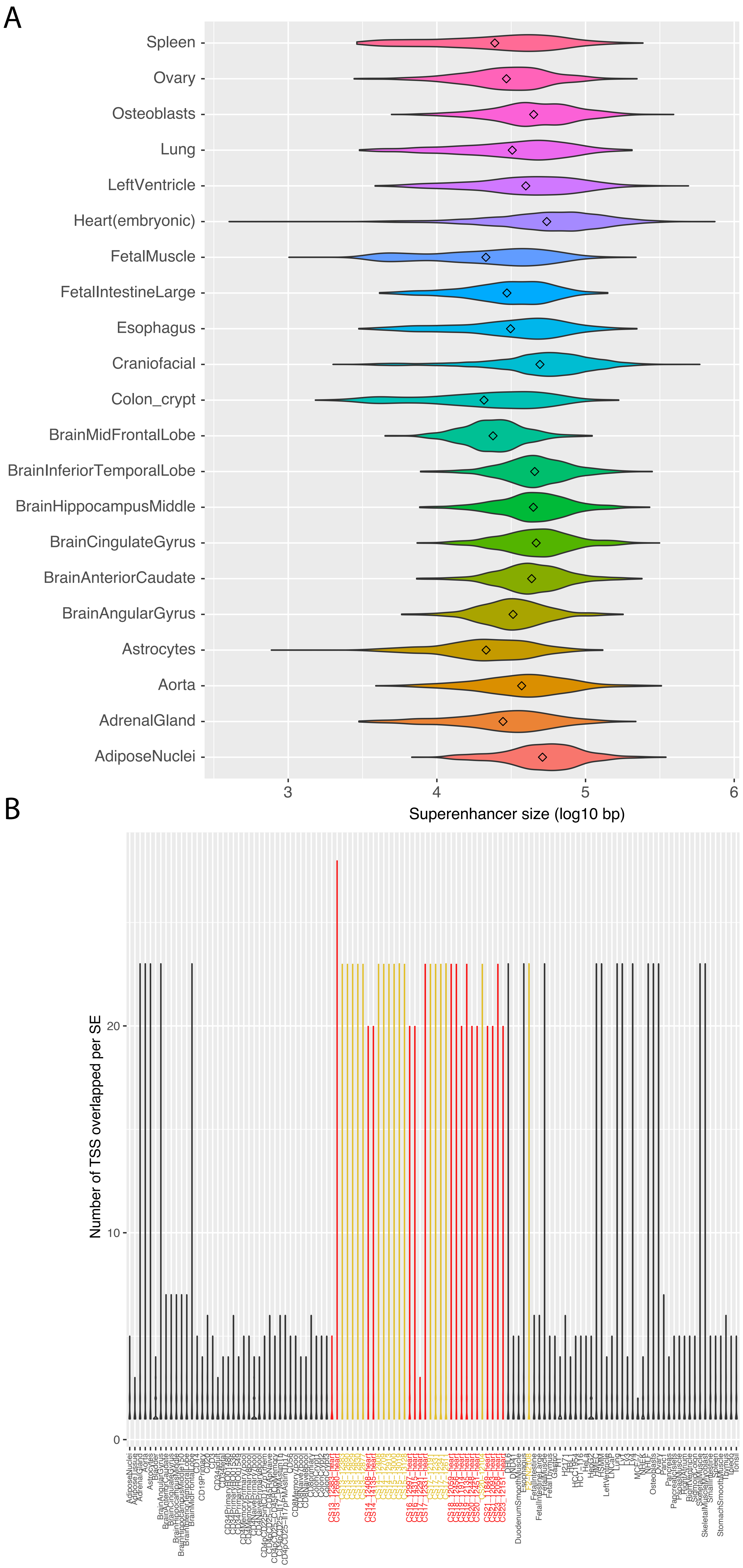
A
Spleen
Ovary
Osteoblasts
Lung
LeftVentricle
Heart(embryonic)
FetalMuscle
FetalIntestineLarge
Esophagus
Craniofacial
Colon_crypt
BrainMidFrontalLobe
BrainInferiorTemporalLobe
BrainHippocampusMiddle
BrainCingulateGyrus
BrainAnteriorCaudate
BrainAngularGyrus
Astrocytes
Aorta
AdrenalGland
AdiposeNuclei
3
4
5
6
Superenhancer size (log10 bp)
B
Number of TSS overlapped per SE
0
10
20

Figure S2- Violin plots of number of transcription start sites (TSSs) overlapped by superenhancers in various tissues. Plots in black reference tissues and cell types in the dbSuper database. Plots in yellow are the results from human embryonic craniofacial tissue. Plots in red are the results from human embryonic heart. Superenhancers encompassing at minimum 1 and as many as 23 TSSs are found across multiple tissues. Median number of TSSs for protein-coding genes encompassed by superenhancers in dbSuper tissues and cell lines = 1. Median number of TSSs for protein coding genes encompassed by superenhancers in craniofacial and heart samples ranged from 1-3.

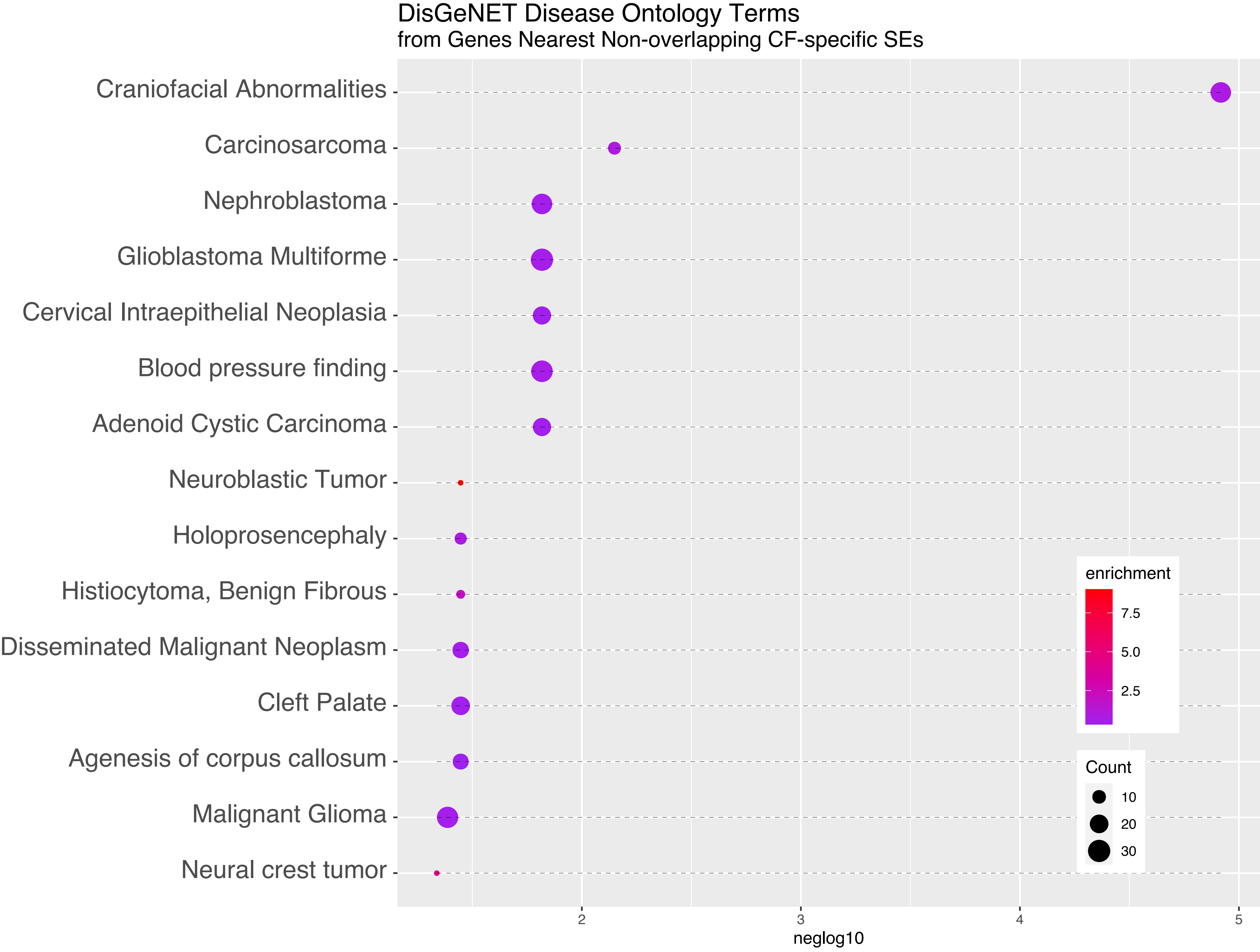
DisGeNET Disease Ontology Terms
from Genes Nearest Non-overlapping CF-specific SEs
Craniofacial Abnormalities
Carcinosarcoma
Nephroblastoma
Glioblastoma Multiforme
Cervical Intraepithelial Neoplasia
Blood pressure finding
Adenoid Cystic Carcinoma
Neuroblastic Tumor
Holoprosencephaly
Histiocytoma, Benign Fibrous
Disseminated Malignant Neoplasm
Cleft Palate
Agenesis of corpus callosum
Malignant Glioma
Neural crest tumor
enrichment
7.5
5.0
2.5
Count
10
20
30
2
3
4
5
neglog10

Figure S3- Biological Process Gene Ontology terms enriched in genes assigned to superenhancers in non-coding regions. Assignment of the two nearest genes was done through the BedTools suite function "closest".

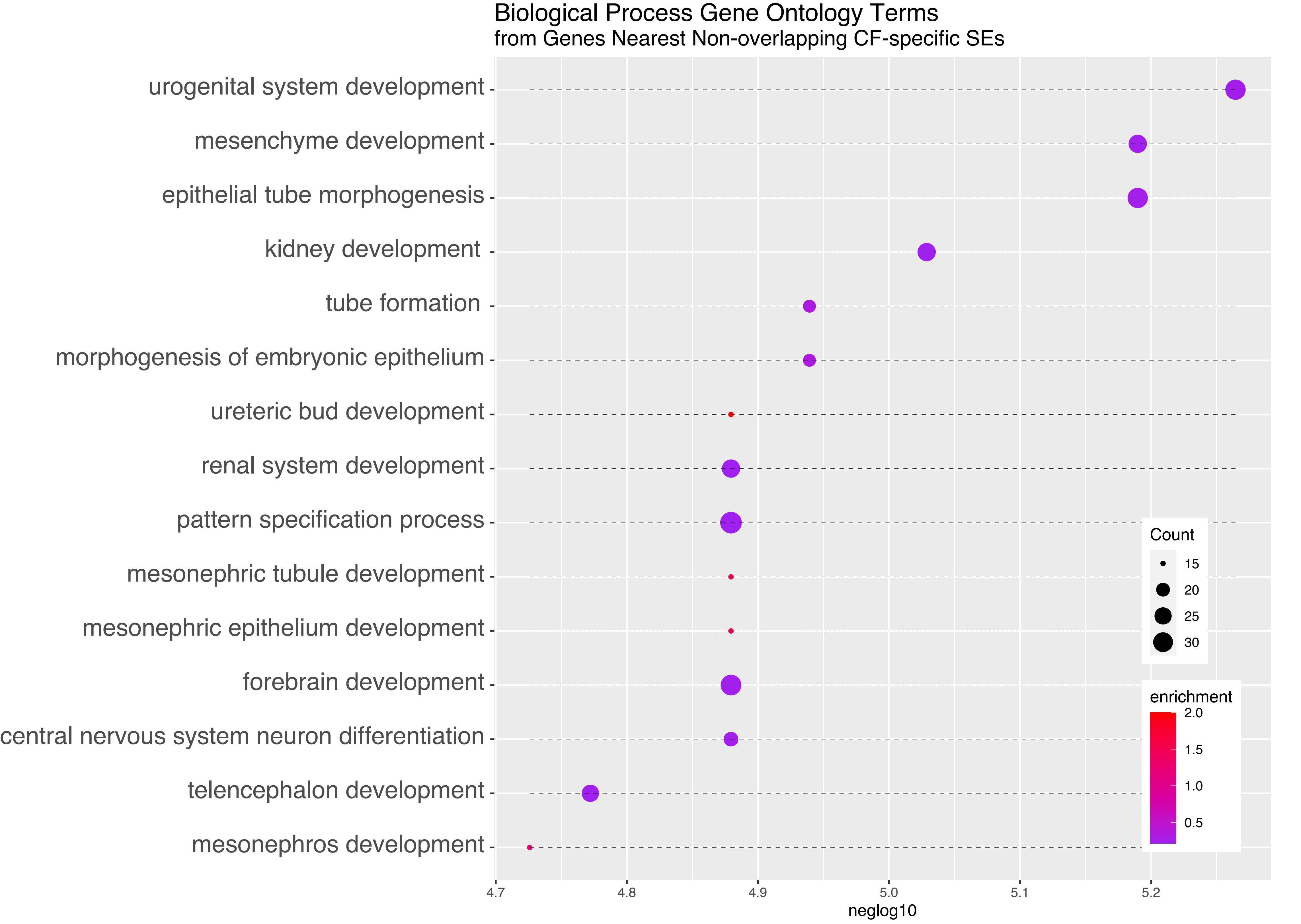

Biological Process Gene Ontology Terms
from Genes Nearest Non-overlapping CF-specific SEs
urogenital system development
mesenchyme development
epithelial tube morphogenesis
kidney development
tube formation
morphogenesis of embryonic epithelium
ureteric bud development
renal system development
pattern specification process
mesonephric tubule development
mesonephric epithelium development
forebrain development
central nervous system neuron differentiation
telencephalon development
mesonephros development
4.7
4.8
4.9
5.0
5.1
5.2
neglog10
Count
15
20
25
30
enrichment
2.0
1.5
1.0
0.5

Figure S4- Disease Ontology terms from DisGeNet enriched in genes assigned to superenhancers in non-coding regions. Assignment of the two nearest genes was done through the BEDTools suite function "closest".

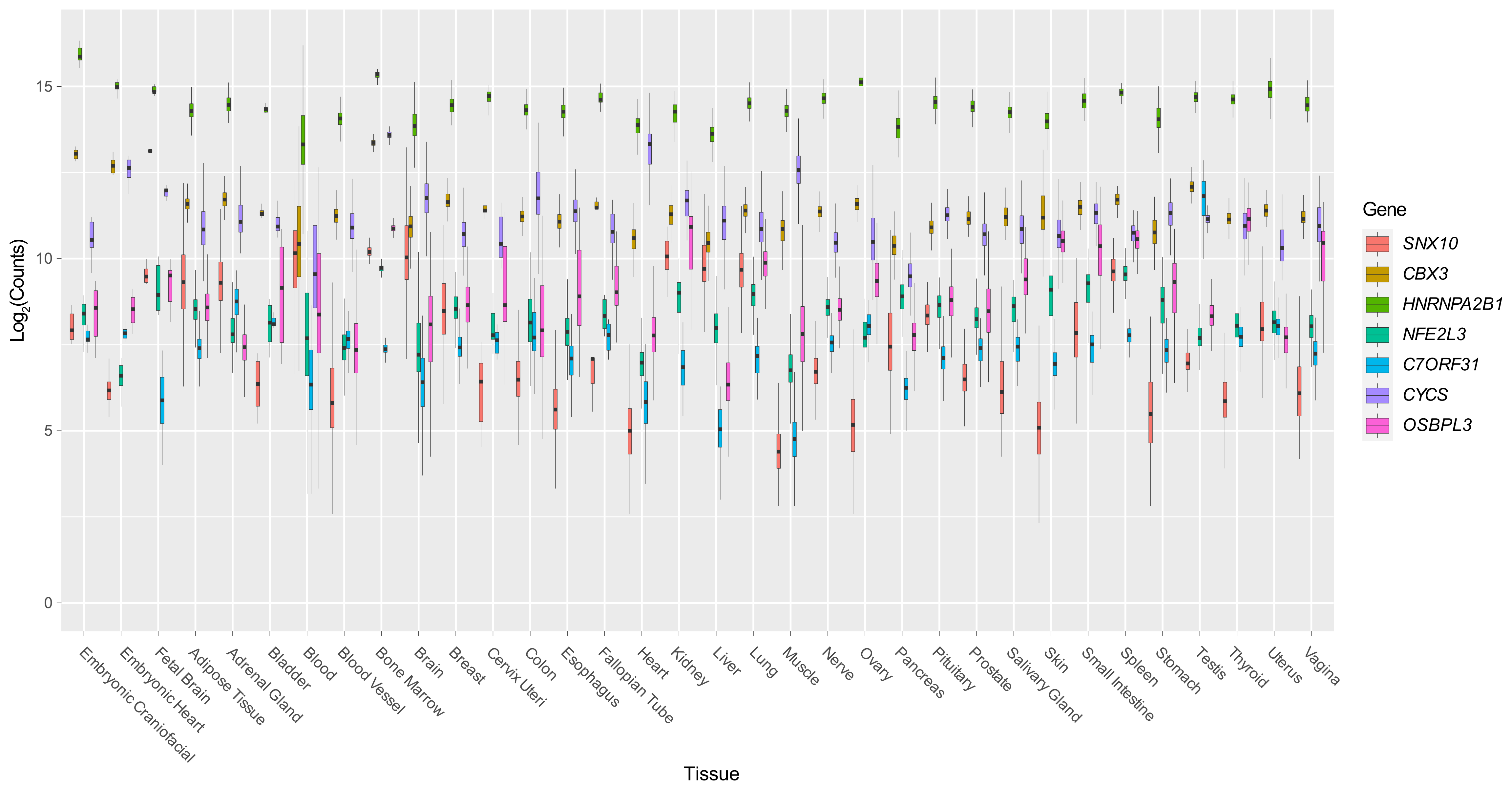

Log2(Counts)
15
10
5
0
Tissue
Embryonic Craniofacial
Embryonic Heart
Fetal Brain
Adipose Tissue
Adrenal Gland
Bladder
Blood
Blood Vessel
Bone Marrow
Brain
Breast
Cervix Uteri
Colon
Esophagus
Fallopian Tube
Heart
Kidney
Liver
Lung
Muscle
Nerve
Ovary
Pancreas
Pituitary
Prostate
Salivary Gland
Skin
Small Intestine
Spleen
Stomach
Testis
Thyroid
Uterus
Vagina
Gene
SNX10
CBX3
HNRNPA2B1
NFE2L3
C7ORF31
CYCS
OSBPL3

Figure S5- Expression of genes within 500kb of gene desert.

The $\log_2 + 1$ of counts from human primary craniofacial tissue, embryonic heart, fetal brain and 31 adult tissues from GTEx. These genes show ubiquitous expression across all tissues surveyed.

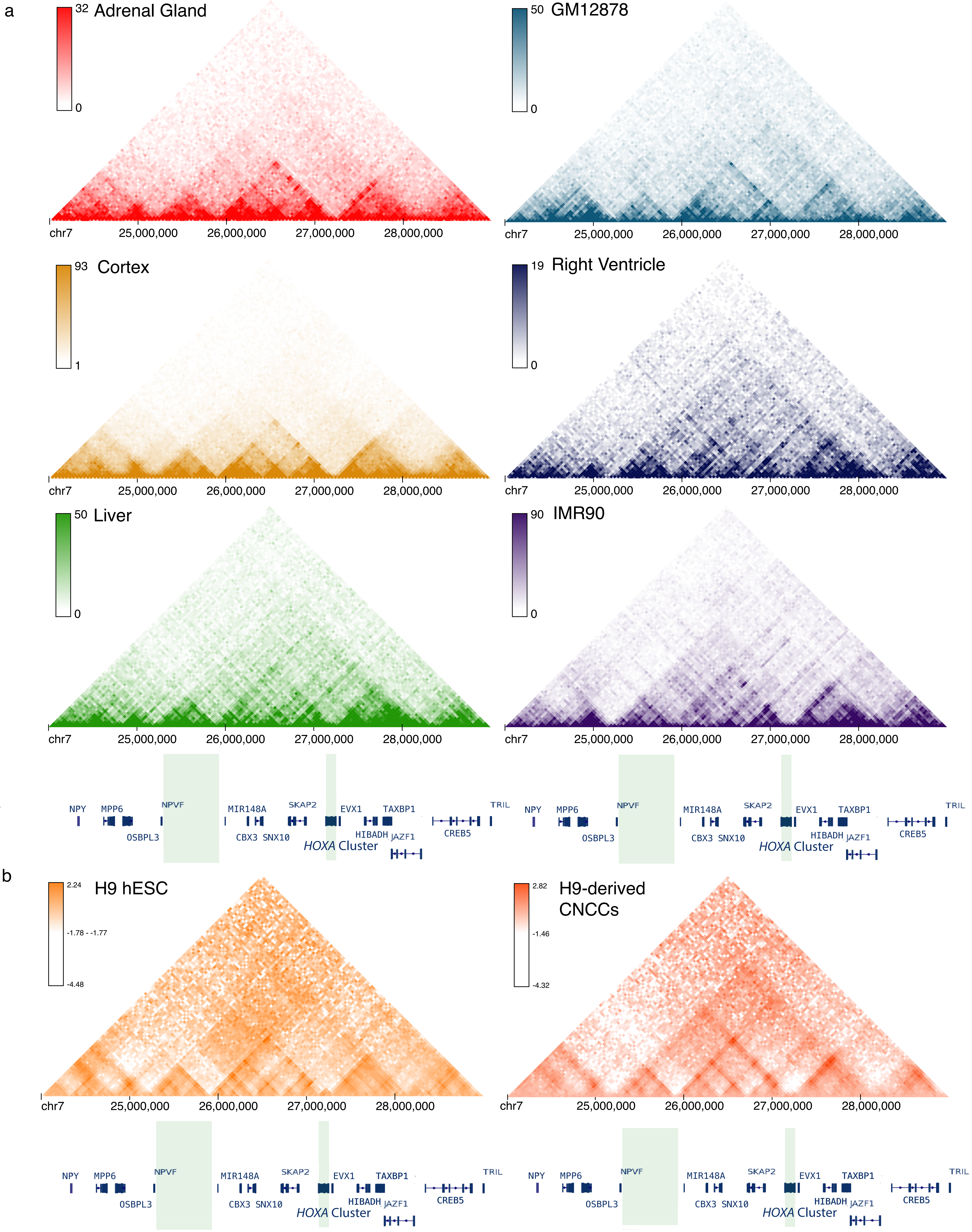

a
Adrenal Gland
32
0
chr7
25,000,000
26,000,000
27,000,000
28,000,000
GM12878
50
0
Cortex
93
1
Right Ventricle
19
0
Liver
50
0
IMR90
90
0
NPY
MPP6
NPVF
MIR148A
SKAP2
EVX1
TAXBP1
TRIL
OSBPL3
CBX3
SNX10
HIBADH
JAZF1
CREB5
HOXA Cluster
b
H9 hESC
2.24
-1.78 - -1.77
-4.48
H9-derived CNCCs
2.82
-1.46
-4.32

Figure S6- HiC from publicly available data of various human tissues and cell lines (a) and publicly available data from H9 (b- left) and H9-derived CNCCs (this study, b-right). Plots were generated through the HiC Browser hosted by Northwestern University. *HOXA* cluster and putative novel superenhancer region are highlighted with green shading.

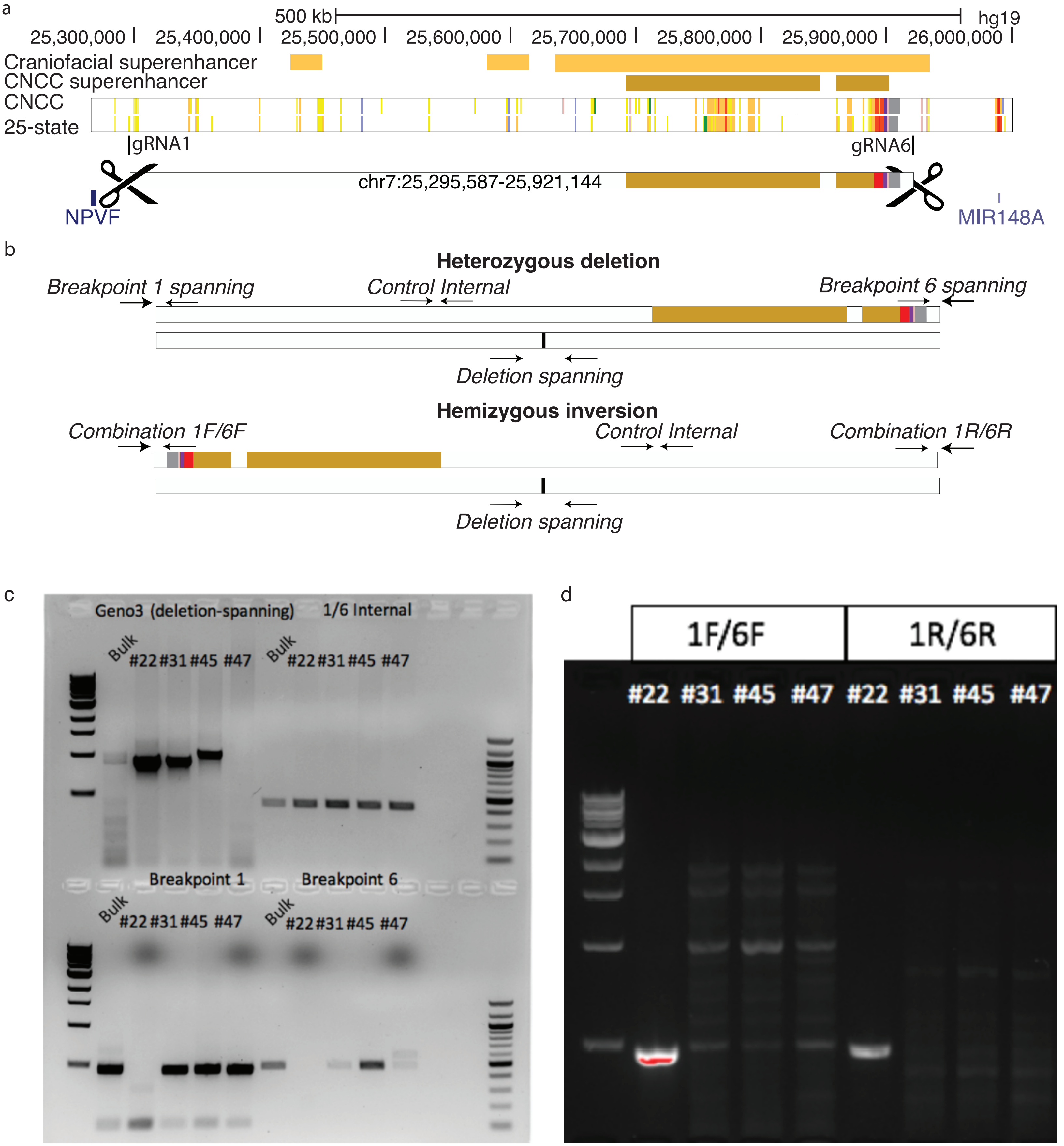
a
500 kb
hg19
25,300,000
25,400,000
25,500,000
25,600,000
25,700,000
25,800,000
25,900,000
26,000,000
Craniofacial superenhancer
CNCC superenhancer
CNCC
25-state
gRNA1
gRNA6
chr7:25,295,587-25,921,144
NPVF
MIR148A
b
Heterozygous deletion
Breakpoint 1 spanning
Control Internal
Breakpoint 6 spanning
Deletion spanning
Hemizygous inversion
Combination 1F/6F
Control Internal
Combination 1R/6R
Deletion spanning
c
Geno3 (deletion-spanning)
1/6 Internal
Bulk
#22 #31 #45 #47
Bulk
#22 #31 #45 #47
Breakpoint 1
Breakpoint 6
Bulk
#22 #31 #45 #47
Bulk
#22 #31 #45 #47
d
1F/6F
1R/6R
#22 #31 #45 #47 #22 #31 #45 #47

Figure S7- a. Location of guide RNAs gRNA1 and gRNA6 relative to the WT orientation. b. Screening strategy for determining whether clones are heterozygous for the 1/6 deletion and determining if a clone contains an inversion of the targeted region. c-d. PCR results identifying heterozygous clones and the clone carrying the hemizygous inversion (#22, also referred to as INV).

Clone 31 Breakpoint 1

```
+Clone_31_1F       AAAAATCATTCTGAAGCATATGGGAGGAGCTGNTTCTACAAACCTGATTTCCCAGCTGCA
                   |||| ||||||||||||||||||||||||||| |||||||||||||||||||||||||||
+Reference_hg19    AAAAGTCATTCTGAAGCATATGGGAGGAGCTGCTTCTACAAACCTGATTTCCCAGCTGCA
-Reference_hg19    TTTTCAGTAAGACTTCGTATACCCTCCTCGACGAAGATGTTTGGACTAAAGGGTCGACGT
                   ||||||||||||||||||||||||||||||||||||||||||||||||||||||||||||
-Clone_31_1R       TTTTCAGTAAGACTTCGTATACCCTCCTCGACGAAGATGTTTGGACTAAAGGGTCGACGT


+Clone_31_1F       CAGTGGGATCAGAGGAGCAGGGCCGGGG-----TGTTCCGCAGAGTCCACCCCCACTCCC
                   ||||||||||||||||||||||||||||     |||||||||||||||||||||||||||
+Reference_hg19    CAGTGGGATCAGAGGAGCAGGGCCGGGGACTTTTGTTCCGCAGAGTCCACCCCCACTCCC
-Reference_hg19    GTCACCCTAGTCTCCTCGTCCCGGCCCCTGAAAACAAGGCGTCTCAGGTGGGGGTGAGGG
                   ||||||||||||||||||||||||||||     |||||||||||||||||||||||||||
-Clone_31_1R       GTCACCCTAGTCTCCTCGTCCCGGCCCC-----ACAAGGCGTCTCAGGTGGGGGTGAGGG


+Clone_31_1F       TATAGAAGGAGAGCTTCCAGGTGGCCTAAGTCAGCAGAATCAAGCAGGGTGCCCCATCCT
                   ||||||||||||||||||||||||||||||||||||||||||||||||||||||||||||
+Reference_hg19    TATAGAAGGAGAGCTTCCAGGTGGCCTAAGTCAGCAGAATCAAGCAGGGTGCCCCATCCT
-Reference_hg19    ATATCTTCCTCTCGAAGGTCCACCGGATTCAGTCGTCTTAGTTCGTCCCACGGGGTAGGA
                   ||||||||||||||||||||||||| |||||||||||||||||||||||||||||||| |
-Clone_31_1R       ATATCTTCCTCTCGAAGGTCCACCGNATTCAGTCGTCTTAGTTCGTCCCACGGGGTAGCA


+Clone_31_1F       GGGCTCCTGGAGACAACAACAGATGGCGGATGGGCAAAGCAAATTAATCAAGCCACCAAA
                   ||||||||||||||||||||||||||||||||||||||||||||||||||||||||||||
+Reference_hg19    GGGCTCCTGGAGACAACAACAGATGGCGGATGGGCAAAGCAAATTAATCAAGCCACCAAA
-Reference_hg19    CCCGAGGACCTCTGTTGTTGTCTACCGCCTACCCGTTTCGTTTAATTAGTTCGGTGGTTT
                   ||||||||||||||||||||||||||||||| || |||||||||||||||||||||||||
-Clone_31_1R       CCCGAGGACCTCTGTTGTTGTCTACCGCCTANCCNTTTCGTTTAATTAGTTCGGTGGTTT


+Clone_31_1F       TGTT
                   ||||
+Reference_hg19    TGTT
-Reference_hg19    ACAA
                   ||||
-Clone_31_1R       ACAA
```

Clone 31 Breakpoint 6

```
+Clone_31_6F       GGGGACCCCCACCTTCCAAACCCCCTCCTGCTTTCCAGTAAGATTTGTANNAAGNNNAGA
                   |||||||||||||||||||||||||||||||||||||||||||||||||    |   |||
+Reference_hg19    GGGGACCCCCACCTTCCAAACCCCCTCCTGCTTTCCAGTAAGATTTGTAGTCTGGTTAGA
-Reference_hg19    CCCCTGGGGGTGGAAGGTTTGGGGGAGGACGAAAGGTCATTCTAAACATCAGACCAATCT
                   ||||||||||||||||||||||||||||||||||||||||||||||||||||||||||||
-Clone_31_6R       CCCCTGGGGGTGGAAGGTTTGGGGGAGGACGAAAGGTCATTCTAAACATCAGACCAATCT

+Clone_31_6F       AGGACCATTTAGATGATGAAAGGAACCAGTGGNCAGCCCCTGATTTATAACCAGTGTGAt
                   |||||||||||||||||||||||||||||||| |||||||||||||||||||||||||||
+Reference_hg19    AGGACCATTTAGATGATGAAAGGAACCAGTGGACAGCCCCTGATTTATAACCAGTGTGAT
-Reference_hg19    TCCTGGTAAATCTACTACTTTCCTTGGTCACCTGTCGGGGACTAAATATTGGTCACACTA
                   ||||||||||||||||||||||||||||||||||||||||||||||||||||||||||||
-Clone_31_6R       TCCTGGTAAATCTACTACTTTCCTTGGTCACCTGTCGGGGACTAAATATTGGTCACACTa
```

```
+Clone_31_6F      tttttttAATATCTACCAAATTAATATTTTTATGTCTGTTCCCCCATTGAGTTGCTTCGG
                  ||||||||||||||||||||||||||||||||||||||||||||||| ||||||||||||
+Reference_hg19   TTTTTTTAATATCTACCAAATTAATATTTTTATGTCTGTTCCCCCATCGAGTTGCTTCGG
-Reference_hg19   AAAAAAATTATAGATGGTTTAATTATAAAAATACAGACAAGGGGGTAGCTCAACGAAGCC
                  ||||||||||||||||||||||||||||||||||||||||||||||| ||||||||||||
-Clone_31_6R      aaaaaaaTTATAGATGGTTTAATTATAAAAATACAGACAAGGGGGTAACTCAACGAAGCC


+Clone_31_6F      TTTGAATTTCAACGAGGGGAGCCTGCCAAGATCGTGTAGTAAAATACGAACTGGATCATA
                  ||||||||||||||||||||||||||||||||||||||||||||||||||||||||||||
+Reference_hg19   TTTGAATTTCAACGAGGGGAGCCTGCCAAGATCGTGTAGTAAAATACGAACTGGATCATA
-Reference_hg19
AAACTTAAAGTTGCTCCCCTCGGACGGTTCTAGCACATCATTTTATGCTTGACCTAGTAT
                  ||||||||||||||||||||||||||||||||||||||||||||||||||||||||||||
-Clone_31_6R      AAACTTAAAGTTGCTCCCCTCGGACGGTTCTAGCACATCATTTTATGCTTGACCTAGTAT

+Clone_31_6F      AATCCCAGCAGGGGTGGGAGAACGTAAGACAGATTGCTAACACCTTaaaaaaaaaagttt
                  ||||||||||||||||||||||||||||||||||||||||||||||||||||||||||||
+Reference_hg19   AATCCCAGCAGGGGTGGGAGAACGTAAGACAGATTGCTAACACCTTAAAAAAAAAAGTTT
-Reference_hg19   TTAGGGTCGTCCCCACCCTCTTGCATTCTGTCTAACGATTGTGGAATTTTTTTTTTCAAA
                  ||||||||||||||||||||||||||||||||||||||||||||||||||||||||||||
-Clone_31_6R      TTAGGGTCGTCCCCACCCTCTTGCATTCTGTCTAACGATTGTGGAAttttttttttcaaa


+Clone_31_6F      aaataaaaaaGGCAGGAATTTTCTTTAAAGTATACCAAGCCCAGCAGGGACTGGGAGATA
                  ||||||||||||||||||||||||||||||||||||||||||||||||||||||||||||
+Reference_hg19   AAATAAAAAAGGCAGGAATTTTCTTTAAAGTATACCAAGCCCAGCAGGGACTGGGAGATA
-Reference_hg19   TTTATTTTTTCCGTCCTTAAAAGAAATTTCATATGGTTCGGGTCGTCCCTGACCCTCTAT
                  ||||||||||||||||||||||||||||||||| || || ||||||||||||||||||||
-Clone_31_6R      ttttattttttCCGTCCTTAAAAGAAATTTCATANGGNTCNGGTCGTCCCTGACCCTCTAT


+Clone_31_6F      CACAAGGCGGCTGAAGGAGGGAGCTA
                  ||||||||||||||||||||||||||
+Reference_hg19   CACAAGGCGGCTGAAGGAGGGAGCTA
-Reference_hg19   GTGTTCCGCCGACTTCCTCCCTCGAT
                  ||||||||||||||||||||||||||
-Clone_31_6R      GTGTTCCGCCGACTTCCTCCCTCGAT
```

Clone 31 across deletion (matching reference on bp1 side) (matching reference on bp6 side)

```
Clone_31_Geno3F  TGACTTCCCNGTATCTAGCTCACNTGTGAACACTCACTATGTTTCTATTGNAAAANGAAT
                 ||||||||| ||||||||||||| |||||||||||||||||||||||||| |||| ||||
Clone_31_Geno3R  TGACTTCCCAGTATCTAGCTCACATGTGAACACTCACTATGTTTCTATTGAAAAATGAAT


Clone_31_Geno3F GGGGCACTTAAGTGTCTGGGTTGGGGGAGCAGGACAAGGGCAAAGATGAGCATCATTTTC
                ||||||||||||||||||||||||||||||||||||||||||||||||||||||||||||
Clone_31_Geno3R GGGGCACTTAAGTGTCTGGGTTGGGGGAGCAGGACAAGGGCAAAGATGAGCATCATTTTC


Clone_31_Geno3F ATGACCTAGTTCAGAAAGCAAAGCTGAGCTTACTTATCAATGGCACTACACACCAAGTTT
                ||||||||||||||||||||||||||||||||||||||||||||||||||||||||||||
Clone_31_Geno3R ATGACCTAGTTCAGAAAGCAAAGCTGAGCTTACTTATCAATGGCACTACACACCAAGTTT


Clone_31_Geno3F GTATAAGGCATTTGGTGAAAAGCTTTGCTCTGAAAAGAATGAGTTAACTCAGCCAAAAAG
                ||||||||||||||||||||||||||||||||||||||||||||||||||||||||||||
Clone_31_Geno3R GTATAAGGCATTTGGTGAAAAGCTTTGCTCTGAAAAGAATGAGTTAACTCAGCCAAAAAG
```

```
Clone_31_Geno3F GGTGGGGCGGGTAGCACAATTCAAATGTGACATTCTACAGTTCAGCTCAAAAGAAGCAAT
                ||||||||||||||||||||||||||||||||||||||||||||||||||||||||||||
Clone_31_Geno3R GGTGGGGCGGGTAGCACAATTCAAATGTGACATTCTACAGTTCAGCTCAAAAGAAGCAAT

Clone_31_Geno3F TGGCTGATTGTCACCAAGTGACCTCACTTTGCTCTGCAGTTATCAGCATGGGAAGATATA
                ||||||||||||||||||||||||||||||||||||||||||||||||||||||||||||
Clone_31_Geno3R TGGCTGATTGTCACCAAGTGACCTCACTTTGCTCTGCAGTTATCAGCATGGGAAGATATA

Clone_31_Geno3F AAACAGGTGAACCTGTGAATGTACACTGGTTTTAAACAGAGATAGAGGATGTAAAAGTCA
                ||||||||||||||||||||||||||||||||||||||||||||||||||||||||||||
Clone_31_Geno3R AAACAGGTGAACCTGTGAATGTACACTGGTTTTAAACAGAGATAGAGGATGTAAAAGTCA

Clone_31_Geno3F TTCTGAAGCATATGGGAGGAGCTGCTTCTACAAACCTGATTTCCCAGCTGCACAGTGGGA
                ||||||||||||||||||||||||||||||||||||||||||||||||||||||||||||
Clone_31_Geno3R TTCTGAAGCATATGGGAGGAGCTGCTTCTACAAACCTGATTTCCCAGCTGCACAGTGGGA

Clone_31_Del_spanning_F TCAGAGGAGCAGGGCCGGGGAACTGGATCATAAATCCCAGCAGGGGTGGGAGAACGTAAG
                        ||||||||||||||||||||||||||||||||||||||||||||||||||||||||||||
Clone_31_Del_spanning_R TCAGAGGAGCAGGGCCGGGGAACTGGATCATAAATCCCAGCAGGGGTGGGAGAACGTAAG

Clone_31_Geno3F ACAGATTGCTAACACCTTaaaaaaaaaaagtttaaataaaaaaGGCAGGAATTTTCTTTAA
                ||||||||||||||||||||||||||||||||||||||||||||||||||||||||||||
Clone_31_Geno3R ACAGATTGCTAACACCTTAAAAAAAAAAGTTTAAATAAAAAAGGCAGGAATTTTCTTTAA

Clone_31_Geno3F AGTATACCAAGCCCAGCAGGGACTGGGAGATACACAAGGCGGCTGAAGGAGGGAGCTACT
                ||||||||||||||||||||||||||||||||||||||||||||||||||||||||||||
Clone_31_Geno3R AGTATACCAAGCCCAGCAGGGACTGGGAGATACACAAGGCGGCTGAAGGAGGGAGCTACT

Clone_31_Geno3F TTACCTTCCCAAGATCACTTTTCCGAGAAGCCCAAACCACAGAAAACCATTCTGGCAGGT
                ||||||||||||||||||||||||||||||||||||| ||||||||||||||||||||||
Clone_31_Geno3R TTACCTTCCCAAGATCACTTTTCCGAGAAGCCCAAANCACAGAAAACCATTCTGGCAGGT

Clone_31_Geno3F AAATCGGGCCCCATGAGCTTTTCATCAAAAGCCAAGGATGTCTCTCTGGGATGTGTGTTA
                |||||||||||||      |||||||||||||||||||||||||||||||||||||||||
Clone_31_Geno3R AAATCGGGCCCCANNNNNNTTTCATCAAAAGCCAAGGATGTCTCTCTGGGATGTGTGTTA

Clone_31_Geno3F GAGATAAGGCAG
                ||||||||||||
Clone_31_Geno3R GAGATAAGGCAG
```

Clone 22

Clone 22 across deletion

Clone 22 deletion-spanning PCR

```
+Clone_22_Geno3F  GACTTCCCAGTATCTAGCTCACATGTGAACACTCACTATGTTTCTATNNNNAANNGAATG
                  |||||||||||||||||||||||||||||||||||||||||||||||    ||  |||||
-Clone_22_Geno3R  GACTTCCCAGTATCTAGCTCACATGTGAACACTCACTATGTTTCTATTGAAAAATGAATG

+Clone_22_Geno3F  GGGCACTTAAGTGTCTGGGTTGGGGGAGCAGGACAAGGGCAAAGATGAGCATCATTTTCA
                  ||||||||||||||||||||||||||||||||||||||||||||||||||||||||||||
-Clone_22_Geno3R  GGGCACTTAAGTGTCTGGGTTGGGGGAGCAGGACAAGGGCAAAGATGAGCATCATTTTCA

+Clone_22_Geno3F  TGACCTAGTTCAGAAAGCAAAGCTGAGCTTACTTATCAATGGCACTACACACCAAGTTTG
                  ||||||||||||||||||||||||||||||||||||||||||||||||||||||||||||
-Clone_22_Geno3R  TGACCTAGTTCAGAAAGCAAAGCTGAGCTTACTTATCAATGGCACTACACACCAAGTTTG

+Clone_22_Geno3F  TATAAGGCATTTGGTGAAAAGCTTTGCTCTGAAAAGAATGAGTTAACTCAGCCAAAAAGG
                  ||||||||||||||||||||||||||||||||||||||||||||||||||||||||||||
-Clone_22_Geno3R  TATAAGGCATTTGGTGAAAAGCTTTGCTCTGAAAAGAATGAGTTAACTCAGCCAAAAAGG

+Clone_22_Geno3F  GTGGGGCGGGTAGCACAATCCAAATGTGACATTCTACAGTTCAGCTCAAAAGAAGCAATT
                  ||||||||||||||||||||||||||||||||||||||||||||||||||||||||||||
-Clone_22_Geno3R  GTGGGGCGGGTAGCACAATCCAAATGTGACATTCTACAGTTCAGCTCAAAAGAAGCAATT

+Clone_22_Geno3F  GGCTGATTGTCACCAAGTGACCTCACTTTGCTCTGCAGTTATCAGCATGGGAAGATATAA
                  ||||||||||||||||||||||||||||||||||||||||||||||||||||||||||||
-Clone_22_Geno3R  GGCTGATTGTCACCAAGTGACCTCACTTTGCTCTGCAGTTATCAGCATGGGAAGATATAA

+Clone_22_Geno3F  AACAGGTGAACCTGTGAATGTACACTGGTTTTAAACAGAGATAGAGGATGTAAAAGTCAT
                  ||||||||||||||||||||||||||||||||||||||||||||||||||||||||||||
-Clone_22_Geno3R  AACAGGTGAACCTGTGAATGTACACTGGTTTTAAACAGAGATAGAGGATGTAAAAGTCAT

+Clone_22_Geno3F  TCTGAAGCATATGGGAGGAGCTGCTTCTACAAACCTGATTTCCCAGCTGCACAGTGGGAT
                  ||||||||||||||||||||||||||||||||||||||||||||||||||||||||||||
-Clone_22_Geno3R  TCTGAAGCATATGGGAGGAGCTGCTTCTACAAACCTGATTTCCCAGCTGCACAGTGGGAT

+Clone_22_Del_spanning_F  CAGAGGAGCAGGGCCGGGGAAACTGGATCATAAATCCCAGCAGGGGTGGGAGAACGTAAG
                          ||||||||||||||||||||||||||||||||||||||||||||||||||||||||||||
-Clone_22_Del_spanning_R  CAGAGGAGCAGGGCCGGGGAAACTGGATCATAAATCCCAGCAGGGGTGGGAGAACGTAAG

+Clone_22_Geno3F  ACAGATTGCTAACACCTTaaaaaaaaaagtttaaataaaaaaGGCAGGAATTTTCTTTAA
                  ||||||||||||||||||||||||||||||||||||||||||||||||||||||||||||
-Clone_22_Geno3R  ACAGATTGCTAACACCTTAAAAAAAAAAGTTTAAATAAAAAAGGCAGGAATTTTCTTTAA

+Clone_22_Geno3F  AGTATACCAAGCCCAGCAGGGACTGGGAGATACACAAGGCGGCTGAAGGAGGGAGCTACT
                  ||||||||||||||||||||||||||||||||||||||||||||||||||||||||||||
-Clone_22_Geno3R  AGTATACCAAGCCCAGCAGGGACTGGGAGATACACAAGGCGGCTGAAGGAGGGAGCTACT

+Clone_22_Geno3F  TTACCTTCCCAAGATCACTTTTCCGAGAAGCCCAAACCACAGAAAACCATTCTGGCAGGT
                  ||||||||||||||||||||||||||||||||||||||||||||||||||||||||||||
-Clone_22_Geno3R  TTACCTTCCCAAGATCACTTTTCCGAGAAGCCCAAACCACAGAAAACCATTCTGGCAGGT

+Clone_22_Geno3F  AAATCGGGCCCCATGAGCTTTTCATCAAAAGCCAAGGATGTCTCTCTGGGATGTGTGTTA
                  ||||||||||||||||   ||  |||||||||||||||||||||||||||||||||  ||
-Clone_22_Geno3R  AAATCGGGCCCCATGANNNTTNNATCAAAAGCCAAGGATGTCTCTCTGGGATGTGTNNTA

+Clone_22_Geno3F  GAGATAAGGCAG
                  ||||||||||||
-Clone_22_Geno3R  GAGATAAGGCAG
```

Clone 22 at inversion

```
Clone_22_1F     nnnnnnnnnn nnnnnnnnnn nnnntggnnn nnnntnnnnc aggngnnnnt  50
+Reference_hg19                       25295459                       T

Clone_22_1F     gtgaatgtac actggtttta aacagagata gnnnnnnnaa aagtcattct  100
+Reference_hg19 GTGAATGTAC ACTGGTTTTA AACAGAGATA GaggatgtAA AAGTCATTCT


Clone_22_1F     gaagcatatg ggaggagctg cttnnncaaa cctgatttcc cagctgcaca  150
+Reference_hg19 GAAGCATATG GGAGGAGCTG CTTctaCAAA CCTGATTTCC CAGCTGCACA

Clone_22_1F     gtgggatcag aggagcaggg ccgggga GT ATTTTACTAC ACGATCTTGG
+Reference_hg19 GTGGGATCAG AGGAGCAGGG CCGGGGAC 25295587
-Reference_hg19                      25921143 CGT ATTTTACTAC ACGATCTTGG

Clone_22_1F     CAGGCTCCCC TCGTTGAAAT TCAAACCGAA GCAACTCaAT GGGGGAACAG  250
-Reference_hg19 CAGGCTCCCC TCGTTGAAAT TCAAACCGAA GCAACTCgAT GGGGGAACAG

Clone_22_1F     ACATAAAAAT ATTAATTTGG TAGATATTAA AAAAAATCAC ACTGGTTATA  300
-Reference_hg19 ACATAAAAAT ATTAATTTGG TAGATATTAA AAAAAATCAC ACTGGTTATA

Clone_22_1F     AATCAGGGGC TGTCCACTGG TTCCTTTCAT CATCTAAATG GTCCTTCTAA  350
-Reference_hg19 AATCAGGGGC TGTCCACTGG TTCCTTTCAT CATCTAAATG GTCCTTCTAA

Clone_22_1F     CCAGACTACA AATCTTACTG GAAAGCAGGA GGGGGTTTGG AAGGTGGGGG  400
-Reference_hg19 CCAGACTACA AATCTTACTG GAAAGCAGGA GGGGGTTTGG AAGGTGGGGG

Clone_22_1F     TCCCCTTCCT GAGGCTTTGG AGACAGCTTA CATCCAACAC TTCCTCAAAC  450
-Reference_hg19 TCCCCTTCCT GAGGCTTTGG AGACAGCTTA CATCCAACAC TTCCTCAAAC

Clone_22_1F     CTGGCTTTGa
-Reference_hg19 CTGGCTTTG 25920862


Clone_22_6F nnnnnnnnnn nntgnnnnnn nnnnnnnnga nnngganccc caaccttnna  50
               agcccctcct gctttccagt aagatttgta gnnnnnnnan aaggaccatt  100
+Ref_hg1925920937CCCCTCCT GCTTTCCAGT AAGATTTGTA GtctggttAg AAGGACCATT

Clone_22_6F tagatgatga aaggaaccag tggacagccc ctgatttata accagtgtga  150
+Ref_hg19   TAGATGATGA AAGGAACCAG TGGACAGCCC CTGATTTATA ACCAGTGTGA

Clone_22_6F tttttttaa tatctaccaa attaatattt ttatgtctgt tccccccattg  200
+Ref_hg19   TTTTTTTTAA TATCTACCAA ATTAATATTT TTATGTCTGT TCCCCCATcG

Clone_22_6F agttgcttcg gtttgaattt caacgagggg agcctgccaa gatcgtgtag  250
+Ref_hg19   AGTTGCTTCG GTTTGAATTT CAACGAGGGG AGCCTGCCAA GATCGTGTAG


Clone_22_6F taaaatacgT CCCCGGCCCT GCTCCTCTGA TCCCACTGTG CAGCTGGGAA  300
+Ref_hg19   TAAAATACG  25921143
-Ref_hg19 25295587  GT CCCCGGCCCT GCTCCTCTGA TCCCACTGTG CAGCTGGGAA

Clone_22_6F  ATCAGGTTTG TAGAAGCAGC TCCTCCCATA TGCTTCAGAA TGACTTTTAC  350
-Ref_hg19    ATCAGGTTTG TAGAAGCAGC TCCTCCCATA TGCTTCAGAA TGACTTTTAC

Clone_22_6F  ATCCTCTATC TCTGTTTAAA ACCAGTGTAC ATTCACAGGT TCACCTGTTT  400
```

```
-Ref_hg19    ATCCTCTATC TCTGTTTAAA ACCAGTGTAC ATTCACAGGT TCACCTGTTT

Clone_22_6F  TATATCTTCC CATGCTGATA ACTGCAGAGC AAAGTGAGGT CACTTGGTGA  450
-Ref_hg19    TATATCTTCC CATGCTGATA ACTGCAGAGC AAAGTGAGGT CACTTGGTGA

Clone_22_6F  CAATCAGCCA A
-Ref_hg19    CAATCAGCCA A 25295385


Clone_22_1R nnnnnnnnnn nnnnnnnnnn nntgnnnnnt aaatatgatg cacagnanca  50
-Ref_hg19   25295806                         T AAATATGATG CACAGtAaCA

Clone_22_1R ntggcggtgt gtaactagta gcgaatttgn nnnnnnnnnn gagaacattt  100
-Ref_hg19   gTGGCGGTGT GTAACTAGTA GCGAATTTGa aaaccaaatctGAGAACATTT

Clone_22_1R ggtggcttga ttaatttgct tcgccnatcc gccatctgtt gttgtctcca  150
-Ref_hg19   GGTGGCTTGA TTAATTTGCT TtGCCcATCC GCCATCTGTT GTTGTCTCCA

Clone_22_1R ggagcccagg atggggcacc ctgcttgatt ctgctgactt aggccacctg  200
-Ref_hg19   GGAGCCCAGG ATGGGGCACC CTGCTTGATT CTGCTGACTT AGGCCACCTG

Clone_22_1R gaagctctcc ttctataggg agtgggggtg gactctgcgg aacaaaagtA  250
-Ref_hg19   GAAGCTCT CCTTCTATAG GGAGTGGGGGTG GACTCTGCGG AACAAAAGT  25295586
+Ref_hg19                                     25921144       A

Clone_22_1R ACTGGATCAT AAATCCCAGC AGGGGTGGGA GAACGTAAGA CAGATTGCTA  300
+Ref_hg19   ACTGGATCA TAAATCCCAG CAGGGGTGGG AGAACGTAAG ACAGATTGCTA

Clone_22_1R ACACCTTAAA AAAAAAAGTT TAAATAAAAA AGGCAGGAAT TTTCTTTAAA  350
+Ref_hg19   ACACCTTAA AAAAAAAAGT TTAAATAAAA AAGGCAGGAA TTTTCTTTAAA

Clone_22_1R GTATACCAAG CCCAGCAGGG ACTGGGAGAT ACACAAGGCG GCTGAAGGAG  400
+Ref_hg19   GTATACCAA GCCCAGCAGG GACTGGGAGA TACACAAGGC GGCTGAAGGAG

Clone_22_1R GGAGCTACTT TACCTTCCCA AGATCACTTT TCCGAGtAGC CCAAACCACA  450
+Ref_hg19   GGAGCTACT TTACCTTCCC AAGATCACTT TTCCGAGaAG CCCAAACCACA

Clone_22_1R GAAAACCATT CTGGCAa
+Ref_hg19   GAAAACCAT TCTGGCA 25921360


Clone_22_6R nnnnnnnnnn nnnnnnnnnn nnntgngnnn gnnagtanct ccctccttca  50
-Ref_h19                            25921303 AGTAgCT CCCTCCTTCA

Clone_22_6R gccgccttgt gtatctccca gtccctgctg gnnttggtat actttaaaga  100
-Ref_h19    GCCGCCTTGT GTATCTCCCA GTCCCTGCTG GgcTTGGTAT ACTTTAAAGA

Clone_22_6R aaattcctgc ctttttttatt taaacttttt tttttaaggt gttagcaatc  150
-Ref_h19    AAATTCCTGC CTTTTTTTATT TAAACTTTTT TTTTTAAGGT GTTAGCAATC

Clone_22_6R tgtcttacgt tctcccaccc ctgctgggat ttatgatcca gttACTTTTG  200
-Ref_h19    TGTCTTACGT TCTCCCACCC CTGCTGGGAT TTATGATCCA GTT        25921144
+Ref_h19                                  25295586       ACTTTTG

Clone_22_6R TTCCGCAGAG TCCACCCCCA CTCCCTATAG AAGGAGAGCT TCCAGGTGGC  250
+Ref_h19    TTCCGCAGAG TCCACCCCCA CTCCCTATAG AAGGAGAGCT TCCAGGTGGC
```

```
Clone_22_6R CTAAGTCAGC AGAATCAAGC AGGGTGCCCC ATCCTGGGCT CCTGGAGACA  300
+Ref_h19    CTAAGTCAGC AGAATCAAGC AGGGTGCCCC ATCCTGGGCT CCTGGAGACA


Clone_22_6R ACAACAGATG GCGGATGGGC AAAGCAAATT AATCAAGCCA CCAAATGTTC  350
+Ref_h19    ACAACAGATG GCGGATGGGC AAAGCAAATT AATCAAGCCA CCAAATGTTC

Clone_22_6R TCAGATTTGG TTTTCAAATT CGCTACTAGT TACACACCGC CACTGTTACT  400
+Ref_h19    TCAGATTTGG TTTTCAAATT CGCTACTAGT TACACACCGC CACTGTTACT

Clone_22_6R GTGCATCATA TTTACTTGAC AGCATTGTCC AGTCCTTAGA TTTCTCTCAC  450
+Ref_h19    GTGCATCATA TTTACTTGAC AGCATTGTCC AGTCCTTAGA TTTCTCTCAC

Clone_22_6R TGGCTCACGC TAAAGAAa
+Ref_h19    TGGCTCACGC TAAAGAA 25295859
```

Figure S8- (Word document) Sequence across breakpoints for clones #31 (heterozygous) and #22 (inversion, INV). Clone sequence based on Sanger sequencing results (see Methods) and compared against hg19 assembly.

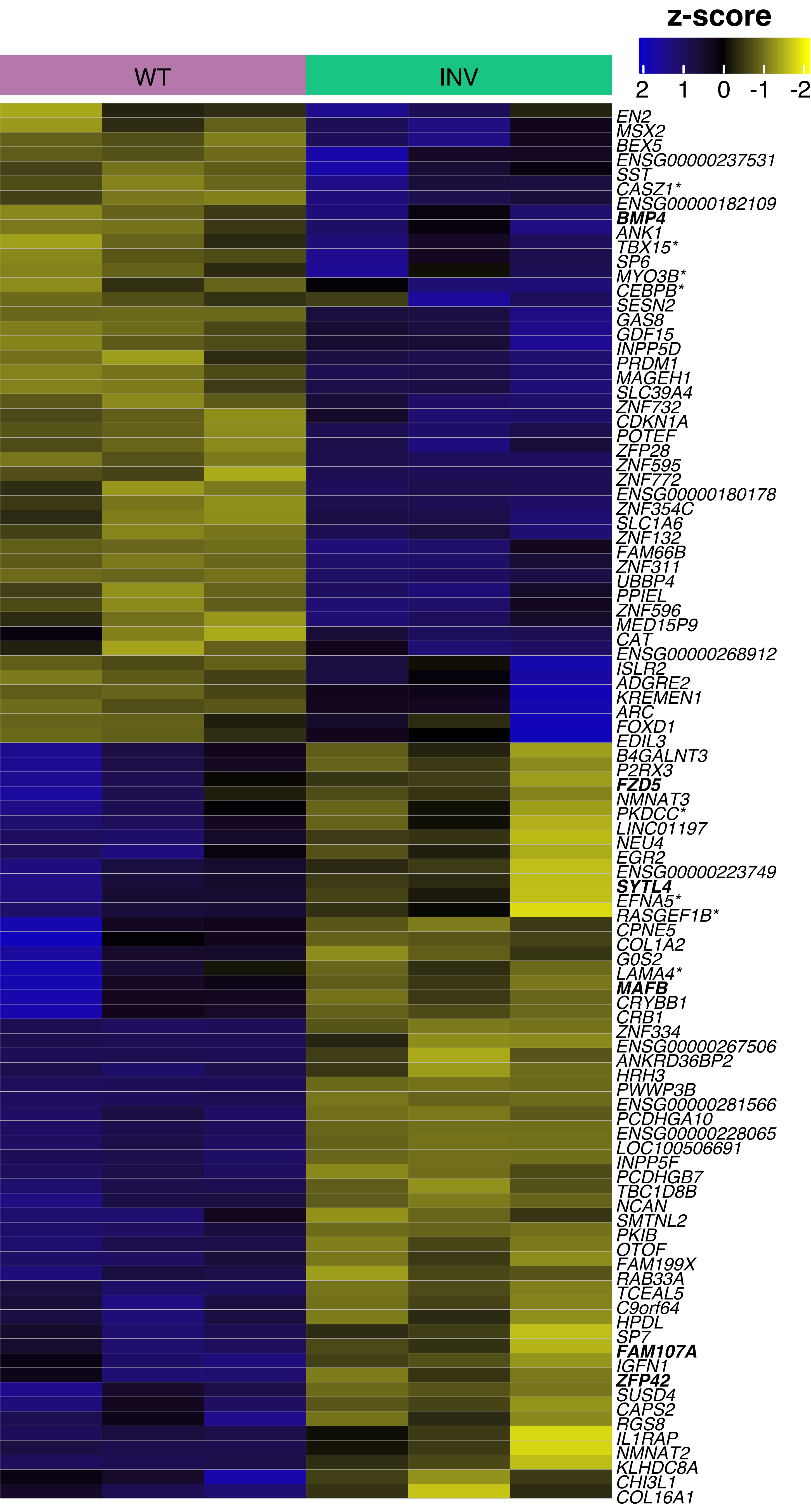
z-score
2
1
0
-1
-2
WT
INV
EN2
MSX2
BEX5
ENSG00000237531
SST
CASZ1*
ENSG00000182109
BMP4
ANK1
TBX15*
SP6
MYO3B*
CEBPB*
SESN2
GAS8
GDF15
INPP5D
PRDM1
MAGEH1
SLC39A4
ZNF732
CDKN1A
POTEF
ZFP28
ZNF595
ZNF772
ENSG00000180178
ZNF354C
SLC1A6
ZNF132
FAM66B
ZNF311
UBBP4
PPIEL
ZNF596
MED15P9
CAT
ENSG00000268912
ISLR2
ADGRE2
KREMEN1
ARC
FOXD1
EDIL3
B4GALNT3
P2RX3
FZD5
NMNAT3
PKDCC*
LINC01197
NEU4
EGR2
ENSG00000223749
SYTL4
EFNA5*
RASGEF1B*
CPNE5
COL1A2
G0S2
LAMA4*
MAFB
CRYBB1
CRB1
ZNF334
ENSG00000267506
ANKRD36BP2
HRH3
PWWP3B
ENSG00000281566
PCDHGA10
ENSG00000228065
LOC100506691
INPP5F
PCDHGB7
TBC1D8B
NCAN
SMTNL2
PKIB
OTOF
FAM199X
RAB33A
TCEAL5
C9orf64
HPDL
SP7
FAM107A
IGFN1
ZFP42
SUSD4
CAPS2
RGS8
IL1RAP
NMNAT2
KLHDC8A
CHI3L1
COL16A1

Figure S9- Heatmap of differentially expressed genes between WT and INV CNCCs. Names followed by asterisks are HOXA2 targets as determined by HOXA2 ChIP-seq (Donaldson et al. 2012). Names in bold are HOXA2 associated genes as determined by RNA-seq from *Hoxa2*-/- mice (Donaldson et al., 2012).

a.

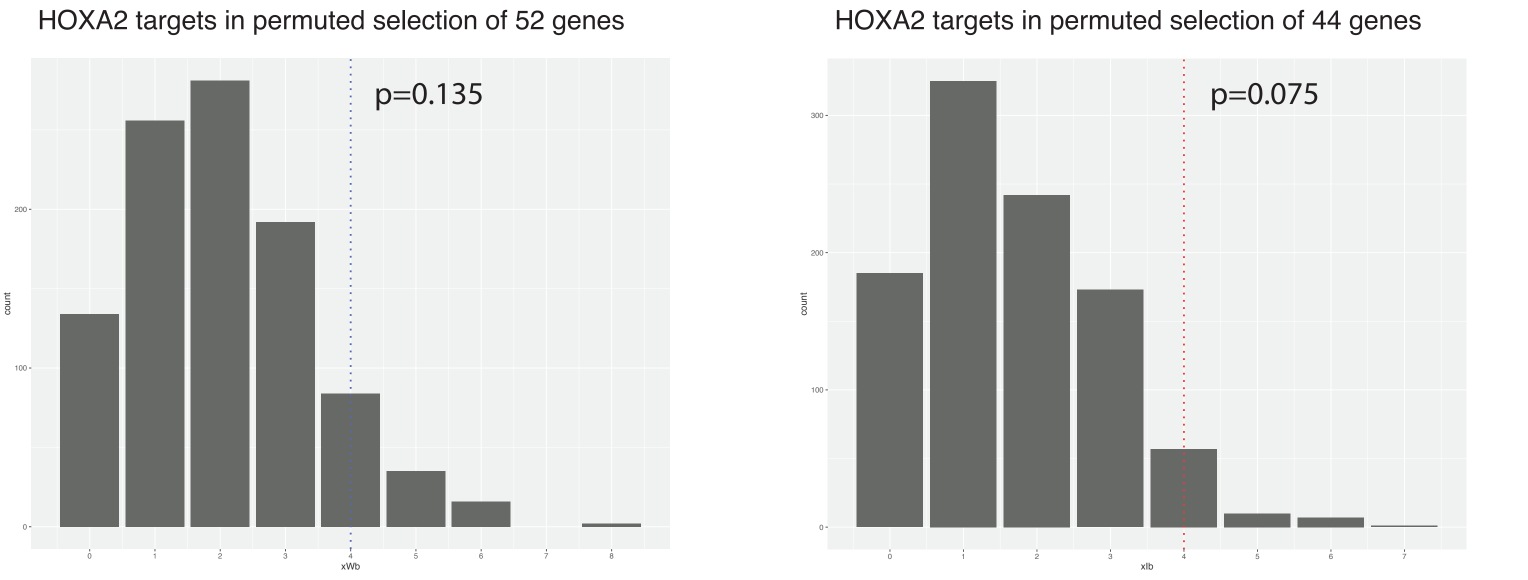
HOXA2 targets in permuted selection of 52 genes
p=0.135
count
xWb
HOXA2 targets in permuted selection of 44 genes
p=0.075
count
xlb

b.

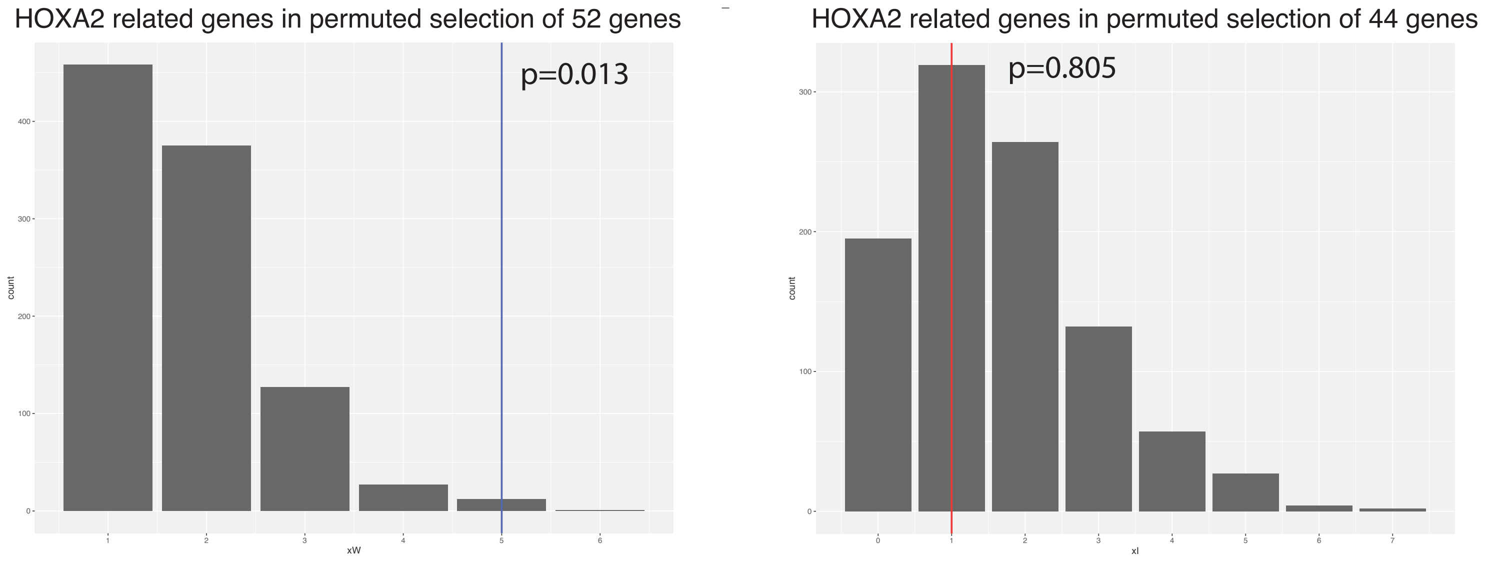
HOXA2 related genes in permuted selection of 52 genes
p=0.013
count
xW
HOXA2 related genes in permuted selection of 44 genes
p=0.805
count
xl

Figure S10- Permutation tests for enrichment of Hoxa2 targets (a) in WT (left, number of *Hoxa2* targets shown as blue dotted line) and inversion (INV) (right, number of *Hoxa2* targets shown as red dotted line). Permutations tests for enrichment of Hoxa2-related genes (b) in WT (left, number of *Hoxa2*-related genes shown as blue solid line) and INV (right, number of *Hoxa2*-related genes shown as red solid line).

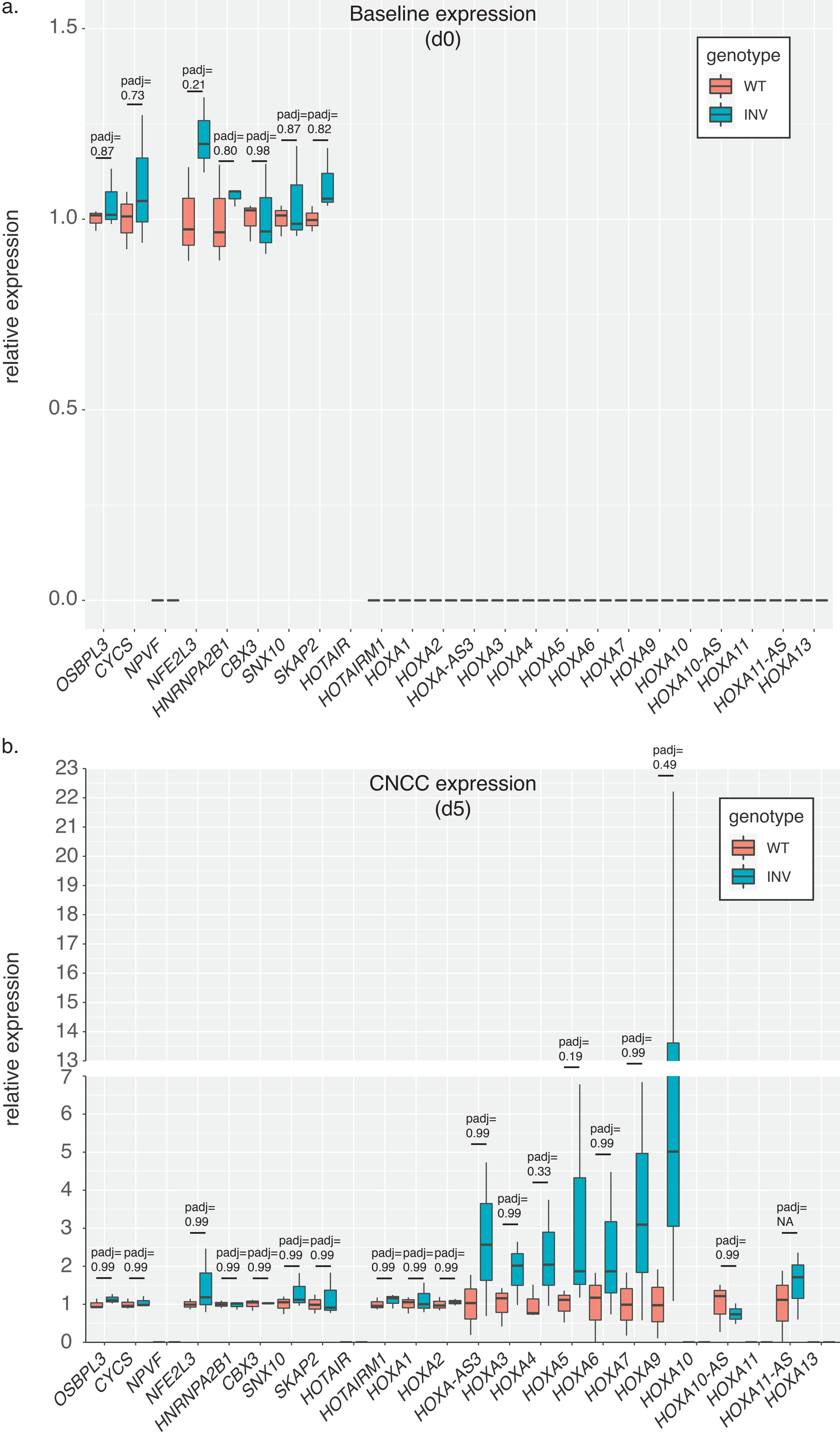
a.
Baseline expression
(d0)
relative expression
1.5
1.0
0.5
0.0
genotype
WT
INV
padj=
0.87
padj=
0.73
padj=
0.21
padj=
0.80
padj=
0.98
padj=
0.87
padj=
0.82
OSBPL3
CYCS
NPVF
NFE2L3
HNRNPA2B1
CBX3
SNX10
SKAP2
HOTAIR
HOTAIRM1
HOXA1
HOXA2
HOXA-AS3
HOXA3
HOXA4
HOXA5
HOXA6
HOXA7
HOXA9
HOXA10
HOXA10-AS
HOXA11
HOXA11-AS
HOXA13
b.
CNCC expression
(d5)
relative expression
23
22
21
20
19
18
17
16
15
14
13
7
6
5
4
3
2
1
0
genotype
WT
INV
padj=
0.99
padj=
0.99
padj=
0.99
padj=
0.99
padj=
0.99
padj=
0.99
padj=
0.99
padj=
0.99
padj=
0.99
padj=
0.99
padj=
0.99
padj=
0.99
padj=
0.33
padj=
0.19
padj=
0.99
padj=
0.99
padj=
0.49
padj=
0.99
padj=
NA
OSBPL3
CYCS
NPVF
NFE2L3
HNRNPA2B1
CBX3
SNX10
SKAP2
HOTAIR
HOTAIRM1
HOXA1
HOXA2
HOXA-AS3
HOXA3
HOXA4
HOXA5
HOXA6
HOXA7
HOXA9
HOXA10
HOXA10-AS
HOXA11
HOXA11-AS
HOXA13

Figure S11- Relative expression of genes in and adjacent to the HOXA cluster at baseline (d0) (a) and after complete CNCC differentiation (d5) (b).

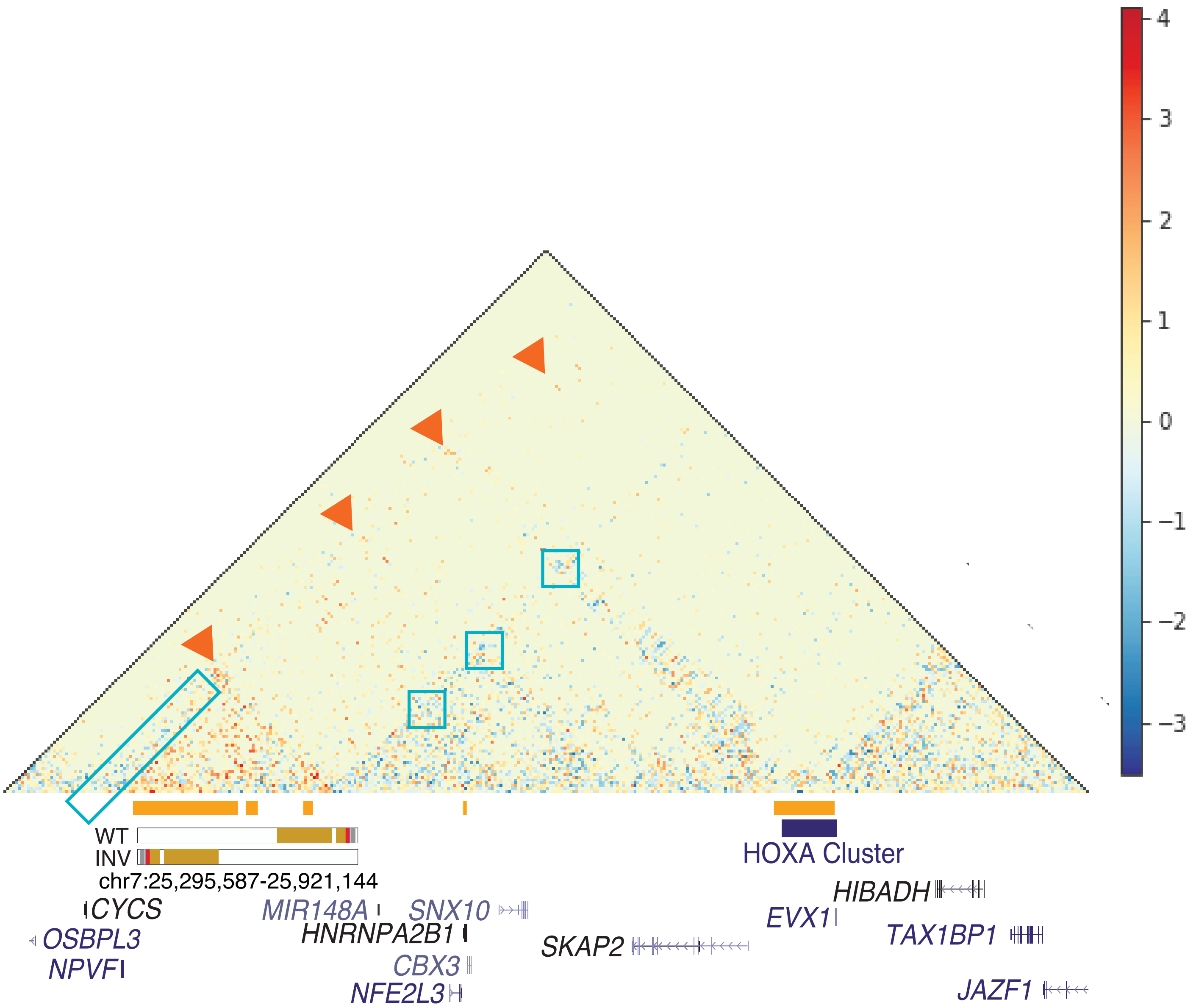

4
3
2
1
0
−1
−2
−3
WT
INV
chr7:25,295,587-25,921,144
HOXA Cluster
CYCS
OSBPL3
NPVF
MIR148A
HNRNPA2B1
SNX10
CBX3
NFE2L3
SKAP2
EVX1
HIBADH
TAX1BP1
JAZF1

Figure S12- Subtraction HiC map generated by comparison of WT CNCC HiC data and INV CNCC HiC data on custom genome accounting for inversion. Representations of the WT and inverted superenhancer region are displayed below the triangular heatmap. Light blue boxes show areas of stronger interaction in the WT CNCCs, red triangles show novel interactions created by the superenhancer inversion.

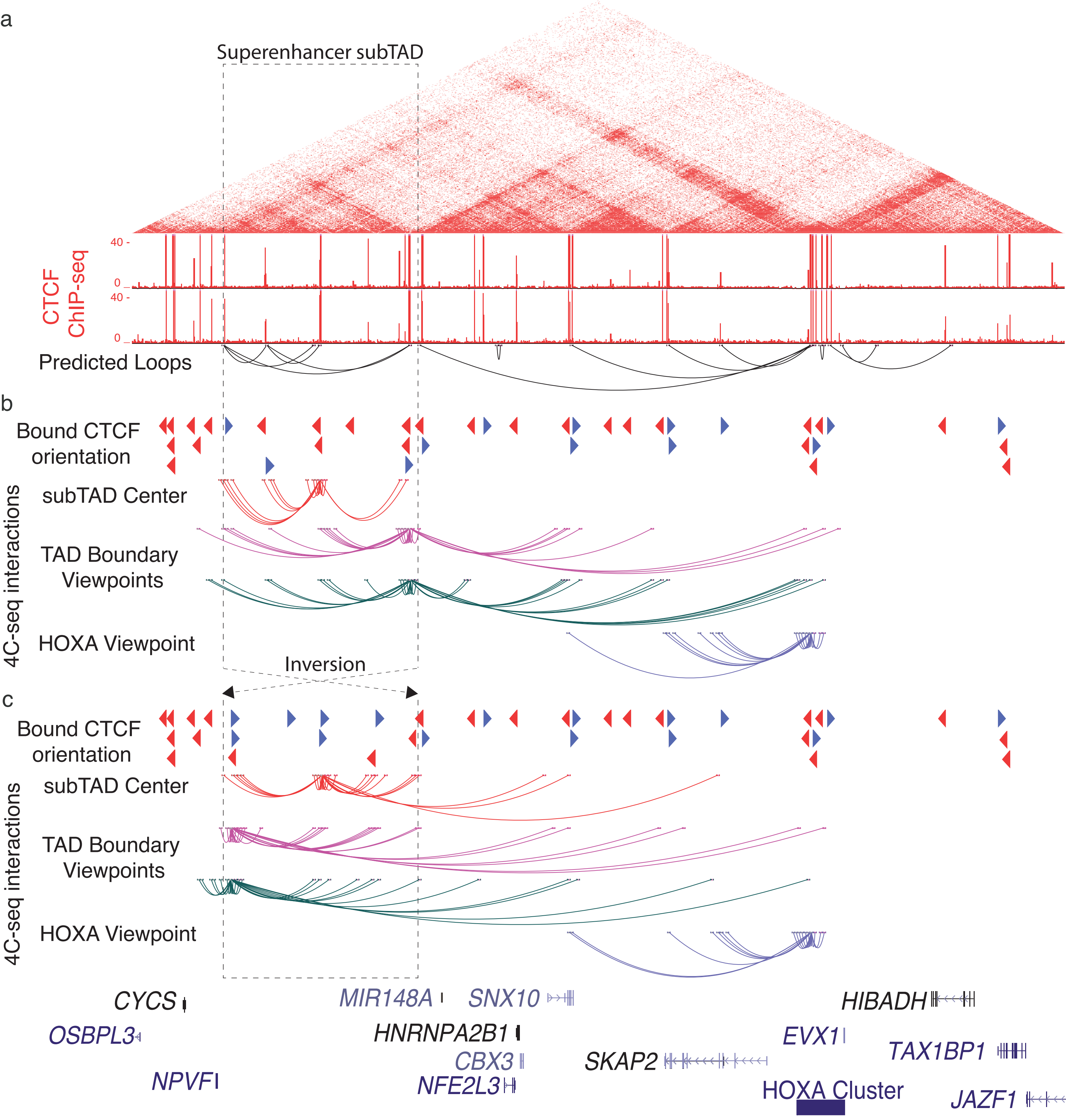
a
Superenhancer subTAD
40 -
0
40 -
0
CTCF ChIP-seq
Predicted Loops
b
Bound CTCF orientation
subTAD Center
TAD Boundary Viewpoints
HOXA Viewpoint
4C-seq interactions
Inversion
c
Bound CTCF orientation
subTAD Center
TAD Boundary Viewpoints
HOXA Viewpoint
4C-seq interactions
CYCS
MIR148A
SNX10
HIBADH
OSBPL3
HNRNPA2B1
EVX1
TAX1BP1
CBX3
SKAP2
NPVF
NFE2L3
HOXA Cluster
JAZF1

Figure S13- Schematic showing consequences of superenhancer inversion on orientation of CTCF sites and boundary strength. Previously insulated 4C viewpoints are capable of interaction with *HOXA* cluster following creation of inversion and viewpoints at the TAD boundary capable of interacting with the *HOXA* cluster in the WT configuration retain contacts following inversion.

a.

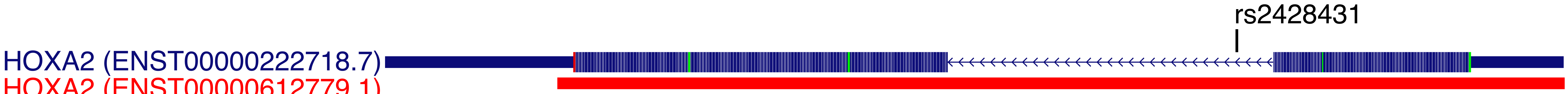

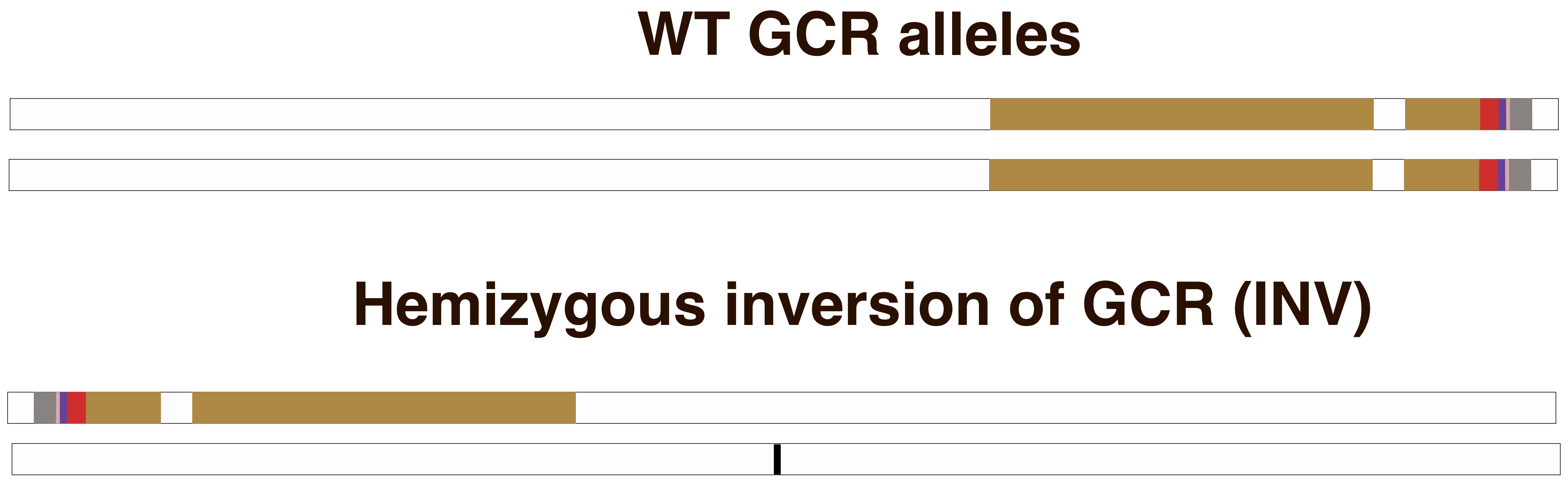

b.

rs2428431 C/G (%) [counts]

WT n=3: (50%/50%) [3/3]
(50%/50%) [5/5]
(45%/55%) [5/6]

INV n=3: (17%/83%) [2/10]
(33%/67%) [2/4]
(21%/79%) [4/15]

Figure S14- IGV browser shot showing C/G allele ratios in WT CNCCs, hemizygous inversion (INV) CNCCs and WT, INV and Heterozygous superenhancer deletion lines in which the TAD boundary of both alleles (WT) or the remaining allele was also deleted.

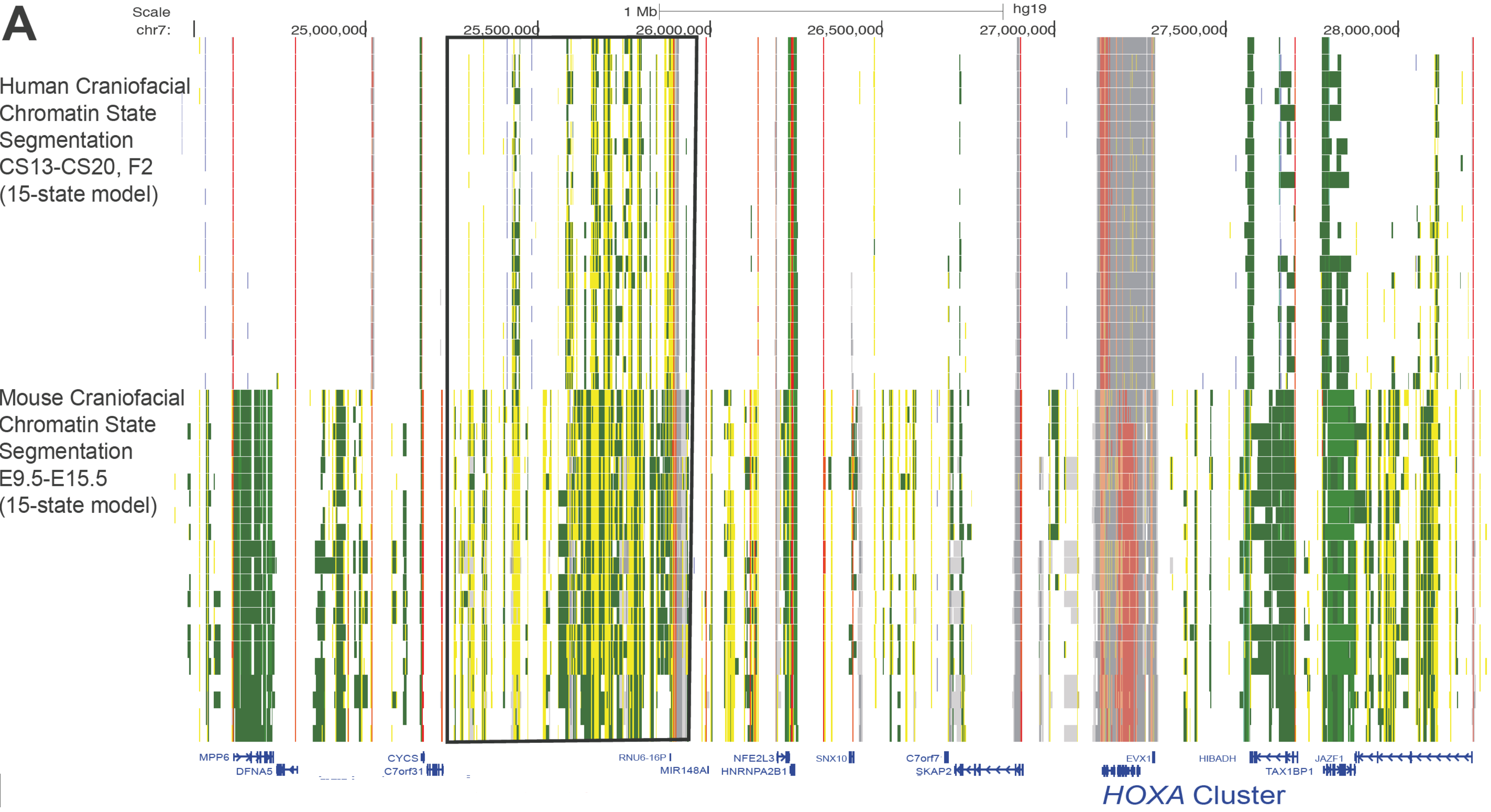
A
Scale
chr7:
1 Mb
hg19
25,000,000
25,500,000
26,000,000
26,500,000
27,000,000
27,500,000
28,000,000
Human Craniofacial Chromatin State Segmentation CS13-CS20, F2 (15-state model)
Mouse Craniofacial Chromatin State Segmentation E9.5-E15.5 (15-state model)
MPP6
DFNA5
CYCS
C7orf31
RNU6-16P
MIR148A
NFE2L3
HNRNPA2B1
SNX10
C7orf7
SKAP2
EVX1
HIBADH
TAX1BP1
JAZF1
HOXA Cluster

Figure S15- Comparison of chromatin states in the 15-state model between human embryonic craniofacial tissue (hg19) and mouse embryonic craniofacial tissue (mm9 lifted to hg19).

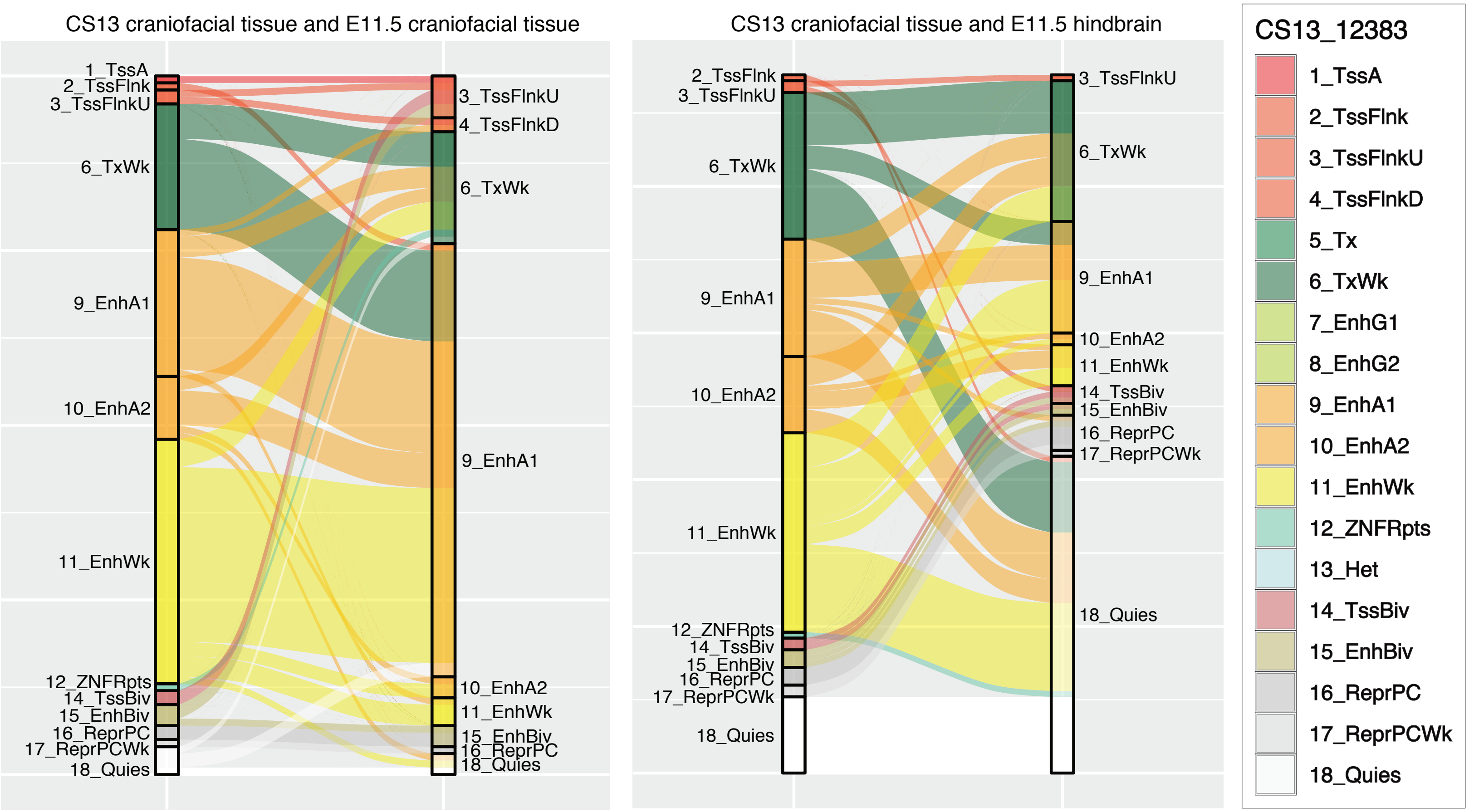

CS13 craniofacial tissue and E11.5 craniofacial tissue
1_TssA
2_TssFlnk
3_TssFlnkU
6_TxWk
9_EnhA1
10_EnhA2
11_EnhWk
12_ZNFRpts
14_TssBiv
15_EnhBiv
16_ReprPC
17_ReprPCWk
18_Quies
3_TssFlnkU
4_TssFlnkD
6_TxWk
9_EnhA1
10_EnhA2
11_EnhWk
15_EnhBiv
16_ReprPC
18_Quies
CS13 craniofacial tissue and E11.5 hindbrain
2_TssFlnk
3_TssFlnkU
6_TxWk
9_EnhA1
10_EnhA2
11_EnhWk
12_ZNFRpts
14_TssBiv
15_EnhBiv
16_ReprPC
17_ReprPCWk
18_Quies
3_TssFlnkU
6_TxWk
9_EnhA1
10_EnhA2
11_EnhWk
14_TssBiv
15_EnhBiv
16_ReprPC
17_ReprPCWk
18_Quies
CS13_12383
1_TssA
2_TssFlnk
3_TssFlnkU
4_TssFlnkD
5_Tx
6_TxWk
7_EnhG1
8_EnhG2
9_EnhA1
10_EnhA2
11_EnhWk
12_ZNFRpts
13_Het
14_TssBiv
15_EnhBiv
16_ReprPC
17_ReprPCWk
18_Quies

Figure S16- Chromatin state composition comparison for 18-state model between CS13 craniofacial tissue and E11.5 craniofacial tissue or E11.5 hindbrain. Mouse chromatin states lifted from mm10 to hg19 using liftOver.

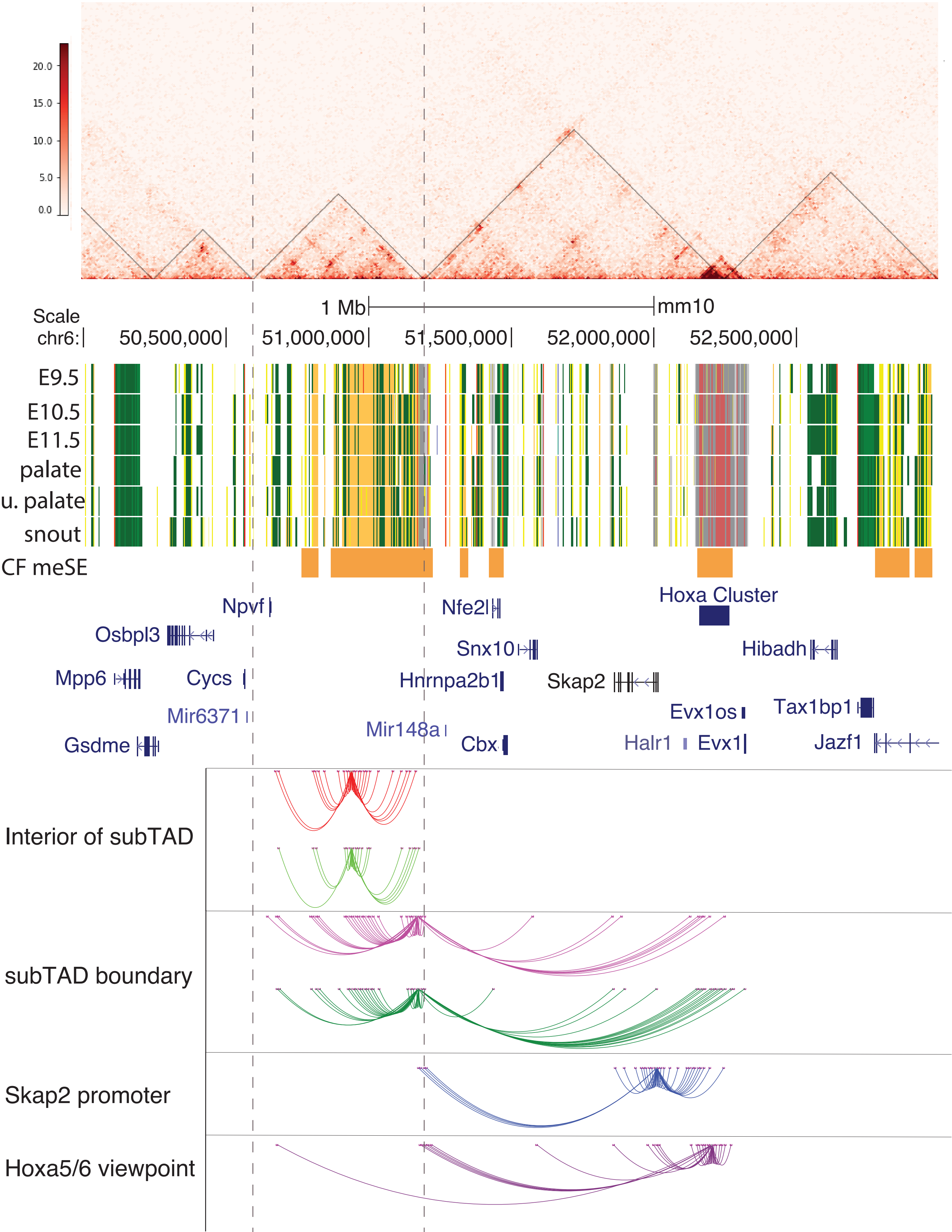

20.0
15.0
10.0
5.0
0.0
1 Mb
mm10
Scale
chr6:
50,500,000
51,000,000
51,500,000
52,000,000
52,500,000
E9.5
E10.5
E11.5
palate
u. palate
snout
CF meSE
Npvf
Nfe2l
Hoxa Cluster
Osbpl3
Snx10
Hibadh
Mpp6
Cycs
Hnrnpa2b1
Skap2
Evx1os
Tax1bp1
Mir6371
Mir148a
Cbx
Halr1
Evx1
Jazf1
Gsdme
Interior of subTAD
subTAD boundary
Skap2 promoter
Hoxa5/6 viewpoint

Figure S17- HiC data from WT E11.5 mouse craniofacial tissue, TADs or subTADs determined at 50Kb resolution. The superenhancer subTAD is marked throughout with dotted lines (Top). Chromatin states in 18-state model and mouse embryonic craniofacial superenhancers shown in orange bars above gene notations (Middle). Interactions identified though 4C-seq at all viewpoints tested (Bottom). Viewpoint1(red) and Viewpoint 2 (light green) are located within the interior of the superenhancer subTAD. Viewpoint 3 (magenta) and Viewpoint 4 (dark green) are located at the boundary of the subTAD. Viewpoint 5 (dark blue) is located near the *Skap2* promoter and Viewpoint 6 (purple) is located near the intergenic space between *Hoxa5* and *Hoxa6*.

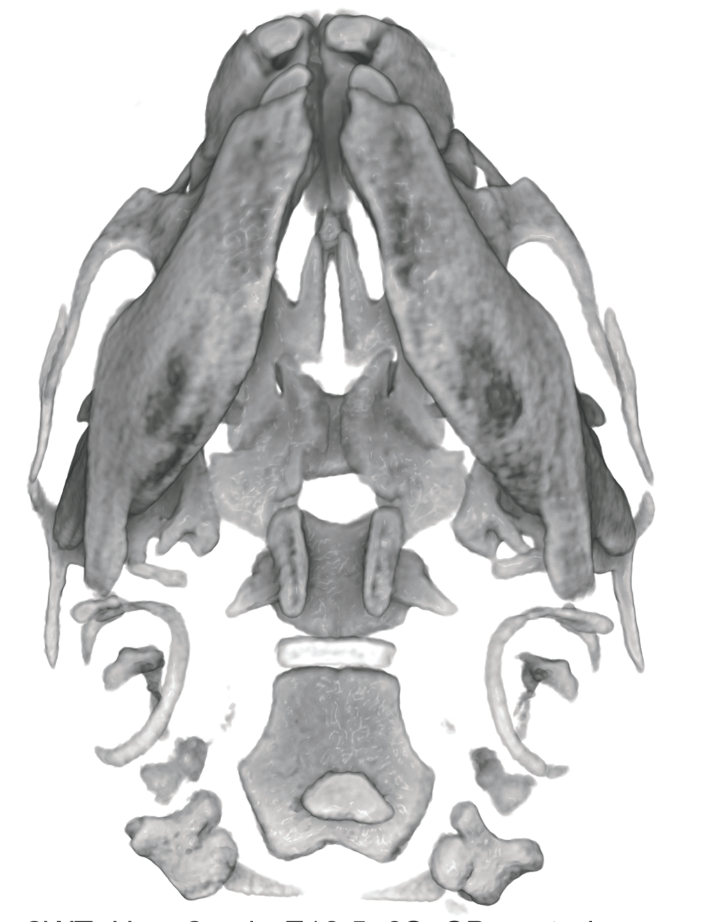

2WT_Hoxa2 enh_E18.5_0S_CB-ventral.png

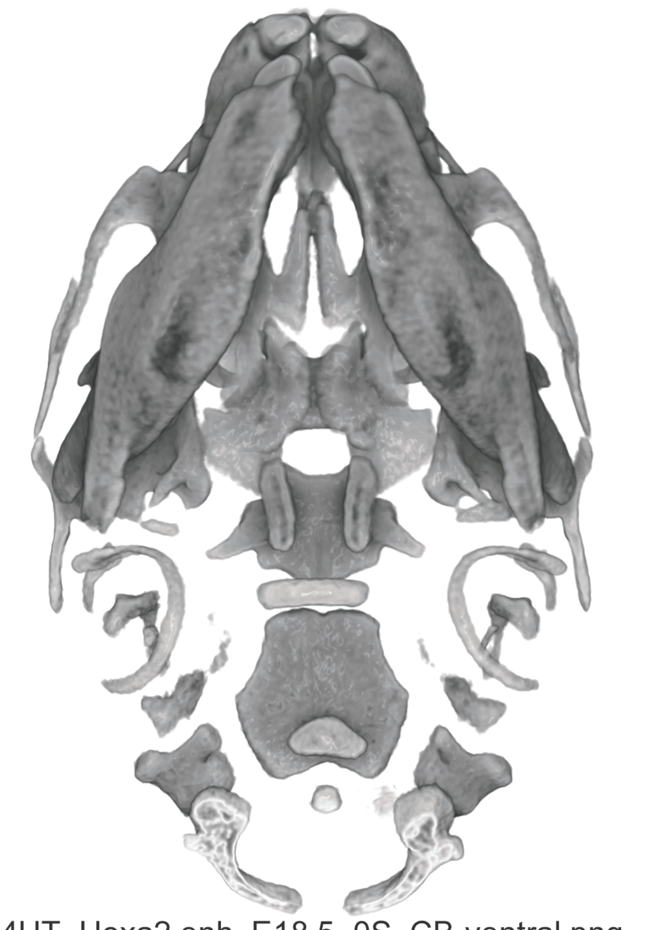

4HT_Hoxa2 enh_E18.5_0S_CB-ventral.png

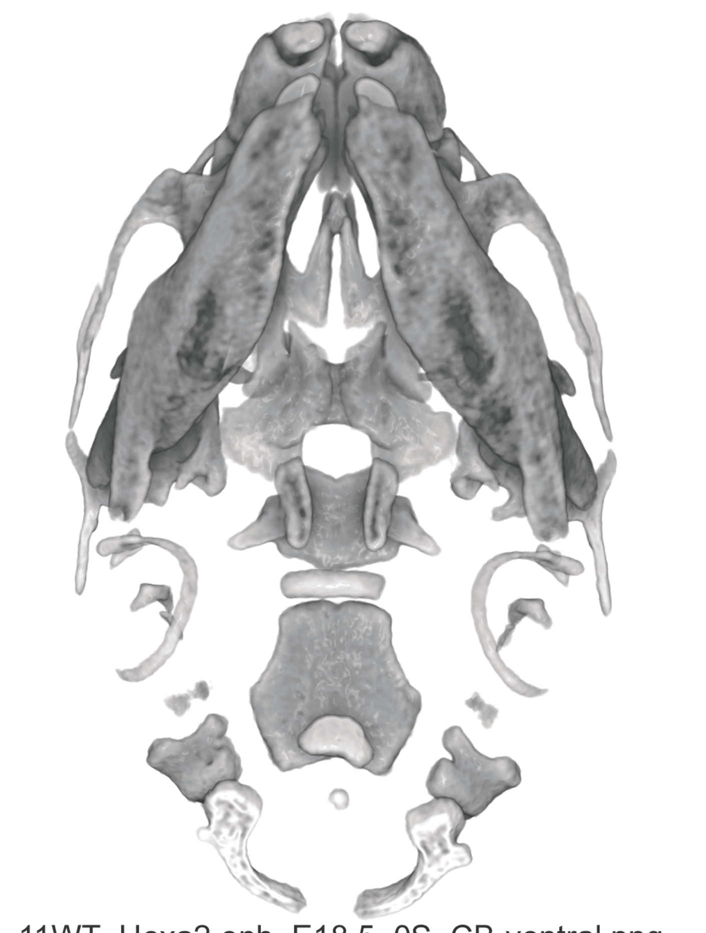

11WT_Hoxa2 enh_E18.5_0S_CB-ventral.png

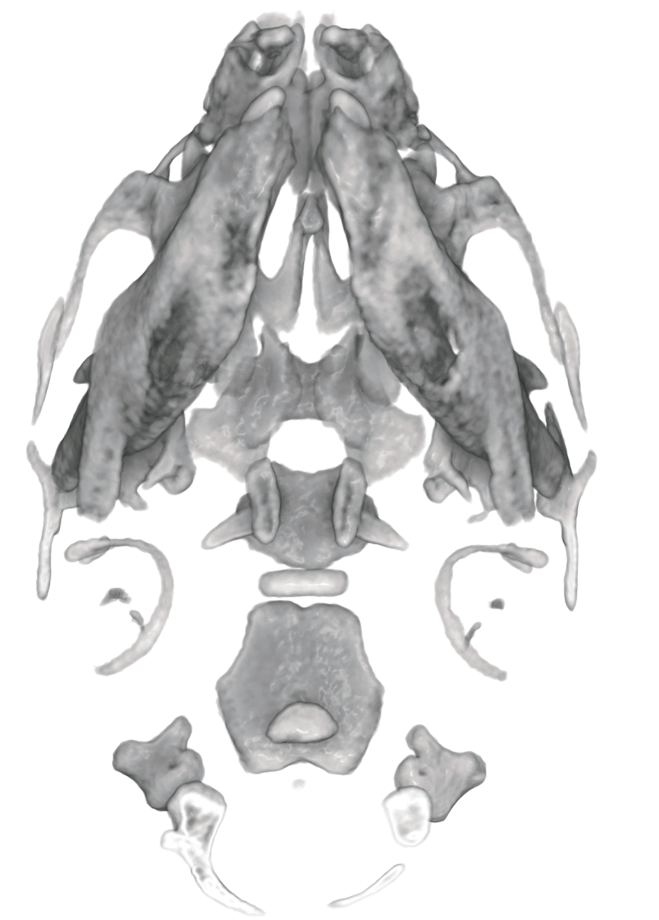

15HT_Hoxa2 enh_E18.5_0S_CB-ventral.png

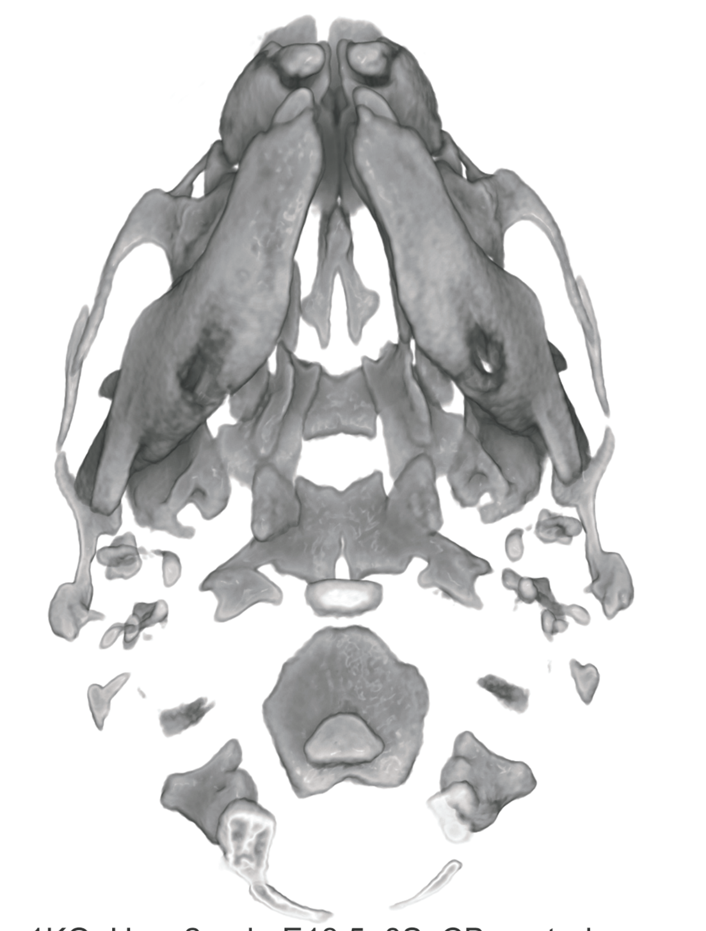

1KO_Hoxa2 enh_E18.5_0S_CB-ventral.png

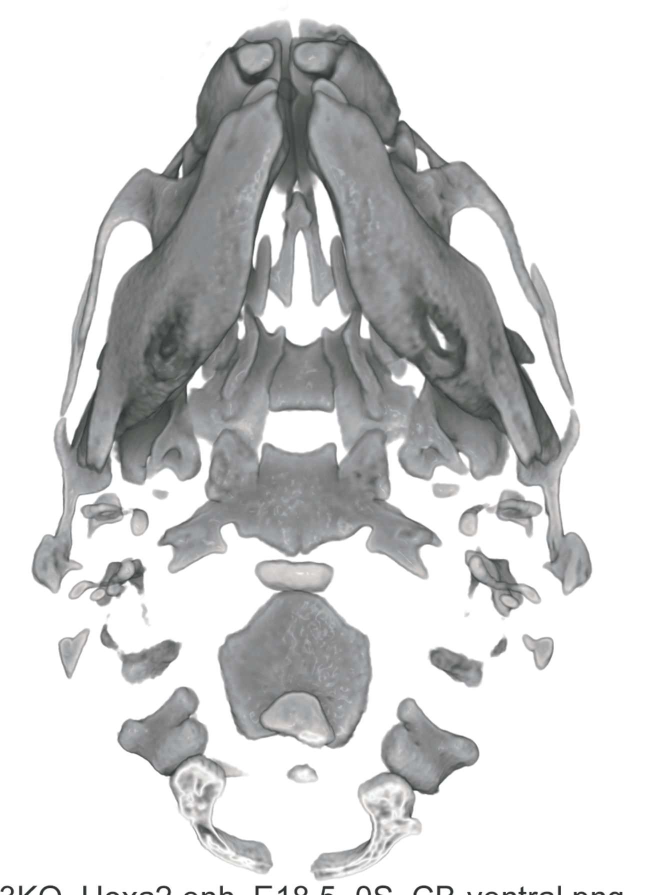

3KO_Hoxa2 enh_E18.5_0S_CB-ventral.png

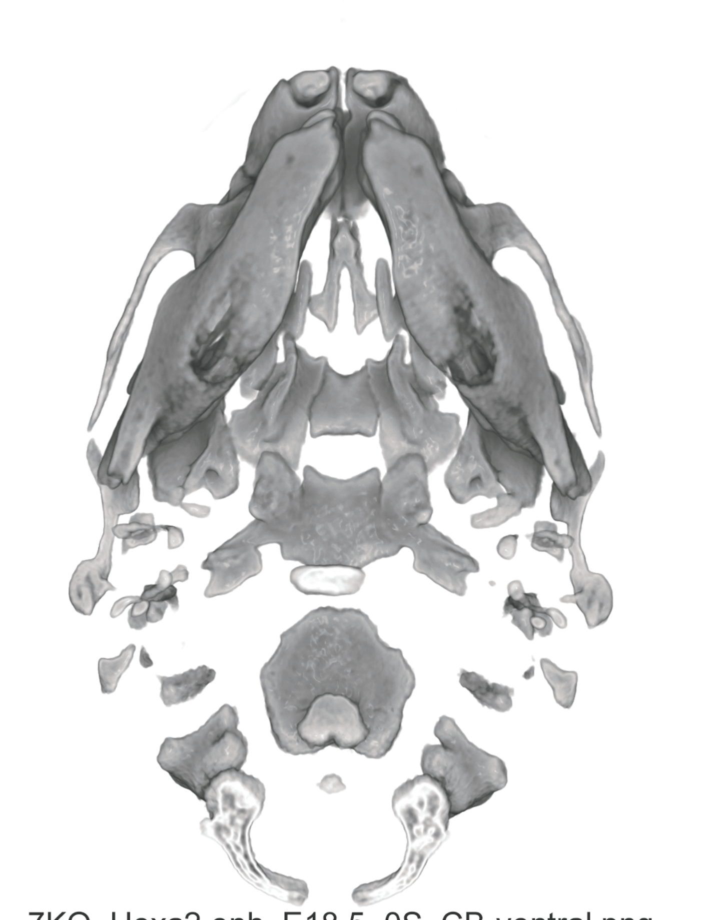

7KO_Hoxa2 enh_E18.5_0S_CB-ventral.png

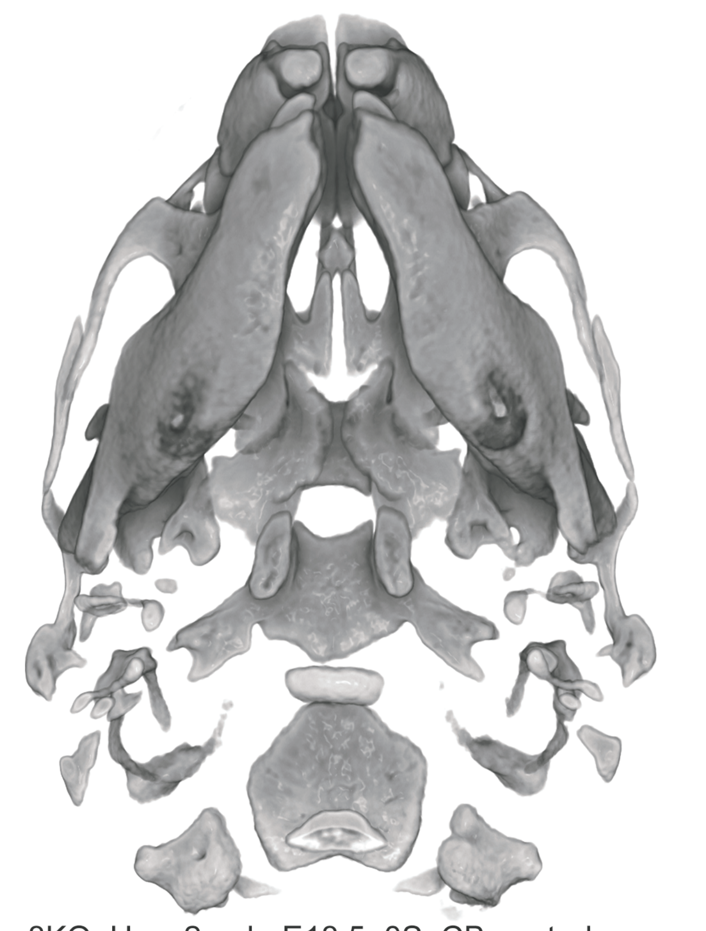

8KO_Hoxa2 enh_E18.5_0S_CB-ventral.png

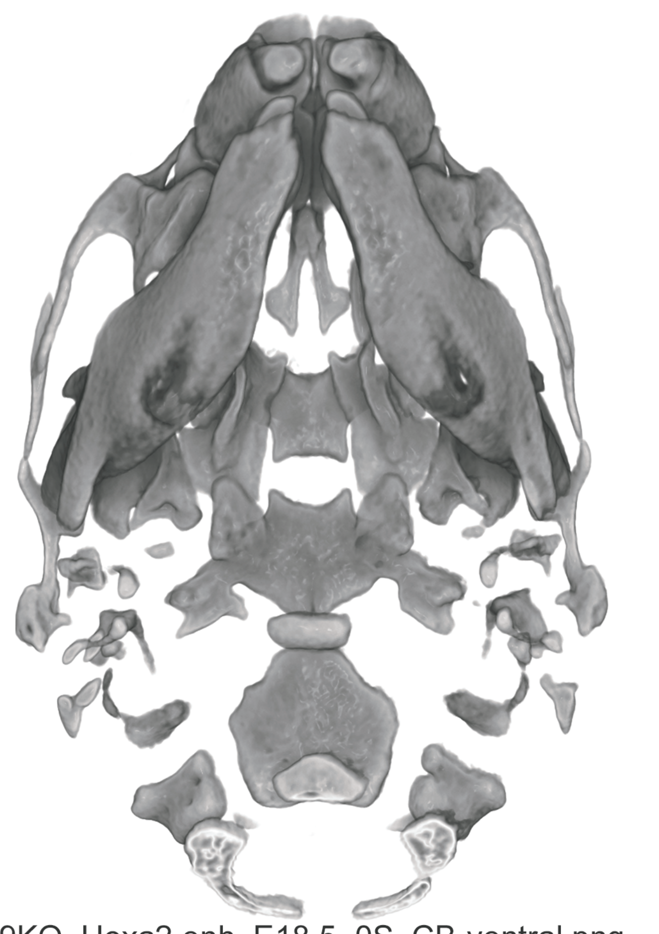

9KO_Hoxa2 enh_E18.5_0S_CB-ventral.png

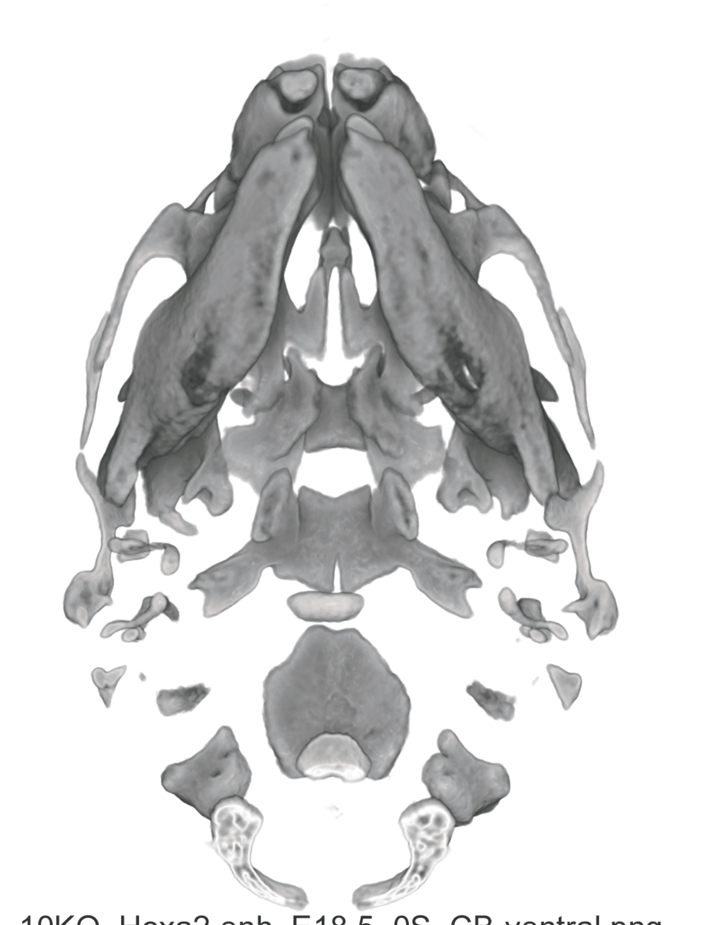

10KO_Hoxa2 enh_E18.5_0S_CB-ventral.png

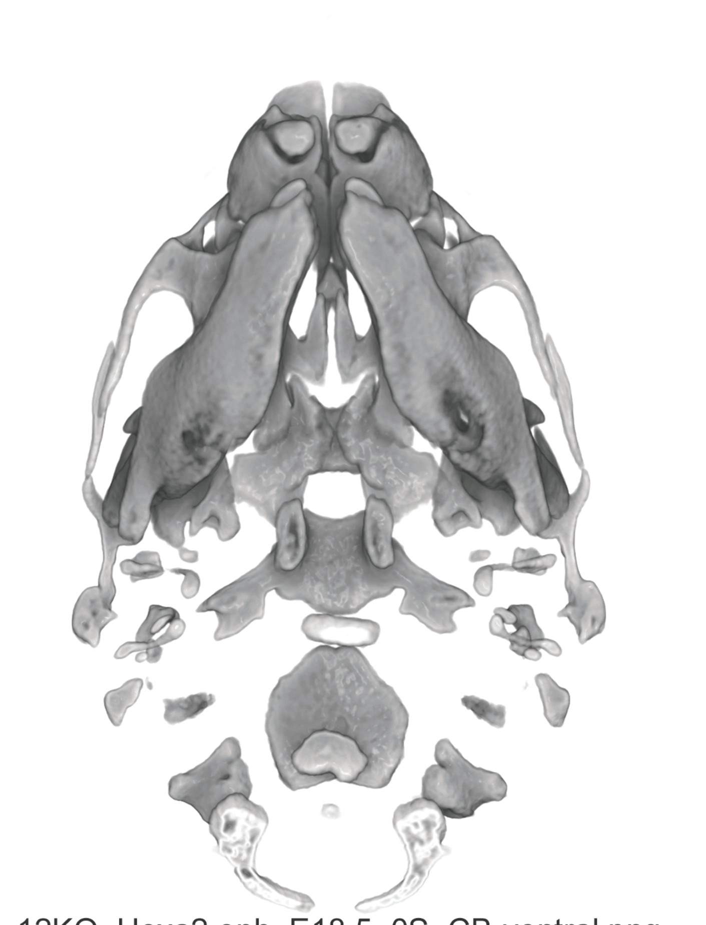

12KO_Hoxa2 enh_E18.5_0S_CB-ventral.png

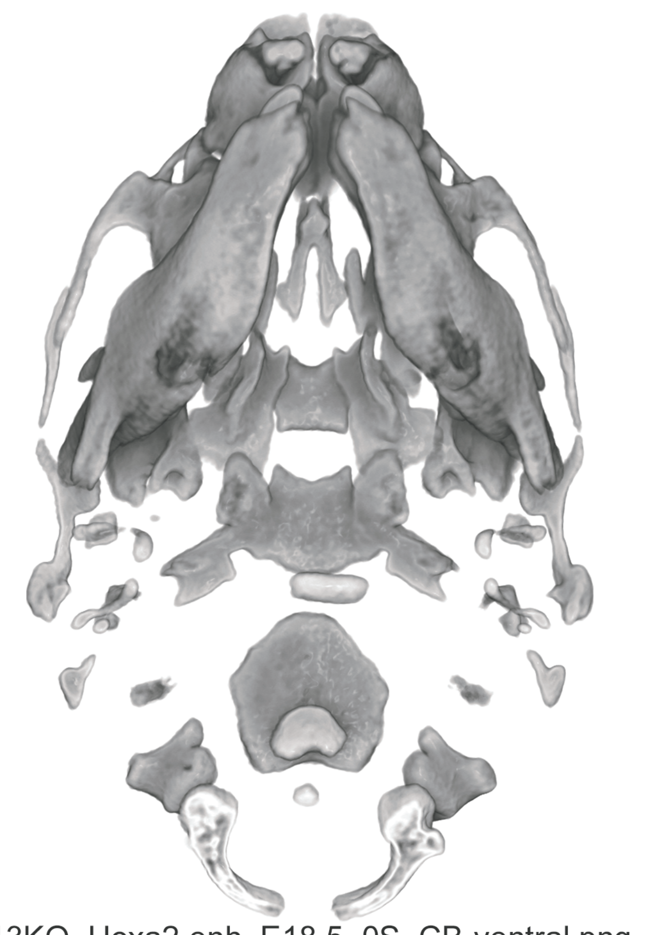

13KO_Hoxa2 enh_E18.5_0S_CB-ventral.png

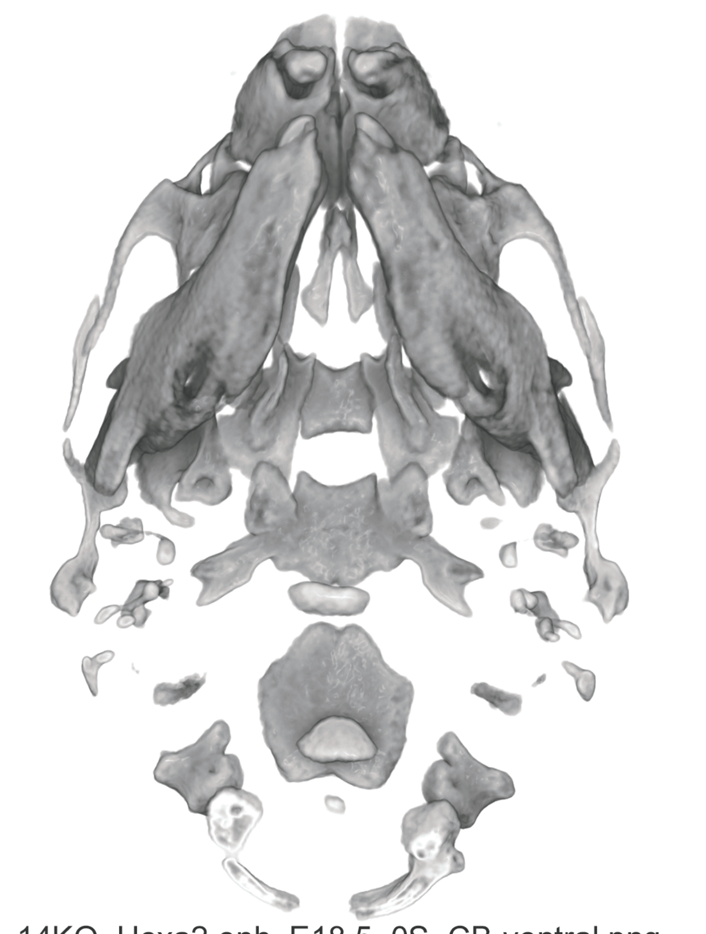

14KO_Hoxa2 enh_E18.5_0S_CB-ventral.png

Figure S18- microCT renderings of skulls from all WT, Hoxa+/ΔGCR (HT), and Hoxa ΔGCR /ΔGCR (KO) E18.5 embryos.

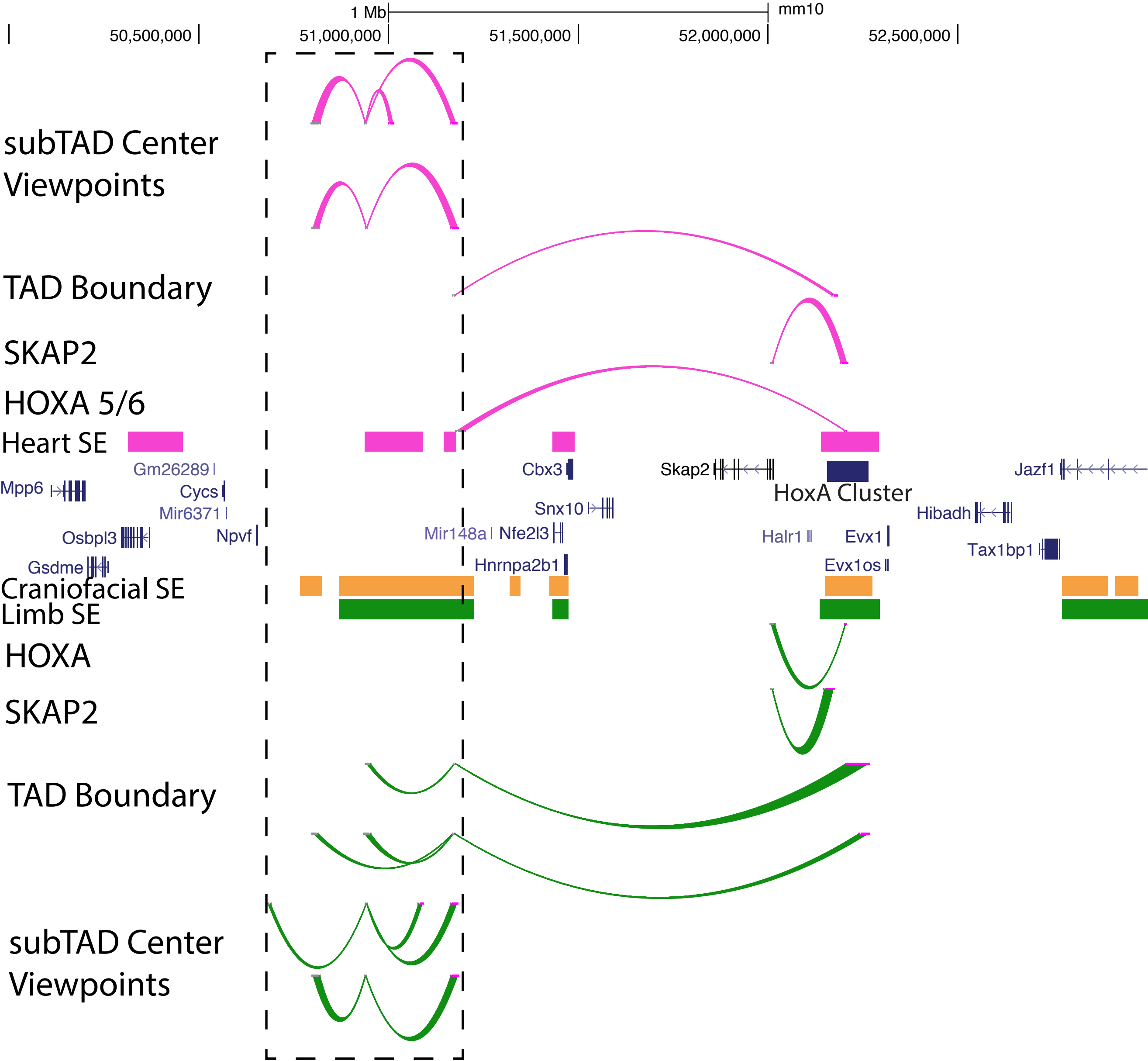

1 Mb
mm10
50,500,000
51,000,000
51,500,000
52,000,000
52,500,000
subTAD Center Viewpoints
TAD Boundary
SKAP2
HOXA 5/6
Heart SE
Gm26289
Mpp6
Cycs
Mir6371
Osbpl3
Npvf
Gsdme
Cbx3
Skap2
Jazf1
HoxA Cluster
Snx10
Hibadh
Mir148a
Nfe2l3
Halr1
Evx1
Tax1bp1
Hnrnpa2b1
Evx1os
Craniofacial SE
Limb SE
HOXA
SKAP2
TAD Boundary
subTAD Center Viewpoints

Figure S19 4C-Seq data from E11.5 mouse heart and limb. The superenhancer subTAD is marked throughout with dotted lines. Same viewpoints depicted in Figure S17 were used for each tissue. Interactions identified though 4C-seq are indicated by loops for each viewpoint in heart (pink) and limb (green). Colored bars indicate superenhancer calls in respective tissues.

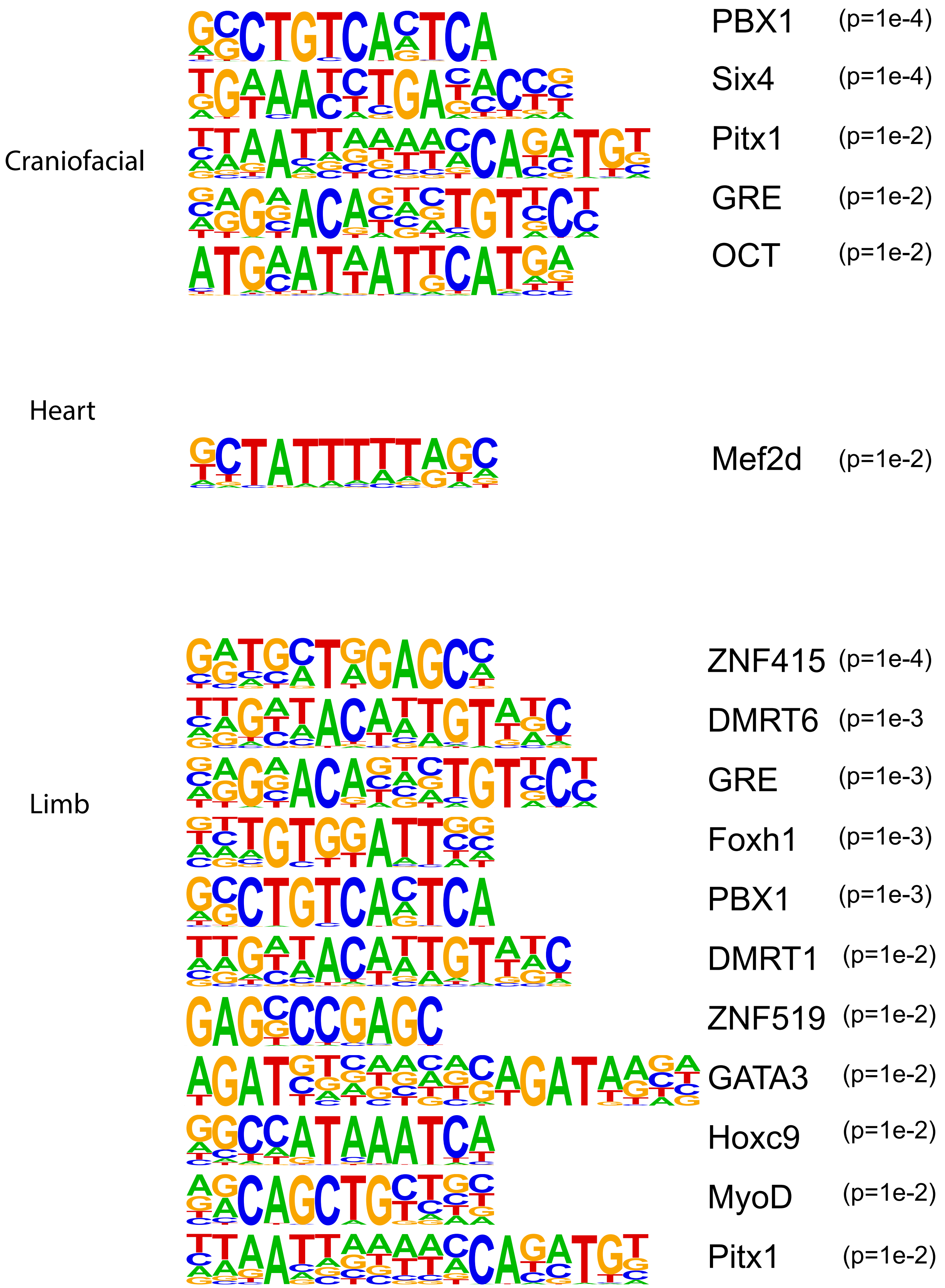

Craniofacial
PBX1 (p=1e-4)
Six4 (p=1e-4)
Pitx1 (p=1e-2)
GRE (p=1e-2)
OCT (p=1e-2)
Heart
Mef2d (p=1e-2)
Limb
ZNF415 (p=1e-4)
DMRT6 (p=1e-3
GRE (p=1e-3)
Foxh1 (p=1e-3)
PBX1 (p=1e-3)
DMRT1 (p=1e-2)
ZNF519 (p=1e-2)
GATA3 (p=1e-2)
Hoxc9 (p=1e-2)
MyoD (p=1e-2)
Pitx1 (p=1e-2)

Figure S20. Significantly enriched TF binding motifs in enhancer modules encompassed by superenhancer calls in the chromosome 7 gene desert.

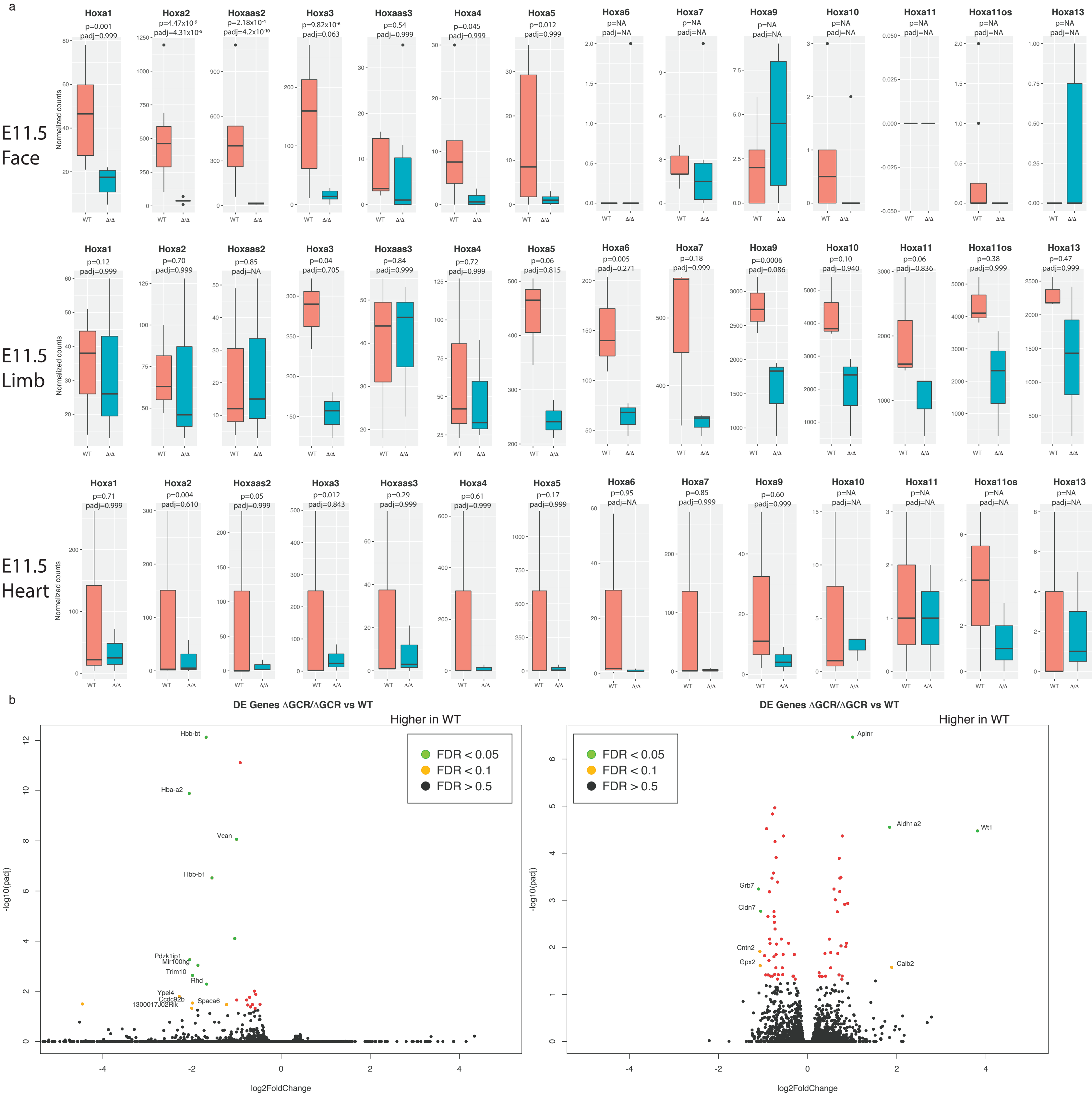
a
E11.5 Face
Hoxa1 p=0.001 padj=0.999
Hoxa2 p=4.47x10-9 padj=4.31x10-5
Hoxaas2 p=2.18x10-4 padj=4.2x10-10
Hoxa3 p=9.82x10-6 padj=0.063
Hoxaas3 p=0.54 padj=0.999
Hoxa4 p=0.045 padj=0.999
Hoxa5 p=0.012 padj=0.999
Hoxa6 p=NA padj=NA
Hoxa7 p=NA padj=NA
Hoxa9 p=NA padj=NA
Hoxa10 p=NA padj=NA
Hoxa11 p=NA padj=NA
Hoxa11os p=NA padj=NA
Hoxa13 p=NA padj=NA
E11.5 Limb
Hoxa1 p=0.12 padj=0.999
Hoxa2 p=0.70 padj=0.999
Hoxaas2 p=0.85 padj=NA
Hoxa3 p=0.04 padj=0.705
Hoxaas3 p=0.84 padj=0.999
Hoxa4 p=0.72 padj=0.999
Hoxa5 p=0.06 padj=0.815
Hoxa6 p=0.005 padj=0.271
Hoxa7 p=0.18 padj=0.999
Hoxa9 p=0.0006 padj=0.086
Hoxa10 p=0.10 padj=0.940
Hoxa11 p=0.06 padj=0.836
Hoxa11os p=0.38 padj=0.999
Hoxa13 p=0.47 padj=0.999
E11.5 Heart
Hoxa1 p=0.71 padj=0.999
Hoxa2 p=0.004 padj=0.610
Hoxaas2 p=0.05 padj=0.999
Hoxa3 p=0.012 padj=0.843
Hoxaas3 p=0.29 padj=0.999
Hoxa4 p=0.61 padj=0.999
Hoxa5 p=0.17 padj=0.999
Hoxa6 p=0.95 padj=NA
Hoxa7 p=0.85 padj=0.999
Hoxa9 p=0.60 padj=0.999
Hoxa10 p=NA padj=NA
Hoxa11 p=NA padj=NA
Hoxa11os p=NA padj=NA
Hoxa13 p=NA padj=NA
Normalized counts
WT
Δ/Δ
b
DE Genes ΔGCR/ΔGCR vs WT
Higher in WT
FDR < 0.05
FDR < 0.1
FDR > 0.5
Hbb-bt
Hba-a2
Vcan
Hbb-b1
Pdzk1ip1
Mir100hg
Trim10
Rhd
Ypel4
Ccdc92b
1300017J02Rik
Spaca6
-log10(padj)
log2FoldChange
DE Genes ΔGCR/ΔGCR vs WT
Higher in WT
FDR < 0.05
FDR < 0.1
FDR > 0.5
Aplnr
Aldh1a2
Wt1
Grb7
Cldn7
Cntn2
Gpx2
Calb2
-log10(padj)
log2FoldChange

Figure S21- Comparison of *Hoxa* gene expression in embryonic face, limb and heart (a). Gene expression changes in heart (b-left panel) and limb (b-right panel). Few significant genes change as a result of the deletion, and did not include the *Hoxa* genes in either heart or limb.

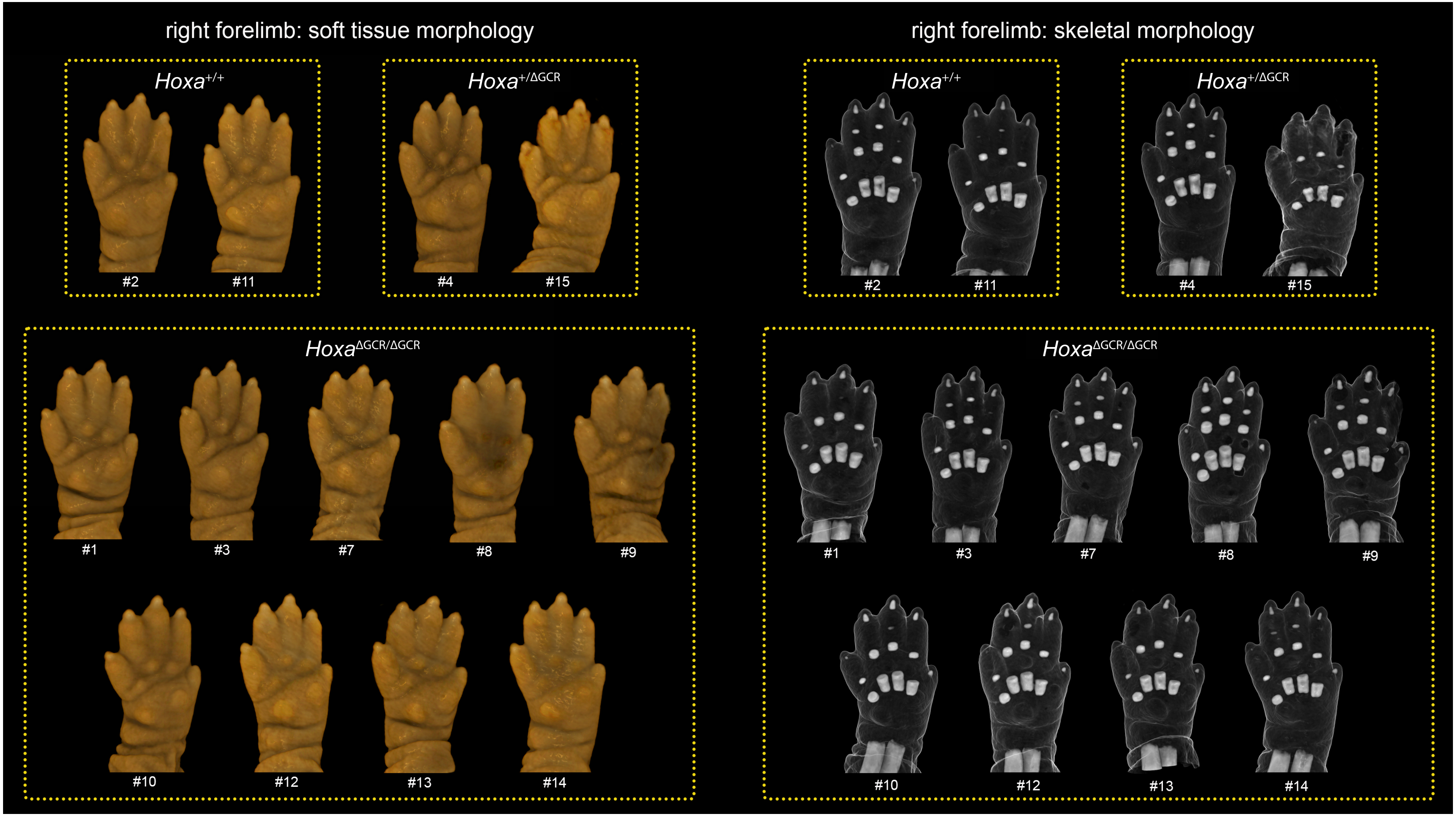
right forelimb: soft tissue morphology
right forelimb: skeletal morphology
Hoxa+/+
Hoxa+/ΔGCR
HoxaΔGCR/ΔGCR
#2
#11
#4
#15
#1
#3
#7
#8
#9
#10
#12
#13
#14

Figure S22. Soft tissue and microCT renderings of limbs from all WT, *Hoxa*$^{+/\Delta GCR,}$ and *Hoxa* $^{\Delta GCR / \Delta GCR}$ E18.5 embryos. Genotypes are indicated in each dashed box and embryos are matched by number across rendering types.

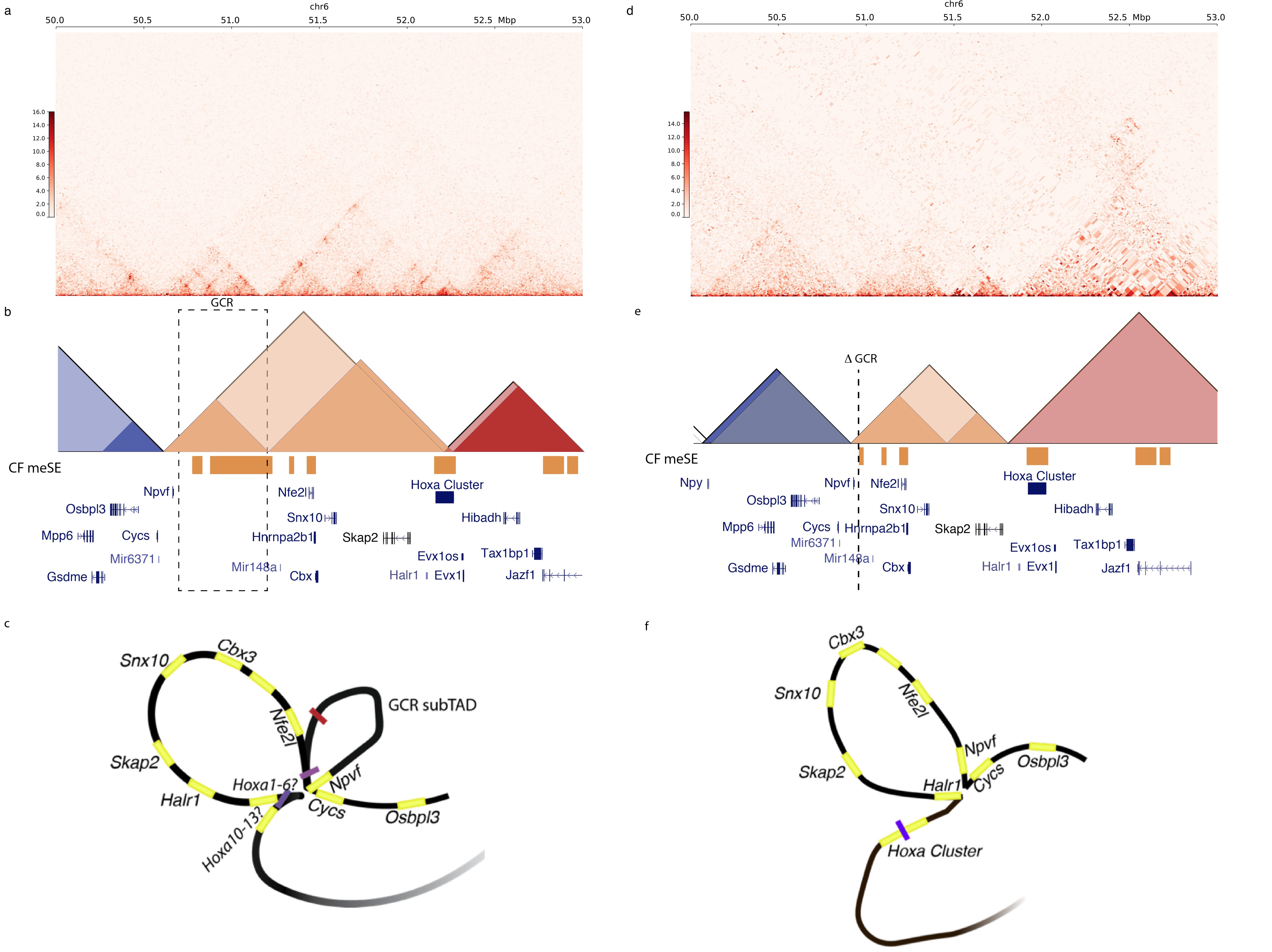
a
chr6
50.0
50.5
51.0
51.5
52.0
52.5 Mbp
53.0
b
GCR
CF meSE
Npvf
Osbpl3
Mpp6
Cycs
Mir6371
Gsdme
Nfe2l
Snx10
Hnrnpa2b1
Mir148a
Cbx
Skap2
Hoxa Cluster
Hibadh
Evx1os
Tax1bp1
Halr1
Evx1
Jazf1
c
Snx10
Cbx3
Nfe2l
GCR subTAD
Skap2
Hoxa1-6?
Npvf
Halr1
Cycs
Osbpl3
Hoxa10-13?
d
chr6
e
Δ GCR
CF meSE
Npy
Npvf
Nfe2l
Hoxa Cluster
Osbpl3
Snx10
Hibadh
Mpp6
Cycs
Hnrnpa2b1
Skap2
Mir6371
Evx1os
Tax1bp1
Mir148a
Gsdme
Cbx
Halr1
Evx1
Jazf1
f
Cbx3
Snx10
Nfe2l
Npvf
Osbpl3
Skap2
Halr1
Cycs
Hoxa Cluster

Figure S23- a. Deletion of GCR re-structures the *HoxA* regulatory landscape. The removal of ~625kb and almost complete removal of the superenhancers in the gene desert between *Npvf* and *Mir148a* disrupts the contact between the GCR and the *HoxA* cluster. Schematics based on HiC of E11.5 cranofacial tissue from WT (top panel) and ΔGCR/ΔGCR mice (bottom panel). The bottom panel was created by alignment to a custom genome based on mm10 with deletion of the GCR coordinates. Presentation of superenhancers in the bottom panel are predicted, based on the superenhancers as they appear in the WT. TADs predicted at 100Kb are represented with black outline, TADs predicted at 50Kb have no outline.

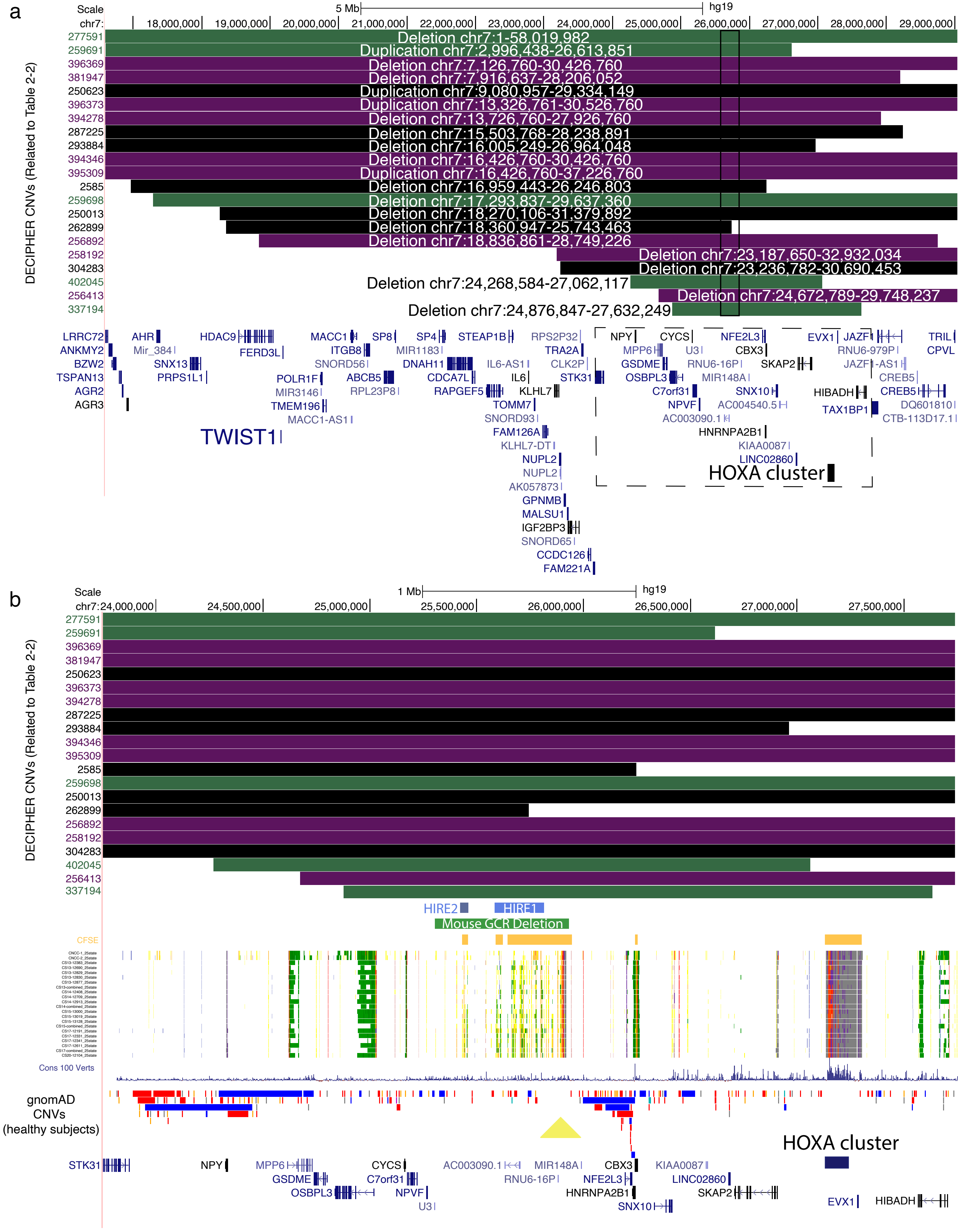

a
Scale
5 Mb
hg19
chr7:
18,000,000
19,000,000
20,000,000
21,000,000
22,000,000
23,000,000
24,000,000
25,000,000
26,000,000
27,000,000
28,000,000
29,000,000
DECIPHER CNVs (Related to Table 2-2)
277591 Deletion chr7:1-58,019,982
259691 Duplication chr7:2,996,438-26,613,851
396369 Deletion chr7:7,126,760-30,426,760
381947 Deletion chr7:7,916,637-28,206,052
250623 Duplication chr7:9,080,957-29,334,149
396373 Duplication chr7:13,326,761-30,526,760
394278 Deletion chr7:13,726,760-27,926,760
287225 Deletion chr7:15,503,768-28,238,891
293884 Deletion chr7:16,005,249-26,964,048
394346 Deletion chr7:16,426,760-30,426,760
395309 Duplication chr7:16,426,760-37,226,760
2585 Deletion chr7:16,959,443-26,246,803
259698 Deletion chr7:17,293,837-29,637,360
250013 Deletion chr7:18,270,106-31,379,892
262899 Deletion chr7:18,360,947-25,743,463
256892 Deletion chr7:18,836,861-28,749,226
258192 Deletion chr7:23,187,650-32,932,034
304283 Deletion chr7:23,236,782-30,690,453
402045 Deletion chr7:24,268,584-27,062,117
256413 Deletion chr7:24,672,789-29,748,237
337194 Deletion chr7:24,876,847-27,632,249
LRRC72
ANKMY2
BZW2
TSPAN13
AGR2
AGR3
AHR
Mir_384
SNX13
PRPS1L1
HDAC9
FERD3L
TWIST1
MACC1
ITGB8
SNORD56
POLR1F
MIR3146
TMEM196
MACC1-AS1
SP8
ABCB5
RPL23P8
SP4
MIR1183
DNAH11
CDCA7L
RAPGEF5
STEAP1B
IL6-AS1
IL6
KLHL7
TOMM7
SNORD93
FAM126A
KLHL7-DT
NUPL2
NUPL2
AK057873
GPNMB
MALSU1
IGF2BP3
SNORD65
CCDC126
FAM221A
RPS2P32
TRA2A
CLK2P
STK31
NPY
MPP6
GSDME
OSBPL3
C7orf31
NPVF
AC003090.1
CYCS
U3
RNU6-16P
MIR148A
AC004540.5
HNRNPA2B1
KIAA0087
LINC02860
NFE2L3
CBX3
SKAP2
SNX10
HIBADH
TAX1BP1
EVX1
JAZF1
RNU6-979P
JAZF1-AS1
CREB5
DQ601810
CTB-113D17.1
TRIL
CPVL
HOXA cluster
b
Scale
1 Mb
hg19
chr7:24,000,000
24,500,000
25,000,000
25,500,000
26,000,000
26,500,000
27,000,000
27,500,000
DECIPHER CNVs (Related to Table 2-2)
277591
259691
396369
381947
250623
396373
394278
287225
293884
394346
395309
2585
259698
250013
262899
256892
258192
304283
402045
256413
337194
HIRE2
HIRE1
Mouse GCR Deletion
CFSE
Cons 100 Verts
gnomAD CNVs (healthy subjects)
HOXA cluster
STK31
NPY
MPP6
GSDME
OSBPL3
CYCS
C7orf31
NPVF
U3
AC003090.1
RNU6-16P
MIR148A
CBX3
NFE2L3
HNRNPA2B1
SNX10
KIAA0087
LINC02860
SKAP2
EVX1
HIBADH

Figure S24. a. Copy number variants from DECIPHER database overlapping the putative novel craniofacial superenhancer region (black box with solid outline). CNVs represented by green bars have a noted phenotype but do not include a specifically described craniofacial phenotype, purple bars have a specifically described craniofacial phenotype and black bars have no phenotype reported. b. Enlargement of region in box with dotted outline. The orthologous positions removed in the GCR deletion mouse are indicated by a green bar. The orthologous positions to HIRE1 and HIRE2 deleted by Kessler et al are shown in shades of blue. Craniofacial superenhancers identified in this study are indicated by large orange bars. The track for gnomAD CNVs, filtered for CNVs >300bp appears below the DECIPHER CNV bars, blue represents gains and red losses. 300bp based on typical size range of CNVs identified in healthy human populations (Zarrei et al., 2015). A region with a notable lack of CNVs in gnomAD subjects is marked by a yellow triangle.

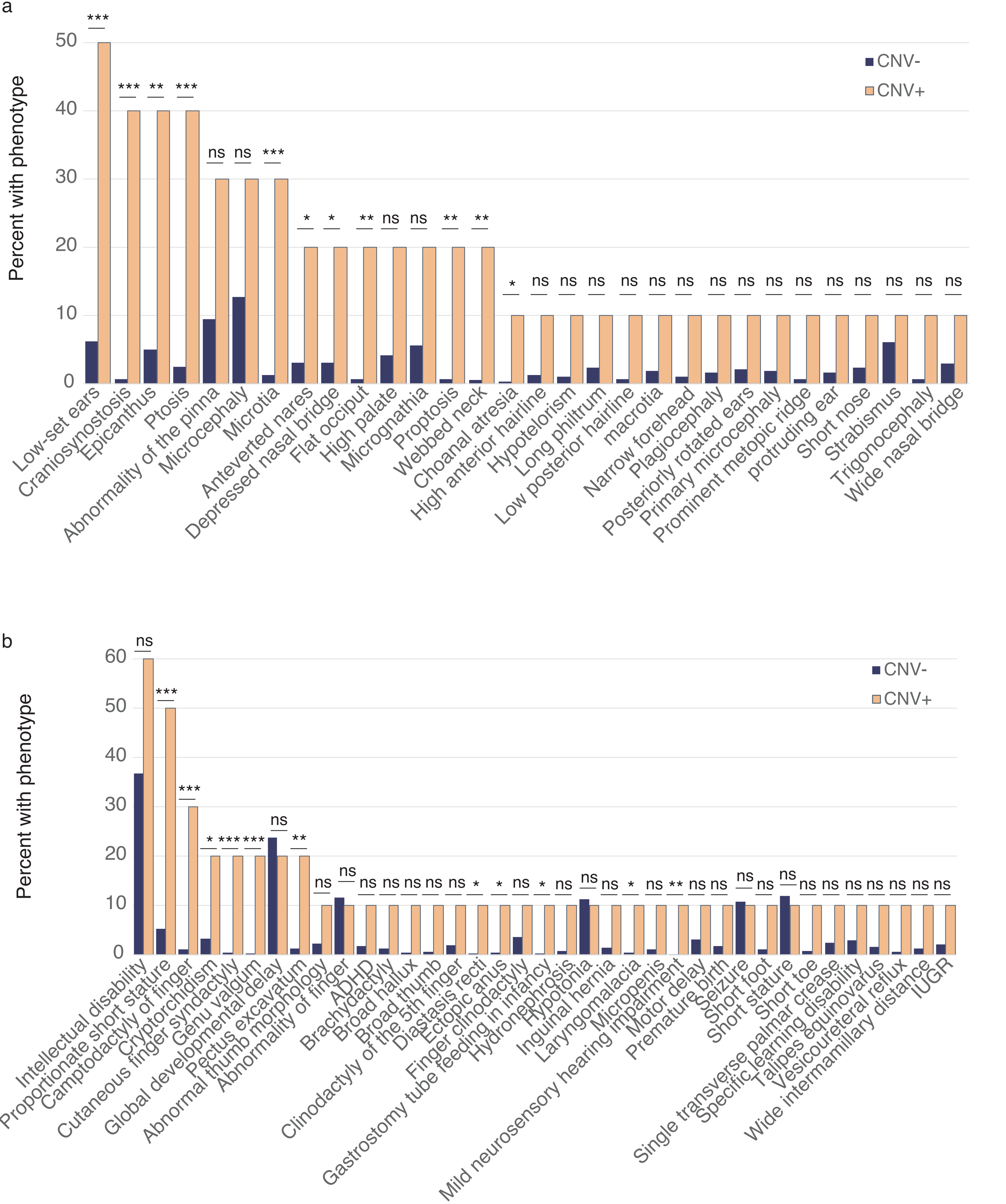
a
Percent with phenotype
50
40
30
20
10
0
CNV-
CNV+
*** *** ** *** ns ns *** * * ** ns ns ** ** * ns ns ns ns ns ns ns ns ns ns ns ns ns ns ns
Low-set ears
Craniosynostosis
Epicanthus
Ptosis
Abnormality of the pinna
Microcephaly
Microtia
Anteverted nares
Depressed nasal bridge
Flat occiput
High palate
Micrognathia
Proptosis
Webbed neck
Choanal atresia
High anterior hairline
Hypotelorism
Long philtrum
Low posterior hairline
macrotia
Narrow forehead
Plagiocephaly
Posteriorly rotated ears
Primary microcephaly
Prominent metopic ridge
protruding ear
Short nose
Strabismus
Trigonocephaly
Wide nasal bridge
b
Percent with phenotype
60
50
40
30
20
10
0
CNV-
CNV+
ns *** *** * *** *** ns ** ns ns ns ns ns ns ns * * ns * ns ns ns * ns ** ns ns ns ns ns ns ns ns ns ns ns ns
Intellectual disability
Proportionate short stature
Camptodactyly of finger
Cryptorchidism
Cutaneous finger syndactyly
Genu valgum
Global developmental delay
Pectus excavatum
Abnormal thumb morphology
Abnormality of finger
ADHD
Brachydactyly
Broad hallux
Broad thumb
Clinodactyly of the 5th finger
Diastasis recti
Ectopic anus
Finger clinodactyly
Gastrostomy tube feeding in infancy
Hydronephrosis
Hypotonia
Inguinal hernia
Laryngomalacia
Micropenis
Mild neurosensory hearing impairment
Motor delay
Premature birth
Seizure
Short foot
Short stature
Short toe
Single transverse palmar crease
Specific learning disability
Talipes equinovarus
Vesicoureteral reflux
Wide intermamillary distance
IUGR

Figure S25. Frequency of phenotypes present in individuals within the DECIPHER Database with CNVs overlapping chr7: 25,580,400-25,849,400 compared to frequency of those phenotypes in the the DECIPHER Database for CNVs not overlapping the region. Statistical test is Fisher Exact Test. * $p<0.05$, ** $p<0.01$, *** $p<0.001$

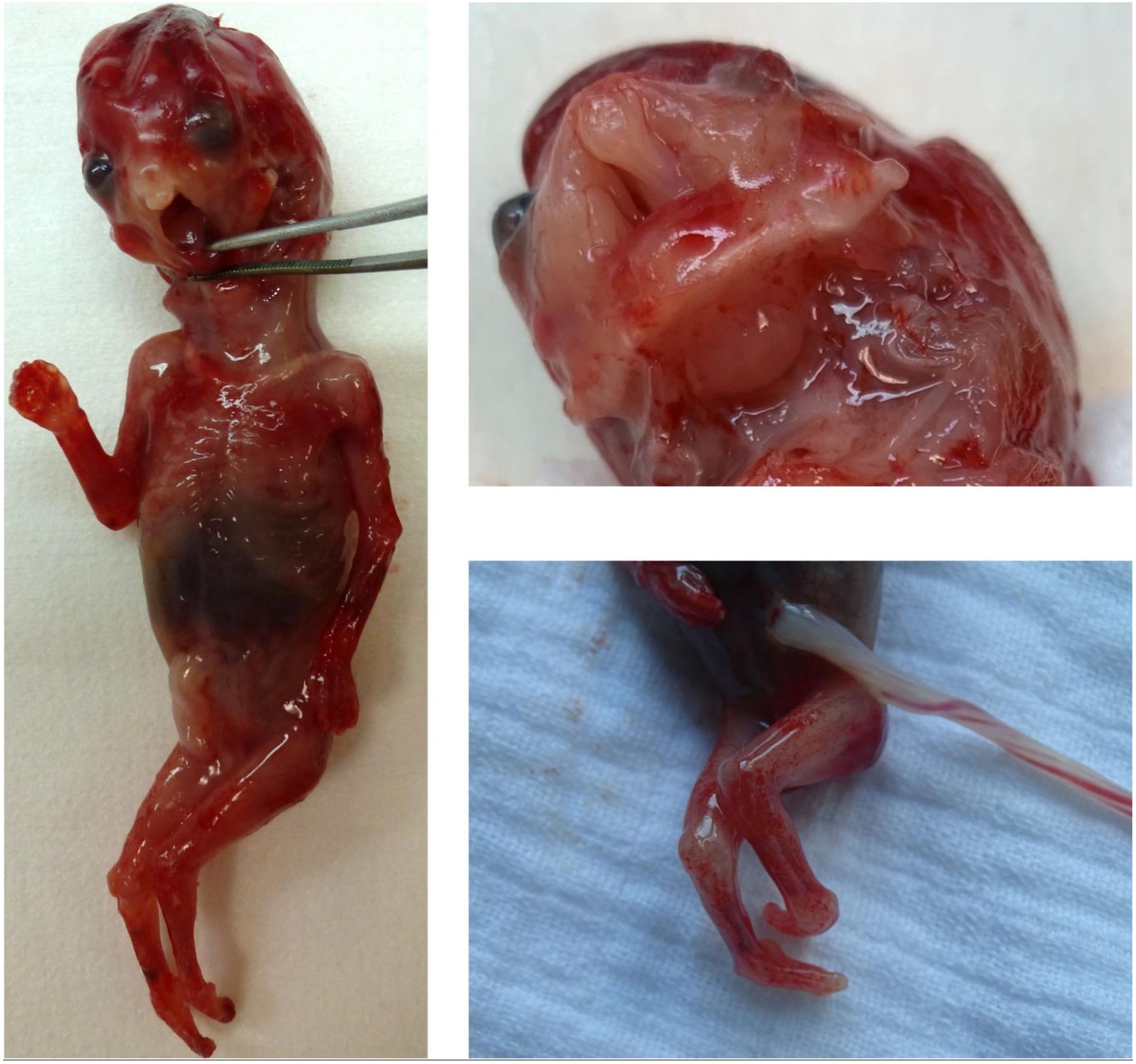

Figure S26. Autopsy images of fetus with partial deletion of the HOXA superenhancer region. The fetus displayed bilateral cleft lip and palate, an underdeveloped nose and only one nostril, clubfeet and anal atresia.

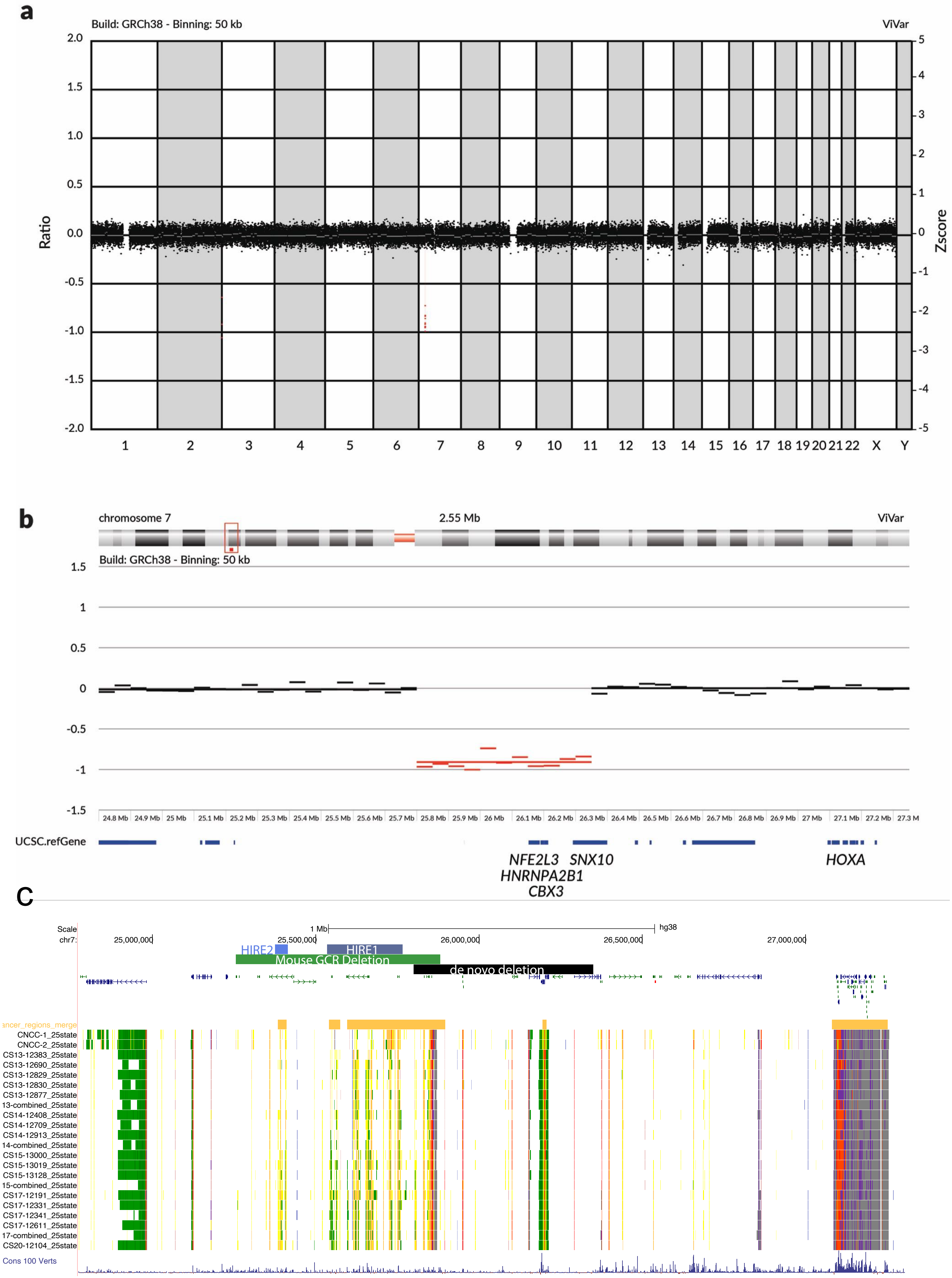
a
Build: GRCh38 - Binning: 50 kb
ViVar
Ratio
Zscore
2.0
1.5
1.0
0.5
0.0
-0.5
-1.0
-1.5
-2.0
5
4
3
2
1
0
-1
-2
-3
-4
-5
1 2 3 4 5 6 7 8 9 10 11 12 13 14 15 16 17 18 19 20 21 22 X Y
b
chromosome 7
2.55 Mb
ViVar
Build: GRCh38 - Binning: 50 kb
1.5
1
0.5
0
-0.5
-1
-1.5
24.8 Mb 24.9 Mb 25 Mb 25.1 Mb 25.2 Mb 25.3 Mb 25.4 Mb 25.5 Mb 25.6 Mb 25.7 Mb 25.8 Mb 25.9 Mb 26 Mb 26.1 Mb 26.2 Mb 26.3 Mb 26.4 Mb 26.5 Mb 26.6 Mb 26.7 Mb 26.8 Mb 26.9 Mb 27 Mb 27.1 Mb 27.2 Mb 27.3 M
UCSC.refGene
NFE2L3
SNX10
HNRNPA2B1
CBX3
HOXA
C
Scale
chr7:
1 Mb
hg38
25,000,000
25,500,000
26,000,000
26,500,000
27,000,000
HIRE2
HIRE1
Mouse GCR Deletion
de novo deletion
ancer_regions_merge
CNCC-1_25state
CNCC-2_25state
CS13-12383_25state
CS13-12690_25state
CS13-12829_25state
CS13-12830_25state
CS13-12877_25state
13-combined_25state
CS14-12408_25state
CS14-12709_25state
CS14-12913_25state
14-combined_25state
CS15-13000_25state
CS15-13019_25state
CS15-13128_25state
15-combined_25state
CS17-12191_25state
CS17-12331_25state
CS17-12341_25state
CS17-12611_25state
17-combined_25state
CS20-12104_25state
Cons 100 Verts

Figure S27- Identification of a 550kb *de novo* deletion upstream of the *HOXA* cluster through shallow whole genome sequencing and copy-number variant (CNV) analysis using ViVar. (a) whole genome lineview. (b) zoomed in on deletion (binning: 50kb). ViVar analysis and visualization (Sante et al., 2014). (c). Genome browser view of same region as in (b). At top the de novo deletion is indicated in black. The orthologous positions removed in the GCR deletion mouse are indicated by a green bar. The orthologous positions to HIRE1 and HIRE2 deleted by Kessler et al are shown in shades of blue.

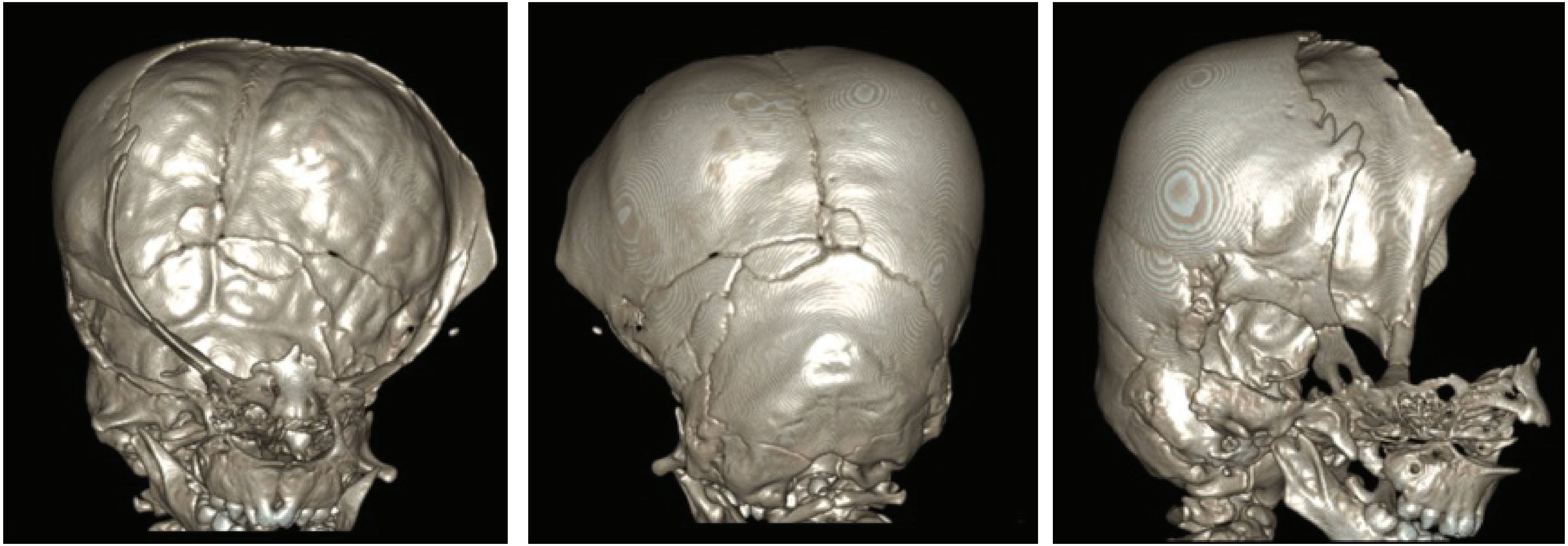

Figure S28. CT scan of patient with in Figure 9 at 18 months of age.

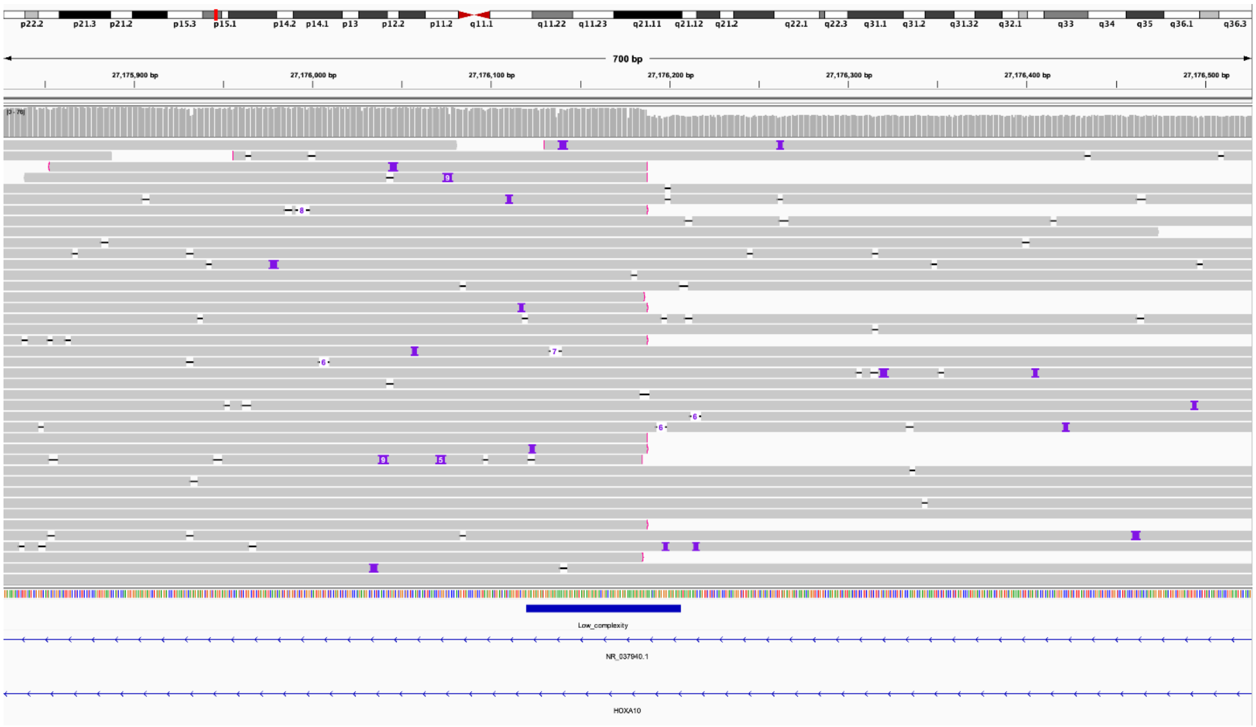

Fig S29: Targeted long-read sequencing identified the exact breakpoints of the duplication identified by clinical testing. In the IGV view shown depth of coverage (top track) shows a drop in coverage after the end of the duplication. Orientation of the mapped reads confirm that the duplication is tandem and that the breakpoint at chr7:27,176,187 lies within HOXA10 and a low-complexity GA-rich region. The breakpoint at chr7:25,220,918 is not shown.

Supplemental References

Sante, T., Vergult, S., Volders, P.-J., Kloosterman, W.P., Trooskens, G., De Preter, K., Dheedene, A., Speleman, F., De Meyer, T., and Menten, B. (2014). ViVar: a comprehensive platform for the analysis and visualization of structural genomic variation. PLoS One *9*, e113800.
